# Guttation capsules containing hydrogen peroxide: an evolutionarily conserved NADPH oxidase gains a role in wars between related fungi

**DOI:** 10.1111/1462-2920.14575

**Published:** 2019-04-22

**Authors:** Jian Zhang, Youzhi Miao, Mohammad Javad Rahimi, Hong Zhu, Andrei Steindorff, Sabine Schiessler, Feng Cai, Guan Pang, Komal Chenthamara, Yu Xu, Christian P. Kubicek, Qirong Shen, Irina S. Druzhinina

**Affiliations:** ^1^ Jiangsu Provincial Key Lab of Organic Solid Waste Utilization Nanjing Agricultural University Nanjing China; ^2^ Microbiology and Applied Genomics Group, Institute of Chemical Environmental and Bioscience Engineering (ICEBE), TU Wien Vienna Austria; ^3^ US Department of Energy Joint Genome Institute Walnut Creek CA USA; ^4^ Steinschoetelgasse 7,1100 Vienna Austria

## Abstract

When resources are limited, the hypocrealean fungus *Trichoderma guizhouense* can overgrow another hypocrealean fungus *Fusarium oxysporum*, cause sporadic cell death and arrest growth. A transcriptomic analysis of this interaction shows that *T. guizhouense* undergoes a succession of metabolic stresses while *F. oxysporum* responded relatively neutrally but used the constitutive expression of several toxin‐encoding genes as a protective strategy. Because of these toxins, *T. guizhouense* cannot approach it is potential host on the substrate surface and attacks *F. oxysporum* from above. The success of *T. guizhouense* is secured by the excessive production of hydrogen peroxide (H_2_O_2_), which is stored in microscopic bag‐like guttation droplets hanging on the contacting hyphae. The deletion of NADPH oxidase *nox1* and its regulator, *nor1* in *T. guizhouense* led to a substantial decrease in H_2_O_2_ formation with concomitant loss of antagonistic activity. We envision the role of NOX proteins in the antagonism of *T. guizhouense* as an example of metabolic exaptation evolved in this fungus because the primary function of these ancient proteins was probably not linked to interfungal relationships. In support of this, *F. oxysporum* showed almost no transcriptional response to *T. guizhouense Δnox1* strain indicating the role of NOX/H_2_O_2_ in signalling and fungal communication.

## Introduction

In fungal communities, most interactions are combative (Jeffries and Young, 1994; Divakar *et al*., 2015). Heterotrophic nutrition led to the evolution of fungi as saprotrophs (feeding on dead organic matter) or biotrophs (deriving nutrients from living organisms in symbiosis). Many symbiotic fungi are also capable of killing their partner, continuing to feed on its remains (Jeffries and Young, 1994; Reynolds and Currie, 2004) and then also using their foraging ground (Hiscox and Boddy, 2017; Hiscox *et al*., 2018). Interkingdom symbiotic interactions of fungi are relatively well studied (Kugler *et al*., 2000; Sebghati *et al*., 2000; Bouwmeester *et al*., 2007; Tarkka *et al*., 2009). Nevertheless, despite the thousands of known cases (Hawksworth, 1981), interfungal biotrophic relations have received scant attention (Jeffries and Young, 1976; Jeffries, 1995; Goh and Vujanovic, 2010b). The territorial ‘wars’ of wood‐degrading fungi are studied from the perspective of lignocellulose decomposition processes and ecosystem modelling (Hiscox *et al*., 2018). The other sector of interest in fungus‐fungus opposition is the interaction between so‐called plant‐beneficial fungi and plant pathogens. For example, if conditions are appropriate, practically any species of *Trichoderma* (Hypocreales, Ascomycota) can directly attack a given plant pathogenic fungus, inhibit its growth by chemicals or beat it in a competition for the resources (Komon‐Zelazowska *et al*., 2007; Jaklitsch, 2009; Seidl *et al*., 2009; Jaklitsch, 2011; Atanasova *et al*., 2013; Steindorff *et al*., 2014; Zhang *et al*., 2016; Druzhinina *et al*., 2018). The multitude of these *Trichoderma* interactions is used in the biological control of fungal pests (biocontrol) (Druzhinina *et al*., 2011).

Contacts between fungi are difficult to record in nature. They are usually observed when some fungi grow on the thalli of the other fungi (Douhan and Rizzo, 2003; Tamm and Poldmaa, 2013). Although, it is challenging to prove true parasitic relationships (the transfer of nutrients from a host to the parasite), these interactions are commonly classified as mycoparasitic (Jeffries and Young, 1976; Jeffries, 1995). Contrary to antagonism and agonism (Chenthamara and Druzhinina, 2016), mycoparasitism should involve a prolonged contact stage between hyphae. Some fungi are obligate parasites and have specialized structures that allow them to invade the host (Goh and Vujanovic, 2010a; Marfetan *et al*., 2015). However, more fungi are facultative mycoparasites (Jeffries and Young, 1994). In given conditions, numerous plant pathogenic fungi such as *Fusarium oxysporum* (Hypocreales, Ascomycota) or *Rhizoctonia solani* (Cantharellales, Basidiomycota) are also potent facultative mycoparasites (see refs in Jeffries and Young, 1994). This strategy can be commonly selected as an alternative to a ‘deadlock’ when a fungus becomes restricted in its feeding territory by its neighbours. To find a new substrate, it attacks its neighbour(s) and feeds on its body (Hiscox *et al*., 2018) or grows through it to the new feeding ground (Ujor *et al*., 2018).

As fungi have primitive bodies with limited attributes, their battles, defences and other interactions strongly depend on chemical warfare (Hiscox and Boddy, 2017). After combatant recognition, a fungus can change its primary metabolism, growth, secondary metabolite production and stress mitigation responses. The subsequent events (the penetration or killing and consumption of the host) are believed to be due to the activity of extracellular enzymes that can lyse the fungal cell wall and the formation of fungistatic or fungicidal toxins. In the case of facultative mycoparasites (when the feeding ground is of primary importance compared to the fungivory *per se*), chemical warfare may occur as a constitutive defence through the deposition of antimicrobial compounds within the substrate (Boddy, 2000), thus making the surrounding habitat not useful for competitors. The diversity of secondary metabolites with antifungal properties is vast and includes volatile and diffusible organic compounds (VOCs and DOCs respectively) (Evans *et al*., 2008; El Ariebi *et al*., 2016). A few fungi also produce reactive oxygen species (ROS), in particular hydrogen peroxide (H_2_O_2_), upon contact with another fungus (Silar, 2005). However, the involvement of ROS in interfungal interactions remains unclear (Hiscox and Boddy, 2017).

## Results

### T. guizhouense *overgrows Foc4 but does not kill it*

**Figure 1:**
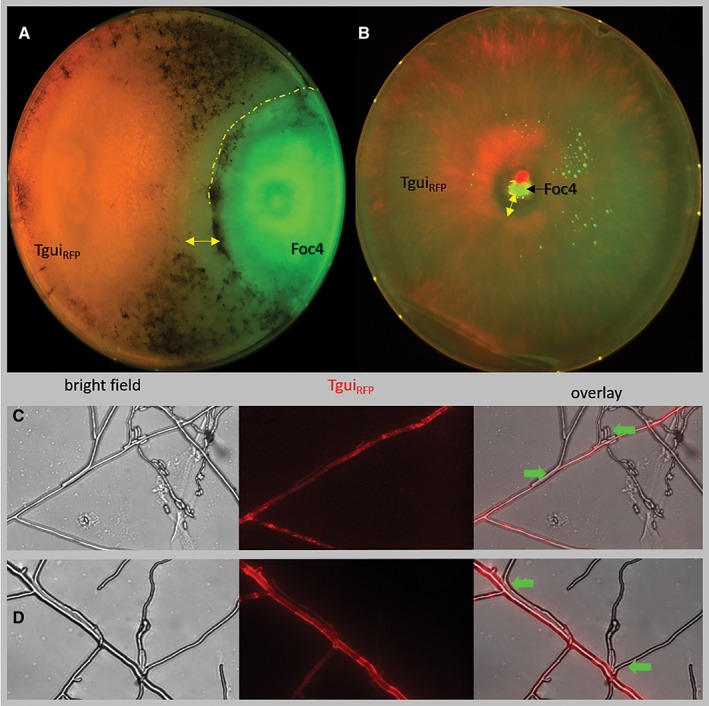
*In vitro* interaction between *T. guizhouense* NJAU 4742 and *F. oxysporum*. A. A dual confrontation assay between RFP‐labelled Tgui and Foc4. The image was taken in ChemiDoc (Bio‐Rad, CA, USA) after 6 days of incubation at 25°C in darkness on GSM covered with cellophane in a 9 cm Petri plate. Tgui conidia are visible as black powder. The dashed line shows the extension of a Foc4 colony; the double arrow indicates the antibiosis zone. B. A co‐culture assay imaged 6 days after the simultaneous inoculation of the plate as in A with the conidia of Tgui and Foc4. C, D. Microscopic investigation (x 400) of the non‐trophic hyphae of Tgui_RFP_/Foc4 interaction on the glass surface. The arrows point to the cases of Foc4 hyphae specifically growing along Tgui_RFP_ hyphae. More images and control self‐confrontations are shown in Supporting Information S2. [Color figure can be viewed at http://wileyonlinelibrary.com]

While investigating the interactions between *Trichoderma* spp. and other hypocrealean fungi (Chenthamara and Druzhinina, 2016; Druzhinina *et al*., 2018), we noticed that a strain of *Fusarium oxysporum* f. sp. *cubense* 4 TUCIM 4848 (Foc4), was highly resistant to antagonism by various *Trichoderma* species, and only *Trichoderma guizhouense* NJAU 4742 (Tgui) repeatedly overgrew it in dual confrontation assays (Supporting Information S1). Other *Trichoderma* strains *s*howed weak and unstable antagonism, or could not combat Foc4 and remained in a ‘deadlock stage’ (the growth of a fungus becomes arrested by its neighbours, *see above*) with no hyphal interaction. Therefore, we focused on the relationships between Tgui and Foc4. The ability to overgrow suggests mycoparasitism but does not prove it. As both partner species are facultative mycoparasites (Vujanovic and Goh, 2010; Chaverri *et al*., 2015), we first had them confront each other on a glass surface using a transformant of Tgui labelled with RFP (Tgui_RFP_). It showed that at the initial stage of the interaction between the non‐trophic hyphae (on the surface of a glass slide), these fungi had affinity for each other with no evident antagonism (Fig. 1). When the two fungi were inoculated together in the centre of a Petri plate, Tgui rapidly covered the plate, while Foc4 developed slower, replacing the oldest part of the Tgui colony. The formation of an antibiosis zone surrounding Foc4 was noticed (Fig. 1). The development of Foc4 stopped at a radius of < 1–1.5 cm, probably due to inhibition by volatile compounds produced by Tgui (Supporting Information S2).

**Figure 2:**
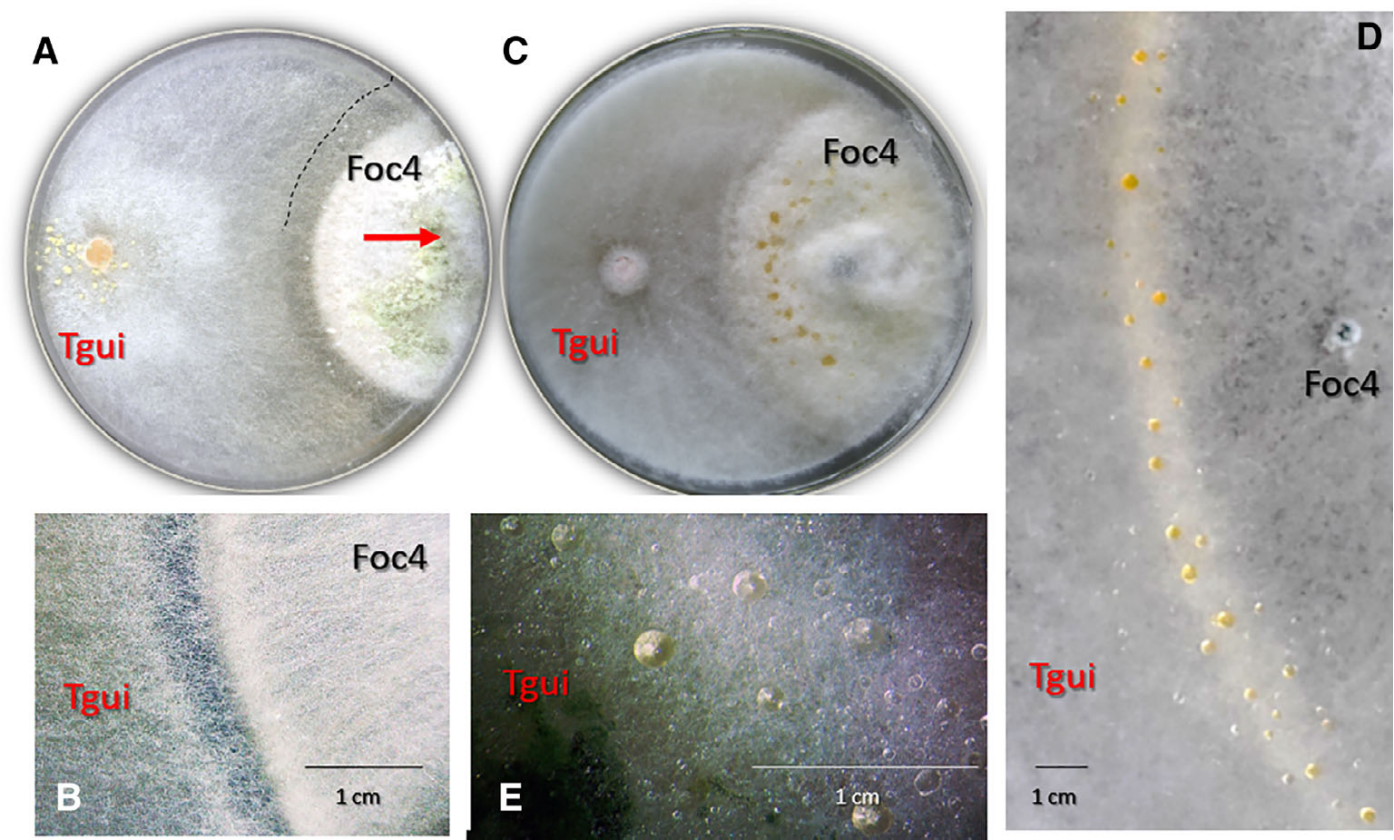
Guttation droplets at the contact zone between *T. guizhouense* and *F. oxysporum*. A, B. Morphology of the contact zones in dual confrontation assays between Tgui and Foc4 after 6 days of incubation at 25°C in darkness. The dashed line in A shows the extension of the antibiosis zone that is magnified in B. The arrow shows the Tgui overgrowth of Foc4. C–E Guttation drops at the contact and overgrowth areas imaged at ten days of incubation. Petri plate diameter is 9 cm. [Color figure can be viewed at http://wileyonlinelibrary.com]

The long‐term observation of the confrontations between Tgui and Foc4 on cellophane‐covered agar plates (i.e. in a closed system with limited resources) revealed a combative interaction that can be divided into three stages: first, both fungi showed normal radial growth with no indications of a response to the presence of each other. However, when the colonies came in contact (48–52 h), a deadlock phase lasting for up to 12 h was observed. During this period, the growth of both fungi stopped, and an antibiosis zone that surrounded the Foc4 colony was formed (Fig. 2A and B). This behaviour is characteristic for the formation of a fungicidal compound by Foc4. At the last stage, Tgui produced abundant aerial hyphae and conidiophores and overgrew Foc4 (Figs 1A, 2A and B, Supporting Information S2). Tgui did not penetrate the mycelial mat of Foc4 to reach the substrate beneath it but formed a dense mycelial net above Foc4 (Fig. 2, Supporting Information S2 13–14). The application of trypan blue, which stains cells with a damaged plasma membrane (Silar, 2005), demonstrated the loss of cell integrity of some Foc4 hyphal compartments (cells), while the Tgui mycelia were largely intact (Supporting Information S2). We noticed that even after prolonged incubation, the complete and abundant overgrowth of Foc4 by Tgui did not result in death of the *Fusarium*. In contrast, this fungus occupied the surface of the Petri plate and formed microconidia (Supporting Information S2 9).

### *The combative interaction between* T. guizhouense *and* F. oxysporum *is accompanied by the formation of abundant droplets on the aerial hyphae*

**Figure 3:**
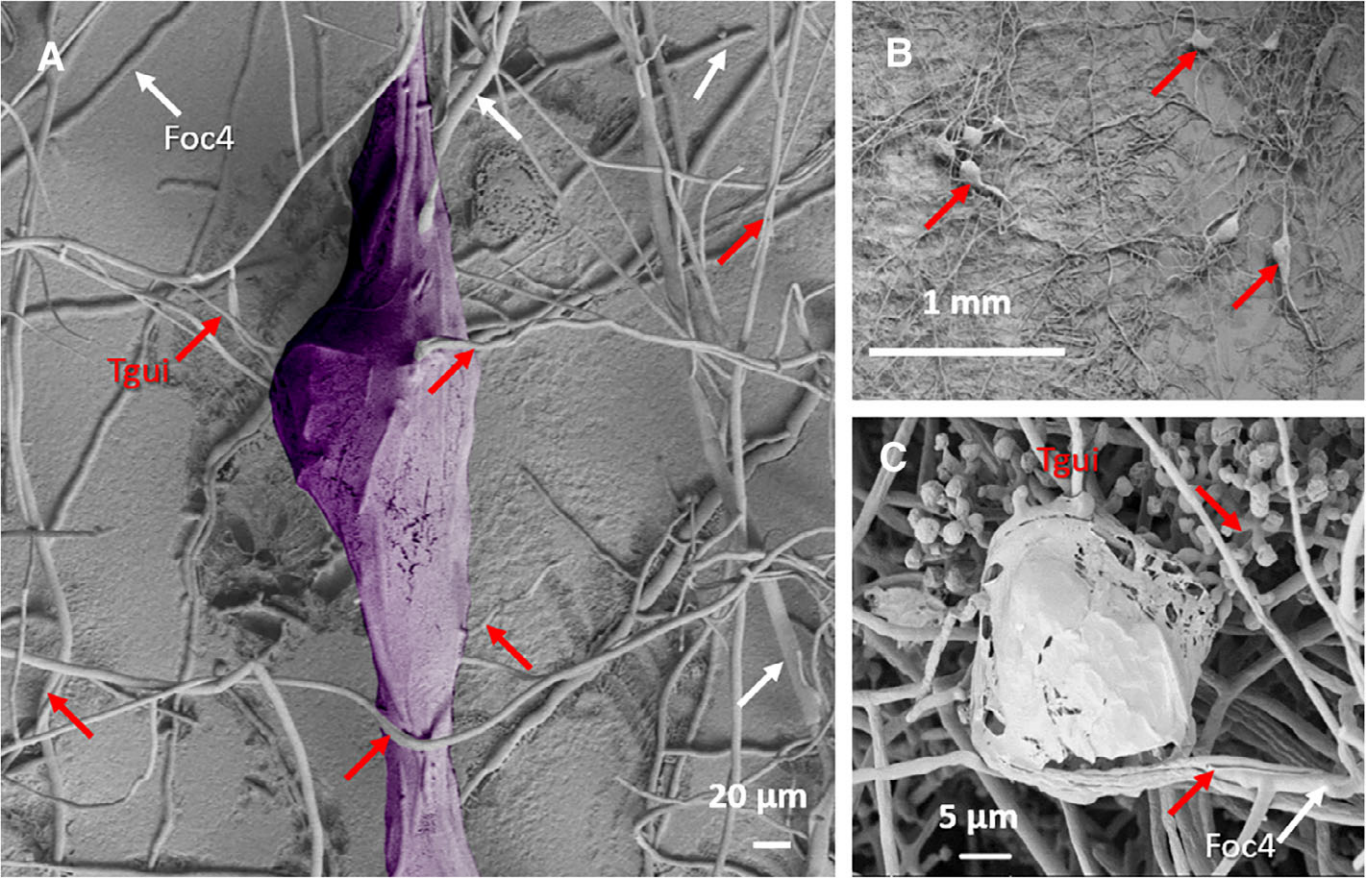
Microscopic bag‐like guttation capsules at the contact zone between *T. guizhouense* and *F. oxysporum*. Cryo‐SEM images of the bag‐like guttation capsules in the interaction zone (shortly after contact) on the surface of the cellophane‐covered GSM. A. A magnified image of a bag‐like guttation droplets (guttation capsule) hanging on the contacting aerial hyphae of Tgui and Foc4. B. The location of guttation capsules on aerial hyphae in the contact zone shortly after Tgui started to overgrow Foc4. C. The mature and partially destroyed guttation capsule on the aerial hyphae of Foc4 and Tgui new the plug of Foc4. The overgrowth by Tgui is evidenced by the respective conidiophores on the backside of the image. White arrows point to Foc4, while red arrows indicate Tgui hyphae or conidia. For each image the attribution of the hyphae to the species was based on the tracing individual hyphae to the plug. A is artificially coloured for clarity; the original image is presented in Supporting Information S2. [Color figure can be viewed at http://wileyonlinelibrary.com]

The Tgui overgrowth of Foc4 was accompanied by the formation of bright yellow macroscopic guttation droplets at the contact area (Fig. 2C–E). The application of cryo‐SEM allowed us to observe also microscopic droplets on the interacting hyphae (Fig. 3). Impressively, the droplets were covered by a film that made the structures resemble plastic bags filled with liquid. The film became particularly visible when advanced interaction stages were examined (Supporting Information S2 2–8). We term these microscopic structures guttation capsules throughout the paper to acknowledge their bag‐like appearance (Fig. 3, see Supporting Information S2 for figures). The formation of the film on the surface of the macroscopic yellow guttation droplets was not apparent. Thus, the guttation capsules are likely specific structures for interactions between the aerial hyphae, while yellow droplets reflect the general guttation that takes place during the interaction. Guttation droplets were also rarely visible on the surface of Tgui where no interactions with Foc4 took place.

As the guttation droplets are putatively linked to the interactions with Foc4 (*see below*), we analysed their content chemically and found the activity of chitinolytic and proteolytic enzymes (Supporting Information S3, Table S3‐1). However, neither n‐butanol extract nor enzymatic cocktails extracted from the content of guttation droplets did essentially influence Foc4 (Supporting Information S3 2).

### *The high concentration of H_2_O_2_ in guttation droplets is produced by* T. guizhouense

We also detected a high concentration of H_2_O_2_ (up to 4.77 ± 0.36 mM) in the content of the guttation droplets. We should note that the true biological concentration of H_2_O_2_ was likely higher, as sampling was performed under ambient illumination and room temperature, which could result in partial degradation of the compound.

**Figure 4:**
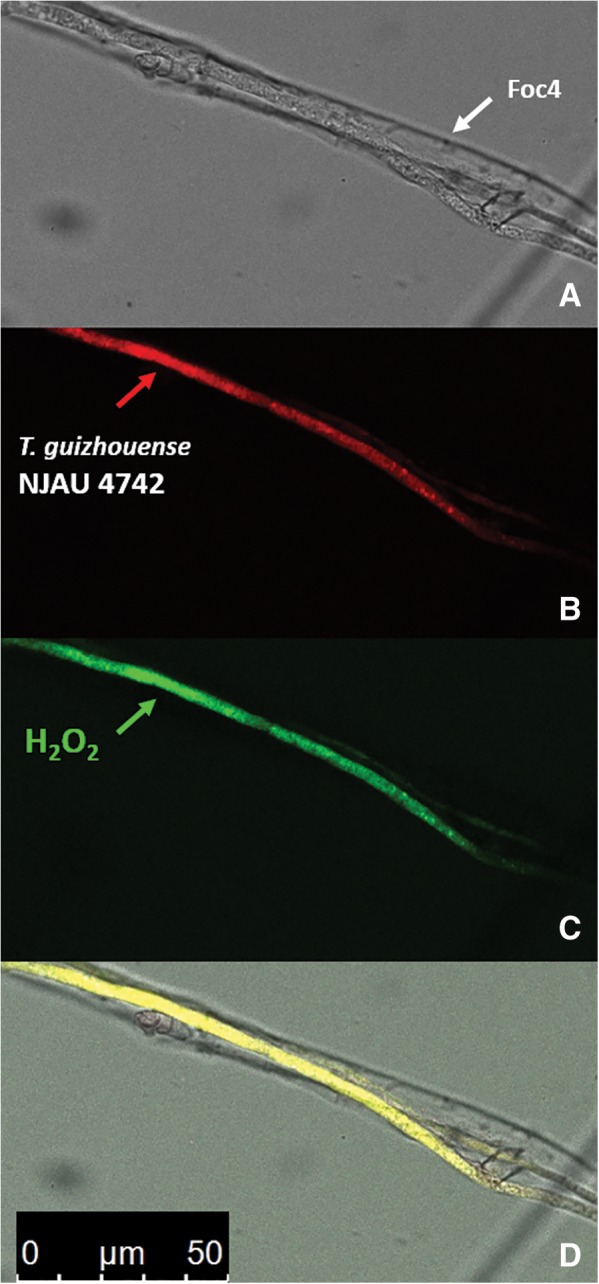
Production of H_2_O_2_ by the hyphae of Tgui interacting with Foc4 on the glass slide. A. Bright field microscopy. B, C. Dark field microscopy. D. An overlay. The observation was performed on the aerial hyphae collected from the interaction zone (A) and incubated in the presence of 2.5 μg/ml H_2_DCFDA for 10 min Leica DMi8 fluorescence microscope (Germany; see Experimental procedures for more details). The red fluorescently labelled Tgui_RFP_ transformant and 571 nm emission was used for the identification of *Trichoderma* partner, Foc4 hyphae were not fluorescent (B). The production of H_2_O_2_ was detected as green fluorescence (C) at 460 to 490 nm excitation and 500 to 550 nm emission. [Color figure can be viewed at http://wileyonlinelibrary.com]

To demonstrate the formation of H_2_O_2_ by the hyphae of Tgui, we confronted the fungi using the Tgui_RFP_ mutant and H_2_DCFDA, which reacts with H_2_O_2_ resulting in a green fluorescent product (Fig. 4). The shape of the stained H_2_O_2_ overlaps with the fluorescently labelled hyphae of Tgui_RFP_, and no stain was found on Foc4 hyphae. We, therefore, conclude that Tgui can form H_2_O_2_.

### *H_2_O_2_ is toxic to* F. oxysporum *and other fungi, while* T. guizhouense *is resistant to it*

When present in the medium, H_2_O_2_ inhibits the growth of Foc4 and other fungi (Supporting Information S4). The use of propidium iodine exclusion, which detects dead and dying cells (Ment *et al*., 2012), revealed that the H_2_O_2_ concentration‐dependent decrease in the growth of Foc4 was paralleled by a significant loss in the viability of mycelia and spores (Supporting Information S4).

### T. guizhouense *has several ways to produce H_2_O_2_, while the ROS arsenal of* F. oxysporum *is characterized by an abundant production of superoxide*

H_2_O_2_ formation by fungi can occur via two main metabolic reactions: one as a by‐product of oxidation by FAD‐dependent oxidases such as glucose oxidase or amino acid oxidases (Stosz *et al*., 1996; Murray *et al*., 1997; Brunner *et al*., 2005; Yang *et al*., 2011; Smirnova *et al*., 2017), and one via the hydrolysis of O_2_^•−^ by superoxide dismutases (SODs) (Heller and Tudzynski, 2011). To test for the first reaction, we investigated the transcriptome of Tgui grown in confrontation with Foc4 before and after contact for the expression of genes encoding FAD‐dependent oxidases (Supporting Information S5). Indeed, we found 27 constitutively expressed genes, four of which had their expression moderately increase after contact with Foc4. None of the proteins encoded by these 27 enzymes has yet been characterized with respect to their substrates, but the data show that Tgui has the theoretical capacity for the production of H_2_O_2_ via FAD‐dependent oxidases.

**Figure 5:**
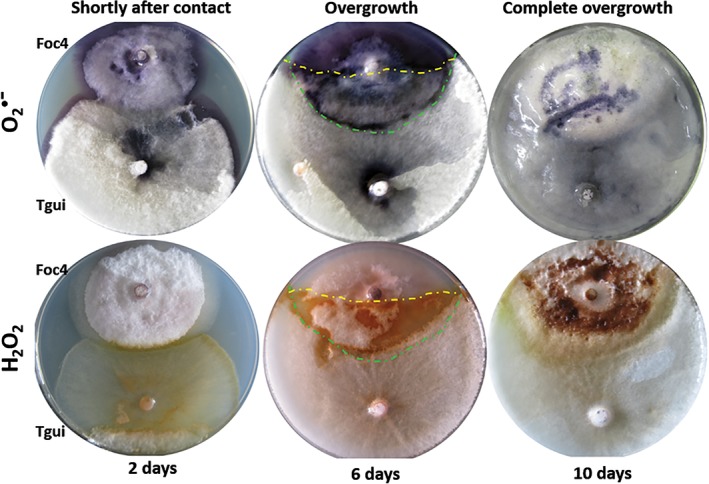
Production of ROS by Tgui and Foc4 during the dual confrontation assays on GSM. Superoxide is stained in dark blue, and H_2_O_2_ is stained in reddish brown. At sixth day, yellow and green lines indicate the extension of Tgui and Foc4 colonies respectively. O_2_^•−^ is stained dark blue by nitro blue tetrazolium, H_2_O_2_ is visualized as the reddish brown colour that developed due to the presence of diaminobenzidine and horseradish peroxidase. The O_2_^•−^ accumulation appeared to be independent on the interaction between the two fungi and specific to the feeding (substrate) hyphae. The production of H_2_O_2_ was associated with aerial hyphae when Tgui contacted and overgrown Foc4. Petri plate diameter is 9 cm. [Color figure can be viewed at http://wileyonlinelibrary.com]

To investigate the alternative hypothesis that H_2_O_2_ could be formed from O_2_^•−^, we used nitro blue tetrazolium, which reacts to form a deep blue colour at the sites of O_2_^•−^ accumulation (Fig. 5). This assay suggests that Foc4 produces O_2_^•−^ all over the colony independent on the interaction. In agreement with this hypothesis, the transcriptomic data revealed three SODs [an iron‐dependent SOD (OPB42955), a copper‐zinc‐dependent SOD (OPB51595) and a manganese‐dependent SOD (OPB 44914)] to be constitutively expressed in Tgui (Supporting Information S5). The level of the manganese‐dependent SOD moderately increased after Tgui contact with Foc4. The respective genes in Foc4 were constitutively expressed at a low level. The upregulation of peroxisomal catalase (EMT6711) is probably related to the general metabolism of the fungus. An SOD (EMT66208) is expressed relatively strongly but independently of the interaction. However, the intensive production of H_2_O_2_ in Tgui is stimulated by the interaction with Foc4.

### *The NADPH oxidase NOX1 is essential for* T. guizhouense *interaction with Foc4*

**Figure 6:**
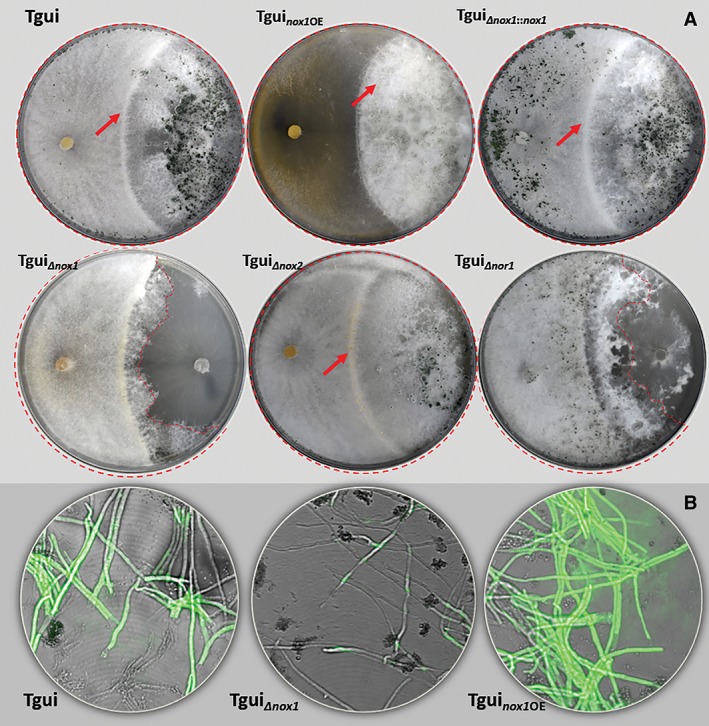
Phenotype of *nox* mutants of Tgui and their ability to produce H_2_O_2_. A. Dual confrontation assays with Tgui *nox* mutants and Foc4 after 7 days of incubation at 25°C in darkness. Petri plate diameter is 9 cm. The red line indicates the expansion of Tgui mycelia. Red arrows show the formation of guttation droplets. B. Qualitative assessment of H_2_O_2_ production by *nox* mutants of Tgui. The H_2_DCFDA assay was performed with hyphae cultivated in PD broth. [Color figure can be viewed at http://wileyonlinelibrary.com]

Our data suggest that the production of H_2_O_2_ can be involved in the ability of Tgui to overgrow Foc4. To test this possibility, we used a reverse genetics approach. In Ascomycota, two NADPH oxidases (NOX1 and NOX2) and their regulator gene (NOR1) are involved in H_2_O_2_ formation (Cano‐Dominguez *et al*., 2008, Hernandez‐Onate *et al*., 2012). We, therefore, constructed Tgui mutants for each of these genes (termed Tgui_*Δnox1*_ *,* Tgui_*Δnox2*_ and Tgui_*Δnor1*_ respectively; verified by PCR and Southern blot analysis (Supporting Information S6) and tested them for interactions with Foc4, the formation of guttation capsules and H_2_O_2_ production (Supporting Information S7). As shown in Fig. 6A, the Tgui_*Δnox1*_ and Tgui_*Δnor1*_ strains lost their ability to efficiently overgrow Foc4, whereas Tgui_*Δnox2*_ exhibited the same antagonistic vigour as the parental strain. The qualitative assays by H_2_DCFDA showed that the ability of Tgui_*Δnox1*_ to produce H_2_O_2_ was diminished but not abandoned (Fig. 6B).

**Figure 7:**
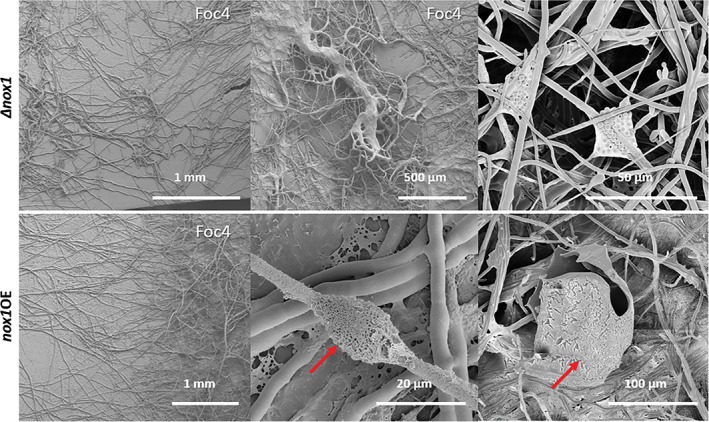
Cryo‐SEM analysis of Tgui *nox* mutants after contact. Interacting mycelia were imaged on the surface of cellophane‐covered GSM shortly after contact. Red arrows point to the guttation capsules seen on the *nox1*OE strain. [Color figure can be viewed at http://wileyonlinelibrary.com]

Cryo‐SEM analysis revealed no guttation capsules in the contact zone between Tgui_*Δnox1*_ and Foc4 (Fig. 7). At a few places, large hyphal knots mainly contained Tgui hyphae. The older colonies had numerous abnormally thin hyphae and spores aggregated in bag‐like structures (Supporting Information S7). However, none of these structures were involved in the interaction with Foc4. The *nox1* overexpressing strain Tgui_*nox1*OE_ formed structures similar to the guttation capsules of Tgui, but their surface was damaged. Numerous droplets were observed on single hyphae (no contact with Foc4). The parental phenotype was recovered when Tgui_*Δnox1*_ was complemented by the respective wild‐type gene (Fig. 6).

We conclude that NOX1 is responsible for some (probably the most part of it) H_2_O_2_ formed by Tgui in combative interaction with Foc4.

### *NOX1 is involved in the combative and defensive responses of* T. guizhouense *when it is confronted with* F. oxysporum

**Figure 8:**
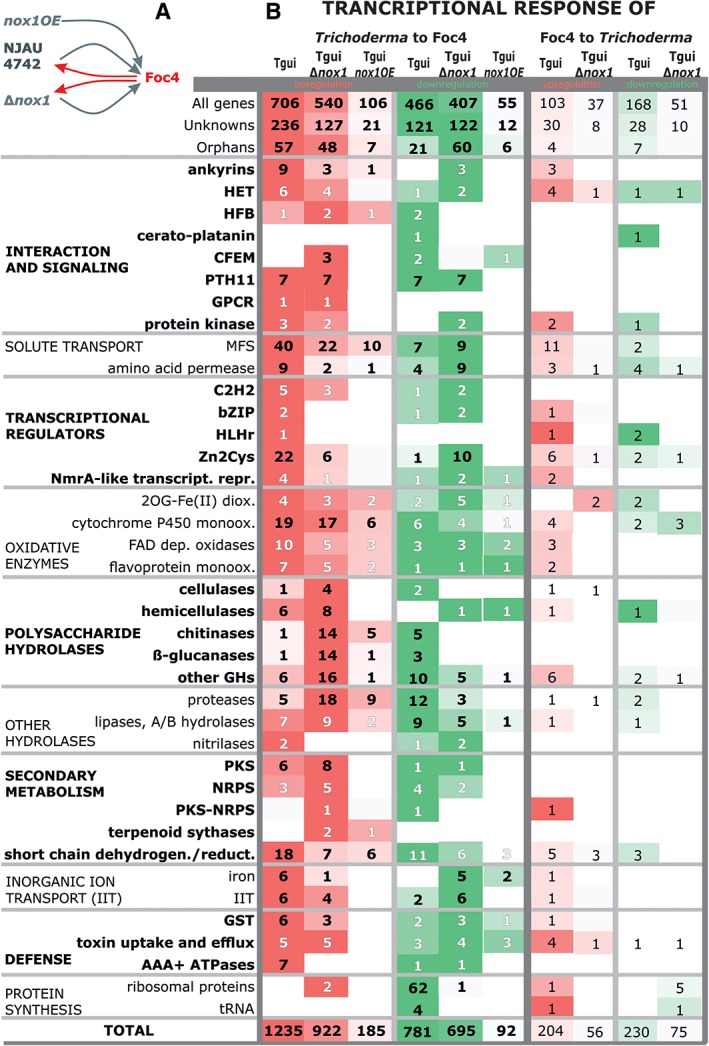
Transcriptional response of Tgui, Tgui_*Δnox1*_ and Foc4 to the combative interaction in dual confrontation assays. A. The scheme showing the transcriptomic experiment design. B. Transcriptional response of both fungi to the presence of the other partner. Shades of red and green highlight the number of genes per gene family involved in the interaction that were upregulated and downregulated respectively. The complete transcriptional response is presented in Supporting Information S5. For the transcriptional response of *Trichoderma* to Foc4, the numbers in black show the gene families or groups where the difference between Tgui and the mutants (either one or both) was confirmed by the statistical analysis of variance (ANOVA) with *p* < 0.05. Numbers in white correspond to the case where the difference was not significant. The details on the statistical analysis and the total number of genes per each family are provided in the Supporting Information S5. For Foc4 no data available. [Color figure can be viewed at http://wileyonlinelibrary.com]

The genome of Tgui harbours 11 255 genes (Druzhinina *et al*., 2018). When the fungus was in contact with Foc4, 706 genes were found to be upregulated (log_2_ > 2; *p* < 0.05) and 466 genes downregulated (log_2_ < 2; *p* < 0.05). An enrichment analysis based on GO_MWU (https://github.com/z0on/GO_MWU) revealed significant changes in the expression of a number of GO categories related to transport, membrane function, polysaccharide degradation and proteolysis (Supporting Information S8). A detailed inspection of these genes and aligning the encoded proteins to functional groups (FunCat) (Ruepp *et al*., 2004) and published gene families (e.g. CAZymes, proteases or secondary metabolites; see Experimental Procedures for details) revealed that 72% of these transcripts represented either several genes from a single family/group, or occurred in one of the groups reported to be involved in interfungal interactions (such as polysaccharide hydrolases, secondary metabolism, defence, interaction and signalling; reviewed in Druzhinina *et al*., 2011), or represented unknown or orphan genes (Fig. 8). Most of the remaining differentially upregulated genes were categorized as ‘metabolism’, ‘recombination’ and ‘transcription’, but they were broadly scattered as single members among different functions or gene families and, therefore, not investigated further (Supporting Information S5). In Tgui, the highest numbers of transcripts within the specific groups and families of upregulated genes comprised unknown proteins, orphans, permeases of the major facilitator superfamily (MFS), cytochrome P450 monooxygenases and short‐chain dehydrogenases/reductases with unknown substrate specificities. All of them were strongly downregulated in the Tgui_*Δnox1*_ strain, but transcribed at comparable levels in Tgui_*nox1*OE_ (Fig. 8). The same observation was made with a number of genes [polyketide synthases (PKS), iron uptake, transport of inorganic ions (other than iron), glutathione‐S‐transferase (GST), PTH11‐type receptors, proteases, AAA+ ATPases, ankyrins and amino acid permeases] of which only a small number was expressed in Tgui, but no expression could be detected in Tgui_*Δnox1*_. Interestingly the metalloprotease *nmp1*, which is important for the interaction of Tgui and Foc4 (Zhang *et al*., 2016) was not present among these genes because its upregulation was below the threshold used in this analysis (log_2_ > 2). These findings suggest that a functional *nox1* gene is required for several competitive reactions such as detoxification, antibiosis, uptake of nutrients and recognition. Interestingly, the transcription of genes encoding extracellular hydrolytic enzymes such as glycoside hydrolases (GH5 and GH12 cellulases, GH18, GH20 and GH75 chitinolytic enzymes, GH16, GH17, GH64 and GH81 ß‐glucanases and GH23 peptidoglycan hydrolases) and proteases, were only little affected in Tgui_*Δnox1*_ but strongly upregulated in Tgui_*nox1*OE_. An upregulation of some glycoside hydrolases has also been observed during interaction of *T. harzianum* with *Pythium ultimum* (Oomycota) (Montero‐Barrientos *et al*., 2011).

Considerably fewer genes were downregulated upon contact with Foc4 in the Tgui, which to the most part again comprised unknown and orphan proteins (Fig. 8). Genes that were downregulated in the parent strains and Tgui_*nox1*OE_, but in the Tgui_*Δnox1*_ strain comprised proteases, short chain dehydrogenases/reductases, glycoside hydrolases not connected with cellulose, chitin or ß‐glucan degradation, AB‐hydrolases including lipases, PTH11 receptors, MFS and amino acid permeases, transporters for inorganic ions and proteases. Several of these gene families/groups were also present in the pool of upregulated transcripts indicating that regulation of specific genes is relevant to the interaction with Foc4 and not of a whole gene family. In addition, there were only small differences within the downregulated gene pools of Tgui and Tgui_*nox1*OE_.

An interesting case, which was only observed in the Tgui parent strain, however, was a massive downregulation of genes (62, Fig. 8) encoding ribosomal proteins, which was paralleled by a downregulation of *cpc2* (OPB36030), a regulator of the cross‐pathway control of amino acid biosynthesis (Hoffmann *et al*., 1999) and of several genes related to tRNA formation and amino acid biosynthesis (Supporting Information S5). This is suggestive of a strong signal of nitrogen shortage which interestingly seems not to occur in Tgui_*nox1*OE._

### *Toxicity is the main strategy used by* F. oxysporum *in combat with* T. guizhouense

The transcriptional response of Foc4 to Tgui is relatively weak, as the number of differentially regulated genes is considerably lower in Foc4 than in Tgui (in view of the number of 18 065 predicted proteins in the Foc4 genome; Guo *et al*., 2014) suggesting that this fungus may express many genes constitutively. Indeed, three NRPSs, three PKSs and five terpene synthase genes were constitutively expressed (Supporting Information S5). However, among the genes that were strongly upregulated upon contact with Tgui or Tgui_*nox1*OE_, transcripts of genes encoding enzymes involved in secondary metabolite biosynthesis or the efflux of and resistance to these metabolites, as well as cytochrome P450 monooxygenases, were the most abundant. These data suggest that the default toxicity of Foc4 was further increased upon the interaction with Tgui.

Upon contact with Tgui and Tgui_*nox1*OE_, Foc4 also increased the expression of a few genes encoding HET proteins and ankyrins but in much lower numbers than Tgui. Foc4 does not upregulate any hydrophobin‐encoding genes, indicating that this pattern is a specific feature of Tgui. An interesting feature of Foc4 in contact with Tgui is the expression of genes encoding the alternative oxidase AOX1, a marker for stress response and several genes encoding enzymes that combat the result of oxidation (an organic hydroperoxide detoxification protein; a catalase; and peroxiredoxin HYR1; EMT 64830, EMT68375, EMT67110 respectively). Finally, several genes involved in membrane biosynthesis and maintenance were upregulated. These data are in good agreement with our hypothesis that Tgui overwhelms Foc4 with H_2_O_2_.

Foc4 shows almost no transcriptional response to the presence of the Tgui_*Δnox1*_ strain. Only very few genes were significantly upregulated or downregulated, most of which do not hint at a particular physiological function. We also note that the three genes involved in peroxide detoxification were not upregulated under these conditions, indicating the importance of H_2_O_2_ in communication between these two fungi.

## Discussion

In this study, we analysed the combative interaction between the two hypocrealean fungi, *T. guizhouense* and *F. oxysporum*. When confronted in a closed environment with limited resources, *T. guizhouense* overgrows *F. oxysporum* and weakens its development by causing sporadic cell death and arrested growth. This interaction can be classified as necrotrophic mycoparasitism (Jeffries and Young, 1994; Kim and Vujanovic, 2016; Zhang *et al*., 2016), as *T. guizhouense* forms abundant conidiation above *F. oxysporum* without touching the substrate beneath and, therefore, probably takes nutrients from the latter fungus. However, as demonstrating the nutrient transfer from one fungus to another was not targeted in this study, we do not claim the relationship to be biotrophic. The interaction involves damage of *F. oxysporum* thallus due to the activity of extracellular diffusates (guttation) of *T. guizhouense* that are enriched in ROS and hydrolytic enzymes. The presence of these enzymes fits with the findings of Zhang and colleagues (2016), who showed that the metalloprotease NMP1 has a role in the combative success of *T. guizhouense* against *F. oxysporum*. In this study, we demonstrated that this process was coupled with the production of H_2_O_2_. As the primary functions of both H_2_O_2_ and these hydrolytic enzymes are not linked to fungivory (*see below*), we suggest that their action (and possibly that of other molecules) represents an example of metabolic exaptation, that is acquisition of a new function for a trait that initially evolved for another purpose or had no adaptive role (True and Carroll, 2002; Barve and Wagner, 2013). NMP1 has orthologues in all Ascomycota lineages, which suggests its long evolutionary history and basic role in helping these fungi receive nutrition from protein‐rich food, which is not limited to fungi feeding on fungal biomass (Zhang *et al*., 2016). Similarly, the NADPH oxidases NOX1 and NOX2 are highly conserved in all eukaryotes, including fungi (Scott, 2015), where they play diverse roles: they are involved in plant pathogenesis, endophytic growth and fungal development (Cano‐Dominguez *et al*., 2008; Hernandez‐Onate *et al*., 2012; Mu *et al*., 2014; Marschall *et al*., 2016; Zhou *et al*., 2017). NOX1 is also essential for trap formation in the nematode‐killing fungus *Arthrobotrys oligospora* (Helotiales, Ascomycota) (Li *et al*., 2017), and mycoparasitism of dothideomycetous fungus *Coniothyrium* (Pleosporales) (Wei *et al*., 2016). In addition, *nox1* overexpression in *Trichoderma simmonsii* (previously named ‘*Trichoderma harzianum’* CECT2413) increases the parasitism on *Pythium ultimum* (Peronosporales, Oomycota) and the formation of cellulases, chitinases and proteases (Montero‐Barrientos *et al*., 2011). Remarkably, in most these studies, the effect was attributed only to the formation of superoxide radicals. However, due to the short life‐time of superoxide in aqueous systems and its non‐enzymatic conversion to H_2_O_2_, many of the inhibitory effects ascribed to it were likely due to hydrogen peroxide. Here, we show that the role of NOX1 in the antagonism of *F. oxysporum* f. sp. *cubense 4* is the production of hydrogen peroxide. Hydrogen peroxide was previously believed to be formed during interfungal relationships by flavoproteins such as glucose oxidase (Brunner *et al*., 2005) or amino acid oxidases (Yang *et al*., 2011). In this study, we show that because the *Δnox1* mutant produces only very little H_2_O_2_, likely by the activity of flavoprotein oxidases, NOX1 indeed accounts for most of the hydrogen peroxide formed.

Comparison of the confrontations between *F. oxysporum* and the parent strain *T. guizhouense* or its *Δnox1* mutant revealed that the induction of oxidative stress in *T. guizhouense* initiates a cascade of metabolic processes. Our results with *T. guizhouense,* therefore, are in agreement with the overall versatility of NOX1 function. Contrary to other fungi, the knocking out of *nox1* from *T. guizhouense* affected neither hyphal growth nor conidiation; however, abnormally thin or damaged hyphae were still observed. This result indicates that NOX signalling has ancient evolutionary roots but has been modified during fungal evolution. Our finding introduces another aspect of the picture, that is the role of *nox1* in accumulating a toxic concentration of H_2_O_2_ to affect *F. oxysporum* via aerial hyphal growth and the formation of guttation droplets.

In this study, we noticed the production of aerial bag‐like structures (guttation capsules) during the interaction of the two fungi. Guttation was originally described as the loss of water and dissolved materials from uninjured plant organs (Stocking, 1956) but is now also known to occur in many fungi (Gareis and Gareis, 2007; Hutwimmer *et al*., 2010; Munoz *et al*., 2011; Gareis and Gottschalk, 2014; Castagnoli *et al*., 2018). Consistent with the present results, these droplets are produced on aerial parts of the mycelia (Sprecher, 1959) and exit the cell by exocytosis (Read, 2011), and contain enzymes (McPhee and Colotelo, 1977) and secondary metabolites (Gareis and Gareis, 2007; Munoz *et al*., 2011; Castagnoli *et al*., 2018). Interestingly, in our settings, the surface of these capsules had a visible film that probably provided the reservoir for the solvent necessary for the biochemical reactions involving hydrolytic enzymes and H_2_O_2_ on the aerial hyphae. *F. oxysporum* hyphae are hydrophilic (Combes *et al*., 2012), and the formation of guttation droplets by *T. guizhouense* on them would, therefore, require a surface‐active protein. In support of this mechanism, we found the upregulation of one hydrophobin‐encoding gene (OPB44528), and its expression was NOX1‐independent. In the Tgui_*nox1*OE_ strain, one additional hydrophobin gene (OPB37525) was upregulated. These secreted proteins self‐assemble in the interface (Szilvay *et al*., 2007; Przylucka *et al*., 2017), and thus, may be linked to the formation of the film on the surface of the guttation capsule. However, at this stage there is no experimental evidence supporting this hypothesis. HFB upregulation was also reported in *nox1*‐overexpressing *T. simmonsii* during antagonism of *Pythium ultimum* (Montero‐Barrientos *et al*., 2011).

The transcriptomic experiments in this work suggest that in *T. guizhouense,* the *nox1* gene is induced at the early stage of the interaction in response to the constitutively produced toxic secondary metabolites of *F. oxysporum*. The toxicity of *F. oxysporum* to *T. guizhouense* was also evident as an antibiosis zone on a confrontation plate. The induction of an oxidative stress response by *Fusarium* spp. toxins in plants has been well documented in the literature (Audenaert *et al*., 2014; Lanubile *et al*., 2015). The nature of the toxins can be only hypothesized: some of the secondary metabolite‐synthesizing gene clusters of *F. oxysporum* include those producing beauvericin, fusaric acid, fusarin C, fumonisin and fusarubin (Ma *et al*., 2013). However, we did not detect the expression of the corresponding genes in the Foc4 transcriptome. On the other hand, we observed constitutive expression of three NRPSs, two terpene synthases and three PKSs; two of these PKSs (EMT 72002 and EMT 66565) were also strongly upregulated during contact with *T. guizhouense*, indicating that they are candidates for producing the toxin.

Although, the lineages leading to extant *Trichoderma* and *Fusarium* genera diverged nearly 200 MYA (Sung *et al*., 2008; Yang *et al*., 2012), the core genomes of these fungi share significant functional similarity (Kubicek *et al*., unpublished). Both fungi can grow and complete their asexual life cycle as saprotrophs, both have herbivory and fungivory abilities; however, *F. oxysporum* is preferentially associated with plants, while *T. guizhouense* is thought to be mycophilic. Attempts to use *Trichoderma* spp. as bioeffectors in the biocontrol of plant diseases caused by *F. oxysporum* frequently fail or show only partial efficiency (Sivan and Chet, 1989; Ordentlich *et al*., 1992). In our study, we noted that the reproducible attack of *T. guizhouense* on *F. oxysporum* is triggered by the conditions of the dual confrontation assay when the saprotrophic growth of both fungi is no longer possible. When both strains simultaneously germinate and coalesce, a succession pattern can be observed: *T. guizhouense* rapidly colonizes the entire foraging ground, while Foc4 later develops, most likely utilizing the aged mycelia of *T. guizhouense* that it kills by toxins. Similar replacement patterns were observed for Basidiomycota wood decay fungi when the mycotrophy was considered to be a secondary strategy to the primary strategy of saprotrophy (Hiscox *et al*., 2018). The interactions between non‐trophic hyphae on the surface of the glass slide showed that both fungi grew alongside the partner (Fig. 1). In summary, our data show that the current model of interfungal interactions (mycoparasitism) needs to be expanded with respect to the partners, mechanisms, morphological structures formed and the chemicals involved.

## Experimental procedures

### *Fungal strains and cultivation conditions*

The fungal strains used in this study are given in Supporting Information S9.

For molecular biological work, fungi were propagated on potato dextrose agar (PDA) (Difco, Germany) or glucose synthetic medium (GSM) (Zhang *et al*., 2016). In all experiments, unless specified differently, fungi were cultivated at 25°C in darkness.

### *Detection of ROS production and cell damage assays*

For the assessment of H_2_O_2_ toxicity to fungi, PDA plates were supplemented with 5, 10 and 20 mM H_2_O_2_ and kept in darkness. The diameter of fungal colonies was estimated after 2 or 6 days. Hyphal integrity was tested by the careful spread of trypan blue solution (5 ml, 0.1% in distilled water), which stains dead hyphae above the fungal colony dark blue. After incubation for 10 min at room temperature, plates were extensively rinsed with distilled water and photographed. The production of O_2_^•−^ and H_2_O_2_ was assayed as previously reported (Silar, 2005). Alternatively, the integrity of the cytoplasmic membrane was determined using propidium iodine (PI) (Sigma‐Aldrich, MO, USA). For this assay, fungal hyphae from the interaction zone or germinating spores [10^6^ spores/ml after 18 h of incubation in potato dextrose broth (PDB)] were incubated for 5 min in 10 mg/l PI and observed under a Leica DMi8 fluorescence microscope (Germany) using 495 nm excitation and 500 to 550 nm emission. Red fluorescence indicates areas of damaged plasma membrane.

The detection of O_2_^•−^ and H_2_O_2_ production on Petri plates was performed as described by Malagnac and colleagues (2004) using nitro blue tetrazolium (for O_2_^•−^) or diaminobenzidine (2.5 mM) and 5 purpurogallin units of horseradish peroxidase (Sigma‐Aldrich) per ml in potassium phosphate buffer, pH 6.5 (for H_2_O_2_). The formation of deep blue and reddish colours indicated the production of O_2_^•−^ and H_2_O_2_ respectively.

For the fluorescence assays of H_2_O_2_ produced by fungal hyphae, an agar block (1 cm^2^) was excised from the confrontation zone (*see above*) and incubated in the presence of 2.5 μg/ml 2′,7′‐dichlorodihydrofluorescein diacetate (H_2_DCFDA) (Molecular Probes, Thermo Fisher, MA, USA) for 10 min. Then, the interacting hyphae were transferred to a microscopy slide and observed by a fluorescence light microscope (TCS SP8, Leica, Germany) using 460 to 490 nm excitation and 500 to 550 nm emission for detecting H_2_O_2_ and 561 nm excitation and 571 nm emission for detecting red fluorescent protein (RFP).

### *Dual confrontation assays and transcriptomic analysis*

Fungi were cultivated on PDA at 25°C for 5 days. First, an agar plug (5 mm) with Foc4 was placed 1 cm from the edge of a 9 cm Petri plate filled with 15 ml of GSM covered with sterile cellophane, which was then sealed with Parafilm and incubated for 48 h in darkness at 25°C. Then, a similar plug of *T. guizhouense* NJAU 4742 (Tgui) was placed on the opposite edge of the plate, and the plate was sealed with Parafilm and incubated under the same conditions. Mycelia of each fungus were sampled before contact when the gap between the colonies was equal to 1 cm. The mixed samples of both fungi were collected 30 h after the contact. All mycelia were shock‐frozen in liquid nitrogen and ground to a fine powder under liquid nitrogen. Total genomic DNA was extracted using the Qiagen Plant Tissue Kit from an aliquot of each sample and tested for the absence of bacterial DNA using PCR with universal primers 27F (5′‐AGRGTTTGATCMTGGCTCAG) and 1492R (5′‐GGTTACCTTGTTACGACTT) and a standard amplification protocol (Margulies *et al*., 2006). The extraction of the total RNA, cDNA synthesis, library preparation, deep sequencing and data analysis are described in Supporting Information S8. The reads were deposited in the Sequence Read Archive of the NCBI under accession number (GSE117839).

Transcripts were identified by comparison with the manual genome annotations for Tgui (complete genome) and Foc4 (partial genome) which are provided in Supporting Information S10. Specific gene families or groups were assembled according to the criteria of FunCat (Ruepp *et al*., 2004). Fungal specific gene families and groups were identified using established databases (CAZy database: http://www.cazy.org/ ; secondary metabolite synthesis: antiSMASH (Medema *et al*., 2011) and SMURF (http://www.jcvi.org/smurf/index.php); MEROPS protease database: https://www.ebi.ac.uk/merops/).

Functional enrichment analysis of differentially expressed genes (for each condition WT, Δ*nox1* and *nox1* OE) based on Gene Ontology (GO) terms was performed using the R package GO_MWU (https://github.com/z0on/GO_MWU) using the whole protein set as background for Fisher exact test and *p* value as a measure of significance.

### *Microscopy analyses*

The interaction between fungi in dual confrontation assays (*see above*) was investigated using either a light stereomicroscope [LIOO, China; imaged with a ‘Mikroskop‐Kamera 8 MP’ camera (Oowl, Germany)] or a Leica DMi8 fluorescence microscope (Germany). For scanning electron cryomicroscopy (cryo‐SEM), agar blocks (0.3 × 0.6 cm) were excised with a sterile scalpel, plunge‐frozen in a liquid nitrogen slush and observed in the cryo‐SEM HITACHI SU8010 (Tokyo, Japan).

### *Chemical analysis of guttation droplets*

Guttation droplets were collected from plates with dual confrontation assays between Foc4 and Tgui (150 plates) at room temperature and ambient illumination, centrifuged at 12 000 *g* for 5 min at +4°C and kept at −20°C before their analysis. H_2_O_2_ was quantified with Amplex® Red Hydrogen Peroxide/Peroxidase Assay Kit (Invitrogen, USA) according to the manufacture protocol. Enzymatic activity assays and a chemical analysis of the mixture are described in Supporting Information S3.

### *Construction of the mutants*

Details are provided in Supporting Information S6. The open reading frames of genes *nox1* (OPB44254), *nox2* (OPB36463) and *nor1* (OPB40972) were retrieved from the genome of Tgui (NCBI GenBank: LVVK00000000.1). A plasmid with a hygromycin resistance cassette (Derntl *et al*., 2015), a complementary plasmid encoding geneticin resistance (Seiboth *et al*., 2012) and an overexpression plasmid harbouring the *T. reesei cdna1* promoter (Uzbas *et al*., 2012) were constructed and directly used for polyethylene glycol (PEG)‐mediated protoplast transformation as described in in Supporting Information S6. Transformants were screened by PCR with a Phire Plant Direct PCR kit (Thermo Scientific, USA) and subjected to three rounds of single‐spore purification.

## Supporting information


**Supporting Information S1.** Dual confrontation assays between different *Trichoderma* spp. and *Fusarium oxysporum* f. sp. *cubense* 4 (Foc4)

**Supporting Information S2.** Combative interactions between *T. guizhouense* NJAU 4742 and *Fusarium oxysporum* f. sp. *cubense* 4 (Foc 4)

**Supporting Information S3.** Enzymatic and chemical analysis of guttation droplets

**Supporting Information S4.** Effect of H_2_O_2_ on Foc4 and other fungi

**Supporting Information S5.** All significantly regulated genes in FOC4 when confronted to NJAU 4742 of WT, *Δnox1* and *nox1*OE respectively.

**Supporting Information S6.** Transformation of *T. guizhouense* NJAU 4742 and verification of the mutants

**Supporting Information S7.** Phenotype of *T. guizhouense* NJAU 4742 *nox*‐mutants

**Supporting Information S8.** Transcriptomic analysis

**Supporting Information S9.** Strains used in this study

**Supporting Information S10.** Annotation of the genome of *T. guizhouense* NJAU 4742 and manual annotation of differentially expressed genes of *Fusarium oxysporum* f. sp. *cubense* 4 (Foc4)
