# Supplementary material for: Guttation capsules containing hydrogen peroxide: an evolutionarily conserved NADPH oxidase gains a role in wars between related fungi
Source: Environ Microbiol. 2019 Apr 22;21(8):2644–58. doi: 10.1111/1462-2920.14575 (PMC6850483; doi:10.1111/1462-2920.14575)
Supplement: Supplementary file 1 — Supporting Information S1. Dual confrontation assays between different Trichoderma spp. and Fusarium oxysporum f. sp. cubense 4 (Foc4) [file EMI-21-2644-s001.pdf]

Supporting Information S1: Dual confrontation assays between different *Trichoderma* spp. and *Fusarium oxysporum* f. sp. *cubense* 4 (Foc4)

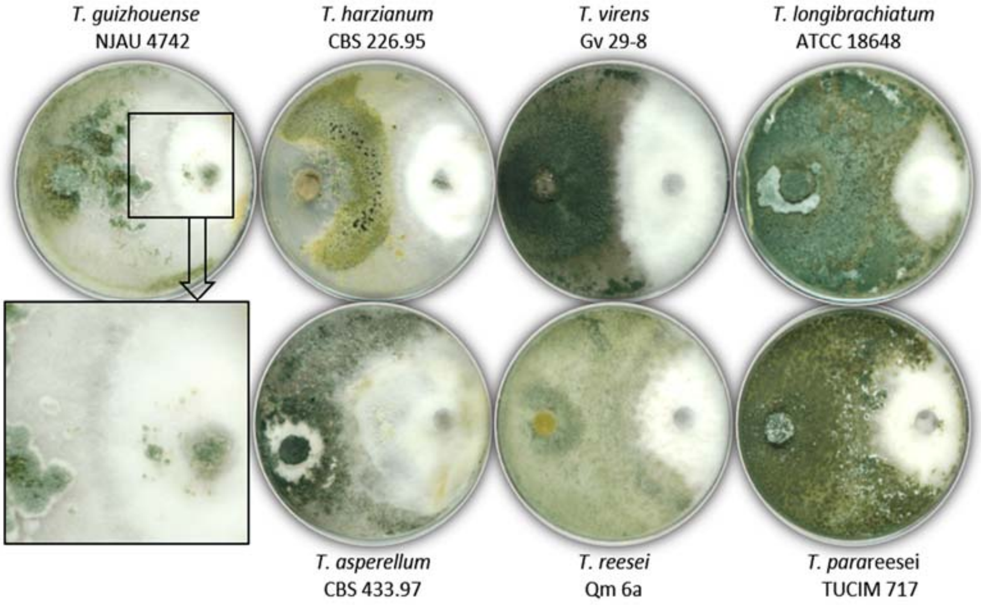

Results of the dual confrontation assays between different *Trichoderma* spp. and Foc4. Only *T. guizhouense* NJAU 4742 could overgrow Foc4, while other species remain in the deadlock phase. The images were obtained after seven days of incubation on PDA at 25 C in 12 hours light/darkness cycle. The diameter of the plates is 9 cm.

Trichoderma was inoculated on the left side, Foc4 on the right side.

Strains used in this study are listed in Supportive information S8.
