# Supplementary material for: Guttation capsules containing hydrogen peroxide: an evolutionarily conserved NADPH oxidase gains a role in wars between related fungi
Source: Environ Microbiol. 2019 Apr 22;21(8):2644–58. doi: 10.1111/1462-2920.14575 (PMC6850483; doi:10.1111/1462-2920.14575)
Supplement: Supplementary file 2 — Supporting Information S2. Combative interactions between T. guizhouense NJAU 4742 and Fusarium oxysporum f. sp. cubense 4 (Foc 4) [file EMI-21-2644-s002.pdf]

Supporting Information S2: Combative interactions between *T. guizhouense* NJAU 4742 and *Fusarium oxysporum* f. sp. *cubense* 4 (Foc 4)

Contents

Partial cell death of Foc4 overgrown by Tgui 2

Guttation capsules formed by Tgui in interactions with Foc4 3

Interaction of Tgui hyphae when confronted to itself. 10

Interaction of Foc4 hyphae when confronted to itself. 12

Macromorphology of the contact zone in the dual confrontation assay between Tgui and Foc4. .. 13

Microscopic examination of the early stage of the interaction between Tgui and Foc4 on the glass slide 14

Interaction between Tgui and $Tgui_{RFP}$ on the glass slide. 15

Interaction between Tgui and $Tgui_{RFP}$ on the glass slide 16

Inhibition of Foc4 by volatile organic compounds of Tgui. 17

Partial cell death of Foc4 overgrown by Tgui

Figure S2-1 Trypan blue staining of the dual interaction assay between *T. guizhouense* NJAU 4742 (Tgui) and *Fusarium oxysporum* f. sp. *cubense* 4 (Foc 4).

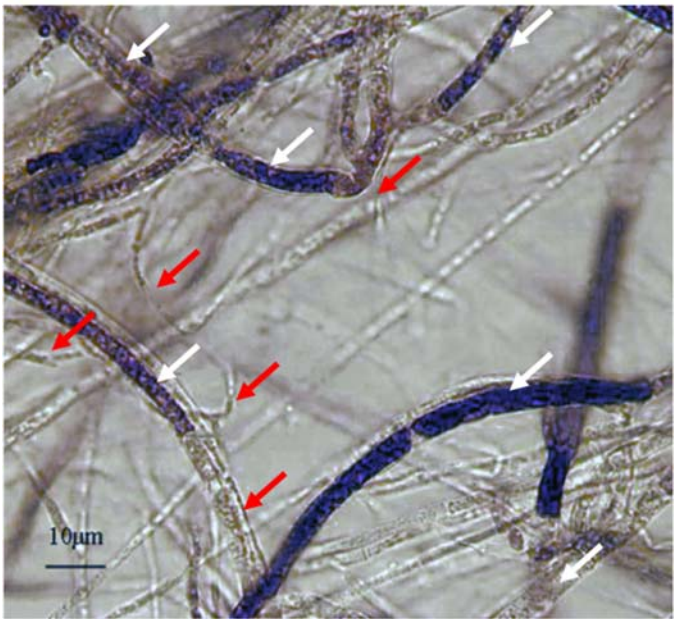

Plates were incubated on GSM in darkness at 25°C. The Trypan blue stain that differentiates between dead (dark blue) and living (hyaline) hyphae was applied. Briefly, 5 ml of a 0.1% Trypan blue solution in distilled water were spread on the confrontation plates with a glass rod to ensure homogenous access of the dye to all mycelia. After incubation for 10 min at room temperature, plates were then extensively rinsed with distilled water and photographed. The dark blue stain accumulated in damaged hyphae. Red arrows indicate Tgui, white arrows show mycelium of Foc4. The sample was collected from the interaction zone.

Guttation capsules formed by Tgui in interactions with Foc4

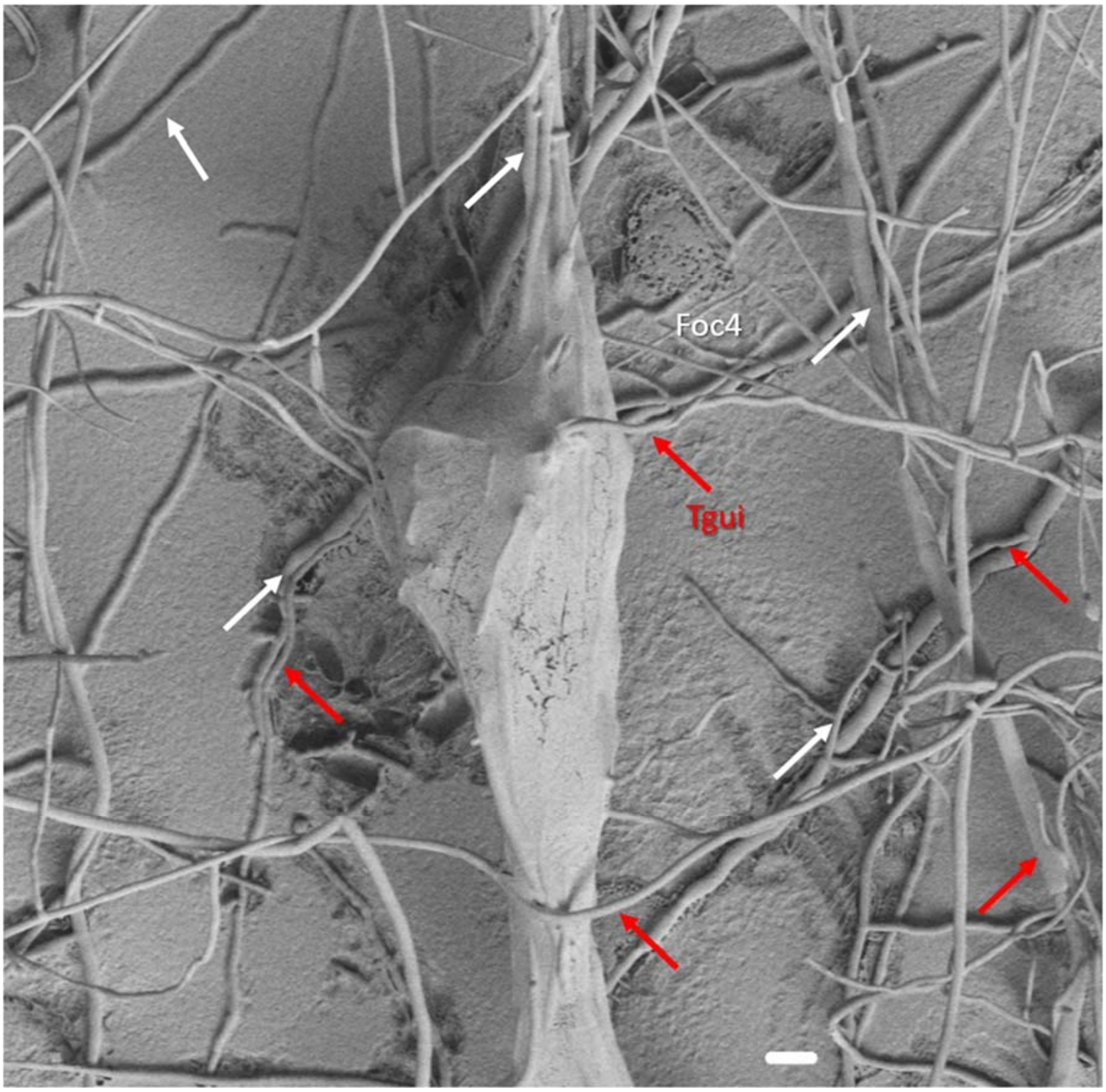

Figure S2-2: The original non-colored Cryo-SEM image of the guttation capsule shown on Figure 3. Red arrows indicate *T. guizhouense* NJAU 4742, white arrows show hyphae of Foc4. The scale – 20 µm

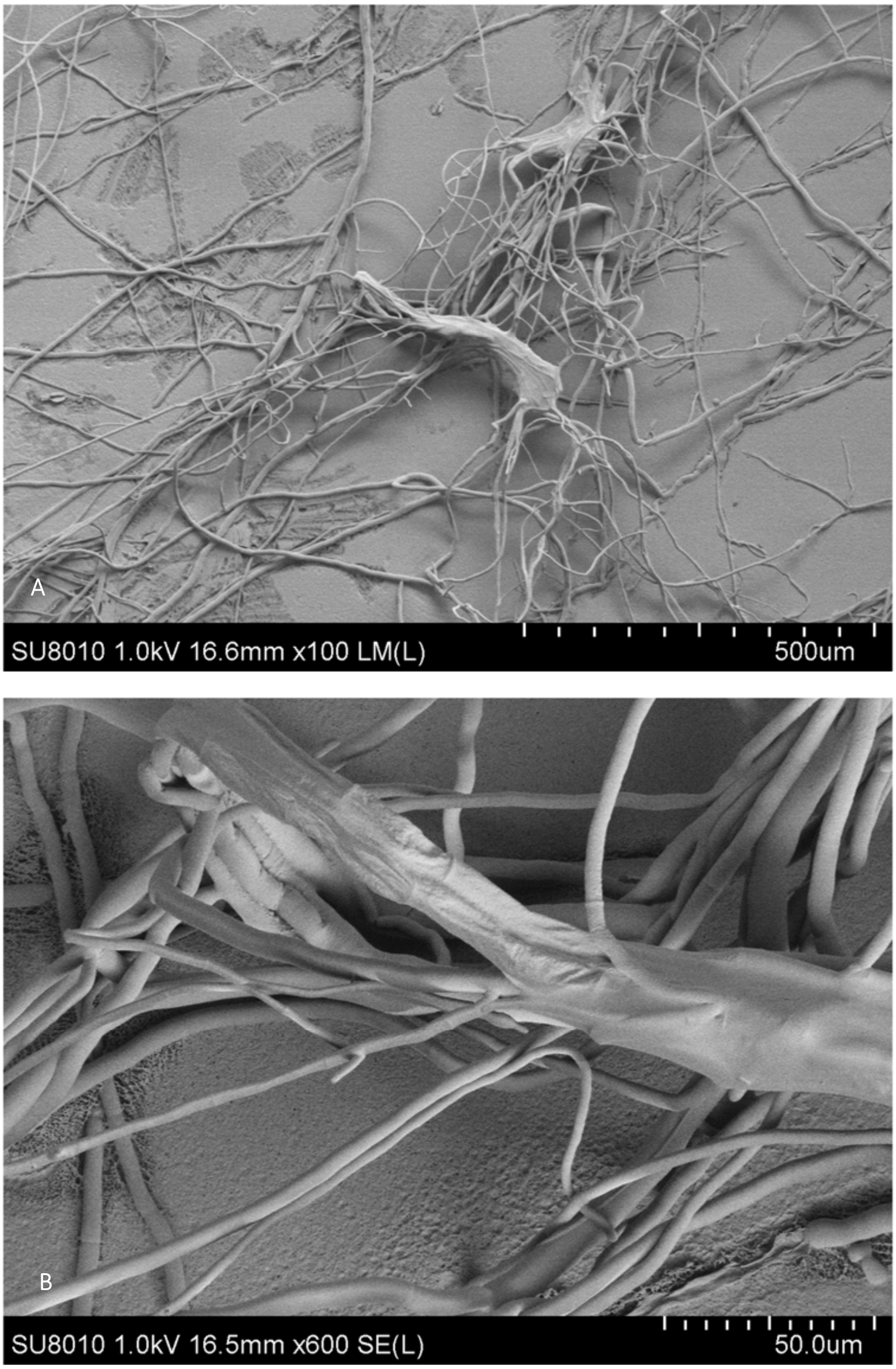

Figure S2-3: Cryo-SEM image of the bag-like guttation capsules on aerial hyphae at the early stages of the contact between Tgui and Foc4. A and B show different biological repeats.

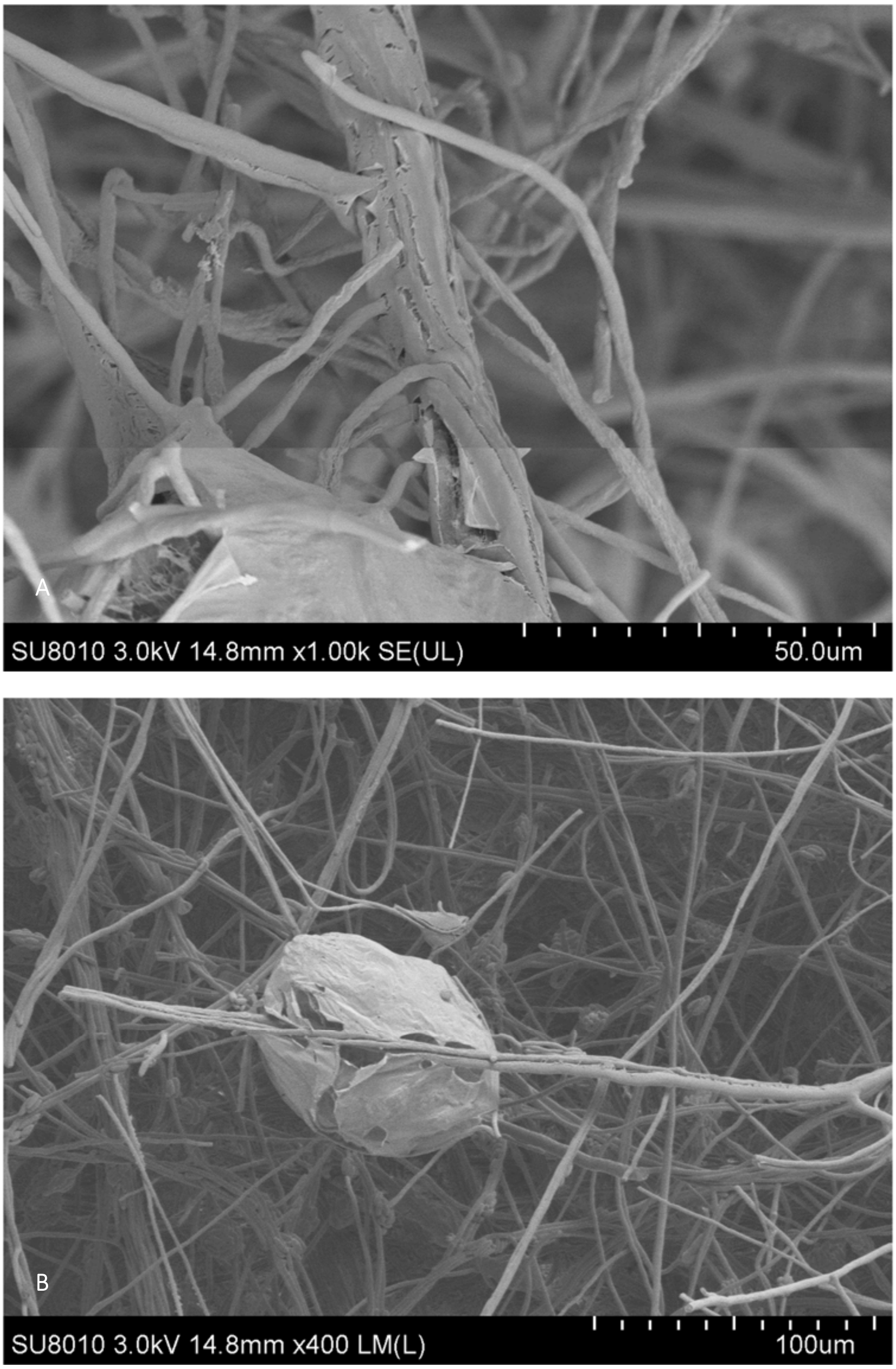

Figure S2-4: Cryo-SEM image of the bag-like guttation capsules on aerial hyphae at late stages of the interaction between Tgui and Foc4. A and B show different biological repeats. A and B represent different locations on the same plate. On B the conidiation of Foc4 is seen beneath the aerial mycelium.

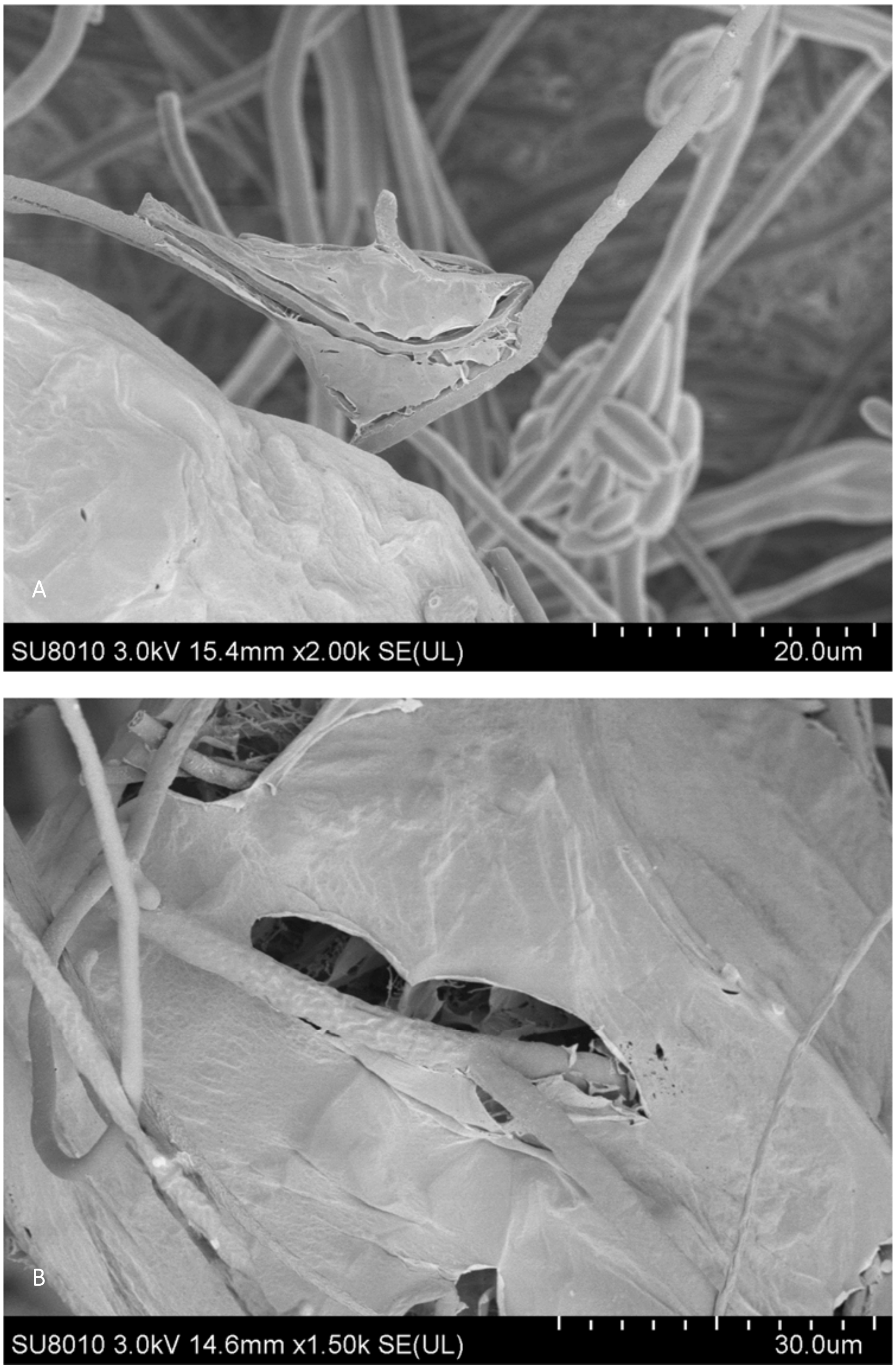

Figure S2-5: Cryo-SEM image of the bag-like guttation capsules on aerial hyphae at late stages of the interaction between Tgui and Foc4. A and B show the details of the guttation capsule on Fig. S2-4.

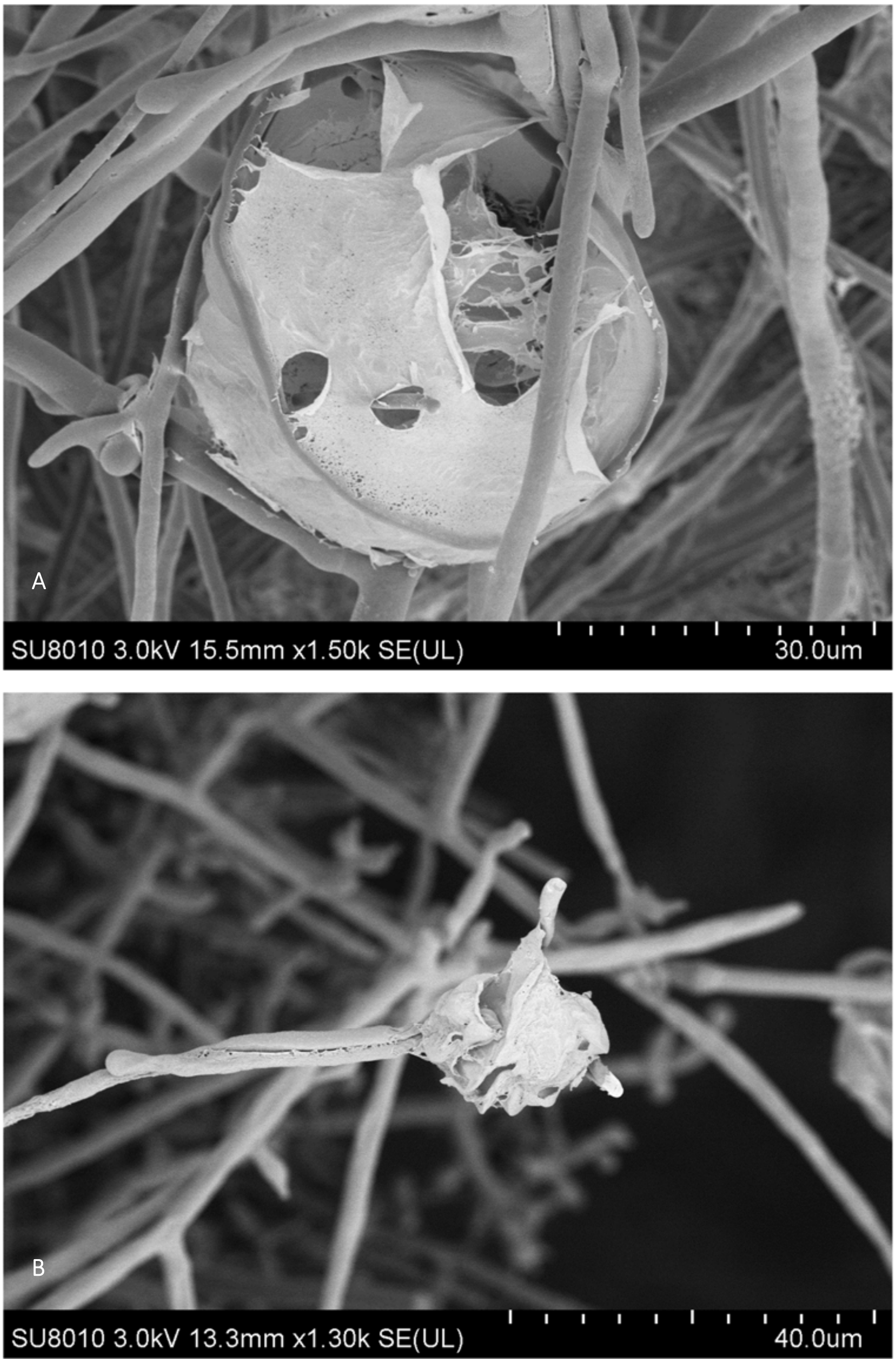

Figure S2-6: Cryo-SEM image of the bag-like guttation capsules on aerial hyphae at late stages of the interaction between Tgui and Foc4. A shows partially destroyed guttation capsule. On B Tgui conidiophores are visible beneath the front view.

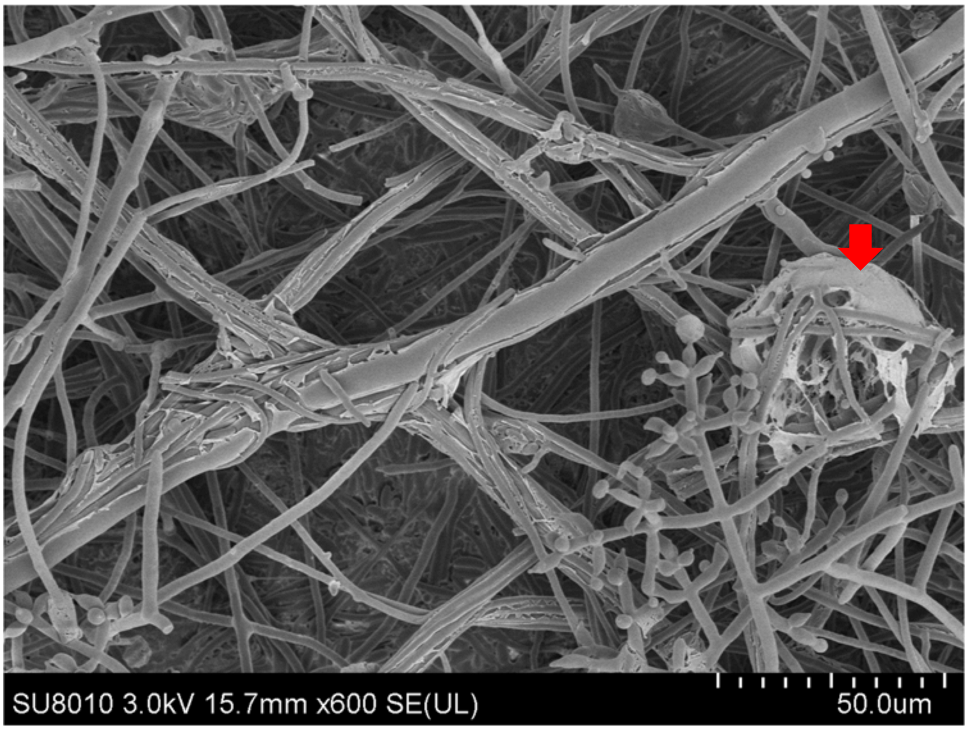

Figure S2-7: Cryo-SEM image of the of the climax stage of the interaction between Tgui and Foc4. Red arrow shows the remains of the old guttation capsule.

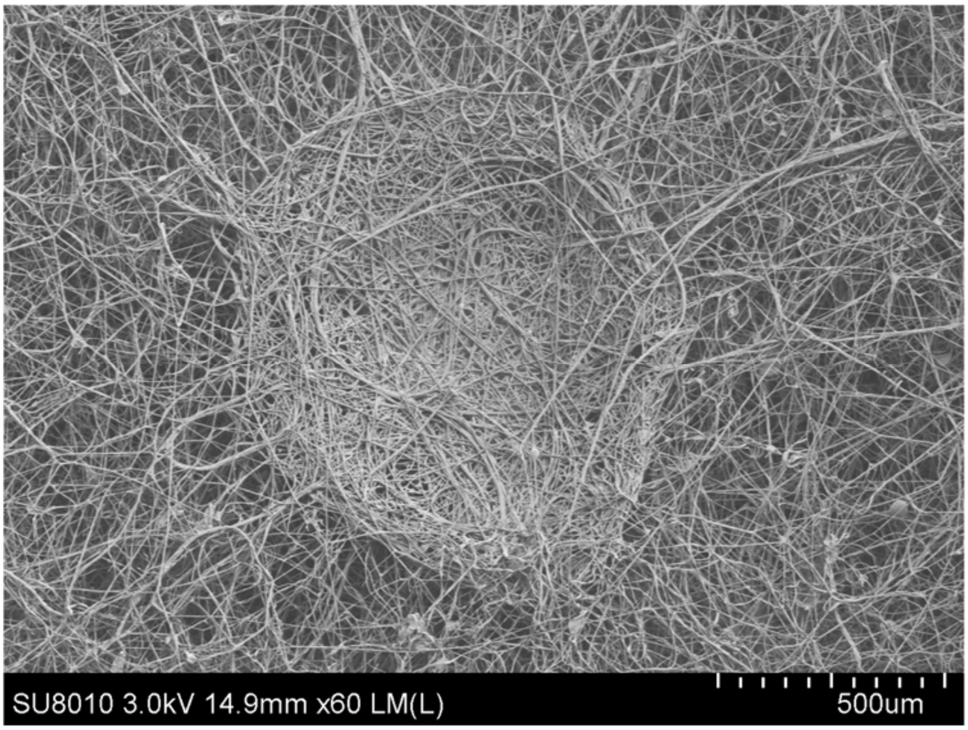

Figure S2-8: Cryo-SEM image of the of the aged interaction zone between Tgui and Foc4. The footprint corresponds to the area where a macroscopic guttation droplet was located (before it dried out). Numerous microscopic guttation capsules are visible on the aerial hyphae.

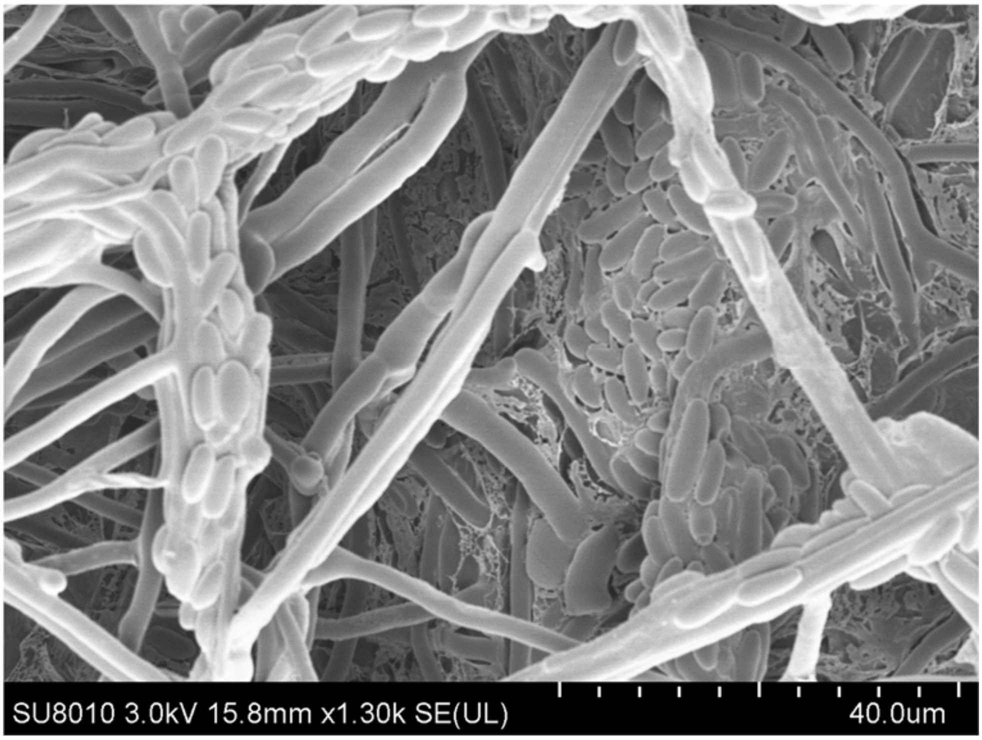

Figure S2-9: Cryo-SEM image of the aged plate on which Tgui and Foc4 were confronted. Despite Foc4 was overgrown by Tgui, the surface of the plate is covered by its microconidia indicating that Fc4 is not killed by Tgui.

Interaction of Tgui hyphae when confronted to itself.

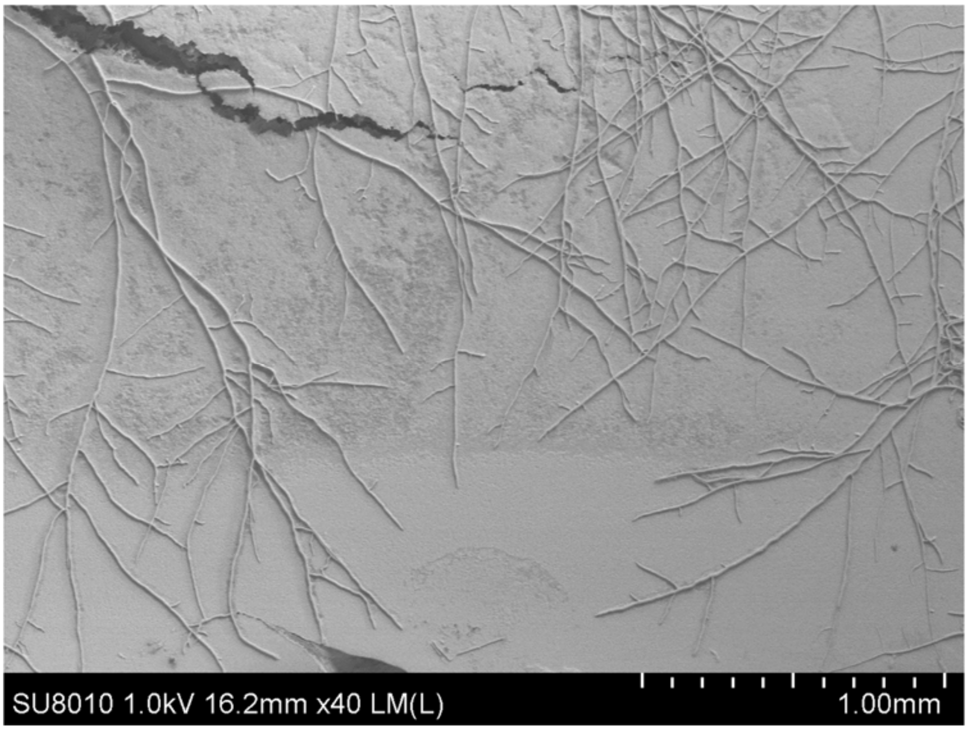

Figure S2-10: Cryo-SEM image of the contact zone between Tgui was confronted to itself.

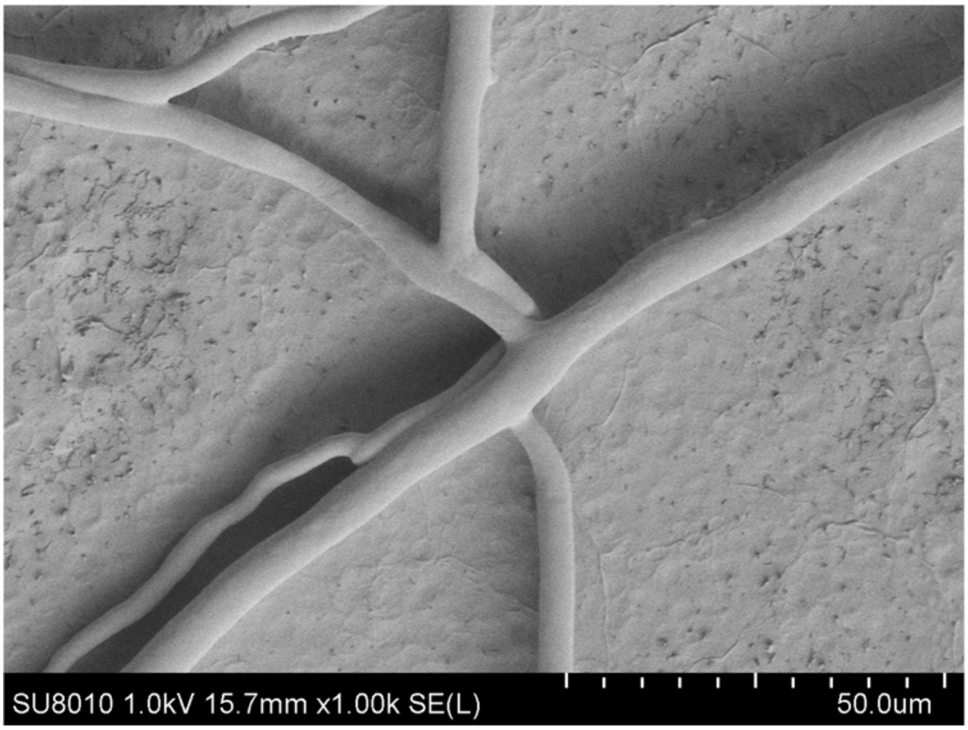

Figure S2-11: Cryo-SEM image of the contact zone between Tgui was confronted to itself.

Interaction of Foc4 hyphae when confronted to itself.

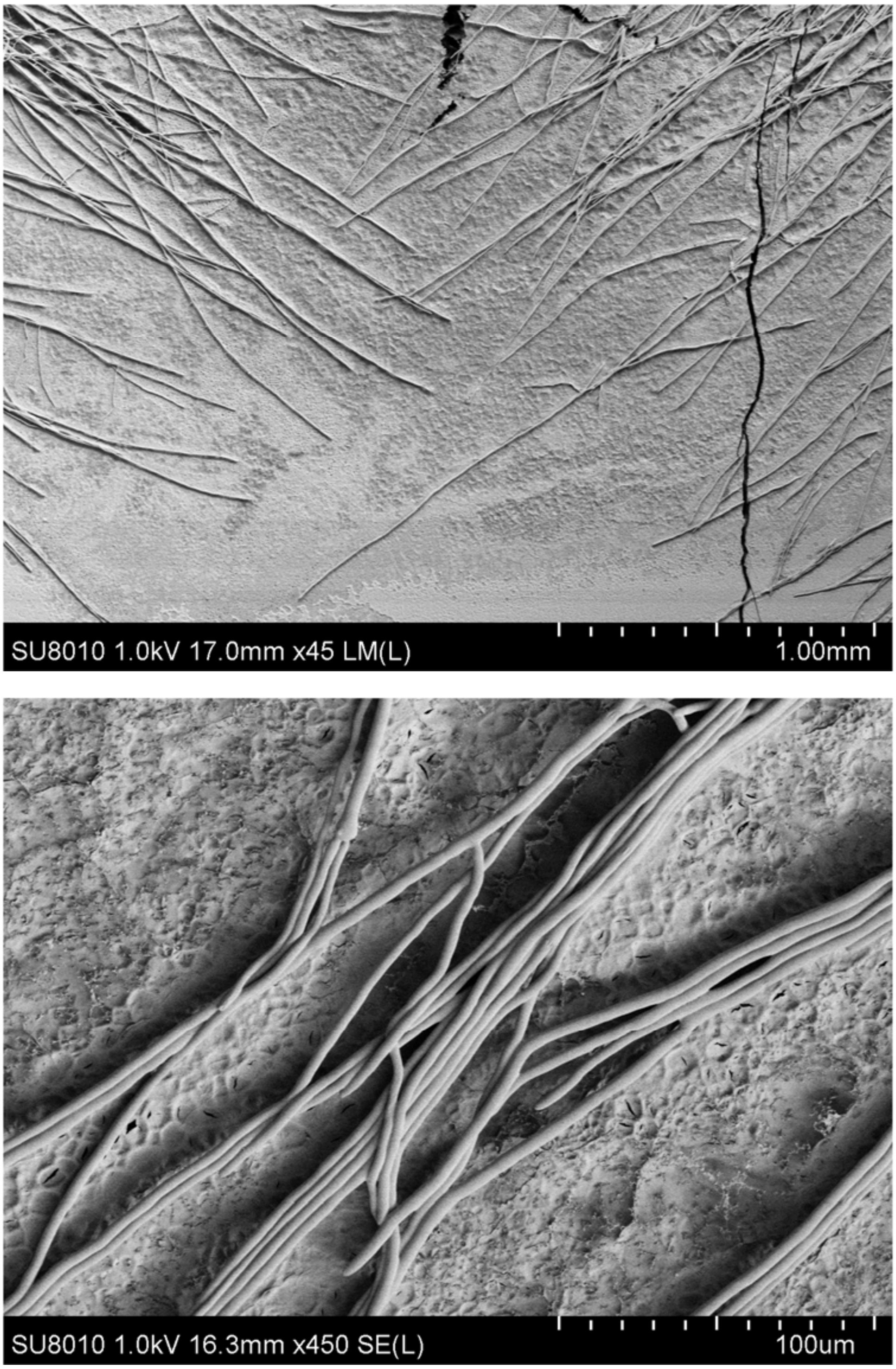

Figure S2-12: Two Cryo-SEM images of the contact zone when Foc4 was confronted to itself.

Macromorphology of the contact zone in the dual confrontation assay between Tgui and Foc4.

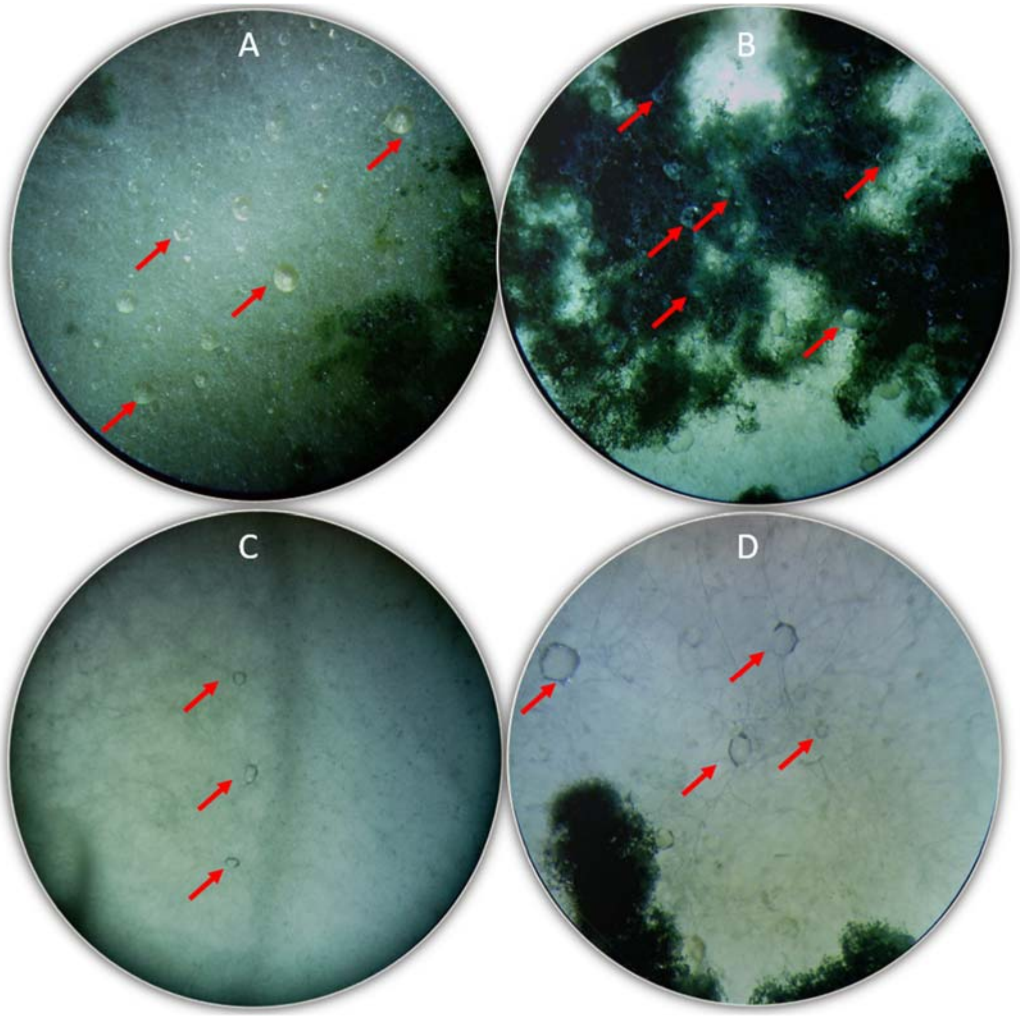

Figure S2-13. Guttation drops on aerial hyphae at the contact zone between Tgui and Foc4. Red arrows indicate guttation drops. The image is obtained in the light stereo microscope, magnification x 50 – 70. Green color originates from Tgui conidiation. A -D show biological repeats on diferent plates.

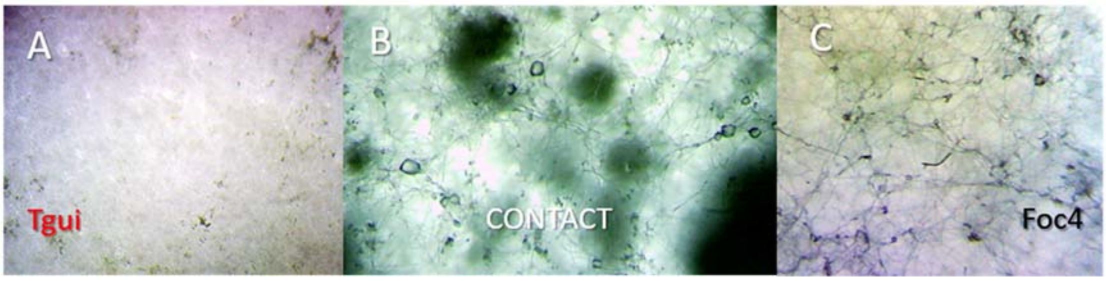

Figure S2-14. Comparison of the contact zone (B) with the area occupied solely by Tgui (A) and Foc4 (C). Guttation droplets are mainly observed in the contact area (B) and later on spread following the overgrowth of Foc4 by Tgui.

Microscopic examination of the early stage of the interaction between Tgui and Foc4 on the glass slide

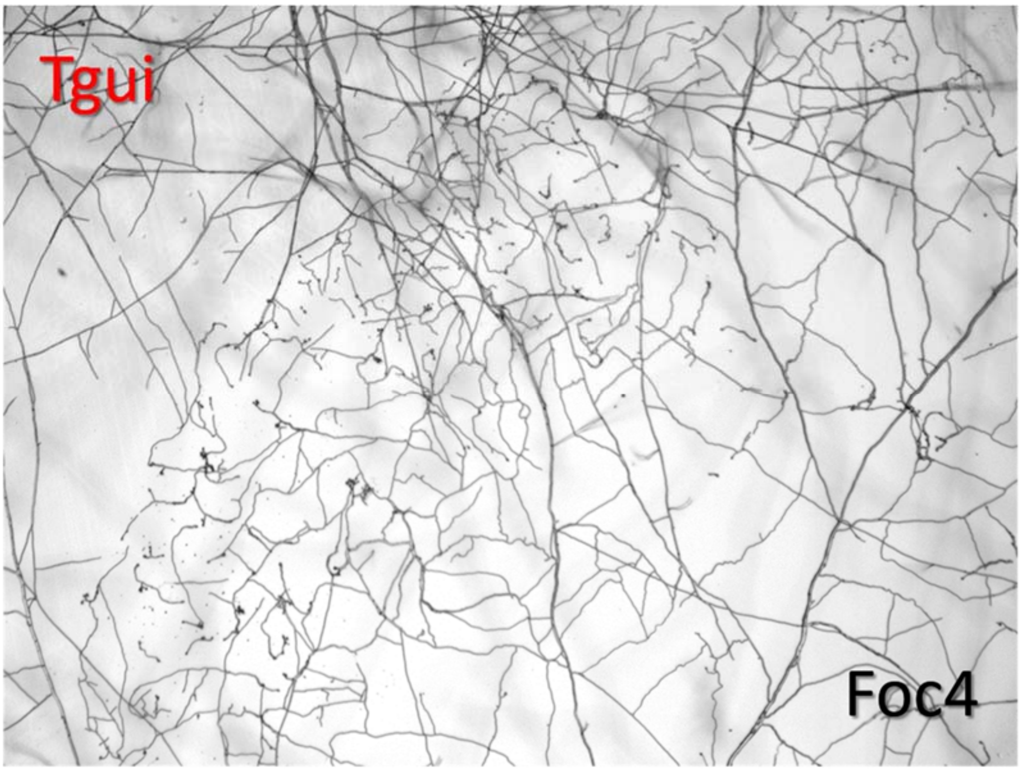

Figure S2-15. Overview of the contact zone between non-trophic hyphae of Tgui and Foc4 on the surface of the cover glass. Image was taken in light microscope, x 200 magnification. The glass slide was placed between the interacting fungi.

Interaction between Tgui and Tgui$_{RFP}$ on the glass slide.

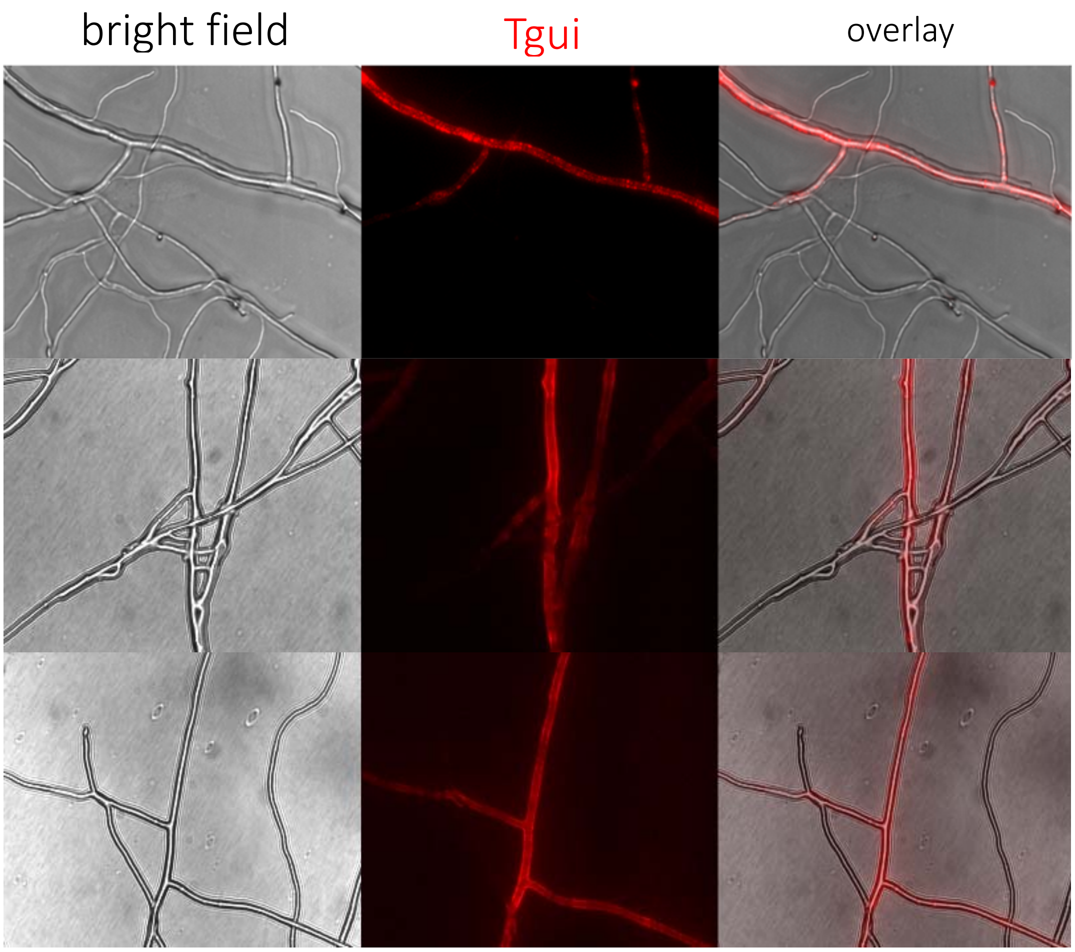

Figure S2-16. Early stage of the interaction between Tgui and Foc4 on the surface of the glass slide. Tgui is labelled with RFP (Tgu$_{RFP}$). The hyphae of each fungus frequently grew alongside the hyphae of the other. No cases of cellular necrosis or hyphal penetration were observed, but the contact zone remained an area of low mycelial density throughout the experiment. Coiling of Tgui around the hyphae of Foc4 was sporadically observed. When each fungus was confronted with itself, the interaction between hyphae was similar; however, no antibiosis area at the contact zone was observed, x 400.

Interaction between Tgui and $Tgui_{RFP}$ on the glass slide

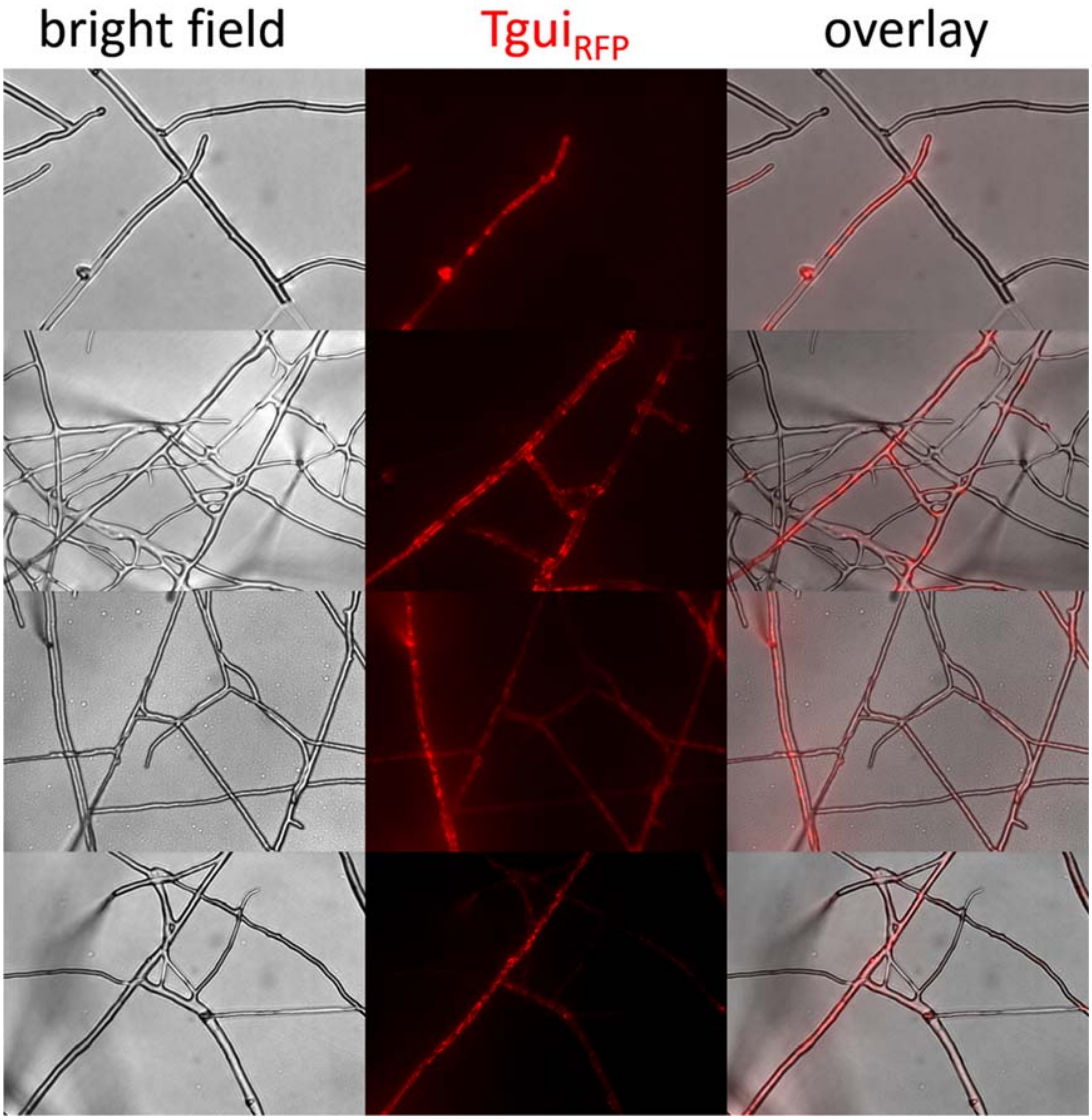

Figure S2-17. Similar interaction as of Figure S2-18 but $Tgui_{RFP}$ was confronted to the wild type. The morphology was equal to the early interaction stage with Foc4 indicating that these fungi do not develop combative interactions in all cases but only when they are confronted in a closed environment with limited resources

Inhibition of Foc4 by volatile organic compounds of Tgui.

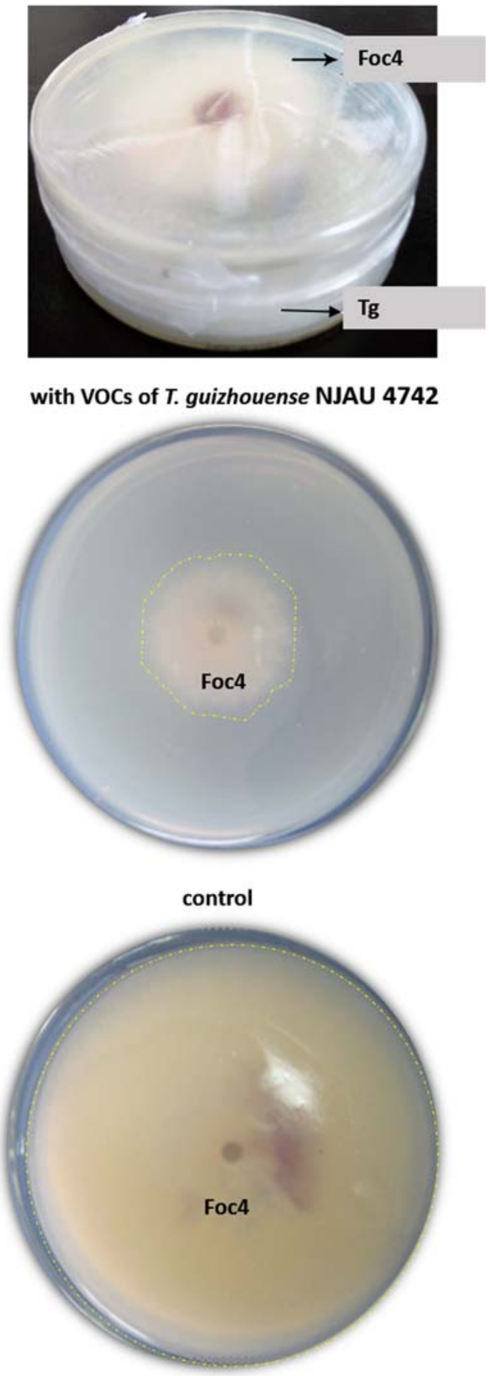

Figure S2-18. Experiment design is imaged on the upper panel, the growth of Foc4 in presence of VOCs of Tgui is shown in the middle panel compared to the confrontation to itself as imaged below. Yellow dashed line indicates the extension of the Foc4 colony.
