# Supplementary material for: Guttation capsules containing hydrogen peroxide: an evolutionarily conserved NADPH oxidase gains a role in wars between related fungi
Source: Environ Microbiol. 2019 Apr 22;21(8):2644–58. doi: 10.1111/1462-2920.14575 (PMC6850483; doi:10.1111/1462-2920.14575)
Supplement: Supplementary file 3 — Supporting Information S3. Enzymatic and chemical analysis of guttation droplets [file EMI-21-2644-s003.pdf]

Supporting Information S3: Enzymatic and chemical analysis of guttation droplets

Contents

Supporting Information S3: Enzymatic and chemical analysis of guttation droplets: Enzymatic and chemical analysis of guttation droplets 1
Materials and Methods 1
Enzymatic assays 1
Crude enzymes and n-butanol extraction 2
Inhibition assay of H_2O_2, crude enzymes and n-butanol extract 2
Analysis of guttation droplets by GC-MS 3
Results 3
Enzymatic activity of guttation droplets 3
GC-MS analysis of n-butanol extract of guttation droplets 4
The effect of n-butanol extract and enzymes on Foc4 6
References 7

Materials and Methods

Enzymatic assays

The activities of chitobiosidase, β-N-acetylglucosaminidase and endochitinase were assayed using the fluorometric chitinase assay kit (Sigma-Aldrich) with following modifications: A 10 µl aliquot of the guttation drop content was incubated with 90 µl substrate (10 µg/ml in phosphate buffer, pH 6.0) in 96-well plates in a 37 °C water bath for 20 min. The enzymatic reactions were stopped with 100 µl Na_2CO_3 (100 g/l), and the fluorescence was measured using a 96-well microplate reader (Molecular Devices, USA) with excitation and emission filters of 360 and 450 nm, respectively. The chitinase activity was expressed in nmol of methylumbelliferone (4-MU) released from the

substrate per min per ml of the test liquid.

Extracellular trypsin-like and chymotrypsin-like protease activities were measured using the chromogenic substrates N-benzoyl-Phe-Val-Arg-p-nitroanilide and N-succinyl-Ala-Ala-Pro-Phe-p-nitroanilide (Sigma Aldrich, respectively, as described previously (Szekeres *et al.*, 2004). One unit (U) of protease activity was defined as the amount of enzyme required to release one nmol p-nitroaniline per min per ml of liquid at 35 °C. Additionally, proteolytic activity was visualized on the casein agar plate as a halo appeared 12 hours after the application of 100 µl of the guttation drop content.

Crude enzymes and n-butanol extraction

Tgui culture filtrate was harvested from 100 ml GSM supplemented with 10 % (w/v) wet heat-killed Foc4 mycelia as the carbon source and cultivated for the three days. Crude enzymes were extracted after centrifugation from the culture broth by ammonium sulfate precipitation with 80% saturation (Zhou *et al.*, 2008). The precipitate was recovered by centrifugation and dissolved in a 10 ml volume of Dulbecco's phosphate buffered saline at pH 7.4. The final solution was dialyzed against the same buffer at 4 °C overnight.

n-butanol extraction was performed from the wet heat-killed Foc4 mycelia. With three times' extraction with 50 ml n-butanol, evaporation under vacuum to near dryness, and dissolution in 5 ml methanol.

Inhibition assay of H_2O_2, crude enzymes and n-butanol extract

Antifungal effects of liquids containing different active compounds were assayed using sterilized oxford cups that were placed on the edge of two days Foc4 mycelia and

injected with 200 µl aliquots of n-butanol extract, crude enzyme extract, or H_2O_2, respectively. The plates were further cultured at 25 °C in darkness for two days.

Analysis of guttation droplets by GC-MS

A 400 µl aliquot of guttation droplets was extracted three times with 200 µl of n-butanol supplemented with two drops of 36.5% hydrochloric acid (HCl) (v/v) for esterifying organic acid. The n-butanol extracts (600 µl) were pooled, evaporated in vacuum almost to dryness, further dried with anhydrous sodium sulfate, and then dissolved in 200 µl methanol. The chemical constituents of the mixture were identified by gas chromatography-mass spectrometry (GC-MS). GC-MS was performed using Trace DSQ (Finnigan, USA) with a Rtx-5MS column (30 m, 0.25 mm ID, 0.25 um) for analysis. Each run lasted 24 min. The initial oven temperature was maintained at 50 °C for 1.5 min, ramped up at 30 °C min^{-1} to 280 °C, and maintained for 8 min. The mass spectrometer was operated in the electron ionization mode at 70 eV at a source temperature of 250 °C and with a continuous scan from m/z 50 to 650. Compounds were identified by comparison of their mass spectra and retention indices with the mass spectral search software NIST/EPA/NIH Mass Spec. Library (Version 2.0).

Results

Enzymatic activity of guttation droplets

A total aliquot of 800 µl guttation droplets was collected from one hundred plates with dual confrontation assays between Tgui and Foc4. The results of the enzymatic activity assays are listed in Table S3-1.

Table S3-1. Enzymatic activity in guttation drops and the crude extract.

	Enzymatic activity (U·ml-1)	
	Guttation drops	Crude extract
Exochitinase	0.57±0.15	9.15 ±0.31
Endochitinase	0.50±0.04	9.60±0.40
Chitobiosidase	0.66±0.05	0.71±0.02
Chymotrypsin-like proteases	1.19±0.05	2.27±0.07
Trypsin-like proteases	1.27±0.07	1.91±0.10

GC-MS analysis of n-butanol extract of guttation droplets

Yellowish pigment components in guttation droplets could not be extracted from the mixture with *n*-butanol alone but can be successfully extracted with the addition of HCl in the buffer. To detect organic components in exudates we used the GC-MS analysis. The subsequent database search yielded two organic compounds with butyl, butyl palmitate (72%) and butyl octadecanoate (76%) (Figure S3- 1). However, several peaks we observed had either no matches to any database entry or the similarity indices were below 70%.

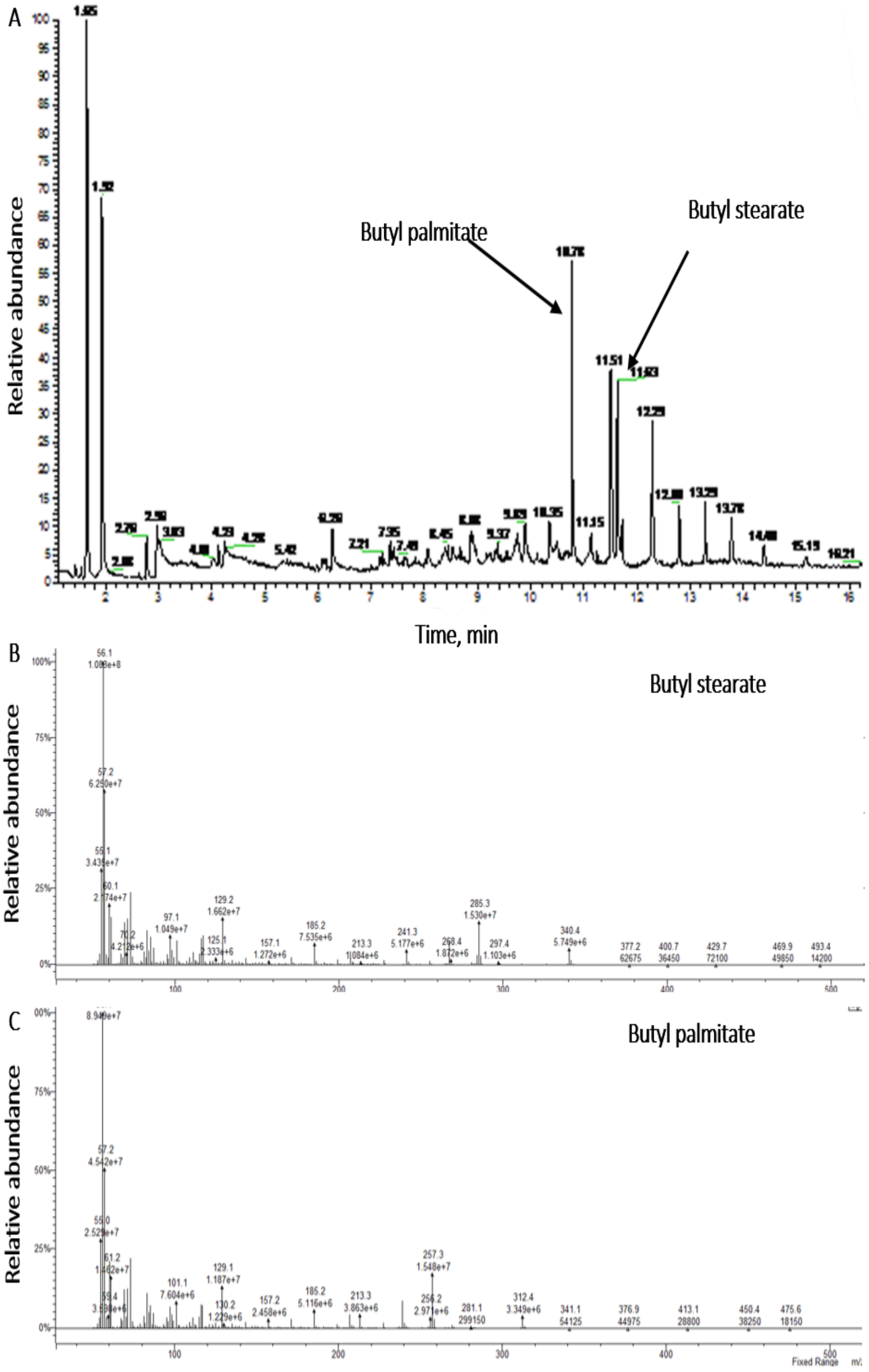

Figure S3 – 1. Analysis of compounds in the yellow mixture by GC-MS

A, The butyl searate and butyl palmitate were identified with similarity indices >70%, and their corresponding peaks are indicated in GC. B, The MS map of predicted butyl searate; C, The MS map of predicted butyl palmitete.

The effect of n-butanol extract and enzymes on Foc4

To investigate the factor contributing to growth inhibition of Foc4, antagonistic activity of crude enzymes, H_2O_2 and the n-butanol extract of Tgui secondary metabolites were investigated. Crude enzymes were harvested and showed higher activity than the chitinases, chymotrypsin-like proteases, and trypsin-like proteases detected *in situ* in the guttation droplets (Table S3-1). However, they displayed no antagonistic activity against Foc4 growth (Figure S3-2). The n-butanol extract also showed no antagonistic activity. In contrast, 20 mM H_2O_2 was observed to display an inhibition zone, and the repression against Foc4 was dose dependent (Figure S3-2).

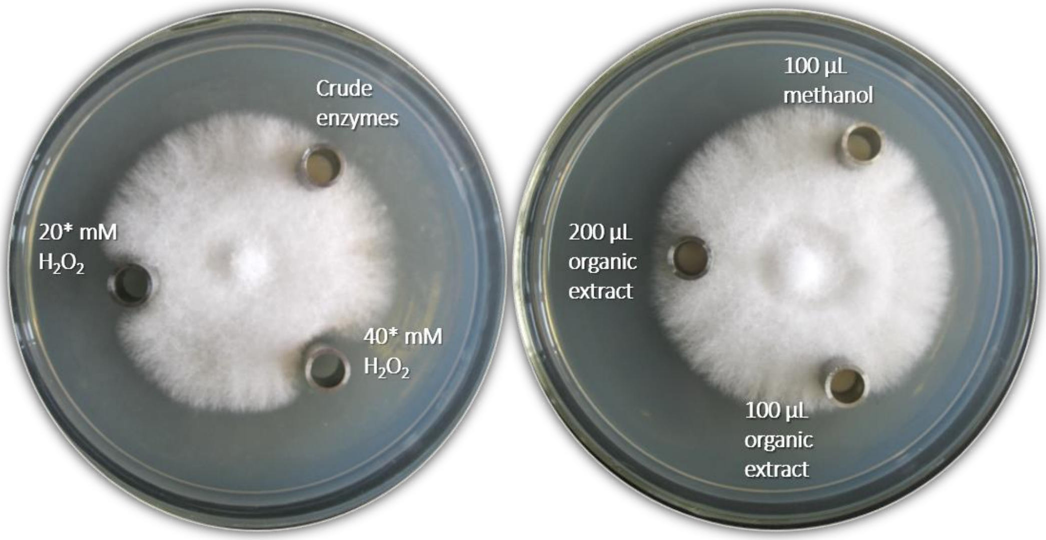

Figure S3-2 Inhibition growth of Foc4 mycelia in the presence of H_2O_2, crude enzymes and n-butanol extract. * initially applied concentration, the active concentration is lower due to the degradation of the compound during incubation time.

Crude enzymes and n-butanol extract cannot impede Foc4 growth.

The content of guttation droplets has strong proteolytic activity (Figure S3-3).

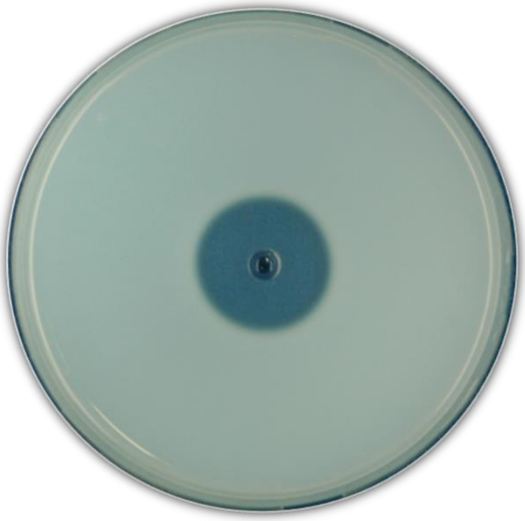

Figure S3-3. Proteolytic activity of the guttation drop content sampled from the interaction zone of *T. guizhouense* NJAU 4742 and Foc4 after 10 days of incubation. The diameter of Petri plate is 9 cm.

References

Szekeres, A., Kredics, L., Antal, Z., Kevei, F., and Manczinger, L. (2004) Isolation and characterization of protease overproducing mutants of Trichoderma harzianum. *FEMS Microbiol Lett* **233**: 215-222.

Zhou, J., Wang, Y.H., Chu, J., Zhuang, Y.P., Zhang, S.L., and Yin, P. (2008) Identification and purification of the main components of cellulases from a mutant strain of *Trichoderma viride* T 100-14. *Bioresour Technol* **99**: 6826-6833.
