# Supplementary material for: Guttation capsules containing hydrogen peroxide: an evolutionarily conserved NADPH oxidase gains a role in wars between related fungi
Source: Environ Microbiol. 2019 Apr 22;21(8):2644–58. doi: 10.1111/1462-2920.14575 (PMC6850483; doi:10.1111/1462-2920.14575)

Supporting Information S4: Effect of H_2O_2 on Foc4 and other fungi

Contents

Supporting Information S4: Effect of H_2O_2 on Foc4 and other fungi 1

Toxicity of H_2O_2 to fungi 2

Effect of H_2O_2 on spores and hyphae of FOC4 3

Toxicity of H_2O_2 to fungi

Figure S4-1 H_2O_2 inhibiting growth of Foc4 and other fungi.

Fungi used in this assay are listed in Supportive information S8.

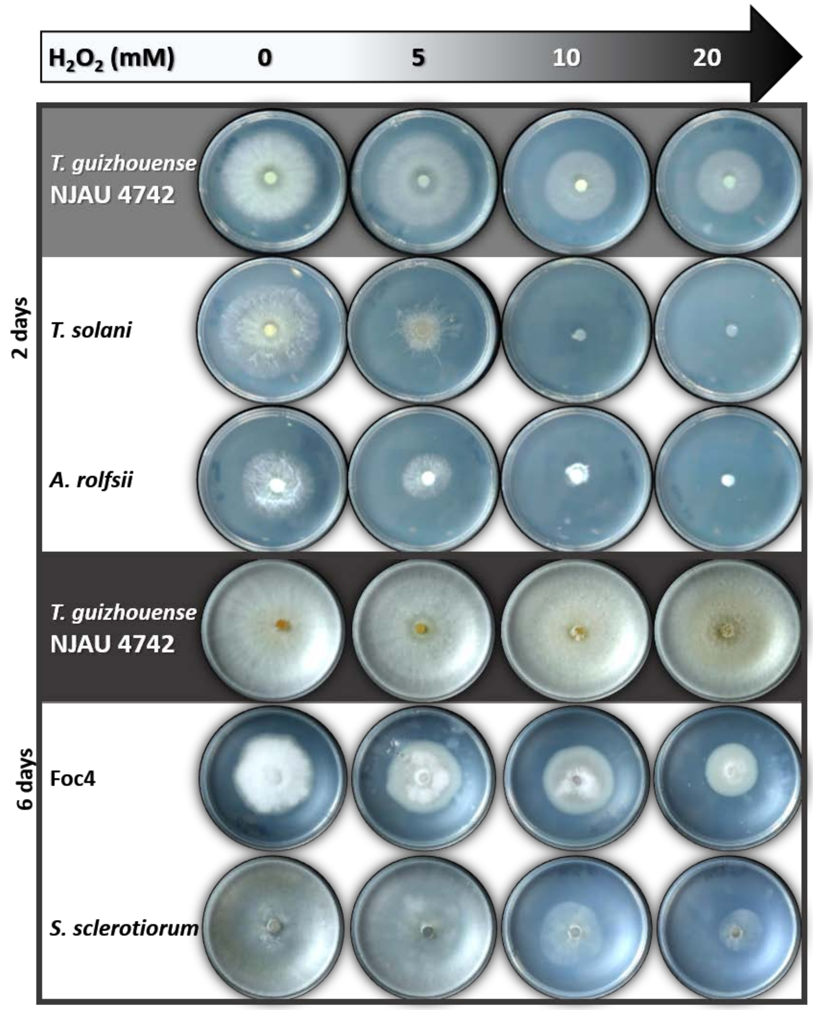

Effect of H_2O_2 on spores and hyphae of FOC4

Figure S4-2. Effect of H_2O_2. On FOC4. Germinating spores and hyphae of FOC4 were treated with 10 mM H_2O_2 for two hours at 28 °C and stained with propidium iodide. The images were made using Leica DMi8 fluorescence microscope (Germany), scale bar, 50 μm. The red fluorescence allows revealing the loss of plasma membrane integrity (pointed by arrows) due to the treatment by H2O2.

Before the test, Foc4 spores were cultivated in potato dextrose broth for 12 hours. Mycelium was harvested from the edges of FOC4 colony grown on PDA for three days.

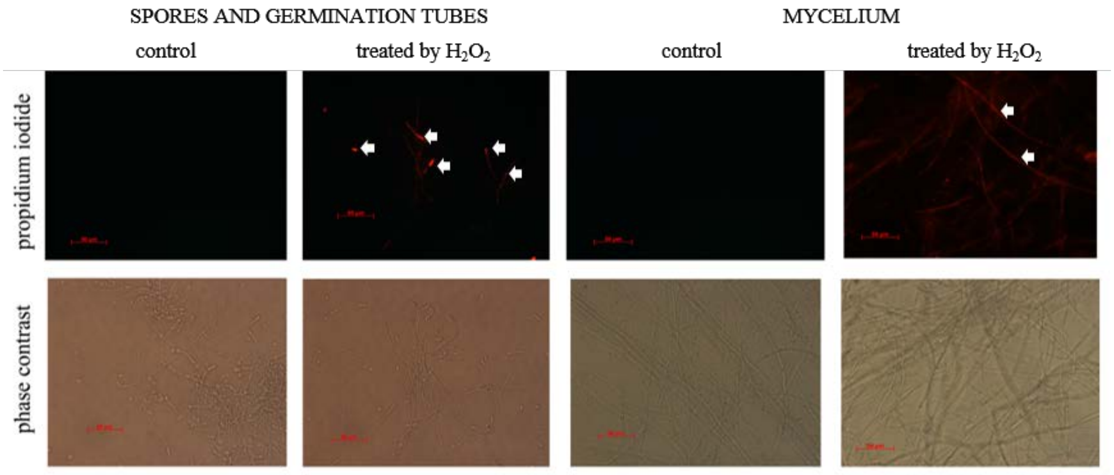

Supplement: Supplementary file 4 — Supporting Information S4. Effect of H2O2 on Foc4 and other fungi [file EMI-21-2644-s004.pdf]
