# Supplementary material for: Guttation capsules containing hydrogen peroxide: an evolutionarily conserved NADPH oxidase gains a role in wars between related fungi
Source: Environ Microbiol. 2019 Apr 22;21(8):2644–58. doi: 10.1111/1462-2920.14575 (PMC6850483; doi:10.1111/1462-2920.14575)
Supplement: Supplementary file 6 — Supporting Information S6. Transformation of T. guizhouense NJAU 4742 and verification of the mutants [file EMI-21-2644-s006.pdf]

Supporting Information S6: Transformation of *T. guizhouense* NJAU 4742 and verification of the mutants

Contents

Materials and Methods 1
1. Constructing deleting, complementary and overexpressing plasmids 1
2. Transformation of *T. guizhouense* 2
3. Screening for positive deletion mutants 3
4. Extraction of RNA and quantitative PCR 4
5. Southern blot 7
Results 7
1. Mutants of *nox* genes 7
2. Southern blot 10
References 11

Materials and Methods

1. Constructing deleting, complementary and overexpressing plasmids

The open reading frames of genes *nox1* (OPB44254), *nox2* (OPB36463), and *nor1* (OPB40972) were retrieved from the genome of *T. guizhouense* NJAU 4742 (NCBI GenBank: LVVK00000000.1), respectively. Gene *nox1* 5' flanking fragment was amplified with primers 5F-*nox1* and 5R-*nox1*. The 3' flanking fragment was obtained with primers 3F-*nox*1 and 3R-*nox1* (see all primers in Table S6-1). Hygromycin cassette was amplified with *hyg-nox1*-fw and *hyg-nox1*-rev from the plasmid pBC-*hyg* (Derntl *et al.*, 2015). These three PCR products were ligated together and inserted in to the BamHI site in pUC19 (TaKaRa) using the In-Fusion HD cloning kit (Clontech), constructing the plasmid pUC19-*nox1* used for knockout gene *nox1*. Primers 5F-*nox*2, 5R-*nox2,* 3F-*nox2*, 3R-*nox2 hyg-nox2-fw* and *hyg-nox2-rev* were used to construct the plasmid pUC19-*nox2* for deletion of *nox2.* Primers 5F-*nor1*, 5R-*nor1*, 3F-*nor1*, 3R-*nor1*, *hyg-nor1-fw and hyg-nor1-rev* were used to construct the plasmid pUC19-*nor1* for deletion of *nor1*. The *nox2*

deleting cassette was amplified with primers 5F-*nox2* and 3R-*nox2* and used for transformation. The *nor1* deleting cassette was obtained with primers 5F-*nor1* and 3R-*nor1*.

For overexpression of *nox1* in *T. guizhouense* NJAU 4742 (WT), 550 bp of the *nox1* terminator, amplified from the WT genomic DNA with primers OEinfu-ter-fw and OEinfu-ter-rev and the coding region of *nox1*, cloned from cDNA with primers OEinfu-*nox1*-fw and OEinfu-*nox1*-rev, inserted to the site between SalI and ClaI of the linearized plasmid P*cdna1*-*cel7b* harboring *T. reesei cdna1* promoter (Uzbas *et al.*, 2012), with In-Fusion HD Cloning Kit. The resulting plasmid p*cdna1*-*nox1* was used for the transformation (Figure S6-1).

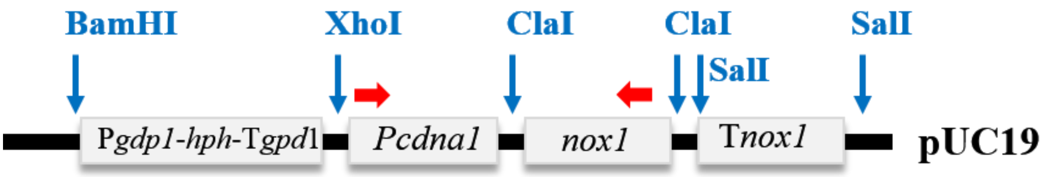

Figure S6-1 The construction of *nox1* overexpressing plasmid.

The plasmids contained the Hygromycin B expression cassette with glycerol-3-phosphate dehydrogenase promoter (P*gdp1*) and glycerol-3-phosphate dehydrogenase terminator (T*gpd1*), followed by the *cDNA1* promoter region (P*cdna1*), the *nox1* coding region and *nox1* terminator region (T*nox1*).

For complementation of Δ*nox1* mutants, a genecticin resistance cassette (*neo* cassette) was amplified from the plasmid pPki-Gen (Seiboth *et al.*, 2012) using the primers com-neo-fw, and com-neo- rev, and a 4.4-kb *nox1* genomic fragment amplified with primers comG*nox1*-fw and comG*nox1*-rev, were inserted to the BamHI site of the linearized plasmid pUC19 using the In-Fusion HD cloning kit, getting the plasmid pUC19-*neo*-*nox1*. The resulting vector was transformed into the Δ*nox1* background.

2. Transformation of *T. guizhouense*

Spores were harvested by pouring 12 ml of a freshly sterilized physiological salt solution (0.8-0.9 % NaCl, 0.05% Tween20) on a Petri plate with conidiating culture. The resulting

suspension was collected and filtered through the glass wool in a 1.5 ml tube. 100 µl of spore solution was spread out on a cellophane covered plate. The protoplasting solution was prepared by dissolving 0.15 g lysing enzymes (Sigma-Aldrich) in 20 ml solution A (1.2 M sorbitol, 0.1 M KH_2PO_4, pH 5.6) and filter sterilized. After incubation at 28 °C for 10-12 hours in darkness, 2-3 ml of the protoplasting solution was used to wash the geminating spores on the cellophane and harvested in a 50 ml tube. The harvested protoplast suspension was filtered through a layer of gauze for removing young mycelia and incubated at 28 °C for 120 min with gentle agitation. After centrifugation for 10 min, 3500 rpm under 4 °C, supernatant was discarded and protoplats were re-suspended with ca. 1 ml solution B (1 M sorbitol, 50 mM $CaCl_2$, 10mM Tris-HCl, pH 7.5). The protoplasts were ready after the subsequent centrifugation and the end concentration of protoplasts should be more than 10^8 cells/ ml by dilution with solution B.

200 µl protoplast suspension, 10 µl purified DNA (concentration should be more than 100 ng/µl) and 50 µl PEG were mixed in a 15 ml tube on ice. After gentle shaking and incubation for 20 min on ice, another 2 ml PEG (room temperature) was added and mixed gently again. 3 ml solution B was added after 5 min incubation at room temperature, and mixed gently. 1 ml mixture was spread on PDA plus 1M sucrose covered by the strip-type chromatography paper (Whatman) and incubated for 16 hours. Chromatography paper strips were transferred to PDA containing 100 ug/ml hygromycin B and incubated at 28 °C for 2 days. Single colonies along the strips were picked up and cultured for another 2 day. The mutants were directly screened for positive deletion mutant.

3. Screening for positive deletion mutants

The PCR verification of transformants was conducted according to the manual of Thermo Scientific Phire Plant Direct PCR kit (Thermo fisher scientific). A fragment of the transformant mycelia was transferred into a tube containing 20 µL dilution buffer by 10 µL pipette tips and incubated in 98 °C for 1 min. The supernatant was used for PCR template after centrifuging at 3000 rpm in room temperature. The primers probe1-*nox1*-fw and probe1-*nox1*-rev were used for amplification for upstream sequence of the

positive mutant. The primers probe2-*nox1*-fw and probe2-*nox1*-fw-rev were used for screening for downstream sequences of the positive mutant. The schematic drawing for screening positive mutants is shown in Figure S6-2. After screening and purification with single spore isolation, genomic DNA of positive mutants was extract with DNeasy Plant Mini Kit (Qiagen). The primers probe3-*nox1*-fw and probe3-*nox1*-rev were applied to verify the deletion mutant. The same strategy was used for the deletion of *nox2* and *nor1*.

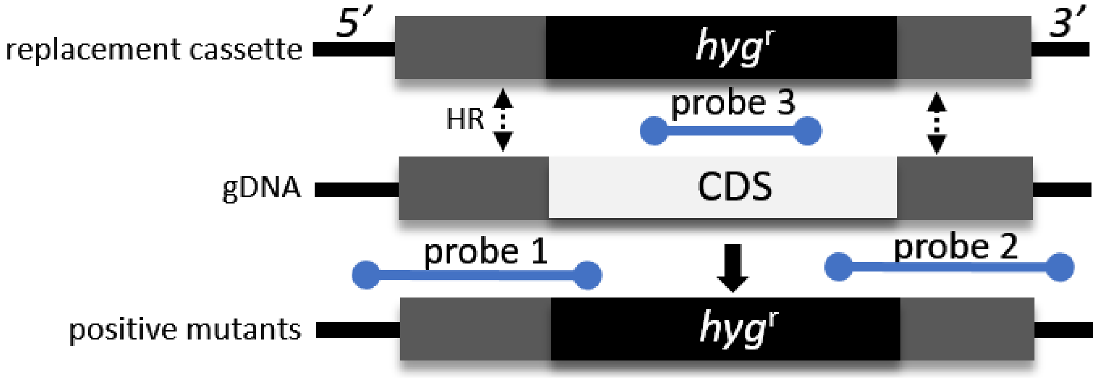

Figure S6-2 The strategies of deleting genes and screening positive mutants

(HR, homology recombination, CDS, coding sequence, hyg^r – hygromycin resistance)

4. Extraction of RNA and quantitative PCR

T. guizhouense strains mycelia were frozen, lyophilized, and ground, and then RNA was extracted with the RNeasy Plant Mini Kit (Qiagen) according to the manufacturer's instructions. Optional on-column DNase digestion with RNase-free DNase Set (Qiagen) was used to eliminate genomic DNA. cDNAs were synthesized from 1 µg of total RNA using the Primerscript RT reagent Kit with gDNA eraser (TaKaRa) with an oligo (dT) primer. Two microliters of the cDNA were used in the subsequent PCR. Quantitative real-time PCR was performed using an ABI Prism 7500 sequence detection system (ABI, USA) with SYBR premix ExTaqTM II (Takara). Three-step PCR reactions were performed in triplicate in a total volume of 25 µl for 40 cycles according to the operating

instructions. CT values were calculated using the β-tubulin gene as an endogenous control, and data were expressed as $2^{-\Delta\Delta Ct}$ (Livak and Schmittgen, 2001). Three replicates were used for statistical analysis.

Table S6-1 Primers used in this study.

Primers	Sequence（5'-3'）	Comments
nox1-fw	CACCACCTGTTCATCCC	qPCR for *nox1*
nox1-rev	GTCAAATGGAGAGAATCC	
5F-nox1	GACGGCCAGTGAATTC TGCTACTCCCTGACTAACTCGGT	*nox1* deletion plasmid
5R-nox1	TCTCGGATCCGGTCGTTCAAGGCAGAATACAAA	
3F-nox1	TACCCTCGAG GTCGGAGAGTAGCAGCATCATG	
3R-nox1	TGATTACGCCAAGCTTGAGAGCAGTGTTACGATTTGAGC	
hyg-nox1-fw	TTGAACGACC GGATCCGAGAGCTACCTTACATC	
hyg-nox1-rev	ACTCTCCGACCTCGAGGGTACTATGGCTTAGATG	
probe1-nox1-fw	TTCCACGGTTCAATAGAGGCA	screening *nox1* mutant
probe1-nox1-rev	CGTCCATCACAGTTTGCCAGT	
probe2-nox1-fw	CCGATCTTAGCCAGACGAGC	
probe2-nox1-rev	AGTCGAATAGGCAATAGCACCA	
probe3-nox1-fw	GCTCAAGCCAAGTTCTACGACG	
probe3-nox1-rev	CTCGAGACCGCCAAGATAGG	
OEinfu-ter-fw	AAGTGCCTACGATTGC	*nox1* overexpression
OEinfu-ter-rev	ATGCCTGCAGGTCGACATTCTAAATCGGGCATCCCAG	
OEinfu-nox1-fw	CAACTTCTCTCATCGATATGGCTGGCCAACTCACATA	
OEinfu-nox1-rev	GCAATCGTAGGCACTTTTAAAAGTTTTCTTTGCCCCA	
comGnox1-fw	cgactctagaggatcTGTGCCTGAGATTGCGCAC	*nox1* reverse complementation
comGnox1-rev	CAAAGCCGTTTCTTTGCCCCAGACGAACGAGA	
com-neo-fw	AAAGAAACGGCTTTGATTTCCTTCAGGTCA	
com-neo-rev	cggtacccggggatcTCTAAACAAGTGTACCTGTGCATTCTGG	
5F-nox2-fw	GACGGCCAGTGAATTCAGGCGTCGATAAGCTGAAGG	*nox2* deletion plasmid

5F-nox2-rev	TCTCGGATCCCAGGCAGTATCGGTGGTTCA	
hyg-nox2-fw	ATACTGCCTGGGATCCGAGAGCTACCTTACATC	
hyg-nox2-rev	TGCGTCCAGCCTCGAGGGTACTATGGCTTAGATG	
3F-nox2-fw	TACCCTCGAG GCTGGACGCATACACGTAACTTA	
3f-nox2-rev	TGATTACGCCAAGCTT GTCCGTTCTCACTGTAATGCTCG	
probe1-nox2-fw	CATGAGATTAGCGCCAGGTC	Screening *nox2* mutant
probe1-nox2-rev	AGGGCTCGTTGAATCCCTC	
probe2-nox2-fw	CCATCGCTTTCTATGATTAGGTAC	
probe2-nox2-rev	AATACGGGCTGCGAAACTG	
probe3-nox2-fw	GGTTACTGACTCAATGCGAACAG	
probe3-nox2-rev	GAGGGCAATCAGCATCACG	
5F-nor1-fw	GACGGCCAGTGAATTCGGCAGTGATGATGAGCAAGATG	*nor1* deletion plasmid
5F-nor1-rev	TCTCGGATCCGCTCCCTGCGTGTATCTTCTCT	
hyg-nor1-fw	CGCAGGGAGCGGATCCGAGAGCTACCTTACATC	
hyg-nor1-rev	CGCAGCATGCCTCGAGGGTACTATGGCTTAGATG	
3F-nor1-fw	TACCCTCGAGGCATGCTGCGATTTGGTCAG	
3f-nor1-rev	TGATTACGCCAAGCTT TGCACATCCGCAAACTAGCAC	
probe1-nor1-fw	CGATCGACTACGCGGAGGA	Screening *nor1* mutant
probe1-nor1-rev	CTGCTGGTATAGTACGAGCCTACC	
probe2-nor1-fw	GAGAAAGCGTTGCCATCACA	
probe2-nor1rev	GATCTCACCAGAGGGCTTCG	
probe3-nor1-fw	CATCCTAGGACGGGAGGAACA	
probe3-nor1-rev	TTGGATGCCTGGCTTGACC	
southern-probe-fw	AAATTGCCGTCAACCAAG	Southern blot
southern-probe-rev	GTCCTGCGGGTAAATAGC	
β-tubulin	TTCTTGCATTGGTACACTAGCG<br>ATCGTTCATGTTGGACTCAGCC	qPCR

5. Southern blot

Genomic DNA was extracted from the fresh mycelia using DNeasy Plant Mini Kit (Qiagen, Germany). Genomic DNA was digested with Hind III, fractionated in a 0.8% agarose gel at 30 V for 12 h and blotted onto a Hybond N^+ membrane (Amersham Pharmacia Biotech, England). Interior probe was amplified from Hygromycin cassette in plasmid pBC-*hyg*, with the primer pair southern-probe-fw and southern-probe-rev (Table S6-1) . Southern blots were carried out using the DIG DNA Labelling and Detection Kit (Roche Applied Sciences), followed by an anti-DIG detection according to the manufacturer's protocol.

Results

1. Mutants of *nox* genes

After purification and verification, one Δ*nox1* mutant was confirmed by PCR (Figure S6-3), two Δ*nox2* mutants were got (Figure S6-4), and six Δ*nor1* mutants were obtained (Figure S6-5). The efficiency of homologous recombination with PCR fragments is higher than transformation with deleting plasmid. Five reverse complemented mutants were confirmed by RT-PCR (Reverse Transcription PCR) and used for research (Figure S6-6). The *nox1*OE1 mutant was selected with the highest level of transcription of *nox1* from five overexpressing mutants, by Real-Time quantitative PCR (Figure S6-7).

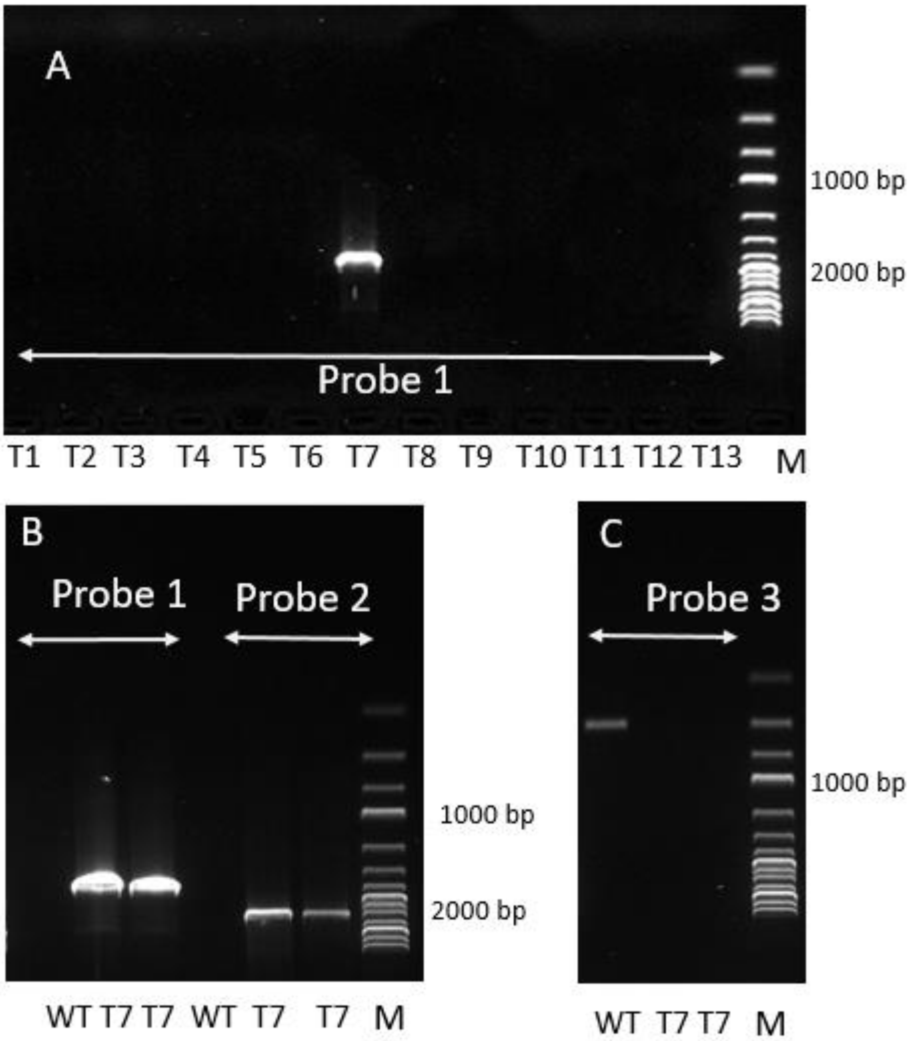

Figure S6-3 Screening and verification of Δ*nox1* mutants.

A. The primers for probe 1 and probe 2 were used for screening positive Δ*nox1* mutants in 13 transformants (from T1 to T13); B. The primers for probe 3 were applied for verification of Δ*nox1* mutants. M, 1kb marker. WT, the wild strain *T. guizhouense* NJAU 4742.

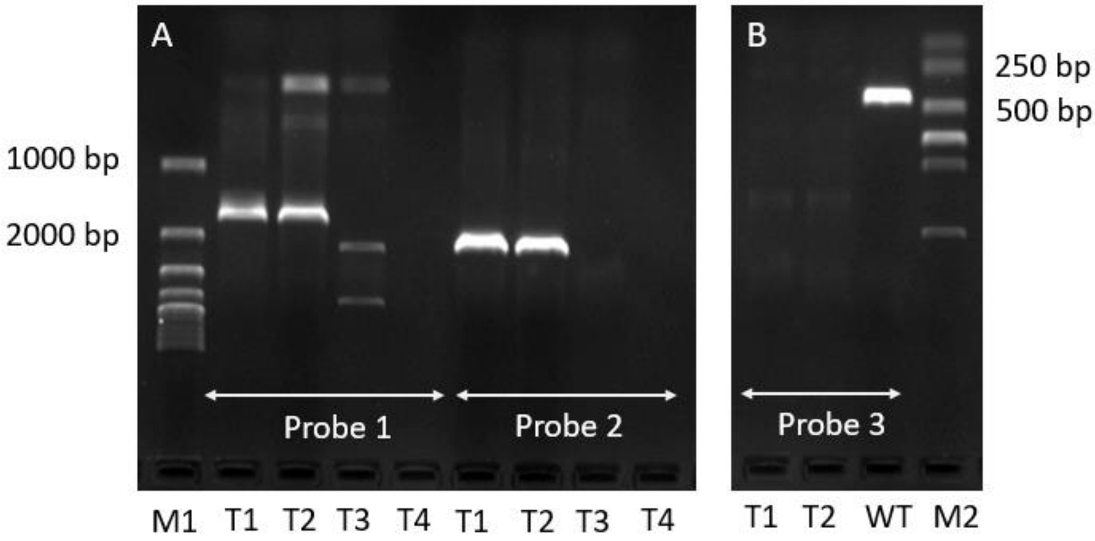

Figure S6-4 Screening and verification of Δ*nox2* mutants.

A. The primers for probe1 and probe2 were used for screening positive Δ*nox2* mutants in 4 transformants (T1, T2, T3 and T4); B. The primers for probe3 were applied for verification of Δ*nox2* mutants. M1, 1kb marker. M2, DL2000 marker. WT, the wild strain *T. guizhouense* NJAU 4742.

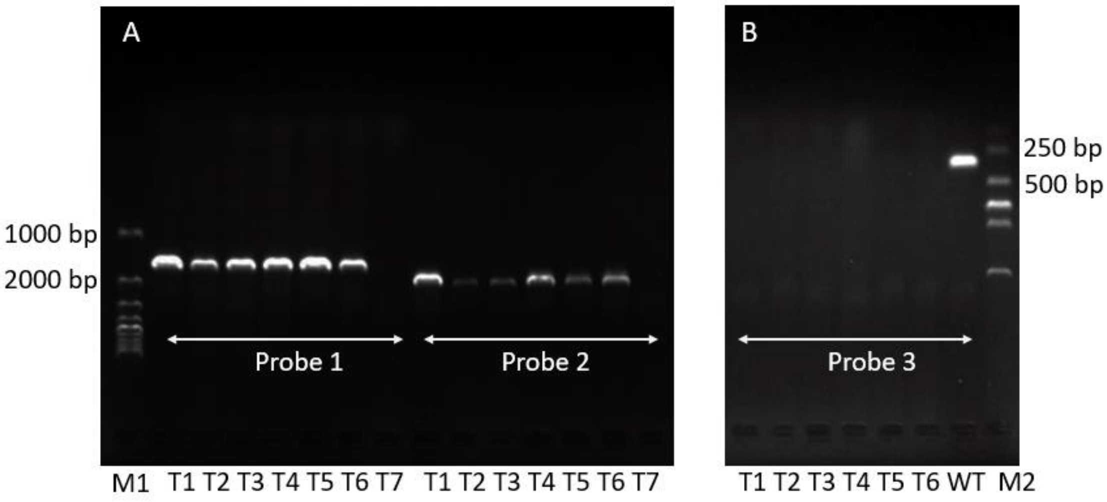

Figure S6-5 Screening and verification of Δ*nor1* mutants

A. The primers for probe 1 and probe 2 were used for screening positive Δ*nor1* mutants in 7 transformants (T1, T2, T3, T4, T5, T6 and T7); B. The primers for probe 3 were applied for verification of Δ*nor1* mutants. M1, 1kb marker. M2, DL2000 marker. WT, the wild strain *T. guizhouense* NJAU 4742.

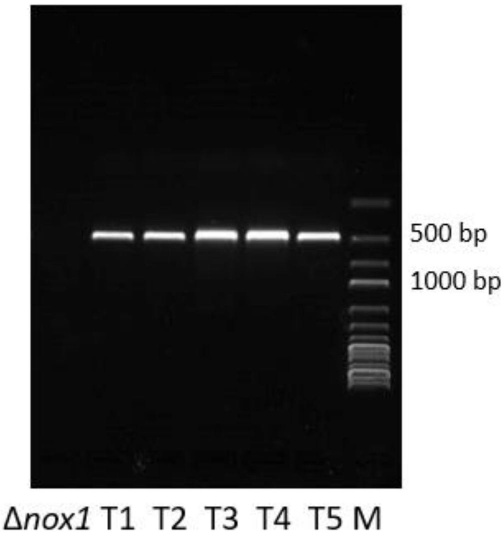

Figure S6-6 Verification complementary of Δ*nox1* mutants by RT-PCR.

Five Δ*nox1* complementary mutants were verified by RT-PCR. M, 1kb marker.

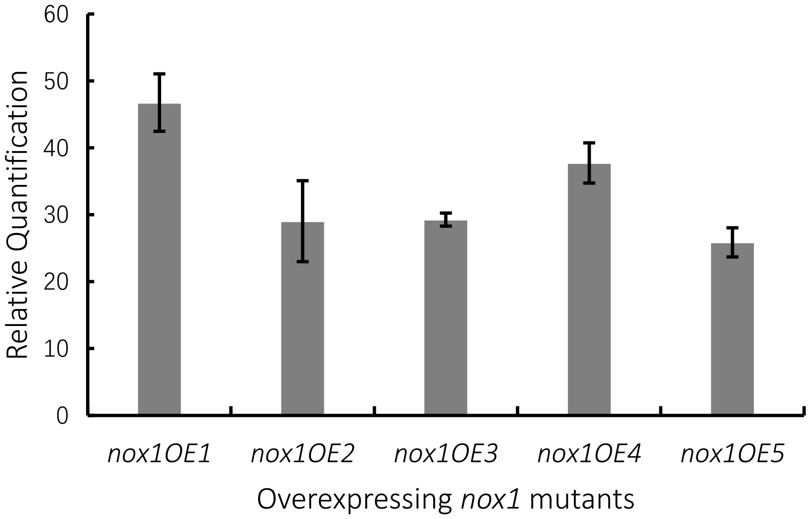

Figure S6-7 Verification of Δ*nox1* complementary mutants by qPCR

Values ($2^{-\Delta\Delta Ct}$) correspond to relative measurements against the transcript in the NJAU 4742 obtained from PDA ($2^{-\Delta\Delta Ct}=1$). β-tubulin gene was used as an internal reference.

2. Southern blot

By Southern bolt (Figure S6-8), deleting *nox1* cassette and *nox2* cassette was confirmed to exist as a single copy. There were two copies of overexpressing *nox1* cassette in *nox1*OE1. The wild strain *T. guizhouense* NJAU 4742 was used as a negative control.

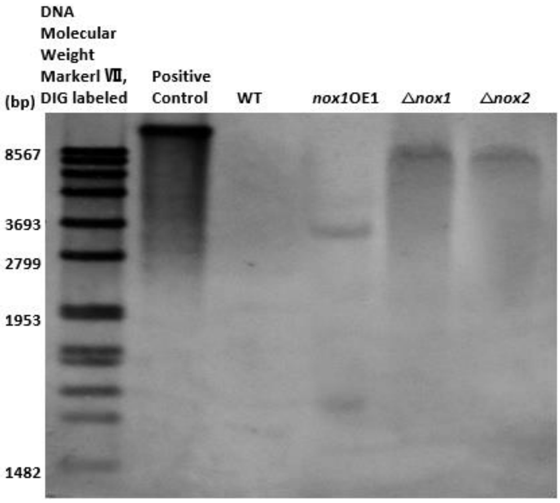

Figure S6-8 Southern blot of the selected mutants

WT, the wild strain *T. guizhouense* NJAU 4742. The interior probe was amplified from Hygromycin cassette in plasmid pBC-hyg, with the primer pair southern-probe-fw and southern-probe-rev. The wild strain *T. guizhouense* NJAU 4742 was used as a negative control.

References

Derntl, C., Kiesenhofer, D.P., Mach, R.L., and Mach-Aigner, A.R. (2015) Novel Strategies for Genomic Manipulation of *Trichoderma reesei* with the Purpose of Strain Engineering. *Applied and Environmental Microbiology* **81**: 6314-6323.

Livak, K.J., and Schmittgen, T.D. (2001) Analysis of relative gene expression data using real-time quantitative PCR and the $2^{-\Delta\Delta CT}$ method. *Methods* **25**: 402-408.

Seiboth, B., Karimi, R.A., Phatale, P.A., Linke, R., Hartl, L., Sauer, D.G. et al. (2012) The putative protein methyltransferase LAE1 controls cellulase gene expression in *Trichoderma reesei*. *Molecular Microbiology* **84**: 1150-1164.

Uzbas, F., Sezerman, U., Hartl, L., Kubicek, C.P., and Seiboth, B. (2012) A homologous production system for *Trichoderma reesei* secreted proteins in a cellulase-free background. *Applied Microbiology and Biotechnology* **93**: 1601-1608.
