# Supplementary material for: Guttation capsules containing hydrogen peroxide: an evolutionarily conserved NADPH oxidase gains a role in wars between related fungi
Source: Environ Microbiol. 2019 Apr 22;21(8):2644–58. doi: 10.1111/1462-2920.14575 (PMC6850483; doi:10.1111/1462-2920.14575)

Supplementary Material S7 Phenotype of *T. guizhouense* NJAU 4742 *nox*-mutants

Contents

Dual confrontation assays between Tgui and its *nox*-mutants 2

Cryo-SEM analysis of Tgui$_{\Delta nox1}$ mutant 3

Cryo-SEM analysis of Tgui$_{noxOE}$ mutant 5

Production of H_2O_2 by *T. guizhouense* NJAU 4742 *nox*-mutants 7

Dual confrontation assays between Tgui and its *nox*-mutants

Figure S7-1 Impaired, recovered and enhanced mycoparasite activity of *nox* muants.
Results of the dual confrontation assays between *T. guizhouense* (inoculated on the down of every plate) WT (NJAU 4742), Δ*nox1*, Δ*nox2*, Δ*no1R*, Δ*nox1::nox1* and *nox1*OE1, respectively. Cultures were incubated on GSM at 25°C for 10 days in darkness. Host fungi were inoculated on the top of the plate. DNA Barcodes of the fungi used for this assay are provided in Supporting Information S9.

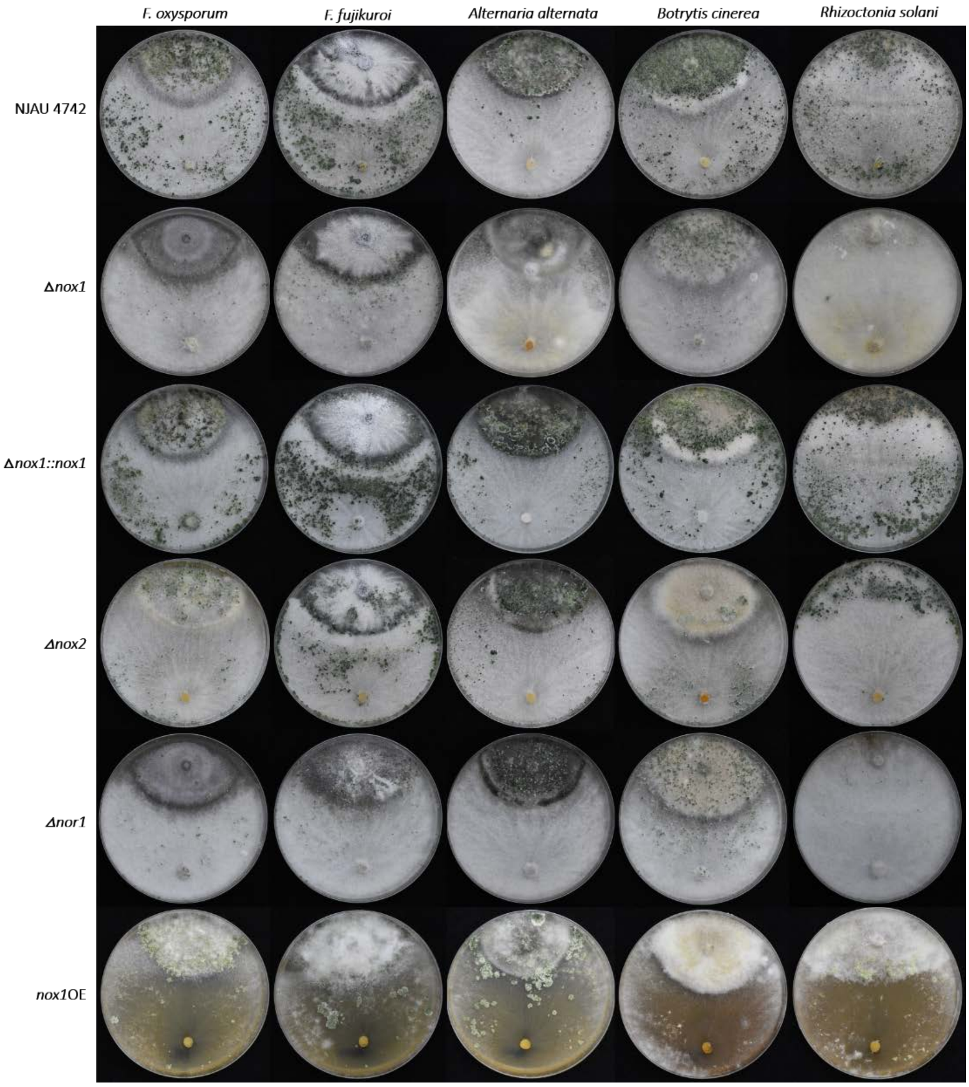

Cryo-SEM analysis of Tgui$_{\Delta nox1}$ mutant

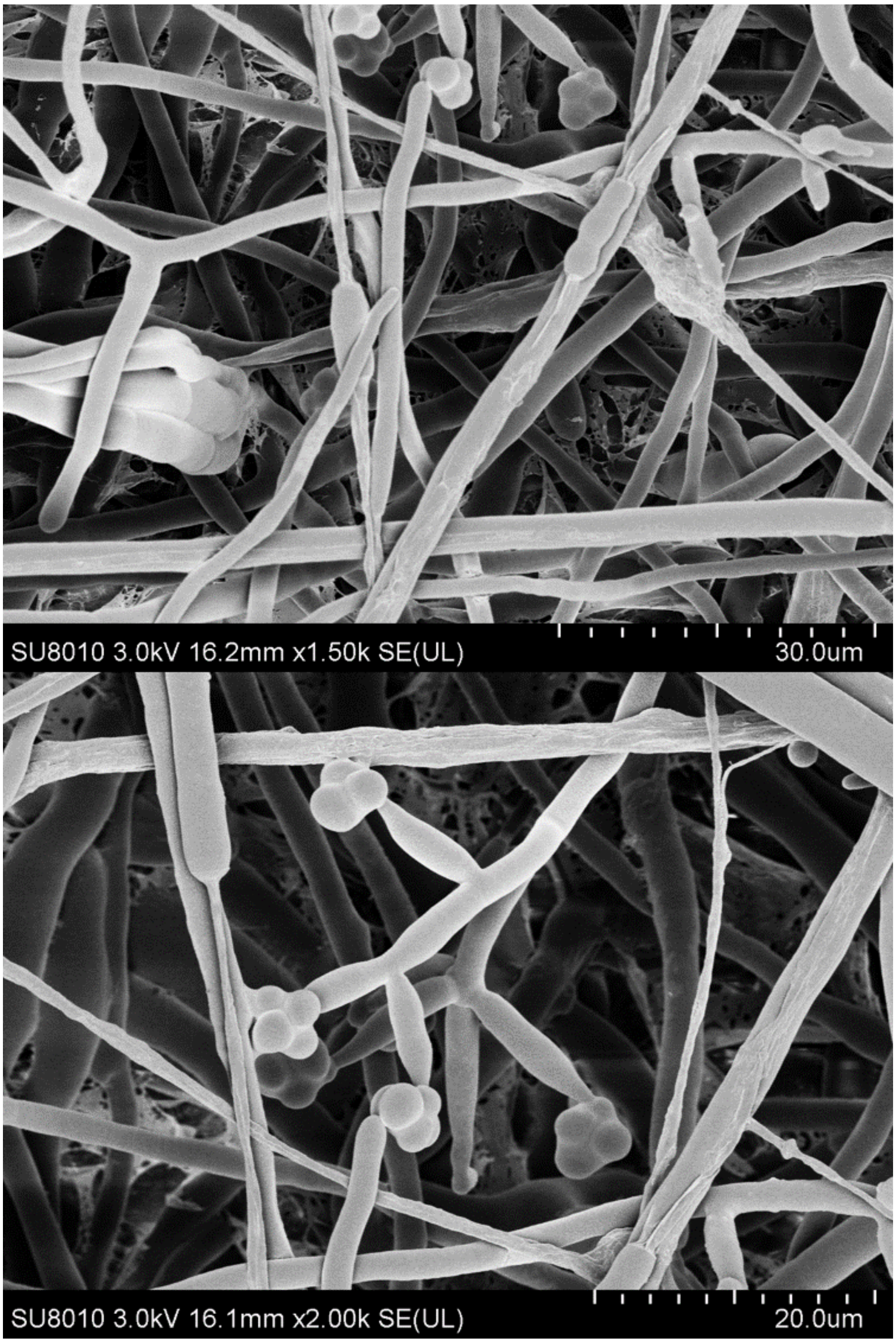

Figure S7-2 All images were taken from the contact zone of the dual confrontation assays on cellophane-covered GSM incubated at 25℃ in darkness. The observation was performed shortly after contact.

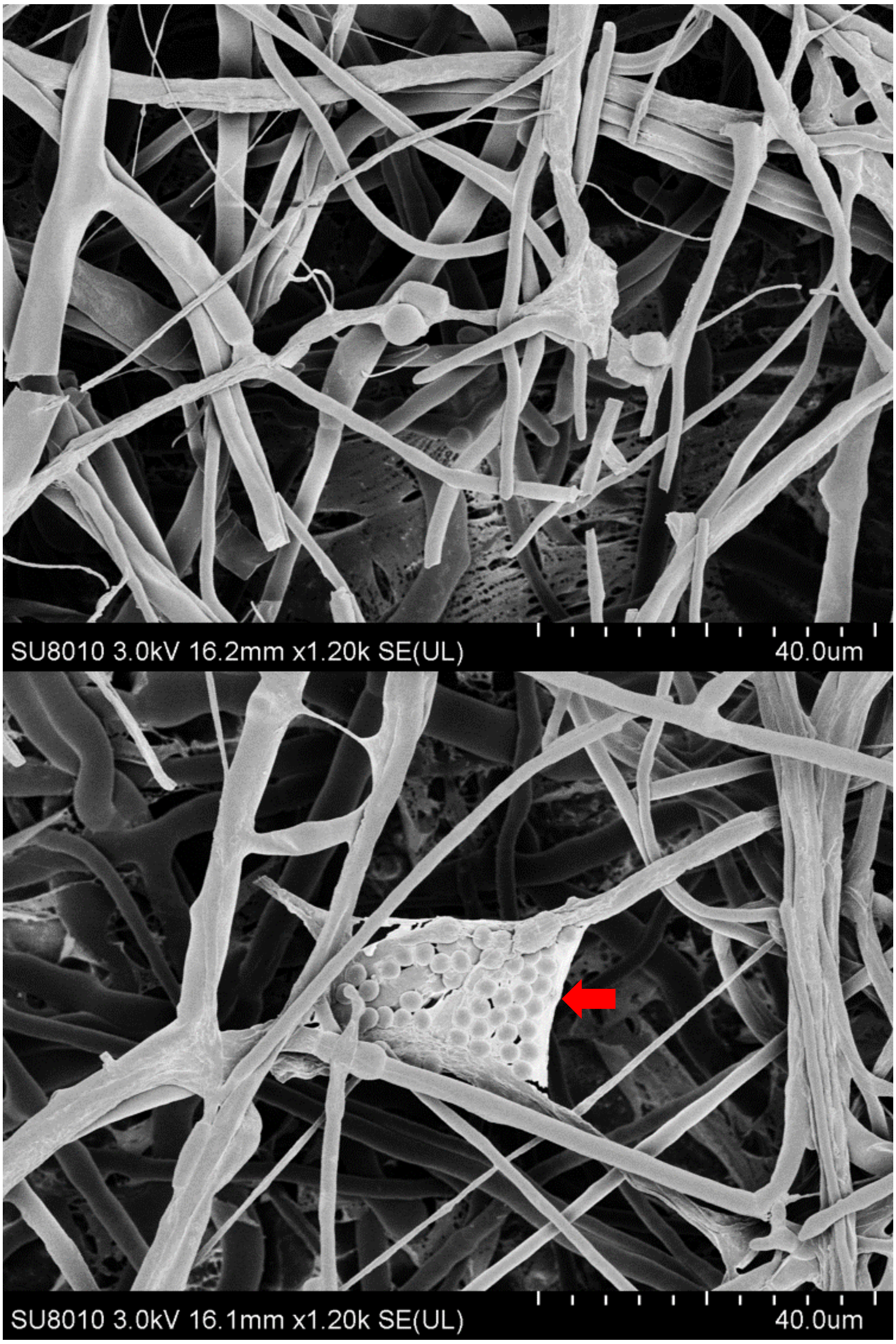

Figure S7-3. Red arrow points to the bag-like structure filled with conidia of Tgui$_{\Delta nox1}$. No interaction with hyphae of Foc4 were detected. Red arrows indicate abnormally thin hyphae of Tgui$_{\Delta nox1}$

Cryo-SEM analysis of $\mathrm{Tgui}_{\mathit{noxOE}}$ mutant

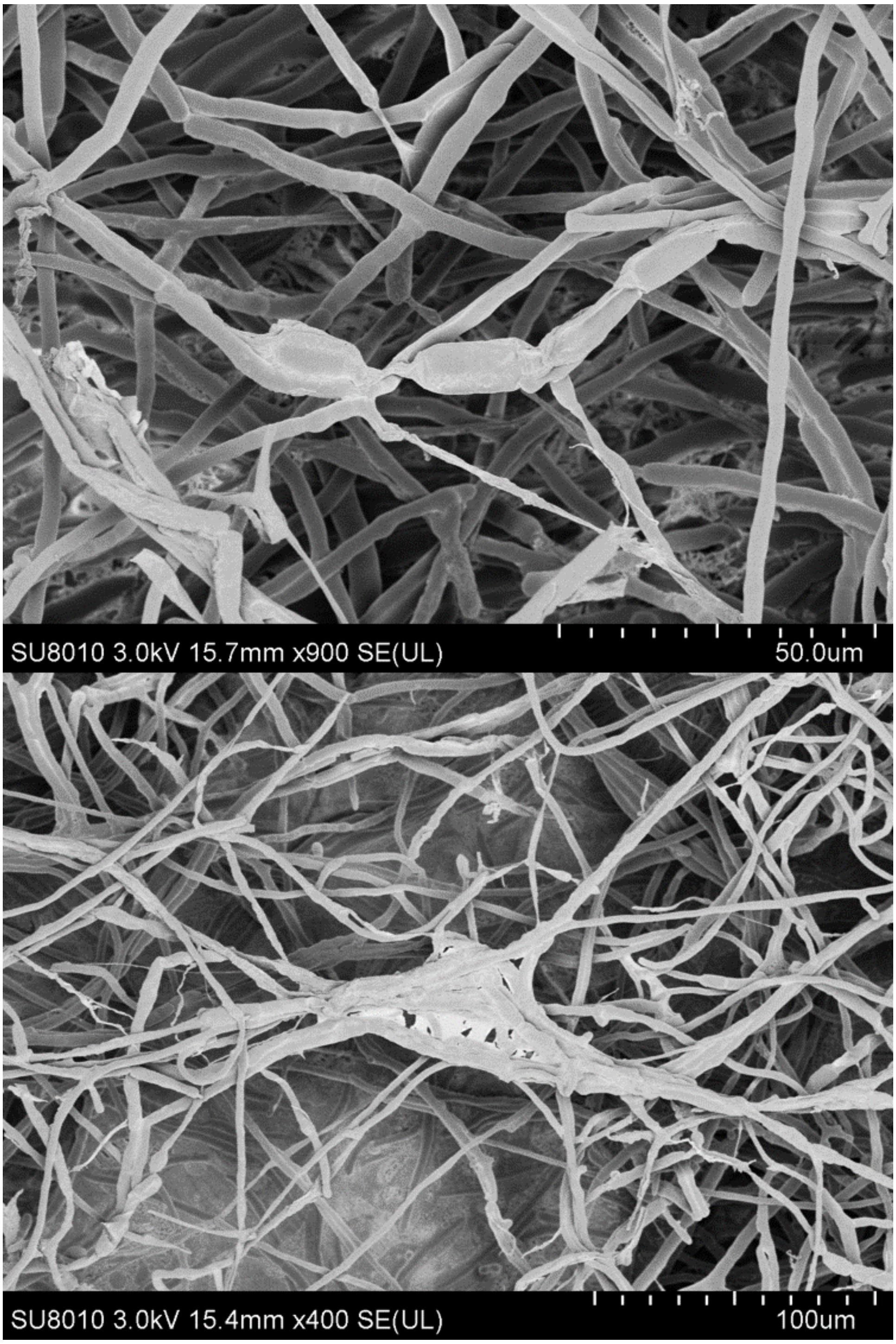

Figure S7-4 All images were taken from the contact zone of the dual confrontation assays on cellophane-covered GSM incubated at 25℃ in darkness. The observation was performed shortly after contact.

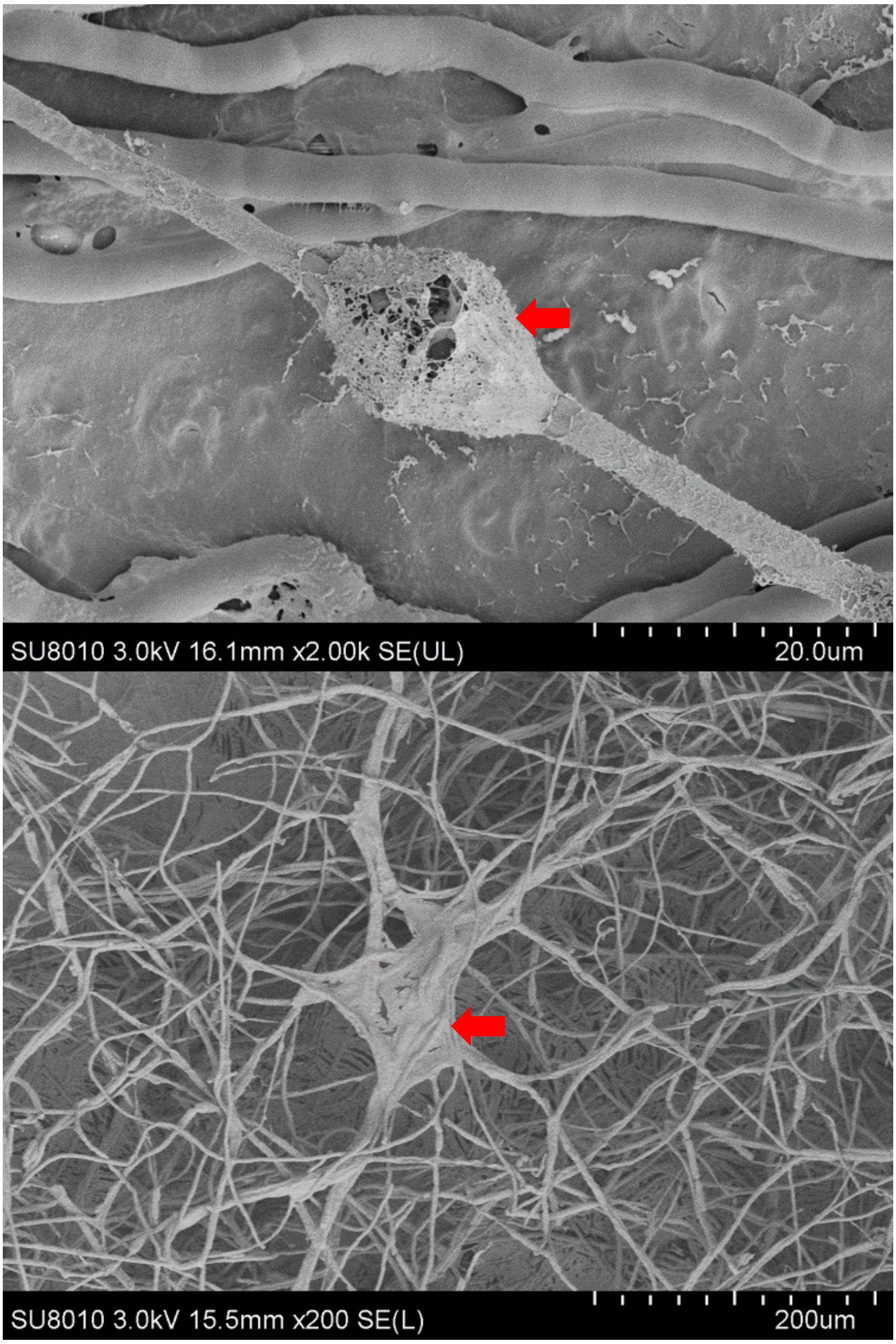

Figure S7-5. Guttation droplets on single hyphae of Tgui$_{noxOE}$ mutant (above) or in interacting hyphae between Tgui and Foc4 (below). Red arrow points to the bag-like structures.

Production of H_2O_2 by *T. guizhouense* NJAU 4742 *nox*-mutants

Figure S7-6. The detection of $O_2^{\bullet-}$ production on Petri plates was performed as described using nitro blue tetrazolium (NBT) in Petri plates after 72 hours of incubation on PDA plates in darkness. For the fluorescence assays of H_2O_2 produced by fungal hyphae, an agar block (1 cm^2) was excised from the confrontation zone and incubated in the presence of 2.5 µg/ml 2',7'-dichlorodihydrofluorescein diacetate (H_2DCFDA; Molecular Probes, Thermo Fisher, USA) for 10 min. Then, the interacting hyphae were transferred to a microscopy slide and observed by a fluorescence light microscope (TCS SP8, Leica, Germany) using 460 to 490 nm excitation and 500 to 550 nm emission.

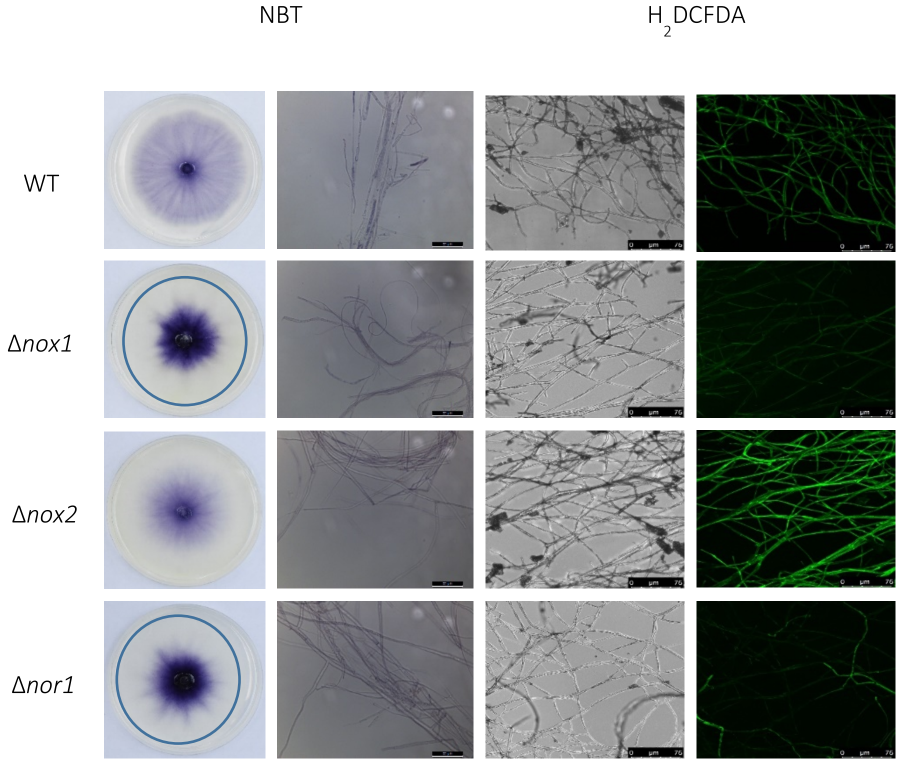

Supplement: Supplementary file 7 — Supporting Information S7. Phenotype of T. guizhouense NJAU 4742 nox‐mutants [file EMI-21-2644-s007.pdf]
