# Supplementary material for: Guttation capsules containing hydrogen peroxide: an evolutionarily conserved NADPH oxidase gains a role in wars between related fungi
Source: Environ Microbiol. 2019 Apr 22;21(8):2644–58. doi: 10.1111/1462-2920.14575 (PMC6850483; doi:10.1111/1462-2920.14575)
Supplement: Supplementary file 8 — Supporting Information S8. Transcriptomic analysis [file EMI-21-2644-s008.docx]

Supporting Information S8: Transcriptomic protocol and the functional enrichment analysis of differentially expressed genes based on Gene Ontology

Contents

[Extraction of the total RNA 1](#_Toc1848884)

[cDNA synthesis and Library preparation 1](#_Toc1848885)

[Deep sequencing 2](#_Toc1848886)

[Data analysis 3](#_Toc1848887)

[Functional enrichment analysis of differentially expressed genes based on Gene Ontology 5](#_Toc1848888)

[References 6](#_Toc1848889)

# Extraction of the total RNA

Total RNA was extracted using the RNeasy Plant Mini Kit (Qiagen, Germany) combined with the RNase-Free DNase set (Qiagen, Germany). RNA degradation and contamination was monitored on 1% agarose gels. RNA purity was checked using the NanoPhotometer spectrophotometer (IMPLEN, CA, USA). RNA concentration was measured using Qubit RNA Assay Kit in Qubit 2.0 Flurometer (Life Technologies, CA, USA). RNA integrity was assessed using the RNA Nano 6000 Assay Kit of the Bioanalyzer 2100 system (Agilent Technologies, CA, USA).

# cDNA synthesis and Library preparation

Three to seven cultivations were performed for each condition. Mycelial samples were pooled together and divided in three technical repeats further used for the extraction of RNA. A total amount of 3 μg RNA per each sample was used as input material for the RNA sample preparations. Sequencing libraries were generated using NEBNext® UltraTM RNA Library Prep Kit for Illumina® (NEB, USA) following manufacturer’s recommendations and index codes were added to attribute sequences to each sample. Briefly, mRNA was purified from total RNA using poly-T oligo-attached magnetic beads. Fragmentation was carried out using divalent cations under elevated temperature in NEBNext First Strand Synthesis Reaction Buffer(5X). First strand cDNA was synthesized using random hexamer primer and M-MuLV Reverse Transcriptase (RNase H-). Second strand cDNA synthesis was subsequently performed using DNA Polymerase I and RNase H. Remaining overhangs were converted into blunt ends via exonuclease/polymerase activities. After adenylation of 3’ ends of DNA fragments, NEBNext Adaptor with hairpin loop structure were ligated to prepare for hybridization. In order to select cDNA fragments of preferentially 250~300 bp in length, the library fragments were purified with AMPure XP system (Beckman Coulter, Leading Edge Genomic Services & Solutions Beverly, USA). Then 3 μl USER Enzyme (NEB, USA) was used with size-selected, adaptor-ligated cDNA at 37°C for 15 min followed by 5 min at 95 °C before PCR. Then PCR was performed with Phusion High-Fidelity DNA polymerase, Universal PCR primers and Index (X) Primer. At last, PCR products were purified (AMPure XP system) and library quality was assessed on the Agilent Bioanalyzer 2100 system.

# Deep sequencing

The clustering of the index-coded samples was performed on a cBot Cluster Generation System using TruSeq PE Cluster Kit v3-cBot-HS (Illumia) according to the manufacturer’s instructions. After cluster generation, the library preparations were sequenced on an Illumina platform and a total of 330.5 x 10^6^ 125 bp/150 bp paired-end reads were generated.

# Data analysis

Transcriptome sequences were obtained from Illumina NextSeq 500 System (Microsynth, Switzerland, for Tgui, Foc4 and *∆nox1*) or Illumina HiSeq™ PE150 for *nox1OE* (Novogene, China), respectively. The raw sequences generated were quality checked using FastQC-VO.8.0 (http:// http://www.bioinformatics.babraham.ac.uk/projects/fastqc/). All the high quality sequences were aligned against the reference genome with the spliced aligner Bowtie integrated in TopHat2 (Kim *et al.*, 2013). With a threshold of 2 base-mismatches, all samples have a reads-matching rate of > 75%. For the mixed samples (After contact), which include read sequences from both Tgui and Foc4, any reads mapped to each genome were considered belonging to itself because of their far evolutionary distance. Reads mapped to the genome were used to calculate every gene’s expression in each sample. We used RPKM (Reads Per Kb per Million reads) to estimate expressions of genes so as to normalize the effects of different gene lengths and different total mapped reads among samples. Differentially expressed genes (DEGs) were detected using the method described by Chen et al. (Chen *et al.*, 2016). This method is based on the Poisson distribution and normalization for differences in RNA output sizes and sequencing depths between samples. P-value was used to test the statistical significance and FDR (false discovery rate) to determine the threshold of P-value in multiple tests. Expression differences characterized by |log_2_ of foldchanges| >= 2, a p-value ≤ 0.0001 and RPKM values > 2.0 were considered significant.

We used a manually curated annotation for identifying the potential identity or function of all significantly regulated genes in the Tgui and the Foc4, which was performed as follows: first, all genes were subjected to conserved domain search (https://www.ncbi.nlm.nih.gov/Structure/bwrpsb/bwrpsb.cgi), applying a shortcut of <E-20. Genes with E higher than that or encoding proteins with no identified function, lacking a conserved domain or possessing a domain with unknown function were considered “unknown proteins”. Genes which were present only in *Trichoderma* or *Fusarium,* respectively, but did not occur (Blastp >E-30) in any other fungal genus were termed orphans. Genes with putative functional domains were then subjected to NCBI blastp for fine analysis. If the gene had already been characterized in another species and had a negative probability of <E-75, this name was used in the annotation. Identification of carbohydrate active enzymes (CAZys) and proteolytic enzymes was performed by use of the respective databases (Lombard *et al.*, 2014; Rawlings *et al.*, 2016). SMURF (Khaldi *et al.*, 2010) was used to predict secondary metabolite clusters in NJAU 4742. For Foc4, the cluster predicted by Guo *et al*. (2014) were used.

Functional enrichment analysis of differentially expressed based on Gene Ontology (GO) terms was performed using the R package GO_MWU (<https://github.com/z0on/GO_MWU>) using the whole protein set as background for Fisher exact test and p-value as a measure of significance. This method corrects the test p-values using the Benjamini-Hochberg false discovery rate procedure.

# Functional enrichment analysis of differentially expressed genes based on Gene Ontology


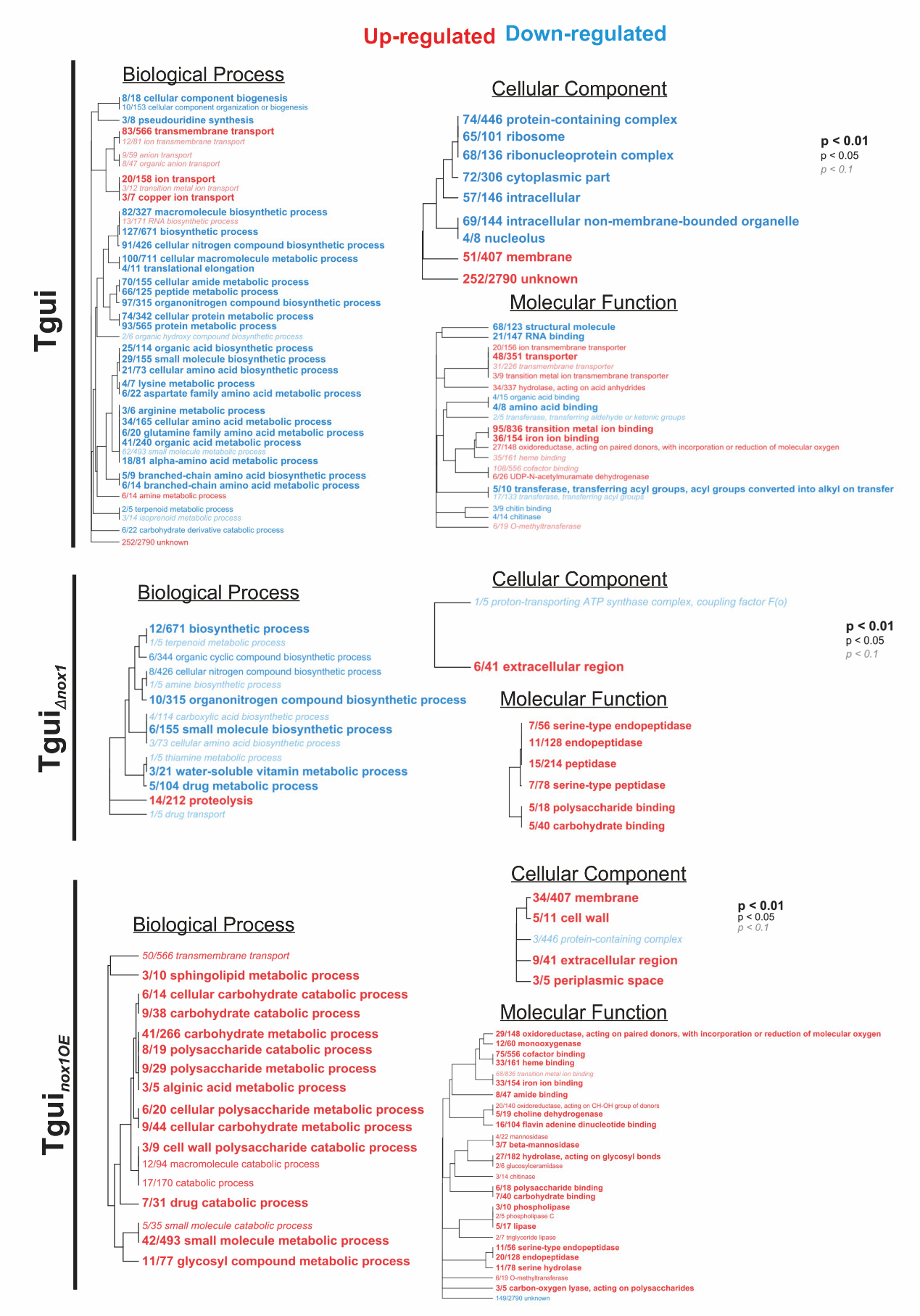


**Figure S8 1:** Enrichment analysis of GO terms (Biological process, Molecular Function and Cellular Component) of differentially expressed genes. Each plot shows a hierarchical clustering tree of significant GO categories based on shared genes. Category names are plotted in different colors and fonts. Fonts indicate the level of statistical significance, and colors indicate enrichment of GO categories with either up- (red) or down- (blue) regulated genes. The category names are preceded by the fraction indicating the number of differentially expressed genes relative to the total number of genes belonging to this category. The legend provides the correspondence of the fonts to significance thresholds. The method corrects the p-values using Benjamini-Hochberg false discovery rate procedure.

# References

Chen, H.I.H., Jin, Y.F., Huang, Y.F., and Chen, Y.D. (2016) Detection of high variability in gene expression from single-cell RNA-seq profiling. *Bmc Genomics* **17**: 508.

Guo, L.J., Han, L.J., Yang, L.Y., Zeng, H.C., Fan, D.D., Zhu, Y.B. et al. (2014) Genome and Transcriptome Analysis of the Fungal Pathogen *Fusarium oxysporum* f. sp *cubense* Causing Banana Vascular Wilt Disease. *Plos One* **10**: e0117621.

Khaldi, N., Seifuddin, F.T., Turner, G., Haft, D., Nierman, W.C., Wolfe, K.H., and Fedorova, N.D. (2010) SMURF: Genomic mapping of fungal secondary metabolite clusters. *Fungal Genetics and Biology* **47**: 736-741.

Kim, D., Pertea, G., Trapnell, C., Pimentel, H., Kelley, R., and Salzberg, S.L. (2013) TopHat2: accurate alignment of transcriptomes in the presence of insertions, deletions and gene fusions. *Genome Biology* **14**: R36.

Lombard, V., Ramulu, H.G., Drula, E., Coutinho, P.M., and Henrissat, B. (2014) The carbohydrate-active enzymes database (CAZy) in 2013. *Nucleic Acids Research* **42**: D490-D495.

Rawlings, N.D., Barrett, A.J., and Finn, R. (2016) Twenty years of the MEROPS database of proteolytic enzymes, their substrates and inhibitors. *Nucleic Acids Research* **44**: D343-D350.
