# Supplementary material for: Guttation capsules containing hydrogen peroxide: an evolutionarily conserved NADPH oxidase gains a role in wars between related fungi
Source: Environ Microbiol. 2019 Apr 22;21(8):2644–58. doi: 10.1111/1462-2920.14575 (PMC6850483; doi:10.1111/1462-2920.14575)
Supplement: Supplementary file 10 — Supporting Information S10. Annotation of the genome of T. guizhouense NJAU 4742 and manual annotation of differentially expressed genes of Fusarium oxysporum f. sp. cubense 4 (Foc4) [file EMI-21-2644-s010.pdf]

**Complete manual annotation of the genome of *T. guizhouense* NJAU 4742**

| NCBI assession | PI | Annotation |
|---|---|---|
| OPB44706 | T4742_S00013.10 | (chloro)peroxidase |
| OPB45937 | T4742_S00008.3 | 1,2-dihydroxy-3-keto-5-methylthiopentene dioxygenase , putative |
| OPB39008 | T4742_S00006.67 | 14-3-3 protein |
| OPB42058 | T4742_S00004.160 | 19S regulatory particle ATPase Rpt1 |
| OPB44918 | T4742_S00013.223 | 19S regulatory particle ATPase Rpt2 |
| OPB36749 | T4742_S00007.467 | 19S regulatory particle ATPase Rpt3 |
| OPB36995 | T4742_S00005.22 | 19S regulatory particle ATPase Rpt4 |
| OPB40429 | T4742_S00001.508 | 19S regulatory particle ATPase Rpt5 |
| OPB39683 | T4742_S00015.27 | 19S regulatory particle ATPase Rpt6 |
| OPB39554 | T4742_S00006.614 | 1-acyl-sn-glycerol-3-phosphate acyltransferase |
| OPB43035 | T4742_S00012.272 | 1-acyl-sn-gylcerol-3-phosphate acyltransferase (Lysophosphatidic acid acyltransferase) |
| OPB41341 | T4742_S00011.182 | 1-alkyl-2-acetylglycerophosphocholine esterase |
| OPB46055 | T4742_S00008.121 | 1-aminocyclopropane-1-carboxylate oxidase |
| OPB38941 | T4742_S00002.990 | 1-aminocyclopropane-1-carboxylate synthase |
| OPB41325 | T4742_S00011.166 | 1-aminocyclopropane-1-carboxylate synthase |
| OPB44802 | T4742_S00013.107 | 1-aminocyclopropane-1-carboxylate synthase |
| OPB36576 | T4742_S00007.293 | 2,2-dialkylglycine decarboxylase, AIB forming ? |
| OPB46618 | T4742_S00009.60 | 2,3-diketo-5-methylthio-1-phosphopentane phosphatase |
| OPB39221 | T4742_S00006.281 | 2,4-dienoyl-CoA-reductase |
| OPB43525 | T4742_S00016.45 | 2,4-dihydroxyhept-2-ene-1,7-dioic acid aldolase |
| OPB36685 | T4742_S00007.403 | 2,5-didehydrogluconate reductase |
| OPB37408 | T4742_S00005.435 | 2,6-dihydropseudooxynicotine hydrolase |
| OPB38431 | T4742_S00002.479 | 20S proteasome alpha subunit Pup2 |
| OPB39477 | T4742_S00006.537 | 20S proteasome alpha subunit Scl1 |
| OPB42052 | T4742_S00004.154 | 20S proteasome alpha-type subunit Pre10 |
| OPB37308 | T4742_S00005.335 | 20S proteasome alpha-type subunit Pre5 |
| OPB40175 | T4742_S00001.254 | 20S proteasome beta-type subunit Pre1 |
| OPB38969 | T4742_S00006.28 | 20S proteasome beta-type subunit Pre2 |
| OPB41411 | T4742_S00011.253 | 20S proteasome beta-type subunit Pre3 |
| OPB36997 | T4742_S00005.24 | 20S proteasome beta-type subunit Pre4 |
| OPB43711 | T4742_S00016.231 | 20S proteasome beta-type subunit Pre7 |
| OPB46255 | T4742_S00008.322 | 20S proteasome beta-type subunit Pre8 |
| OPB42908 | T4742_S00012.145 | 20S proteasome beta-type subunit Pup1 |
| OPB39455 | T4742_S00006.515 | 20S proteasome beta-type subunit Pup3 |

| | | |
|---|---|---|
| OPB40605 | T4742_S00001.685 | 20S proteasome, A and B subunits |
| OPB39330 | T4742_S00006.390 | 20S proteasome, alpha subunit Pre6 |
| OPB46761 | T4742_S00009.203 | 26S proteasome non-ATPase regulatory subunit 9, putative |
| OPB38275 | T4742_S00002.322 | 26S proteasome regulatory complex subunit Rpn1 |
| OPB41926 | T4742_S00004.28 | 26S proteasome regulatory complex subunit Rpn10 |
| OPB37769 | T4742_S00017.36 | 26S proteasome regulatory complex subunit Rpn11 |
| OPB37750 | T4742_S00017.17 | 26S proteasome regulatory complex subunit Rpn12 |
| OPB44667 | T4742_S00003.939 | 26S proteasome regulatory complex subunit Rpn2 |
| OPB36741 | T4742_S00007.459 | 26S proteasome regulatory complex subunit Rpn3 |
| OPB46578 | T4742_S00009.18 | 26S proteasome regulatory complex subunit Rpn4 |
| OPB45284 | T4742_S00010.195 | 26S proteasome regulatory complex subunit Rpn5 |
| OPB42458 | T4742_S00004.560 | 26S proteasome regulatory complex subunit Rpn6 |
| OPB36401 | T4742_S00007.118 | 26S proteasome regulatory complex subunit Rpn7 |
| OPB43511 | T4742_S00016.31 | 26S proteasome regulatory complex subunit Rpn8 |
| OPB36971 | T4742_S00007.689 | 26S proteasome regulatory complex subunit Rpn9 |
| OPB42351 | T4742_S00004.453 | 2-dehydropantoate 2-reductase family protein |
| OPB40715 | T4742_S00001.795 | 2-dehydropantoate-2-reductase |
| OPB38732 | T4742_S00002.781 | 2-deoxy-D-gluconate 3-dehydrogenase |
| OPB45900 | T4742_S00014.236 | 2-deoxyglucose-6-phosphate phosphatase |
| OPB35993 | T4742_S00023.28 | 2-hydroxyacid dehydrogenase |
| OPB40412 | T4742_S00001.491 | 2-keto-3-deoxy-l-rhamnonate aldolase [Fusarium langsethiae] |
| OPB40671 | T4742_S00001.751 | 2-keto-3-deoxy-L-rhamnonate aldolase [Valsa mali var. pyri] |
| OPB41180 | T4742_S00011.20 | 2-keto-4-pentenoate hydratase/2-oxohepta-3-ene-1,7-dioic acid hydratase [Aspergillus oryzae 3.042] |
| OPB40631 | T4742_S00001.711 | 2-ketoglutarate-FeII-dioxigenase |
| OPB41895 | T4742_S00018.218 | 2-ketoglutarate-FeII-dioxigenase |
| OPB43393 | T4742_S00019.126 | 2-ketoglutarate-FeII-dioxigenase |
| OPB43399 | T4742_S00019.132 | 2-ketoglutarate-FeII-dioxigenase |
| OPB45628 | T4742_S00010.539 | 2-ketoglutarate-FeII-dioxigenase |
| OPB41355 | T4742_S00011.196 | 2-methylcitrate dehydratase PrpD [Xylona heveae TC161] |
| OPB38600 | T4742_S00002.649 | 2-methylcitrate dehydratase-like protein |
| OPB41276 | T4742_S00011.117 | 2-methylcitrate synthase |
| OPB36410 | T4742_S00007.127 | 2-nitropropane dioxygenase |
| OPB37578 | T4742_S00005.605 | 2-nitropropane dioxygenase |
| OPB43453 | T4742_S00019.186 | 2OG-Fe(II) oxygenase [Clohesyomyces aquaticus] |
| OPB38880 | T4742_S00002.929 | 2OG-Fe(II) oxygenase superfamily protein |
| OPB37262 | T4742_S00005.289 | 2OG-Fe(II) oxygenase superfamily protein |

| | | |
|---|---|---|
| OPB44329 | T4742_S00003.600 | 2OG-Fe(II) oxygenase superfamily protein |
| OPB46437 | T4742_S00008.504 | 2OG-Fe(II) oxygenase superfamily protein |
| OPB37742 | T4742_S00017.9 | 2-oxoglutarate dehydrogenase component E2 |
| OPB41661 | T4742_S00011.504 | 2-oxoglutarate dehydrogenase-like protein |
| OPB42620 | T4742_S00004.724 | 2-oxoglutarate-dependent ethylene/succinate-forming enzyme, putative |
| OPB36357 | T4742_S00007.74 | 2-oxoisovalerate dehydrogenase subunit beta, putative |
| OPB43566 | T4742_S00016.86 | 3' exoribonuclease |
| OPB45195 | T4742_S00010.103 | 3' exoribonuclease |
| OPB42604 | T4742_S00004.708 | 3' exoribonuclease family protein |
| OPB39916 | T4742_S00015.260 | 3' exoribonuclease involved in RNA processing during translation. |
| OPB40160 | T4742_S00001.239 | 3'-5' exonuclease |
| OPB41780 | T4742_S00018.103 | 3'-5' exonuclease |
| OPB42057 | T4742_S00004.159 | 3'-5' exonuclease |
| OPB45979 | T4742_S00008.45 | 3'-5' exonuclease |
| OPB42150 | T4742_S00004.252 | 39S ribosomal protein L53/MRP-L53, putative |
| OPB39216 | T4742_S00006.276 | 3-beta hydroxysteroid dehydrogenase/isomerase |
| OPB41219 | T4742_S00011.59 | 3-beta hydroxysteroid dehydrogenase/isomerase |
| OPB45389 | T4742_S00010.300 | 3-beta hydroxysteroid dehydrogenase/isomerase, putative |
| OPB41892 | T4742_S00018.215 | 3-carboxymuconate cyclase |
| OPB47038 | T4742_S00009.480 | 3-dehydroshikimate dehydratase |
| OPB42868 | T4742_S00012.105 | 3-dehydroshikimate dehydratase |
| OPB41477 | T4742_S00011.320 | 3-dehydroshikimate dehydratase [Alternaria alternata] |
| OPB37889 | T4742_S00017.156 | 3-deoxy-7-phosphoheptulonate synthase |
| OPB43295 | T4742_S00019.28 | 3-hydroxi-isobutyrate dehydrogenase |
| OPB37719 | T4742_S00005.747 | 3-hydroxyacyl-CoA dehydrogenase |
| OPB40166 | T4742_S00001.245 | 3-hydroxyacyl-CoA dehydrogenase |
| OPB37720 | T4742_S00005.748 | 3-hydroxyacyl-CoA dehydrogenase [Trichoderma guizhouense] |
| OPB37646 | T4742_S00005.673 | 3-hydroxyacyl-CoA dehydrogenase, NAD binding domain, putative |
| OPB42751 | T4742_S00004.857 | 3-hydroxyacyl-CoA dehyrogenase |
| OPB39003 | T4742_S00006.62 | 3-hydroxyisobutyrate dehydrogenase |
| OPB38082 | T4742_S00002.129 | 3-isopropylmalate dehydratase |
| OPB43056 | T4742_S00012.293 | 3-isopropylmalate dehydrogenase |
| OPB45831 | T4742_S00014.166 | 3-isopropylmalate dehydrogenase |
| OPB42743 | T4742_S00004.849 | 3-ketoacyl-acyl carrier protein reductase |
| OPB43591 | T4742_S00016.111 | 3-ketoacyl-CoA thiolase |
| OPB46954 | T4742_S00009.396 | 3-ketoacyl-CoA thiolase |

| | | |
|---|---|---|
| OPB36598 | T4742_S00007.316 | 3-ketoacyl-CoA thiolase |
| OPB36599 | T4742_S00007.317 | 3-ketoacyl-CoA thiolase |
| OPB41976 | T4742_S00004.78 | 3-methyl-2-oxobutanoate hydroxymethyltransferase |
| OPB35939 | T4742_S00025.12 | 3-Methylcrotonyl-CoA carboxylase, non-biotin containing subunit/Acetyl-CoA carboxylase carboxyl transferase, subunit beta |
| OPB45541 | T4742_S00010.452 | 3-octaprenyl-4-hydroxybenzoate carboxy-lyase |
| OPB39289 | T4742_S00006.349 | 3-oxo-5-alpha-steroid 4-dehydrogenase |
| OPB39380 | T4742_S00006.440 | 3-oxo-5-alpha-steroid 4-dehydrogenase |
| OPB45746 | T4742_S00014.81 | 3-oxo-5-alpha-steroid 4-dehydrogenase |
| OPB39398 | T4742_S00006.458 | 3-oxoacyl-(acyl carrier protein) synthase |
| OPB43446 | T4742_S00019.179 | 3-oxoacyl-[acyl-carrier protein] reductase |
| OPB43247 | T4742_S00022.50 | 3-oxoacyl-[acyl-carrier-protein] FabG [Phialophora attae] |
| OPB44591 | T4742_S00003.863 | 3-oxoadipate enol-lactonase 2 [ |
| OPB46409 | T4742_S00008.476 | 3'-tRNA processing endoribonuclease |
| OPB43690 | T4742_S00016.210 | 4,5-dihydroxyphthalate decarboxylase |
| OPB42810 | T4742_S00012.47 | 4,5-dioxygenase (only in Amanita muscaria) |
| OPB40868 | T4742_S00001.949 | 40s mitochodrial ribosome subunit protein Mrp17 |
| OPB40869 | T4742_S00001.950 | 40s mitochodrial ribosome subunit protein Mrp17 |
| OPB46094 | T4742_S00008.161 | 40s ribosomal protein |
| OPB37138 | T4742_S00005.165 | 40s ribosomal protein L44e by homology to the corresponding protein of several yeasts and fungi. Homologue of yeast RPL42a/b. |
| OPB46691 | T4742_S00009.133 | 40s ribosomal protein S14 (S11 family). |
| OPB39207 | T4742_S00006.267 | 40s ribosomal protein S15 based on homology to corresponding proteins in other fungi. |
| OPB46741 | T4742_S00009.183 | 40s ribosomal protein S19e by homology to the corresponding proteins of G. zeae and E. nidulans. |
| OPB37791 | T4742_S00017.58 | 40s ribosomal protein S21 |
| OPB42340 | T4742_S00004.442 | 40s ribosomal protein S22 (S8 family) by homology to the corresponding protein of N. crassa. Homologue of yeast RPS22a/b. |
| OPB43168 | T4742_S00012.405 | 40s ribosomal protein S25 |
| OPB36495 | T4742_S00007.212 | 40s ribosomal protein S26E (CRP5) (13.6 kDa ribosomal protein). |
| OPB39285 | T4742_S00006.345 | 40s ribosomal protein S6 |
| OPB37206 | T4742_S00005.233 | 4-aminobutyrate aminotransferase |
| OPB44673 | T4742_S00003.945 | 4-coumarate:coenzyme A ligase |
| OPB41540 | T4742_S00011.383 | 4-coumarate-CoA ligase 1 [Trichoderma harzianum] |
| OPB36252 | T4742_S00021.170 | 4-hydroxy-2-oxo-heptane-1,7-dioate aldolase [Tolypocladium ophioglossoides CBS 100239] |
| OPB43190 | T4742_S00012.427 | 4-hydroxyphenylpyruvate dioxygenase |
| OPB39425 | T4742_S00006.485 | 4-nitrophenylphosphatase |
| OPB40297 | T4742_S00001.376 | 4-oxalocrotonate tautomerase |
| OPB41250 | T4742_S00011.91 | 4-trimethylaminobutyraldehyde dehydrogenase |
| OPB44887 | T4742_S00013.192 | 5' nucleotidase |

| | | |
|---|---|---|
| OPB38277 | T4742_S00002.324 | 5-formyltetrahydrofolate cyclo-ligase |
| OPB40419 | T4742_S00001.498 | 5'-nucleotidase |
| OPB39904 | T4742_S00015.248 | 5-phospho-ribosyl-1(alpha)-pyrophosphate synthetase |
| OPB43715 | T4742_S00016.235 | 6,7-dimethyl-8-ribityllumazine synthase |
| OPB43948 | T4742_S00003.219 | 60s acid ribosomal protein P1 based on homology to corresponding proteins in fungi and yeasts. |
| OPB37496 | T4742_S00005.523 | 60S ribosomal protein L1 |
| OPB42214 | T4742_S00004.316 | 60s ribosomal protein L1. |
| OPB45081 | T4742_S00013.386 | 60S ribosomal protein L12 (L11 family). |
| OPB36617 | T4742_S00007.335 | 60S ribosomal protein L13 |
| OPB44829 | T4742_S00013.134 | 60S ribosomal protein L16 (L13 family). |
| OPB45607 | T4742_S00010.518 | 60S ribosomal protein L19 [Aschersonia aleyrodis RCEF 2490] |
| OPB38499 | T4742_S00002.548 | 60S ribosomal protein L24 |
| OPB46566 | T4742_S00009.6 | 60s ribosomal protein L29 based on homology to the L29 protein of yeast. |
| OPB46822 | T4742_S00009.264 | 60S ribosomal protein L35 (L29 family) by homology with the corresponding protein in other fungi. |
| OPB39457 | T4742_S00006.517 | 60s ribosomal protein L36 based on homology to the corresponding protein in other fungi. |
| OPB37251 | T4742_S00005.278 | 60S ribosomal protein L38 by homology with corresponding proteins in other fungi and yeasts. |
| OPB41444 | T4742_S00011.287 | 60s ribosomal protein rla2 |
| OPB39581 | T4742_S00006.641 | 60S ribosomal protein RML2, mitochondrial precursor from Ashbya gossypii. |
| OPB40953 | T4742_S00001.1034 | 60S ribosomal subunit (protein L34e). |
| OPB41452 | T4742_S00011.295 | 60S ribosome biogenesis protein Mak11, putative |
| OPB39885 | T4742_S00015.229 | 64 kDa mitochondrial NADH dehydrogenase, putative |
| OPB39758 | T4742_S00015.102 | 6-phosphofructo-2-kinase |
| OPB45222 | T4742_S00010.131 | 6-phosphofructokinase |
| OPB36039 | T4742_S00023.74 | 6-phosphogluconate dehydrogenase, decarboxylating |
| OPB42869 | T4742_S00012.106 | 6-phosphogluconate dehydrogenase, decarboxylating |
| OPB42277 | T4742_S00004.379 | 6-phosphogluconolactonase |
| OPB43837 | T4742_S00003.108 | 6-phosphogluconolactonase, putative (check!) |
| OPB40149 | T4742_S00001.228 | 75k gamma secalin [Colletotrichum gloeosporioides Nara gc5] |
| OPB40680 | T4742_S00001.760 | A/B hydrolase |
| OPB42350 | T4742_S00004.452 | A/B hydrolase |
| OPB42739 | T4742_S00004.845 | A/B hydrolase |
| OPB42821 | T4742_S00012.58 | A/B hydrolase |
| OPB43463 | T4742_S00019.196 | A/B hydrolase |
| OPB44474 | T4742_S00003.746 | A/B hydrolase |
| OPB44494 | T4742_S00003.766 | A/B hydrolase |
| OPB44780 | T4742_S00013.85 | A/B hydrolase |

| | | |
|---|---|---|
| OPB45476 | T4742_S00010.387 | A/B hydrolase |
| OPB45673 | T4742_S00014.7 | A/B hydrolase |
| OPB45888 | T4742_S00014.223 | A/B hydrolase |
| OPB45927 | T4742_S00014.264 | A/B hydrolase |
| OPB46001 | T4742_S00008.67 | A/B hydrolase |
| OPB46661 | T4742_S00009.103 | A/B hydrolase |
| OPB38378 | T4742_S00002.426 | A/B hydrolase lipase |
| OPB36263 | T4742_S00021.182 | A/B-hydrolase |
| OPB36431 | T4742_S00007.148 | A/B-hydrolase |
| OPB38826 | T4742_S00002.875 | A/B-hydrolase |
| OPB39185 | T4742_S00006.245 | A/B-hydrolase |
| OPB40301 | T4742_S00001.380 | A/B-hydrolase |
| OPB40512 | T4742_S00001.591 | A/B-hydrolase |
| OPB40639 | T4742_S00001.719 | A/B-hydrolase |
| OPB40802 | T4742_S00001.883 | A/B-hydrolase |
| OPB41127 | T4742_S00020.151 | A/B-hydrolase |
| OPB41158 | T4742_S00020.182 | A/B-hydrolase |
| OPB41161 | T4742_S00020.185 | A/B-hydrolase |
| OPB41213 | T4742_S00011.53 | A/B-hydrolase |
| OPB41229 | T4742_S00011.70 | A/B-hydrolase |
| OPB41712 | T4742_S00018.35 | A/B-hydrolase |
| OPB42695 | T4742_S00004.801 | A/B-hydrolase |
| OPB42724 | T4742_S00004.830 | A/B-hydrolase |
| OPB43239 | T4742_S00022.42 | A/B-hydrolase |
| OPB43414 | T4742_S00019.147 | A/B-hydrolase |
| OPB43523 | T4742_S00016.43 | A/B-hydrolase |
| OPB43534 | T4742_S00016.54 | A/B-hydrolase |
| OPB43760 | T4742_S00003.31 | A/B-hydrolase |
| OPB43814 | T4742_S00003.85 | A/B-hydrolase |
| OPB44046 | T4742_S00003.317 | A/B-hydrolase |
| OPB44212 | T4742_S00003.483 | A/B-hydrolase |
| OPB44522 | T4742_S00003.794 | A/B-hydrolase |
| OPB44596 | T4742_S00003.868 | A/B-hydrolase |
| OPB44619 | T4742_S00003.891 | A/B-hydrolase |
| OPB44708 | T4742_S00013.12 | A/B-hydrolase |
| OPB44719 | T4742_S00013.23 | A/B-hydrolase |

| | | |
|---|---|---|
| OPB45439 | T4742_S00010.350 | A/B-hydrolase |
| OPB45745 | T4742_S00014.80 | A/B-hydrolase |
| OPB45855 | T4742_S00014.190 | A/B-hydrolase |
| OPB46044 | T4742_S00008.110 | A/B-hydrolase |
| OPB47154 | T4742_S00009.596 | A/B-hydrolase |
| OPB37329 | T4742_S00005.356 | Aa_transTransmembrane amino acid transporter protein |
| OPB45579 | T4742_S00010.490 | Aa_transTransmembrane amino acid transporter protein |
| OPB46620 | T4742_S00009.62 | Aa_transTransmembrane amino acid transporter protein |
| OPB40308 | T4742_S00001.387 | AA1 Auxiliary Activity Family 1 |
| OPB38520 | T4742_S00002.569 | AA1 Auxiliary Activity Family 1 multicopper oxidase |
| OPB42865 | T4742_S00012.102 | AA1 Auxiliary Activity Family 1 multicopper oxidase |
| OPB45593 | T4742_S00010.504 | AA1 Auxiliary Activity Family 1, multicopper oxidase |
| OPB42382 | T4742_S00004.484 | AA1_2 Ferroxidase |
| OPB45341 | T4742_S00010.252 | AA1_3 Laccase-like multicopper oxidase |
| OPB41940 | T4742_S00004.42 | AA12 |
| OPB36086 | T4742_S00021.2 | AA3_2 GMC oxidoreductase |
| OPB37177 | T4742_S00005.204 | AA3_2 GMC oxidoreductase |
| OPB40614 | T4742_S00001.694 | AA3_2 GMC oxidoreductase |
| OPB41039 | T4742_S00020.63 | AA3_2 GMC oxidoreductase |
| OPB44840 | T4742_S00013.145 | AA3_2 GMC oxidoreductase |
| OPB41235 | T4742_S00011.76 | AA3_3 Alcohol oxidase |
| OPB45591 | T4742_S00010.502 | AA3_3 Alcohol oxidase |
| OPB41190 | T4742_S00011.30 | AA9 cellulose-monooxygenase, with CBM1 domain |
| OPB36083 | T4742_S00024.44 | AAA ATPase |
| OPB36371 | T4742_S00007.88 | AAA ATPase |
| OPB36961 | T4742_S00007.679 | AAA ATPase |
| OPB37224 | T4742_S00005.251 | AAA ATPase |
| OPB37961 | T4742_S00002.8 | AAA ATPase |
| OPB38042 | T4742_S00002.89 | AAA ATPase |
| OPB38662 | T4742_S00002.711 | AAA ATPase |
| OPB39600 | T4742_S00006.660 | AAA ATPase |
| OPB41538 | T4742_S00011.381 | AAA ATPase |
| OPB42085 | T4742_S00004.187 | AAA ATPase |
| OPB43563 | T4742_S00016.83 | AAA ATPase |
| OPB46152 | T4742_S00008.219 | AAA ATPase |
| OPB41404 | T4742_S00011.246 | AAA ATPase [Alternaria alternata] |

| | | |
|---|---|---|
| OPB41385 | T4742_S00011.226 | AAA ATPase [Trichoderma parareesei] |
| OPB40461 | T4742_S00001.540 | AAA ATPase, central region |
| OPB40861 | T4742_S00001.942 | AAA ATPase, core |
| OPB41678 | T4742_S00011.521 | AAA ATPase, core |
| OPB42872 | T4742_S00012.109 | AAA ATPase, DNA repair and recombination protein PIF1 (ATP dependent helicase) |
| OPB46815 | T4742_S00009.257 | AAA ATPase/HSP104 |
| OPB39338 | T4742_S00006.398 | aaa family ATPase |
| OPB46587 | T4742_S00009.27 | AAA family ATPase |
| OPB47125 | T4742_S00009.567 | AAA family ATPase |
| OPB41155 | T4742_S00020.179 | AAA family ATPase [Lentinula edodes] |
| OPB42391 | T4742_S00004.493 | AAA family ATPase Pontin, putative |
| OPB42419 | T4742_S00004.521 | AAA family ATPase Rvb2/Reptin, putative |
| OPB46311 | T4742_S00008.378 | AAA+-ATPase |
| OPB46886 | T4742_S00009.328 | AAA+Type ATPase |
| OPB41386 | T4742_S00011.227 | AAA+-type ATPase |
| OPB42673 | T4742_S00004.778 | AAA+-type ATPase |
| OPB44264 | T4742_S00003.535 | AAA+-type ATPase |
| OPB44598 | T4742_S00003.870 | AAA+-type ATPase |
| OPB44601 | T4742_S00003.873 | AAA+-type ATPase |
| OPB47111 | T4742_S00009.553 | AAA+-type ATPase |
| OPB45161 | T4742_S00010.68 | AAA-ATPase Cdc48 |
| OPB36572 | T4742_S00007.289 | AATF-like transcription factor Bfr2 |
| OPB37686 | T4742_S00005.713 | AB hydrolase |
| OPB40753 | T4742_S00001.834 | AB hydrolase |
| OPB43854 | T4742_S00003.125 | AB hydrolase |
| OPB46439 | T4742_S00008.506 | AB hydrolase |
| OPB41323 | T4742_S00011.164 | ABC metal resistance protein YCF1 |
| OPB46351 | T4742_S00008.418 | ABC multidrug resistance protein |
| OPB36051 | T4742_S00024.12 | ABC multidrug transporter |
| OPB43681 | T4742_S00016.201 | ABC multidrug transporter |
| OPB46124 | T4742_S00008.191 | ABC multidrug transporter |
| OPB46345 | T4742_S00008.412 | ABC multidrug transporter |
| OPB38116 | T4742_S00002.163 | ABC transporter |
| OPB40682 | T4742_S00001.762 | ABC transporter |
| OPB42763 | T4742_S00004.869 | ABC transporter |
| OPB44267 | T4742_S00003.538 | ABC transporter |

| | | |
|---|---|---|
| OPB45894 | T4742_S00014.230 | ABC transporter |
| OPB38373 | T4742_S00002.421 | ABC transporter |
| OPB44905 | T4742_S00013.210 | ABC transporter |
| OPB45648 | T4742_S00010.559 | ABC transporter |
| OPB40064 | T4742_S00001.143 | ABC transporter superfamily?! |
| OPB43731 | T4742_S00003.2 | ABC transporter,multidrug resistance associated protein |
| OPB46524 | T4742_S00008.591 | ABC transporter,multidrug resistance associated protein |
| OPB45512 | T4742_S00010.423 | ABC transporter/ATPase-like protein |
| OPB40683 | T4742_S00001.763 | ABC transporter/spermidine/putrescine import ATP-binding protein |
| OPB42982 | T4742_S00012.219 | ABC transporter/translation initiation factor RLI1 |
| OPB38902 | T4742_S00002.951 | ABC transporters, PDR type |
| OPB37096 | T4742_S00005.123 | ABC1 family protein |
| OPB36567 | T4742_S00007.284 | ABC1 family protein |
| OPB44685 | T4742_S00003.957 | ABC-transporter Ste6p |
| OPB46051 | T4742_S00008.117 | ABC-type Fe3+ transport system [Penicillium digitatum Pd1] |
| OPB39562 | T4742_S00006.622 | abhydrolase domain-containing protein |
| OPB41222 | T4742_S00011.63 | AB-hydrolase YheT [Aureobasidium pullulans EXF-150] |
| OPB43740 | T4742_S00003.11 | AC transposase |
| OPB41314 | T4742_S00011.155 | ACC deaminase |
| OPB37548 | T4742_S00005.575 | acetamidase |
| OPB42732 | T4742_S00004.838 | acetamidase |
| OPB44963 | T4742_S00013.268 | acetamidase |
| OPB39574 | T4742_S00006.634 | Acetate kinase |
| OPB39629 | T4742_S00006.689 | acetate non-utilizing protein 9, putative |
| OPB44235 | T4742_S00003.506 | Acetate transporter required for normal sporulation; phosphorylated in mitochondria |
| OPB40264 | T4742_S00001.343 | acetate—CoA ligase |
| OPB41081 | T4742_S00020.105 | acetoacetyl-CoA synthase |
| OPB42573 | T4742_S00004.676 | acetolactate synthase |
| OPB41803 | T4742_S00018.126 | Acetolactate synthase [Cercospora beticola] |
| OPB42482 | T4742_S00004.584 | Acetolactate synthase small (regulatory) subunit IlvH |
| OPB41579 | T4742_S00011.422 | Acetyl/propionyl-CoA carboxylase alpha subunit |
| OPB36347 | T4742_S00007.64 | acetylcholinesterase |
| OPB46032 | T4742_S00008.98 | acetylcholinesterase |
| OPB44202 | T4742_S00003.473 | acetyl-CoA acetyltransferase |
| OPB42858 | T4742_S00012.95 | acetyl-CoA carboxylase |
| OPB42464 | T4742_S00004.566 | acetyl-CoA synthase |

| | | |
|---|---|---|
| OPB44012 | T4742_S00003.283 | acetylglutamate kinase ARG6 |
| OPB45683 | T4742_S00014.17 | acetylornithine aminotransferase-like protein |
| OPB37659 | T4742_S00005.686 | acetylornithine deacetylase |
| OPB47065 | T4742_S00009.507 | acetylornithine deacetylase |
| OPB42557 | T4742_S00004.660 | Acetyltransf_Acetyltransferase (GNAT) family |
| OPB44558 | T4742_S00003.830 | Acetyltransf_Acetyltransferase (GNAT) family |
| OPB36925 | T4742_S00007.643 | acetyltransferase |
| OPB43679 | T4742_S00016.199 | acetyltransferase |
| OPB41813 | T4742_S00018.136 | acetyltransferase [Trichoderma harzianum] |
| OPB38857 | T4742_S00002.906 | acetyltransferase, GNAT family family |
| OPB38792 | T4742_S00002.841 | acetyl-transferring pyruvate dehydrogenase, E1 component beta subunit |
| OPB43884 | T4742_S00003.155 | acid phosphatase with metalloesterase domain |
| OPB37683 | T4742_S00005.710 | acid phosphatase, putative |
| OPB42866 | T4742_S00012.103 | acid phosphatase-like protein |
| OPB37257 | T4742_S00005.284 | acid sphingomyelin phosphodiesterase (probably vacuolar) |
| OPB43304 | T4742_S00019.37 | acid-CoA ligase |
| OPB41353 | T4742_S00011.194 | aconitase family protein |
| OPB36969 | T4742_S00007.687 | aconitate hydratase |
| OPB37468 | T4742_S00005.495 | aconitate hydratase |
| OPB40140 | T4742_S00001.219 | actin |
| OPB43944 | T4742_S00003.215 | actin polymerization protein Bzz1, putative |
| OPB43903 | T4742_S00003.174 | Actin/actin-like |
| OPB44835 | T4742_S00013.140 | Actin-crosslinking proteins |
| OPB39549 | T4742_S00006.609 | acting-binding cofilin/tropomyosin domain |
| OPB39327 | T4742_S00006.387 | actinin actin binding and calcium-binding EF-hand domains |
| OPB43257 | T4742_S00022.61 | actin-interacting protein AIP3 |
| OPB36805 | T4742_S00007.523 | actin-like protein |
| OPB38206 | T4742_S00002.253 | actin-like protein |
| OPB44300 | T4742_S00003.571 | actin-like protein |
| OPB43588 | T4742_S00016.108 | Actin-like protein 3 |
| OPB43834 | T4742_S00003.105 | actin-like protein; high identity to predicted Fusarium graminearum FG04485.1 |
| OPB46876 | T4742_S00009.318 | actin-related protein 2/3 complex subunit, putative |
| OPB41486 | T4742_S00011.329 | Actin-related protein-like protein [Acremonium chrysogenum ATCC 11550] |
| OPB44574 | T4742_S00003.846 | acyl carrier protein precursor, mitochondrial [NADH-ubiquinone oxidoreductase subunit] |
| OPB36131 | T4742_S00021.48 | Acyl CoA acyltransferase-like protein |
| OPB42408 | T4742_S00004.510 | Acyl CoA binding protein, putative |

| | | |
|---|---|---|
| OPB40678 | T4742_S00001.758 | acyl-CoA dehydrogenase |
| OPB43277 | T4742_S00019.10 | acyl-CoA dehydrogenase |
| OPB44000 | T4742_S00003.271 | Acyl-CoA dehydrogenase |
| OPB36759 | T4742_S00007.477 | acyl-CoA dehydrogenase |
| OPB36828 | T4742_S00007.546 | acyl-CoA dehydrogenase |
| OPB37463 | T4742_S00005.490 | Acyl-CoA dehydrogenase, central region |
| OPB37337 | T4742_S00005.364 | acyl-CoA dehydrogenase, putative |
| OPB46119 | T4742_S00008.186 | Acyl-CoA N-acyltransferase |
| OPB43467 | T4742_S00019.200 | acyl-CoA oxidase [Coccidioides immitis RS] |
| OPB43468 | T4742_S00019.201 | acyl-CoA oxidase [Trichoderma harzianum] |
| OPB36465 | T4742_S00007.182 | Acyl-CoA oxidase/dehydrogenase, type 1 |
| OPB45184 | T4742_S00010.91 | acyl-CoA synthetase |
| OPB46057 | T4742_S00008.123 | acyl-CoA synthetase |
| OPB45207 | T4742_S00010.115 | Acyl-CoA thioesterase |
| OPB40993 | T4742_S00020.17 | Acyl-CoA thioesterase [Metarhizium rileyi RCEF 4871] |
| OPB44436 | T4742_S00003.708 | Acyl-CoA:GlcN-PI inositol acyltransferase |
| OPB38352 | T4742_S00002.400 | Acyl-CoA-binding protein, ACBP |
| OPB36843 | T4742_S00007.561 | acyl-CoA-synthase |
| OPB39258 | T4742_S00006.318 | acylphosphatase, putative |
| OPB40199 | T4742_S00001.278 | Acyltransferase 3 |
| OPB44400 | T4742_S00003.671 | acyltransferase 3 |
| OPB45953 | T4742_S00008.19 | acyltransferase, putative |
| OPB36924 | T4742_S00007.642 | acyltransferases that catalyze diacylglycerol esterification; phospholipid:diacylglycerol acyltransferase |
| OPB45868 | T4742_S00014.203 | ADA, Adenosine deaminase |
| OPB40638 | T4742_S00001.718 | Ada, Zn binding protein |
| OPB42800 | T4742_S00012.37 | Ada, Zn binding protein |
| OPB41908 | T4742_S00004.10 | adaptin N-terminal region. Homologue of yeast GCN1 which encodes a translational activator of GCN4 through activation of GCN2 in response to starvation. |
| OPB41909 | T4742_S00004.11 | adaptin N-terminal region. Homologue of yeast GCN1 which encodes a translational activator of GCN4 through activation of GCN2 in response to starvation. |
| OPB41296 | T4742_S00011.137 | Adenine deaminase/adenosine deaminase |
| OPB43167 | T4742_S00012.404 | adenine nucleotide translocator |
| OPB43363 | T4742_S00019.96 | adenine nucleotide translocator |
| OPB38230 | T4742_S00002.277 | Adenine phosphoribosyl transferases |
| OPB44417 | T4742_S00003.689 | adenine phosphoribosyltransferase |
| OPB39593 | T4742_S00006.653 | Adenosine 5'-phosphosulfate kinase |
| OPB37650 | T4742_S00005.677 | Adenosine deaminase-related growth factor |
| OPB37462 | T4742_S00005.489 | Adenosine deaminase-related growth factors |

| | | |
|---|---|---|
| OPB40494 | T4742_S00001.573 | Adenosine deaminase-related growth factors |
| OPB41093 | T4742_S00020.117 | Adenosine/AMP deaminase |
| OPB44860 | T4742_S00013.165 | Adenosine/AMP deaminase |
| OPB42222 | T4742_S00004.324 | adenosine_kinase |
| OPB45585 | T4742_S00010.496 | Adenosylmethionine/aminooxononanoate aminotransferase |
| OPB39961 | T4742_S00001.40 | Adenosylmethionine--amino--oxononanoate aminotransferase BioA |
| OPB46389 | T4742_S00008.456 | Adenosylmethionine--amino--oxononanoate aminotransferase BioA |
| OPB37043 | T4742_S00005.70 | adenylate cyclase |
| OPB37971 | T4742_S00002.18 | Adenylate cyclase-associated CAP, N-terminal |
| OPB39655 | T4742_S00006.715 | Adenylate kinase |
| OPB44145 | T4742_S00003.416 | adenylate kinase |
| OPB40949 | T4742_S00001.1030 | adenylosuccinate lyase |
| OPB40109 | T4742_S00001.188 | adenylosuccinate synthase |
| OPB45251 | T4742_S00010.160 | adenylosuccinate synthetase |
| OPB41268 | T4742_S00011.109 | ADP-ribose pyrophosphatase |
| OPB46824 | T4742_S00009.266 | ADP-ribosylation factor Arf1 |
| OPB43836 | T4742_S00003.107 | ADP-ribosylation factor Arf6 |
| OPB38358 | T4742_S00002.406 | ADP-ribosylation factor GTPase activating protein |
| OPB41403 | T4742_S00011.245 | ADP-ribosylation factor like protein |
| OPB36896 | T4742_S00007.614 | ADP-ribosylation factor-binding protein Gga2 |
| OPB43033 | T4742_S00012.270 | ADP-ribosylation factor-like protein 2, Ras small GTPase |
| OPB45194 | T4742_S00010.102 | ADP-ribosylation factor-like protein, arl3; GTPase of the Ras superfamily |
| OPB41185 | T4742_S00011.25 | ADP-ribosylation/Crystallin J1 |
| OPB39092 | T4742_S00006.151 | ADP-ribosylglycohydrolase |
| OPB42666 | T4742_S00004.771 | ADP-ribosylglycohydrolase-like protein |
| OPB38144 | T4742_S00002.191 | AFG1-like ATPase |
| OPB47101 | T4742_S00009.543 | Aflatoxin biosynthesis regulatory protein |
| OPB41123 | T4742_S00020.147 | agmatinase |
| OPB40692 | T4742_S00001.772 | agmatinase [Colletotrichum tofieldiae] |
| OPB41495 | T4742_S00011.338 | agmatinase [Colletotrichum tofieldiae] |
| OPB45901 | T4742_S00014.237 | Alanine racemase |
| OPB36402 | T4742_S00007.119 | alanine transaminase-like protein |
| OPB43137 | T4742_S00012.374 | alanine-glyoxylate aminotransferase |
| OPB45834 | T4742_S00014.169 | alanyl-transfer RNA synthetase. |
| OPB44347 | T4742_S00003.618 | alanyl-tRNA synthetase, class IIc. |
| OPB43589 | T4742_S00016.109 | alcohol dehydrogenase |

| | | |
|---|---|---|
| OPB36256 | T4742_S00021.174 | alcohol dehydrogenase [Trichoderma harzianum] |
| OPB43244 | T4742_S00022.47 | Alcohol dehydrogenase superfamily, zinc-containing [Penicillium camemberti] |
| OPB36059 | T4742_S00024.20 | aldehyde dehydrogenase |
| OPB36100 | T4742_S00021.16 | aldehyde dehydrogenase |
| OPB36779 | T4742_S00007.497 | aldehyde dehydrogenase |
| OPB39805 | T4742_S00015.149 | Aldehyde dehydrogenase |
| OPB40216 | T4742_S00001.295 | Aldehyde dehydrogenase |
| OPB40615 | T4742_S00001.695 | Aldehyde dehydrogenase |
| OPB40747 | T4742_S00001.828 | aldehyde dehydrogenase |
| OPB40763 | T4742_S00001.844 | aldehyde dehydrogenase |
| OPB40776 | T4742_S00001.857 | aldehyde dehydrogenase |
| OPB41251 | T4742_S00011.92 | aldehyde dehydrogenase |
| OPB41254 | T4742_S00011.95 | Aldehyde dehydrogenase |
| OPB42155 | T4742_S00004.257 | aldehyde dehydrogenase |
| OPB42775 | T4742_S00012.12 | aldehyde dehydrogenase |
| OPB43072 | T4742_S00012.309 | Aldehyde dehydrogenase |
| OPB43669 | T4742_S00016.189 | aldehyde dehydrogenase |
| OPB44831 | T4742_S00013.136 | Aldehyde dehydrogenase |
| OPB44974 | T4742_S00013.279 | Aldehyde dehydrogenase |
| OPB45929 | T4742_S00014.266 | aldehyde dehydrogenase |
| OPB46227 | T4742_S00008.294 | aldehyde dehydrogenase |
| OPB46365 | T4742_S00008.432 | aldehyde dehydrogenase |
| OPB46418 | T4742_S00008.485 | aldehyde dehydrogenase |
| OPB46540 | T4742_S00008.607 | aldehyde dehydrogenase |
| OPB46853 | T4742_S00009.295 | Aldehyde dehydrogenase |
| OPB36270 | T4742_S00021.189 | aldehyde dehydrogenase |
| OPB38385 | T4742_S00002.433 | aldehyde dehydrogenase |
| OPB44744 | T4742_S00013.49 | aldehyde dehydrogenase (NAD(P)(+)) ALD5 [Sugiyamaella lignohabitans] |
| OPB40221 | T4742_S00001.300 | aldehyde dehydrogenase [Colletotrichum gloeosporioides Cg-14] |
| OPB45490 | T4742_S00010.401 | Aldehyde dehydrogenase family protein [Aspergillus parasiticus SU-1] |
| OPB44287 | T4742_S00003.558 | aldehyde dehydrogenase family protein [Metarhizium robertsii] |
| OPB43471 | T4742_S00019.204 | Aldehyde dehydrogenase, C-terminal [Penicillium expansum] |
| OPB45378 | T4742_S00010.289 | aldehyde reductase |
| OPB38654 | T4742_S00002.703 | aldehyde reductase (GliO) |
| OPB46531 | T4742_S00008.598 | aldehyde reductase (GliO) [Trichoderma guizhouense] |
| OPB39303 | T4742_S00006.363 | aldehyde reductase [Trichoderma guizhouense] |

| | | |
|---|---|---|
| OPB40658 | T4742_S00001.738 | aldehyde reductase II [Colletotrichum higginsianum] |
| OPB42827 | T4742_S00012.64 | aldehyde reductase member 2 |
| OPB45777 | T4742_S00014.112 | aldo keto reductase [Colletotrichum orbiculare MAFF 240422] |
| OPB45755 | T4742_S00014.90 | Aldo/keto reductase [Aureobasidium pullulans EXF-150] |
| OPB37160 | T4742_S00005.187 | Aldo/keto reductase [Trichoderma guizhouense] |
| OPB41543 | T4742_S00011.386 | aldolase, class II |
| OPB41016 | T4742_S00020.40 | aldolase/citrate lyase family protein |
| OPB36702 | T4742_S00007.420 | aldose-1-epimerase |
| OPB37888 | T4742_S00017.155 | aldose-1-epimerase |
| OPB40310 | T4742_S00001.389 | alginate lyase [Actinobacteria bacterium OK006] |
| OPB40653 | T4742_S00001.733 | alginate lyase [Penicillium brasilianum] |
| OPB37407 | T4742_S00005.434 | Alkaline phosphatase |
| OPB40013 | T4742_S00001.92 | Alkaline phosphatase |
| OPB40950 | T4742_S00001.1031 | Alkaline phosphatase |
| OPB42788 | T4742_S00012.25 | Alkaline phytoceramidase |
| OPB37691 | T4742_S00005.718 | Alkaline proteinase [Trichoderma guizhouense] |
| OPB46218 | T4742_S00008.285 | Alkyl hydroperoxide reductase |
| OPB38500 | T4742_S00002.549 | Alkyl hydroperoxide reductase/peroxiredoxin |
| OPB39089 | T4742_S00006.148 | allantoate permease, 10 TM domains |
| OPB43841 | T4742_S00003.112 | allantoate permease, 10 TM domains |
| OPB44859 | T4742_S00013.164 | allantoate permease, 10 TM domains |
| OPB38943 | T4742_S00006.2 | Allantoicase |
| OPB40384 | T4742_S00001.463 | allantoin permease [Aspergillus kawachii IFO 4308] |
| OPB42046 | T4742_S00004.148 | Allergen V5/Tpx-1 related |
| OPB42750 | T4742_S00004.856 | alpha methylacyl-CoA racemase |
| OPB36214 | T4742_S00021.132 | alpha/beta hydrolase, partial [Metarhizium brunneum ARSEF 3297] |
| OPB40632 | T4742_S00001.712 | alpha/beta-hydrolase [Coniochaeta ligniaria NRRL 30616] |
| OPB40567 | T4742_S00001.646 | alpha/beta-hydrolase [Glonium stellatum] |
| OPB40324 | T4742_S00001.403 | alpha/beta-hydrolase [Phialocephala scopiformis] |
| OPB39618 | T4742_S00006.678 | alpha-1,6-mannosyltransferase subunit |
| OPB38875 | T4742_S00002.924 | alpha-1,6-mannosyltransferase subunit [Trichoderma guizhouense] |
| OPB41804 | T4742_S00018.127 | alpha-acetolactate decarboxylase [Fusarium oxysporum f. sp. raphani 54005] |
| OPB39309 | T4742_S00006.369 | alpha-aminoadipate reductase lys2 |
| OPB44039 | T4742_S00003.310 | alpha-amylase |
| OPB36233 | T4742_S00021.151 | alpha-ketoglutarate (FeII) dependent dioxygenase |
| OPB37157 | T4742_S00005.184 | alpha-ketoglutarate (FeII) dependent dioxygenase |

| | | |
|---|---|---|
| OPB37530 | T4742_S00005.557 | alpha-ketoglutarate (FeII) dependent dioxygenase |
| OPB39067 | T4742_S00006.126 | alpha-ketoglutarate (FeII) dependent dioxygenase |
| OPB40806 | T4742_S00001.887 | alpha-ketoglutarate (FeII) dependent dioxygenase |
| OPB42691 | T4742_S00004.797 | alpha-ketoglutarate (FeII) dependent dioxygenase |
| OPB45875 | T4742_S00014.210 | alpha-ketoglutarate (FeII) dependent dioxygenase |
| OPB41779 | T4742_S00018.102 | alpha-ketoglutarate-(FeII) dependent dioxygenase |
| OPB37208 | T4742_S00005.235 | alpha-ketoglutarate-dependent sulfonate dioxygenase |
| OPB45102 | T4742_S00010.9 | Alpha-ketoglutarate-dependent sulfonate dioxygenase [Colletotrichum higginsianum IMI 349063] |
| OPB41379 | T4742_S00011.220 | alpha-ketoglutarate-dependent taurine dioxygenase [Trichoderma guizhouense] |
| OPB42514 | T4742_S00004.616 | alpha-tubulin cofactor B. |
| OPB40962 | T4742_S00001.1043 | alpha-tubulin suppressor protein Aats1 |
| OPB40110 | T4742_S00001.189 | Altered inheritance of mitochondria protein 9, mitochondrial [Madurella mycetomatis] |
| OPB41102 | T4742_S00020.126 | alternative NADH-dehydrogenase |
| OPB39606 | T4742_S00006.666 | alternative oxidase aox1 |
| OPB43504 | T4742_S00016.24 | AMA1, activator of meiotic anaphase promoting complex |
| OPB36074 | T4742_S00024.35 | amidase |
| OPB36184 | T4742_S00021.102 | amidase |
| OPB36325 | T4742_S00007.42 | amidase |
| OPB36421 | T4742_S00007.138 | amidase |
| OPB37631 | T4742_S00005.658 | amidase |
| OPB38036 | T4742_S00002.83 | amidase |
| OPB38468 | T4742_S00002.516 | amidase |
| OPB38822 | T4742_S00002.871 | Amidase |
| OPB38859 | T4742_S00002.908 | Amidase |
| OPB39133 | T4742_S00006.193 | amidase |
| OPB39297 | T4742_S00006.357 | amidase |
| OPB39306 | T4742_S00006.366 | amidase |
| OPB40403 | T4742_S00001.482 | Amidase |
| OPB40404 | T4742_S00001.483 | amidase |
| OPB40428 | T4742_S00001.507 | Amidase |
| OPB40556 | T4742_S00001.635 | Amidase |
| OPB40569 | T4742_S00001.648 | amidase |
| OPB40710 | T4742_S00001.790 | amidase |
| OPB40842 | T4742_S00001.923 | amidase |
| OPB40932 | T4742_S00001.1013 | amidase |
| OPB41179 | T4742_S00011.19 | amidase |

| | | |
|---|---|---|
| OPB41223 | T4742_S00011.64 | amidase |
| OPB41255 | T4742_S00011.96 | Amidase |
| OPB41320 | T4742_S00011.161 | amidase |
| OPB41782 | T4742_S00018.105 | amidase |
| OPB41842 | T4742_S00018.165 | amidase |
| OPB41847 | T4742_S00018.170 | amidase |
| OPB42111 | T4742_S00004.213 | amidase |
| OPB43739 | T4742_S00003.10 | amidase |
| OPB43853 | T4742_S00003.124 | amidase |
| OPB43969 | T4742_S00003.240 | amidase |
| OPB44015 | T4742_S00003.286 | Amidase |
| OPB44423 | T4742_S00003.695 | amidase |
| OPB44623 | T4742_S00003.895 | amidase |
| OPB44786 | T4742_S00013.91 | Amidase |
| OPB44876 | T4742_S00013.181 | amidase |
| OPB44931 | T4742_S00013.236 | amidase |
| OPB45326 | T4742_S00010.237 | Amidase |
| OPB45760 | T4742_S00014.95 | amidase |
| OPB45829 | T4742_S00014.164 | amidase |
| OPB46224 | T4742_S00008.291 | amidase |
| OPB46228 | T4742_S00008.295 | amidase |
| OPB46554 | T4742_S00008.621 | amidase |
| OPB46699 | T4742_S00009.141 | Amidase |
| OPB47135 | T4742_S00009.577 | amidase |
| OPB36093 | T4742_S00021.9 | amidase [Trichoderma harzianum] |
| OPB44737 | T4742_S00013.42 | amine dehydrogenase |
| OPB45649 | T4742_S00010.560 | amine dehydrogenase |
| OPB45919 | T4742_S00014.256 | amine oxidase |
| OPB36149 | T4742_S00021.66 | amino acid permease |
| OPB36272 | T4742_S00021.191 | amino acid permease |
| OPB36345 | T4742_S00007.62 | amino acid permease |
| OPB36782 | T4742_S00007.500 | amino acid permease |
| OPB37330 | T4742_S00005.357 | amino acid permease |
| OPB37395 | T4742_S00005.422 | amino acid permease |
| OPB37676 | T4742_S00005.703 | amino acid permease |
| OPB37953 | T4742_S00017.220 | amino acid permease |

| | | |
|---|---|---|
| OPB38876 | T4742_S00002.925 | amino acid permease |
| OPB39085 | T4742_S00006.144 | Amino acid permease |
| OPB39183 | T4742_S00006.243 | amino acid permease |
| OPB39313 | T4742_S00006.373 | amino acid permease |
| OPB40078 | T4742_S00001.157 | amino acid permease |
| OPB40397 | T4742_S00001.476 | amino acid permease |
| OPB40411 | T4742_S00001.490 | amino acid permease |
| OPB40470 | T4742_S00001.549 | amino acid permease |
| OPB40691 | T4742_S00001.771 | amino acid permease |
| OPB40761 | T4742_S00001.842 | Amino acid permease |
| OPB40933 | T4742_S00001.1014 | amino acid permease |
| OPB41017 | T4742_S00020.41 | amino acid permease |
| OPB41247 | T4742_S00011.88 | amino acid permease |
| OPB41368 | T4742_S00011.209 | amino acid permease |
| OPB41393 | T4742_S00011.235 | amino acid permease |
| OPB42525 | T4742_S00004.627 | amino acid permease |
| OPB42564 | T4742_S00004.667 | amino acid permease |
| OPB42844 | T4742_S00012.81 | Amino acid permease |
| OPB43408 | T4742_S00019.141 | amino acid permease |
| OPB43423 | T4742_S00019.156 | amino acid permease |
| OPB43424 | T4742_S00019.157 | amino acid permease |
| OPB43444 | T4742_S00019.177 | Amino acid permease |
| OPB43968 | T4742_S00003.239 | amino acid permease |
| OPB44629 | T4742_S00003.901 | amino acid permease |
| OPB45263 | T4742_S00010.172 | amino acid permease |
| OPB45271 | T4742_S00010.182 | amino acid permease |
| OPB45625 | T4742_S00010.536 | amino acid permease |
| OPB45743 | T4742_S00014.78 | amino acid permease |
| OPB46207 | T4742_S00008.274 | amino acid permease |
| OPB46268 | T4742_S00008.335 | amino acid permease |
| OPB46412 | T4742_S00008.479 | amino acid permease |
| OPB46498 | T4742_S00008.565 | amino acid permease |
| OPB46539 | T4742_S00008.606 | amino acid permease |
| OPB46552 | T4742_S00008.619 | amino acid permease |
| OPB46805 | T4742_S00009.247 | amino acid permease |
| OPB46873 | T4742_S00009.315 | Amino acid permease |

| | | |
|---|---|---|
| OPB46938 | T4742_S00009.380 | amino acid permease |
| OPB43952 | T4742_S00003.223 | amino acid permease |
| OPB43443 | T4742_S00019.176 | amino acid permease [Trichoderma harzianum] |
| OPB36477 | T4742_S00007.194 | amino acid permease, methionine |
| OPB39597 | T4742_S00006.657 | aminoacyl-tRNA synthase. |
| OPB36418 | T4742_S00007.135 | aminoacyl-tRNA synthetase |
| OPB42738 | T4742_S00004.844 | Aminoglycoside phosphotransferase [Cordyceps confragosa RCEF 1005] |
| OPB40456 | T4742_S00001.535 | Aminoglycoside phosphotransferase [Cordyceps militaris CM01] |
| OPB46985 | T4742_S00009.427 | Aminoglycoside phosphotransferase [Metarhizium robertsii ARSEF 23] |
| OPB46131 | T4742_S00008.198 | aminoglycoside phosphotransferase [Trichoderma guizhouense] |
| OPB45935 | T4742_S00008.1 | Aminopeptidase P |
| OPB39170 | T4742_S00006.230 | aminopeptidase Y |
| OPB41979 | T4742_S00004.81 | aminopeptidase-like protein [Hypoxylon sp. EC38] |
| OPB38618 | T4742_S00002.667 | aminotransferase |
| OPB38619 | T4742_S00002.668 | aminotransferase |
| OPB43767 | T4742_S00003.38 | aminotransferase |
| OPB44771 | T4742_S00013.76 | aminotransferase |
| OPB45137 | T4742_S00010.44 | aminotransferase |
| OPB46479 | T4742_S00008.546 | aminotransferase |
| OPB47047 | T4742_S00009.489 | aminotransferase |
| OPB44838 | T4742_S00013.143 | ammonium permease |
| OPB42100 | T4742_S00004.202 | ammonium permease MEA1 |
| OPB43356 | T4742_S00019.89 | AMP deaminase |
| OPB43509 | T4742_S00016.29 | AMP deaminase |
| OPB37831 | T4742_S00017.98 | AMP-dependent synthetase and ligase [Trichoderma guizhouense] |
| OPB39026 | T4742_S00006.85 | AMP-dependent synthetase and ligase [Trichoderma guizhouense] |
| OPB39335 | T4742_S00006.395 | AMP-dependent synthetase and ligase [Trichoderma guizhouense] |
| OPB37567 | T4742_S00005.594 | AMP-dependent synthetase and ligase, acetoacetyl-CoA synthase-like [Trichoderma guizhouense] |
| OPB37851 | T4742_S00017.118 | anaphase promoting complex subunit APC11 |
| OPB38552 | T4742_S00002.601 | anaphase-promoting complex protein |
| OPB37552 | T4742_S00005.579 | anaphase-promoting complex subunit cut9 |
| OPB40784 | T4742_S00001.865 | Ank-repeat protein mbp1 [Colletotrichum higginsianum IMI 349063] |
| OPB36014 | T4742_S00023.49 | Ankyrin |
| OPB36427 | T4742_S00007.144 | Ankyrin |
| OPB36460 | T4742_S00007.177 | ankyrin |
| OPB36865 | T4742_S00007.583 | ankyrin |

| | | |
|---|---|---|
| OPB37030 | T4742_S00005.57 | Ankyrin |
| OPB37098 | T4742_S00005.125 | Ankyrin |
| OPB37151 | T4742_S00005.178 | Ankyrin |
| OPB37316 | T4742_S00005.343 | Ankyrin |
| OPB38107 | T4742_S00002.154 | Ankyrin |
| OPB38573 | T4742_S00002.622 | Ankyrin |
| OPB38755 | T4742_S00002.804 | Ankyrin |
| OPB38761 | T4742_S00002.810 | ankyrin |
| OPB38863 | T4742_S00002.912 | ankyrin |
| OPB38934 | T4742_S00002.983 | Ankyrin |
| OPB39071 | T4742_S00006.130 | Ankyrin |
| OPB39378 | T4742_S00006.438 | Ankyrin |
| OPB39716 | T4742_S00015.60 | ankyrin |
| OPB39958 | T4742_S00001.37 | ankyrin |
| OPB40202 | T4742_S00001.281 | ankyrin |
| OPB40453 | T4742_S00001.532 | ankyrin |
| OPB40882 | T4742_S00001.963 | Ankyrin |
| OPB41000 | T4742_S00020.24 | ankyrin |
| OPB41134 | T4742_S00020.158 | Ankyrin |
| OPB41319 | T4742_S00011.160 | ankyrin |
| OPB41547 | T4742_S00011.390 | ankyrin |
| OPB41681 | T4742_S00018.3 | ankyrin |
| OPB41799 | T4742_S00018.122 | ankyrin |
| OPB41964 | T4742_S00004.66 | Ankyrin |
| OPB42886 | T4742_S00012.123 | ankyrin |
| OPB43637 | T4742_S00016.157 | ankyrin |
| OPB44043 | T4742_S00003.314 | Ankyrin |
| OPB44314 | T4742_S00003.585 | Ankyrin |
| OPB44478 | T4742_S00003.750 | ankyrin |
| OPB44479 | T4742_S00003.751 | Ankyrin |
| OPB44617 | T4742_S00003.889 | Ankyrin |
| OPB44870 | T4742_S00013.175 | Ankyrin |
| OPB44939 | T4742_S00013.244 | ankyrin |
| OPB45095 | T4742_S00010.2 | Ankyrin |
| OPB45206 | T4742_S00010.114 | Ankyrin |
| OPB45528 | T4742_S00010.439 | Ankyrin |

| | | |
|---|---|---|
| OPB45571 | T4742_S00010.482 | ankyrin |
| OPB45722 | T4742_S00014.56 | ankyrin |
| OPB45737 | T4742_S00014.72 | ankyrin |
| OPB45810 | T4742_S00014.145 | ankyrin |
| OPB46160 | T4742_S00008.227 | Ankyrin |
| OPB46465 | T4742_S00008.532 | ankyrin |
| OPB46466 | T4742_S00008.533 | Ankyrin |
| OPB46656 | T4742_S00009.98 | ankyrin |
| OPB46730 | T4742_S00009.172 | ankyrin |
| OPB46923 | T4742_S00009.365 | Ankyrin |
| OPB46967 | T4742_S00009.409 | Ankyrin |
| OPB47160 | T4742_S00009.602 | Ankyrin |
| OPB35975 | T4742_S00023.10 | ankyrin |
| OPB35997 | T4742_S00023.32 | ankyrin |
| OPB36873 | T4742_S00007.591 | ankyrin |
| OPB37001 | T4742_S00005.28 | ankyrin |
| OPB37505 | T4742_S00005.532 | ankyrin |
| OPB37511 | T4742_S00005.538 | ankyrin |
| OPB37534 | T4742_S00005.561 | ankyrin |
| OPB37654 | T4742_S00005.681 | ankyrin |
| OPB37934 | T4742_S00017.201 | ankyrin |
| OPB38814 | T4742_S00002.863 | ankyrin |
| OPB38942 | T4742_S00006.1 | ankyrin |
| OPB39165 | T4742_S00006.225 | ankyrin |
| OPB39220 | T4742_S00006.280 | ankyrin |
| OPB39602 | T4742_S00006.662 | ankyrin |
| OPB40218 | T4742_S00001.297 | ankyrin |
| OPB40358 | T4742_S00001.437 | ankyrin |
| OPB40433 | T4742_S00001.512 | ankyrin |
| OPB40459 | T4742_S00001.538 | ankyrin |
| OPB40809 | T4742_S00001.890 | ankyrin |
| OPB41048 | T4742_S00020.72 | ankyrin |
| OPB41068 | T4742_S00020.92 | ankyrin |
| OPB41130 | T4742_S00020.154 | ankyrin |
| OPB41775 | T4742_S00018.98 | ankyrin |
| OPB42070 | T4742_S00004.172 | ankyrin |

| | | |
|---|---|---|
| OPB42080 | T4742_S00004.182 | ankyrin |
| OPB42501 | T4742_S00004.603 | ankyrin |
| OPB42720 | T4742_S00004.826 | ankyrin |
| OPB42877 | T4742_S00012.114 | ankyrin |
| OPB44877 | T4742_S00013.182 | ankyrin |
| OPB44910 | T4742_S00013.215 | ankyrin |
| OPB44958 | T4742_S00013.263 | ankyrin |
| OPB44973 | T4742_S00013.278 | ankyrin |
| OPB45429 | T4742_S00010.340 | ankyrin |
| OPB45486 | T4742_S00010.397 | ankyrin |
| OPB45637 | T4742_S00010.548 | ankyrin |
| OPB45793 | T4742_S00014.128 | ankyrin |
| OPB46113 | T4742_S00008.180 | ankyrin |
| OPB46191 | T4742_S00008.258 | ankyrin |
| OPB46269 | T4742_S00008.336 | ankyrin |
| OPB46410 | T4742_S00008.477 | ankyrin |
| OPB46596 | T4742_S00009.38 | ankyrin |
| OPB46669 | T4742_S00009.111 | ankyrin |
| OPB46716 | T4742_S00009.158 | ankyrin |
| OPB40465 | T4742_S00001.544 | ankyrin [Aschersonia aleyrodis RCEF 2490] |
| OPB40228 | T4742_S00001.307 | ankyrin [Fusarium fujikuroi] |
| OPB42533 | T4742_S00004.635 | ankyrin [Fusarium fujikuroi] |
| OPB42857 | T4742_S00012.94 | ankyrin [Fusarium fujikuroi] |
| OPB40466 | T4742_S00001.545 | ankyrin 3 [Fusarium fujikuroi] |
| OPB46235 | T4742_S00008.302 | ankyrin repeat domain protein, putative [Talaromyces stipitatus ATCC 10500] |
| OPB45101 | T4742_S00010.8 | ankyrin repeat protein [Colletotrichum gloeosporioides Nara gc5] |
| OPB46030 | T4742_S00008.96 | ankyrin repeat protein [Metarhizium robertsii] |
| OPB46063 | T4742_S00008.130 | ankyrin repeat protein [Metarhizium robertsii] |
| OPB46667 | T4742_S00009.109 | ankyrin repeat protein [Metarhizium robertsii] |
| OPB41144 | T4742_S00020.168 | ankyrin repeat protein [Trichoderma guizhouense] |
| OPB41991 | T4742_S00004.93 | ankyrin repeat protein [Trichoderma guizhouense] |
| OPB42534 | T4742_S00004.636 | ankyrin repeat protein [Trichoderma guizhouense] |
| OPB39839 | T4742_S00015.183 | ankyrin repeat protein [Trichoderma harzianum] |
| OPB36274 | T4742_S00021.193 | ankyrin repeat protein, partial [Metarhizium anisopliae ARSEF 549] |
| OPB45419 | T4742_S00010.330 | ankyrin/NACHT protein |
| OPB46851 | T4742_S00009.293 | Annexin A7 |

| | | |
|---|---|---|
| OPB41647 | T4742_S00011.490 | annexin ANXC4 |
| OPB45152 | T4742_S00010.59 | anthranilate synthase component II [Beauveria bassiana ARSEF 2860] |
| OPB40213 | T4742_S00001.292 | AP-3 complex subunit mu-1 [Escovopsis weberi] |
| OPB37192 | T4742_S00005.219 | Apm1, of the adaptor protein complex AP-1 of clathrin-coated vesicles |
| OPB44825 | T4742_S00013.130 | Apm4, subunit of adaptor protein complex AP-2 of clathrin-coated vesicles |
| OPB44830 | T4742_S00013.135 | Apoptosis regulator Bcl-2 protein, BAG |
| OPB39494 | T4742_S00006.554 | aps1, subunit of the adaptor protein complex AP-1 |
| OPB38399 | T4742_S00002.447 | aps2, of the adaptor protein complex AP-2 of clathrin-coated vesicles, small subunit |
| OPB43182 | T4742_S00012.419 | Aps3, of the adaptor protein complex AP-3 |
| OPB38531 | T4742_S00002.580 | apsA, Protein with homology to anucleate Primary sterigmata (Aspergillus nidulans) |
| OPB39622 | T4742_S00006.682 | apurinic/apyrimidinic endonuclease-like protein |
| OPB39154 | T4742_S00006.214 | aquaglyceroporin |
| OPB40314 | T4742_S00001.393 | aquaglyceroporin |
| OPB44053 | T4742_S00003.324 | aquaglyceroporin |
| OPB38877 | T4742_S00002.926 | Aquaporin |
| OPB39740 | T4742_S00015.84 | Aquaporin |
| OPB45845 | T4742_S00014.180 | Aquaporin |
| OPB39866 | T4742_S00015.210 | Arc15 (N.crassa) ortholog |
| OPB38061 | T4742_S00002.108 | ARF GAP zinc finger protein Gcs1 |
| OPB43486 | T4742_S00016.6 | ARF GEF2 Gea2 |
| OPB35979 | T4742_S00023.14 | ARF GTPase activator |
| OPB43004 | T4742_S00012.241 | ARF GTPase activator (Csx2), putative |
| OPB46952 | T4742_S00009.394 | ARF-GAP effector |
| OPB42441 | T4742_S00004.543 | Arfs GEF Sec7 |
| OPB45228 | T4742_S00010.137 | ARG5,6 |
| OPB39508 | T4742_S00006.568 | ArgE, Acetylornithine deacetylase/Succinyl-diaminopimelate desuccinylase |
| OPB40526 | T4742_S00001.605 | ArgE, Acetylornithine deacetylase/Succinyl-diaminopimelate desuccinylase |
| OPB36513 | T4742_S00007.230 | Arginase family protein |
| OPB37908 | T4742_S00017.175 | Arginase SpeB |
| OPB41050 | T4742_S00020.74 | arginine deiminase type-3 |
| OPB39644 | T4742_S00006.704 | arginine N-methyltransferase |
| OPB43663 | T4742_S00016.183 | arginine N-methyltransferase |
| OPB43138 | T4742_S00012.375 | arginine N-methyltransferase skb1 |
| OPB43567 | T4742_S00016.87 | arginine-tRNA-protein transferase. |
| OPB42082 | T4742_S00004.184 | arginosuccinate synthetase |
| OPB39607 | T4742_S00006.667 | Arginyl-tRNA ligase. |

| | | |
|---|---|---|
| OPB36331 | T4742_S00007.48 | Argonaute and Dicer protein, PAZ |
| OPB45463 | T4742_S00010.374 | Argonaute siRNA chaperone (ARC) complex subunit |
| OPB36712 | T4742_S00007.430 | arid/bright domain protein, AT-rich interaction region |
| OPB39586 | T4742_S00006.646 | ARO Transcriptional regulator [Trichoderma guizhouense] |
| OPB38753 | T4742_S00002.802 | ARO8, Transcriptional regulator [Trichoderma guizhouense] |
| OPB46360 | T4742_S00008.427 | aromatic amino acid aminotransferase |
| OPB38007 | T4742_S00002.54 | Aromatic L-amino acid decarboxylase |
| OPB40921 | T4742_S00001.1002 | aromatic ring-opening dioxygenase LigB subunit, putative |
| OPB42919 | T4742_S00012.156 | ARP2/3 complex 34 kDa subunit [Trichoderma harzianum] |
| OPB39669 | T4742_S00015.13 | ARP2/3 complex, 21 kDa p21-Arc subunit |
| OPB42918 | T4742_S00012.155 | Arp2/3 complex, 34kDa subunit p34-Arc |
| OPB38643 | T4742_S00002.692 | ARP23 complex 20 kDa subunit |
| OPB44515 | T4742_S00003.787 | arsenate reductase Arc2 |
| OPB44514 | T4742_S00003.786 | arsenical resistance protein ArsH |
| OPB45989 | T4742_S00008.55 | arsenite methyltransferase |
| OPB37959 | T4742_S00002.6 | Arv1-like family protein [Trichoderma guizhouense] |
| OPB36085 | T4742_S00021.1 | aryl-alcohol dehydrogenase |
| OPB46477 | T4742_S00008.544 | arylamine N-acetyltransferase 1 |
| OPB44757 | T4742_S00013.62 | arylamine N-acetyltransferase 2 |
| OPB36642 | T4742_S00007.360 | arylsulfatase |
| OPB44484 | T4742_S00003.756 | arylsulfatase |
| OPB46005 | T4742_S00008.71 | arylsulfatase |
| OPB44483 | T4742_S00003.755 | arylsulfatase |
| OPB40665 | T4742_S00001.745 | arylsulfatase [Pseudomonas chlororaphis] |
| OPB40005 | T4742_S00001.84 | Asparaginase |
| OPB40534 | T4742_S00001.613 | Asparaginase |
| OPB37092 | T4742_S00005.119 | asparagine synthase |
| OPB45658 | T4742_S00010.569 | asparagine synthase |
| OPB46890 | T4742_S00009.332 | asparagine synthase-like protein |
| OPB40702 | T4742_S00001.782 | asparaginyl-tRNA synthetase |
| OPB40673 | T4742_S00001.753 | aspartate aminotransferase |
| OPB40785 | T4742_S00001.866 | aspartate aminotransferase |
| OPB39972 | T4742_S00001.51 | aspartate kinase |
| OPB43412 | T4742_S00019.145 | aspartate racemase |
| OPB37919 | T4742_S00017.186 | Aspartate/tyrosine/aromatic aminotransferase |
| OPB38334 | T4742_S00002.382 | Aspartate/tyrosine/aromatic aminotransferase |

| | | |
|---|---|---|
| OPB42373 | T4742_S00004.475 | Aspartate/tyrosine/aromatic aminotransferase |
| OPB43516 | T4742_S00016.36 | Aspartate/tyrosine/aromatic aminotransferase |
| OPB35951 | T4742_S00025.24 | Aspartate-semialdehyde dehydrogenase |
| OPB36212 | T4742_S00021.130 | aspartyl protease |
| OPB36280 | T4742_S00021.199 | aspartyl protease |
| OPB36755 | T4742_S00007.473 | aspartyl protease |
| OPB37368 | T4742_S00005.395 | aspartyl protease |
| OPB37918 | T4742_S00017.185 | aspartyl protease |
| OPB38613 | T4742_S00002.662 | aspartyl protease |
| OPB39078 | T4742_S00006.137 | aspartyl protease |
| OPB40378 | T4742_S00001.457 | aspartyl protease |
| OPB41714 | T4742_S00018.37 | Aspartyl protease |
| OPB43114 | T4742_S00012.351 | aspartyl protease |
| OPB43162 | T4742_S00012.399 | aspartyl protease |
| OPB43461 | T4742_S00019.194 | aspartyl protease |
| OPB44618 | T4742_S00003.890 | aspartyl protease |
| OPB46159 | T4742_S00008.226 | aspartyl protease |
| OPB46545 | T4742_S00008.612 | aspartyl protease |
| OPB47166 | T4742_S00009.608 | aspartyl protease |
| OPB43587 | T4742_S00016.107 | Aspartylglucosaminidase family |
| OPB44152 | T4742_S00003.423 | aspartyl-tRNA synthase. |
| OPB43798 | T4742_S00003.69 | Aspartyl-tRNA synthetase, class IIb |
| OPB38048 | T4742_S00002.95 | aspartyl-tRNA synthetase, class IIb. |
| OPB44299 | T4742_S00003.570 | Aspartyl-tRNA synthetases |
| OPB42621 | T4742_S00004.725 | AT DNA binding protein, putative |
| OPB45316 | T4742_S00010.227 | ATG  protein Atg22 |
| OPB46714 | T4742_S00009.156 | ATG protein 16 |
| OPB44263 | T4742_S00003.534 | ATG protein ATG6 |
| OPB38812 | T4742_S00002.861 | ATG protein IMH1, mediates transport between an endosomal compartment and the Golgi |
| OPB46645 | T4742_S00009.87 | ATG protein, AUT7 |
| OPB45144 | T4742_S00010.51 | ATG1 autophagy protein |
| OPB36531 | T4742_S00007.248 | ATG13 autophagy related, unknown protein |
| OPB39124 | T4742_S00006.184 | ATG17_NEUCR RecName: Full=Autophagy-related protein 17 |
| OPB41674 | T4742_S00011.517 | ATG18 |
| OPB44002 | T4742_S00003.273 | ATG2, C-terminal |
| OPB37034 | T4742_S00005.61 | ATG22_NEUCR RecName: Full=Autophagy-related protein 22 |

| | | |
|---|---|---|
| OPB44559 | T4742_S00003.831 | ATG26 |
| OPB38528 | T4742_S00002.577 | ATG3_NEUCR RecName: Full=Autophagy-related protein 3; AltName: Full=Autophagy-related E2-like conjugation enzyme atg-3 |
| OPB37741 | T4742_S00017.8 | ATG4_NEUCR RecName: Full=Probable cysteine protease atg-4; AltName: Full=Autophagy-related protein 4 |
| OPB37455 | T4742_S00005.482 | ATG5 |
| OPB44067 | T4742_S00003.338 | ATG7_NEUCR RecName: Full=Ubiquitin-like modifier-activating enzyme atg-7; AltName: Full=ATG12-activating enzyme E1 atg-7; AltName: Full=Autophagy-related protein 7 |
| OPB43513 | T4742_S00016.33 | ATG8 |
| OPB36945 | T4742_S00007.663 | ATG9 |
| OPB39059 | T4742_S00006.118 | ATP adenylyltransferase, putative |
| OPB42642 | T4742_S00004.747 | ATP binding protein |
| OPB43149 | T4742_S00012.386 | ATP citrate lyase, alpha subunit |
| OPB43150 | T4742_S00012.387 | ATP citrate lyase, beta subunit |
| OPB37261 | T4742_S00005.288 | ATP dependent DNA ligase domain-containing protein |
| OPB43498 | T4742_S00016.18 | ATP phosphoribosyltransferaseHisG |
| OPB43076 | T4742_S00012.313 | ATP syntase delta chain |
| OPB46749 | T4742_S00009.191 | ATP synthase alpha chain, mitochondrial precursor |
| OPB41668 | T4742_S00011.511 | ATP synthase beta chain, mitochondrial precursor, associated to cellulase signal transduction (PMID: 15288024) |
| OPB41606 | T4742_S00011.449 | ATP synthase D chain, mitochondrial |
| OPB39877 | T4742_S00015.221 | ATP synthase delta subunit |
| OPB38210 | T4742_S00002.257 | ATP synthase regulation protein NCA2, putative |
| OPB42902 | T4742_S00012.139 | ATP synthase subunit 4, mitochondrial precursor |
| OPB35963 | T4742_S00025.36 | ATP synthase subunit E, putative |
| OPB38983 | T4742_S00006.42 | ATP synthase subunit gamma precursor |
| OPB37958 | T4742_S00002.5 | ATP synthase subunit H, putative |
| OPB41541 | T4742_S00011.384 | ATP11 protein |
| OPB38624 | T4742_S00002.673 | ATP12 chaperone , putative |
| OPB44991 | T4742_S00013.296 | ATPase |
| OPB43127 | T4742_S00012.364 | ATPase Sec18, required for ER to Golgi transport etc |
| OPB45374 | T4742_S00010.285 | ATPase synthesis protein 25, mitochondrial [Trichoderma reesei RUT C-30] |
| OPB39506 | T4742_S00006.566 | ATPase, anion transport |
| OPB42375 | T4742_S00004.477 | ATPase, F1/V1/A1 complex, alpha/beta subunit, nucleotide-binding |
| OPB36515 | T4742_S00007.232 | ATPase, P-type, K/Mg/Cd/Cu/Zn/Na/Ca/Na/H-transporter |
| OPB41664 | T4742_S00011.507 | ATPase, V-type |
| OPB44538 | T4742_S00003.810 | ATP-binding cassette sub-family B member 5 |
| OPB39231 | T4742_S00006.291 | ATP-binding protein |
| OPB41671 | T4742_S00011.514 | ATP-dependent Clp protease |
| OPB44900 | T4742_S00013.205 | ATP-dependent Clp protease proteolytic subunit |

| | | |
|---|---|---|
| OPB36381 | T4742_S00007.98 | ATP-dependent DNA helicase |
| OPB36855 | T4742_S00007.573 | ATP-dependent DNA ligase |
| OPB43139 | T4742_S00012.376 | ATP-dependent helicase Prp16 |
| OPB41101 | T4742_S00020.125 | ATP-dependent helicase, DEAD-box |
| OPB38256 | T4742_S00002.303 | ATP-dependent protease La |
| OPB38347 | T4742_S00002.395 | ATP-dependent protease La, putative |
| OPB39259 | T4742_S00006.319 | ATP-dependent RNA helicase |
| OPB38293 | T4742_S00002.340 | ATP-dependent RNA helicase DBP10 |
| OPB41798 | T4742_S00018.121 | ATP-dependent RNA helicase DBP3 |
| OPB45383 | T4742_S00010.294 | ATP-dependent RNA helicase DBP4 , putative |
| OPB44112 | T4742_S00003.383 | ATP-dependent RNA helicase DBP5 |
| OPB38381 | T4742_S00002.429 | ATP-dependent RNA helicase DBP7, putative |
| OPB42345 | T4742_S00004.447 | ATP-dependent RNA helicase DBP8 (EC 3.6.4.13) |
| OPB40889 | T4742_S00001.970 | ATP-dependent RNA helicase dbp9 |
| OPB40174 | T4742_S00001.253 | ATP-dependent RNA helicase ded-1 |
| OPB37516 | T4742_S00005.543 | ATP-dependent RNA helicase FAL1 |
| OPB39014 | T4742_S00006.73 | ATP-dependent RNA helicase FAL1 |
| OPB37579 | T4742_S00005.606 | ATP-dependent RNA helicase HAS1 |
| OPB40586 | T4742_S00001.666 | ATP-dependent RNA helicase MAK5, putative |
| OPB42253 | T4742_S00004.355 | ATP-dependent RNA helicase Mrh4 [Pochonia chlamydosporia 170] |
| OPB44298 | T4742_S00003.569 | ATP-dependent RNA helicase ROK1, putative |
| OPB37561 | T4742_S00005.588 | ATP-dependent RNA helicases. |
| OPB46757 | T4742_S00009.199 | ATP-NAD kinase |
| OPB44881 | T4742_S00013.186 | ATP-NAD/AcoX kinase |
| OPB36657 | T4742_S00007.375 | aureobasidin resistance protein Aur1 [Trichoderma guizhouense] |
| OPB44169 | T4742_S00003.440 | autophagy related, unknown protein |
| OPB44259 | T4742_S00003.530 | autophagy related, unknown protein |
| OPB45682 | T4742_S00014.16 | autophagy related, unknown protein |
| OPB46140 | T4742_S00008.207 | Auxiliary Activity Family 1 |
| OPB43226 | T4742_S00022.29 | AvaB protein [Aschersonia aleyrodis RCEF 2490] |
| OPB38895 | T4742_S00002.944 | bacterial (MHYT) signaling domain protein [Trichoderma guizhouense] |
| OPB44811 | T4742_S00013.116 | BAR |
| OPB42785 | T4742_S00012.22 | Barwin-related endoglucanase |
| OPB39979 | T4742_S00001.58 | Basic-leucine zipper (bZIP) transcription factor |
| OPB45379 | T4742_S00010.290 | Basic-leucine zipper (bZIP) transcription factor |
| OPB46969 | T4742_S00009.411 | BCAT_beta_family |

| | | |
|---|---|---|
| OPB43312 | T4742_S00019.45 | Bem1p, scaffold protein for complexes that include Cdc24p,Ste5p,Ste20p and Rsr1p |
| OPB42945 | T4742_S00012.182 | bem46 family protein |
| OPB45262 | T4742_S00010.171 | beta carbonic anhydrase |
| OPB43790 | T4742_S00003.61 | beta lactamase family protein |
| OPB40895 | T4742_S00001.976 | beta transducin-like protein HET-E2C [Podospora anserina] |
| OPB45950 | T4742_S00008.16 | Beta tubulin, autoregulation binding site |
| OPB40543 | T4742_S00001.622 | Beta-(1-6) glucan synthase [Trichoderma guizhouense] |
| OPB37000 | T4742_S00005.27 | Beta_HSD-beta hydroxysteroid dehydrogenase/isomerase family |
| OPB38338 | T4742_S00002.386 | beta-adaptin, apl1, large subunit of the adaptor protein complex (AP-2) of clathrin-coated vesicles |
| OPB36854 | T4742_S00007.572 | beta-adaptin, apl2, large subunit of the adaptor protein (AP-1) complex of clathrin-coated vesicles |
| OPB43297 | T4742_S00019.30 | Beta-adaptin, apl6, of the AP-3 adaptor protein complex of clathrin-coated vesicles, involved in transport to the vacuole |
| OPB38873 | T4742_S00002.922 | beta-alanine synthase |
| OPB36087 | T4742_S00021.3 | beta-lactamase |
| OPB41537 | T4742_S00011.380 | beta-lactamase |
| OPB41805 | T4742_S00018.128 | beta-lactamase |
| OPB42793 | T4742_S00012.30 | beta-lactamase |
| OPB43480 | T4742_S00019.214 | beta-lactamase |
| OPB38927 | T4742_S00002.976 | beta-lactamase family protein |
| OPB39662 | T4742_S00015.6 | beta-lactamase family protein |
| OPB40296 | T4742_S00001.375 | beta-lactamase family protein |
| OPB40390 | T4742_S00001.469 | beta-lactamase family protein |
| OPB40811 | T4742_S00001.892 | beta-lactamase family protein |
| OPB41868 | T4742_S00018.191 | beta-lactamase family protein |
| OPB42726 | T4742_S00004.832 | beta-lactamase family protein |
| OPB43750 | T4742_S00003.21 | beta-lactamase family protein |
| OPB44251 | T4742_S00003.522 | beta-lactamase family protein |
| OPB44715 | T4742_S00013.19 | beta-lactamase family protein |
| OPB44768 | T4742_S00013.73 | beta-lactamase family protein |
| OPB44850 | T4742_S00013.155 | beta-lactamase family protein |
| OPB45664 | T4742_S00010.575 | beta-lactamase family protein |
| OPB45974 | T4742_S00008.40 | beta-lactamase family protein |
| OPB44027 | T4742_S00003.298 | beta-lactamase family protein, putative glyoxalase |
| OPB42728 | T4742_S00004.834 | beta-lactamase protein |
| OPB44705 | T4742_S00013.9 | beta-lactamase superfamily protein [Fusarium oxysporum] |
| OPB46092 | T4742_S00008.159 | beta-subunit of phenylalanyl-tRNA synthetetase |
| OPB44457 | T4742_S00003.729 | bHLH regulator protein |

| | | |
|---|---|---|
| OPB36379 | T4742_S00007.96 | bHLH transcriptional regulator |
| OPB39566 | T4742_S00006.626 | bHLH transcriptional regulator |
| OPB40826 | T4742_S00001.907 | bHLH transcriptional regulator |
| OPB41504 | T4742_S00011.347 | bHLH transcriptional regulator |
| OPB42075 | T4742_S00004.177 | bHLH transcriptional regulator |
| OPB42403 | T4742_S00004.505 | bHLH transcriptional regulator |
| OPB43668 | T4742_S00016.188 | bHLH transcriptional regulator |
| OPB43726 | T4742_S00016.246 | bHLH transcriptional regulator |
| OPB41980 | T4742_S00004.82 | bHLH transcriptional regulator XPP1, binding to AGAA box |
| OPB40164 | T4742_S00001.243 | bicarbonate transporter |
| OPB40552 | T4742_S00001.631 | bifunctional catalase/peroxidase |
| OPB38002 | T4742_S00002.49 | Bifunctional P-450:NADPH-P450 reductase |
| OPB39029 | T4742_S00006.88 | BIM1, possibly involved in mating, karyogamy or meiosis |
| OPB41558 | T4742_S00011.401 | bimA |
| OPB46391 | T4742_S00008.458 | biotin synthase |
| OPB36620 | T4742_S00007.338 | biotin-protein ligase |
| OPB38158 | T4742_S00002.205 | blue light inducible protein BLI-3 |
| OPB44424 | T4742_S00003.696 | Blue light regulator BLR2 |
| OPB37744 | T4742_S00017.11 | Blue light regulator BRL1 |
| OPB36023 | T4742_S00023.58 | Branched chain alpha-keto acid dehydrogenase complex, alpha subunit |
| OPB43846 | T4742_S00003.117 | branched-chain amino acid aminotransferase [Colletotrichum fioriniae PJ7] |
| OPB40876 | T4742_S00001.957 | branched-chain amino transferase |
| OPB42352 | T4742_S00004.454 | branched-chain amino transferase |
| OPB47033 | T4742_S00009.475 | branched-chain amino transferase |
| OPB36377 | T4742_S00007.94 | BRCT domain-containing protein |
| OPB36031 | T4742_S00023.66 | Bromodomain transcription factor |
| OPB38364 | T4742_S00002.412 | Bromodomain transcription factor |
| OPB44459 | T4742_S00003.731 | Bromodomain transcription factor |
| OPB46765 | T4742_S00009.207 | Bromodomain transcription factor |
| OPB41359 | T4742_S00011.200 | BTB/POZ-like |
| OPB42609 | T4742_S00004.713 | BTB/POZ-like |
| OPB38806 | T4742_S00002.855 | Bys1 family protein [Trichoderma harzianum] |
| OPB40320 | T4742_S00001.399 | bZIP [Trichoderma guizhouense] |
| OPB36341 | T4742_S00007.58 | Bzip family transcription factor |
| OPB37498 | T4742_S00005.525 | Bzip family transcription factor |
| OPB37616 | T4742_S00005.643 | Bzip family transcription factor |

| | | |
|---|---|---|
| OPB38205 | T4742_S00002.252 | Bzip family transcription factor |
| OPB38509 | T4742_S00002.558 | Bzip family transcription factor |
| OPB39553 | T4742_S00006.613 | Bzip family transcription factor |
| OPB39704 | T4742_S00015.48 | Bzip family transcription factor |
| OPB40254 | T4742_S00001.333 | Bzip family transcription factor |
| OPB40481 | T4742_S00001.560 | Bzip family transcription factor |
| OPB41431 | T4742_S00011.274 | Bzip family transcription factor |
| OPB41948 | T4742_S00004.50 | Bzip family transcription factor |
| OPB42133 | T4742_S00004.235 | Bzip family transcription factor |
| OPB42371 | T4742_S00004.473 | Bzip family transcription factor |
| OPB43059 | T4742_S00012.296 | Bzip family transcription factor |
| OPB44570 | T4742_S00003.842 | Bzip family transcription factor |
| OPB45438 | T4742_S00010.349 | Bzip family transcription factor |
| OPB47084 | T4742_S00009.526 | Bzip family transcription factor |
| OPB39535 | T4742_S00006.595 | C1 tetrahydrofolate synthase, putative |
| OPB39110 | T4742_S00006.170 | C-14 sterol reductase ERG24 |
| OPB42921 | T4742_S00012.158 | C2H2 conidiation transcription factor FlbC |
| OPB38607 | T4742_S00002.656 | C2H2 regulator with ankyrin repeat |
| OPB36160 | T4742_S00021.77 | C2H2 transcription factor |
| OPB38874 | T4742_S00002.923 | C2H2 transcription factor |
| OPB40878 | T4742_S00001.959 | C2H2 transcription factor |
| OPB43336 | T4742_S00019.69 | C2H2 transcription factor |
| OPB43619 | T4742_S00016.139 | C2H2 transcription factor |
| OPB43640 | T4742_S00016.160 | C2H2 transcription factor |
| OPB46291 | T4742_S00008.358 | C2H2 transcription factor |
| OPB46428 | T4742_S00008.495 | C2H2 transcription factor |
| OPB41100 | T4742_S00020.124 | C2H2 transcription factor (Con7) |
| OPB45851 | T4742_S00014.186 | C2H2 transcription factor [Aspergillus arachidicola] |
| OPB41794 | T4742_S00018.117 | C2H2 transcription factor/early growth response protein [Trichoderma guizhouense] |
| OPB36287 | T4742_S00007.4 | C2H2 transcriptional regulator |
| OPB36289 | T4742_S00007.6 | C2H2 transcriptional regulator |
| OPB36352 | T4742_S00007.69 | C2H2 transcriptional regulator |
| OPB36411 | T4742_S00007.128 | C2H2 transcriptional regulator |
| OPB36814 | T4742_S00007.532 | C2H2 transcriptional regulator |
| OPB36860 | T4742_S00007.578 | C2H2 transcriptional regulator |
| OPB37125 | T4742_S00005.152 | C2H2 transcriptional regulator |

| | | |
|---|---|---|
| OPB37230 | T4742_S00005.257 | C2H2 transcriptional regulator |
| OPB37660 | T4742_S00005.687 | C2H2 transcriptional regulator |
| OPB37738 | T4742_S00017.5 | C2H2 transcriptional regulator |
| OPB37757 | T4742_S00017.24 | C2H2 transcriptional regulator |
| OPB38074 | T4742_S00002.121 | C2H2 transcriptional regulator |
| OPB38121 | T4742_S00002.168 | C2H2 transcriptional regulator |
| OPB38156 | T4742_S00002.203 | C2H2 transcriptional regulator |
| OPB38298 | T4742_S00002.345 | C2H2 transcriptional regulator |
| OPB38441 | T4742_S00002.489 | C2H2 transcriptional regulator |
| OPB38688 | T4742_S00002.737 | C2H2 transcriptional regulator |
| OPB38799 | T4742_S00002.848 | C2H2 transcriptional regulator |
| OPB38834 | T4742_S00002.883 | C2H2 transcriptional regulator |
| OPB38973 | T4742_S00006.32 | C2H2 transcriptional regulator |
| OPB39429 | T4742_S00006.489 | C2H2 transcriptional regulator |
| OPB39563 | T4742_S00006.623 | C2H2 transcriptional regulator |
| OPB39573 | T4742_S00006.633 | C2H2 transcriptional regulator |
| OPB39605 | T4742_S00006.665 | C2H2 transcriptional regulator |
| OPB39782 | T4742_S00015.126 | C2H2 transcriptional regulator |
| OPB40145 | T4742_S00001.224 | C2H2 transcriptional regulator |
| OPB40501 | T4742_S00001.580 | C2H2 transcriptional regulator |
| OPB40602 | T4742_S00001.682 | C2H2 transcriptional regulator |
| OPB40713 | T4742_S00001.793 | C2H2 transcriptional regulator |
| OPB41025 | T4742_S00020.49 | C2H2 transcriptional regulator |
| OPB41829 | T4742_S00018.152 | C2H2 transcriptional regulator |
| OPB42165 | T4742_S00004.267 | C2H2 transcriptional regulator |
| OPB42574 | T4742_S00004.677 | C2H2 transcriptional regulator |
| OPB42575 | T4742_S00004.678 | C2H2 transcriptional regulator |
| OPB42682 | T4742_S00004.788 | C2H2 transcriptional regulator |
| OPB42944 | T4742_S00012.181 | C2H2 transcriptional regulator |
| OPB43040 | T4742_S00012.277 | C2H2 transcriptional regulator |
| OPB43096 | T4742_S00012.333 | C2H2 transcriptional regulator |
| OPB43265 | T4742_S00022.69 | C2H2 transcriptional regulator |
| OPB43932 | T4742_S00003.203 | C2H2 transcriptional regulator |
| OPB43943 | T4742_S00003.214 | C2H2 transcriptional regulator |
| OPB44273 | T4742_S00003.544 | C2H2 transcriptional regulator |
| OPB44956 | T4742_S00013.261 | C2H2 transcriptional regulator |

| | | |
|---|---|---|
| OPB45295 | T4742_S00010.206 | C2H2 transcriptional regulator |
| OPB45349 | T4742_S00010.260 | C2H2 transcriptional regulator |
| OPB45677 | T4742_S00014.11 | C2H2 transcriptional regulator |
| OPB45944 | T4742_S00008.10 | C2H2 transcriptional regulator |
| OPB45959 | T4742_S00008.25 | C2H2 transcriptional regulator |
| OPB45991 | T4742_S00008.57 | C2H2 transcriptional regulator |
| OPB46882 | T4742_S00009.324 | C2H2 transcriptional regulator |
| OPB46908 | T4742_S00009.350 | C2H2 transcriptional regulator |
| OPB47102 | T4742_S00009.544 | C2H2 transcriptional regulator |
| OPB41207 | T4742_S00011.47 | C2H2 transcriptional regulator (amdA ?) |
| OPB46556 | T4742_S00008.623 | C2H2 transcriptional regulator (amdA ?) |
| OPB38360 | T4742_S00002.408 | C2H2 transcriptional regulator CRE1 |
| OPB38986 | T4742_S00006.45 | C2H2 transcriptional regulator, SEB1, responds toosmotic stress |
| OPB37126 | T4742_S00005.153 | C2H2 type zinc finger domain protein |
| OPB46361 | T4742_S00008.428 | C2H2 type zinc finger domain protein |
| OPB42224 | T4742_S00004.326 | C2H2 type zinc finger domain protein [Metarhizium anisopliae] |
| OPB36142 | T4742_S00021.59 | C2H2 type zinc finger domain-containing protein [Colletotrichum gloeosporioides Nara gc5] |
| OPB42353 | T4742_S00004.455 | C2H2 zinc finger contain protein |
| OPB40372 | T4742_S00001.451 | C2HC5 finger protein |
| OPB41691 | T4742_S00018.13 | C2HC-type zinc finger protein |
| OPB44865 | T4742_S00013.170 | C2HC-type zinc finger protein |
| OPB40282 | T4742_S00001.361 | C-4 sterol methyl oxidase |
| OPB38211 | T4742_S00002.258 | C4-dicarboxylate transporter/malic acid transport protein |
| OPB40338 | T4742_S00001.417 | C4-dicarboxylate transporter/malic acid transport protein |
| OPB42399 | T4742_S00004.501 | C4-dicarboxylate transporter/malic acid transport protein |
| OPB36747 | T4742_S00007.465 | C-5 cytosine-specific DNA methylase |
| OPB46622 | T4742_S00009.64 | C-5 sterol desaturase |
| OPB40075 | T4742_S00001.154 | C6 transcription factor [Aspergillus fumigatus Af293] |
| OPB43175 | T4742_S00012.412 | C6HC zinc finger protein |
| OPB43176 | T4742_S00012.413 | C6HC zinc finger protein |
| OPB38566 | T4742_S00002.615 | Ca2+ channel |
| OPB43497 | T4742_S00016.17 | Ca2+ permeable channel, related to N. crassa NCU02762.1 |
| OPB42519 | T4742_S00004.621 | Ca2+ permeable channel, related to N. crassa NCU07605.1 |
| OPB36315 | T4742_S00007.32 | Ca2+ transporter |
| OPB38289 | T4742_S00002.336 | Ca2+ transporter |
| OPB39677 | T4742_S00015.21 | Ca2+ transporter |

| | | |
|---|---|---|
| OPB45089 | T4742_S00013.394 | Ca2+ transporter, putative |
| OPB36036 | T4742_S00023.71 | Ca2+-binding actin-bundling protein (fimbrin/plastin), EF-Hand protein superfamily |
| OPB39454 | T4742_S00006.514 | Ca-ATPases |
| OPB46604 | T4742_S00009.46 | CaaX-protease, related to E. nidulans rce1, involved in signal transduction |
| OPB42952 | T4742_S00012.189 | cadmium resistance transporter |
| OPB40447 | T4742_S00001.526 | caffeine-induced death protein 2 |
| OPB36267 | T4742_S00021.186 | CAIB/BAIF family enzyme |
| OPB39999 | T4742_S00001.78 | Calcineurin B subunit, Protein phosphatase 2B subunit |
| OPB37418 | T4742_S00005.445 | calcineurin catalytic subunit |
| OPB38928 | T4742_S00002.977 | calcineurin-like phosphoesterase |
| OPB41550 | T4742_S00011.393 | calcineurin-like phosphoesterase |
| OPB45123 | T4742_S00010.30 | calcineurin-like phosphoesterase [Beauveria bassiana ARSEF 2860] |
| OPB39764 | T4742_S00015.108 | calcipressin |
| OPB42001 | T4742_S00004.103 | calcium binding EF-hand protein |
| OPB43241 | T4742_S00022.44 | calcium dependent mitochondrial carrier protein, putative |
| OPB46972 | T4742_S00009.414 | Calcium ion pump, ATPase |
| OPB38080 | T4742_S00002.127 | calcium sensor NCS1, regulates sporulation and confers calcium tolerance |
| OPB37973 | T4742_S00002.20 | Calcium transporter |
| OPB39684 | T4742_S00015.28 | Calcium transporter |
| OPB42995 | T4742_S00012.232 | Calcium transporter |
| OPB41200 | T4742_S00011.40 | calcium transporting ATPase, ion pump |
| OPB44684 | T4742_S00003.956 | calcium transporting ATPase, ion pump |
| OPB45994 | T4742_S00008.60 | Calcium/calmodulin dependent protein kinase |
| OPB36656 | T4742_S00007.374 | calcium/calmodulin dependent protein kinase 2 |
| OPB37771 | T4742_S00017.38 | calcium/calmodulin-dependent protein kinase |
| OPB38978 | T4742_S00006.37 | Calcium/calmodulin-dependent protein kinase |
| OPB44438 | T4742_S00003.710 | calcium/sodium antiporter, contains 9 transmembrane domains |
| OPB39254 | T4742_S00006.314 | Calcium-binding EF-hand |
| OPB40045 | T4742_S00001.124 | Calcium-binding EF-hand |
| OPB37130 | T4742_S00005.157 | calcium-spray protein |
| OPB45055 | T4742_S00013.360 | calmodulin is involved in control of enzymes by Ca(2+), stimulates many protein kinases and phosphatases and contains 4 EF-hand calcium binding domains |
| OPB42154 | T4742_S00004.256 | calnexin, high identity with A. niger clxA |
| OPB39123 | T4742_S00006.183 | calpain-like protease |
| OPB41692 | T4742_S00018.14 | calpain-like protease |
| OPB45946 | T4742_S00008.12 | Calpain-like protease palB/rim-13, putative |
| OPB42279 | T4742_S00004.381 | calponin-like actin binding domain |

| | | |
|---|---|---|
| OPB38655 | T4742_S00002.704 | CAMK kinase |
| OPB42230 | T4742_S00004.332 | CAMK type kinase, related to N. crassa CAMK-3 |
| OPB39368 | T4742_S00006.428 | cAMP dependent protein kinase, protein kinase A, catalytic subunit |
| OPB39525 | T4742_S00006.585 | cAMP dependent protein kinase, protein kinase A, regulatory subunit |
| OPB39244 | T4742_S00006.304 | CAMP kinase |
| OPB39024 | T4742_S00006.83 | cAMP phosphodiesterase class II PDE1, low affinity |
| OPB42207 | T4742_S00004.309 | cAMP phosphodiesterase PDE2, high affinity |
| OPB42663 | T4742_S00004.768 | CAMP type kinase |
| OPB42396 | T4742_S00004.498 | cAMP-mediated signaling protein SOK1 |
| OPB39467 | T4742_S00006.527 | cAMP-regulated phosphoprotein/endosulphine conserved region |
| OPB45453 | T4742_S00010.364 | candidate a-glycosidase related to a-1,2-L-fucosidase |
| OPB42188 | T4742_S00004.290 | CAP20 virulence factor |
| OPB39975 | T4742_S00001.54 | Ca-permeable channel, related to A. nidulans Mid2 |
| OPB39237 | T4742_S00006.297 | CAP-Gly domain aligned |
| OPB44010 | T4742_S00003.281 | capsule polysaccharide biosynthesis protein |
| OPB44009 | T4742_S00003.280 | capsule polysaccharide biosynthesis protein, putative |
| OPB46547 | T4742_S00008.614 | carbamoyl phosphate synthase; putative |
| OPB37016 | T4742_S00005.43 | carbamoyl-phosphate synthase (glutamine-hydrolyzing)arginine-specific large chain |
| OPB38059 | T4742_S00002.106 | Carbamoyl-phosphate synthase, small chain |
| OPB38177 | T4742_S00002.224 | carbamoylphosphate synthase/aspartate carbamoyltransferase |
| OPB40948 | T4742_S00001.1029 | carbohydrate binding module |
| OPB45816 | T4742_S00014.151 | carbohydrate esterase family 1 protein |
| OPB46088 | T4742_S00008.155 | Carbohydrate/puine kinase, PfkB, conserved site |
| OPB41261 | T4742_S00011.102 | Carbohydrate-Binding Module Family 13 |
| OPB40458 | T4742_S00001.537 | carbohydrate-binding module family 13 protein |
| OPB43751 | T4742_S00003.22 | Carbohydrate-binding WSC |
| OPB44579 | T4742_S00003.851 | Carbohydrate-binding WSC |
| OPB46803 | T4742_S00009.245 | Carbohydrate-binding WSC |
| OPB44816 | T4742_S00013.121 | Carbonic anhydrase |
| OPB45733 | T4742_S00014.68 | Carbonic anhydrase |
| OPB46983 | T4742_S00009.425 | Carbonic anhydrase |
| OPB40392 | T4742_S00001.471 | carbonic anhydrase |
| OPB39473 | T4742_S00006.533 | carbon-nitrogen hydrolase |
| OPB41983 | T4742_S00004.85 | Carbon-nitrogen hydrolase |
| OPB41238 | T4742_S00011.79 | Carbon-nitrogen hydrolase [Trichoderma guizhouense] |
| OPB42012 | T4742_S00004.114 | carboxy-cis,cis-muconate cyclase |

| | | |
|---|---|---|
| OPB36127 | T4742_S00021.44 | carboxyl esterase |
| OPB36789 | T4742_S00007.507 | carboxyl esterase |
| OPB37940 | T4742_S00017.207 | carboxylesterase |
| OPB44533 | T4742_S00003.805 | carboxylesterase |
| OPB38887 | T4742_S00002.936 | carboxylesterase family protein |
| OPB42192 | T4742_S00004.294 | carboxylesterase family protein |
| OPB46453 | T4742_S00008.520 | carboxylesterase family protein |
| OPB45931 | T4742_S00014.268 | carboxylesterase type B |
| OPB46070 | T4742_S00008.137 | carboxylesterase type b |
| OPB38903 | T4742_S00002.952 | Carboxylesterase, type B |
| OPB42713 | T4742_S00004.819 | Carboxylic acid reductase [Tolypocladium ophioglossoides CBS 100239] |
| OPB37524 | T4742_S00005.551 | carboxylic acid transporter |
| OPB42889 | T4742_S00012.126 | carboxylyase/3-octaprenyl-4-hydroxybenzoate carboxylyase |
| OPB37182 | T4742_S00005.209 | carboxymethylenebutenolidase |
| OPB41209 | T4742_S00011.49 | Carboxymuconolactone decarboxylase |
| OPB39490 | T4742_S00006.550 | carboxymuconolactone decarboxylase, putative |
| OPB44490 | T4742_S00003.762 | carboxypeptidase A |
| OPB41321 | T4742_S00011.162 | carboxypeptidase Y |
| OPB38955 | T4742_S00006.14 | Carboxypeptidase Y homolog |
| OPB41356 | T4742_S00011.197 | carboxyphosphonoenolpyruvate mutase |
| OPB42700 | T4742_S00004.806 | carboxyphosphonoenolpyruvate phosphonomutase-like protein |
| OPB46825 | T4742_S00009.267 | Cargo transport protein Emp24 (p24 protein family) |
| OPB42092 | T4742_S00004.194 | Cargo transport protein Erv29 |
| OPB37967 | T4742_S00002.14 | Carn_acyltransfCholine/Carnitine o-acyltransferase |
| OPB39844 | T4742_S00015.188 | Carnitine o-acyltransferase |
| OPB43462 | T4742_S00019.195 | Carnitinyl-CoA dehydratase [Fusarium oxysporum f. sp. cubense race 4] |
| OPB40737 | T4742_S00001.817 | carnosin synthase 1 |
| OPB42118 | T4742_S00004.220 | Carnosine synthase [Aspergillus fumigatus Z5] |
| OPB46202 | T4742_S00008.269 | carotenoid synthesis regulator CarF |
| OPB37444 | T4742_S00005.471 | Casein kinase 1Cki1, regulator of phosphatidylinositol 4-phosphate 5-kinase |
| OPB41655 | T4742_S00011.498 | casein kinase I homolog hhp1 |
| OPB44261 | T4742_S00003.532 | casein kinase II, alpha subunit |
| OPB36553 | T4742_S00007.270 | casein kinase II, beta (regulatory) subunit |
| OPB39584 | T4742_S00006.644 | casein kinase II, beta subunit, regulatory |
| OPB39159 | T4742_S00006.219 | Catalase |
| OPB40299 | T4742_S00001.378 | Catalase |

| | | |
|---|---|---|
| OPB42210 | T4742_S00004.312 | Catalase |
| OPB45111 | T4742_S00010.18 | catalase |
| OPB47062 | T4742_S00009.504 | Catalase |
| OPB37380 | T4742_S00005.407 | catalase CAT6 |
| OPB37636 | T4742_S00005.663 | catalase protein |
| OPB40739 | T4742_S00001.820 | Catalase, N-terminal |
| OPB44593 | T4742_S00003.865 | catechol dioxigenase |
| OPB42011 | T4742_S00004.113 | catechol dioxygenase |
| OPB39522 | T4742_S00006.582 | catechol O-methyltransferase |
| OPB38475 | T4742_S00002.523 | cation channel family protein |
| OPB37844 | T4742_S00017.111 | cation diffusion facilitator family transporter |
| OPB39928 | T4742_S00001.7 | cation diffusion facilitator family transporter |
| OPB38153 | T4742_S00002.200 | cation efflux family protein |
| OPB43713 | T4742_S00016.233 | cation efflux family protein |
| OPB45198 | T4742_S00010.106 | cation efflux family protein, putative |
| OPB45047 | T4742_S00013.352 | Cation efflux protein |
| OPB40370 | T4742_S00001.449 | cation pump, Calcium transport |
| OPB39253 | T4742_S00006.313 | cation transport protein ChaC, putative |
| OPB40393 | T4742_S00001.472 | cation transporting ATPase |
| OPB41717 | T4742_S00018.40 | cation transporting ATPase |
| OPB45296 | T4742_S00010.207 | Cation transporting ATPase |
| OPB45469 | T4742_S00010.380 | Cation transporting ATPase |
| OPB39295 | T4742_S00006.355 | CBM 13 |
| OPB45388 | T4742_S00010.299 | CBM 13 |
| OPB42845 | T4742_S00012.82 | CBM 21 |
| OPB39836 | T4742_S00015.180 | CBM13 protein [Trichoderma guizhouense] |
| OPB43656 | T4742_S00016.176 | CBM48 |
| OPB45518 | T4742_S00010.429 | CBM50, LysM |
| OPB40505 | T4742_S00001.584 | CBM50/LysM |
| OPB41063 | T4742_S00020.87 | CBM50/LysM |
| OPB38232 | T4742_S00002.279 | CBM50/LysM domain protein |
| OPB45519 | T4742_S00010.430 | CBM50/LysM domain-containing protein [Colletotrichum gloeosporioides Nara gc5] |
| OPB42929 | T4742_S00012.166 | CCAAT-binding transcription factor, subunit B |
| OPB37112 | T4742_S00005.139 | CCCH transcriptional regulator |
| OPB41589 | T4742_S00011.432 | CCCH transcriptional regulator |
| OPB46588 | T4742_S00009.28 | CCCH transcriptional regulator |

| | | |
|---|---|---|
| OPB41820 | T4742_S00018.143 | CCD1 |
| OPB37437 | T4742_S00005.464 | CCHC transcriptional regulator |
| OPB40252 | T4742_S00001.331 | CCHC zinc finger protein |
| OPB41903 | T4742_S00004.5 | CCR4-NOT core complex subunit Not4 |
| OPB36523 | T4742_S00007.240 | Ccr4-Not transcription complex subunit |
| OPB38291 | T4742_S00002.338 | Cd2+/Zn2+ transporter protein |
| OPB45537 | T4742_S00010.448 | Cdc15/Fes/CIP4 |
| OPB44275 | T4742_S00003.546 | CDC20, SLP1 |
| OPB46433 | T4742_S00008.500 | CDC24, rho-family GTPase |
| OPB36517 | T4742_S00007.234 | CDC28, Catalytic subunit of the main cell cycle cyclin-dependent kinase (CDK), |
| OPB36537 | T4742_S00007.254 | CDC31, putative spindle pole body component |
| OPB41496 | T4742_S00011.339 | cdc42 |
| OPB38135 | T4742_S00002.182 | CDC45-like protein |
| OPB41500 | T4742_S00011.343 | cdc9p ligase |
| OPB44150 | T4742_S00003.421 | Cdk-activating kinase assembly factor (MAT 1) |
| OPB42383 | T4742_S00004.485 | CDP-alcohol phosphatidyltransferase |
| OPB38510 | T4742_S00002.559 | CDP-alcohol phosphatidyltransferase |
| OPB44210 | T4742_S00003.481 | CDP-alcohol phosphatidyltransferase |
| OPB46569 | T4742_S00009.9 | CDP-alcohol phosphatidyltransferase |
| OPB45048 | T4742_S00013.353 | CE1 alpha/beta hydrolase |
| OPB37536 | T4742_S00005.563 | CE1 esterase |
| OPB46658 | T4742_S00009.100 | CE1 esterase |
| OPB41260 | T4742_S00011.101 | CE1 esterase (PHB?) |
| OPB43043 | T4742_S00012.280 | CE1 Esterase/lipase/thioesterase |
| OPB46102 | T4742_S00008.169 | CE1 esterase/lipase/thioesterase |
| OPB36332 | T4742_S00007.49 | CE1 protein |
| OPB40171 | T4742_S00001.250 | CE1 protein |
| OPB38726 | T4742_S00002.775 | CE1 α/ß hydrolase lipase/epoxide hydrolase |
| OPB36002 | T4742_S00023.37 | CE10 Arylacetamide deacetylase |
| OPB36430 | T4742_S00007.147 | CE10 Arylacetamide deacetylase |
| OPB39711 | T4742_S00015.55 | CE10 carbohydrate esterease |
| OPB39667 | T4742_S00015.11 | CE10 carboxylesterase type B |
| OPB38614 | T4742_S00002.663 | CE10 esterase/lipase |
| OPB39190 | T4742_S00006.250 | CE10 esterase/lipase/thioesterase |
| OPB45685 | T4742_S00014.19 | CE10 Esterase/lipase/thioesterase |
| OPB36337 | T4742_S00007.54 | CE10 Esterase/lipase/thioesterase superfamily |

| | | |
|---|---|---|
| OPB38047 | T4742_S00002.94 | CE10 lipase/esterase |
| OPB41055 | T4742_S00020.79 | CE10 protein |
| OPB41561 | T4742_S00011.404 | CE10 protein |
| OPB37879 | T4742_S00017.146 | CE10 prpotein |
| OPB46149 | T4742_S00008.216 | CE12 GDSL lipase |
| OPB39357 | T4742_S00006.417 | CE12 protein |
| OPB38179 | T4742_S00002.226 | CE16 acetyl esterase |
| OPB45856 | T4742_S00014.191 | CE1-feruloyl esterase |
| OPB38703 | T4742_S00002.752 | CE3 acetyl xylan esterase |
| OPB46178 | T4742_S00008.245 | CE3 acetyl xylan esterase |
| OPB43347 | T4742_S00019.80 | CE3 protein [Trichoderma guizhouense] |
| OPB45589 | T4742_S00010.500 | CE4 chitin deacetylase |
| OPB46551 | T4742_S00008.618 | CE4 imidase |
| OPB39263 | T4742_S00006.323 | CE4 polysaccharide deacetylase |
| OPB40352 | T4742_S00001.431 | CE5 acetyl xylan esterase AXE1 |
| OPB42582 | T4742_S00004.685 | CE5 acetyl xylan esterase AXE2 |
| OPB40798 | T4742_S00001.879 | CE5 cutinase |
| OPB44214 | T4742_S00003.485 | CE7 Alpha/beta hydrolase |
| OPB45283 | T4742_S00010.194 | CE7 protein |
| OPB40302 | T4742_S00001.381 | cell cycle control protein (Cwf19), putative |
| OPB42225 | T4742_S00004.327 | cell cycle control protein cwf14 |
| OPB40997 | T4742_S00020.21 | cell cycle control protein cwf8 |
| OPB45069 | T4742_S00013.374 | cell cycle regulated protein, WD and fizzy domains |
| OPB41950 | T4742_S00004.52 | cell differentiation family protein (rcd1) |
| OPB38027 | T4742_S00002.74 | cell division control protein 3 |
| OPB43153 | T4742_S00012.390 | cell division control protein CDC10 septin |
| OPB44124 | T4742_S00003.395 | cell division control protein cdc14, putative |
| OPB40532 | T4742_S00001.611 | cell division control protein Cdc4, putative |
| OPB46807 | T4742_S00009.249 | cell division control protein Cdc6 |
| OPB42312 | T4742_S00004.414 | cell division cycle 37 protein, CDC37 |
| OPB45155 | T4742_S00010.62 | cell division cycle protein 123 , putative |
| OPB37430 | T4742_S00005.457 | cell division cycle protein 23, putative |
| OPB38515 | T4742_S00002.564 | Cell division/GTP binding protein |
| OPB40008 | T4742_S00001.87 | Cell division/GTP binding protein |
| OPB38467 | T4742_S00002.515 | cell lysis protein-like protein [Trichoderma guizhouense] |
| OPB38271 | T4742_S00002.318 | cell morphogenesis protein PAG1 |

| | | |
|---|---|---|
| OPB36294 | T4742_S00007.11 | cell polarity protein (Tea1) |
| OPB37265 | T4742_S00005.292 | cell wall biogenesis protein phosphatase Ssd1, putative |
| OPB39282 | T4742_S00006.342 | cell wall mannoprotein |
| OPB41256 | T4742_S00011.97 | cell wall organization protein/glutathione transferase (Gto3) |
| OPB38610 | T4742_S00002.659 | cell wall protein (Metarhizium adhesion Mad1 ?) |
| OPB41959 | T4742_S00004.61 | cell wall protein, CwpA |
| OPB47032 | T4742_S00009.474 | cell wall protein, distantly related to A. niger CwpA. |
| OPB40510 | T4742_S00001.589 | cell wall protein, istantly related to S. cerevisiae Pir3p. |
| OPB37867 | T4742_S00017.134 | cell wall protein. Ser/Thr-rich. |
| OPB41413 | T4742_S00011.255 | cell wall protein. Ser/Thr-rich. |
| OPB42171 | T4742_S00004.273 | cellobiose dehydrogenase/ |
| OPB42920 | T4742_S00012.157 | cellular morphogenesis protein, putative |
| OPB42964 | T4742_S00012.201 | cellular morphogenesis regulator DopA, putative |
| OPB43995 | T4742_S00003.266 | cellular nucleic acid binding protein |
| OPB40290 | T4742_S00001.369 | cellulose binding protein |
| OPB41202 | T4742_S00011.42 | Cellulose-binding region, fungal |
| OPB43780 | T4742_S00003.51 | Cellulose-binding region, fungal |
| OPB44732 | T4742_S00013.37 | Cellulose-binding region, fungal |
| OPB45635 | T4742_S00010.546 | Cellulose-binding region, fungal |
| OPB39376 | T4742_S00006.436 | CENP-B, kinetochore assembly |
| OPB36744 | T4742_S00007.462 | centromere binding and kinetochore assembly |
| OPB39646 | T4742_S00006.706 | centromere binding and kinetochore assembly |
| OPB40105 | T4742_S00001.184 | centromere/microtubule binding protein CBF5 |
| OPB36382 | T4742_S00007.99 | Ceramidase family protein, associated toCellulase signal transduction (PMID: 15288024) |
| OPB40243 | T4742_S00001.322 | ceramidase, nonlysosomal |
| OPB45164 | T4742_S00010.71 | ceramide synthase membrane component Lag1 |
| OPB45524 | T4742_S00010.435 | ceratoplatanin Epl1/Sm1 |
| OPB41614 | T4742_S00011.457 | CFEM domain membrane protein |
| OPB42186 | T4742_S00004.288 | CFEM domain membrane protein |
| OPB46904 | T4742_S00009.346 | CFEM domain membrane protein |
| OPB46506 | T4742_S00008.573 | CFEM domain protein |
| OPB45667 | T4742_S00010.578 | CFEM domain-containing protein [Colletotrichum tofieldiae] |
| OPB40450 | T4742_S00001.529 | CFEM membrane protein |
| OPB40231 | T4742_S00001.310 | CFEM-domain protein |
| OPB44833 | T4742_S00013.138 | Chalcone isomerase-like |
| OPB38295 | T4742_S00002.342 | chaperone DnaJ |

| | | |
|---|---|---|
| OPB40968 | T4742_S00001.1049 | chaperone protein dnaJ 2, HSP40 family |
| OPB36318 | T4742_S00007.35 | chaperone protein dnaJ 6 |
| OPB46115 | T4742_S00008.182 | chaperone protein HSP31 [Cladophialophora carrionii] |
| OPB38666 | T4742_S00002.715 | Chaperonin Cpn10/HSP10 |
| OPB43382 | T4742_S00019.115 | chaperonine Cpn60/TCP1, t-complex protein t, subunit ß |
| OPB36621 | T4742_S00007.339 | CHCH domain-containing protein [Metarhizium guizhouense ARSEF 977] |
| OPB46232 | T4742_S00008.299 | CHD5 domain-containing protein |
| OPB43003 | T4742_S00012.240 | checkpoint kinase 2-like protein, RAD53 |
| OPB39613 | T4742_S00006.673 | checkpoint serine/threonine-protein kinase BUB1, putative |
| OPB39614 | T4742_S00006.674 | checkpoint serine/threonine-protein kinase BUB1, putative |
| OPB38681 | T4742_S00002.730 | chitin biosynthesis protein CHS5 |
| OPB39446 | T4742_S00006.506 | Chitin synthase |
| OPB39445 | T4742_S00006.505 | chitin synthase activator, putative |
| OPB40864 | T4742_S00001.945 | Chitin synthase export chaperone |
| OPB39879 | T4742_S00015.223 | Chitin synthase, homolog of N.CrassaChs2,Chitin synthase |
| OPB41737 | T4742_S00018.60 | chitinase 18-13 |
| OPB39167 | T4742_S00006.227 | Chitin-binding, type 1 |
| OPB44911 | T4742_S00013.216 | chloroperoxidase |
| OPB46006 | T4742_S00008.72 | chloroperoxidase |
| OPB47100 | T4742_S00009.542 | chloroperoxidase |
| OPB45289 | T4742_S00010.200 | choline kinase , putative |
| OPB43845 | T4742_S00003.116 | Choline phosphate cytidylyltransferase/Predicted CDP-ethanolamine synthase |
| OPB43620 | T4742_S00016.140 | choline-phosphate cytidylyltransferase |
| OPB36152 | T4742_S00021.69 | cholinesterase |
| OPB38127 | T4742_S00002.174 | Chorismate mutase |
| OPB39621 | T4742_S00006.681 | Chorismate_syntChorismate synthase |
| OPB42336 | T4742_S00004.438 | chromate transporter |
| OPB43173 | T4742_S00012.410 | chromate transporter |
| OPB38835 | T4742_S00002.884 | Chromate transporter [Penicillium occitanis] |
| OPB44675 | T4742_S00003.947 | chromatin (transcription) elongation factor spt5. |
| OPB40180 | T4742_S00001.259 | chromatin assembly factor 1 subunit C |
| OPB46674 | T4742_S00009.116 | chromatin assembly protein, putative |
| OPB45192 | T4742_S00010.100 | chromatin modification-like protein VID21 [Fusarium oxysporum f. sp. conglutinans race 2 54008] |
| OPB43158 | T4742_S00012.395 | chromatin related sumoylation, UBA2 |
| OPB43485 | T4742_S00016.5 | chromatin remodelling factors, contains a SWIRM domain and a DNA-binding Myb-domain |
| OPB43050 | T4742_S00012.287 | chromatin remodelling subunit ARP8 |

| | | |
|---|---|---|
| OPB37746 | T4742_S00017.13 | chromatin structure-remodeling complex protein RSC1, putative |
| OPB36992 | T4742_S00005.19 | chromatin structure-remodeling complex protein, putative |
| OPB39627 | T4742_S00006.687 | chromatin-binding domain proteins |
| OPB43215 | T4742_S00022.18 | chromo domain-containing protein [Ophiocordyceps sinensis CO18] |
| OPB37145 | T4742_S00005.172 | chromodomain-helicase-DNA-binding protein |
| OPB40944 | T4742_S00001.1025 | chromosome condensation protein (CrcB), putative |
| OPB46630 | T4742_S00009.72 | Chromosome condensation protein 3, C-terminal |
| OPB42003 | T4742_S00004.105 | Chromosome segregation protein |
| OPB39723 | T4742_S00015.67 | chy and ring finger domain-containing protein [Colletotrichum gloeosporioides Nara gc5] |
| OPB38283 | T4742_S00002.330 | CinA Predicted nucleotide-utilizing enzyme related to molybdopterin-biosynthesis enzyme MoeA |
| OPB43696 | T4742_S00016.216 | cinnamoyl-CoA reductase |
| OPB44819 | T4742_S00013.124 | cinnamoyl-CoA reductase |
| OPB41189 | T4742_S00011.29 | CIP1 |
| OPB43793 | T4742_S00003.64 | CipA (Aspergillus) oxidoreductase |
| OPB39734 | T4742_S00015.78 | citrate (Si)-synthase |
| OPB39251 | T4742_S00006.311 | citrate lyase, beta subunit |
| OPB45457 | T4742_S00010.368 | class I glutamine amidotransferase, putative |
| OPB43819 | T4742_S00003.90 | class I peptide chain release factor. |
| OPB37765 | T4742_S00017.32 | Clathrin associated epsin 2A |
| OPB41396 | T4742_S00011.238 | clathrin heavy chain, chc1, vesicle coat protein |
| OPB45051 | T4742_S00013.356 | Clathrin lightChain,Clc1, vesicleCoat protein |
| OPB42201 | T4742_S00004.303 | cleavage and polyadenylation specificity factor subunit 5 |
| OPB36761 | T4742_S00007.479 | cleavage and polyadenylylation specificity factor, putative |
| OPB40038 | T4742_S00001.117 | close homology to karyopherin alpha subunit from several other fungi |
| OPB40709 | T4742_S00001.789 | CmcJ-like methyltransferase |
| OPB40004 | T4742_S00001.83 | CMGC protein kinase BUR1 |
| OPB39970 | T4742_S00001.49 | CMGC type protein kinase |
| OPB39223 | T4742_S00006.283 | CMP/dCMP deaminase, zinc-binding |
| OPB46660 | T4742_S00009.102 | CMP/dCMP deaminase, zinc-binding |
| OPB38921 | T4742_S00002.970 | CND01770-like protein |
| OPB42002 | T4742_S00004.104 | CoA-binding |
| OPB37670 | T4742_S00005.697 | CoA-transferase family III |
| OPB42580 | T4742_S00004.683 | CoA-transferase family III |
| OPB46893 | T4742_S00009.335 | CoA-transferase family III |
| OPB46210 | T4742_S00008.277 | Cobalamin (vitamin B12) biosynthesis CobW-like |
| OPB36846 | T4742_S00007.564 | cobalamin synthesis protein |

| | | |
|---|---|---|
| OPB37331 | T4742_S00005.358 | CobW domain protein |
| OPB44998 | T4742_S00013.303 | Coenzyme A transferase |
| OPB43404 | T4742_S00019.137 | coenzyme F420-dependent N5,N10-methylene tetrahydromethanopterin reductase |
| OPB41051 | T4742_S00020.75 | coenzyme F420-dependent NADP oxidoreductase |
| OPB38583 | T4742_S00002.632 | Coenzyme Q (ubiquinone) biosynthesis protein Coq4, putative |
| OPB42436 | T4742_S00004.538 | cofilin |
| OPB42242 | T4742_S00004.344 | Cohesin |
| OPB40036 | T4742_S00001.115 | cohesin complex subunit |
| OPB45067 | T4742_S00013.372 | cohesin complex subunit (Psm1) |
| OPB39371 | T4742_S00006.431 | coiled-coil domain-containing protein 55, putative |
| OPB42659 | T4742_S00004.764 | coiled-coil domain-containing protein, putative |
| OPB39138 | T4742_S00006.198 | Collagen triple helix repeat |
| OPB44040 | T4742_S00003.311 | Collagen triple helix repeat |
| OPB38072 | T4742_S00002.119 | COMPASS complex protein, putative |
| OPB41734 | T4742_S00018.57 | competence/damage-inducible protein [Trichoderma guizhouense] |
| OPB36959 | T4742_S00007.677 | Complex 1 LYR protein |
| OPB37049 | T4742_S00005.76 | complex 1 protein (LYR family) protein |
| OPB38548 | T4742_S00002.597 | complex I intermediate-associated protein 30 |
| OPB41426 | T4742_S00011.269 | Complex_LYRComplex  protein (LYR family) |
| OPB44946 | T4742_S00013.251 | Complex1_LYR, likely to be involved in Fe-S cluster biogenesis in mitochondria; putative |
| OPB36644 | T4742_S00007.362 | Component of oligomeric golgi complex Cog3/Sec34 |
| OPB42140 | T4742_S00004.242 | Component of oligomeric golgi complex Cog4/Sec38 |
| OPB37755 | T4742_S00017.22 | Component of oligomeric golgi complex Cog5 |
| OPB37734 | T4742_S00017.1 | Component of oligomeric golgi complex Cog6/Sec37 |
| OPB39638 | T4742_S00006.698 | Component of oligomeric golgi complex Cog8 |
| OPB42235 | T4742_S00004.337 | Component, Pan1, of actinCytoskeleton-regulatoryComplex Pan1p-Sla1p-End3p, involved in endocytosis |
| OPB42996 | T4742_S00012.233 | condensin complex component cnd2 |
| OPB38411 | T4742_S00002.459 | condensin complex component SMC2 |
| OPB44564 | T4742_S00003.836 | conensin complex subunit |
| OPB41395 | T4742_S00011.237 | conidiation-specific protein 10 |
| OPB44171 | T4742_S00003.442 | conidiospore surface protein cmp1 |
| OPB44531 | T4742_S00003.803 | conserved hypothetical protein [Byssochlamys spectabilis No. 5] |
| OPB43753 | T4742_S00003.24 | conserved hypothetical protein [Talaromyces marneffei ATCC 18224] |
| OPB41718 | T4742_S00018.41 | conserved hypothetical protein [Verticillium alfalfae VaMs.102] |
| OPB42015 | T4742_S00004.117 | Conserved hypothetical protein CHP02453 |
| OPB43949 | T4742_S00003.220 | contains: UBA/TS-N domain and DNA-binding domain |

| | | |
|---|---|---|
| OPB37881 | T4742_S00017.148 | COP9 signalosome complex subunit 12 |
| OPB41270 | T4742_S00011.111 | COP9 signalosome subunit 2 (CsnB) |
| OPB38331 | T4742_S00002.379 | COPI-coated vesicle protein, putative |
| OPB37324 | T4742_S00005.351 | COPII coat assembly protein Sec16 |
| OPB36163 | T4742_S00021.80 | copper amine oxidase |
| OPB38821 | T4742_S00002.870 | Copper amine oxidase |
| OPB45312 | T4742_S00010.223 | Copper amine oxidase |
| OPB45488 | T4742_S00010.399 | copper amine oxidase |
| OPB40973 | T4742_S00001.1054 | Copper chaperone for superoxide dismutase |
| OPB36557 | T4742_S00007.274 | copper fist DNA-binding domain-containing protein |
| OPB42947 | T4742_S00012.184 | copper radical oxidase [Trichoderma gamsii] |
| OPB36285 | T4742_S00007.2 | Copper transporter |
| OPB37761 | T4742_S00017.28 | Copper transporter |
| OPB39964 | T4742_S00001.43 | copper transporter |
| OPB41197 | T4742_S00011.37 | Copper transporter |
| OPB42286 | T4742_S00004.388 | copper transporter |
| OPB43512 | T4742_S00016.32 | Coproporphyrinogen III oxidase |
| OPB42904 | T4742_S00012.141 | CorA family metal ion transporter [Metarhizium anisopliae ARSEF 23] |
| OPB42061 | T4742_S00004.163 | COX17, cytochrome C oxidase Cu chaperone |
| OPB41955 | T4742_S00004.57 | CPC1, cross-pathway control protein 1 |
| OPB36030 | T4742_S00023.65 | CPC2, ativator of general amino acid control |
| OPB36957 | T4742_S00007.675 | CRAL/TRIO domain-containing protein |
| OPB43584 | T4742_S00016.104 | CRO1, required for syncytial to cellular transision, involved in sexual development |
| OPB41029 | T4742_S00020.53 | CsdB Selenocysteine lyase |
| OPB46903 | T4742_S00009.345 | CsdB Selenocysteine lyase |
| OPB43188 | T4742_S00012.425 | Csh7, an ER membrane chaperone protein |
| OPB39362 | T4742_S00006.422 | CTD kinase subunit gamma, putative |
| OPB38115 | T4742_S00002.162 | CTP synthase |
| OPB42938 | T4742_S00012.175 | Ctr copper transporter, putative |
| OPB43240 | T4742_S00022.43 | C-type cyclin |
| OPB45529 | T4742_S00010.440 | C-type cyclin |
| OPB38405 | T4742_S00002.453 | Cue1 domain protein involved in ER-associated protein degradation. Distantly related to mammalian AMFR2 |
| OPB42934 | T4742_S00012.171 | Cullin |
| OPB45170 | T4742_S00010.77 | Cullin (cell cycle) |
| OPB46791 | T4742_S00009.233 | Cullin family protein |
| OPB41834 | T4742_S00018.157 | Cu-oxidase_2,Cu-oxidase_3 |

| | | |
|---|---|---|
| OPB44694 | T4742_S00003.966 | cupin domain protein |
| OPB45483 | T4742_S00010.394 | cupin domain protein |
| OPB46333 | T4742_S00008.400 | cupin domain protein |
| OPB46535 | T4742_S00008.602 | cupin domain protein |
| OPB37544 | T4742_S00005.571 | Cupin, RmlC-type |
| OPB40718 | T4742_S00001.798 | Cupin, RmlC-type |
| OPB38023 | T4742_S00002.70 | Cut9 interacting protein Scn1, putative |
| OPB40012 | T4742_S00001.91 | CutC family protein |
| OPB40797 | T4742_S00001.878 | cutinase |
| OPB46214 | T4742_S00008.281 | Cutinase |
| OPB45196 | T4742_S00010.104 | Cwf15/Cwc15 cell cycle control protein |
| OPB39132 | T4742_S00006.192 | cyanamide hydratase |
| OPB46023 | T4742_S00008.89 | cyanamide hydratase |
| OPB46049 | T4742_S00008.115 | cyanamide hydratase |
| OPB43469 | T4742_S00019.202 | cyanate hydratase |
| OPB46736 | T4742_S00009.178 | cyanoviridin N |
| OPB38142 | T4742_S00002.189 | Cyanovirin-N |
| OPB46349 | T4742_S00008.416 | cyanovirin-N family protein |
| OPB37997 | T4742_S00002.44 | CybS |
| OPB44450 | T4742_S00003.722 | CybS |
| OPB46363 | T4742_S00008.430 | cyclase |
| OPB46419 | T4742_S00008.486 | cyclase |
| OPB36516 | T4742_S00007.233 | Cyclin |
| OPB44810 | T4742_S00013.115 | Cyclin |
| OPB41106 | T4742_S00020.130 | Cyclin C-dependent kinase CDK8 |
| OPB38261 | T4742_S00002.308 | cyclin domain-containing protein |
| OPB36988 | T4742_S00005.15 | cyclin like protein |
| OPB40863 | T4742_S00001.944 | Cyclin, N-terminal |
| OPB44174 | T4742_S00003.445 | cyclin-dependent kinase regulatory subunit |
| OPB42174 | T4742_S00004.276 | Cyclin-K , putative |
| OPB39302 | T4742_S00006.362 | cyclin-like F-box |
| OPB39501 | T4742_S00006.561 | Cyclin-like F-box |
| OPB42611 | T4742_S00004.715 | Cyclin-like F-box |
| OPB45219 | T4742_S00010.128 | Cyclin-like F-box |
| OPB41410 | T4742_S00011.252 | Cyclin-related 2 |
| OPB44765 | T4742_S00013.70 | cyclohexanone monooxygenase |

| | | |
|---|---|---|
| OPB41625 | T4742_S00011.468 | Cyclophilin type peptidyl-prolyl cis-trans isomerase |
| OPB43936 | T4742_S00003.207 | cyclophilin type peptidyl-prolyl cis-trans isomerase/CLD |
| OPB44332 | T4742_S00003.603 | cyclophilin type peptidyl-prolyl cis-trans isomerase/CLD |
| OPB42163 | T4742_S00004.265 | cyclophilin-type peptidyl-prolyl cis-trans isomerase |
| OPB43852 | T4742_S00003.123 | cyclopropane/fatty acid synthase; plant related |
| OPB41461 | T4742_S00011.304 | Cyclopropane-fatty-acyl-phospholipid synthase |
| OPB36975 | T4742_S00005.2 | Cys/Met metabolism PLP-dependent enzyme |
| OPB39264 | T4742_S00006.324 | Cys/Met metabolism, pyridoxal phosphate-dependent enzyme |
| OPB45502 | T4742_S00010.413 | Cys/Met metabolism, pyridoxal phosphate-dependent enzyme |
| OPB39171 | T4742_S00006.231 | CysK, Cysteine synthase |
| OPB39324 | T4742_S00006.384 | CysKCysteine synthase |
| OPB44345 | T4742_S00003.616 | CysKCysteine synthase |
| OPB37972 | T4742_S00002.19 | cystathionine beta-lyase |
| OPB36964 | T4742_S00007.682 | Cystathionine beta-lyases/cystathionine gamma-synthases |
| OPB37384 | T4742_S00005.411 | Cystathionine beta-lyases/cystathionine gamma-synthases |
| OPB44160 | T4742_S00003.431 | Cystathionine beta-lyases/cystathionine gamma-synthases |
| OPB37039 | T4742_S00005.66 | cystathionine beta-synthase (beta-thionase), putative |
| OPB36695 | T4742_S00007.413 | cysteic acid decarboxylase |
| OPB38212 | T4742_S00002.259 | cysteine dioxygenase |
| OPB46021 | T4742_S00008.87 | cysteine peptidase |
| OPB36912 | T4742_S00007.630 | cysteine protease, mammalian caspases |
| OPB40668 | T4742_S00001.748 | cysteine synthase A |
| OPB45597 | T4742_S00010.508 | cysteine synthase A |
| OPB43419 | T4742_S00019.152 | cysteine synthase, putative |
| OPB37277 | T4742_S00005.304 | cysteinyl-tRNA synthetase, class Ia. |
| OPB40024 | T4742_S00001.103 | cystinosin |
| OPB41544 | T4742_S00011.387 | cytidine and deoxycytidylate deaminase zinc-binding region |
| OPB44073 | T4742_S00003.344 | Cytidine/deoxycytidylate deaminase, zinc-binding region |
| OPB40114 | T4742_S00001.193 | Cytidylyltransferase |
| OPB38043 | T4742_S00002.90 | cytochrome b2, mitochondrial precursor |
| OPB40402 | T4742_S00001.481 | Cytochrome b5 |
| OPB42566 | T4742_S00004.669 | Cytochrome b5 |
| OPB37647 | T4742_S00005.674 | cytochrome b5 reductase |
| OPB43642 | T4742_S00016.162 | cytochrome b5 reductase |
| OPB44460 | T4742_S00003.732 | cytochrome b5 reductase |
| OPB36355 | T4742_S00007.72 | cytochrome b5, putative |

| | | |
|---|---|---|
| OPB37473 | T4742_S00005.500 | cytochrome b5, putative |
| OPB43164 | T4742_S00012.401 | cytochrome b5-like Heme/Steroid binding domain-containing protein |
| OPB37127 | T4742_S00005.154 | cytochrome b5-like Heme/Steroid binding domain-containing protein |
| OPB43983 | T4742_S00003.254 | Cytochrome bd ubiquinol oxidase, 14 kDa subunit |
| OPB43998 | T4742_S00003.269 | Cytochrome bd ubiquinol oxidase, 14 kDa subunit |
| OPB43564 | T4742_S00016.84 | cytochrome c |
| OPB46405 | T4742_S00008.472 | Cytochrome c and c1 heme-lyase, shares homology to Protein prenyltransferase, alpha subunit |
| OPB36568 | T4742_S00007.285 | cytochrome c heme lyase, putative |
| OPB37326 | T4742_S00005.353 | cytochrome c mitochondrial import factor CYC2 |
| OPB36313 | T4742_S00007.30 | cytochrome c oxidase assembly protein |
| OPB35931 | T4742_S00025.4 | cytochrome c oxidase assembly protein (Pet117), putative |
| OPB45337 | T4742_S00010.248 | cytochrome c oxidase assembly protein COX16, putative |
| OPB39544 | T4742_S00006.604 | cytochrome c oxidase assembly protein COX19, putative |
| OPB43597 | T4742_S00016.117 | Cytochrome c oxidase assembly protein CtaG/Cox11 |
| OPB41670 | T4742_S00011.513 | Cytochrome c oxidase subunit IV |
| OPB38551 | T4742_S00002.600 | cytochrome c oxidase subunit VIa |
| OPB40991 | T4742_S00020.15 | Cytochrome c oxidase, subunit Vb |
| OPB46990 | T4742_S00009.432 | cytochrome c oxidase-like protein |
| OPB37066 | T4742_S00005.93 | cytochrome C peroxidase |
| OPB42055 | T4742_S00004.157 | Cytochrome c1, heme protein, mitochondrial |
| OPB42414 | T4742_S00004.516 | cytochrome oxidase c subunit VIb |
| OPB36836 | T4742_S00007.554 | cytochrome P450 |
| OPB37732 | T4742_S00005.760 | cytochrome P450 |
| OPB38536 | T4742_S00002.585 | cytochrome P450 |
| OPB39534 | T4742_S00006.594 | Cytochrome P450 |
| OPB40730 | T4742_S00001.810 | Cytochrome P450 |
| OPB40768 | T4742_S00001.849 | cytochrome P450 |
| OPB41710 | T4742_S00018.33 | cytochrome P450 |
| OPB41872 | T4742_S00018.195 | Cytochrome P450 |
| OPB42894 | T4742_S00012.131 | Cytochrome P450 |
| OPB44461 | T4742_S00003.733 | cytochrome P450 |
| OPB44468 | T4742_S00003.740 | Cytochrome P450 |
| OPB44526 | T4742_S00003.798 | Cytochrome P450 |
| OPB44605 | T4742_S00003.877 | cytochrome P450 |
| OPB45217 | T4742_S00010.126 | cytochrome p450 |
| OPB45442 | T4742_S00010.353 | Cytochrome P450 |

| | | |
|---|---|---|
| OPB46087 | T4742_S00008.154 | Cytochrome P450 |
| OPB46285 | T4742_S00008.352 | cytochrome P450 |
| OPB46559 | T4742_S00008.626 | cytochrome p450 |
| OPB38919 | T4742_S00002.968 | cytochrome P450 |
| OPB40341 | T4742_S00001.420 | cytochrome P450 |
| OPB46387 | T4742_S00008.454 | cytochrome P450 |
| OPB43559 | T4742_S00016.79 | Cytochrome P450 / E-class P450, group I |
| OPB40645 | T4742_S00001.725 | cytochrome P450 [Trichoderma guizhouense] |
| OPB46485 | T4742_S00008.552 | Cytochrome P450 [Trichoderma parareesei] |
| OPB45647 | T4742_S00010.558 | Cytochrome P450 CYP11/CYP12/CYP24/CYP27 subfamilies |
| OPB36219 | T4742_S00021.137 | Cytochrome P450 CYP2 subfamily |
| OPB39843 | T4742_S00015.187 | Cytochrome P450 CYP2 subfamily |
| OPB41216 | T4742_S00011.56 | Cytochrome P450 CYP2 subfamily |
| OPB43354 | T4742_S00019.87 | Cytochrome P450 CYP2 subfamily |
| OPB44065 | T4742_S00003.336 | Cytochrome P450 CYP2 subfamily |
| OPB45738 | T4742_S00014.73 | Cytochrome P450 CYP2 subfamily |
| OPB45934 | T4742_S00014.271 | Cytochrome P450 CYP2 subfamily |
| OPB46126 | T4742_S00008.193 | Cytochrome P450 CYP2 subfamily |
| OPB46303 | T4742_S00008.370 | Cytochrome P450 CYP2 subfamily |
| OPB44615 | T4742_S00003.887 | Cytochrome P450 CYP3/CYP5/CYP6/CYP9 subfamilies |
| OPB44196 | T4742_S00003.467 | Cytochrome P450 CYP4/CYP19/CYP26 subfamilies |
| OPB45820 | T4742_S00014.155 | Cytochrome P450 dependent monooxygenase |
| OPB45822 | T4742_S00014.157 | Cytochrome P450 dependent monooxygenase |
| OPB40328 | T4742_S00001.407 | cytochrome P450 dependent quinol monooxygenase |
| OPB44036 | T4742_S00003.307 | Cytochrome P450 family protein |
| OPB41171 | T4742_S00011.11 | cytochrome P-450 lanosterol demethylase; ergosterol biosynthetic pathway |
| OPB46839 | T4742_S00009.281 | Cytochrome P-450 lanosterol demethylase; ergosterol biosynthetic pathway |
| OPB45826 | T4742_S00014.161 | cytochrome P450 monoounknown proteingenase (trichothecene C-15 hydrounknown proteinlase) |
| OPB36088 | T4742_S00021.4 | cytochrome P450 monooxygenase |
| OPB36089 | T4742_S00021.5 | cytochrome P450 monooxygenase |
| OPB36091 | T4742_S00021.7 | cytochrome P450 monooxygenase |
| OPB36128 | T4742_S00021.45 | cytochrome P450 monooxygenase |
| OPB36502 | T4742_S00007.219 | cytochrome P450 monooxygenase |
| OPB36709 | T4742_S00007.427 | cytochrome P450 monooxygenase |
| OPB36800 | T4742_S00007.518 | cytochrome P450 monooxygenase |
| OPB36803 | T4742_S00007.521 | cytochrome P450 monooxygenase |

| | | |
|---|---|---|
| OPB37648 | T4742_S00005.675 | cytochrome P450 monooxygenase |
| OPB37693 | T4742_S00005.720 | cytochrome P450 monooxygenase |
| OPB37780 | T4742_S00017.47 | cytochrome P450 monooxygenase |
| OPB37781 | T4742_S00017.48 | cytochrome P450 monooxygenase |
| OPB39125 | T4742_S00006.185 | Cytochrome P450 monooxygenase |
| OPB39587 | T4742_S00006.647 | cytochrome P450 monooxygenase |
| OPB39959 | T4742_S00001.38 | cytochrome P450 monooxygenase |
| OPB40752 | T4742_S00001.833 | Cytochrome P450 monooxygenase |
| OPB41007 | T4742_S00020.31 | cytochrome P450 monooxygenase |
| OPB41192 | T4742_S00011.32 | cytochrome P450 monooxygenase |
| OPB41205 | T4742_S00011.45 | Cytochrome P450 monooxygenase |
| OPB41220 | T4742_S00011.61 | cytochrome P450 monooxygenase |
| OPB41700 | T4742_S00018.22 | cytochrome P450 monooxygenase |
| OPB41703 | T4742_S00018.25 | Cytochrome P450 monooxygenase |
| OPB41811 | T4742_S00018.134 | cytochrome P450 monooxygenase |
| OPB41874 | T4742_S00018.197 | cytochrome P450 monooxygenase |
| OPB42091 | T4742_S00004.193 | cytochrome P450 monooxygenase |
| OPB42737 | T4742_S00004.843 | cytochrome P450 monooxygenase |
| OPB42893 | T4742_S00012.130 | cytochrome P450 monooxygenase |
| OPB43641 | T4742_S00016.161 | cytochrome P450 monooxygenase |
| OPB43736 | T4742_S00003.7 | cytochrome P450 monooxygenase |
| OPB44033 | T4742_S00003.304 | cytochrome P450 monooxygenase |
| OPB44509 | T4742_S00003.781 | cytochrome P450 monooxygenase |
| OPB44808 | T4742_S00013.113 | Cytochrome P450 monooxygenase |
| OPB44873 | T4742_S00013.178 | cytochrome P450 monooxygenase |
| OPB44890 | T4742_S00013.195 | cytochrome P450 monooxygenase |
| OPB44969 | T4742_S00013.274 | cytochrome P450 monooxygenase |
| OPB45043 | T4742_S00013.348 | cytochrome P450 monooxygenase |
| OPB45230 | T4742_S00010.139 | cytochrome P450 monooxygenase |
| OPB45870 | T4742_S00014.205 | cytochrome P450 monooxygenase |
| OPB45993 | T4742_S00008.59 | cytochrome P450 monooxygenase |
| OPB46522 | T4742_S00008.589 | cytochrome P450 monooxygenase |
| OPB46523 | T4742_S00008.590 | cytochrome P450 monooxygenase |
| OPB46543 | T4742_S00008.610 | Cytochrome P450 monooxygenase |
| OPB43401 | T4742_S00019.134 | cytochrome P450 monooxygenase |
| OPB41526 | T4742_S00011.369 | cytochrome P450 monooxygenase, putative |

| | | |
|---|---|---|
| OPB45266 | T4742_S00010.177 | cytochrome P450 protein, class IV |
| OPB42283 | T4742_S00004.385 | Cytochrome P450, E-class, group I |
| OPB41981 | T4742_S00004.83 | Cytochrome P450, E-class, group IV |
| OPB43930 | T4742_S00003.201 | Cytochrome P450, E-class, group IV |
| OPB44434 | T4742_S00003.706 | Cytochrome P450, E-class, group IV |
| OPB46123 | T4742_S00008.190 | Cytochrome P450, E-class, group IV |
| OPB36842 | T4742_S00007.560 | Cytochrome P450, putative |
| OPB46117 | T4742_S00008.184 | Cytochrome P450, putative |
| OPB40585 | T4742_S00001.665 | cytochrome P450, putative |
| OPB43459 | T4742_S00019.192 | cytochrome P450, putative |
| OPB46332 | T4742_S00008.399 | cytochrome P450, putative |
| OPB45493 | T4742_S00010.404 | cytochrome P450/benzoate 4-monooxygenase |
| OPB38757 | T4742_S00002.806 | Cytochrome P450/O-methylsterigmatocystin oxidoreductase |
| OPB46662 | T4742_S00009.104 | cytochrome P450-like protein |
| OPB39841 | T4742_S00015.185 | Cytochrome P450monooxygenase |
| OPB46889 | T4742_S00009.331 | Cytochrome P450monooxygenase |
| OPB37695 | T4742_S00005.722 | cytochrome-b5 reductase [Fusarium verticillioides 7600] |
| OPB42438 | T4742_S00004.540 | cytokinesis regulator, putative |
| OPB37766 | T4742_S00017.33 | Cytoplasmic Cyclophilin |
| OPB39240 | T4742_S00006.300 | cytoplasmic dynein intermediate chain (?) |
| OPB38330 | T4742_S00002.378 | cytosceleton binding protein |
| OPB41841 | T4742_S00018.164 | cytosin/purin permease |
| OPB40695 | T4742_S00001.775 | cytosine permease |
| OPB44568 | T4742_S00003.840 | Cytosine-purine permease |
| OPB39639 | T4742_S00006.699 | Cytoskeleton assemblyControl protein, sla1, interacts with proteins regulating actin dynamics and with proteins required for endocytosis |
| OPB38329 | T4742_S00002.377 | cytosolic asparaginyl-tRNA synthetase, required for protein synthesis, catalyzes the specific attachment of asparagine to its cognate tRNA. |
| OPB37652 | T4742_S00005.679 | Cytosolic fatty-acid binding |
| OPB42166 | T4742_S00004.268 | Cytosolic Fe-S cluster assembly factor NAR1, putative |
| OPB37998 | T4742_S00002.45 | cytosolic phospholipase A2, putative |
| OPB38109 | T4742_S00002.156 | D-3-phosphoglycerate dehydrogenase |
| OPB42635 | T4742_S00004.740 | D-3-phosphoglycerate dehydrogenase |
| OPB45345 | T4742_S00010.256 | DadA Glycine/D-amino acid oxidases (deaminating) [Amino acid transport and metabolism] |
| OPB36612 | T4742_S00007.330 | DAHP synthase ARO4 |
| OPB36231 | T4742_S00021.149 | D-Alanine aminotransferase |
| OPB38906 | T4742_S00002.955 | D-Alanine aminotransferase |
| OPB44703 | T4742_S00013.7 | D-aminoacylase |

| | | |
|---|---|---|
| OPB46037 | T4742_S00008.103 | D-aminoacylase |
| OPB45610 | T4742_S00010.521 | D-aminoacylase, putative |
| OPB46487 | T4742_S00008.554 | D-aminopeptidase |
| OPB36394 | T4742_S00007.111 | DANN binding protein with copper fist |
| OPB37927 | T4742_S00017.194 | D-arabinitol 2-dehydrogenase [ribulose-forming] [Tolypocladium ophioglossoides CBS 100239] |
| OPB41907 | T4742_S00004.9 | D-arabinitol dehydrogenase |
| OPB45203 | T4742_S00010.111 | D-arabinono-1,4-lactone oxidase |
| OPB42507 | T4742_S00004.609 | DASH complex subunit DAD1 |
| OPB38817 | T4742_S00002.866 | DASH complex subunit Dad3 |
| OPB38207 | T4742_S00002.254 | DASH complex subunit Dad4 |
| OPB36227 | T4742_S00021.145 | D-aspartate oxidase |
| OPB43851 | T4742_S00003.122 | D-aspartate oxidase |
| OPB38701 | T4742_S00002.750 | DBF2, cell cyle protein kinase |
| OPB45552 | T4742_S00010.463 | DCL1, Dicer-like protein, involved in quelling |
| OPB46253 | T4742_S00008.320 | DCL2, Dicer-like protein, involved in quelling |
| OPB44841 | T4742_S00013.146 | Dcp1-like decapping |
| OPB40039 | T4742_S00001.118 | DEAD box helicase |
| OPB38413 | T4742_S00002.461 | DEAD box helicase Hel1 |
| OPB46084 | T4742_S00008.151 | DEAD helicases superfamily protein (Aquarius), putative |
| OPB40040 | T4742_S00001.119 | DEAD/DEAH box helicase |
| OPB38485 | T4742_S00002.533 | DEAD/DEAH box helicase |
| OPB39028 | T4742_S00006.87 | DEAD/DEAH box helicase |
| OPB43016 | T4742_S00012.253 | DEAD/DEAH box helicase |
| OPB43017 | T4742_S00012.254 | DEAD/DEAH box helicase |
| OPB43133 | T4742_S00012.370 | DEAD/DEAH box helicase |
| OPB43482 | T4742_S00016.2 | DEAD/DEAH box helicase, putative |
| OPB43483 | T4742_S00016.3 | DEAD/DEAH box helicase, putative |
| OPB38791 | T4742_S00002.840 | DEAD/DEAH box RNA helicase (Ski2), putative |
| OPB36055 | T4742_S00024.16 | decarboxylase/2-amino-3-carboxymuconate-6-semialdehyde decarboxylase |
| OPB45496 | T4742_S00010.407 | dehydrogenase associated with cellulase signal transduction (PMID: 15288024) |
| OPB41481 | T4742_S00011.324 | Dehydroquinase class I |
| OPB41476 | T4742_S00011.319 | Dehydroquinase, class II |
| OPB39364 | T4742_S00006.424 | Delta 1-pyrroline-5-carboxylate reductase |
| OPB46097 | T4742_S00008.164 | Delta subunit, ret2, of the coatomer complex (COPI), which coats Golgi-derived transport vesicles; involved in retrograde transport between Golgi and ER |
| OPB39056 | T4742_S00006.115 | delta(14)-sterol reductase |
| OPB39840 | T4742_S00015.184 | Delta(6)-protoilludene synthase [Valsa mali var. pyri] |

| | | |
|---|---|---|
| OPB36778 | T4742_S00007.496 | delta-1-pyrroline-5-carboxylate dehydrogenase |
| OPB37364 | T4742_S00005.391 | delta-1-pyrroline-5-carboxylate dehydrogenase |
| OPB37076 | T4742_S00005.103 | Demethoxyubiquinone hydroxylase |
| OPB43526 | T4742_S00016.46 | demethylmenaquinone methyltransferase family protein |
| OPB38286 | T4742_S00002.333 | deounknown proteinhypusine hydrounknown proteinlase |
| OPB42509 | T4742_S00004.611 | deounknown proteinhypusine synthase |
| OPB42183 | T4742_S00004.285 | deounknown proteinribonuclease TatD |
| OPB36726 | T4742_S00007.444 | deoxyribonuclease, extracellular |
| OPB45181 | T4742_S00010.88 | deoxyribose-phosphate aldolase |
| OPB46783 | T4742_S00009.225 | deoxyuridine 5'-triphosphate nucleotidohydrolase |
| OPB40116 | T4742_S00001.195 | Der1, and ER membrane protein involved in ER-associated protein degradation. |
| OPB45941 | T4742_S00008.7 | Der1-like |
| OPB37120 | T4742_S00005.147 | Developmental regulatory protein WetA |
| OPB38730 | T4742_S00002.779 | D-galacturonic acid reductase |
| OPB37349 | T4742_S00005.376 | D-galacturonic acid reductase |
| OPB41284 | T4742_S00011.125 | dgpfaetke family protein |
| OPB36485 | T4742_S00007.202 | DHBP_synthase,-dihydroxy--butanone -phosphate synthase |
| OPB45976 | T4742_S00008.42 | DHHD transcriptional regulator |
| OPB43651 | T4742_S00016.171 | DHQase_I, Type I 3-dehydroquinase |
| OPB43132 | T4742_S00012.369 | Diacylglycerol kinase |
| OPB40606 | T4742_S00001.686 | diacylglycerol pyrophosphate phosphatase |
| OPB39623 | T4742_S00006.683 | diacylglycerol synthase |
| OPB46182 | T4742_S00008.249 | dicarboxylic acid permease, malate |
| OPB37699 | T4742_S00005.726 | Dienelactone hydrolase |
| OPB38180 | T4742_S00002.227 | dienelactone hydrolase |
| OPB40697 | T4742_S00001.777 | dienelactone hydrolase |
| OPB41628 | T4742_S00011.471 | dienelactone hydrolase |
| OPB42699 | T4742_S00004.805 | Dienelactone hydrolase |
| OPB43732 | T4742_S00003.3 | Dienelactone hydrolase |
| OPB43873 | T4742_S00003.144 | dienelactone hydrolase |
| OPB45423 | T4742_S00010.334 | Dienelactone hydrolase |
| OPB45871 | T4742_S00014.206 | dienelactone hydrolase |
| OPB47051 | T4742_S00009.493 | Dienelactone hydrolase |
| OPB40508 | T4742_S00001.587 | dienelactone hydrolase [Colletotrichum salicis] |
| OPB45474 | T4742_S00010.385 | dienelactone hydrolase domain protein |
| OPB41897 | T4742_S00018.220 | dienelactone hydrolase family protein |

| | | |
|---|---|---|
| OPB41303 | T4742_S00011.144 | dihydrodipicolinate synthase |
| OPB41465 | T4742_S00011.308 | dihydrodipicolinate synthase , putative |
| OPB40270 | T4742_S00001.349 | dihydrodipicolinate synthase, putative |
| OPB44385 | T4742_S00003.656 | dihydrodipicolinate synthetase [Colletotrichum graminicola M1.001] |
| OPB36824 | T4742_S00007.542 | dihydrodipicolinate synthetase, putative |
| OPB37598 | T4742_S00005.625 | dihydroflavonal-4-reductase |
| OPB42816 | T4742_S00012.53 | dihydroflavonol-4-reductase |
| OPB45932 | T4742_S00014.269 | dihydroflavonol-4-reductase |
| OPB39400 | T4742_S00006.460 | dihydrofolate reductase |
| OPB39401 | T4742_S00006.461 | dihydrofolate reductase |
| OPB46162 | T4742_S00008.229 | Dihydrolipoamide acetyltransferase |
| OPB46819 | T4742_S00009.261 | Dihydrolipoamide transacylase (alpha-keto acid dehydrogenase E2 subunit) |
| OPB36946 | T4742_S00007.664 | dihydrolipoyl dehydrogenase (E3 component of both 2-oxoglutarate dehydrogenase and pyruvate dehydrogenase complexes) |
| OPB42491 | T4742_S00004.593 | dihydrolipoyllysine-residue acetyltransferase-like protein |
| OPB44074 | T4742_S00003.345 | dihydroorotase |
| OPB43269 | T4742_S00019.2 | dihydroorotate dehydrogenase |
| OPB43447 | T4742_S00019.180 | dihydrounknown protein-acid dehydratase |
| OPB44269 | T4742_S00003.540 | Dihydrouridine synthase, DuS. |
| OPB37421 | T4742_S00005.448 | dihydroxacetione kinase Dak1 |
| OPB39153 | T4742_S00006.213 | dihydroxacetione kinase Dak1 |
| OPB41466 | T4742_S00011.309 | dihydroxyacetone kinase |
| OPB44386 | T4742_S00003.657 | dihydroxyacetone kinase (Dak kinase) |
| OPB40286 | T4742_S00001.365 | Dihydroxy-acid and 6-phosphogluconate dehydratase |
| OPB40504 | T4742_S00001.583 | dihydroxyacid dehydratase [Trichoderma guizhouense] |
| OPB42796 | T4742_S00012.33 | Dimeric alpha-beta barrel |
| OPB43946 | T4742_S00003.217 | Dimeric alpha-beta barrel protein |
| OPB42769 | T4742_S00012.6 | dimethylamide:corrinoid transferase, putative |
| OPB44295 | T4742_S00003.566 | Dioxygenases related to -nitropropane dioxygenase |
| OPB42917 | T4742_S00012.154 | DIP2, encoding a nucleolar protein, specifically associated with the U3 snoRNA of the SSU processome. |
| OPB39944 | T4742_S00001.23 | dipeptidase |
| OPB37784 | T4742_S00017.51 | Dipeptidyl aminopeptidase |
| OPB41397 | T4742_S00011.239 | dipeptidyl aminopeptidase, alpha factor processing |
| OPB36136 | T4742_S00021.53 | dipeptidyl peptidase 5 |
| OPB36777 | T4742_S00007.495 | dipeptidyl peptidase 5 |
| OPB37941 | T4742_S00017.208 | dipeptidyl peptidase 5 |
| OPB38782 | T4742_S00002.831 | dipeptidyl peptidase 5 |

| | | |
|---|---|---|
| OPB39557 | T4742_S00006.617 | dipeptidyl peptidase 5 |
| OPB40815 | T4742_S00001.896 | dipeptidyl peptidase 5 |
| OPB42265 | T4742_S00004.367 | dipeptidyl peptidase 5 |
| OPB43332 | T4742_S00019.65 | dipeptidyl peptidase 5 |
| OPB45477 | T4742_S00010.388 | dipeptidyl peptidase 5 |
| OPB46073 | T4742_S00008.140 | dipeptidyl peptidase 5 |
| OPB46039 | T4742_S00008.105 | dipeptidyl peptidase III |
| OPB46817 | T4742_S00009.259 | Dipeptidyl peptidase III, member of MEROPS peptidase family M49 of metallopeptidases; associated to cellulase signal transduction (PMID: 15288024) |
| OPB40788 | T4742_S00001.869 | dipeptidyl peptidase IV |
| OPB39283 | T4742_S00006.343 | diphthamide biosynthesis protein 1 |
| OPB38157 | T4742_S00002.204 | Diphthine synthase |
| OPB41420 | T4742_S00011.263 | D-isomer specific 2-hydroxyacid dehydrogenase |
| OPB46515 | T4742_S00008.582 | D-isomer specific 2-hydroxyacid dehydrogenase |
| OPB42744 | T4742_S00004.850 | D-isomer specific 2-hydroxyacid dehydrogenase, catalytic region |
| OPB41164 | T4742_S00011.4 | D-isomer specific 2-hydroxyacid dehydrogenase, NAD-binding |
| OPB41609 | T4742_S00011.452 | D-isomer-specific 2-hydroxy acid dehydrogenase |
| OPB43895 | T4742_S00003.166 | distant similarity to Mollulscan rhodopsin C-terminal tail, potentially involved in signal transduction |
| OPB43896 | T4742_S00003.167 | distant similarity to Mollulscan rhodopsin C-terminal tail, potentially involved in signal transduction |
| OPB44511 | T4742_S00003.783 | disulfide oxidoreductase |
| OPB40583 | T4742_S00001.663 | Dit1p, spore wall maturation |
| OPB39875 | T4742_S00015.219 | di-trans,poly-cis-decaprenylcistransferase |
| OPB37055 | T4742_S00005.82 | Di-trans,poly-cis-decaprenylcistransferase, putative |
| OPB42615 | T4742_S00004.719 | dityrosine transporter, required for spore wall synthesis |
| OPB46294 | T4742_S00008.361 | DJ-1/PfpI family protein |
| OPB43638 | T4742_S00016.158 | D-lactate dehydrogenase |
| OPB38501 | T4742_S00002.550 | D-lactate dehydrogenase, mitochondrial precursor |
| OPB46998 | T4742_S00009.440 | DNA damage-binding protein 1 |
| OPB38435 | T4742_S00002.483 | DNA damage-inducible v-SNARE binding protein Ddi1 |
| OPB39117 | T4742_S00006.177 | DNA directed RNA polymerase II 15 kDa subunit, putative |
| OPB42093 | T4742_S00004.195 | DNA glycosylase |
| OPB39148 | T4742_S00006.208 | DNA helicase |
| OPB39798 | T4742_S00015.142 | DNA helicase [Colletotrichum higginsianum IMI 349063] |
| OPB38989 | T4742_S00006.48 | DNA helicase, putative |
| OPB40946 | T4742_S00001.1027 | DNA ligase IV involved in non-homologous end joining of double-strand DNA breaks |
| OPB38550 | T4742_S00002.599 | DNA lyase |
| OPB37956 | T4742_S00002.3 | DNA mismatch repair protein, C-terminal |

| | | |
|---|---|---|
| OPB37792 | T4742_S00017.59 | DNA photolyase, class I, rapidly regulated by blue light in T. harzianum, PHR1 |
| OPB36878 | T4742_S00007.596 | DNA photolyase, N-terminal, class 1, FAD-binding |
| OPB38601 | T4742_S00002.650 | DNA photolyase, N-terminal, class 1, FAD-binding |
| OPB46082 | T4742_S00008.149 | DNA polymerase alpha catalytic subunit |
| OPB37425 | T4742_S00005.452 | DNA polymerase alpha-primase complex, polymerase-associated subunit B |
| OPB45064 | T4742_S00013.369 | DNA polymerase betadeoxyribonucleotidyl transferase activity. |
| OPB40080 | T4742_S00001.159 | DNA polymerase delta, catalytic subunit |
| OPB40063 | T4742_S00001.142 | DNA polymerase delta, small regulatory subunit |
| OPB39517 | T4742_S00006.577 | DNA polymerase epsilon, subunit B |
| OPB38649 | T4742_S00002.698 | DNA polymerase eta (RAD30) |
| OPB45177 | T4742_S00010.84 | DNA polymerase gamma, the mitochondrial replicativeDNA polymerase |
| OPB37898 | T4742_S00017.165 | DNA polymerase kappa, a Y family lesion bypass polymerase |
| OPB36705 | T4742_S00007.423 | DNA polymerase subunit epsilon |
| OPB42051 | T4742_S00004.153 | DNA polymerase V (phi) |
| OPB46866 | T4742_S00009.308 | DNA polymerase X family |
| OPB42476 | T4742_S00004.578 | DNA polymerase y family member involved in translesion synthesis during DNA repair |
| OPB39896 | T4742_S00015.240 | DNA polymeraseDelta, subunit 3 |
| OPB43156 | T4742_S00012.393 | DNA polymeraseDelta, subunit 4 |
| OPB36884 | T4742_S00007.602 | DNA primase, small subunit |
| OPB39832 | T4742_S00015.176 | DNA recombination and repair protein Rad51 [Aschersonia aleyrodis RCEF 2490] |
| OPB41542 | T4742_S00011.385 | DNA repair and TFIIH regulator, required for both nucleotide excision repair and RNA polymerase II transcription |
| OPB44343 | T4742_S00003.614 | DNA repair endonuclease Rad2p of Saccharomyces cerevisiae and ERCC5 (XPG) of Homo sapiens |
| OPB36275 | T4742_S00021.194 | DNA repair exonuclease SIA1 |
| OPB46570 | T4742_S00009.10 | DNA repair helicase |
| OPB44567 | T4742_S00003.839 | DNA repair helicase family protein |
| OPB38480 | T4742_S00002.528 | DNA repair protein |
| OPB40165 | T4742_S00001.244 | DNA repair protein (Tof1) |
| OPB39897 | T4742_S00015.241 | DNA repair protein rad18 |
| OPB37242 | T4742_S00005.269 | DNA repair protein Rad7 |
| OPB46690 | T4742_S00009.132 | DNA repair protein Rhp26/Rad26 |
| OPB43996 | T4742_S00003.267 | DNA repair protein Sae2/CtIP |
| OPB46996 | T4742_S00009.438 | DNA replication complex GINS protein (Psf2), putative |
| OPB46831 | T4742_S00009.273 | DNA replication complex GINS protein PSF1, putative |
| OPB46321 | T4742_S00008.388 | DNA replication complex GINS protein PSF3 |
| OPB42161 | T4742_S00004.263 | DNA replication complex GINS protein SLD5, putative |
| OPB37617 | T4742_S00005.644 | DNA replication factor A subunit Ssb3, putative |

| | | |
|---|---|---|
| OPB42460 | T4742_S00004.562 | DNA replication factor C, large subunit [Metarhizium rileyi RCEF 4871] |
| OPB46959 | T4742_S00009.401 | DNA replication factor Dna2 |
| OPB37434 | T4742_S00005.461 | DNA replication licensing factor mcm2 |
| OPB41833 | T4742_S00018.156 | DNA replication licensing factor mcm2 |
| OPB37965 | T4742_S00002.12 | DNA replication licensing factor mcm3, putative |
| OPB42143 | T4742_S00004.245 | DNA replication licensing factor mcm4 |
| OPB39891 | T4742_S00015.235 | DNA replication licensing factor mcm6 |
| OPB42986 | T4742_S00012.223 | DNA replication licensing factor mcm7 |
| OPB44943 | T4742_S00013.248 | DNA segregation ATPase FtsK/SpoIIIE |
| OPB38202 | T4742_S00002.249 | DNA/RNA helicase, C-terminal |
| OPB41912 | T4742_S00004.14 | DNA/RNA helicase, DEAD/DEAH box type, N-terminal |
| OPB40435 | T4742_S00001.514 | DNA/RNA methyltransferase. Shares significant amino acid sequence identity (E = 0.0) with yeast TRM11 |
| OPB45008 | T4742_S00013.313 | DNA-binding SAP |
| OPB45373 | T4742_S00010.284 | DNA-dependent RNA polymerase |
| OPB41553 | T4742_S00011.396 | DNA-directed DNA polymerase B |
| OPB40162 | T4742_S00001.241 | DNA-directed RNA polymerase II |
| OPB37020 | T4742_S00005.47 | DNA-directed RNA polymerase II subunit RPB7 |
| OPB39831 | T4742_S00015.175 | DNA-directed RNA polymerase III [Metarhizium anisopliae] |
| OPB41617 | T4742_S00011.460 | DNA-directed RNA polymerase III subunit C11 |
| OPB38828 | T4742_S00002.877 | DNA-directed RNA polymerase III subunit RPC1 |
| OPB43604 | T4742_S00016.124 | DNA-directed RNA polymerase III subunit, putative |
| OPB42227 | T4742_S00004.329 | DNA-directed RNA polymerase, 13 to 16 kDa subunit |
| OPB39760 | T4742_S00015.104 | DNA-directed RNA polymerase, 30-40 kDa subunit |
| OPB42910 | T4742_S00012.147 | DNA-directed RNA polymerase, 30-40 kDa subunit |
| OPB35929 | T4742_S00025.2 | DNA-directed RNA polymerase, beta subunit |
| OPB37346 | T4742_S00005.373 | DNAJ heat shock family protein, HSP40 |
| OPB37432 | T4742_S00005.459 | DNAse |
| OPB38846 | T4742_S00002.895 | Doa1, WD protein required for ubiquitin-mediated proteolysis |
| OPB37398 | T4742_S00005.425 | Dolichol kinase Sec59 |
| OPB39412 | T4742_S00006.472 | Dolichol-phosphate (beta-D) mannosyltransferase 2 |
| OPB42654 | T4742_S00004.759 | Dolichyl-phosphate mannosyltransferase |
| OPB36837 | T4742_S00007.555 | Dolichyl-phosphate mannosyltransferase polypeptide 3 |
| OPB36015 | T4742_S00023.50 | dolichyl-phosphate-mannose a-mannosyltransferases |
| OPB42138 | T4742_S00004.240 | Double-strand-break repair protein rad21 homolog |
| OPB44651 | T4742_S00003.923 | DPH2, required for diphthamide synthesis. |
| OPB44823 | T4742_S00013.128 | DSBA oxidoreductase |

| | | |
|---|---|---|
| OPB41286 | T4742_S00011.127 | DSBA-like thioredoxin domain-containing protein |
| OPB36353 | T4742_S00007.70 | DSBA-like thioredoxin domain-containing protein |
| OPB38584 | T4742_S00002.633 | dsDNA-binding protein PDCD5 |
| OPB41673 | T4742_S00011.516 | DSPc, Dual specificity phosphatases (DSP) |
| OPB41862 | T4742_S00018.185 | D-stereospecific peptide hydrolase |
| OPB44333 | T4742_S00003.604 | DSZ1-family protein, xenobiotic monooxygenase |
| OPB45103 | T4742_S00010.10 | dTDP-glucose 4,6-dehydratase |
| OPB43935 | T4742_S00003.206 | D-tyrosyl-tRNA(Tyr) deacylase |
| OPB40951 | T4742_S00001.1032 | dual specificity phosphatase |
| OPB46987 | T4742_S00009.429 | DUF1348 domain protein |
| OPB41785 | T4742_S00018.108 | DUF1446 domain protein |
| OPB46367 | T4742_S00008.434 | DUF1446 domain protein |
| OPB46420 | T4742_S00008.487 | DUF1446 domain protein |
| OPB38443 | T4742_S00002.491 | DUF500 domain-containing protein |
| OPB38444 | T4742_S00002.492 | DUF500 domain-containing protein |
| OPB35994 | T4742_S00023.29 | DUF636 domain protein |
| OPB40106 | T4742_S00001.185 | DUF636 domain protein |
| OPB41375 | T4742_S00011.216 | DUF636 domain protein |
| OPB41749 | T4742_S00018.72 | DUF636 domain protein |
| OPB37730 | T4742_S00005.758 | Duf895 10 TM domain protein |
| OPB42752 | T4742_S00004.858 | DUF895 domain membrane protein |
| OPB42479 | T4742_S00004.581 | DUF974 domain protein |
| OPB43540 | T4742_S00016.60 | dUTPase |
| OPB36866 | T4742_S00007.584 | D-xylulose 5-phosphate/D-fructose 6-phosphate phosphoketolase |
| OPB46728 | T4742_S00009.170 | D-xylulose 5-phosphate/D-fructose 6-phosphate phosphoketolase |
| OPB43975 | T4742_S00003.246 | dynactin Arp1 p62 subunit |
| OPB42389 | T4742_S00004.491 | dynactin ro-3 |
| OPB45409 | T4742_S00010.320 | dynamin family protein |
| OPB47040 | T4742_S00009.482 | dynamin GTPase [Trichoderma parareesei] |
| OPB47041 | T4742_S00009.483 | dynamin GTPase [Trichoderma parareesei] |
| OPB39929 | T4742_S00001.8 | Dynamin, GTPase region |
| OPB44964 | T4742_S00013.269 | Dynamin, GTPase region |
| OPB40071 | T4742_S00001.150 | Dynamin-like GTPase Vps1 |
| OPB39545 | T4742_S00006.605 | Dynamin-related GTPase |
| OPB37046 | T4742_S00005.73 | dynamitin p50 |
| OPB38119 | T4742_S00002.166 | dynein heavy chain |

| | | |
|---|---|---|
| OPB44325 | T4742_S00003.596 | dynein light chain |
| OPB45304 | T4742_S00010.215 | dynein light intermediate chain (DLIC). |
| OPB46627 | T4742_S00009.69 | E3 SUMO-protein ligase pli1 [Colletotrichum chlorophyti] |
| OPB42295 | T4742_S00004.397 | E3 ubiquitin-protein ligase BRE1 (EC 6.3.2.-) [BRE1] |
| OPB45535 | T4742_S00010.446 | E3 ubiquitin-protein ligase listerin [Beauveria bassiana D1-5] |
| OPB36389 | T4742_S00007.106 | E3 ubiquitin-protein ligase, IQ and HECT domain |
| OPB46096 | T4742_S00008.163 | E3 ubiquitin-protein ligase/Putative upstream regulatory element binding protein |
| OPB39395 | T4742_S00006.455 | E3-binding protein |
| OPB36076 | T4742_S00024.37 | early growth response protein |
| OPB36666 | T4742_S00007.384 | effector of Sec4; Sro7 / Sro77 |
| OPB42878 | T4742_S00012.115 | efflux pump antibiotic resistance protein |
| OPB38741 | T4742_S00002.790 | EGF-like region, conserved site |
| OPB39068 | T4742_S00006.127 | EGF-like region, conserved site |
| OPB41421 | T4742_S00011.264 | EGF-like region, conserved site |
| OPB38351 | T4742_S00002.399 | EH domain-containing protein,End3, involved in actin cytoskeletal organization and cell wall morphogenesis |
| OPB39012 | T4742_S00006.71 | eIF2 gamma subunit. |
| OPB40519 | T4742_S00001.598 | elastinolytic metalloproteinase |
| OPB38060 | T4742_S00002.107 | electron transfer flavoprotein domain-containing protein |
| OPB43490 | T4742_S00016.10 | Electron transfer flavoprotein, alpha subunit |
| OPB36390 | T4742_S00007.107 | Electron transfer flavoprotein-ubiquinone oxidoreductase |
| OPB45287 | T4742_S00010.198 | electron transport protein, probably involved in cytochrome C assembly |
| OPB36497 | T4742_S00007.214 | elongation factor 1-alpha |
| OPB41882 | T4742_S00018.205 | elongation factor 1-beta |
| OPB38100 | T4742_S00002.147 | Elongation factor 2 |
| OPB35935 | T4742_S00025.8 | elongation factor G (Tu) and the LepA membrane protein family. |
| OPB46718 | T4742_S00009.160 | elongation factor Tu (G). |
| OPB40856 | T4742_S00001.937 | elongation factor Tu (GTP binding). |
| OPB40155 | T4742_S00001.234 | elongation of fatty acids protein [Verticillium dahliae VdLs.17] |
| OPB43256 | T4742_S00022.60 | elongator complex protein |
| OPB42505 | T4742_S00004.607 | Elongin C, Global genomic repair |
| OPB37737 | T4742_S00017.4 | Emg1p, a protein required for the maturation of the 18S rRNA and for 40S ribosome production; associated with spindle/microtubules; nuclear localization depends on physical interaction with |
| OPB37443 | T4742_S00005.470 | Emp24/gp25L/p24 family protein Erp1 |
| OPB37406 | T4742_S00005.433 | Emp24/gp25L/p24 family protein Erp3 |
| OPB43657 | T4742_S00016.177 | endo alpha-1,4 polygalactosaminidase precusor |
| OPB40513 | T4742_S00001.592 | endochitinase CHI2 |
| OPB42194 | T4742_S00004.296 | endocytic protein, Ede1 |

| | | |
|---|---|---|
| OPB38006 | T4742_S00002.53 | endomembrane protein 70 |
| OPB47145 | T4742_S00009.587 | endomembrane protein 70 |
| OPB37464 | T4742_S00005.491 | endonuclease III-like excision repair N-glycosylase involved in the repair of DNA base damage |
| OPB44842 | T4742_S00013.147 | Endonuclease/exonuclease/phosphatase |
| OPB40305 | T4742_S00001.384 | endonuclease/exonuclease/phosphatase family protein |
| OPB46937 | T4742_S00009.379 | endonuclease/exonuclease/phosphatase family protein |
| OPB44944 | T4742_S00013.249 | endonuclease/Exonuclease/phosphatase, putative |
| OPB45016 | T4742_S00013.321 | endonuclease/exonuclease/phosphatase, putative |
| OPB44173 | T4742_S00003.444 | Endoplasmatic reticulum oxidising protein Ero1 |
| OPB43108 | T4742_S00012.345 | Endoplasmic reticulum protein EP58 |
| OPB40257 | T4742_S00001.336 | Endoplasmic reticulum vesicle protein, Erv25 |
| OPB42366 | T4742_S00004.468 | endoribonuclease L-PSP |
| OPB43762 | T4742_S00003.33 | Endoribonuclease L-PSP |
| OPB43792 | T4742_S00003.63 | Endoribonuclease L-PSP |
| OPB44716 | T4742_S00013.20 | Endoribonuclease L-PSP |
| OPB45902 | T4742_S00014.238 | endoribonuclease L-PSP |
| OPB39870 | T4742_S00015.214 | Endoribonuclease YSH1 (mRNA 3'-end-processing protein YSH1) |
| OPB41962 | T4742_S00004.64 | endosomal peripheral membrane protein [Trichoderma reesei RUT C-30] |
| OPB43503 | T4742_S00016.23 | endosomal t-SNARE Syn8 |
| OPB36470 | T4742_S00007.187 | Endosome-associated ubiquitin isopeptidase |
| OPB36042 | T4742_S00024.3 | enolase |
| OPB39612 | T4742_S00006.672 | enolase |
| OPB37297 | T4742_S00005.324 | enoyl-CoA hydratase |
| OPB37423 | T4742_S00005.450 | enoyl-CoA hydratase |
| OPB37655 | T4742_S00005.682 | enoyl-CoA hydratase/isomerase |
| OPB40906 | T4742_S00001.987 | enoyl-CoA hydratase/isomerase |
| OPB41132 | T4742_S00020.156 | enoyl-CoA hydratase/isomerase |
| OPB41221 | T4742_S00011.62 | enoyl-CoA hydratase/isomerase |
| OPB42736 | T4742_S00004.842 | enoyl-CoA hydratase/isomerase |
| OPB44971 | T4742_S00013.276 | enoyl-CoA hydratase/isomerase |
| OPB45141 | T4742_S00010.48 | enoyl-CoA hydratase/isomerase |
| OPB36729 | T4742_S00007.447 | enoyl-CoA hydratase/isomerase |
| OPB37667 | T4742_S00005.694 | enoyl-CoA hydratase/isomerase |
| OPB39321 | T4742_S00006.381 | enoyl-CoA hydratase/isomerase |
| OPB44105 | T4742_S00003.376 | enoyl-CoA hydratase/isomerase |
| OPB44432 | T4742_S00003.704 | enoyl-CoA hydratase/isomerase |

| | | |
|---|---|---|
| OPB44505 | T4742_S00003.777 | enoyl-CoA hydratase/isomerase |
| OPB45561 | T4742_S00010.472 | enoyl-CoA hydratase/isomerase |
| OPB45719 | T4742_S00014.53 | enoyl-CoA hydratase/isomerase |
| OPB45397 | T4742_S00010.308 | enoyl-CoA hydratase/isomerase family protein |
| OPB40725 | T4742_S00001.805 | Enoyl-CoA hydratase/isomerase, putative |
| OPB36488 | T4742_S00007.205 | ENV1 photoreceptor |
| OPB35996 | T4742_S00023.31 | Epl1/Sm1 |
| OPB37788 | T4742_S00017.55 | Epl1/Sm1 |
| OPB38845 | T4742_S00002.894 | Epl1/Sm1 |
| OPB41292 | T4742_S00011.133 | Epl1/Sm1 |
| OPB41482 | T4742_S00011.325 | Epl1/Sm1 |
| OPB41716 | T4742_S00018.39 | Epl1/Sm1 |
| OPB41840 | T4742_S00018.163 | Epl1/Sm1 |
| OPB43804 | T4742_S00003.75 | Epl1/Sm1 |
| OPB43811 | T4742_S00003.82 | Epl1-like protein |
| OPB40329 | T4742_S00001.408 | epoxid hydrolase |
| OPB46442 | T4742_S00008.509 | epoxidase hydrolase |
| OPB43869 | T4742_S00003.140 | epoxidase subunit A |
| OPB38819 | T4742_S00002.868 | epoxide hydrolase |
| OPB40330 | T4742_S00001.409 | epoxide hydrolase |
| OPB40981 | T4742_S00020.5 | epoxide hydrolase |
| OPB41387 | T4742_S00011.228 | epoxide hydrolase |
| OPB45665 | T4742_S00010.576 | epoxide hydrolase |
| OPB46738 | T4742_S00009.180 | epoxide hydrolase |
| OPB46847 | T4742_S00009.289 | Epoxide hydrolase |
| OPB41809 | T4742_S00018.132 | epoxide hydrolase 1 |
| OPB46184 | T4742_S00008.251 | Epoxide hydrolase-like |
| OPB44818 | T4742_S00013.123 | epoxide hydrolase-like protein |
| OPB37139 | T4742_S00005.166 | Epsilon subunit of the coatomer complex, sec28 |
| OPB36536 | T4742_S00007.253 | Epsin-like protein,Ent2, involved inEndocytosis and actin patch assembly |
| OPB42754 | T4742_S00004.860 | EPTP domain containing protein [Acidovorax sp. CF316] |
| OPB44146 | T4742_S00003.417 | ER lumen protein retaining receptor Erd2 |
| OPB45762 | T4742_S00014.97 | ER lumen protein retaining receptor Erd2 |
| OPB37019 | T4742_S00005.46 | ER membrane protein. |
| OPB44339 | T4742_S00003.610 | ER membrane proteins involved in ER exit of secreted proteins. |
| OPB39940 | T4742_S00001.19 | ER protein BIG1 [Pochonia chlamydosporia 170] |

| | | |
|---|---|---|
| OPB36639 | T4742_S00007.357 | ER to golgi transport protein/RAD50-interacting protein 1. |
| OPB44352 | T4742_S00003.623 | ERAD-associated E3 ubiquitin-protein ligase component HRD3 |
| OPB45036 | T4742_S00013.341 | ERAD-associated E3 ubiquitin-protein ligase HRD1 |
| OPB37456 | T4742_S00005.483 | ERCC8 (CSA, CKN1) involved in transcription-coupled nucleotideExcision repair |
| OPB37911 | T4742_S00017.178 | ER-derived vesicles protein Erv14 |
| OPB43897 | T4742_S00003.168 | ER-derived vesicles protein Erv41 |
| OPB42906 | T4742_S00012.143 | ER-derived vesicles protein Erv46 |
| OPB41003 | T4742_S00020.27 | eRF1 (translation factor pelota). |
| OPB46403 | T4742_S00008.470 | Erg19p of Saccharomyces cerevisiae, a mevalonate pyrophosphate decarboxylase in theErgosterol biosynthetic pathway |
| OPB38209 | T4742_S00002.256 | ERG25 methylsterol desaturase, SUR2-type hydroxylase/desaturase, catalytic region |
| OPB46940 | T4742_S00009.382 | Erg27 3-keto steroid reductase |
| OPB45403 | T4742_S00010.314 | ERg28 protein |
| OPB40902 | T4742_S00001.983 | ERG5 C-22 sterol desaturase, a cytochrome P450 enzyme that catalyzes the formation of the C-22(23) double bond in the sterol side chain in ergosterol biosynthesis |
| OPB37985 | T4742_S00002.32 | ERG5 C-22 sterol desaturase, a cytochrome P450Enzyme that catalyzes the formation of the C-22(23) double bond in the sterol side chain inErgosterol biosynthesis |
| OPB38383 | T4742_S00002.431 | Erg8p of Saccharomyces cerevisiae; phosphomevalonate kinase |
| OPB38386 | T4742_S00002.434 | ESF2, involved in pre-18S rRNA processing. |
| OPB47099 | T4742_S00009.541 | esterase |
| OPB47151 | T4742_S00009.593 | esterase |
| OPB40922 | T4742_S00001.1003 | esterase family 9 |
| OPB38008 | T4742_S00002.55 | Esterase of the alpha-beta hydrolase superfamily (Neuropathy targetEsterase), contains cAMP-binding domains |
| OPB44693 | T4742_S00003.965 | esterase or lipase |
| OPB45956 | T4742_S00008.22 | Esterase, SGNH hydrolase-type |
| OPB42875 | T4742_S00012.112 | esterase/lipase |
| OPB43973 | T4742_S00003.244 | esterase/lipase |
| OPB46179 | T4742_S00008.246 | esterase/lipase |
| OPB36157 | T4742_S00021.74 | esterase/lipase [Trichoderma guizhouense] |
| OPB42761 | T4742_S00004.867 | Esterase/lipase superfamily protein |
| OPB37689 | T4742_S00005.716 | esterase/lipase, HGT |
| OPB41177 | T4742_S00011.17 | esterase/lipase, HGT |
| OPB45333 | T4742_S00010.244 | Esterase/lipase/thioesterase |
| OPB45480 | T4742_S00010.391 | Esterase/lipase/thioesterase |
| OPB42290 | T4742_S00004.392 | Esterase;too long in N-term; 214 was better |
| OPB39564 | T4742_S00006.624 | ethanolamine kinase, putative |
| OPB44066 | T4742_S00003.337 | Ethanolamine-P-transferase GPI11/PIG-F, involved in glycosylphosphatidylinositol anchor biosynthesis |
| OPB45003 | T4742_S00013.308 | eukaryotic and archaeal DNA primase |
| OPB43282 | T4742_S00019.15 | eukaryotic initiation factor 1A (eIF-1A). |

| | | |
|---|---|---|
| OPB39670 | T4742_S00015.14 | eukaryotic initiation factor 3, gamma subunit. |
| OPB40427 | T4742_S00001.506 | eukaryotic ribosome biogenesis protein 1 |
| OPB42025 | T4742_S00004.127 | eukaryotic translation initiation factor 2, subunit 1 alpha, 35kDa. |
| OPB46719 | T4742_S00009.161 | eukaryotic translation initiation factor 3 |
| OPB36856 | T4742_S00007.574 | eukaryotic translation initiation factor 3 subunit 11. |
| OPB46694 | T4742_S00009.136 | eukaryotic translation initiation factor 3 subunit 8, N-terminal. |
| OPB36714 | T4742_S00007.432 | eukaryotic translation initiation factor 3 subunit E |
| OPB39608 | T4742_S00006.668 | eukaryotic translation initiation factor 3 subunit EifCj, putative |
| OPB40192 | T4742_S00001.271 | eukaryotic translation initiation factor 3 subunit F |
| OPB41418 | T4742_S00011.261 | Eukaryotic translation initiation factor 3 subunit H (eIF3h), putative |
| OPB43002 | T4742_S00012.239 | eukaryotic translation initiation factor 3 subunit M |
| OPB39552 | T4742_S00006.612 | eukaryotic translation initiation factor 3, subunit 7. |
| OPB40044 | T4742_S00001.123 | Eukaryotic translation initiation factor 4E (eIF-4E) |
| OPB38984 | T4742_S00006.43 | Eukaryotic-like DNA topoisomerase I |
| OPB39630 | T4742_S00006.690 | exhibits similarity to ATP-dependent RNA helicases. |
| OPB38217 | T4742_S00002.264 | Exocyst component Ex084 |
| OPB43494 | T4742_S00016.14 | Exocyst component Exo70 |
| OPB37054 | T4742_S00005.81 | Exocyst component Sec10 |
| OPB46562 | T4742_S00009.2 | Exocyst component Sec15 |
| OPB41905 | T4742_S00004.7 | Exocyst component Sec3 |
| OPB38095 | T4742_S00002.142 | Exocyst component Sec5 |
| OPB42193 | T4742_S00004.295 | Exocyst component Sec6 |
| OPB37253 | T4742_S00005.280 | Exocyst component Sec8 |
| OPB36674 | T4742_S00007.392 | Exonuclease |
| OPB40179 | T4742_S00001.258 | exonuclease |
| OPB43982 | T4742_S00003.253 | exonuclease |
| OPB39202 | T4742_S00006.262 | exonuclease family protein |
| OPB36554 | T4742_S00007.271 | exonuclease, putative |
| OPB38944 | T4742_S00006.3 | exoribonuclease Dhp1 |
| OPB39647 | T4742_S00006.707 | exoribonuclease Dhp1 |
| OPB45213 | T4742_S00010.121 | Exoribonuclease, phosphorolytic domain 1 |
| OPB40215 | T4742_S00001.294 | exosome complex exonuclease RRP4 |
| OPB38689 | T4742_S00002.738 | exosome complex exonuclease RRP40, putative |
| OPB38587 | T4742_S00002.636 | Exosome-associated factor Rrp47/DNA strand repair C1D |
| OPB44083 | T4742_S00003.354 | expansin |
| OPB42942 | T4742_S00012.179 | EXS, C-terminal |

| | | |
|---|---|---|
| OPB43395 | T4742_S00019.128 | extracellular lipase |
| OPB39142 | T4742_S00006.202 | Extracellular membrane protein, 8-cysteine region, CFEM |
| OPB41914 | T4742_S00004.16 | Extracellular membrane protein, 8-cysteine region, CFEM |
| OPB38891 | T4742_S00002.940 | extracellular salicylate hydroxylase/monooxygenase, putative |
| OPB44711 | T4742_S00013.15 | Extradiol ring-cleavage dioxygenase, class III enzyme, subunit B |
| OPB39502 | T4742_S00006.562 | FACT complex protein (Facilitates chromatin transcription complex subunit SPT16) |
| OPB46643 | T4742_S00009.85 | FACT complex subunit pob3 |
| OPB37036 | T4742_S00005.63 | F-actin capping protein, beta subunit domain |
| OPB36356 | T4742_S00007.73 | F-acting capping protein, alpha subunit |
| OPB44650 | T4742_S00003.922 | FAD binding domain containing protein |
| OPB36063 | T4742_S00024.24 | FAD binding domain protein |
| OPB36084 | T4742_S00024.45 | FAD binding domain protein |
| OPB36276 | T4742_S00021.195 | FAD binding domain protein |
| OPB41126 | T4742_S00020.150 | FAD binding domain protein |
| OPB41877 | T4742_S00018.200 | FAD binding domain protein |
| OPB45930 | T4742_S00014.267 | FAD binding domain protein |
| OPB40482 | T4742_S00001.561 | FAD binding domain protein [Talaromyces cellulolyticus] |
| OPB36001 | T4742_S00023.36 | FAD binding domain-containing protein |
| OPB37582 | T4742_S00005.609 | FAD binding domain-containing protein |
| OPB42362 | T4742_S00004.464 | FAD binding domain-containing protein |
| OPB43211 | T4742_S00022.14 | FAD binding domain-containing protein |
| OPB41704 | T4742_S00018.26 | FAD binding domain-containing protein [Trichoderma harzianum] |
| OPB41535 | T4742_S00011.378 | FAD binding protein |
| OPB45066 | T4742_S00013.371 | FAD binding protein |
| OPB45498 | T4742_S00010.409 | FAD binding protein |
| OPB45666 | T4742_S00010.577 | FAD binding protein |
| OPB46505 | T4742_S00008.572 | FAD binding protein |
| OPB43789 | T4742_S00003.60 | FAD dependent oxidase |
| OPB40762 | T4742_S00001.843 | FAD dependent oxidoreductase |
| OPB43284 | T4742_S00019.17 | FAD dependent oxidoreductase |
| OPB44071 | T4742_S00003.342 | FAD dependent oxidoreductase |
| OPB44159 | T4742_S00003.430 | FAD dependent oxidoreductase |
| OPB45576 | T4742_S00010.487 | FAD dependent oxidoreductase |
| OPB45819 | T4742_S00014.154 | FAD dependent oxidoreductase |
| OPB38425 | T4742_S00002.473 | FAD dependent oxidoreductase [Trichoderma guizhouense] |
| OPB41708 | T4742_S00018.31 | FAD linked oxidase [Macrophomina phaseolina MS6] |

| | | |
|---|---|---|
| OPB42124 | T4742_S00004.226 | FAD linked oxidase [Macrophomina phaseolina MS6] |
| OPB46045 | T4742_S00008.111 | FAD linked oxidase domain protein |
| OPB39328 | T4742_S00006.388 | FAD linked oxidase domain-containing protein |
| OPB41265 | T4742_S00011.106 | FAD linked oxidase, N-terminal |
| OPB45428 | T4742_S00010.339 | FAD linked oxidase, N-terminal |
| OPB46282 | T4742_S00008.349 | FAD linked oxidase, N-terminal |
| OPB42157 | T4742_S00004.259 | FAD monooxygenase |
| OPB40582 | T4742_S00001.662 | FAD monooxygenase |
| OPB44503 | T4742_S00003.775 | FAD/NAD binding protein |
| OPB45521 | T4742_S00010.432 | FAD/NAD-linked reductase, dimerisation |
| OPB40311 | T4742_S00001.390 | FAD-binding dehydrogenase |
| OPB46329 | T4742_S00008.396 | FAD-binding domain and SignalP-predicted secretion signal. Distantly related to tre36816, tre34100, and tre39566. |
| OPB41027 | T4742_S00020.51 | FAD-binding domain-containing protein [Trichoderma reesei RUT C-30] |
| OPB46331 | T4742_S00008.398 | FAD-binding oxidase |
| OPB36798 | T4742_S00007.516 | FAD-binding protein |
| OPB41896 | T4742_S00018.219 | FAD-containing monooxygenase |
| OPB41770 | T4742_S00018.93 | FAD-dependen tmonooxygenase |
| OPB40917 | T4742_S00001.998 | FAD-dependent monooxygenase , putative |
| OPB41701 | T4742_S00018.23 | FAD-dependent monooxygenase , putative |
| OPB44805 | T4742_S00013.110 | FAD-dependent monooxygenase , putative |
| OPB44477 | T4742_S00003.749 | FAD-dependent oxidase [Aspergillus kawachii IFO 4308] |
| OPB36113 | T4742_S00021.29 | FAD-dependent oxidoreductase |
| OPB36479 | T4742_S00007.196 | FAD-dependent oxidoreductase |
| OPB37932 | T4742_S00017.199 | FAD-dependent oxidoreductase |
| OPB38870 | T4742_S00002.919 | FAD-dependent oxidoreductase |
| OPB42801 | T4742_S00012.38 | FAD-dependent oxidoreductase |
| OPB46203 | T4742_S00008.270 | FAD-dependent oxidoreductase |
| OPB46745 | T4742_S00009.187 | FAD-dependent oxidoreductase |
| OPB46869 | T4742_S00009.311 | FAD-dependent oxidoreductase |
| OPB41317 | T4742_S00011.158 | FAD-dependent pyridine nucleotide-disulfide oxidoreductase |
| OPB36660 | T4742_S00007.378 | FAD-dependent pyridine nucleotide-disulphide oxidoreductase |
| OPB40729 | T4742_S00001.809 | FAD-dependent pyridine nucleotide-disulphide oxidoreductase |
| OPB44784 | T4742_S00013.89 | FAD-dependent pyridine nucleotide-disulphide oxidoreductase |
| OPB36935 | T4742_S00007.653 | FAD-dependent sulfhydryl oxidase Erv1 |
| OPB39090 | T4742_S00006.149 | FAD-linked oxidase |
| OPB36801 | T4742_S00007.519 | FAD-monooxygenase |

| | | |
|---|---|---|
| OPB37156 | T4742_S00005.183 | FAD-monooxygenase |
| OPB38766 | T4742_S00002.815 | FAD-monooxygenase |
| OPB39804 | T4742_S00015.148 | FAD-monooxygenase |
| OPB43452 | T4742_S00019.185 | FAD-monooxygenase |
| OPB44594 | T4742_S00003.866 | FAD-monooxygenase |
| OPB43234 | T4742_S00022.37 | far upstream element-binding protein 2 |
| OPB46353 | T4742_S00008.420 | fatty acid desaturase |
| OPB36952 | T4742_S00007.670 | fatty acid desaturase |
| OPB43951 | T4742_S00003.222 | fatty acid desaturase |
| OPB38954 | T4742_S00006.13 | fatty acid elongase, 5 TM, GNS1/SUR4 membrane protein |
| OPB39864 | T4742_S00015.208 | fatty acid elongase-like protein |
| OPB39194 | T4742_S00006.254 | fatty acid hydroxylase |
| OPB42377 | T4742_S00004.479 | fatty acid hydroxylase, Cytb5, SUR2-type hydroxylase/desaturase, catalytic region |
| OPB38034 | T4742_S00002.81 | fatty acid synthase - candidate FAS2 |
| OPB38035 | T4742_S00002.82 | fatty acid synthase beta subunit [Includes: 3-hydroxypalmitoyl-[acyl-carrier-protein] dehydratase ;Enoyl-[acyl-carrier-protein] reductase [NADH] ;[Acyl-carrier-protein] acetyltransferase |
| OPB41855 | T4742_S00018.178 | fatty-acid amide hydrolase, putative |
| OPB38066 | T4742_S00002.113 | F-box and WD domain-containing protein |
| OPB47089 | T4742_S00009.531 | F-box domain containing protein |
| OPB46525 | T4742_S00008.592 | F-box domain protein |
| OPB44191 | T4742_S00003.462 | F-box domain-containing protein |
| OPB44695 | T4742_S00003.967 | F-box domain-containing protein |
| OPB39971 | T4742_S00001.50 | F-box protein involved in recycling plasma membrane proteins internalized by endocytosis |
| OPB42955 | T4742_S00012.192 | Fe superoxide dismutase |
| OPB46902 | T4742_S00009.344 | Fe(II)/2-oxoglutarate-dependent diounknown proteingenase |
| OPB39812 | T4742_S00015.156 | Fe/S biogenesis protein NfuA [Fusarium oxysporum f. sp. conglutinans race 2 54008] |
| OPB45515 | T4742_S00010.426 | Fe3+ ABC transporter periplasmic protein [Beauveria bassiana ARSEF 2860] |
| OPB39640 | T4742_S00006.700 | female and male fertility-1 [Trichoderma harzianum] |
| OPB43532 | T4742_S00016.52 | Ferredoxin reductase-like, C-terminal NADP-linked [Glarea lozoyensis ATCC 20868] |
| OPB36653 | T4742_S00007.371 | ferric reductase |
| OPB36784 | T4742_S00007.502 | ferric reductase |
| OPB37167 | T4742_S00005.194 | ferric reductase |
| OPB37952 | T4742_S00017.219 | ferric reductase |
| OPB39177 | T4742_S00006.237 | ferric reductase |
| OPB39963 | T4742_S00001.42 | ferric reductase |
| OPB43646 | T4742_S00016.166 | ferric reductase |
| OPB44797 | T4742_S00013.102 | ferric reductase |

| | | |
|---|---|---|
| OPB44926 | T4742_S00013.231 | ferric reductase |
| OPB45212 | T4742_S00010.120 | ferric reductase |
| OPB45245 | T4742_S00010.154 | ferric reductase |
| OPB41198 | T4742_S00011.38 | ferric-chelate reductase |
| OPB41298 | T4742_S00011.139 | ferric-chelate reductase |
| OPB42768 | T4742_S00012.5 | Ferritin/ribonucleotide reductase-like protein [Macrophomina phaseolina MS6] |
| OPB38028 | T4742_S00002.75 | ferrochelatase |
| OPB41257 | T4742_S00011.98 | ferrooxidoreductase |
| OPB41818 | T4742_S00018.141 | FeS cluster assembly scaffold IscU |
| OPB36686 | T4742_S00007.404 | FHA domain protein SNIP1, putative |
| OPB40587 | T4742_S00001.667 | filament-forming protein [Ustilaginoidea virens] |
| OPB46773 | T4742_S00009.215 | FKBP-type peptidyl-prolyl isomerase |
| OPB47116 | T4742_S00009.558 | FKBP-type peptidyl-prolyl isomerase, putative |
| OPB44001 | T4742_S00003.272 | flavin containing amine oxidoreductase |
| OPB45744 | T4742_S00014.79 | flavin containing polyamine oxidase |
| OPB36056 | T4742_S00024.17 | flavin-binding monooxygenase, putative |
| OPB44470 | T4742_S00003.742 | flavin-binding monooxygenase, putative |
| OPB46146 | T4742_S00008.213 | Flavin-binding monooxygenase-like family protein [Coccidioides posadasii C735 delta SOWgp] |
| OPB36451 | T4742_S00007.168 | Flavin-containing monooxygenase |
| OPB38840 | T4742_S00002.889 | Flavin-containing monooxygenase |
| OPB39957 | T4742_S00001.36 | Flavin-containing monooxygenase |
| OPB40756 | T4742_S00001.837 | Flavin-containing monooxygenase |
| OPB40836 | T4742_S00001.917 | Flavin-containing monooxygenase |
| OPB44335 | T4742_S00003.606 | Flavin-containing monooxygenase |
| OPB44753 | T4742_S00013.58 | Flavin-containing monooxygenase |
| OPB40657 | T4742_S00001.737 | flavin-containing monooxygenase FMO |
| OPB40092 | T4742_S00001.171 | Flavin-containing monooxygenase Fmo1 |
| OPB44546 | T4742_S00003.818 | Flavin-containing monooxygenase Fmo1 like |
| OPB45501 | T4742_S00010.412 | flavin-dependent halogenase O-methyltransferase bifunctional protein [Trichoderma guizhouense] |
| OPB36079 | T4742_S00024.40 | flavin-dependent monooxygenase |
| OPB36112 | T4742_S00021.28 | flavin-dependent monooxygenase |
| OPB36432 | T4742_S00007.149 | flavin-dependent monooxygenase |
| OPB37635 | T4742_S00005.662 | flavin-dependent monooxygenase |
| OPB40570 | T4742_S00001.649 | flavin-dependent monooxygenase |
| OPB41851 | T4742_S00018.174 | flavin-dependent monooxygenase |
| OPB45696 | T4742_S00014.30 | flavin-dependent monooxygenase |

| | | |
|---|---|---|
| OPB45874 | T4742_S00014.209 | flavin-dependent monooxygenase |
| OPB45891 | T4742_S00014.227 | flavin-dependent monooxygenase |
| OPB46226 | T4742_S00008.293 | flavin-dependent monooxygenase |
| OPB46350 | T4742_S00008.417 | flavin-dependent monooxygenase |
| OPB41412 | T4742_S00011.254 | Flavin-linked sulfhydryl oxidase |
| OPB38219 | T4742_S00002.266 | flavodoxin and radical SAM domain protein |
| OPB38063 | T4742_S00002.110 | flavodoxin domain containing protein |
| OPB38818 | T4742_S00002.867 | flavohemoglobin |
| OPB41330 | T4742_S00011.171 | flavohemoglobin |
| OPB40936 | T4742_S00001.1017 | flavonol reductase cinnamoyl-reductase [Umbilicaria pustulata] |
| OPB36815 | T4742_S00007.533 | Flavonol reductase/cinnamoyl-CoA reductase |
| OPB43784 | T4742_S00003.55 | Flavonol reductase/cinnamoyl-CoA reductase |
| OPB43674 | T4742_S00016.194 | flavonol synthase/flavanone 3-hydroxylase |
| OPB42890 | T4742_S00012.127 | Flavoprotein |
| OPB41011 | T4742_S00020.35 | flavoprotein monooxygenaase |
| OPB36543 | T4742_S00007.260 | flavoprotein monooxygenase |
| OPB37711 | T4742_S00005.739 | Flavoprotein monooxygenase |
| OPB40719 | T4742_S00001.799 | Flavoprotein monooxygenase |
| OPB42681 | T4742_S00004.787 | flavoprotein monooxygenase |
| OPB42725 | T4742_S00004.831 | Flavoprotein monooxygenase |
| OPB43260 | T4742_S00022.64 | flavoprotein monooxygenase |
| OPB43305 | T4742_S00019.38 | flavoprotein monooxygenase |
| OPB43396 | T4742_S00019.129 | flavoprotein monooxygenase |
| OPB43427 | T4742_S00019.160 | flavoprotein monooxygenase |
| OPB44729 | T4742_S00013.34 | Flavoprotein monooxygenase |
| OPB44735 | T4742_S00013.40 | Flavoprotein monooxygenase |
| OPB44754 | T4742_S00013.59 | flavoprotein monooxygenase |
| OPB44782 | T4742_S00013.87 | Flavoprotein monooxygenase |
| OPB45602 | T4742_S00010.513 | flavoprotein monooxygenase |
| OPB45879 | T4742_S00014.214 | flavoprotein monooxygenase |
| OPB46098 | T4742_S00008.165 | Flavoprotein monooxygenase |
| OPB46142 | T4742_S00008.209 | Flavoprotein monooxygenase |
| OPB45618 | T4742_S00010.529 | flavoprotein monooxygenase [Aspergillus arachidicola] |
| OPB40287 | T4742_S00001.366 | flavoprotein monooxygenase SOR5 |
| OPB42551 | T4742_S00004.654 | Flavoprotein monooxygenase, |
| OPB36429 | T4742_S00007.146 | Flavoprotein monooxygenase, putative |

| | | |
|---|---|---|
| OPB42976 | T4742_S00012.213 | Flavoprotein monooxygenase, putative |
| OPB43362 | T4742_S00019.95 | Flavoprotein monooxygenase, putative |
| OPB37175 | T4742_S00005.202 | flavoprotein monooxygenases |
| OPB37914 | T4742_S00017.181 | flavoprotein monooxygenases |
| OPB46241 | T4742_S00008.308 | flavoprotein monooxygenases |
| OPB43848 | T4742_S00003.119 | flavoprotein, putative |
| OPB41797 | T4742_S00018.120 | Flippase |
| OPB45817 | T4742_S00014.152 | flotillin domain protein [Trichoderma guizhouense] |
| OPB45752 | T4742_S00014.87 | fluG protein |
| OPB46592 | T4742_S00009.32 | FMN-binding flavin reductase family protein |
| OPB46810 | T4742_S00009.252 | FMN-dependent dehydrogenase |
| OPB38627 | T4742_S00002.676 | FOG: Hormone receptors |
| OPB44395 | T4742_S00003.666 | folyl polyglutamate synthase, cytosolic, related to S. cerevisiae Met7p |
| OPB44396 | T4742_S00003.667 | folylpolyglutamate synthase , putative |
| OPB43769 | T4742_S00003.40 | FoNIIA |
| OPB37794 | T4742_S00017.61 | Forkhead |
| OPB42234 | T4742_S00004.336 | Forkhead |
| OPB43630 | T4742_S00016.150 | Forkhead |
| OPB45948 | T4742_S00008.14 | forkhead family, related FHK1 of Acremonium chrysogenum |
| OPB40416 | T4742_S00001.495 | formamidase |
| OPB36489 | T4742_S00007.206 | formamidopyrimidine-DNA glycosylase |
| OPB37394 | T4742_S00005.421 | formate dehydrogenase |
| OPB40833 | T4742_S00001.914 | Formyl_trans_NFormyl transferase |
| OPB37590 | T4742_S00005.617 | Formyltetrahydrofolate hydrolase PurU |
| OPB45187 | T4742_S00010.94 | FRE ferric reductase-like transmembrane component |
| OPB36682 | T4742_S00007.400 | Frequency clock protein FRQ-1 |
| OPB43561 | T4742_S00016.81 | fructosamine kinase |
| OPB46584 | T4742_S00009.24 | Fructosamine kinase |
| OPB40135 | T4742_S00001.214 | Fructose-2,6-bisphosphatase |
| OPB43231 | T4742_S00022.34 | fructose-2,6-bisphosphate 2-phosphatase |
| OPB41946 | T4742_S00004.48 | fructose-bisphosphatase |
| OPB40445 | T4742_S00001.524 | fructose-bisphosphate aldolase |
| OPB36392 | T4742_S00007.109 | fructosyl amino acid oxidases |
| OPB37070 | T4742_S00005.97 | Ftt2 encoding a 14-3-3 class protein modulating protein-protein interactions. |
| OPB41789 | T4742_S00018.112 | fucose-specific lectin FleA |
| OPB36008 | T4742_S00023.43 | fumarate hydratase-like protein |

| | | |
|---|---|---|
| OPB38055 | T4742_S00002.102 | fumarate lyase |
| OPB44734 | T4742_S00013.39 | Fumarate lyase |
| OPB44184 | T4742_S00003.455 | fumarate reductase |
| OPB44730 | T4742_S00013.35 | Fumarylacetoacetase, C-terminal-like |
| OPB41367 | T4742_S00011.208 | Fumarylacetoacetate (FAA) hydrolase |
| OPB36573 | T4742_S00007.290 | fumarylacetoacetate hydrolase |
| OPB40223 | T4742_S00001.302 | fumarylacetoacetate hydrolase |
| OPB37050 | T4742_S00005.77 | fumarylacetoacetate hydrolase |
| OPB40722 | T4742_S00001.802 | fumarylacetoacetate hydrolase |
| OPB42935 | T4742_S00012.172 | fumarylacetoacetate hydrolase |
| OPB42753 | T4742_S00004.859 | fumarylacetoacetate hydrolase family protein |
| OPB43756 | T4742_S00003.27 | fumarylacetoacetate hydrolase family protein |
| OPB46215 | T4742_S00008.282 | fumarylacetoacetate hydrolase family protein |
| OPB36078 | T4742_S00024.39 | fumarylacetoacetate hydrolase/5-carboxymethyl-2-hydroxymuconate isomerase |
| OPB44640 | T4742_S00003.912 | fumarylacetoacetate hydrolase-like protein [Phialocephala scopiformis] |
| OPB37975 | T4742_S00002.22 | Fungal chitin synthase |
| OPB36661 | T4742_S00007.379 | Fungal specific transcription factor |
| OPB38853 | T4742_S00002.902 | Fungal specific transcription factor |
| OPB40751 | T4742_S00001.832 | Fungal specific transcription factor |
| OPB44731 | T4742_S00013.36 | Fungal specific transcription factor |
| OPB41366 | T4742_S00011.207 | fungal specific transcription factor domain protein |
| OPB38831 | T4742_S00002.880 | fungal specific transcription factor domain-containing protein |
| OPB37539 | T4742_S00005.566 | Fungal transcriptional regulatory protein, N-terminal |
| OPB37669 | T4742_S00005.696 | Fungal transcriptional regulatory protein, N-terminal |
| OPB37680 | T4742_S00005.707 | Fungal transcriptional regulatory protein, N-terminal |
| OPB38052 | T4742_S00002.99 | Fungal transcriptional regulatory protein, N-terminal |
| OPB38260 | T4742_S00002.307 | Fungal transcriptional regulatory protein, N-terminal |
| OPB38409 | T4742_S00002.457 | Fungal transcriptional regulatory protein, N-terminal |
| OPB38743 | T4742_S00002.792 | Fungal transcriptional regulatory protein, N-terminal |
| OPB38763 | T4742_S00002.812 | Fungal transcriptional regulatory protein, N-terminal |
| OPB40020 | T4742_S00001.99 | Fungal transcriptional regulatory protein, N-terminal |
| OPB40396 | T4742_S00001.475 | Fungal transcriptional regulatory protein, N-terminal |
| OPB40717 | T4742_S00001.797 | Fungal transcriptional regulatory protein, N-terminal |
| OPB40790 | T4742_S00001.871 | Fungal transcriptional regulatory protein, N-terminal |
| OPB40916 | T4742_S00001.997 | Fungal transcriptional regulatory protein, N-terminal |
| OPB41169 | T4742_S00011.9 | Fungal transcriptional regulatory protein, N-terminal |

| | | |
|---|---|---|
| OPB41170 | T4742_S00011.10 | Fungal transcriptional regulatory protein, N-terminal |
| OPB41184 | T4742_S00011.24 | Fungal transcriptional regulatory protein, N-terminal |
| OPB41348 | T4742_S00011.189 | Fungal transcriptional regulatory protein, N-terminal |
| OPB41533 | T4742_S00011.376 | Fungal transcriptional regulatory protein, N-terminal |
| OPB42584 | T4742_S00004.687 | Fungal transcriptional regulatory protein, N-terminal |
| OPB42674 | T4742_S00004.779 | Fungal transcriptional regulatory protein, N-terminal |
| OPB42766 | T4742_S00012.3 | Fungal transcriptional regulatory protein, N-terminal |
| OPB42778 | T4742_S00012.15 | Fungal transcriptional regulatory protein, N-terminal |
| OPB43733 | T4742_S00003.4 | Fungal transcriptional regulatory protein, N-terminal |
| OPB43747 | T4742_S00003.18 | Fungal transcriptional regulatory protein, N-terminal |
| OPB44501 | T4742_S00003.773 | Fungal transcriptional regulatory protein, N-terminal |
| OPB44622 | T4742_S00003.894 | Fungal transcriptional regulatory protein, N-terminal |
| OPB44681 | T4742_S00003.953 | Fungal transcriptional regulatory protein, N-terminal |
| OPB44709 | T4742_S00013.13 | Fungal transcriptional regulatory protein, N-terminal |
| OPB44717 | T4742_S00013.21 | Fungal transcriptional regulatory protein, N-terminal |
| OPB44844 | T4742_S00013.149 | Fungal transcriptional regulatory protein, N-terminal |
| OPB44847 | T4742_S00013.152 | Fungal transcriptional regulatory protein, N-terminal |
| OPB45294 | T4742_S00010.205 | Fungal transcriptional regulatory protein, N-terminal |
| OPB45990 | T4742_S00008.56 | Fungal transcriptional regulatory protein, N-terminal |
| OPB46067 | T4742_S00008.134 | Fungal transcriptional regulatory protein, N-terminal |
| OPB46143 | T4742_S00008.210 | Fungal transcriptional regulatory protein, N-terminal |
| OPB46185 | T4742_S00008.252 | Fungal transcriptional regulatory protein, N-terminal |
| OPB46196 | T4742_S00008.263 | Fungal transcriptional regulatory protein, N-terminal |
| OPB46197 | T4742_S00008.264 | Fungal transcriptional regulatory protein, N-terminal |
| OPB46198 | T4742_S00008.265 | Fungal transcriptional regulatory protein, N-terminal |
| OPB46290 | T4742_S00008.357 | Fungal transcriptional regulatory protein, N-terminal |
| OPB46384 | T4742_S00008.451 | Fungal transcriptional regulatory protein, N-terminal |
| OPB46490 | T4742_S00008.557 | Fungal transcriptional regulatory protein, N-terminal |
| OPB46514 | T4742_S00008.581 | Fungal transcriptional regulatory protein, N-terminal |
| OPB46707 | T4742_S00009.149 | Fungal transcriptional regulatory protein, N-terminal |
| OPB44310 | T4742_S00003.581 | G/T mismatch-specific thymine DNA glycosylase |
| OPB40027 | T4742_S00001.106 | G2/mitotic-specific cyclin CLB4 |
| OPB39580 | T4742_S00006.640 | G2/mitotic-specific cyclin-B [Neurospora crassa OR74A] |
| OPB37472 | T4742_S00005.499 | GadB Glutamate decarboxylase and related PLP-dependent proteins |
| OPB46258 | T4742_S00008.325 | GadB Glutamate decarboxylase and related PLP-dependent proteins |
| OPB42576 | T4742_S00004.679 | GAL1 Galactokinase |

| | | |
|---|---|---|
| OPB45404 | T4742_S00010.315 | GAL7 UDP glucose-1-phosphate galactosyltransferase |
| OPB44185 | T4742_S00003.456 | galactose permease |
| OPB44525 | T4742_S00003.797 | Galactose-binding like |
| OPB41769 | T4742_S00018.92 | galactoside-O-acetyltransferase |
| OPB43582 | T4742_S00016.102 | Gamma-glutamyl transpeptidase |
| OPB44801 | T4742_S00013.106 | Gamma-glutamyltranspeptidase |
| OPB45120 | T4742_S00010.27 | gamma-tubulin-like protein |
| OPB43617 | T4742_S00016.137 | GAP Gyp1 |
| OPB38073 | T4742_S00002.120 | GATA type transcriptional regulator |
| OPB38702 | T4742_S00002.751 | GATA type transcriptional regulator, related to NIT2/AreA |
| OPB37424 | T4742_S00005.451 | GATA type Zn finger |
| OPB40592 | T4742_S00001.672 | GCD |
| OPB37343 | T4742_S00005.370 | GCN2, a kinase phosphorylating the alpha subunit of the translation initiation factor eIF2. |
| OPB44245 | T4742_S00003.516 | GCN5 histone acetyltransferase, regulates cellulase gene expression |
| OPB40794 | T4742_S00001.875 | GCN5 N-acetyltransferase |
| OPB45071 | T4742_S00013.376 | GCN5 N-acetyltransferase |
| OPB36886 | T4742_S00007.604 | GCN5-N-acetyltransferase |
| OPB38436 | T4742_S00002.484 | GCN5-N-acetyltransferase |
| OPB46099 | T4742_S00008.166 | GCN5-N-Acetyltransferase |
| OPB41748 | T4742_S00018.71 | GCN5-N-acetyltransferase activity |
| OPB41389 | T4742_S00011.230 | GCN5-related acetyltransferase |
| OPB41846 | T4742_S00018.169 | GCN5-related acetyltransferase |
| OPB42898 | T4742_S00012.135 | GCN5-related acetyltransferase |
| OPB47107 | T4742_S00009.549 | GCN5-related acetyltransferase |
| OPB36723 | T4742_S00007.441 | GCN5-related N-acetyltransferase |
| OPB36863 | T4742_S00007.581 | GCN5-related N-acetyltransferase |
| OPB36914 | T4742_S00007.632 | GCN5-related N-acetyltransferase |
| OPB37318 | T4742_S00005.345 | GCN5-related N-acetyltransferase |
| OPB38368 | T4742_S00002.416 | GCN5-related N-acetyltransferase |
| OPB41999 | T4742_S00004.101 | GCN5-related N-acetyltransferase |
| OPB43361 | T4742_S00019.94 | GCN5-related N-acetyltransferase |
| OPB43749 | T4742_S00003.20 | GCN5-related N-acetyltransferase |
| OPB43990 | T4742_S00003.261 | GCN5-related N-acetyltransferase |
| OPB44017 | T4742_S00003.288 | GCN5-related N-acetyltransferase |
| OPB44539 | T4742_S00003.811 | GCN5-related N-acetyltransferase |
| OPB44697 | T4742_S00013.1 | GCN5-related N-acetyltransferase |

| | | |
|---|---|---|
| OPB46500 | T4742_S00008.567 | GCN5-related N-acetyltransferase |
| OPB47054 | T4742_S00009.496 | GCN5-related N-acetyltransferase |
| OPB38451 | T4742_S00002.499 | GCN5-related N-acetyltransferase |
| OPB42662 | T4742_S00004.767 | GCN5-related N-acetyltransferase |
| OPB40247 | T4742_S00001.326 | GCN5-related N-acetyltransferase |
| OPB41960 | T4742_S00004.62 | GCN5-related N-acetyltransferase, putative |
| OPB40467 | T4742_S00001.546 | GCPR,  mPR-type |
| OPB40462 | T4742_S00001.541 | GCPR,  mPR-type group VIII |
| OPB37166 | T4742_S00005.193 | GCPR,  mPR-type VIII |
| OPB35980 | T4742_S00023.15 | GCPR,  mPR-type, family VIII |
| OPB42658 | T4742_S00004.763 | GCPR, VIII (PAQR, HlyIII family, mPR-like) |
| OPB37601 | T4742_S00005.628 | GcvHGlycine cleavage system H protein (lipoate-binding) |
| OPB43702 | T4742_S00016.222 | GcvTGlycine cleavage system T protein |
| OPB38439 | T4742_S00002.487 | GDP dissociation inhibitor Gdi1 |
| OPB44920 | T4742_S00013.225 | GDP-forming succinate-CoA ligase, alpha subunit |
| OPB41768 | T4742_S00018.91 | GDP-forming succinate-CoA ligase, beta subunit |
| OPB43560 | T4742_S00016.80 | GDP-forming succinate-CoA ligase, beta subunit |
| OPB40646 | T4742_S00001.726 | GDP-fucose transporter (putative) |
| OPB40590 | T4742_S00001.670 | GDP-mannose a-mannosyltransferases;Distant relative |
| OPB35988 | T4742_S00023.23 | GDP-mannose transporter |
| OPB44139 | T4742_S00003.410 | GDP-mannose transporter |
| OPB41378 | T4742_S00011.219 | GDSL lipase/acylhydrolase family protein |
| OPB44486 | T4742_S00003.758 | GDSL-like Lipase/Acylhydrolase |
| OPB45653 | T4742_S00010.564 | GDSL-like Lipase/Acylhydrolase |
| OPB43034 | T4742_S00012.271 | gelsolin |
| OPB39924 | T4742_S00001.3 | Gelsolin region |
| OPB44720 | T4742_S00013.24 | General substrate transporter |
| OPB40220 | T4742_S00001.299 | gentisate 1,2-dioxygenase |
| OPB46225 | T4742_S00008.292 | gentisate 1,2-dioxygenase |
| OPB42773 | T4742_S00012.10 | gentisate 1,2-dioxygenase [Cladophialophora psammophila CBS 110553] |
| OPB45741 | T4742_S00014.76 | geranygeranyltransferase Bet4, required for vesicle traffic between ER and Golgi |
| OPB44123 | T4742_S00003.394 | geranylgeranyl diphosphate synthase, related to N. crassa albino-3 |
| OPB42990 | T4742_S00012.227 | geranylgeranyltransferase beta subunit |
| OPB47049 | T4742_S00009.491 | germinal center kinase, related to S. cerevisiae Kic1p |
| OPB40151 | T4742_S00001.230 | germinal center kinase, related to S. cerevisiae Sps1p |
| OPB43418 | T4742_S00019.151 | GFO_IDH_MocA dehydrogenase |

| | | |
|---|---|---|
| OPB37901 | T4742_S00017.168 | GH 2ß-glycosidase |
| OPB40484 | T4742_S00001.563 | GH protein [Trichoderma guizhouense] |
| OPB39337 | T4742_S00006.397 | GH1 ß-glucosidase BGL2/CEL1a |
| OPB36831 | T4742_S00007.549 | GH1 ß-glucosidase CEL1b |
| OPB40690 | T4742_S00001.770 | GH10 endo-ß-1,4-xylanase XYN3 |
| OPB43436 | T4742_S00019.169 | GH105/GH88 glycosyl hydrolase |
| OPB40663 | T4742_S00001.743 | GH106 protein |
| OPB40303 | T4742_S00001.382 | GH109 protein |
| OPB41822 | T4742_S00018.145 | GH109 protein |
| OPB44867 | T4742_S00013.172 | GH109 protein |
| OPB46247 | T4742_S00008.314 | GH109 protein |
| OPB44639 | T4742_S00003.911 | GH109 α-N-acetylgalactosaminidase |
| OPB46129 | T4742_S00008.196 | GH109 α-N-acetylgalactosaminidase |
| OPB46017 | T4742_S00008.83 | GH11 endo-ß-1,4-xylanase XYN1 |
| OPB36320 | T4742_S00007.37 | GH115 xylan-α-1,2-glucuronidase or α-(4-O-methyl)-glucuronidase |
| OPB42586 | T4742_S00004.689 | GH12 endo-1,4-beta-glucanase |
| OPB37643 | T4742_S00005.670 | GH12 endo-ß-1,4-glucanase |
| OPB41394 | T4742_S00011.236 | GH12 endo-ß-1,4-glucanase |
| OPB45807 | T4742_S00014.142 | GH125 protein |
| OPB43334 | T4742_S00019.67 | GH127 ß-arabinofuranosidase |
| OPB38565 | T4742_S00002.614 | GH128 protein |
| OPB40394 | T4742_S00001.473 | GH128 protein |
| OPB44862 | T4742_S00013.167 | GH128 protein |
| OPB36138 | T4742_S00021.55 | GH128 ß-1,3-glucanase, putative |
| OPB45584 | T4742_S00010.495 | GH13 |
| OPB43200 | T4742_S00022.3 | GH13 1,4-α-glucan branching enzyme |
| OPB47086 | T4742_S00009.528 | GH13 glycogen debranching enyzme |
| OPB38777 | T4742_S00002.826 | GH13 α-amylase |
| OPB38773 | T4742_S00002.822 | GH13 α-glucosidase |
| OPB37044 | T4742_S00005.71 | GH13 α-glucosidase/oligo α-glucosidase |
| OPB38390 | T4742_S00002.438 | GH132 glycoside hydrolase |
| OPB41934 | T4742_S00004.36 | GH15 alpha-glycosidase |
| OPB42498 | T4742_S00004.600 | GH15 glucamylase with starch binding domain |
| OPB38302 | T4742_S00002.349 | GH16 |
| OPB38985 | T4742_S00006.44 | GH16 cell wall glucanosyltransferase |
| OPB40888 | T4742_S00001.969 | GH16 cell wall glucanosyltransferase |

| | | |
|---|---|---|
| OPB41971 | T4742_S00004.73 | GH16 cell wall glucanosyltransferase |
| OPB45234 | T4742_S00010.143 | GH16 endo-1,3-β-D-glucosidase/1,3-glucan binding protein |
| OPB42708 | T4742_S00004.814 | GH16 endo-1,3-β-glucanase |
| OPB38965 | T4742_S00006.24 | GH16 glucan endo-1,3(4)-β-D-glucosidase |
| OPB40363 | T4742_S00001.442 | GH16 glucan endo-1,3(4)-β-D-glucosidase |
| OPB41264 | T4742_S00011.105 | GH16 glucan endo-1,3(4)-β-D-glucosidase |
| OPB43060 | T4742_S00012.297 | GH16 glucan endo-1,3(4)-β-D-glucosidase |
| OPB46012 | T4742_S00008.78 | GH16 glucan endo-1,3-1,4-β-D-glucosidase |
| OPB37104 | T4742_S00005.131 | GH16 ß-1,3/4-glucanase |
| OPB45960 | T4742_S00008.26 | GH16 ß-glycosidase (endo-beta-1,3(4)-β-D-glucanase) |
| OPB41331 | T4742_S00011.172 | GH17  glucan endo-1,3-β-glucosidase |
| OPB38838 | T4742_S00002.887 | GH17 glucan 1,3 β-glucosidase/ glucan endo-1,3-β-glucosidase |
| OPB37059 | T4742_S00005.86 | GH17 glucan 1,3-β-glucosidase |
| OPB38054 | T4742_S00002.101 | GH17 glucan endo-1,3-β-glucosidase |
| OPB41278 | T4742_S00011.119 | GH17 ß-(1-6) glucan synthase [Cladophialophora carrionii] |
| OPB35984 | T4742_S00023.19 | GH18 |
| OPB41571 | T4742_S00011.414 | GH18 chitinase |
| OPB42807 | T4742_S00012.44 | GH18 chitinase C, CBM50-CBM50-CBM18-GH18; T. reesei GH18-9 |
| OPB42808 | T4742_S00012.45 | GH18 chitinase C, CBM50-CBM50-CBM18-GH18; T. reesei GH18-9 |
| OPB35995 | T4742_S00023.30 | GH18 chitinase CHI18-11 |
| OPB43505 | T4742_S00016.25 | GH18 chitinase Chi18-5 |
| OPB37282 | T4742_S00005.309 | GH18 chitinase CHI18-rel2 |
| OPB40095 | T4742_S00001.174 | GH18 chitinase TVC10 with CBM18 and CBM50 |
| OPB45516 | T4742_S00010.427 | GH18 chitinase TVC5, with CBM18 domain |
| OPB35921 | T4742_S00027.2 | GH18 class V chitinase |
| OPB46374 | T4742_S00008.441 | GH18 endo-N-acetyl-ß-D-glucosaminidase Endo T |
| OPB41061 | T4742_S00020.85 | GH18, chitinase |
| OPB42733 | T4742_S00004.839 | GH18, chitinase |
| OPB45433 | T4742_S00010.344 | GH18, chitinase CHI 18-16 [Trichoderma guizhouense] |
| OPB46103 | T4742_S00008.170 | GH18, chitinase CHI18-13 |
| OPB40629 | T4742_S00001.709 | GH18, chitinase CHI18-14 [Trichoderma guizhouense] |
| OPB37700 | T4742_S00005.727 | GH18, chitinase CHI18-15 group A |
| OPB41236 | T4742_S00011.77 | GH18, chitinase CHI18-17, CBM1 |
| OPB37839 | T4742_S00017.106 | GH18, chitinase CHI18-3 |
| OPB44662 | T4742_S00003.934 | GH18, chitinase CHI18-4 |
| OPB36499 | T4742_S00007.216 | GH18, chitinase CHI18-7 |

| | | |
|---|---|---|
| OPB40749 | T4742_S00001.830 | GH18_2 chitinase |
| OPB42446 | T4742_S00004.548 | GH18_B chitinase |
| OPB42356 | T4742_S00004.458 | GH18_C, killer toxin ? |
| OPB42885 | T4742_S00012.122 | GH2 beta-galactosidase |
| OPB41176 | T4742_S00011.16 | GH2 beta-mannosidase |
| OPB37664 | T4742_S00005.691 | GH2 Exo-β-D-glucosaminidase GLS93 |
| OPB42334 | T4742_S00004.436 | GH2 ß-galactosidase, intracellular |
| OPB38657 | T4742_S00002.706 | GH2 β-galactosidase/β-glucuronidase |
| OPB36282 | T4742_S00021.201 | GH2 β-mannosidase |
| OPB37808 | T4742_S00017.75 | GH2 β-mannosidase |
| OPB39410 | T4742_S00006.470 | GH2 β-mannosidase |
| OPB43763 | T4742_S00003.34 | GH2 β-mannosidase |
| OPB45605 | T4742_S00010.516 | GH20 exochitinase |
| OPB38804 | T4742_S00002.853 | GH20 N-acetyl-β-hexosaminidase |
| OPB39538 | T4742_S00006.598 | GH20 NAG1 |
| OPB40814 | T4742_S00001.895 | GH23 exo-ß-1,3-glucanase, distantly related |
| OPB43771 | T4742_S00003.42 | GH23 exo-ß-1,3-glucanase, distantly related |
| OPB42825 | T4742_S00012.62 | GH23 glucanase [Trichoderma guizhouense] |
| OPB38227 | T4742_S00002.274 | GH23 hydrolase |
| OPB46018 | T4742_S00008.84 | GH23 protein |
| OPB46444 | T4742_S00008.511 | GH24lysozyme |
| OPB41758 | T4742_S00018.81 | GH25 N,O-diacetylmuramidase |
| OPB41463 | T4742_S00011.306 | GH27 |
| OPB44779 | T4742_S00013.84 | GH27 |
| OPB44806 | T4742_S00013.111 | GH27 |
| OPB36281 | T4742_S00021.200 | GH27 α-galactosidase |
| OPB39851 | T4742_S00015.195 | GH27 α-galactosidase |
| OPB39855 | T4742_S00015.199 | GH27 α-galactosidase |
| OPB40654 | T4742_S00001.734 | GH27 α-galactosidase |
| OPB43865 | T4742_S00003.136 | GH27 α-D-galactosidase |
| OPB46961 | T4742_S00009.403 | GH27 α-galactosidase |
| OPB41843 | T4742_S00018.166 | GH28 |
| OPB46019 | T4742_S00008.85 | GH28 endo-polygalacturonase |
| OPB42767 | T4742_S00012.4 | GH28 endo-polygalacturonase [Aspergillus arachidicola] |
| OPB42884 | T4742_S00012.121 | GH28 endo-polygalacturonase [Fusarium fujikuroi] |
| OPB46541 | T4742_S00008.608 | GH28 exo-rhamnogalacturonase RGX1 |

| | | |
|---|---|---|
| OPB41736 | T4742_S00018.59 | GH3 β-glucosidase/glucan 1,4-β-glucosidase BGL3f |
| OPB36372 | T4742_S00007.89 | GH3 β-N-acetylglucosaminidase |
| OPB45603 | T4742_S00010.514 | GH3 β-N-acetylglucosaminidase |
| OPB43421 | T4742_S00019.154 | GH3 ß-xylosidase |
| OPB41313 | T4742_S00011.154 | GH3 β-glucosidase |
| OPB36250 | T4742_S00021.168 | GH3 β-glucosidase BGL1/CEL3a |
| OPB37244 | T4742_S00005.271 | GH3 β-glucosidase BGL3i |
| OPB39470 | T4742_S00006.530 | GH3 β-glucosidase BGL3j |
| OPB39055 | T4742_S00006.114 | GH3 β-glucosidase CEL3b |
| OPB41826 | T4742_S00018.149 | GH3 β-glucosidase CEL3c |
| OPB36181 | T4742_S00021.99 | GH3 β-glucosidase CEL3d |
| OPB36249 | T4742_S00021.167 | GH3 β-xylosidase BXL1 |
| OPB40840 | T4742_S00001.921 | GH3 β-xylosidase XYL3b |
| OPB44551 | T4742_S00003.823 | GH30 endo-β-1 6-galactanase |
| OPB43779 | T4742_S00003.50 | GH30 endo-β-1,4-xylanase |
| OPB45639 | T4742_S00010.550 | GH30 endo-β-1,4-xylanase XYN4 |
| OPB41026 | T4742_S00020.50 | GH30 glucan endo 1,6-β-glucanase |
| OPB40642 | T4742_S00001.722 | GH31 α-xylosidase/α-glucosidase |
| OPB37286 | T4742_S00005.313 | GH31 Glucosidase II alpha subunit GLS2 |
| OPB37662 | T4742_S00005.689 | GH31 α-glucosidase |
| OPB41830 | T4742_S00018.153 | GH31 α-glucosidase |
| OPB41632 | T4742_S00011.475 | GH35 β-galactosidase BGA1 |
| OPB44301 | T4742_S00003.572 | GH36 raffinose synthase domain protein |
| OPB41400 | T4742_S00011.242 | GH37 α,α-trehalase |
| OPB39511 | T4742_S00006.571 | GH37 α,α-trehalase |
| OPB43020 | T4742_S00012.257 | GH38 α-mannosidase |
| OPB36161 | T4742_S00021.78 | GH39 |
| OPB43333 | T4742_S00019.66 | GH39 β-irunidase |
| OPB46013 | T4742_S00008.79 | GH39 β-irunidase |
| OPB42759 | T4742_S00004.865 | GH43 |
| OPB36776 | T4742_S00007.494 | GH43 β-xylosidase/α-L-arabinofuranosidase |
| OPB46246 | T4742_S00008.313 | GH43 β-xylosidase/α-L-arabinofuranosidase |
| OPB40708 | T4742_S00001.788 | GH43 L-arabinofuranosidase/ß--xylosidase [Micromonospora eburnea] |
| OPB44077 | T4742_S00003.348 | GH43 β-xylosidase/α-L-arabinofuranosidase |
| OPB40224 | T4742_S00001.303 | GH45 endo-ß-1,3-glucanase |
| OPB36011 | T4742_S00023.46 | GH47 α-1,2-mannosidase |

| | | |
|---|---|---|
| OPB37089 | T4742_S00005.116 | GH47 α-1,2-mannosidase |
| OPB41941 | T4742_S00004.43 | GH47 α-1,2-mannosidase |
| OPB45248 | T4742_S00010.157 | GH47 α-1,2-mannosidase |
| OPB46874 | T4742_S00009.316 | GH47 α-1,2-mannosidase |
| OPB44416 | T4742_S00003.688 | GH47 α-mannosidase |
| OPB43431 | T4742_S00019.164 | GH5 |
| OPB44863 | T4742_S00013.168 | GH5 endo-ß-1,6-glucanase |
| OPB42102 | T4742_S00004.204 | GH5 endo-β-1,4-glucanase |
| OPB46546 | T4742_S00008.613 | GH5 endo-β-1,6-glucanase |
| OPB36358 | T4742_S00007.75 | GH5 glucan β-1,3-glucosidase |
| OPB46276 | T4742_S00008.343 | GH5 membrane bound endoglucanase CEL5b |
| OPB44552 | T4742_S00003.824 | GH5 ß-mannanase |
| OPB36847 | T4742_S00007.565 | GH5 β-glycosidase CEL5d |
| OPB42282 | T4742_S00004.384 | GH5 β-Mannanase MAN1 |
| OPB38106 | T4742_S00002.153 | GH54 alpha-L-arabinofuranosidase ABF3 [Trichoderma parareesei] |
| OPB41212 | T4742_S00011.52 | GH54 α-L-arabinofuranosidase ABF3 |
| OPB44331 | T4742_S00003.602 | GH55 |
| OPB39036 | T4742_S00006.95 | GH55 exo-1 3-β-glucanase GLUC78 |
| OPB37172 | T4742_S00005.199 | GH55 exo-1,3-β-glucanase |
| OPB45782 | T4742_S00014.117 | GH55 exo-ß-1,3-glucanase |
| OPB43367 | T4742_S00019.100 | GH55 β-1 3-glucanase |
| OPB42709 | T4742_S00004.815 | GH55 β-1,3-glucanase |
| OPB44855 | T4742_S00013.160 | GH55 β-1,3-glucanase |
| OPB43438 | T4742_S00019.171 | GH6 Cellobiohydrolase CEL6A/CBH2 |
| OPB37570 | T4742_S00005.597 | GH61 polysaccharide monooxygenase |
| OPB46655 | T4742_S00009.97 | GH61 polysaccharide monooxygenase CEL61b |
| OPB40801 | T4742_S00001.882 | GH62 α-L-arabinofuranosidase ABF2 |
| OPB37319 | T4742_S00005.346 | GH63 processing α-glucosidase |
| OPB39876 | T4742_S00015.220 | GH63 α-glucosidase |
| OPB45961 | T4742_S00008.27 | GH64 |
| OPB46229 | T4742_S00008.296 | GH64 |
| OPB45421 | T4742_S00010.332 | GH64 endo-1,3-β-glucanase |
| OPB39098 | T4742_S00006.157 | GH65 α,α-trehalase |
| OPB39965 | T4742_S00001.44 | GH67 α-Glucuronidase GLR1 |
| OPB40799 | T4742_S00001.880 | GH71 |
| OPB40898 | T4742_S00001.979 | GH71 |

| | | |
|---|---|---|
| OPB45538 | T4742_S00010.449 | GH71 alpha-1,3-glucanase/mutanase |
| OPB38608 | T4742_S00002.657 | GH71 α-1 3-glucanase |
| OPB41732 | T4742_S00018.55 | GH71 α-1 3-glucanase |
| OPB44599 | T4742_S00003.871 | GH71 α-1 3-glucanase |
| OPB39767 | T4742_S00015.111 | GH72 ß-(1-3) glucanosyltransferase |
| OPB43151 | T4742_S00012.388 | GH72 β-1 3-glucanosyltransferase |
| OPB44104 | T4742_S00003.375 | GH72 β-1 3-glucanosyltransferase |
| OPB44756 | T4742_S00013.61 | GH72 β-1 3-glucanosyltransferase |
| OPB44894 | T4742_S00013.199 | GH72 β-1 3-glucanosyltransferase |
| OPB46497 | T4742_S00008.564 | GH74 Xyloglucanase CEL74a |
| OPB40420 | T4742_S00001.499 | GH75 chitosanase |
| OPB41309 | T4742_S00011.150 | GH75 chitosanase |
| OPB42786 | T4742_S00012.23 | GH75 chitosanase |
| OPB45882 | T4742_S00014.217 | GH75 chitosanase |
| OPB38306 | T4742_S00002.353 | GH76 GPI-anchored α-1,6-mannanase |
| OPB37420 | T4742_S00005.447 | GH76 α-1,6-mannanase |
| OPB38186 | T4742_S00002.233 | GH76 α-1,6-mannanase |
| OPB43898 | T4742_S00003.169 | GH76 α-1,6-mannanase |
| OPB44070 | T4742_S00003.341 | GH76 α-1,6-mannanase |
| OPB45226 | T4742_S00010.135 | GH76 α-1,6-mannanase |
| OPB37823 | T4742_S00017.90 | GH76 α-mannosidase |
| OPB41755 | T4742_S00018.78 | GH76 α-mannosidase |
| OPB41187 | T4742_S00011.27 | GH78 alpha-L-rhamnosidase |
| OPB40850 | T4742_S00001.931 | GH78 α-L-rhamnosidase |
| OPB37565 | T4742_S00005.592 | GH79 |
| OPB40848 | T4742_S00001.929 | GH79 ß-glucuronidase |
| OPB43434 | T4742_S00019.167 | GH79 ß-glucuronidase |
| OPB42685 | T4742_S00004.791 | GH79 β-glucuronidase |
| OPB37508 | T4742_S00005.535 | GH81 endo-1,3-β-glucanase |
| OPB41037 | T4742_S00020.61 | GH88 d-4,5-unsaturated β-glucuronyl hydrolase |
| OPB40745 | T4742_S00001.826 | GH88 glycosidase |
| OPB40649 | T4742_S00001.729 | GH88 unsaturated glucuronyl hydrolase |
| OPB39391 | T4742_S00006.451 | GH89 α-N-acetylglucosaminidase |
| OPB42689 | T4742_S00004.795 | GH92 |
| OPB47137 | T4742_S00009.579 | GH92 protein |
| OPB36783 | T4742_S00007.501 | GH92 α-1,2-mannosidase |

| | | |
|---|---|---|
| OPB38097 | T4742_S00002.144 | GH92 α-1,2-mannosidase |
| OPB41973 | T4742_S00004.75 | GH92 α-1,2-mannosidase |
| OPB45471 | T4742_S00010.382 | GH92 α-1,2-mannosidase |
| OPB45613 | T4742_S00010.524 | GH92 α-1,2-mannosidase |
| OPB41880 | T4742_S00018.203 | GH93 exo-α-L-1,5-arabinanase |
| OPB47165 | T4742_S00009.607 | GH93 glycoside hydrolase family 93 [Sporothrix brasiliensis 5110] |
| OPB42883 | T4742_S00012.120 | GH95 |
| OPB44798 | T4742_S00013.103 | GH95 alpha-fucosidase [Alternaria alternata] |
| OPB40919 | T4742_S00001.1000 | GH95 α-L-fucosidase |
| OPB43881 | T4742_S00003.152 | GH95 α-L-fucosidase |
| OPB40852 | T4742_S00001.933 | GH99 protein |
| OPB38089 | T4742_S00002.136 | GlcNAc-1-P transferase ALG7 |
| OPB37404 | T4742_S00005.431 | glucan endo-1,3(4)-β-D-glucosidase |
| OPB41686 | T4742_S00018.8 | glucan endo-1,3(4)-β-D-glucosidase |
| OPB37573 | T4742_S00005.600 | Glucan synthesis regulatory protein |
| OPB41622 | T4742_S00011.465 | glucokinase |
| OPB45877 | T4742_S00014.212 | gluconate 5-dehydrogenase |
| OPB45998 | T4742_S00008.64 | Gluconate kinase |
| OPB45999 | T4742_S00008.65 | Gluconate kinase |
| OPB46818 | T4742_S00009.260 | gluconokinase, thermoresistant glucokinase family |
| OPB44540 | T4742_S00003.812 | gluconolactonase-like protein |
| OPB36996 | T4742_S00005.23 | glucosamine 6-phosphate synthetase |
| OPB36375 | T4742_S00007.92 | glucosamine-6-phosphate isomerase |
| OPB38733 | T4742_S00002.782 | glucosamine-6-P-N-acetyltransferase |
| OPB44891 | T4742_S00013.196 | glucose oxidase |
| OPB40051 | T4742_S00001.130 | glucose repressible protein Grg1 |
| OPB37698 | T4742_S00005.725 | Glucose/ribitol dehydrogenase |
| OPB41172 | T4742_S00011.12 | Glucose/ribitol dehydrogenase |
| OPB43797 | T4742_S00003.68 | Glucose/ribitol dehydrogenase |
| OPB44743 | T4742_S00013.48 | Glucose/ribitol dehydrogenase |
| OPB44794 | T4742_S00013.99 | Glucose/ribitol dehydrogenase |
| OPB45205 | T4742_S00010.113 | Glucose/ribitol dehydrogenase |
| OPB45779 | T4742_S00014.114 | Glucose/ribitol dehydrogenase |
| OPB39439 | T4742_S00006.499 | glucose-6-phosphate dehydrogenase |
| OPB40469 | T4742_S00001.548 | glucose-methanol-choline (gmc) oxidoreductase |
| OPB40911 | T4742_S00001.992 | glucose-methanol-choline (gmc) oxidoreductase [Aspergillus nomius NRRL 13137] |

| | | |
|---|---|---|
| OPB44871 | T4742_S00013.176 | Glucose-methanol-choline oxidoreductase, C-terminal |
| OPB36340 | T4742_S00007.57 | glucose-repressible protein |
| OPB36810 | T4742_S00007.528 | glucosidase 2 subunit beta [Trichoderma guizhouense] |
| OPB44367 | T4742_S00003.638 | glutamate carbounknown proteinpeptidase |
| OPB46300 | T4742_S00008.367 | glutamate decarbounknown proteinlase |
| OPB45684 | T4742_S00014.18 | Glutamate decarboxylase |
| OPB45850 | T4742_S00014.185 | glutamate decarboxylase |
| OPB37554 | T4742_S00005.581 | glutamate N-acetyltransferase precursor |
| OPB39743 | T4742_S00015.87 | glutamate synthase |
| OPB36983 | T4742_S00005.10 | Glutamate-1-semialdehyde aminotransferase |
| OPB39468 | T4742_S00006.528 | Glutamate-cysteine ligase |
| OPB42249 | T4742_S00004.351 | Glutamate-kinase |
| OPB43941 | T4742_S00003.212 | glutaminase A |
| OPB47057 | T4742_S00009.499 | glutamine amidotransferase |
| OPB37370 | T4742_S00005.397 | Glutamine amidotransferase, class-II |
| OPB41105 | T4742_S00020.129 | Glutamine Phosphoribosylpyrophosphate amidotransferase PurF |
| OPB46376 | T4742_S00008.443 | glutamine synthetase |
| OPB36271 | T4742_S00021.190 | glutamine synthetase [Fusarium oxysporum f. sp. vasinfectum 25433] |
| OPB45185 | T4742_S00010.92 | glutamine synthetase protein fluG |
| OPB44211 | T4742_S00003.482 | Glutaminylpeptide cyclotransferase |
| OPB46328 | T4742_S00008.395 | glutaminyl-trna synthetase. |
| OPB37495 | T4742_S00005.522 | Glutamyl/glutaminyl-tRNA synthetase, class Ic |
| OPB36721 | T4742_S00007.439 | glutamyl-tRNA amidotransferase subunit B in other fungi. |
| OPB40969 | T4742_S00001.1050 | glutamyl-tRNA synthetase, class Ic. |
| OPB36205 | T4742_S00021.123 | glutamyl-tRNA(Gln) amidotransferase |
| OPB38534 | T4742_S00002.583 | glutamyl-tRNA(Gln) amidotransferase |
| OPB40460 | T4742_S00001.539 | glutamyl-tRNA(Gln) amidotransferase |
| OPB45795 | T4742_S00014.130 | glutamyl-tRNA(Gln) amidotransferase subunit A |
| OPB41281 | T4742_S00011.122 | glutamyl-tRNA(Gln) amidotransferase subunit A [Trichoderma harzianum] |
| OPB43448 | T4742_S00019.181 | Glutarate-semialdehyde dehydrogenase DavD [Neonectria ditissima] |
| OPB43849 | T4742_S00003.120 | glutaredoxin |
| OPB43723 | T4742_S00016.243 | glutaredoxin |
| OPB37022 | T4742_S00005.49 | glutaredoxin domain-containing protein |
| OPB44832 | T4742_S00013.137 | glutaredoxin Grx1, putative |
| OPB39548 | T4742_S00006.608 | glutaredoxin-4 |
| OPB37360 | T4742_S00005.387 | Glutathione peroxidase |

| | | |
|---|---|---|
| OPB42195 | T4742_S00004.297 | glutathione reductase |
| OPB38487 | T4742_S00002.536 | glutathione S transferase |
| OPB41301 | T4742_S00011.142 | glutathione S transferase |
| OPB43400 | T4742_S00019.133 | glutathione S transferase |
| OPB46379 | T4742_S00008.446 | glutathione S transferase |
| OPB45400 | T4742_S00010.311 | glutathione S transferase, 2 TM |
| OPB45063 | T4742_S00013.368 | glutathione S transferase, microsomal. |
| OPB36324 | T4742_S00007.41 | glutathione S-transferase |
| OPB39107 | T4742_S00006.167 | glutathione S-transferase |
| OPB40666 | T4742_S00001.746 | glutathione S-transferase |
| OPB41302 | T4742_S00011.143 | glutathione S-transferase |
| OPB41949 | T4742_S00004.51 | Glutathione S-transferase |
| OPB42782 | T4742_S00012.19 | glutathione S-transferase |
| OPB42809 | T4742_S00012.46 | Glutathione S-transferase |
| OPB43821 | T4742_S00003.92 | Glutathione S-transferase |
| OPB45216 | T4742_S00010.124 | glutathione S-transferase |
| OPB46000 | T4742_S00008.66 | Glutathione S-transferase |
| OPB46186 | T4742_S00008.253 | glutathione S-transferase |
| OPB46305 | T4742_S00008.372 | glutathione S-transferase |
| OPB46544 | T4742_S00008.611 | Glutathione S-transferase |
| OPB46932 | T4742_S00009.374 | glutathione S-transferase |
| OPB40226 | T4742_S00001.305 | glutathione S-transferase |
| OPB41208 | T4742_S00011.48 | glutathione S-transferase |
| OPB42029 | T4742_S00004.131 | glutathione S-transferase |
| OPB44988 | T4742_S00013.293 | glutathione S-transferase |
| OPB37607 | T4742_S00005.634 | glutathione S-transferase [Trichoderma harzianum] |
| OPB41911 | T4742_S00004.13 | Glutathione S-transferase domain |
| OPB39577 | T4742_S00006.637 | glutathione S-transferase domain-containing protein |
| OPB46121 | T4742_S00008.188 | glutathione S-transferase GliG, putative |
| OPB46234 | T4742_S00008.301 | glutathione S-transferase Gst3 |
| OPB41351 | T4742_S00011.192 | glutathione S-transferase II |
| OPB44733 | T4742_S00013.38 | Glutathione S-transferase, C-terminal |
| OPB45215 | T4742_S00010.123 | Glutathione S-transferase, C-terminal |
| OPB42734 | T4742_S00004.840 | Glutathione S-transferase, C-terminal-like |
| OPB46988 | T4742_S00009.430 | glutathione S-transferase-like protein |
| OPB39351 | T4742_S00006.411 | glutathione synthase |

| | | |
|---|---|---|
| OPB46654 | T4742_S00009.96 | glutathione transferase |
| OPB37866 | T4742_S00017.133 | glutathione-dependent formaldehyde-activating |
| OPB36595 | T4742_S00007.312 | glutathione-dependent formaldehyde-activating enzyme |
| OPB38940 | T4742_S00002.989 | glutathione-dependent formaldehyde-activating enzyme |
| OPB43672 | T4742_S00016.192 | glutathione-dependent formaldehyde-activating enzyme |
| OPB45481 | T4742_S00010.392 | glutathione-dependent formaldehyde-activating enzyme |
| OPB36643 | T4742_S00007.361 | Glutathione-dependent formaldehyde-activating, GFA |
| OPB38807 | T4742_S00002.856 | Glutathione-dependent formaldehyde-activating, GFA |
| OPB44394 | T4742_S00003.665 | Glutathione-dependent formaldehyde-activating,GFA |
| OPB37709 | T4742_S00005.737 | glutathione-S-transferase |
| OPB40319 | T4742_S00001.398 | glutathione-S-transferase |
| OPB40640 | T4742_S00001.720 | glutathione-S-transferase |
| OPB40941 | T4742_S00001.1022 | glutathione-S-transferase |
| OPB42799 | T4742_S00012.36 | glutathione-S-transferase |
| OPB44549 | T4742_S00003.821 | glutathione-S-transferase |
| OPB45427 | T4742_S00010.338 | glutathione-S-transferase |
| OPB47114 | T4742_S00009.556 | glutathione-S-transferase |
| OPB41019 | T4742_S00020.43 | glycan biosynthesis protein (PigL), N-acetylglucosaminyl-phosphatidylinositol de-N-acetylase |
| OPB42895 | T4742_S00012.132 | Glyceraldehyde-3-phosphate dehydrogenase(GAPDH) |
| OPB42422 | T4742_S00004.524 | Glyceraldehyde-3-phosphate dehydrogenase, isozyme 2 |
| OPB39966 | T4742_S00001.45 | glycerate dehydrogenase |
| OPB45749 | T4742_S00014.84 | glycerate dehydrogenase |
| OPB40315 | T4742_S00001.394 | glycerol kinase |
| OPB44189 | T4742_S00003.460 | glycerol:H+ symporter (Gup1), putative |
| OPB36307 | T4742_S00007.24 | Glycerol-3-phosphate 1-O-acyltransferase/glycerone-phosphate O-acyltransferase |
| OPB42530 | T4742_S00004.632 | Glycerol-3-phosphate 1-O-acyltransferase/glycerone-phosphate O-acyltransferase |
| OPB44884 | T4742_S00013.189 | Glycerol-3-phosphate 1-O-acyltransferase/glycerone-phosphate O-acyltransferase |
| OPB40313 | T4742_S00001.392 | Glycerol-3-phosphate dehydrogenase |
| OPB47083 | T4742_S00009.525 | glycerol-3-phosphate dehydrogenase, NAD-dependent |
| OPB42977 | T4742_S00012.214 | glycerol-3-phosphate phosphatase, putative |
| OPB40388 | T4742_S00001.467 | glycerone kinase |
| OPB39783 | T4742_S00015.127 | glycerophosphodiester phosphodiesterase GDE1 |
| OPB37078 | T4742_S00005.105 | Glycerophosphoryl diester phosphodiesterase |
| OPB44661 | T4742_S00003.933 | glycerophosphoryl diester phosphodiesterase family protein |
| OPB39310 | T4742_S00006.370 | Glycine cleavage T protein (aminomethyl transferase), putative |
| OPB45275 | T4742_S00010.186 | glycine dehydrogenase |

| | | |
|---|---|---|
| OPB40134 | T4742_S00001.213 | glycogen synthase kinase 3 |
| OPB46133 | T4742_S00008.200 | Glycolate oxidase |
| OPB38046 | T4742_S00002.93 | glycolipid transfer protein |
| OPB45659 | T4742_S00010.570 | glycoside hydrolase family 10 protein |
| OPB46304 | T4742_S00008.371 | glycoside hydrolase family 11 protein |
| OPB46640 | T4742_S00009.82 | glycoside hydrolase family 18 protein/killer toxin alpha/beta |
| OPB42562 | T4742_S00004.665 | glycoside hydrolase family 2 protein |
| OPB44852 | T4742_S00013.157 | Glycoside hydrolase family 2, immunoglobulin-like beta-sandwich |
| OPB40664 | T4742_S00001.744 | glycoside hydrolase family 28 protein |
| OPB38105 | T4742_S00002.152 | glycoside hydrolase family 3 protein |
| OPB41188 | T4742_S00011.28 | glycoside hydrolase family 3 protein |
| OPB36162 | T4742_S00021.79 | glycoside hydrolase family 31 protein |
| OPB44482 | T4742_S00003.754 | Glycoside hydrolase family 38 |
| OPB40914 | T4742_S00001.995 | glycoside hydrolase family 5 protein |
| OPB38913 | T4742_S00002.962 | glycoside hydrolase family 55 protein |
| OPB40700 | T4742_S00001.780 | glycoside hydrolase family 67 protein |
| OPB45592 | T4742_S00010.503 | glycoside hydrolase family 75 protein |
| OPB40699 | T4742_S00001.779 | glycoside hydrolase family GH30 |
| OPB43840 | T4742_S00003.111 | Glycoside hydrolase, family 11 |
| OPB36725 | T4742_S00007.443 | Glycoside hydrolase, family 16 |
| OPB41191 | T4742_S00011.31 | Glycoside hydrolase, family 16 |
| OPB40957 | T4742_S00001.1038 | Glycoside hydrolase, family 18, catalytic domain |
| OPB42365 | T4742_S00004.467 | Glycoside hydrolase, family 2 |
| OPB40831 | T4742_S00001.912 | Glycoside hydrolase, family 3, N-terminal |
| OPB44723 | T4742_S00013.28 | Glycoside hydrolase, family 3, N-terminal |
| OPB44809 | T4742_S00013.114 | Glycoside hydrolase, family 30 |
| OPB44836 | T4742_S00013.141 | Glycoside hydrolase, family 36 |
| OPB37715 | T4742_S00005.743 | Glycoside hydrolase, family 4 |
| OPB46594 | T4742_S00009.35 | Glycoside hydrolase, family 45 |
| OPB36637 | T4742_S00007.355 | Glycoside hydrolase, family 47 |
| OPB44848 | T4742_S00013.153 | Glycoside hydrolase, family 47 |
| OPB37031 | T4742_S00005.58 | Glycoside hydrolase, family 5 |
| OPB38665 | T4742_S00002.714 | Glycoside hydrolase, family 61 |
| OPB45393 | T4742_S00010.304 | Glycoside hydrolase, family 71 |
| OPB36316 | T4742_S00007.33 | Glycoside hydrolase, family 81 |
| OPB38172 | T4742_S00002.219 | glycosyl transferase, family 35, glycogen phosphorylase 1 |

| | | |
|---|---|---|
| OPB38148 | T4742_S00002.195 | glycosylphosphatidylinositol transamidase GPI8 |
| OPB46154 | T4742_S00008.221 | Glycosylphosphatidylinositol-specific phospholipase C |
| OPB41423 | T4742_S00011.266 | glycosyltransferase family 20 protein |
| OPB45078 | T4742_S00013.383 | glycosyltransferase family 41 |
| OPB46293 | T4742_S00008.360 | glycosyltransferase family 69 |
| OPB40759 | T4742_S00001.840 | glycosyltransferase family 8 |
| OPB40644 | T4742_S00001.724 | glycosyltransferase, Glycosyltransferases not yet assigned to a family [Trichoderma guizhouense] |
| OPB42248 | T4742_S00004.350 | glycyl-tRNA synthase, alpha 2 dimer. |
| OPB44788 | T4742_S00013.93 | glyoxalase [Gaeumannomyces tritici R3-111a-1] |
| OPB46913 | T4742_S00009.355 | Glyoxalase I |
| OPB37576 | T4742_S00005.603 | Glyoxalase/bleomycin resistance protein/dioxygenase |
| OPB43652 | T4742_S00016.172 | Glyoxalase/bleomycin resistance protein/dioxygenase |
| OPB44728 | T4742_S00013.33 | Glyoxalase/extradiol ring-cleavage dioxygenase |
| OPB45500 | T4742_S00010.411 | Glyoxylase |
| OPB36130 | T4742_S00021.47 | GMC oxidoreductase |
| OPB36879 | T4742_S00007.597 | GMC oxidoreductase |
| OPB38507 | T4742_S00002.556 | GMC oxidoreductase |
| OPB41881 | T4742_S00018.204 | GMC oxidoreductase |
| OPB45654 | T4742_S00010.565 | GMC oxidoreductase |
| OPB46284 | T4742_S00008.351 | GMC oxidoreductase |
| OPB46560 | T4742_S00008.627 | GMC oxidoreductase |
| OPB45112 | T4742_S00010.19 | GMC oxidoreductase [Aspergillus arachidicola] |
| OPB40331 | T4742_S00001.410 | GMC oxidoreductase [Glonium stellatum] |
| OPB38907 | T4742_S00002.956 | GMC oxidoreductase family protein |
| OPB45536 | T4742_S00010.447 | GMGC protein kinase csk1 |
| OPB46597 | T4742_S00009.39 | GMP synthase GuA |
| OPB36444 | T4742_S00007.161 | GNAT family acetyltransferase |
| OPB38366 | T4742_S00002.414 | GNAT family acetyltransferase |
| OPB44356 | T4742_S00003.627 | GNAT family acetyltransferase |
| OPB43757 | T4742_S00003.28 | GNAT family N-acetyltransferase |
| OPB39250 | T4742_S00006.310 | Golgi apparatus membrane protein tvp-23 |
| OPB45140 | T4742_S00010.47 | Golgi complex component, cog1 |
| OPB43647 | T4742_S00016.167 | Golgi GDP-mannose transporter |
| OPB46407 | T4742_S00008.474 | Golgi integral membrane protein (Cln3) [Aschersonia aleyrodis RCEF 2490] |
| OPB41430 | T4742_S00011.273 | Golgi integral membrane protein Cln3 |
| OPB46165 | T4742_S00008.232 | Golgi matrix protein, rud3, involved in the structural organization of the cis-Golgi |

| | | |
|---|---|---|
| OPB45038 | T4742_S00013.343 | Golgi membrane protein, coy1, with similarity to mammalian CASP |
| OPB42972 | T4742_S00012.209 | Golgi reassembly stacking protein GRASP65 |
| OPB46471 | T4742_S00008.538 | G-patch domain-containing protein |
| OPB46722 | T4742_S00009.164 | GPCR |
| OPB45392 | T4742_S00010.303 | GPCR , contains RGS domain |
| OPB45758 | T4742_S00014.93 | GPCR , contains RGS domain |
| OPB41747 | T4742_S00018.70 | GPCR , group VI, contains RGS domain |
| OPB45028 | T4742_S00013.333 | GPCR family 2, secretin like, related to methionine sensing G-protein coupled receptor in Cryptococcus neoformans |
| OPB38606 | T4742_S00002.655 | GPCR III, related to A nidulans GprC |
| OPB35952 | T4742_S00025.25 | GPCR IV (nitrogen), related to N. crassa Stm1-like GPR-6 |
| OPB41513 | T4742_S00011.356 | GPCR IV, nitrogen sensor |
| OPB42103 | T4742_S00004.205 | GPCR VII, secretin like |
| OPB38215 | T4742_S00002.262 | GPCR XII |
| OPB45414 | T4742_S00010.325 | GPCR XIII, rhodopsin type |
| OPB43807 | T4742_S00003.78 | GPCR, secretin like |
| OPB46980 | T4742_S00009.422 | GPCR, secretin like |
| OPB47128 | T4742_S00009.570 | GPCR, secretin like |
| OPB39349 | T4742_S00006.409 | GPCR, STE3, mating type pheromone G-protein coupled receptor |
| OPB41683 | T4742_S00018.5 | GPCR, mating type pheromone G-protein coupled receptor |
| OPB45348 | T4742_S00010.259 | GPCR, mPR-like |
| OPB38098 | T4742_S00002.145 | GPCR, related to NCU07701 (Neurospora) |
| OPB46629 | T4742_S00009.71 | GPI ethanolamine phosphate transferase GPI7 |
| OPB41458 | T4742_S00011.301 | GPI ethanolamine phosphate transferase, putative |
| OPB37960 | T4742_S00002.7 | GPI inositol deacylase of the ER Bst1 |
| OPB42881 | T4742_S00012.118 | GPI secreted protein |
| OPB43867 | T4742_S00003.138 | GPI secreted protein |
| OPB36972 | T4742_S00007.690 | GPI transamidase component GAA1 |
| OPB40520 | T4742_S00001.599 | GPI transamidase component GPI16 |
| OPB44407 | T4742_S00003.678 | GPI transamidase subunit PIG-U |
| OPB44704 | T4742_S00013.8 | GPI-anchored mannosidases |
| OPB40235 | T4742_S00001.314 | GPI-anchored protein, potential CFEM domain |
| OPB45157 | T4742_S00010.64 | G-protein alpha subunit 1 GNA1 |
| OPB39471 | T4742_S00006.531 | G-protein alpha subunit 2 GNA2 |
| OPB38171 | T4742_S00002.218 | G-protein alpha subunit 3 GNA-3 |
| OPB41082 | T4742_S00020.106 | G-protein beta subunit |
| OPB44157 | T4742_S00003.428 | G-protein coupled receptor protein, contains Molluscan rhodopsine C-terminal tail, possibly involved in signal transduction |

| | | |
|---|---|---|
| OPB39384 | T4742_S00006.444 | G-protein gamma subunit |
| OPB40742 | T4742_S00001.823 | GroES-like protein [Pochonia chlamydosporia 170] |
| OPB40099 | T4742_S00001.178 | Growth-arrest-specific protein 2 |
| OPB37859 | T4742_S00017.126 | GrpB domain protein |
| OPB41427 | T4742_S00011.270 | Gß-WD40 protein |
| OPB43155 | T4742_S00012.392 | Gß-WD40 protein Ski8 (Sordaria) |
| OPB45858 | T4742_S00014.193 | GT 1 glycosyl transferase |
| OPB37989 | T4742_S00002.36 | GT 2, chitin synthase |
| OPB45805 | T4742_S00014.140 | GT 31 glycosyltransferase |
| OPB44011 | T4742_S00003.282 | GT 32 |
| OPB46992 | T4742_S00009.434 | GT alpha 1,2 mannosyltransferase ALG9 |
| OPB41864 | T4742_S00018.187 | GT distantly  glycosyltransferases, Glycosyltransferases not yet assigned to a family |
| OPB45235 | T4742_S00010.144 | GT Glycosyltransferases not yet assigned to a family |
| OPB36787 | T4742_S00007.505 | GT glycosyltransferases not yet assigned to a family, 3 TMs [Trichoderma guizhouense] |
| OPB40018 | T4742_S00001.97 | GT α-1,2-mannosyltransferase |
| OPB40989 | T4742_S00020.13 | GT α-1,2-mannosyltransferase |
| OPB43581 | T4742_S00016.101 | GT α-1,2-mannosyltransferase ALG11 |
| OPB43283 | T4742_S00019.16 | GT α-1,3-mannosyltransferase |
| OPB41446 | T4742_S00011.289 | GT α-1,3-mannosyltransferase ALG6 |
| OPB39001 | T4742_S00006.60 | GT α-1,6-mannosyltransferase |
| OPB41021 | T4742_S00020.45 | GT α-1,6-mannosyltransferase |
| OPB44116 | T4742_S00003.387 | GT α-1,6-mannosyltransferase |
| OPB45311 | T4742_S00010.222 | GT α-1,6-mannosyltransferase |
| OPB46257 | T4742_S00008.324 | GT α-1,6-mannosyltransferase |
| OPB37042 | T4742_S00005.69 | GT α-1,6-mannosyltransferase ALG12 |
| OPB45633 | T4742_S00010.544 | GT, glycosyl transferase; putative |
| OPB36795 | T4742_S00007.513 | GT1 glycosyltransferase |
| OPB38810 | T4742_S00002.859 | GT1 ß-glycosyltransferase |
| OPB43275 | T4742_S00019.8 | GT15 α-1,2-mannosyltransferase |
| OPB41402 | T4742_S00011.244 | GT17 β-1,4-mannosyl-glycoprotein β-1,4-N-acetylglucosaminyltransferase (EC 2.4.1.144). |
| OPB37988 | T4742_S00002.35 | GT2 Chitin synthase |
| OPB38369 | T4742_S00002.417 | GT2 chitin synthase |
| OPB39168 | T4742_S00006.228 | GT2 chitin synthase |
| OPB44140 | T4742_S00003.411 | GT2 Chitin synthase |
| OPB43967 | T4742_S00003.238 | GT2 chitin synthase chs3 |
| OPB38659 | T4742_S00002.708 | GT2 dolichyl-phosphate ß-glucosyltransferase |

| | | |
|---|---|---|
| OPB37644 | T4742_S00005.671 | GT2 glycosyltransferase |
| OPB41517 | T4742_S00011.360 | GT2 polysaccharide-forming ß-glycosyltransferase; distantly animal hyaluronan synthase |
| OPB44435 | T4742_S00003.707 | GT2 ß-glycosyltransferase, related to hyaluronan synthases |
| OPB36736 | T4742_S00007.454 | GT20 A,a-trehalose-phosphate synthase |
| OPB44519 | T4742_S00003.791 | GT20 Bifunctional trehalose-6-phosphate synthase/trehalose-6-phosphate phosphatase |
| OPB47034 | T4742_S00009.476 | GT20 Trehalose-6-phosphate synthase component TPS1 and related subunits |
| OPB38842 | T4742_S00002.891 | GT20 trehalose-6-phosphate synthase/trehalose-6-phosphate phosphatase |
| OPB42213 | T4742_S00004.315 | GT22 ALG9 mannosyltransferase |
| OPB45146 | T4742_S00010.53 | GT22 Dolichyl-phosphate-mannose a-mannosyltransferase |
| OPB37745 | T4742_S00017.12 | GT25 b-glycosyltransferases |
| OPB40158 | T4742_S00001.237 | GT3 glycogen synthase |
| OPB36443 | T4742_S00007.160 | GT31 ß-glycosyltransferases |
| OPB40242 | T4742_S00001.321 | GT31 ß-glycosyltransferases |
| OPB43116 | T4742_S00012.353 | GT31 galactosyltransferase |
| OPB41516 | T4742_S00011.359 | GT31 ß-glycosyltransferase |
| OPB42863 | T4742_S00012.100 | GT31 ß-glycosyltransferase |
| OPB43388 | T4742_S00019.121 | GT31 ß-glycosyltransferases |
| OPB41225 | T4742_S00011.66 | GT32 glycosyltransferase, related to A. fumigatus capsule polysaccharide biosynthesis |
| OPB46338 | T4742_S00008.405 | GT32 a-glycosyltransferase |
| OPB43375 | T4742_S00019.108 | GT32 protein |
| OPB41020 | T4742_S00020.44 | GT32 α-1,6-mannosyltransferase |
| OPB44399 | T4742_S00003.670 | GT32 α-1,6-mannosyltransferase |
| OPB44908 | T4742_S00013.213 | GT32 α-1,6-mannosyltransferase |
| OPB38966 | T4742_S00006.25 | GT32 α-glycosyltransferase |
| OPB45778 | T4742_S00014.113 | GT32protein |
| OPB38154 | T4742_S00002.201 | GT33 ß-1,4-mannosyltransferase |
| OPB46408 | T4742_S00008.475 | GT34 protein |
| OPB38723 | T4742_S00002.772 | GT39 Dolichyl-P-mannose:protein O-mannosyl transferase |
| OPB46878 | T4742_S00009.320 | GT39 Dolichyl-P-mannose:protein O-mannosyl transferase |
| OPB39724 | T4742_S00015.68 | GT39Dolichyl-P-mannose:protein O-mannosyl transferase |
| OPB40414 | T4742_S00001.493 | GT4 glycosyl transferase |
| OPB37681 | T4742_S00005.708 | GT4 α,α'-trehalose phosphorylase/synthase |
| OPB45720 | T4742_S00014.54 | GT4 α-1,3-mannosyltransferase |
| OPB45077 | T4742_S00013.382 | GT41 UDP-N-acetylglucosamin transferase |
| OPB37747 | T4742_S00017.14 | GT48 ß-1,3-glucan synthase |
| OPB44223 | T4742_S00003.494 | GT50 GPI mannosyltransferase 1 |

| | | |
|---|---|---|
| OPB46675 | T4742_S00009.117 | GT57 dolichyl-phosphate-glucose a-glucosyltransferase Alg8 |
| OPB47148 | T4742_S00009.590 | GT59 α-1,2 glucosyltransferase |
| OPB37970 | T4742_S00002.17 | GT62 α-1,6-mannosyltransferase |
| OPB43374 | T4742_S00019.107 | GT64 a-N-acetylhexosaminyltransferases |
| OPB44548 | T4742_S00003.820 | GT64 a-N-acetylhexosaminyltransferases |
| OPB39242 | T4742_S00006.302 | GT66, STT3 subunit of ER oligosaccharyltransferase |
| OPB46879 | T4742_S00009.321 | GT69 protein |
| OPB41587 | T4742_S00011.430 | GT69 α-1,3-mannosyltransferase CMT1 |
| OPB45100 | T4742_S00010.7 | GT71 α-1,3-mannosyltransferase |
| OPB44003 | T4742_S00003.274 | GT8 a-glycosyltransferases |
| OPB45046 | T4742_S00013.351 | GT8 glycogenin |
| OPB42339 | T4742_S00004.441 | GT90  protein |
| OPB37892 | T4742_S00017.159 | GT90 protein |
| OPB41778 | T4742_S00018.101 | GT90 protein |
| OPB46760 | T4742_S00009.202 | GTP binding nuclear protein Ran, member of the superfamily of RAS small GTPases, related to N. crassa GTP-binding nuclear protein SPI1 |
| OPB38313 | T4742_S00002.360 | GTP binding protein |
| OPB39377 | T4742_S00006.437 | GTP binding protein |
| OPB37339 | T4742_S00005.366 | GTP binding protein (Gtp1), putative |
| OPB45084 | T4742_S00013.389 | GTP cyclohydrolase |
| OPB42707 | T4742_S00004.813 | GTP cyclohydrolase 1 |
| OPB43803 | T4742_S00003.74 | GTP cyclohydrolase II |
| OPB45147 | T4742_S00010.54 | GTPase acitvating protein for Rho subfamily of RAS smallGTPases, related to S. cerevisiae SAC7p |
| OPB43937 | T4742_S00003.208 | GTPase activating factor (RasGAP) related to S. cerevisiae Bud2 |
| OPB37296 | T4742_S00005.323 | GTPase activating protein for Rab family members of Ras smallGTPases, related to S. cerevisiaeGyp7p |
| OPB41658 | T4742_S00011.501 | GTPase activating protein for RabGTPases (Ras superfamily of smallGTPases), related to S. cerevisiaeGYP2 |
| OPB37862 | T4742_S00017.129 | GTPase activator protein for Ras-likeGTPase (RasGAP) |
| OPB41092 | T4742_S00020.116 | GTPase FZO1, transmembrane location |
| OPB36962 | T4742_S00007.680 | GTPase involved in G-protein signaling in the adenylate cyclase activating pathway |
| OPB42989 | T4742_S00012.226 | GTPase regulator |
| OPB38628 | T4742_S00002.677 | GTPase Rsr1 (A. fumigatus) |
| OPB42211 | T4742_S00004.313 | GTPase with a role in regulation of membrane traffic, arl1; G protein of the Ras superfamily, ADP-ribosylation factor |
| OPB36982 | T4742_S00005.9 | GTPase, related to S. cerevisiae NuclearGTP-binding protein NUG1 (NuclearGTPase 1) |
| OPB39287 | T4742_S00006.347 | GTPase-activating protein (GAP) for Rab family members,Gyp5, involved in ER toGolgi trafficking |
| OPB37979 | T4742_S00002.26 | GTPase-activating protein gyp10, putative |
| OPB42646 | T4742_S00004.751 | GTPase-activating protein Msb3 (Sec4/Rabs) |
| OPB36551 | T4742_S00007.268 | GTP-binding nuclear protein Ran-related |

| | | |
|---|---|---|
| OPB36618 | T4742_S00007.336 | GTP-binding protein |
| OPB46734 | T4742_S00009.176 | GTP-binding protein |
| OPB37081 | T4742_S00005.108 | GTP-binding protein |
| OPB37521 | T4742_S00005.548 | GTP-binding protein GTR1 |
| OPB38389 | T4742_S00002.437 | GTP-binding protein involved in protein synthesis |
| OPB40473 | T4742_S00001.552 | GTP-binding protein YchF |
| OPB40498 | T4742_S00001.577 | GTP-binding protein, HSR1-related |
| OPB37923 | T4742_S00017.190 | GTP-binding protein, HSR1-related, member ofGTP1/OBG family |
| OPB44895 | T4742_S00013.200 | Guanine deaminase (GDEase), an aminohydrolase responsible for the conversion ofGuanine to xanthine and ammonia, the first step to utilizeGuanine as a nitrogen source. |
| OPB36724 | T4742_S00007.442 | Guanine nucleotide exchange factor |
| OPB39820 | T4742_S00015.164 | Guanine nucleotide exchange factor for Ras-likeGTPases (RasGEF) |
| OPB45958 | T4742_S00008.24 | Guanine nucleotide exchange factor for Ras-likeGTPases (RasGEF) |
| OPB43071 | T4742_S00012.308 | Guanine nucleotide exchange factor Sec12 |
| OPB37885 | T4742_S00017.152 | guanine nucleotide exchange factor synembryn, putative |
| OPB45061 | T4742_S00013.366 | Guanine nucleotide exchange factor, syt1 |
| OPB43126 | T4742_S00012.363 | guanine nucleotide exchange factor-like protein |
| OPB43225 | T4742_S00022.28 | Guanine nucleotide exchange factors (GEFs) |
| OPB44193 | T4742_S00003.464 | Guanine nucleotide-binding protein beta subunit-like protein, contains WD40-repeats |
| OPB37334 | T4742_S00005.361 | Guanosine diphosphatase, transport of GDP-mannose into the Golgi lumen |
| OPB36190 | T4742_S00021.108 | guanylate kinase |
| OPB43152 | T4742_S00012.389 | guanylate kinase |
| OPB46085 | T4742_S00008.152 | guanylate kinase, putative |
| OPB38727 | T4742_S00002.776 | Guanyl-nucleotide exchange factor Sec2 |
| OPB42423 | T4742_S00004.525 | GYF domain-containing protein |
| OPB36921 | T4742_S00007.639 | H/ACA ribonucleoprotein complex subunit 1 |
| OPB36158 | T4742_S00021.75 | H/K ATPase alpha subunit |
| OPB46475 | T4742_S00008.542 | H+ nucleoside cotransporter |
| OPB45818 | T4742_S00014.153 | haem peroxidase |
| OPB43868 | T4742_S00003.139 | Haem peroxidase, plant/fungal/bacterial |
| OPB36119 | T4742_S00021.36 | haem peroxidases. Secretion signal predicted by SignalP |
| OPB36449 | T4742_S00007.166 | half-sized ABC transporter |
| OPB37874 | T4742_S00017.141 | half-sized ABC transporter |
| OPB37921 | T4742_S00017.188 | half-sized ABC transporter |
| OPB42571 | T4742_S00004.674 | half-sized ABC transporter |
| OPB43177 | T4742_S00012.414 | half-sized ABC transporter |
| OPB44740 | T4742_S00013.45 | half-sized ABC transporter |

| | | |
|---|---|---|
| OPB45049 | T4742_S00013.354 | half-sized ABC transporter |
| OPB45163 | T4742_S00010.70 | half-sized ABC transporter |
| OPB42480 | T4742_S00004.582 | haloacid dehalogenase |
| OPB41433 | T4742_S00011.276 | Haloacid dehalogenase-like hydrolase |
| OPB42538 | T4742_S00004.641 | Haloacid dehalogenase-like hydrolase |
| OPB44136 | T4742_S00003.407 | Haloacid dehalogenase-like hydrolase |
| OPB39519 | T4742_S00006.579 | haloacid dehalogenase-like hydrolase |
| OPB40353 | T4742_S00001.432 | haloacid dehalogenase-like hydrolase, putative |
| OPB37347 | T4742_S00005.374 | Haloacid dehalogenase-like protein |
| OPB45632 | T4742_S00010.543 | Haloalkane dehalogenase |
| OPB42993 | T4742_S00012.230 | ham1, FSO1 protein |
| OPB39933 | T4742_S00001.12 | HAM1-like protein, probably related to DNA-repair |
| OPB39690 | T4742_S00015.34 | HAM3, straitin Pro11 (stalk rot protein) |
| OPB42936 | T4742_S00012.173 | -HAO-hydroxyanthranilic acid dioxygenase |
| OPB38016 | T4742_S00002.63 | HAP5 |
| OPB37902 | T4742_S00017.169 | hard-surface induced protein 5 |
| OPB42137 | T4742_S00004.239 | Has homology to tubuling binding cofactor A. |
| OPB45407 | T4742_S00010.318 | HD family hydrolase, putative |
| OPB38856 | T4742_S00002.905 | HEAT |
| OPB38884 | T4742_S00002.933 | HEAT |
| OPB40085 | T4742_S00001.164 | heat shock factor binding protein |
| OPB44471 | T4742_S00003.743 | heat shock factor STI1 that binds to and inhibits ATPase activity of HSP90 |
| OPB37976 | T4742_S00002.23 | heat shock protein (Hsp70 chaperone Hsp88) |
| OPB39845 | T4742_S00015.189 | Heat shock protein 70 |
| OPB43892 | T4742_S00003.163 | Heat shock protein DnaJ |
| OPB45770 | T4742_S00014.105 | Heat shock protein DnaJ |
| OPB38294 | T4742_S00002.341 | Heat shock protein DnaJ |
| OPB39307 | T4742_S00006.367 | Heat shock protein DnaJ |
| OPB43024 | T4742_S00012.261 | Heat shock protein DnaJ , HSP40 |
| OPB46809 | T4742_S00009.251 | Heat shock protein DnaJ, N-terminal |
| OPB42435 | T4742_S00004.537 | heat shock protein hsp60 mitochondrial precursor protein |
| OPB44982 | T4742_S00013.287 | Heat shock protein HSP80 |
| OPB36943 | T4742_S00007.661 | heat shock protein, Hsp40, DnaJ |
| OPB36004 | T4742_S00023.39 | HEC/Ndc80p family protein |
| OPB39286 | T4742_S00006.346 | HECT domain containing protein (E3 ubiquitin-protein ligase ) |
| OPB36034 | T4742_S00023.69 | HECT-domain-containing protein |

| | | |
|---|---|---|
| OPB39432 | T4742_S00006.492 | HECT-domain-containing protein |
| OPB42492 | T4742_S00004.594 | HECT-domain-containing protein |
| OPB42639 | T4742_S00004.744 | Helicase, C-terminal |
| OPB37452 | T4742_S00005.479 | helicase, DEAD-box superfamily |
| OPB44372 | T4742_S00003.643 | helicase, putative |
| OPB41624 | T4742_S00011.467 | helix-turn-helix domain-containing protein |
| OPB44387 | T4742_S00003.658 | helix-turn-helix transcription factor |
| OPB37140 | T4742_S00005.167 | Helix-turn-helix type 3 |
| OPB44222 | T4742_S00003.493 | Helix-turn-helix, AraC type |
| OPB46211 | T4742_S00008.278 | Heme peroxidase, unknown in Sordariomycetes |
| OPB43783 | T4742_S00003.54 | hemerythrin |
| OPB45525 | T4742_S00010.436 | hemerythrin HHE cation binding domain protein |
| OPB36399 | T4742_S00007.116 | Hemopexin |
| OPB39615 | T4742_S00006.675 | HET |
| OPB44633 | T4742_S00003.905 | HET |
| OPB36309 | T4742_S00007.26 | HET and Ankyrin domain protein |
| OPB46915 | T4742_S00009.357 | HET and Ankyrin domain protein |
| OPB36173 | T4742_S00021.90 | HET domain protein |
| OPB37118 | T4742_S00005.145 | HET domain protein |
| OPB38532 | T4742_S00002.581 | HET domain protein |
| OPB38564 | T4742_S00002.613 | HET domain protein |
| OPB39150 | T4742_S00006.210 | HET domain protein |
| OPB40550 | T4742_S00001.629 | HET domain protein |
| OPB40980 | T4742_S00020.4 | HET domain protein |
| OPB41277 | T4742_S00011.118 | HET domain protein |
| OPB41806 | T4742_S00018.129 | HET domain protein |
| OPB42832 | T4742_S00012.69 | HET domain protein |
| OPB43718 | T4742_S00016.238 | HET domain protein |
| OPB43885 | T4742_S00003.156 | HET domain protein |
| OPB47103 | T4742_S00009.545 | HET domain protein |
| OPB41052 | T4742_S00020.76 | HET domain protein |
| OPB44996 | T4742_S00013.301 | HET domain protein [Cordyceps brongniartii RCEF 3172] |
| OPB44882 | T4742_S00013.187 | HET domain protein [Trichoderma guizhouense] |
| OPB42880 | T4742_S00012.117 | HET domain protein, related to N. crassa pin-c3 |
| OPB41022 | T4742_S00020.46 | HET domain-containing protein [Trichoderma harzianum] |
| OPB36166 | T4742_S00021.83 | HET protein |

| | | |
|---|---|---|
| OPB39616 | T4742_S00006.676 | HET protein |
| OPB39617 | T4742_S00006.677 | HET protein |
| OPB40514 | T4742_S00001.593 | HET protein |
| OPB40553 | T4742_S00001.632 | HET protein |
| OPB41282 | T4742_S00011.123 | HET protein |
| OPB45319 | T4742_S00010.230 | HET protein |
| OPB45472 | T4742_S00010.383 | HET protein |
| OPB45712 | T4742_S00014.46 | HET protein |
| OPB45750 | T4742_S00014.85 | HET protein |
| OPB46912 | T4742_S00009.354 | HET protein |
| OPB47139 | T4742_S00009.581 | HET protein |
| OPB42332 | T4742_S00004.434 | HET protein |
| OPB46212 | T4742_S00008.279 | HET protein [Trichoderma parareesei] |
| OPB41133 | T4742_S00020.157 | HET, related to heterokaryon incompatibility protein het-6 [Fusarium fujikuroi IMI 58289] |
| OPB46666 | T4742_S00009.108 | HET, vegetative incompatibility protein HET-E-1 |
| OPB41010 | T4742_S00020.34 | HET, vegetative incompatibility protein HET-E-2 |
| OPB43186 | T4742_S00012.423 | HET, vegetative incompatibility protein HET-E-3 |
| OPB43187 | T4742_S00012.424 | HET, vegetative incompatibility protein HET-E-4 |
| OPB46931 | T4742_S00009.373 | HET, vegetative incompatibility protein HET-E-6 |
| OPB39184 | T4742_S00006.244 | HET/ankyrin |
| OPB38883 | T4742_S00002.932 | HET-6OR heterokaryon incompatibility protein (het-6OR allele) [Trichoderma guizhouense] |
| OPB44444 | T4742_S00003.716 | HET-containing protein, unknown |
| OPB36367 | T4742_S00007.84 | HET-domain-containing protein [Alternaria alternata] |
| OPB39960 | T4742_S00001.39 | HET-domain-containing protein [Colletotrichum tofieldiae] |
| OPB41358 | T4742_S00011.199 | HET-domain-containing protein, partial [Trichoderma reesei RUT C-30] |
| OPB37027 | T4742_S00005.54 | HET-E2C protein |
| OPB41784 | T4742_S00018.107 | Heterochromatin-associated protein HP1 and related CHROMO domain proteins |
| OPB46585 | T4742_S00009.25 | heterocompatibility domain protein |
| OPB38894 | T4742_S00002.943 | Heterokaryon incompatibility |
| OPB39582 | T4742_S00006.642 | Heterokaryon incompatibility |
| OPB40872 | T4742_S00001.953 | Heterokaryon incompatibility |
| OPB45771 | T4742_S00014.106 | Heterokaryon incompatibility |
| OPB46595 | T4742_S00009.37 | Heterokaryon incompatibility |
| OPB40111 | T4742_S00001.190 | Heterokaryon incompatibility [Penicillium italicum] |
| OPB42378 | T4742_S00004.480 | Heterokaryon incompatibility protein  HEC-C |
| OPB40492 | T4742_S00001.571 | heterokaryon incompatibility protein [Pochonia chlamydosporia 170] |

| | | |
|---|---|---|
| OPB42967 | T4742_S00012.204 | Heteromeric CCAAT factors |
| OPB46050 | T4742_S00008.116 | HET-R |
| OPB46236 | T4742_S00008.303 | HET-R |
| OPB42851 | T4742_S00012.88 | HET-s/LopB domain protein |
| OPB41649 | T4742_S00011.492 | hex1, encodes the major protein of the Woronin body forming a plug between fungal compartments. |
| OPB36419 | T4742_S00007.136 | hexaprenyl pyrophosphate synthase |
| OPB38234 | T4742_S00002.281 | hexokinase |
| OPB42146 | T4742_S00004.248 | hexokinase |
| OPB45526 | T4742_S00010.437 | hexokinase_1 |
| OPB43185 | T4742_S00012.422 | hexose transporter |
| OPB45549 | T4742_S00010.460 | hydrophobin HFB3 |
| OPB41013 | T4742_S00020.37 | HgmAhomogentisate ,-dioxygenase |
| OPB46582 | T4742_S00009.22 | HgmAhomogentisate ,-dioxygenase |
| OPB42842 | T4742_S00012.79 | HhH-GPD |
| OPB44892 | T4742_S00013.197 | HhH-GPD family base excision DNA repair protein |
| OPB44115 | T4742_S00003.386 | HhH-GPD superfamily base excision DNA repair protein |
| OPB42139 | T4742_S00004.241 | High affinity Ca2+/Mn2+ P-type ATPase (Golgi) |
| OPB39122 | T4742_S00006.182 | high affinity nickel permease |
| OPB38982 | T4742_S00006.41 | High mobility group box, HMG1/HMG2 |
| OPB45273 | T4742_S00010.184 | high osmolarity signaling protein Sho1, putative |
| OPB41693 | T4742_S00018.15 | high-affinity glucose transporter |
| OPB41159 | T4742_S00020.183 | hippurate hydrolase |
| OPB40541 | T4742_S00001.620 | HIR1 (histone transcription regulator ) |
| OPB44100 | T4742_S00003.371 | HisF Imidazoleglycerol-phosphate synthase |
| OPB41695 | T4742_S00018.17 | histidine acid phytase |
| OPB46261 | T4742_S00008.328 | Histidine kinase |
| OPB36558 | T4742_S00007.275 | histidine kinase  PHY1p, phytochrome-like |
| OPB38776 | T4742_S00002.825 | histidine kinase class I, M27Mp |
| OPB40288 | T4742_S00001.367 | histidine kinase class IV Fos-1 |
| OPB43195 | T4742_S00012.432 | histidine kinase class X, HHK1 |
| OPB41145 | T4742_S00020.169 | histidine kinase HHK3 |
| OPB37833 | T4742_S00017.100 | histidine kinase HHK6 class IX |
| OPB42999 | T4742_S00012.236 | histidine kinase Nik1 class III |
| OPB45869 | T4742_S00014.204 | Histidine kinase, class VI |
| OPB37987 | T4742_S00002.34 | Histidine kinase, part of a two component signal transduction system |
| OPB42473 | T4742_S00004.575 | Histidine kinase, part of a two-component phosphorelay system involved in signal transduction |

| | | |
|---|---|---|
| OPB44289 | T4742_S00003.560 | Histidine kinase, part of a two-component signal transduction system, related to N. crassa DCC1 |
| OPB44304 | T4742_S00003.575 | Histidinol dehydrogenase |
| OPB45358 | T4742_S00010.269 | histidinol phosphatase |
| OPB38185 | T4742_S00002.232 | Histidinol-phosphate/aromatic aminotransferase and cobyric acid decarboxylase |
| OPB38976 | T4742_S00006.35 | histidyl-tRNA synthetase, class IIa, from other fungi. |
| OPB45742 | T4742_S00014.77 | histone 2A |
| OPB43147 | T4742_S00012.384 | histone acetylase complex subunit |
| OPB37568 | T4742_S00005.595 | histone acetylase complex subunit Paf400, putative |
| OPB44921 | T4742_S00013.226 | histone acetyltransferase |
| OPB43310 | T4742_S00019.43 | Histone acetyltransferase (MYST family) |
| OPB39894 | T4742_S00015.238 | histone acetyltransferase ESA1 |
| OPB44118 | T4742_S00003.389 | Histone acetyltransferase SAGA/ADA, catalytic subunit PCAF/GCN5 and related proteins |
| OPB42328 | T4742_S00004.430 | histone acetyltransferase subunit (Yaf9) [Pochonia chlamydosporia 170] |
| OPB39365 | T4742_S00006.425 | histone acetyltransferase type B catalytic subunit |
| OPB37179 | T4742_S00005.206 | histone acetyltransferases (HATs) RTT109 |
| OPB36669 | T4742_S00007.387 | histone chaperone ASF1 |
| OPB46431 | T4742_S00008.498 | histone chaperones class I |
| OPB44266 | T4742_S00003.537 | histone chaperones class II |
| OPB41972 | T4742_S00004.74 | histone chapterones class II |
| OPB43212 | T4742_S00022.15 | histone deacetylase |
| OPB39626 | T4742_S00006.686 | histone deacetylase |
| OPB44203 | T4742_S00003.474 | histone deacetylase complex subunit SIN3 |
| OPB37768 | T4742_S00017.35 | Histone deacetylase; regulates transcription and silencing |
| OPB45359 | T4742_S00010.270 | histone deacetylases class I |
| OPB36770 | T4742_S00007.488 | Histone H1/H5 [Trichoderma guizhouense] |
| OPB43653 | T4742_S00016.173 | histone H1-binding protein, putative |
| OPB36953 | T4742_S00007.671 | histone H2A |
| OPB36954 | T4742_S00007.672 | histone H2B |
| OPB46576 | T4742_S00009.16 | Histone H3 |
| OPB45135 | T4742_S00010.42 | histone H3 lysine 36 (K36) methyltransferase |
| OPB40440 | T4742_S00001.519 | histone H3 methyltransferase |
| OPB46071 | T4742_S00008.138 | histone H3 methyltransferase complex and RNA cleavage factor II complex, subunit SWD2 |
| OPB42256 | T4742_S00004.358 | histone H4 |
| OPB41557 | T4742_S00011.400 | histone H4 variant |
| OPB38319 | T4742_S00002.367 | histone H4, putative |
| OPB42316 | T4742_S00004.418 | Histone Kinases |

| | | |
|---|---|---|
| OPB39386 | T4742_S00006.446 | histone phosphatases |
| OPB45527 | T4742_S00010.438 | histone tail methylase containing SET domain |
| OPB36742 | T4742_S00007.460 | Histone-fold protein [Trichoderma guizhouense] |
| OPB38473 | T4742_S00002.521 | histone-lysine N methyltransferase |
| OPB42997 | T4742_S00012.234 | HMG box protein |
| OPB39058 | T4742_S00006.117 | HMG box-containing protein |
| OPB44445 | T4742_S00003.717 | HMG family protein |
| OPB43143 | T4742_S00012.380 | HMGA subfamily |
| OPB42619 | T4742_S00004.723 | HMG-CoA reductase. |
| OPB39280 | T4742_S00006.340 | HMGL-like |
| OPB36005 | T4742_S00023.40 | HNWD1 |
| OPB42706 | T4742_S00004.812 | Homeobox |
| OPB40167 | T4742_S00001.246 | homeobox domain-containing protein |
| OPB41532 | T4742_S00011.375 | homeobox domain-containing protein |
| OPB37213 | T4742_S00005.240 | homeobox transcriptional regulator |
| OPB42978 | T4742_S00012.215 | homeobox transcriptional regulator |
| OPB46759 | T4742_S00009.201 | homeobox transcriptional regulator |
| OPB47158 | T4742_S00009.600 | Homeodomain-like |
| OPB45178 | T4742_S00010.85 | Homoaconitase |
| OPB41065 | T4742_S00020.89 | Homoaconitase catalytic domain |
| OPB45059 | T4742_S00013.364 | homocitrate synthase |
| OPB46440 | T4742_S00008.507 | homocitrate synthase |
| OPB45346 | T4742_S00010.257 | Homocysteine S-methyltransferase |
| OPB38540 | T4742_S00002.589 | homogentisate 1,2-dioxygenase |
| OPB36586 | T4742_S00007.303 | homogentisate dioxygenase HgmA |
| OPB45821 | T4742_S00014.156 | homoserine acetyltransferase [Aureobasidium melanogenum CBS 110374] |
| OPB41788 | T4742_S00018.111 | homoserine acetyltransferase family protein |
| OPB41953 | T4742_S00004.55 | Homoserine dehydrogenase |
| OPB44016 | T4742_S00003.287 | Homoserine kinase |
| OPB45412 | T4742_S00010.323 | homoserine o-acetyltransferase |
| OPB41360 | T4742_S00011.201 | homoserine O-acetyltransferase [Aschersonia aleyrodis RCEF 2490] |
| OPB37021 | T4742_S00005.48 | HpcH/HpaI aldolase/citrate lyase family protein |
| OPB42572 | T4742_S00004.675 | Hsc70 cochaperone (SGT), putative [Cordyceps militaris CM01] |
| OPB41524 | T4742_S00011.367 | HSP104 and related ATP-dependent Clp proteases |
| OPB39187 | T4742_S00006.247 | Hsp26/Hsp42 |
| OPB43709 | T4742_S00016.229 | Hsp26/Hsp42 |

| | | |
|---|---|---|
| OPB38270 | T4742_S00002.317 | Hsp27-ERE-TATA-binding protein/Scaffold attachment factor (SAF-B) [Trichoderma guizhouense] |
| OPB45522 | T4742_S00010.433 | Hsp30 |
| OPB41167 | T4742_S00011.7 | HSP30, putative |
| OPB44987 | T4742_S00013.292 | HSP40 |
| OPB38948 | T4742_S00006.7 | HSP60 cyt family |
| OPB47000 | T4742_S00009.442 | HSP60 cyt family |
| OPB38690 | T4742_S00002.739 | Hsp70 chaperone (BiP), putative |
| OPB36237 | T4742_S00021.155 | Hsp70 family chaperone |
| OPB43006 | T4742_S00012.243 | hsp70 family chaperone [Colletotrichum incanum] |
| OPB38427 | T4742_S00002.475 | Hsp70 family protein |
| OPB39188 | T4742_S00006.248 | Hsp70 family protein |
| OPB41335 | T4742_S00011.176 | Hsp70 family protein |
| OPB43172 | T4742_S00012.409 | Hsp70 family protein |
| OPB44374 | T4742_S00003.645 | hsp70 family protein |
| OPB40139 | T4742_S00001.218 | Hsp70 nucleotide exchange factor FES1 |
| OPB46264 | T4742_S00008.331 | hsp70 protein |
| OPB41986 | T4742_S00004.88 | Hsp90 co-chaperone AHA1 |
| OPB37323 | T4742_S00005.350 | Hus1, a component of the 9-1-1 replication checkpoint clamp |
| OPB36623 | T4742_S00007.341 | HVA22 domain membrane protein, pathogenicity related |
| OPB36081 | T4742_S00024.42 | hydantoinase/oxoprolinase |
| OPB40385 | T4742_S00001.464 | hydantoinase/oxoprolinase |
| OPB36730 | T4742_S00007.448 | Hydantoinase/oxoprolinase |
| OPB41058 | T4742_S00020.82 | Hydantoinase/oxoprolinase |
| OPB36189 | T4742_S00021.107 | hydrolase |
| OPB41812 | T4742_S00018.135 | hydrolase |
| OPB42828 | T4742_S00012.65 | hydrolase |
| OPB45258 | T4742_S00010.167 | hydrolase |
| OPB46068 | T4742_S00008.135 | hydrolase |
| OPB47105 | T4742_S00009.547 | hydrolase |
| OPB45652 | T4742_S00010.563 | Hydrolases of alpha/beta hydrolase superfamily |
| OPB37525 | T4742_S00005.552 | hydrophobin HFB4 |
| OPB38365 | T4742_S00002.413 | hydrophobin HFB16, with extended N and C terminus |
| OPB38530 | T4742_S00002.579 | Hydrophobin HFB8 |
| OPB38772 | T4742_S00002.821 | hydrophobin |
| OPB38878 | T4742_S00002.927 | Hydrophobin HFB6 |
| OPB42521 | T4742_S00004.623 | hydrophobin, new |

| | | |
|---|---|---|
| OPB44528 | T4742_S00003.800 | hydrophobin, pseudo-class I, extended N-terminus |
| OPB44529 | T4742_S00003.801 | hydrophobin, pseudo-class I |
| OPB44696 | T4742_S00003.968 | hydrophobin with extended N and C terminus, HFB10 |
| OPB40046 | T4742_S00001.125 | hydroxyacylglutathione hydrolase |
| OPB43309 | T4742_S00019.42 | hydroxybutyrate dehydrogenase |
| OPB39355 | T4742_S00006.415 | hydroxyethylthiazole kinase |
| OPB42348 | T4742_S00004.450 | hydroxyisocaproate dehydrogenase |
| OPB46526 | T4742_S00008.593 | hydroxylase [Streptomyces sp. NBS 14/10] |
| OPB36000 | T4742_S00023.35 | hydroxymethylglutaryl CoA synthase |
| OPB37716 | T4742_S00005.744 | Hydroxyneurosporene synthase [Metarhizium guizhouense ARSEF 977] |
| OPB40740 | T4742_S00001.821 | hydroxyneurosporene synthase [Umbilicaria pustulata] |
| OPB39104 | T4742_S00006.164 | Hydroxysteroid 17-beta dehydrogenase 11 |
| OPB42696 | T4742_S00004.802 | HypA |
| OPB43342 | T4742_S00019.75 | HypA-like protein [Trichoderma guizhouense] |
| OPB37963 | T4742_S00002.10 | hypothetical protein A0O28_0010670 [Trichoderma guizhouense] |
| OPB37964 | T4742_S00002.11 | hypothetical protein A0O28_0010680 [Trichoderma guizhouense] |
| OPB37978 | T4742_S00002.25 | hypothetical protein A0O28_0010820 [Trichoderma guizhouense] |
| OPB38087 | T4742_S00002.134 | hypothetical protein A0O28_0011910 [Trichoderma guizhouense] |
| OPB38111 | T4742_S00002.158 | hypothetical protein A0O28_0012150 [Trichoderma guizhouense] |
| OPB38114 | T4742_S00002.161 | hypothetical protein A0O28_0012180 [Trichoderma guizhouense] |
| OPB38162 | T4742_S00002.209 | hypothetical protein A0O28_0012660 [Trichoderma guizhouense] |
| OPB38204 | T4742_S00002.251 | hypothetical protein A0O28_0013080 [Trichoderma guizhouense] |
| OPB38220 | T4742_S00002.267 | hypothetical protein A0O28_0013240 [Trichoderma guizhouense] |
| OPB38224 | T4742_S00002.271 | hypothetical protein A0O28_0013280 [Trichoderma guizhouense] |
| OPB38238 | T4742_S00002.285 | hypothetical protein A0O28_0013420 [Trichoderma guizhouense] |
| OPB38255 | T4742_S00002.302 | hypothetical protein A0O28_0013590 [Trichoderma guizhouense] |
| OPB38322 | T4742_S00002.370 | hypothetical protein A0O28_0014270 [Trichoderma guizhouense] |
| OPB38325 | T4742_S00002.373 | hypothetical protein A0O28_0014300 [Trichoderma guizhouense] |
| OPB38357 | T4742_S00002.405 | hypothetical protein A0O28_0014620 [Trichoderma guizhouense] |
| OPB38372 | T4742_S00002.420 | hypothetical protein A0O28_0014770 [Trichoderma guizhouense] |
| OPB38407 | T4742_S00002.455 | hypothetical protein A0O28_0015120 [Trichoderma guizhouense] |
| OPB38415 | T4742_S00002.463 | hypothetical protein A0O28_0015200 [Trichoderma guizhouense] |
| OPB38448 | T4742_S00002.496 | hypothetical protein A0O28_0015530 [Trichoderma guizhouense] |
| OPB38462 | T4742_S00002.510 | hypothetical protein A0O28_0015670 [Trichoderma guizhouense] |
| OPB38521 | T4742_S00002.570 | hypothetical protein A0O28_0016270 [Trichoderma guizhouense] |
| OPB38522 | T4742_S00002.571 | hypothetical protein A0O28_0016280 [Trichoderma guizhouense] |

| | | |
|---|---|---|
| OPB38525 | T4742_S00002.574 | hypothetical protein A0O28_0016310 [Trichoderma guizhouense] |
| OPB38541 | T4742_S00002.590 | hypothetical protein A0O28_0016470 [Trichoderma guizhouense] |
| OPB38545 | T4742_S00002.594 | hypothetical protein A0O28_0016510 [Trichoderma guizhouense] |
| OPB38579 | T4742_S00002.628 | hypothetical protein A0O28_0016850 [Trichoderma guizhouense] |
| OPB38580 | T4742_S00002.629 | hypothetical protein A0O28_0016860 [Trichoderma guizhouense] |
| OPB38612 | T4742_S00002.661 | hypothetical protein A0O28_0017180 [Trichoderma guizhouense] |
| OPB38621 | T4742_S00002.670 | hypothetical protein A0O28_0017270 [Trichoderma guizhouense] |
| OPB38738 | T4742_S00002.787 | hypothetical protein A0O28_0018440 [Trichoderma guizhouense] |
| OPB38783 | T4742_S00002.832 | hypothetical protein A0O28_0018890 [Trichoderma guizhouense] |
| OPB38841 | T4742_S00002.890 | hypothetical protein A0O28_0019470 [Trichoderma guizhouense] |
| OPB38901 | T4742_S00002.950 | hypothetical protein A0O28_0020070 [Trichoderma guizhouense] |
| OPB38908 | T4742_S00002.957 | hypothetical protein A0O28_0020140 [Trichoderma guizhouense] |
| OPB38912 | T4742_S00002.961 | hypothetical protein A0O28_0020180 [Trichoderma guizhouense] |
| OPB38916 | T4742_S00002.965 | hypothetical protein A0O28_0020220 [Trichoderma guizhouense] |
| OPB38938 | T4742_S00002.987 | hypothetical protein A0O28_0020440 [Trichoderma guizhouense] |
| OPB37023 | T4742_S00005.50 | hypothetical protein A0O28_0039350 [Trichoderma guizhouense] |
| OPB37047 | T4742_S00005.74 | hypothetical protein A0O28_0039590 [Trichoderma guizhouense] |
| OPB37057 | T4742_S00005.84 | hypothetical protein A0O28_0039690 [Trichoderma guizhouense] |
| OPB37084 | T4742_S00005.111 | hypothetical protein A0O28_0039960 [Trichoderma guizhouense] |
| OPB37090 | T4742_S00005.117 | hypothetical protein A0O28_0040020 [Trichoderma guizhouense] |
| OPB37147 | T4742_S00005.174 | hypothetical protein A0O28_0040590 [Trichoderma guizhouense] |
| OPB37162 | T4742_S00005.189 | hypothetical protein A0O28_0040740 [Trichoderma guizhouense] |
| OPB37176 | T4742_S00005.203 | hypothetical protein A0O28_0040880 [Trichoderma guizhouense] |
| OPB37189 | T4742_S00005.216 | hypothetical protein A0O28_0041010 [Trichoderma guizhouense] |
| OPB37190 | T4742_S00005.217 | hypothetical protein A0O28_0041020 [Trichoderma guizhouense] |
| OPB37194 | T4742_S00005.221 | hypothetical protein A0O28_0041060 [Trichoderma guizhouense] |
| OPB37221 | T4742_S00005.248 | hypothetical protein A0O28_0041330 [Trichoderma guizhouense] |
| OPB37231 | T4742_S00005.258 | hypothetical protein A0O28_0041430 [Trichoderma guizhouense] |
| OPB37276 | T4742_S00005.303 | hypothetical protein A0O28_0041880 [Trichoderma guizhouense] |
| OPB37284 | T4742_S00005.311 | hypothetical protein A0O28_0041960 [Trichoderma guizhouense] |
| OPB37294 | T4742_S00005.321 | hypothetical protein A0O28_0042060 [Trichoderma guizhouense] |
| OPB37320 | T4742_S00005.347 | hypothetical protein A0O28_0042320 [Trichoderma guizhouense] |
| OPB37352 | T4742_S00005.379 | hypothetical protein A0O28_0042640 [Trichoderma guizhouense] |
| OPB37377 | T4742_S00005.404 | hypothetical protein A0O28_0042890 [Trichoderma guizhouense] |
| OPB37378 | T4742_S00005.405 | hypothetical protein A0O28_0042900 [Trichoderma guizhouense] |
| OPB37413 | T4742_S00005.440 | hypothetical protein A0O28_0043250 [Trichoderma guizhouense] |

| | | |
|---|---|---|
| OPB37489 | T4742_S00005.516 | hypothetical protein A0O28_0044010 [Trichoderma guizhouense] |
| OPB37494 | T4742_S00005.521 | hypothetical protein A0O28_0044060 [Trichoderma guizhouense] |
| OPB37512 | T4742_S00005.539 | hypothetical protein A0O28_0044240 [Trichoderma guizhouense] |
| OPB37569 | T4742_S00005.596 | hypothetical protein A0O28_0044810 [Trichoderma guizhouense] |
| OPB37581 | T4742_S00005.608 | hypothetical protein A0O28_0044930 [Trichoderma guizhouense] |
| OPB37609 | T4742_S00005.636 | hypothetical protein A0O28_0045210 [Trichoderma guizhouense] |
| OPB37618 | T4742_S00005.645 | hypothetical protein A0O28_0045300 [Trichoderma guizhouense] |
| OPB37622 | T4742_S00005.649 | hypothetical protein A0O28_0045340 [Trichoderma guizhouense] |
| OPB37638 | T4742_S00005.665 | hypothetical protein A0O28_0045500 [Trichoderma guizhouense] |
| OPB37645 | T4742_S00005.672 | hypothetical protein A0O28_0045570 [Trichoderma guizhouense] |
| OPB37671 | T4742_S00005.698 | hypothetical protein A0O28_0045830 [Trichoderma guizhouense] |
| OPB37690 | T4742_S00005.717 | hypothetical protein A0O28_0046020 [Trichoderma guizhouense] |
| OPB37713 | T4742_S00005.741 | hypothetical protein A0O28_0046260 [Trichoderma guizhouense] |
| OPB37724 | T4742_S00005.752 | hypothetical protein A0O28_0046370 [Trichoderma guizhouense] |
| OPB37728 | T4742_S00005.756 | hypothetical protein A0O28_0046410 [Trichoderma guizhouense] |
| OPB38968 | T4742_S00006.27 | hypothetical protein A0O28_0046730 [Trichoderma guizhouense] |
| OPB38974 | T4742_S00006.33 | hypothetical protein A0O28_0046790 [Trichoderma guizhouense] |
| OPB38987 | T4742_S00006.46 | hypothetical protein A0O28_0046920 [Trichoderma guizhouense] |
| OPB38999 | T4742_S00006.58 | hypothetical protein A0O28_0047040 [Trichoderma guizhouense] |
| OPB39010 | T4742_S00006.69 | hypothetical protein A0O28_0047150 [Trichoderma guizhouense] |
| OPB39027 | T4742_S00006.86 | hypothetical protein A0O28_0047320 [Trichoderma guizhouense] |
| OPB39037 | T4742_S00006.96 | hypothetical protein A0O28_0047420 [Trichoderma guizhouense] |
| OPB39040 | T4742_S00006.99 | hypothetical protein A0O28_0047450 [Trichoderma guizhouense] |
| OPB39082 | T4742_S00006.141 | hypothetical protein A0O28_0047870 [Trichoderma guizhouense] |
| OPB39093 | T4742_S00006.152 | hypothetical protein A0O28_0047980 [Trichoderma guizhouense] |
| OPB39099 | T4742_S00006.158 | hypothetical protein A0O28_0048040 [Trichoderma guizhouense] |
| OPB39101 | T4742_S00006.161 | hypothetical protein A0O28_0048070 [Trichoderma guizhouense] |
| OPB39114 | T4742_S00006.174 | hypothetical protein A0O28_0048200 [Trichoderma guizhouense] |
| OPB39144 | T4742_S00006.204 | hypothetical protein A0O28_0048500 [Trichoderma guizhouense] |
| OPB39149 | T4742_S00006.209 | hypothetical protein A0O28_0048550 [Trichoderma guizhouense] |
| OPB39156 | T4742_S00006.216 | hypothetical protein A0O28_0048620 [Trichoderma guizhouense] |
| OPB39195 | T4742_S00006.255 | hypothetical protein A0O28_0049010 [Trichoderma guizhouense] |
| OPB39197 | T4742_S00006.257 | hypothetical protein A0O28_0049030 [Trichoderma guizhouense] |
| OPB39211 | T4742_S00006.271 | hypothetical protein A0O28_0049170 [Trichoderma guizhouense] |
| OPB39213 | T4742_S00006.273 | hypothetical protein A0O28_0049190 [Trichoderma guizhouense] |
| OPB39239 | T4742_S00006.299 | hypothetical protein A0O28_0049450 [Trichoderma guizhouense] |

| | | |
|---|---|---|
| OPB39257 | T4742_S00006.317 | hypothetical protein A0O28_0049630 [Trichoderma guizhouense] |
| OPB39299 | T4742_S00006.359 | hypothetical protein A0O28_0050050 [Trichoderma guizhouense] |
| OPB39300 | T4742_S00006.360 | hypothetical protein A0O28_0050060 [Trichoderma guizhouense] |
| OPB39323 | T4742_S00006.383 | hypothetical protein A0O28_0050290 [Trichoderma guizhouense] |
| OPB39342 | T4742_S00006.402 | hypothetical protein A0O28_0050480 [Trichoderma guizhouense] |
| OPB39343 | T4742_S00006.403 | hypothetical protein A0O28_0050490 [Trichoderma guizhouense] |
| OPB39360 | T4742_S00006.420 | hypothetical protein A0O28_0050660 [Trichoderma guizhouense] |
| OPB39375 | T4742_S00006.435 | hypothetical protein A0O28_0050810 [Trichoderma guizhouense] |
| OPB39385 | T4742_S00006.445 | hypothetical protein A0O28_0050910 [Trichoderma guizhouense] |
| OPB39387 | T4742_S00006.447 | hypothetical protein A0O28_0050930 [Trichoderma guizhouense] |
| OPB39399 | T4742_S00006.459 | hypothetical protein A0O28_0051050 [Trichoderma guizhouense] |
| OPB39464 | T4742_S00006.524 | hypothetical protein A0O28_0051700 [Trichoderma guizhouense] |
| OPB39514 | T4742_S00006.574 | hypothetical protein A0O28_0052200 [Trichoderma guizhouense] |
| OPB39515 | T4742_S00006.575 | hypothetical protein A0O28_0052210 [Trichoderma guizhouense] |
| OPB39518 | T4742_S00006.578 | hypothetical protein A0O28_0052240 [Trichoderma guizhouense] |
| OPB39524 | T4742_S00006.584 | hypothetical protein A0O28_0052300 [Trichoderma guizhouense] |
| OPB39558 | T4742_S00006.618 | hypothetical protein A0O28_0052640 [Trichoderma guizhouense] |
| OPB39560 | T4742_S00006.620 | hypothetical protein A0O28_0052660 [Trichoderma guizhouense] |
| OPB39594 | T4742_S00006.654 | hypothetical protein A0O28_0053000 [Trichoderma guizhouense] |
| OPB36362 | T4742_S00007.79 | hypothetical protein A0O28_0054410 [Trichoderma guizhouense] |
| OPB36366 | T4742_S00007.83 | hypothetical protein A0O28_0054450 [Trichoderma guizhouense] |
| OPB36370 | T4742_S00007.87 | hypothetical protein A0O28_0054490 [Trichoderma guizhouense] |
| OPB36397 | T4742_S00007.114 | hypothetical protein A0O28_0054760 [Trichoderma guizhouense] |
| OPB36408 | T4742_S00007.125 | hypothetical protein A0O28_0054870 [Trichoderma guizhouense] |
| OPB36409 | T4742_S00007.126 | hypothetical protein A0O28_0054880 [Trichoderma guizhouense] |
| OPB36434 | T4742_S00007.151 | hypothetical protein A0O28_0055130 [Trichoderma guizhouense] |
| OPB36437 | T4742_S00007.154 | hypothetical protein A0O28_0055160 [Trichoderma guizhouense] |
| OPB36482 | T4742_S00007.199 | hypothetical protein A0O28_0055610 [Trichoderma guizhouense] |
| OPB36493 | T4742_S00007.210 | hypothetical protein A0O28_0055720 [Trichoderma guizhouense] |
| OPB36500 | T4742_S00007.217 | hypothetical protein A0O28_0055790 [Trichoderma guizhouense] |
| OPB36510 | T4742_S00007.227 | hypothetical protein A0O28_0055890 [Trichoderma guizhouense] |
| OPB36521 | T4742_S00007.238 | hypothetical protein A0O28_0056000 [Trichoderma guizhouense] |
| OPB36524 | T4742_S00007.241 | hypothetical protein A0O28_0056030 [Trichoderma guizhouense] |
| OPB36525 | T4742_S00007.242 | hypothetical protein A0O28_0056040 [Trichoderma guizhouense] |
| OPB36527 | T4742_S00007.244 | hypothetical protein A0O28_0056060 [Trichoderma guizhouense] |
| OPB36562 | T4742_S00007.279 | hypothetical protein A0O28_0056410 [Trichoderma guizhouense] |

| | | |
|---|---|---|
| OPB36564 | T4742_S00007.281 | hypothetical protein A0O28_0056430 [Trichoderma guizhouense] |
| OPB36569 | T4742_S00007.286 | hypothetical protein A0O28_0056480 [Trichoderma guizhouense] |
| OPB36610 | T4742_S00007.328 | hypothetical protein A0O28_0056900 [Trichoderma guizhouense] |
| OPB36641 | T4742_S00007.359 | hypothetical protein A0O28_0057210 [Trichoderma guizhouense] |
| OPB36645 | T4742_S00007.363 | hypothetical protein A0O28_0057250 [Trichoderma guizhouense] |
| OPB36662 | T4742_S00007.380 | hypothetical protein A0O28_0057420 [Trichoderma guizhouense] |
| OPB36715 | T4742_S00007.433 | hypothetical protein A0O28_0057950 [Trichoderma guizhouense] |
| OPB36716 | T4742_S00007.434 | hypothetical protein A0O28_0057960 [Trichoderma guizhouense] |
| OPB36745 | T4742_S00007.463 | hypothetical protein A0O28_0058250 [Trichoderma guizhouense] |
| OPB36763 | T4742_S00007.481 | hypothetical protein A0O28_0058430 [Trichoderma guizhouense] |
| OPB36765 | T4742_S00007.483 | hypothetical protein A0O28_0058450 [Trichoderma guizhouense] |
| OPB36807 | T4742_S00007.525 | hypothetical protein A0O28_0058870 [Trichoderma guizhouense] |
| OPB36823 | T4742_S00007.541 | hypothetical protein A0O28_0059030 [Trichoderma guizhouense] |
| OPB36835 | T4742_S00007.553 | hypothetical protein A0O28_0059150 [Trichoderma guizhouense] |
| OPB36848 | T4742_S00007.566 | hypothetical protein A0O28_0059280 [Trichoderma guizhouense] |
| OPB36868 | T4742_S00007.586 | hypothetical protein A0O28_0059480 [Trichoderma guizhouense] |
| OPB36955 | T4742_S00007.673 | hypothetical protein A0O28_0060350 [Trichoderma guizhouense] |
| OPB39671 | T4742_S00015.15 | hypothetical protein A0O28_0095180 [Trichoderma guizhouense] |
| OPB39703 | T4742_S00015.47 | hypothetical protein A0O28_0095500 [Trichoderma guizhouense] |
| OPB37751 | T4742_S00017.18 | hypothetical protein A0O28_0100350 [Trichoderma guizhouense] |
| OPB37754 | T4742_S00017.21 | hypothetical protein A0O28_0100380 [Trichoderma guizhouense] |
| OPB37764 | T4742_S00017.31 | hypothetical protein A0O28_0100480 [Trichoderma guizhouense] |
| OPB37819 | T4742_S00017.86 | hypothetical protein A0O28_0101030 [Trichoderma guizhouense] |
| OPB37852 | T4742_S00017.119 | hypothetical protein A0O28_0101360 [Trichoderma guizhouense] |
| OPB37873 | T4742_S00017.140 | hypothetical protein A0O28_0101570 [Trichoderma guizhouense] |
| OPB37883 | T4742_S00017.150 | hypothetical protein A0O28_0101670 [Trichoderma guizhouense] |
| OPB37905 | T4742_S00017.172 | hypothetical protein A0O28_0101890 [Trichoderma guizhouense] |
| OPB37907 | T4742_S00017.174 | hypothetical protein A0O28_0101910 [Trichoderma guizhouense] |
| OPB37937 | T4742_S00017.204 | hypothetical protein A0O28_0102210 [Trichoderma guizhouense] |
| OPB37939 | T4742_S00017.206 | hypothetical protein A0O28_0102230 [Trichoderma guizhouense] |
| OPB36105 | T4742_S00021.21 | hypothetical protein A0O28_0108790 [Trichoderma guizhouense] |
| OPB36118 | T4742_S00021.35 | hypothetical protein A0O28_0108930 [Trichoderma guizhouense] |
| OPB36145 | T4742_S00021.62 | hypothetical protein A0O28_0109200 [Trichoderma guizhouense] |
| OPB36171 | T4742_S00021.88 | hypothetical protein A0O28_0109460 [Trichoderma guizhouense] |
| OPB36193 | T4742_S00021.111 | hypothetical protein A0O28_0109690 [Trichoderma guizhouense] |
| OPB36226 | T4742_S00021.144 | hypothetical protein A0O28_0110020 [Trichoderma guizhouense] |

| | | |
|---|---|---|
| OPB36268 | T4742_S00021.187 | hypothetical protein A0O28_0110450 [Trichoderma guizhouense] |
| OPB36269 | T4742_S00021.188 | hypothetical protein A0O28_0110460 [Trichoderma guizhouense] |
| OPB36044 | T4742_S00024.5 | hypothetical protein A0O28_0112100 [Trichoderma guizhouense] |
| OPB36052 | T4742_S00024.13 | hypothetical protein A0O28_0112180 [Trichoderma guizhouense] |
| OPB36057 | T4742_S00024.18 | hypothetical protein A0O28_0112230 [Trichoderma guizhouense] |
| OPB36222 | T4742_S00021.140 | hypothetical protein PENVUL_c024G00282 [Penicillium vulpinum] |
| OPB37838 | T4742_S00017.105 | hypothetical protein THAR02_09258 [Trichoderma harzianum] |
| OPB39469 | T4742_S00006.529 | hypothetical protein THAR02_10432 [Trichoderma harzianum] |
| OPB37460 | T4742_S00005.487 | HyuA, N-methylhydantoinase A/acetone carboxylase, beta subunit |
| OPB43925 | T4742_S00003.196 | IEC3 subunit of the ino80 complex, chromatin re-modelling domain-containing protein [Pochonia chlamydosporia 170] |
| OPB44901 | T4742_S00013.206 | ILVD_EDD Dehydratase family |
| OPB40892 | T4742_S00001.973 | ILVD_EDDDehydratase family |
| OPB39129 | T4742_S00006.189 | imidazole proprionase-related amidohydrolase |
| OPB37553 | T4742_S00005.580 | Imidazoleglycerol-phosphate dehydratase |
| OPB39103 | T4742_S00006.163 | IMP dehydrogenase |
| OPB47091 | T4742_S00009.533 | IMP dehydrogenase |
| OPB37087 | T4742_S00005.114 | IMP4, an SSU processome componentInvolvedIn pre 18s rRNA processing. |
| OPB38359 | T4742_S00002.407 | import inner membrane translocase subunit tim-21, mitochondrial , putative |
| OPB45220 | T4742_S00010.129 | importin ß KapB |
| OPB45021 | T4742_S00013.326 | importin ß KapC |
| OPB38651 | T4742_S00002.700 | Importin ß KapD |
| OPB40068 | T4742_S00001.147 | importin ß KapE |
| OPB43325 | T4742_S00019.58 | importin ß KapG |
| OPB44455 | T4742_S00003.727 | importin ß KapH |
| OPB36928 | T4742_S00007.646 | importin ß KapI |
| OPB40506 | T4742_S00001.585 | importin ß KapJ |
| OPB43136 | T4742_S00012.373 | importin ß KapK |
| OPB37736 | T4742_S00017.3 | importin ß KapL |
| OPB43064 | T4742_S00012.301 | importin ß KapM |
| OPB45940 | T4742_S00008.6 | importin ß KapN |
| OPB37623 | T4742_S00005.650 | importin α KapA |
| OPB38652 | T4742_S00002.701 | IMP-specific 5'-nucleotidase |
| OPB46705 | T4742_S00009.147 | increased rDNA silencing protein 4 |
| OPB43544 | T4742_S00016.64 | IndA1 amino acid transporter |
| OPB40273 | T4742_S00001.352 | indoleamine 2,3-dioxygenase |
| OPB41682 | T4742_S00018.4 | indoleamine 2,3-dioxygenase [Trichoderma guizhouense] |

| | | |
|---|---|---|
| OPB46520 | T4742_S00008.587 | indoleamine 2,3-dioxygenase family protein |
| OPB44126 | T4742_S00003.397 | Indoleamine 2,3-dioxygenase-like protein |
| OPB40610 | T4742_S00001.690 | initiation factor 2B |
| OPB43988 | T4742_S00003.259 | initiation factor 2B. |
| OPB45448 | T4742_S00010.359 | initiation factor 2B. |
| OPB39411 | T4742_S00006.471 | Initiation factor eIF-4 gamma, middle |
| OPB37348 | T4742_S00005.375 | initiator tRNA phosphoribosyl transferase. |
| OPB38412 | T4742_S00002.460 | inner centromere protein |
| OPB38482 | T4742_S00002.530 | inner membrane magnesium transporter MRS2 |
| OPB40122 | T4742_S00001.201 | INO80 chromatin remodeling complex Ies1 |
| OPB36792 | T4742_S00007.510 | Inorganic phosphate transporter |
| OPB38616 | T4742_S00002.665 | Inorganic phosphate transporter |
| OPB42149 | T4742_S00004.251 | Inorganic pyrophosphatase |
| OPB36043 | T4742_S00024.4 | inorganic pyrophosphatase |
| OPB46823 | T4742_S00009.265 | Inosine monophosphate cyclohydrolase |
| OPB42176 | T4742_S00004.278 | inosine-uridine preferring nucleoside hydrolase |
| OPB46800 | T4742_S00009.242 | inosine-uridine preferring nucleoside hydrolase |
| OPB47005 | T4742_S00009.447 | inosine-uridine preferring nucleoside hydrolase, variant [Neurospora crassa OR74A] |
| OPB37994 | T4742_S00002.41 | inositol monophosphatase |
| OPB45790 | T4742_S00014.125 | inositol monophosphatase family protein |
| OPB39060 | T4742_S00006.119 | inositol phospholipid synthesis protein Scs3p, putative |
| OPB45715 | T4742_S00014.49 | Inositol polyphosphate kinase, putative |
| OPB42330 | T4742_S00004.432 | inositol-pentakisphosphate 2-kinase |
| OPB43950 | T4742_S00003.221 | Integral ER membrane protein that regulates phospholipid metabolism |
| OPB36082 | T4742_S00024.43 | integral membrane protein |
| OPB36154 | T4742_S00021.71 | integral membrane protein |
| OPB36764 | T4742_S00007.482 | integral membrane protein |
| OPB38964 | T4742_S00006.23 | integral membrane protein |
| OPB42364 | T4742_S00004.466 | integral membrane protein |
| OPB46130 | T4742_S00008.197 | integral membrane protein |
| OPB46287 | T4742_S00008.354 | integral membrane protein |
| OPB46499 | T4742_S00008.566 | integral membrane protein |
| OPB39480 | T4742_S00006.540 | integral membrane protein |
| OPB45964 | T4742_S00008.30 | integral membrane protein (Ptm1), putative |
| OPB42402 | T4742_S00004.504 | integral membrane protein [Aschersonia aleyrodis RCEF 2490] |
| OPB40966 | T4742_S00001.1047 | integral membrane protein [Ophiostoma piceae UAMH 11346] |

| | | |
|---|---|---|
| OPB37386 | T4742_S00005.413 | integral membrane protein comprising an SPX and an EXS domain, therefore most probably invoved in G-protein associated signalling |
| OPB36772 | T4742_S00007.490 | Integral membrane protein Sys1 |
| OPB41468 | T4742_S00011.311 | integral membrane protein, Mpv17/PMP22 family, putative |
| OPB37003 | T4742_S00005.30 | integral membrane protein, putative |
| OPB46572 | T4742_S00009.12 | Integral membrane protein, putative |
| OPB41637 | T4742_S00011.480 | integral membrane protein,Mpv17/PMP22 |
| OPB40576 | T4742_S00001.656 | integral membrane protein/MFS transporter |
| OPB44122 | T4742_S00003.393 | integral peroxisomal membrane peroxin, putative |
| OPB41626 | T4742_S00011.469 | intermembrane peptidase |
| OPB40345 | T4742_S00001.424 | intermembrane space import and assembly protein 40 [Metarhizium acridum CQMa 102] |
| OPB40463 | T4742_S00001.542 | intracellular serine protease |
| OPB41066 | T4742_S00020.90 | intracellular serine protease |
| OPB38563 | T4742_S00002.612 | Intradiol ring-cleavage dioxygenase, C-terminal |
| OPB43330 | T4742_S00019.63 | Ion transport protein |
| OPB44206 | T4742_S00003.477 | IQ calmodulin-binding domain-containing protein [Purpureocillium lilacinum] |
| OPB44207 | T4742_S00003.478 | IQ-calmodulin binding motif-containing protein |
| OPB41258 | T4742_S00011.99 | iron transporter |
| OPB41530 | T4742_S00011.373 | iron transporter |
| OPB42381 | T4742_S00004.483 | iron transporter |
| OPB42867 | T4742_S00012.104 | iron transporter |
| OPB44221 | T4742_S00003.492 | iron transporter |
| OPB38767 | T4742_S00002.816 | Iron/ascorbate family oxidoreductases |
| OPB39305 | T4742_S00006.365 | Iron/ascorbate family oxidoreductases |
| OPB40630 | T4742_S00001.710 | Iron/ascorbate family oxidoreductases |
| OPB40988 | T4742_S00020.12 | Iron/ascorbate family oxidoreductases |
| OPB36021 | T4742_S00023.56 | Iron-containing alcohol dehydrogenase |
| OPB43398 | T4742_S00019.131 | iron-dependent alcohol dehydrogenase, class IV |
| OPB46024 | T4742_S00008.90 | iron-dependent alcohol dehydrogenase, class IV |
| OPB45110 | T4742_S00010.17 | iron-dependent peroxidase |
| OPB43557 | T4742_S00016.77 | iron-sulfur cluster assembly accessory protein Isa1, putative |
| OPB46693 | T4742_S00009.135 | iron-sulfur cluster assembly accessory protein, putative |
| OPB40493 | T4742_S00001.572 | isoamyl alcohol oxidase |
| OPB43351 | T4742_S00019.84 | isoamyl alcohol oxidase |
| OPB44506 | T4742_S00003.778 | isoamyl alcohol oxidase |
| OPB46145 | T4742_S00008.212 | isoamyl alcohol oxidase |
| OPB45644 | T4742_S00010.555 | isoamyl alcohol oxidase [Trichoderma guizhouense] |

| | | |
|---|---|---|
| OPB39359 | T4742_S00006.419 | Isochorismatase hydrolase |
| OPB41030 | T4742_S00020.54 | Isochorismatase hydrolase |
| OPB42618 | T4742_S00004.722 | Isochorismatase hydrolase |
| OPB43785 | T4742_S00003.56 | Isochorismatase hydrolase |
| OPB44554 | T4742_S00003.826 | Isochorismatase hydrolase |
| OPB40841 | T4742_S00001.922 | Isochorismatase hydrolase [Trichoderma reesei RUT C-30] |
| OPB47048 | T4742_S00009.490 | isocitrate dehydrogenase subunit IDH1, NAD-specific |
| OPB38094 | T4742_S00002.141 | Isocitrate dehydrogenase, subunit 2, NAD-dependent, mitochondrial |
| OPB39512 | T4742_S00006.572 | isocitrate lyase |
| OPB47155 | T4742_S00009.597 | Isocitrate/isopropylmalate dehydrogenase |
| OPB44609 | T4742_S00003.881 | isoflavon reductase [Trichoderma guizhouense] |
| OPB41890 | T4742_S00018.213 | isoflavone reductase |
| OPB40574 | T4742_S00001.654 | Isoflavone reductase [Colletotrichum chlorophyti] |
| OPB45621 | T4742_S00010.532 | Isoflavone reductase [Colletotrichum chlorophyti] |
| OPB39077 | T4742_S00006.136 | Isoflavone reductase [Trichoderma guizhouense] |
| OPB40381 | T4742_S00001.460 | isoflavone reductase [Trichoderma guizhouense] |
| OPB40536 | T4742_S00001.615 | isoflavone reductase family protein [Trichoderma gamsii] |
| OPB40562 | T4742_S00001.641 | isoflavone reductase family protein [Trichoderma gamsii] |
| OPB43428 | T4742_S00019.161 | isoflavone reductase family protein [Trichoderma guizhouense] |
| OPB43508 | T4742_S00016.28 | Isoleucine tRNA synthase. |
| OPB44404 | T4742_S00003.675 | isoleucyl-tRNA synthetase, class Ia. |
| OPB44475 | T4742_S00003.747 | Isopenicillin N synthase |
| OPB38350 | T4742_S00002.398 | Isopentenyl diphosphate:dimethylallyl diphosphate isomerase |
| OPB47018 | T4742_S00009.460 | Isopentenyl-diphosphate delta isomerase 2 |
| OPB43084 | T4742_S00012.321 | isoprenylcysteine carboxyl methyltransferase |
| OPB36169 | T4742_S00021.86 | isopropanol dehydrogenase |
| OPB41696 | T4742_S00018.18 | Isopropylmalate/homocitrate/citramalate synthases |
| OPB35938 | T4742_S00025.11 | Isovaleryl-CoA dehydrogenase |
| OPB36919 | T4742_S00007.637 | Jacalin-like lectin |
| OPB36794 | T4742_S00007.512 | K(+)/H(+) antiporter 1 |
| OPB40930 | T4742_S00001.1011 | K+ channel |
| OPB37445 | T4742_S00005.472 | K+ channel protein |
| OPB45825 | T4742_S00014.160 | K+ dependent Na+/Ca++ exchanger, putative |
| OPB39333 | T4742_S00006.393 | K+ transporter Trk1 |
| OPB39353 | T4742_S00006.413 | keto--aminopelargonate synthetase BioF |
| OPB46390 | T4742_S00008.457 | keto--aminopelargonate synthetase BioF |

| | | |
|---|---|---|
| OPB44141 | T4742_S00003.412 | ketol-acid reductoisomerase, mitochondrial precursor |
| OPB46689 | T4742_S00009.131 | Ketopantoate reductase |
| OPB37161 | T4742_S00005.188 | Ketosamine-3-kinase |
| OPB40250 | T4742_S00001.329 | Kex1p involved in alpha factor processing |
| OPB44142 | T4742_S00003.413 | Kex2 protease to S. cerevisiae Kex2 protease |
| OPB40803 | T4742_S00001.884 | Killer toxin, Kp4/SMK-like, core, partial [Metarhizium brunneum ARSEF 3297] |
| OPB42622 | T4742_S00004.727 | kinesin |
| OPB38394 | T4742_S00002.442 | kinesin heavy chain |
| OPB46100 | T4742_S00008.167 | kinesin light chain [Colletotrichum incanum] |
| OPB44903 | T4742_S00013.208 | kinesin-like motor protein Kar3p required for karyogamy |
| OPB36875 | T4742_S00007.593 | kinesin-like protein |
| OPB42683 | T4742_S00004.789 | kinesin-like protein |
| OPB44426 | T4742_S00003.698 | kinesin-like protein |
| OPB45843 | T4742_S00014.178 | kinesin-like protein |
| OPB46834 | T4742_S00009.276 | kinesin-like protein |
| OPB37477 | T4742_S00005.504 | kinesin-like protein, a kinesin-3 family member. |
| OPB40042 | T4742_S00001.121 | kinesin-like protein, a kinesin-3 family member. |
| OPB41821 | T4742_S00018.144 | Kinetochore protein fta4 [Fusarium oxysporum f. sp. cubense race 4] |
| OPB43299 | T4742_S00019.32 | kinetochore protein NUF2 |
| OPB38025 | T4742_S00002.72 | kinetochore protein spc24, putative |
| OPB39721 | T4742_S00015.65 | kinetochore protein SPC25 |
| OPB39722 | T4742_S00015.66 | kinetochore protein SPC25 |
| OPB46314 | T4742_S00008.381 | KP4 killer toxin |
| OPB38029 | T4742_S00002.76 | ku70 |
| OPB39422 | T4742_S00006.482 | Ku80p (Hdf2p) |
| OPB40272 | T4742_S00001.351 | kynureninase [Fusarium fujikuroi] |
| OPB39699 | T4742_S00015.43 | Kynurenine aminotransferase, glutamine transaminase K |
| OPB43476 | T4742_S00019.210 | Kynurenine aminotransferase, glutamine transaminase K |
| OPB46302 | T4742_S00008.369 | Kynurenine aminotransferase, glutamine transaminase K |
| OPB39568 | T4742_S00006.628 | La domain family [Trichoderma guizhouense] |
| OPB42162 | T4742_S00004.264 | lactate 2-monooxygenase [Escovopsis weberi] |
| OPB42830 | T4742_S00012.67 | lactate dehydrogenase |
| OPB46625 | T4742_S00009.67 | lactate/malate dehydrogenase |
| OPB40707 | T4742_S00001.787 | lactonase-superfamily protein |
| OPB44277 | T4742_S00003.548 | LAE1 protein arginine methyltransferase |
| OPB41791 | T4742_S00018.114 | LamB/YcsF family protein |

| | | |
|---|---|---|
| OPB46414 | T4742_S00008.481 | LamB/YcsF family protein |
| OPB43392 | T4742_S00019.125 | L-amino acid oxidase |
| OPB45577 | T4742_S00010.488 | L-amino acid oxidase |
| OPB44279 | T4742_S00003.550 | L-arabinitol 4-dehydrogenase |
| OPB37729 | T4742_S00005.757 | large subunit of N2CN-dimethylformamidase |
| OPB38192 | T4742_S00002.239 | large-conductance mechanosensitive channel |
| OPB42504 | T4742_S00004.606 | lariat debranching enzyme domain-containing protein |
| OPB40705 | T4742_S00001.785 | L-asparaginase |
| OPB44790 | T4742_S00013.95 | L-asparaginase II |
| OPB44307 | T4742_S00003.578 | Late Golgi protein sorting complex, subunit Vps53 |
| OPB39567 | T4742_S00006.627 | Late Golgi transport protein Sft2 |
| OPB45841 | T4742_S00014.176 | L-carnitine dehydratase |
| OPB44592 | T4742_S00003.864 | L-carnitine dehydratase/alpha-methylacyl-CoA racemase |
| OPB37811 | T4742_S00017.78 | LdhA Lactate dehydrogenase and related dehydrogenases |
| OPB41206 | T4742_S00011.46 | LdhA Lactate dehydrogenase and related dehydrogenases |
| OPB47112 | T4742_S00009.554 | LdhA Lactate dehydrogenase and related dehydrogenases |
| OPB39369 | T4742_S00006.429 | lectin family integral membrane protein, putative |
| OPB46676 | T4742_S00009.118 | legume-like lectin |
| OPB40843 | T4742_S00001.924 | Leucine aminopeptidase 1 |
| OPB46994 | T4742_S00009.436 | Leucine aminopeptidase 1 |
| OPB38396 | T4742_S00002.444 | leucine carboxyl methyltransferase, putative |
| OPB44837 | T4742_S00013.142 | Leucine-rich repeat |
| OPB45331 | T4742_S00010.242 | Leucine-rich repeat |
| OPB36049 | T4742_S00024.10 | leucoanthocyanidin dioxygenase |
| OPB43180 | T4742_S00012.417 | leucyl-tRNA synthetase, class Ia |
| OPB36799 | T4742_S00007.517 | L-galactose dehydrogenase (L-GalDH), putative |
| OPB42439 | T4742_S00004.541 | Lif1p, involved in DNA double strand break repair; physically interacts with DNA ligase 4. |
| OPB45916 | T4742_S00014.252 | Linalool dehydratase-isomerase [Colletotrichum higginsianum IMI 349063] |
| OPB45010 | T4742_S00013.315 | LipA and NB-ARC domain protein [Metarhizium anisopliae] |
| OPB37243 | T4742_S00005.270 | lipase |
| OPB38595 | T4742_S00002.644 | lipase |
| OPB39393 | T4742_S00006.453 | lipase |
| OPB44878 | T4742_S00013.183 | lipase |
| OPB42771 | T4742_S00012.8 | lipase |
| OPB44495 | T4742_S00003.767 | Lipase 1 [Beauveria bassiana D1-5] |
| OPB42062 | T4742_S00004.164 | lipase 2 |

| | | |
|---|---|---|
| OPB37159 | T4742_S00005.186 | lipase G-D-S-L |
| OPB38760 | T4742_S00002.809 | Lipase, class III |
| OPB45367 | T4742_S00010.278 | Lipase, GDSL |
| OPB42694 | T4742_S00004.800 | lipase, lipocalin related |
| OPB44613 | T4742_S00003.885 | lipase, lipocalin related |
| OPB37071 | T4742_S00005.98 | lipase, putative |
| OPB36261 | T4742_S00021.180 | lipase, secreted |
| OPB43337 | T4742_S00019.70 | lipase, secreted |
| OPB41611 | T4742_S00011.454 | lipase/esterase (α/ß fold) |
| OPB46905 | T4742_S00009.347 | lipase/esterase (α/ß fold) |
| OPB43981 | T4742_S00003.252 | lipase/serine esterase, putative |
| OPB43303 | T4742_S00019.36 | lipid-transfer protein, mitochondrial precursor |
| OPB45253 | T4742_S00010.162 | Lipoate synthase |
| OPB45728 | T4742_S00014.63 | lipoate-protein ligase A, putative |
| OPB36512 | T4742_S00007.229 | Lipopolysaccharide-modifying protein |
| OPB44580 | T4742_S00003.852 | L-isoaspartate(D-aspartate) O-methyltransferase |
| OPB37546 | T4742_S00005.573 | L-lactate dehydrogenase, putative |
| OPB39366 | T4742_S00006.426 | LMBR1 domain-containing protein |
| OPB43220 | T4742_S00022.23 | Long chain fatty acid acyl-CoA ligase |
| OPB45310 | T4742_S00010.221 | Long chain fatty acid acyl-CoA ligase |
| OPB44634 | T4742_S00003.906 | long-chain fatty acid transporter, |
| OPB36296 | T4742_S00007.13 | long-chain-fatty-acid-CoA ligase |
| OPB39728 | T4742_S00015.72 | long-chain-fatty-acid--CoA ligase 2 |
| OPB42453 | T4742_S00004.555 | Low temperature requirement A [Metarhizium robertsii ARSEF 23] |
| OPB45837 | T4742_S00014.172 | low temperature requirement A [Pochonia chlamydosporia 170] |
| OPB40102 | T4742_S00001.181 | low temperature viability protein |
| OPB43791 | T4742_S00003.62 | L-PSP endoribonuclease family protein |
| OPB36825 | T4742_S00007.543 | L-PSP endoribonuclease family protein Brt1 |
| OPB42266 | T4742_S00004.368 | LPXTG-motif cell wall secreted domain protein |
| OPB37002 | T4742_S00005.29 | Lrg1 which is highly expressed in sporulation [Trichoderma guizhouense] |
| OPB40062 | T4742_S00001.141 | L-serine dehydratase [Ceratocystis platani] |
| OPB37350 | T4742_S00005.377 | L-threo-3-deoxy-hexulosonate aldolase |
| OPB37309 | T4742_S00005.336 | lysine decarboxylase |
| OPB46715 | T4742_S00009.157 | lysine methyltransferases (HMKTs) - non SET |
| OPB38088 | T4742_S00002.135 | lysine methyltransferases (HMKTs) - SET domain |
| OPB39935 | T4742_S00001.14 | lysine methyltransferases (HMKTs) - SET domain |

| | | |
|---|---|---|
| OPB44340 | T4742_S00003.611 | lysine methyltransferases (HMKTs) - SET domain |
| OPB44094 | T4742_S00003.365 | lysine N-methyltransferase SET9 |
| OPB44504 | T4742_S00003.776 | LysM domain-containing protein [Trichoderma harzianum] |
| OPB45987 | T4742_S00008.53 | lysophospholipase |
| OPB44866 | T4742_S00013.171 | Lysophospholipase, catalytic region |
| OPB39069 | T4742_S00006.128 | lysophospholipase-like protein |
| OPB47046 | T4742_S00009.488 | Lysyl-tRNA synthetase (class II) |
| OPB41861 | T4742_S00018.184 | macrophomate synthase |
| OPB44182 | T4742_S00003.453 | MADS-box protein |
| OPB40947 | T4742_S00001.1028 | magnesium and cobalt transporter CorA |
| OPB45510 | T4742_S00010.421 | magnesium-dependent phosphatase, putative |
| OPB39878 | T4742_S00015.222 | maintenance of ploidy protein mob1 |
| OPB42132 | T4742_S00004.234 | maintenance of ploidy protein Mob2 (Mob1/phocein family protein) [Colletotrichum tofieldiae] |
| OPB43978 | T4742_S00003.249 | MAK1; MAPK2, mitogen activated protein kinase 2, involved in hyphal fusion |
| OPB37299 | T4742_S00005.326 | MAK16 protein, putative |
| OPB36932 | T4742_S00007.650 | MAK2; MAPK1, mitogen activated protein kinase 1 involved in biocontrol activity in H. virens |
| OPB37801 | T4742_S00017.68 | malate dehydrogenase |
| OPB40371 | T4742_S00001.450 | malate dehydrogenase |
| OPB36290 | T4742_S00007.7 | malate synthase |
| OPB39121 | T4742_S00006.181 | Malate/L-lactate dehydrogenase |
| OPB46486 | T4742_S00008.553 | Maleylacetoacetate isomerase, putative |
| OPB40097 | T4742_S00001.176 | malic enzyme |
| OPB40212 | T4742_S00001.291 | malonyl CoA-acyl carrier protein transacylase [Beauveria bassiana ARSEF 2860] |
| OPB41920 | T4742_S00004.22 | mandelate racemase/muconate lactonase-like protein |
| OPB36013 | T4742_S00023.48 | Mandelate racemase/muconate lactonizing enzyme |
| OPB40714 | T4742_S00001.794 | Mandelate racemase/muconate lactonizing enzyme |
| OPB41464 | T4742_S00011.307 | Mandelate racemase/muconate lactonizing enzyme |
| OPB41350 | T4742_S00011.191 | Mandelate racemase/muconate lactonizing protein |
| OPB46299 | T4742_S00008.366 | Manganese and iron superoxide dismutase |
| OPB44480 | T4742_S00003.752 | manganese lipoxygenase |
| OPB43692 | T4742_S00016.212 | mannitol 1-phosphate dehydrogenase, partial [Trichoderma gamsii] |
| OPB42690 | T4742_S00004.796 | mannitol dehydrogenase LXR1 |
| OPB45641 | T4742_S00010.552 | mannitol-1-phosphate dehydrogenase |
| OPB45566 | T4742_S00010.477 | mannitol-1-phosphate dehydrogenase |
| OPB36301 | T4742_S00007.18 | mannose-1-phosphate guanyltransferase |
| OPB47081 | T4742_S00009.523 | mannose-1-phosphate guanylyltransferase, putative |

| | | |
|---|---|---|
| OPB42871 | T4742_S00012.108 | Mannose-6-phosphate isomerase |
| OPB43799 | T4742_S00003.70 | mannose-6-phosphate isomerase |
| OPB46580 | T4742_S00009.20 | Mannose-binding lectin [Ceraceosorus bombacis] |
| OPB44240 | T4742_S00003.511 | Mannose-P-dolichol utilization defect 1 protein |
| OPB39689 | T4742_S00015.33 | mannosylphosphate transferase |
| OPB44147 | T4742_S00003.418 | mannosylphosphate transferase |
| NCBI_IDs | Old_IDs | manual annotation |
| OPB39236 | T4742_S00006.296 | MaoC-like peroxisomal dehydratase |
| OPB46942 | T4742_S00009.384 | MAPK  mitogen activated protein kinase STE20 |
| OPB38173 | T4742_S00002.220 | MAPK3,  probably involved in stress response and osmosensing, Hog1p |
| OPB36607 | T4742_S00007.325 | MAPKK  MAP kinase kinase MIk1, involved in hyphal fusion |
| OPB41576 | T4742_S00011.419 | MAPKK, hypothetical MAP kinase kinase Pbs2p |
| OPB39520 | T4742_S00006.580 | MAPKK, MAP kinase kinase MEK2, hyphal fusion |
| OPB42344 | T4742_S00004.446 | MAPKK, MAP kinase kinase Mkk1/Mek1 |
| OPB39222 | T4742_S00006.282 | MAPKK, mitogen activated protein kinase kinase Ssk2p |
| OPB41520 | T4742_S00011.363 | MAPKKK mitogen activated protein kinase kinase kinase Ste11/MIK2 |
| OPB38549 | T4742_S00002.598 | mating protein MAT1-2-1 |
| OPB47026 | T4742_S00009.468 | mating type pheromone precursor, alpha type |
| OPB39541 | T4742_S00006.601 | mature-T-cell-proliferation protein |
| OPB38975 | T4742_S00006.34 | MDR multidrug transporter |
| OPB40333 | T4742_S00001.412 | MDR multidrug transporter |
| OPB40726 | T4742_S00001.806 | MDR multidrug transporter |
| OPB40920 | T4742_S00001.1001 | MDR multidrug transporter |
| OPB42293 | T4742_S00004.395 | MDR multidrug transporter |
| OPB43224 | T4742_S00022.27 | MDR multidrug transporter |
| OPB45176 | T4742_S00010.83 | MDR multidrug transporter |
| OPB44726 | T4742_S00013.31 | MDR protein |
| OPB37291 | T4742_S00005.318 | Mechanosensitive ion channel family protein |
| OPB43047 | T4742_S00012.284 | mediator of RNA polymerase II transcription subunit 10, putative |
| OPB39438 | T4742_S00006.498 | mediator of RNA polymerase II transcription subunit 14, putative |
| OPB41616 | T4742_S00011.459 | mediator of RNA polymerase II transcription subunit 31 |
| OPB40603 | T4742_S00001.683 | mediator of RNA polymerase II transcription subunit 7, putative |
| OPB43708 | T4742_S00016.228 | MEI-3, meiosis defective protein |
| OPB42605 | T4742_S00004.709 | Meiosis-specific protein, required for spore wall formation during sporulation, SPO75 |
| OPB39682 | T4742_S00015.26 | meiotic chromosome segregation protein, putative |
| OPB37533 | T4742_S00005.560 | meiotic recombination protein rec12 |

| | | |
|---|---|---|
| OPB37740 | T4742_S00017.7 | meiotically up-regulated protein |
| OPB42363 | T4742_S00004.465 | Member of SIR2 proteins |
| OPB43706 | T4742_S00016.226 | Membrane coat complex Retromer, subunit Vps26 |
| OPB39707 | T4742_S00015.51 | Membrane coat complex Retromer, subunit Vps29 |
| OPB42568 | T4742_S00004.671 | Membrane coat complex Retromer, subunit Vps35 |
| OPB46120 | T4742_S00008.187 | membrane dipeptidase GliJ |
| OPB39022 | T4742_S00006.81 | Membrane protein Erj5 with DnaJ domain |
| OPB41636 | T4742_S00011.479 | Membrane protein, yif1, involved in biogenesis of COPII transport vesicles |
| OPB46606 | T4742_S00009.48 | membrane-bound O-acyltransferase domain-containing protein |
| OPB42009 | T4742_S00004.111 | menaquinone methyltransferase [Epichloe typhina] |
| OPB37056 | T4742_S00005.83 | Met-10+ like-protein |
| OPB45351 | T4742_S00010.262 | Metacaspase-1A |
| OPB42610 | T4742_S00004.714 | metal dependent phosphohydrolase |
| OPB43530 | T4742_S00016.50 | metal dependent phosphohydrolase |
| OPB43788 | T4742_S00003.59 | metal dependent phosphohydrolase |
| OPB36350 | T4742_S00007.67 | metal ion transporter SMF2 |
| OPB44024 | T4742_S00003.295 | metal-dependent amidase/aminoacylase/carbounknown proteinpeptidase |
| OPB43028 | T4742_S00012.265 | Metal-dependent hydrolases belonging to the beta-lactamase superfamily. |
| OPB40365 | T4742_S00001.444 | metal-dependent phosphohydrolase |
| OPB42803 | T4742_S00012.40 | Metallocarboxypeptidase, putative |
| OPB45062 | T4742_S00013.367 | Metallocarboxypeptidase, putative |
| OPB38053 | T4742_S00002.100 | Metallo-dependent phosphatase |
| OPB46373 | T4742_S00008.440 | Metallo-hydrolase/oxidoreductase |
| OPB41969 | T4742_S00004.71 | metallopeptidase |
| OPB41970 | T4742_S00004.72 | metallopeptidase |
| OPB37236 | T4742_S00005.263 | Metallopeptidase M48, Ste24p, involved in a-factor peptide pheromone processing |
| OPB45808 | T4742_S00014.143 | metallopeptidase MepB |
| OPB47030 | T4742_S00009.472 | metallopeptidase MepB |
| OPB42205 | T4742_S00004.307 | metallopeptidase, Ste23 |
| OPB37701 | T4742_S00005.728 | metallophosphoesterase |
| OPB41615 | T4742_S00011.458 | Metallophosphoesterase |
| OPB36025 | T4742_S00023.60 | Metallophosphoesterase |
| OPB38303 | T4742_S00002.350 | metallophosphoesterase domain-containing protein |
| OPB45905 | T4742_S00014.241 | metallophosphoesterase domain-containing protein, putative |
| OPB41326 | T4742_S00011.167 | metalloprotease zincin |
| OPB40821 | T4742_S00001.902 | methionine aminopeptidase |

| | | |
|---|---|---|
| OPB42244 | T4742_S00004.346 | methionine aminopeptidase |
| OPB43957 | T4742_S00003.228 | methionine aminopeptidase |
| OPB37913 | T4742_S00017.180 | methionine aminopeptidase |
| OPB36625 | T4742_S00007.343 | methionine synthase, vitamin-B12 independent |
| OPB43146 | T4742_S00012.383 | methionine synthase, vitamin-B12 independent |
| OPB45748 | T4742_S00014.83 | methionine synthase, vitamin-B12 independent |
| OPB37245 | T4742_S00005.272 | methionine tRNA synthetase (MetRS) |
| OPB37246 | T4742_S00005.273 | methionine tRNA synthetase (MetRS) |
| OPB46953 | T4742_S00009.395 | methionine-R-sulfoxide reductase B2 |
| OPB43991 | T4742_S00003.262 | methionyl-tRNA synthetase. |
| OPB45844 | T4742_S00014.179 | Methylated-DNA-[protein]-cysteine S-methyltransferase |
| OPB36349 | T4742_S00007.66 | Methylene-fatty-acyl-phospholipid synthase |
| OPB38102 | T4742_S00002.149 | Methylenetetrahydrofolate reductase |
| OPB40022 | T4742_S00001.101 | Methylenetetrahydrofolate reductase |
| OPB41275 | T4742_S00011.116 | methylisocitrate lyase |
| OPB45906 | T4742_S00014.242 | methylitaconate delta2-delta3-isomerase |
| OPB41860 | T4742_S00018.183 | Methylmalonate semialdehyde dehydrogenase |
| OPB36483 | T4742_S00007.200 | Methylthioadenosine phosphorylase MTAP |
| OPB42152 | T4742_S00004.254 | methylthioribulose-1-phosphate dehydratase |
| OPB36486 | T4742_S00007.203 | methyltransferase |
| OPB39860 | T4742_S00015.204 | methyltransferase |
| OPB41272 | T4742_S00011.113 | methyltransferase |
| OPB42792 | T4742_S00012.29 | methyltransferase |
| OPB44034 | T4742_S00003.305 | methyltransferase |
| OPB44449 | T4742_S00003.721 | methyltransferase |
| OPB44727 | T4742_S00013.32 | methyltransferase |
| OPB45780 | T4742_S00014.115 | methyltransferase |
| OPB36816 | T4742_S00007.534 | methyltransferase |
| OPB41193 | T4742_S00011.33 | methyltransferase/trans-aconitate 2-methyltransferase |
| OPB44114 | T4742_S00003.385 | meu10, a GPI-anhored cell wall protein. |
| OPB37249 | T4742_S00005.276 | mevalonate kinase |
| OPB43281 | T4742_S00019.14 | MFS  permease |
| OPB46340 | T4742_S00008.407 | MFS allantoate transporter [Pochonia chlamydosporia 170] |
| OPB44523 | T4742_S00003.795 | MFS alpha-glucoside transporter |
| OPB46583 | T4742_S00009.23 | MFS antiporter QDR2 [Madurella mycetomatis] |
| OPB36796 | T4742_S00007.514 | MFS cellobiose transporter Stp1 |

| | | |
|---|---|---|
| OPB41175 | T4742_S00011.15 | MFS cellulose response transporter CTR1 |
| OPB40572 | T4742_S00001.651 | MFS cycloheximide resistance protein |
| OPB43243 | T4742_S00022.46 | MFS drug efflux transporter |
| OPB45322 | T4742_S00010.233 | MFS drug efflux transporter |
| OPB36168 | T4742_S00021.85 | MFS drug efflux transporter [Aspergillus kawachii IFO 4308] |
| OPB41047 | T4742_S00020.71 | MFS drug efflux transporter [Aspergillus kawachii IFO 4308] |
| OPB45969 | T4742_S00008.35 | MFS efflux pump |
| OPB40655 | T4742_S00001.735 | MFS general substrate transporter [Aspergillus ruber CBS 135680] |
| OPB42347 | T4742_S00004.449 | MFS general substrate transporter [Aureobasidium pullulans EXF-150] |
| OPB46934 | T4742_S00009.376 | MFS general substrate transporter [Cenococcum geophilum 1.58] |
| OPB45430 | T4742_S00010.341 | MFS general substrate transporter [Peniophora sp. CONT] |
| OPB42532 | T4742_S00004.634 | MFS gliotoxin efflux transporter [Colletotrichum orbiculare MAFF 240422] |
| OPB43553 | T4742_S00016.73 | MFS H+/oligopeptide transporter |
| OPB40913 | T4742_S00001.994 | MFS hexose transporter |
| OPB36180 | T4742_S00021.98 | MFS hexose transporter CLP1, transports cellodextrins in N. crassa |
| OPB36830 | T4742_S00007.548 | MFS hexose transporter; putative CDT2 cellodextrin transporter |
| OPB47060 | T4742_S00009.502 | MFS high-affinity glucose transporter ght2 |
| OPB40656 | T4742_S00001.736 | MFS inner membrane transporter yfaV |
| OPB36867 | T4742_S00007.585 | MFS monocarboxylate [Trichoderma guizhouense] |
| OPB36204 | T4742_S00021.122 | MFS monocarboxylate transporter |
| OPB40746 | T4742_S00001.827 | MFS monocarboxylate transporter |
| OPB40873 | T4742_S00001.954 | MFS monocarboxylate transporter |
| OPB45787 | T4742_S00014.122 | MFS monocarboxylate transporter |
| OPB46510 | T4742_S00008.577 | MFS monocarboxylate transporter |
| OPB47090 | T4742_S00009.532 | MFS monocarboxylate transporter |
| OPB36334 | T4742_S00007.51 | MFS multidrug resistance protein |
| OPB41300 | T4742_S00011.141 | MFS multidrug resistance protein |
| OPB40540 | T4742_S00001.619 | MFS multidrug transporter |
| OPB41042 | T4742_S00020.66 | MFS multidrug transporter |
| OPB41109 | T4742_S00020.133 | MFS multidrug transporter |
| OPB42287 | T4742_S00004.389 | MFS multidrug transporter |
| OPB43391 | T4742_S00019.124 | MFS multidrug transporter |
| OPB45622 | T4742_S00010.533 | MFS multidrug transporter |
| OPB46009 | T4742_S00008.75 | MFS multidrug transporter |
| OPB46194 | T4742_S00008.261 | MFS multidrug transporter |
| OPB46558 | T4742_S00008.625 | MFS multidrug transporter |

| | | |
|---|---|---|
| OPB47028 | T4742_S00009.470 | MFS multidrug transporter |
| OPB47031 | T4742_S00009.473 | MFS multidrug transporter |
| OPB47097 | T4742_S00009.539 | MFS multidrug transporter |
| OPB43917 | T4742_S00003.188 | MFS multidrug transporter [Trichoderma guizhouense] |
| OPB42368 | T4742_S00004.470 | MFS multidrug transporter-GliA |
| OPB45907 | T4742_S00014.243 | MFS nicotinic acid transporter |
| OPB45922 | T4742_S00014.259 | MFS oligopeptide transporter |
| OPB36264 | T4742_S00021.183 | MFS pantothenate transporter |
| OPB41866 | T4742_S00018.189 | MFS pantothenate transporter |
| OPB41472 | T4742_S00011.315 | MFS peptide transporter |
| OPB43415 | T4742_S00019.148 | MFS peremase, xylose transporter |
| OPB36070 | T4742_S00024.31 | MFS permease |
| OPB36077 | T4742_S00024.38 | MFS permease |
| OPB36103 | T4742_S00021.19 | MFS permease |
| OPB36141 | T4742_S00021.58 | MFS permease |
| OPB36143 | T4742_S00021.60 | MFS permease |
| OPB36159 | T4742_S00021.76 | MFS permease |
| OPB36176 | T4742_S00021.94 | MFS permease |
| OPB36217 | T4742_S00021.135 | MFS permease |
| OPB36273 | T4742_S00021.192 | MFS permease |
| OPB36277 | T4742_S00021.196 | MFS permease |
| OPB36423 | T4742_S00007.140 | MFS permease |
| OPB36433 | T4742_S00007.150 | MFS permease |
| OPB36436 | T4742_S00007.153 | MFS permease |
| OPB36442 | T4742_S00007.159 | MFS permease |
| OPB36461 | T4742_S00007.178 | MFS permease |
| OPB36467 | T4742_S00007.184 | MFS permease |
| OPB36501 | T4742_S00007.218 | MFS permease |
| OPB36597 | T4742_S00007.315 | MFS permease |
| OPB36820 | T4742_S00007.538 | MFS permease |
| OPB36822 | T4742_S00007.540 | MFS permease |
| OPB36826 | T4742_S00007.544 | MFS permease |
| OPB36829 | T4742_S00007.547 | MFS permease |
| OPB37135 | T4742_S00005.162 | MFS permease |
| OPB37289 | T4742_S00005.316 | MFS permease |
| OPB37290 | T4742_S00005.317 | MFS permease |

| | | |
|---|---|---|
| OPB37304 | T4742_S00005.331 | MFS permease |
| OPB37391 | T4742_S00005.418 | MFS permease |
| OPB37547 | T4742_S00005.574 | MFS permease |
| OPB37612 | T4742_S00005.639 | MFS permease |
| OPB37658 | T4742_S00005.685 | MFS permease |
| OPB37661 | T4742_S00005.688 | MFS permease |
| OPB37718 | T4742_S00005.746 | MFS permease |
| OPB37722 | T4742_S00005.750 | MFS permease |
| OPB37726 | T4742_S00005.754 | MFS permease |
| OPB37814 | T4742_S00017.81 | MFS permease |
| OPB37815 | T4742_S00017.82 | MFS permease |
| OPB37887 | T4742_S00017.154 | MFS permease |
| OPB37947 | T4742_S00017.214 | MFS permease |
| OPB37995 | T4742_S00002.42 | MFS permease |
| OPB38031 | T4742_S00002.78 | MFS permease |
| OPB38051 | T4742_S00002.98 | MFS permease |
| OPB38132 | T4742_S00002.179 | MFS permease |
| OPB38242 | T4742_S00002.289 | MFS permease |
| OPB38244 | T4742_S00002.291 | MFS permease |
| OPB38246 | T4742_S00002.293 | MFS permease |
| OPB38336 | T4742_S00002.384 | MFS permease |
| OPB38420 | T4742_S00002.468 | MFS permease |
| OPB38492 | T4742_S00002.541 | MFS permease |
| OPB38569 | T4742_S00002.618 | MFS permease |
| OPB38596 | T4742_S00002.645 | MFS permease |
| OPB38720 | T4742_S00002.769 | MFS permease |
| OPB38748 | T4742_S00002.797 | MFS permease |
| OPB38762 | T4742_S00002.811 | MFS permease |
| OPB38774 | T4742_S00002.823 | MFS permease |
| OPB38867 | T4742_S00002.916 | MFS permease |
| OPB38904 | T4742_S00002.953 | MFS permease |
| OPB38939 | T4742_S00002.988 | MFS permease |
| OPB39050 | T4742_S00006.109 | MFS permease |
| OPB39127 | T4742_S00006.187 | MFS permease |
| OPB39131 | T4742_S00006.191 | MFS permease |
| OPB39137 | T4742_S00006.197 | MFS permease |

| | | |
|---|---|---|
| OPB39311 | T4742_S00006.371 | MFS permease |
| OPB39484 | T4742_S00006.544 | MFS permease |
| OPB39531 | T4742_S00006.591 | MFS permease |
| OPB39853 | T4742_S00015.197 | MFS permease |
| OPB39858 | T4742_S00015.202 | MFS permease |
| OPB39906 | T4742_S00015.250 | MFS permease |
| OPB40074 | T4742_S00001.153 | MFS permease |
| OPB40289 | T4742_S00001.368 | MFS permease |
| OPB40291 | T4742_S00001.370 | MFS permease |
| OPB40339 | T4742_S00001.418 | MFS permease |
| OPB40342 | T4742_S00001.421 | MFS permease |
| OPB40349 | T4742_S00001.428 | MFS permease |
| OPB40418 | T4742_S00001.497 | MFS permease |
| OPB40528 | T4742_S00001.607 | MFS permease |
| OPB40571 | T4742_S00001.650 | MFS permease |
| OPB40591 | T4742_S00001.671 | MFS permease |
| OPB40641 | T4742_S00001.721 | MFS permease |
| OPB40669 | T4742_S00001.749 | MFS permease |
| OPB40701 | T4742_S00001.781 | MFS permease |
| OPB40775 | T4742_S00001.856 | MFS permease |
| OPB40828 | T4742_S00001.909 | MFS permease |
| OPB41034 | T4742_S00020.58 | MFS permease |
| OPB41038 | T4742_S00020.62 | MFS permease |
| OPB41046 | T4742_S00020.70 | MFS permease |
| OPB41122 | T4742_S00020.146 | MFS permease |
| OPB41125 | T4742_S00020.149 | MFS permease |
| OPB41186 | T4742_S00011.26 | MFS permease |
| OPB41194 | T4742_S00011.34 | MFS permease |
| OPB41231 | T4742_S00011.72 | MFS permease |
| OPB41248 | T4742_S00011.89 | MFS permease |
| OPB41269 | T4742_S00011.110 | MFS permease |
| OPB41315 | T4742_S00011.156 | MFS permease |
| OPB41349 | T4742_S00011.190 | MFS permease |
| OPB41370 | T4742_S00011.211 | MFS permease |
| OPB41380 | T4742_S00011.221 | MFS permease |
| OPB41469 | T4742_S00011.312 | MFS permease |

| | | |
|---|---|---|
| OPB41485 | T4742_S00011.328 | MFS permease |
| OPB41641 | T4742_S00011.484 | MFS permease |
| OPB41642 | T4742_S00011.485 | MFS permease |
| OPB41697 | T4742_S00018.19 | MFS permease |
| OPB41746 | T4742_S00018.69 | MFS permease |
| OPB41786 | T4742_S00018.109 | MFS permease |
| OPB41792 | T4742_S00018.115 | MFS permease |
| OPB41835 | T4742_S00018.158 | MFS permease |
| OPB41844 | T4742_S00018.167 | MFS permease |
| OPB41848 | T4742_S00018.171 | MFS permease |
| OPB41875 | T4742_S00018.198 | MFS permease |
| OPB42087 | T4742_S00004.189 | MFS permease |
| OPB42273 | T4742_S00004.375 | MFS permease |
| OPB42335 | T4742_S00004.437 | MFS permease |
| OPB42541 | T4742_S00004.644 | MFS permease |
| OPB42558 | T4742_S00004.661 | MFS permease |
| OPB42560 | T4742_S00004.663 | MFS permease |
| OPB42581 | T4742_S00004.684 | MFS permease |
| OPB42665 | T4742_S00004.770 | MFS permease |
| OPB42702 | T4742_S00004.808 | MFS permease |
| OPB42717 | T4742_S00004.823 | MFS permease |
| OPB42723 | T4742_S00004.829 | MFS permease |
| OPB42746 | T4742_S00004.852 | MFS permease |
| OPB42840 | T4742_S00012.77 | MFS permease |
| OPB42882 | T4742_S00012.119 | MFS permease |
| OPB43258 | T4742_S00022.62 | MFS permease |
| OPB43261 | T4742_S00022.65 | MFS permease |
| OPB43289 | T4742_S00019.22 | MFS permease |
| OPB43301 | T4742_S00019.34 | MFS permease |
| OPB43357 | T4742_S00019.90 | MFS permease |
| OPB43417 | T4742_S00019.150 | MFS permease |
| OPB43435 | T4742_S00019.168 | MFS permease |
| OPB43440 | T4742_S00019.173 | MFS permease |
| OPB43465 | T4742_S00019.198 | MFS permease |
| OPB43466 | T4742_S00019.199 | MFS permease |
| OPB43514 | T4742_S00016.34 | MFS permease |

| | | |
|---|---|---|
| OPB43521 | T4742_S00016.41 | MFS permease |
| OPB43535 | T4742_S00016.55 | MFS permease |
| OPB43537 | T4742_S00016.57 | MFS permease |
| OPB43648 | T4742_S00016.168 | MFS permease |
| OPB43649 | T4742_S00016.169 | MFS permease |
| OPB43691 | T4742_S00016.211 | MFS permease |
| OPB43730 | T4742_S00003.1 | MFS permease |
| OPB43773 | T4742_S00003.44 | MFS permease |
| OPB43810 | T4742_S00003.81 | MFS permease |
| OPB43817 | T4742_S00003.88 | MFS permease |
| OPB43820 | T4742_S00003.91 | MFS permease |
| OPB43822 | T4742_S00003.93 | MFS permease |
| OPB43870 | T4742_S00003.141 | MFS permease |
| OPB43877 | T4742_S00003.148 | MFS permease |
| OPB43878 | T4742_S00003.149 | MFS permease |
| OPB43882 | T4742_S00003.153 | MFS permease |
| OPB44029 | T4742_S00003.300 | MFS permease |
| OPB44120 | T4742_S00003.391 | MFS permease |
| OPB44234 | T4742_S00003.505 | MFS permease |
| OPB44427 | T4742_S00003.699 | MFS permease |
| OPB44485 | T4742_S00003.757 | MFS permease |
| OPB44500 | T4742_S00003.772 | MFS permease |
| OPB44544 | T4742_S00003.816 | MFS permease |
| OPB44597 | T4742_S00003.869 | MFS permease |
| OPB44603 | T4742_S00003.875 | MFS permease |
| OPB44680 | T4742_S00003.952 | MFS permease |
| OPB44691 | T4742_S00003.963 | MFS permease |
| OPB44724 | T4742_S00013.29 | MFS permease |
| OPB44778 | T4742_S00013.83 | MFS permease |
| OPB44781 | T4742_S00013.86 | MFS permease |
| OPB44885 | T4742_S00013.190 | MFS permease |
| OPB44989 | T4742_S00013.294 | MFS permease |
| OPB45001 | T4742_S00013.306 | MFS permease |
| OPB45118 | T4742_S00010.25 | MFS permease |
| OPB45183 | T4742_S00010.90 | MFS permease |
| OPB45257 | T4742_S00010.166 | MFS permease |

| | | |
|---|---|---|
| OPB45318 | T4742_S00010.229 | MFS permease |
| OPB45321 | T4742_S00010.232 | MFS permease |
| OPB45353 | T4742_S00010.264 | MFS permease |
| OPB45360 | T4742_S00010.271 | MFS permease |
| OPB45586 | T4742_S00010.497 | MFS permease |
| OPB45600 | T4742_S00010.511 | MFS permease |
| OPB45612 | T4742_S00010.523 | MFS permease |
| OPB45617 | T4742_S00010.528 | MFS permease |
| OPB45627 | T4742_S00010.538 | MFS permease |
| OPB45642 | T4742_S00010.553 | MFS permease |
| OPB45815 | T4742_S00014.150 | MFS permease |
| OPB45849 | T4742_S00014.184 | MFS permease |
| OPB45866 | T4742_S00014.201 | MFS permease |
| OPB45881 | T4742_S00014.216 | MFS permease |
| OPB45917 | T4742_S00014.253 | MFS permease |
| OPB45920 | T4742_S00014.257 | MFS permease |
| OPB45997 | T4742_S00008.63 | MFS permease |
| OPB46020 | T4742_S00008.86 | MFS permease |
| OPB46027 | T4742_S00008.93 | MFS permease |
| OPB46029 | T4742_S00008.95 | MFS permease |
| OPB46156 | T4742_S00008.223 | MFS permease |
| OPB46188 | T4742_S00008.255 | MFS permease |
| OPB46201 | T4742_S00008.268 | MFS permease |
| OPB46223 | T4742_S00008.290 | MFS permease |
| OPB46238 | T4742_S00008.305 | MFS permease |
| OPB46266 | T4742_S00008.333 | MFS permease |
| OPB46301 | T4742_S00008.368 | MFS permease |
| OPB46325 | T4742_S00008.392 | MFS permease |
| OPB46362 | T4742_S00008.429 | MFS permease |
| OPB46369 | T4742_S00008.436 | MFS permease |
| OPB46460 | T4742_S00008.527 | MFS permease |
| OPB46464 | T4742_S00008.531 | MFS permease |
| OPB46503 | T4742_S00008.570 | MFS permease |
| OPB46542 | T4742_S00008.609 | MFS permease |
| OPB46548 | T4742_S00008.615 | MFS permease |
| OPB46557 | T4742_S00008.624 | MFS permease |

| | | |
|---|---|---|
| OPB46589 | T4742_S00009.29 | MFS permease |
| OPB46754 | T4742_S00009.196 | MFS permease |
| OPB46899 | T4742_S00009.341 | MFS permease |
| OPB46939 | T4742_S00009.381 | MFS permease |
| OPB46981 | T4742_S00009.423 | MFS permease |
| OPB47061 | T4742_S00009.503 | MFS permease |
| OPB47064 | T4742_S00009.506 | MFS permease |
| OPB37773 | T4742_S00017.40 | MFS permease |
| OPB38705 | T4742_S00002.754 | MFS permease |
| OPB38972 | T4742_S00006.31 | MFS permease |
| OPB40849 | T4742_S00001.930 | MFS permease |
| OPB41621 | T4742_S00011.464 | MFS permease |
| OPB43942 | T4742_S00003.213 | MFS permease |
| OPB47004 | T4742_S00009.446 | MFS permease |
| OPB36359 | T4742_S00007.76 | MFS permease |
| OPB36545 | T4742_S00007.262 | MFS permease |
| OPB37356 | T4742_S00005.383 | MFS permease |
| OPB37486 | T4742_S00005.513 | MFS permease |
| OPB38077 | T4742_S00002.124 | MFS permease |
| OPB38602 | T4742_S00002.651 | MFS permease |
| OPB39066 | T4742_S00006.125 | MFS permease |
| OPB40285 | T4742_S00001.364 | MFS permease |
| OPB41253 | T4742_S00011.94 | MFS permease |
| OPB43527 | T4742_S00016.47 | MFS permease |
| OPB43552 | T4742_S00016.72 | MFS permease |
| OPB44368 | T4742_S00003.639 | MFS permease |
| OPB45699 | T4742_S00014.33 | MFS permease |
| OPB45705 | T4742_S00014.39 | MFS permease |
| OPB39672 | T4742_S00015.16 | MFS permease (fucose permease) |
| OPB37372 | T4742_S00005.399 | MFS permease (galactose permease ?) |
| OPB44520 | T4742_S00003.792 | MFS permease (galactose permease ?) |
| OPB40662 | T4742_S00001.742 | MFS permease (glucose permease HXT1) |
| OPB44909 | T4742_S00013.214 | MFS permease (glucose permease HXT1) |
| OPB36050 | T4742_S00024.11 | MFS permease (H+ folate?) |
| OPB36229 | T4742_S00021.147 | MFS permease (maltose permease) |
| OPB43335 | T4742_S00019.68 | MFS permease (maltose permease) |

| | | |
|---|---|---|
| OPB38962 | T4742_S00006.21 | MFS permease (sucrose transporter?) |
| OPB45596 | T4742_S00010.507 | MFS permease [Aspergillus fumigatus var. RP-2014] |
| OPB44334 | T4742_S00003.605 | MFS permease [Metarhizium robertsii] |
| OPB46381 | T4742_S00008.448 | MFS permease [Trichoderma parareesei] |
| OPB45827 | T4742_S00014.162 | MFS permease LIZ1 |
| OPB42804 | T4742_S00012.41 | MFS permease toxin efflux |
| OPB45492 | T4742_S00010.403 | MFS permease, Tetracycline resistance protein, TetB |
| OPB40758 | T4742_S00001.839 | MFS permease/maltose permease MAL61 |
| OPB37678 | T4742_S00005.705 | MFS siderophore iron transporter |
| OPB41763 | T4742_S00018.86 | MFS siderophore iron transporter |
| OPB42570 | T4742_S00004.673 | MFS siderophore iron transporter |
| OPB42789 | T4742_S00012.26 | MFS siderophore iron transporter |
| OPB37207 | T4742_S00005.234 | MFS sugar permease |
| OPB39091 | T4742_S00006.150 | MFS sugar permease |
| OPB40698 | T4742_S00001.778 | MFS sugar permease |
| OPB45614 | T4742_S00010.525 | MFS sugar permease |
| OPB46003 | T4742_S00008.69 | MFS sugar permease |
| OPB46415 | T4742_S00008.482 | MFS sugar permease |
| OPB37545 | T4742_S00005.572 | MFS sugar permease |
| OPB40651 | T4742_S00001.731 | MFS sugar permease (hexose permease) |
| OPB37665 | T4742_S00005.692 | MFS tartrate permease |
| OPB46590 | T4742_S00009.30 | MFS toxin exporter [Hypoxylon sp. EC38] |
| OPB37938 | T4742_S00017.205 | MFS, monocarboxylate transporter |
| OPB38575 | T4742_S00002.624 | MFS, monocarboxylate transporter |
| OPB39685 | T4742_S00015.29 | MFS, monocarboxylate transporter |
| OPB40029 | T4742_S00001.108 | MFS, monocarboxylate transporter |
| OPB41259 | T4742_S00011.100 | MFS, monocarboxylate transporter |
| OPB41766 | T4742_S00018.89 | MFS, monocarboxylate transporter |
| OPB44766 | T4742_S00013.71 | MFS, monocarboxylate transporter |
| OPB46488 | T4742_S00008.555 | MFS, monocarboxylate transporter |
| OPB46710 | T4742_S00009.152 | Mg2+ transporter protein CorA-like/Zinc transport protein ZntB |
| OPB46614 | T4742_S00009.56 | Mg2+ transporter protein, CorA-like |
| OPB40354 | T4742_S00001.433 | Mg2+ transporter protein, CorA-like [Penicillium camemberti] |
| OPB41491 | T4742_S00011.334 | Mgs1, a DNA-dependent ATPase, Okazaki fragment processing |
| OPB43574 | T4742_S00016.94 | Minichromosome loss protein 1 [Escovopsis weberi] |
| OPB42079 | T4742_S00004.181 | MIP transporter |

| | | |
|---|---|---|
| OPB36295 | T4742_S00007.12 | mip1/kog1p, putative guanine nucleotide binding protein |
| OPB39498 | T4742_S00006.558 | mismatched base pair and cruciform DNA recognition protein |
| OPB42406 | T4742_S00004.508 | mitochondrial 37S ribosomal protein S27 |
| OPB37040 | T4742_S00005.67 | mitochondrial (phosphate) carrier |
| OPB36534 | T4742_S00007.251 | mitochondrial 2-oxoglutarate/2-oxoadipate transporter-like protein |
| OPB39943 | T4742_S00001.22 | mitochondrial 40S ribosomal protein [Aspergillus niger]. Possible homologue of yeast SWS2p. |
| OPB38392 | T4742_S00002.440 | mitochondrial acetoacetyl-CoA thiolase-like protein |
| OPB42454 | T4742_S00004.556 | Mitochondrial ATP synthase epsilon chain domain-containing, putative |
| OPB39009 | T4742_S00006.68 | mitochondrial ATPase inhibitor, putative |
| OPB42254 | T4742_S00004.356 | mitochondrial ATP-dependent RNA-helicase mrh4 |
| OPB45556 | T4742_S00010.467 | mitochondrial ATP-dependent RNA-helicase Suv3 |
| OPB38151 | T4742_S00002.198 | Mitochondrial carnitine/acyl carnitine carrier |
| OPB36640 | T4742_S00007.358 | Mitochondrial carnitine-acylcarnitine carrier protein |
| OPB36032 | T4742_S00023.67 | mitochondrial carrier protein |
| OPB36944 | T4742_S00007.662 | mitochondrial carrier protein |
| OPB39814 | T4742_S00015.158 | mitochondrial carrier protein |
| OPB43236 | T4742_S00022.39 | mitochondrial carrier protein |
| OPB43999 | T4742_S00003.270 | mitochondrial carrier protein |
| OPB44108 | T4742_S00003.379 | mitochondrial carrier protein |
| OPB38630 | T4742_S00002.679 | mitochondrial carrier protein |
| OPB43614 | T4742_S00016.134 | mitochondrial carrier protein |
| OPB40772 | T4742_S00001.853 | mitochondrial carrier protein (Pet8) [Metarhizium guizhouense ARSEF 977] |
| OPB42033 | T4742_S00004.135 | mitochondrial carrier protein LEU5 |
| OPB44932 | T4742_S00013.237 | mitochondrial carrier protein, putative |
| OPB43183 | T4742_S00012.420 | mitochondrial chaperone Frataxin, putative |
| OPB42459 | T4742_S00004.561 | mitochondrial co-chaperone GrpE , putative |
| OPB43962 | T4742_S00003.233 | mitochondrial cytochrome b2, putative |
| OPB42199 | T4742_S00004.301 | mitochondrial cytochrome c oxidase assembly factor, putative |
| OPB45688 | T4742_S00014.22 | Mitochondrial cytochrome c oxidase subunit VIIc |
| OPB46799 | T4742_S00009.241 | Mitochondrial deoxynucleotide carrier protein |
| OPB39761 | T4742_S00015.105 | Mitochondrial dicarboxylate:inorganic phosphate antiporter (malate, succinate, oxaloacetate) |
| OPB39609 | T4742_S00006.669 | mitochondrial distribution and morphology protein |
| OPB45167 | T4742_S00010.74 | mitochondrial DNA helicase (Pif1) |
| OPB39733 | T4742_S00015.77 | mitochondrial DNA replication protein (Yhm2), putative |
| OPB37917 | T4742_S00017.184 | mitochondrial elongation factor G. |
| OPB37058 | T4742_S00005.85 | mitochondrial exoribonuclease Cyt-4 |

| | | |
|---|---|---|
| OPB39889 | T4742_S00015.233 | mitochondrial F1F0 ATP synthase subunit F Atp17 |
| OPB36738 | T4742_S00007.456 | Mitochondrial F1F0-ATP synthase, subunit c/ATP9/proteolipid |
| OPB44217 | T4742_S00003.488 | mitochondrial hypoxia responsive domain-containing protein |
| OPB44418 | T4742_S00003.690 | mitochondrial import inner membrane translocase subunit TIM10 |
| OPB39370 | T4742_S00006.430 | mitochondrial import inner membrane translocase subunit TIM13 / Zn-finger, Tim10/DDP type |
| OPB39420 | T4742_S00006.480 | mitochondrial import inner membrane translocase subunit tim-14 |
| OPB36619 | T4742_S00007.337 | mitochondrial import inner membrane translocase subunit tim16 |
| OPB40479 | T4742_S00001.558 | mitochondrial import inner membrane translocase subunit tim-17 |
| OPB42600 | T4742_S00004.704 | Mitochondrial import inner membrane translocase subunit tim-50 |
| OPB37403 | T4742_S00005.430 | Mitochondrial import inner membrane translocase, subunit Tim17/22 |
| OPB41512 | T4742_S00011.355 | Mitochondrial import inner membrane translocase, subunit Tim17/22 |
| OPB40476 | T4742_S00001.555 | Mitochondrial import inner membrane translocase, subunit Tim44 |
| OPB47075 | T4742_S00009.517 | Mitochondrial import protein Pam17 |
| OPB40136 | T4742_S00001.215 | mitochondrial import receptor subunit TOM40 |
| OPB36986 | T4742_S00005.13 | mitochondrial inner import translocase TIM54 |
| OPB37518 | T4742_S00005.545 | Mitochondrial inner membrane insertase |
| OPB42250 | T4742_S00004.352 | Mitochondrial inner membrane protease atp-23 |
| OPB42026 | T4742_S00004.128 | mitochondrial inner membrane protease subunit Imp2 |
| OPB40023 | T4742_S00001.102 | mitochondrial leucyl-tRNA synthetase. |
| OPB46877 | T4742_S00009.319 | Mitochondrial Matrix Factor |
| OPB37411 | T4742_S00005.438 | mitochondrial matrix iron-sulfur protein |
| OPB38026 | T4742_S00002.73 | mitochondrial membrane fission protein (Fis1), putative |
| OPB37256 | T4742_S00005.283 | mitochondrial mRNA processing protein PET127, putative |
| OPB39431 | T4742_S00006.491 | mitochondrial NADH-ubiquinone oxidoreductase 20 kD subunit |
| OPB43926 | T4742_S00003.197 | mitochondrial NADP-dependent isocitrate dehydrogenase |
| OPB39625 | T4742_S00006.685 | Mitochondrial outer membrane and cell wall localized SUN family member required for mitochondrial autophagy |
| OPB46074 | T4742_S00008.141 | mitochondrial oxaloacetate/sulfate/thiosulfate transporter-like protein |
| OPB36561 | T4742_S00007.278 | Mitochondrial oxoglutarate/malate carrier proteins |
| OPB44636 | T4742_S00003.908 | mitochondrial peroxiredoxin PRX1 |
| OPB39660 | T4742_S00015.4 | mitochondrial phosphate carrier |
| OPB37085 | T4742_S00005.112 | mitochondrial precursor proteins import receptor |
| OPB37238 | T4742_S00005.265 | mitochondrial presequence protease CYM1 |
| OPB46711 | T4742_S00009.153 | mitochondrial processing peptidase, α-subunit |
| OPB43956 | T4742_S00003.227 | mitochondrial protein sorting (Msf1) |
| OPB36413 | T4742_S00007.130 | mitochondrial Rho GTPase1 |
| OPB42309 | T4742_S00004.411 | Mitochondrial ribosomal protein L17 |

| | | |
|---|---|---|
| OPB43098 | T4742_S00012.335 | Mitochondrial ribosomal protein L43 |
| OPB37999 | T4742_S00002.46 | mitochondrial ribosomal protein MRPL3. |
| OPB41555 | T4742_S00011.398 | mitochondrial ribosomal protein MRPL31 (S. cerevisiae). |
| OPB41989 | T4742_S00004.91 | mitochondrial ribosomal protein MRPL6. |
| OPB42987 | T4742_S00012.224 | Mitochondrial ribosomal protein of the small subunit, has similarity to mammalian apoptosis mediator proteins |
| OPB45250 | T4742_S00010.159 | mitochondrial ribosomal protein RSM22. |
| OPB45708 | T4742_S00014.42 | mitochondrial ribosomal protein RSM25. |
| OPB45764 | T4742_S00014.99 | mitochondrial ribosomal protein S19/S15. |
| OPB36861 | T4742_S00007.579 | mitochondrial ribosomal protein subunit S4 |
| OPB36970 | T4742_S00007.688 | Mitochondrial substrate carrier |
| OPB39637 | T4742_S00006.697 | Mitochondrial substrate carrier |
| OPB42027 | T4742_S00004.129 | Mitochondrial substrate carrier |
| OPB44192 | T4742_S00003.463 | Mitochondrial substrate carrier |
| OPB39043 | T4742_S00006.102 | Mitochondrial succinate/fumarate antiporter |
| OPB37328 | T4742_S00005.355 | mitochondrial translation optimization protein (Mto1) |
| OPB46601 | T4742_S00009.43 | mitochondrial translation optimization protein [Neurospora crassa OR74A] |
| OPB44583 | T4742_S00003.855 | mitochondrial tricarboxylate transporter (Ctp), putative |
| OPB46251 | T4742_S00008.318 | Mitochondrially-localized peptidyl-tRNA hydrolases |
| OPB44302 | T4742_S00003.573 | mitochondrial-processing peptidase subunit beta |
| OPB40147 | T4742_S00001.226 | mitochonfrial import receptor |
| OPB45873 | T4742_S00014.208 | mitogen-activated protein kinase kinase kinase |
| OPB39495 | T4742_S00006.555 | mitogen-activated protein kinase MAF1 , putative |
| OPB42024 | T4742_S00004.126 | mitotic checkpoint protein BUB3 |
| OPB41816 | T4742_S00018.139 | mitotic spindle checkpoint component mad2 |
| OPB36487 | T4742_S00007.204 | MMms2, chromatin related sumoylation |
| OPB38345 | T4742_S00002.393 | MMR; HR regulation |
| OPB39688 | T4742_S00015.32 | MMR; HR regulation |
| OPB43716 | T4742_S00016.236 | MMR; HR regulation |
| OPB44649 | T4742_S00003.921 | MMR; HR regulation |
| OPB36881 | T4742_S00007.599 | MMR; inhibition of homeologous recombination |
| OPB42992 | T4742_S00012.229 | MMR; inhibition of homeologous recombination |
| OPB45840 | T4742_S00014.175 | MMS4, interacts with MUS81 |
| OPB44914 | T4742_S00013.219 | Mn superoxide dismutase |
| OPB43667 | T4742_S00016.187 | Mn2+ homeostasis protein Per1 |
| OPB38592 | T4742_S00002.641 | Mob1/phocein family protein |
| OPB38408 | T4742_S00002.456 | Mob1/phocein family protein [Trichoderma guizhouense] |

| | | |
|---|---|---|
| OPB44776 | T4742_S00013.81 | Modin [Podospora anserina] |
| OPB37487 | T4742_S00005.514 | Molecular chaperone (DnaJ superfamily) |
| OPB46172 | T4742_S00008.239 | Molecular chaperone Bip |
| OPB43113 | T4742_S00012.350 | Molecular chaperone Hsp70 family Lhs1 |
| OPB42017 | T4742_S00004.119 | Molecular chaperone KAR2 |
| OPB39987 | T4742_S00001.66 | molecular chaperone, contains ABC-1 domain |
| OPB38011 | T4742_S00002.58 | Molybdenum cofactor biosynthesis protein |
| OPB38952 | T4742_S00006.11 | Molybdenum cofactor biosynthesis protein |
| OPB47076 | T4742_S00009.518 | Molybdenum cofactor biosynthesis protein |
| OPB45211 | T4742_S00010.119 | Molybdopterin biosynthesis MoaE |
| OPB37045 | T4742_S00005.72 | molybdopterin synthase small subunit CnxG, putative |
| OPB41754 | T4742_S00018.77 | monoamine oxidase |
| OPB43409 | T4742_S00019.142 | monoamine oxidase |
| OPB41719 | T4742_S00018.42 | monodehydroascorbate reductase |
| OPB37099 | T4742_S00005.126 | Mrc1p [Trichoderma guizhouense] |
| OPB36668 | T4742_S00007.386 | mRNA 3'-end-processing protein RNA14, putative |
| OPB43105 | T4742_S00012.342 | mRNA binding protein Pumilio 2, putative |
| OPB36412 | T4742_S00007.129 | mRNA capping enzyme |
| OPB42240 | T4742_S00004.342 | mRNA capping enzyme |
| OPB45440 | T4742_S00010.351 | mRNA cleavage factor complex II protein Clp1, putative |
| OPB45468 | T4742_S00010.379 | mRNA cleavage factor complex II protein Clp1, putative |
| OPB38236 | T4742_S00002.283 | mRNA decapping hydrolase, putative |
| OPB42073 | T4742_S00004.175 | mRNA export factor MEX67 [Beauveria bassiana D1-5] |
| OPB45236 | T4742_S00010.145 | mRNA nucleus export ATPase |
| OPB36862 | T4742_S00007.580 | MRP4 encoding mitochondrial ribosomal protein of the small subunit. |
| OPB36559 | T4742_S00007.276 | MRP-type ABC transporter |
| OPB36775 | T4742_S00007.493 | MRP-type ABC transporter |
| OPB37216 | T4742_S00005.243 | MRP-type ABC transporter |
| OPB37390 | T4742_S00005.417 | MRP-type ABC transporter |
| OPB38353 | T4742_S00002.401 | MRP-type ABC transporter |
| OPB39175 | T4742_S00006.235 | MRP-type ABC transporter |
| OPB40128 | T4742_S00001.207 | MRP-type ABC transporter |
| OPB41502 | T4742_S00011.345 | MRP-type ABC transporter |
| OPB41503 | T4742_S00011.346 | MRP-type ABC transporter |
| OPB41507 | T4742_S00011.350 | MRP-type ABC transporter |
| OPB44265 | T4742_S00003.536 | MRP-type ABC transporter |

| | | |
|---|---|---|
| OPB45356 | T4742_S00010.267 | MRP-type ABC transporter |
| OPB46926 | T4742_S00009.368 | MRP-type ABC transporter |
| OPB42661 | T4742_S00004.766 | MRSP1/expansin-like |
| OPB46883 | T4742_S00009.325 | MRSP1/expansin-like |
| OPB46925 | T4742_S00009.367 | MRSP1/expansin-like |
| OPB39042 | T4742_S00006.101 | MRSP1/expansin-like protein [Trichoderma guizhouense] |
| OPB35960 | T4742_S00025.33 | MSC7, involved in meiotic recombination |
| OPB42031 | T4742_S00004.133 | MT-A70 |
| OPB43021 | T4742_S00012.258 | mtDNA repair protein |
| OPB44604 | T4742_S00003.876 | multicopper oxidase type 1, secreted |
| OPB45372 | T4742_S00010.283 | multisynthetase complex auxiliary component p43 |
| OPB43323 | T4742_S00019.56 | MUS26 |
| OPB43661 | T4742_S00016.181 | Mus81p, a subunit of the Mus81-Mms4 structure-specific endonuclease funtioning in mitotic and meiotic recombination |
| OPB45158 | T4742_S00010.65 | MutS-related protein involved in mismatch repair |
| OPB36452 | T4742_S00007.169 | Myb transcriptional regulator |
| OPB38459 | T4742_S00002.507 | myb transcriptional regulator |
| OPB38518 | T4742_S00002.567 | Myb transcriptional regulator |
| OPB39986 | T4742_S00001.65 | myb transcriptional regulator |
| OPB40890 | T4742_S00001.971 | Myb transcriptional regulator |
| OPB40891 | T4742_S00001.972 | Myb transcriptional regulator |
| OPB41984 | T4742_S00004.86 | myb transcriptional regulator |
| OPB42296 | T4742_S00004.398 | myb transcriptional regulator |
| OPB42508 | T4742_S00004.610 | myb transcriptional regulator |
| OPB42513 | T4742_S00004.615 | myb transcriptional regulator |
| OPB43018 | T4742_S00012.255 | Myb transcriptional regulator |
| OPB43171 | T4742_S00012.408 | Myb transcriptional regulator |
| OPB44645 | T4742_S00003.917 | myb transcriptional regulator |
| OPB39466 | T4742_S00006.526 | MYG1 protein [Trichoderma guizhouense] |
| OPB44069 | T4742_S00003.340 | MYND-type Zn-finger protein |
| OPB42569 | T4742_S00004.672 | myo-inositol oxygenase , |
| OPB37100 | T4742_S00005.127 | Myo-inositol-1-phosphate synthase |
| OPB37894 | T4742_S00017.161 | myosin heavy chain |
| OPB41588 | T4742_S00011.431 | myosin heavy chain |
| OPB45091 | T4742_S00013.396 | myosin heavy chain |
| OPB45365 | T4742_S00010.276 | Myosin tail region-interacting protein MTI1 [Colletotrichum chlorophyti] |
| OPB44213 | T4742_S00003.484 | Myristoyl-CoA:protein N-myristoyltransferase (nmt) |

| | | |
|---|---|---|
| OPB36951 | T4742_S00007.669 | N(6)-adenine-specific DNA methyltransferase |
| OPB36247 | T4742_S00021.165 | N,N-dimethylformamidase beta subunit [Phialophora attae] |
| OPB38371 | T4742_S00002.419 | N2,N2-dimethylguanosine tRNA methyltransferase. |
| OPB36793 | T4742_S00007.511 | Na/K ATPase alpha 1 subunit, |
| OPB38559 | T4742_S00002.608 | Na+/proline symporter PutP |
| OPB39126 | T4742_S00006.186 | Na+/proline symporter PutP |
| OPB36373 | T4742_S00007.90 | N-acetyl-glucosamine-6-phosphate deacetylase |
| OPB43662 | T4742_S00016.182 | N-acetylglucosamine-phosphate mutase |
| OPB36698 | T4742_S00007.416 | N-acetylglucosaminyl transferase component Gpi1 |
| OPB41074 | T4742_S00020.98 | N-acetylglucosaminyl-phosphatidylinositol deacetylase, putative |
| OPB45314 | T4742_S00010.225 | N-acetylglucosaminyltransferase |
| OPB37435 | T4742_S00005.462 | N-acetyltransferase activity |
| OPB40478 | T4742_S00001.557 | N-acetyltransferase activity |
| OPB40426 | T4742_S00001.505 | N-acetyltransferase of bacterial origin |
| OPB45751 | T4742_S00014.86 | NACHT and ankyrin domain protein |
| OPB47171 | T4742_S00009.613 | NACHT and TPR domain protein |
| OPB40455 | T4742_S00001.534 | NACHT and TPR domain protein [Trichoderma guizhouense] |
| OPB45883 | T4742_S00014.218 | NACHT and TPR domain-containing protein [Colletotrichum gloeosporioides Cg-14] |
| OPB38979 | T4742_S00006.38 | NACHT and WD domain protein |
| OPB38161 | T4742_S00002.208 | NACHT and WD40 domain protein HET-R |
| OPB37082 | T4742_S00005.109 | NACHT domain protein |
| OPB39620 | T4742_S00006.680 | NACHT domain protein |
| OPB39838 | T4742_S00015.182 | NACHT domain protein |
| OPB44252 | T4742_S00003.523 | NACHT domain protein |
| OPB45189 | T4742_S00010.96 | NACHT domain protein |
| OPB45582 | T4742_S00010.493 | NACHT domain protein |
| OPB45775 | T4742_S00014.110 | NACHT domain protein |
| OPB46634 | T4742_S00009.76 | NACHT domain protein |
| OPB38139 | T4742_S00002.186 | NACHT domain WD40 repeat-containing protein |
| OPB42127 | T4742_S00004.229 | NACHT domain WD40 repeat-containing protein, related to HET |
| OPB36150 | T4742_S00021.67 | NACHT nucleoside triphosphatase |
| OPB38075 | T4742_S00002.122 | NACHT nucleoside triphosphatase |
| OPB44990 | T4742_S00013.295 | NACHT nucleoside triphosphatase |
| OPB39320 | T4742_S00006.380 | NACHT/WD domain containing protein |
| OPB38497 | T4742_S00002.546 | NAC-ß, Nascent polypeptide-associated complex subunit beta |
| OPB36010 | T4742_S00023.45 | N-acyl-phosphatidylethanolamine-hydrolyzing phospholipase D, putative |

| | | |
|---|---|---|
| OPB37097 | T4742_S00005.124 | N-acylsphingosine amidohydrolase |
| OPB36069 | T4742_S00024.30 | NAD dependent epimerase/dehydratase |
| OPB37427 | T4742_S00005.454 | NAD dependent epimerase/dehydratase |
| OPB37584 | T4742_S00005.611 | NAD dependent epimerase/dehydratase |
| OPB37928 | T4742_S00017.195 | NAD dependent epimerase/dehydratase |
| OPB40326 | T4742_S00001.405 | NAD dependent epimerase/dehydratase |
| OPB40564 | T4742_S00001.643 | NAD dependent epimerase/dehydratase |
| OPB40817 | T4742_S00001.898 | NAD dependent epimerase/dehydratase |
| OPB40875 | T4742_S00001.956 | NAD dependent epimerase/dehydratase |
| OPB41237 | T4742_S00011.78 | NAD dependent epimerase/dehydratase |
| OPB41796 | T4742_S00018.119 | NAD dependent epimerase/dehydratase |
| OPB45660 | T4742_S00010.571 | NAD dependent epimerase/dehydratase |
| OPB39780 | T4742_S00015.124 | NAD dependent epimerase/dehydratase [Beauveria bassiana ARSEF 2860] |
| OPB47153 | T4742_S00009.595 | NAD dependent epimerase/dehydratase [Beauveria bassiana ARSEF 2860] |
| OPB40546 | T4742_S00001.625 | NAD dependent epimerase/dehydratase [Colletotrichum gloeosporioides Cg-14] |
| OPB46448 | T4742_S00008.515 | NAD dependent epimerase/dehydratase [Trichoderma gamsii] |
| OPB40781 | T4742_S00001.862 | NAD dependent epimerase/dehydratase [Trichoderma harzianum] |
| OPB42284 | T4742_S00004.386 | NAD dependent oxidoreductase |
| OPB40049 | T4742_S00001.128 | NAD kinase |
| OPB37604 | T4742_S00005.631 | NAD(P) transhydrogenase beta subunit |
| OPB45424 | T4742_S00010.335 | NAD(P)-binding |
| OPB40578 | T4742_S00001.658 | NAD(P)-dependent oxidoreductase [Paenibacillus sp. FSL R7-0337] |
| OPB42302 | T4742_S00004.404 | NAD(P)H-dependent FMN reductase LOT6, putative |
| OPB41731 | T4742_S00018.54 | NAD-binding protein |
| OPB45431 | T4742_S00010.342 | NAD-binding Rossmann fold oxidoreductase |
| OPB36058 | T4742_S00024.19 | NAD-dependent 15-hydroxyprostaglandin dehydrogenase |
| OPB44923 | T4742_S00013.228 | NAD-dependent 15-hydroxyprostaglandin dehydrogenase |
| OPB45484 | T4742_S00010.395 | NAD-dependent epimerase |
| OPB36330 | T4742_S00007.47 | NAD-dependent epimerase/dehydratase |
| OPB37704 | T4742_S00005.732 | NAD-dependent epimerase/dehydratase |
| OPB40738 | T4742_S00001.818 | NAD-dependent epimerase/dehydratase |
| OPB41293 | T4742_S00011.134 | NAD-dependent epimerase/dehydratase |
| OPB42191 | T4742_S00004.293 | NAD-dependent epimerase/dehydratase |
| OPB44796 | T4742_S00013.101 | NAD-dependent epimerase/dehydratase |
| OPB44803 | T4742_S00013.108 | NAD-dependent epimerase/dehydratase |
| OPB45768 | T4742_S00014.103 | NAD-dependent epimerase/dehydratase |

| | | |
|---|---|---|
| OPB45791 | T4742_S00014.126 | NAD-dependent epimerase/dehydratase |
| OPB45792 | T4742_S00014.127 | NAD-dependent epimerase/dehydratase |
| OPB41121 | T4742_S00020.145 | NAD-dependent epimerase/dehydratase [Penicillium occitanis] |
| OPB44524 | T4742_S00003.796 | NAD-dependent epimerase/dehydratase [Talaromyces cellulolyticus] |
| OPB41095 | T4742_S00020.119 | NAD-dependent glutamate dehydrogenase |
| OPB37488 | T4742_S00005.515 | NAD-dependent oxidoreductase |
| OPB38543 | T4742_S00002.592 | NAD-dependent oxidoreductase |
| OPB40107 | T4742_S00001.186 | NAD-dependent oxidoreductase |
| OPB40538 | T4742_S00001.617 | NAD-dependent oxidoreductase |
| OPB43643 | T4742_S00016.163 | NAD-dependent oxidoreductase |
| OPB44722 | T4742_S00013.27 | NAD-dependent oxidoreductase |
| OPB44800 | T4742_S00013.105 | NAD-dependent oxidoreductase |
| OPB47157 | T4742_S00009.599 | NAD-dependent oxidoreductase |
| OPB44561 | T4742_S00003.833 | NADH dehydrogenase (ubiquinone) 1 alpha subcomplex, 13, cell death-regulatory protein GRIM19 |
| OPB42488 | T4742_S00004.590 | NADH dehydrogenase (ubiquinone) Fe-S protein 1 |
| OPB41902 | T4742_S00004.4 | NADH dehydrogenase iron-sulfur protein |
| OPB41112 | T4742_S00020.136 | NADH:flavin oxidoreductase/12-oxophytodienoate reductase |
| OPB42354 | T4742_S00004.456 | NADH:flavin oxidoreductase/12-oxophytodienoate reductase |
| OPB36155 | T4742_S00021.72 | NADH:flavin oxidoreductase/NADH oxidase |
| OPB38770 | T4742_S00002.819 | NADH:flavin oxidoreductase/NADH oxidase |
| OPB41241 | T4742_S00011.82 | NADH:flavin oxidoreductase/NADH oxidase |
| OPB42291 | T4742_S00004.393 | NADH:flavin oxidoreductase/NADH oxidase |
| OPB42687 | T4742_S00004.793 | NADH:flavin oxidoreductase/NADH oxidase |
| OPB42770 | T4742_S00012.7 | NADH:flavin oxidoreductase/NADH oxidase |
| OPB37065 | T4742_S00005.92 | NADH:flavin oxidoreductase/NADH oxidase |
| OPB39162 | T4742_S00006.222 | NADH:flavin oxidoreductase/NADH oxidase |
| OPB39312 | T4742_S00006.372 | NADH:flavin oxidoreductase/NADH oxidase |
| OPB40770 | T4742_S00001.851 | NADH:flavin oxidoreductase/NADH oxidase |
| OPB43420 | T4742_S00019.153 | NADH:flavin oxidoreductase/NADH oxidase |
| OPB44758 | T4742_S00013.63 | NADH:flavin oxidoreductase/NADH oxidase |
| OPB44822 | T4742_S00013.127 | NADH:flavin oxidoreductase/NADH oxidase |
| OPB46553 | T4742_S00008.620 | NADH:flavin oxidoreductase/NADH oxidase [Colletotrichum fioriniae PJ7] |
| OPB37605 | T4742_S00005.632 | NADH:ubiquinone oxidoreductase 17.2 kD subunit |
| OPB47014 | T4742_S00009.456 | NADH:ubiquinone oxidoreductase 20.1kD subunit, putative |
| OPB43874 | T4742_S00003.145 | NADH:ubiquinone oxidoreductase, NDUFB7/B18 subunit |
| OPB37556 | T4742_S00005.583 | NADH2 dehydrogenase 14k chain |

| | | |
|---|---|---|
| OPB42237 | T4742_S00004.339 | NADH-cytochrome b5 reductase |
| OPB44376 | T4742_S00003.647 | NADH-cytochrome b5 reductase, putative |
| OPB37628 | T4742_S00005.655 | NADH-dehydrogenase (ubiquinone) |
| OPB39088 | T4742_S00006.147 | NADH-dehydrogenase (ubiquinone) |
| OPB41790 | T4742_S00018.113 | NADH-dependent flavin oxidoreductase |
| OPB44575 | T4742_S00003.847 | NADH-quinone oxidoreductase |
| OPB43993 | T4742_S00003.264 | NADH-ubiquinone oxidoreductase |
| OPB41598 | T4742_S00011.441 | NADH-ubiquinone oxidoreductase 10.5 kDa subunit |
| OPB46842 | T4742_S00009.284 | NADH-ubiquinone oxidoreductase 12 kDa subunit |
| OPB39888 | T4742_S00015.232 | NADH-ubiquinone oxidoreductase 18 kDa subunit, putative |
| OPB44079 | T4742_S00003.350 | NADH-ubiquinone oxidoreductase 24 kDa subunit, mitochondrial |
| OPB37957 | T4742_S00002.4 | NADH-ubiquinone oxidoreductase 299 kDa subunit, putative |
| OPB36956 | T4742_S00007.674 | NADH-ubiquinone oxidoreductase 40 kDa subunit-like protein |
| OPB41346 | T4742_S00011.187 | NADH-ubiquinone oxidoreductase 9.5 kDa subunit, putative |
| OPB45127 | T4742_S00010.34 | NADH-ubiquinone oxidoreductase B12 subunit, putative |
| OPB42226 | T4742_S00004.328 | NADH-ubiquinone oxidoreductase Complex1 subunit |
| OPB42899 | T4742_S00012.136 | NADH-ubiquinone oxidoreductase, chain 49kDa |
| OPB45629 | T4742_S00010.540 | NADP dependent alcohol dehydrogenase C2 |
| OPB37537 | T4742_S00005.564 | NADP oxidoreductase, coenzyme F420-dependent |
| OPB41227 | T4742_S00011.68 | NADP oxidoreductase, coenzyme F420-dependent |
| OPB42669 | T4742_S00004.774 | NADP/FAD dependent oxidoreductase |
| OPB42511 | T4742_S00004.613 | NADP:D-xylose dehydrogenase |
| OPB40079 | T4742_S00001.158 | NADP-glutamate dehydrogenase |
| OPB38272 | T4742_S00002.319 | NADPH cytochrome P450 oxidoreductase |
| OPB41884 | T4742_S00018.207 | NADPH dehydrogenase |
| OPB36463 | T4742_S00007.180 | NADPH oxidase |
| OPB40972 | T4742_S00001.1053 | NADPH oxidase regulator NoxR |
| OPB41228 | T4742_S00011.69 | NADPH:quinone oxidoreductase |
| OPB45623 | T4742_S00010.534 | NADPH2:quinone reductase [Exophiala dermatitidis NIH/UT8656] |
| OPB43489 | T4742_S00016.9 | NADPH-adrenodoxin reductase, putative |
| OPB42787 | T4742_S00012.24 | NADPH-dependent aldehyde reductase [Fusarium fujikuroi] |
| OPB43355 | T4742_S00019.88 | NADPH-dependent FMN reductase |
| OPB44959 | T4742_S00013.264 | NADPH-dependent FMN reductase Lot6 |
| OPB40206 | T4742_S00001.285 | nce2,  involved in secretion of proteins that lack classical secretory signal sequences |
| OPB38355 | T4742_S00002.403 | NDT80/PhoG like DNA-binding domain-containing protein |
| OPB42911 | T4742_S00012.148 | NEDD8-activating enzyme E1 catalytic subunit |

| | | |
|---|---|---|
| OPB41931 | T4742_S00004.33 | Neddylin (ubiquitin-like protein). |
| OPB36072 | T4742_S00024.33 | N-ethylmaleimide reductase |
| OPB36323 | T4742_S00007.40 | N-ethylmaleimide reductase |
| OPB39161 | T4742_S00006.221 | N-ethylmaleimide reductase |
| OPB44760 | T4742_S00013.65 | N-ethylmaleimide reductase |
| OPB36603 | T4742_S00007.321 | neutral metallopeptidase NMP1 |
| OPB38721 | T4742_S00002.770 | Nexin Snx3 |
| OPB44143 | T4742_S00003.414 | nexin,Snx4; involved in maturation of the vacuolar aminopeptidase I in yeast |
| OPB44980 | T4742_S00013.285 | nexin-1-like protein Vps5 |
| OPB42239 | T4742_S00004.341 | nexin-41 |
| OPB46789 | T4742_S00009.231 | N-formyltyrosine cytochrome P450 oxidase |
| OPB42923 | T4742_S00012.160 | N-glycosylase/DNA lyase |
| OPB46252 | T4742_S00008.319 | nicotinamide riboside kinase 1, putative |
| OPB44204 | T4742_S00003.475 | nicotinate phosphoribosyltransferase |
| OPB45313 | T4742_S00010.224 | nicotinate-nucleotide diphosphorylase |
| OPB39499 | T4742_S00006.559 | NifS Cysteine sulfinate desulfinase/cysteine desulfurase and related enzymes |
| OPB45282 | T4742_S00010.193 | NIPSNAP family protein [Aschersonia aleyrodis RCEF 2490] |
| OPB43770 | T4742_S00003.41 | nitrate reductase |
| OPB43768 | T4742_S00003.39 | nitrate transporter |
| OPB37931 | T4742_S00017.198 | nitrilase |
| OPB39867 | T4742_S00015.211 | nitrilase |
| OPB40113 | T4742_S00001.192 | nitrilase |
| OPB40829 | T4742_S00001.910 | nitrilase |
| OPB41304 | T4742_S00011.145 | nitrilase |
| OPB41305 | T4742_S00011.146 | nitrilase |
| OPB41364 | T4742_S00011.205 | nitrilase |
| OPB42384 | T4742_S00004.486 | nitrilase |
| OPB43755 | T4742_S00003.26 | nitrilase |
| OPB43794 | T4742_S00003.65 | nitrilase |
| OPB45774 | T4742_S00014.109 | nitrilase |
| OPB46357 | T4742_S00008.424 | nitrilase |
| OPB46491 | T4742_S00008.558 | nitrilase |
| OPB46495 | T4742_S00008.562 | nitrilase |
| OPB40121 | T4742_S00001.200 | nitrogen metabolite repression regulator NmrA |
| OPB44452 | T4742_S00003.724 | nitrogen permease regulator Npr2, putative |
| OPB36199 | T4742_S00021.117 | nitroreductase family protein |

| | | |
|---|---|---|
| OPB38387 | T4742_S00002.435 | NMD3 family protein (nonsense-mediated mRNA decay protein) |
| OPB45255 | T4742_S00010.164 | N-methylhydantoinase A |
| OPB36167 | T4742_S00021.84 | NmrA family protein |
| OPB36203 | T4742_S00021.121 | NmrA family protein |
| OPB37702 | T4742_S00005.729 | NmrA family protein |
| OPB40883 | T4742_S00001.964 | NmrA family protein |
| OPB43687 | T4742_S00016.207 | NmrA family protein |
| OPB45598 | T4742_S00010.509 | NmrA family protein |
| OPB46183 | T4742_S00008.250 | NmrA family protein |
| OPB46237 | T4742_S00008.304 | NmrA family protein |
| OPB46986 | T4742_S00009.428 | NmrA family protein |
| OPB37538 | T4742_S00005.565 | NmrA family protein |
| OPB40636 | T4742_S00001.716 | NmrA family protein |
| OPB44718 | T4742_S00013.22 | NmrA family protein |
| OPB44773 | T4742_S00013.78 | NmrA family protein |
| OPB44952 | T4742_S00013.257 | NmrA family protein |
| OPB45376 | T4742_S00010.287 | NmrA family protein |
| OPB46394 | T4742_S00008.461 | NmrA family protein |
| OPB44795 | T4742_S00013.100 | NmrA family protein [Trichoderma harzianum] |
| OPB45926 | T4742_S00014.263 | NmrA family protein [Trichoderma harzianum] |
| OPB40558 | T4742_S00001.637 | NmrA-like family domain-containing protein 1 [Penicillium subrubescens] |
| OPB36206 | T4742_S00021.124 | NmrA-like family protein |
| OPB36255 | T4742_S00021.173 | NmrA-like family protein |
| OPB36478 | T4742_S00007.195 | NmrA-like family protein |
| OPB38937 | T4742_S00002.986 | NmrA-like family protein |
| OPB41173 | T4742_S00011.13 | NmrA-like family protein |
| OPB41203 | T4742_S00011.43 | NmrA-like family protein |
| OPB41837 | T4742_S00018.160 | NmrA-like family protein |
| OPB41883 | T4742_S00018.206 | NmrA-like family protein |
| OPB42805 | T4742_S00012.42 | NmrA-like family protein |
| OPB42822 | T4742_S00012.59 | NmrA-like family protein |
| OPB44516 | T4742_S00003.788 | nmrA-like family protein |
| OPB44641 | T4742_S00003.913 | NmrA-like family protein |
| OPB44857 | T4742_S00013.162 | nmrA-like family protein |
| OPB45796 | T4742_S00014.131 | NmrA-like family protein |
| OPB46109 | T4742_S00008.176 | NmrA-like family protein |

| | | |
|---|---|---|
| OPB40549 | T4742_S00001.628 | NmrA-like family protein |
| OPB43919 | T4742_S00003.190 | NmrA-like family protein |
| OPB45798 | T4742_S00014.133 | NmrA-like family protein |
| OPB46386 | T4742_S00008.453 | NmrA-like family protein |
| OPB41287 | T4742_S00011.128 | NmrA-like protein [Macrophomina phaseolina MS6] |
| OPB36790 | T4742_S00007.508 | NMT1 thiamine biosynthesis protein |
| OPB45106 | T4742_S00010.13 | Noc3p, probably involved in nuclear export of pre-ribosomes |
| OPB42903 | T4742_S00012.140 | NOC4, involved in nuclear export of 40S pre-ribosomal subunit to the cytoplasm. |
| OPB42931 | T4742_S00012.168 | nonselective cation channel [Colletotrichum tofieldiae] |
| OPB42930 | T4742_S00012.167 | nonselective cation channel, putative |
| OPB37974 | T4742_S00002.21 | nonsense-mediated mRNA decay factor (Upf2), putative |
| OPB45042 | T4742_S00013.347 | Nop1, component of the SSU processome involved in processing of pre-18S rRNA |
| OPB43027 | T4742_S00012.264 | Nop14p, maturaltion of 18rRNA |
| OPB43934 | T4742_S00003.205 | Not CCR4-Not complex component, N-terminal |
| OPB44254 | T4742_S00003.525 | NOXA, NOX1 |
| OPB36048 | T4742_S00024.9 | NRPS |
| OPB36439 | T4742_S00007.156 | NRPS |
| OPB36804 | T4742_S00007.522 | NRPS |
| OPB37353 | T4742_S00005.380 | NRPS |
| OPB37679 | T4742_S00005.706 | NRPS |
| OPB39084 | T4742_S00006.143 | NRPS |
| OPB39917 | T4742_S00015.261 | NRPS |
| OPB40573 | T4742_S00001.652 | NRPS |
| OPB40613 | T4742_S00001.693 | NRPS |
| OPB40675 | T4742_S00001.755 | NRPS |
| OPB40684 | T4742_S00001.764 | NRPS |
| OPB40688 | T4742_S00001.768 | NRPS |
| OPB40728 | T4742_S00001.808 | NRPS |
| OPB41124 | T4742_S00020.148 | NRPS |
| OPB41327 | T4742_S00011.168 | NRPS |
| OPB41765 | T4742_S00018.88 | NRPS |
| OPB42740 | T4742_S00004.846 | NRPS |
| OPB42762 | T4742_S00004.868 | NRPS |
| OPB43219 | T4742_S00022.22 | NRPS |
| OPB43455 | T4742_S00019.188 | NRPS |
| OPB45171 | T4742_S00010.78 | NRPS |

| | | |
|---|---|---|
| OPB45396 | T4742_S00010.307 | NRPS |
| OPB45505 | T4742_S00010.416 | NRPS |
| OPB46118 | T4742_S00008.185 | NRPS |
| OPB44687 | T4742_S00003.959 | NRPS, nonribosomal peptide synthase [Metarhizium acridum CQMa 102] |
| OPB46518 | T4742_S00008.585 | NRPS; non-ribosomal peptide synthetase [Trichoderma virens Gv29-8] |
| OPB44590 | T4742_S00003.862 | NRPS-like protein [Pseudogymnoascus sp. 05NY08] |
| OPB40575 | T4742_S00001.655 | NRPS-PKS?? |
| OPB39624 | T4742_S00006.684 | NsdC |
| OPB38567 | T4742_S00002.616 | NTPase |
| OPB41554 | T4742_S00011.397 | nuclear cap-binding protein complex, small subunit |
| OPB45689 | T4742_S00014.23 | nuclear exosomal RNA helicase, related to N. crassa FRH |
| OPB46841 | T4742_S00009.283 | nuclear localization sequence binding protein |
| OPB36691 | T4742_S00007.409 | nuclear migration protein NudF |
| OPB44135 | T4742_S00003.406 | nuclear migration protein, ami1, of N. crassa |
| OPB44149 | T4742_S00003.420 | nuclear movement protein |
| OPB46828 | T4742_S00009.270 | nuclear mRNA splicing factor-associated protein, putative |
| OPB40123 | T4742_S00001.202 | Nuclear pore complex component |
| OPB44006 | T4742_S00003.277 | nuclear pore complex protein sonA |
| OPB45276 | T4742_S00010.187 | nuclear pore protein |
| OPB39988 | T4742_S00001.67 | nuclear protein Es2, putative |
| OPB38311 | T4742_S00002.358 | nuclear segregation protein (Bfr1), putative |
| OPB36528 | T4742_S00007.245 | Nuclear transport factor 2 |
| OPB43101 | T4742_S00012.338 | Nuclear transport factor 2 |
| OPB37247 | T4742_S00005.274 | nuclear transport factor 2 domain-containing protein |
| OPB37013 | T4742_S00005.40 | Nuclear transport regulator Npl4 |
| OPB36194 | T4742_S00021.112 | nuclease S1 precursor |
| OPB43081 | T4742_S00012.318 | nuclease, mitochondrial |
| OPB45776 | T4742_S00014.111 | nuclease, Subunit of MRX complex , Repair of DNA double-strand breaks and in telomere stability |
| OPB36317 | T4742_S00007.34 | Nucleic acid binding, OB-fold, tRNA/helicase-type |
| OPB44690 | T4742_S00003.962 | Nucleolar GTPase/ATPase p130 |
| OPB38438 | T4742_S00002.486 | nucleolar GTP-binding protein 1 |
| OPB39210 | T4742_S00006.270 | nucleolar protein 12 |
| OPB46164 | T4742_S00008.231 | nucleolar RNA binding protein NifK |
| OPB46078 | T4742_S00008.145 | nucleolysin TIA-1 |
| OPB38736 | T4742_S00002.785 | nucleoside deaminase |
| OPB39908 | T4742_S00015.252 | Nucleoside diphosphatase involved in protein glycosylation in the Golgi |

| | | |
|---|---|---|
| OPB37285 | T4742_S00005.312 | Nucleoside diphosphate kinase |
| OPB46670 | T4742_S00009.112 | nucleoside phosphorylase |
| OPB42331 | T4742_S00004.433 | nucleoside transporter |
| OPB46512 | T4742_S00008.579 | nucleoside-diphosphate-sugar epimerase |
| OPB44373 | T4742_S00003.644 | nucleoside-diphosphate-sugar epimerase, putative (check!) |
| OPB45088 | T4742_S00013.393 | Nucleosome assembly protein NAP-1 |
| OPB38285 | T4742_S00002.332 | nucleotide binding protein Nbp35, putative |
| OPB39757 | T4742_S00015.101 | nucleotide deaminase TAD3 |
| OPB43722 | T4742_S00016.242 | Nucleotide exchange factor Sil1 |
| OPB39166 | T4742_S00006.226 | nucleotide sugar dehydrogenase (UDP-Glc?) |
| OPB46205 | T4742_S00008.272 | Nucleotidylyl transferase, partial [Trichoderma reesei RUT C-30] |
| OPB44170 | T4742_S00003.441 | nudix domain containing protein |
| OPB36122 | T4742_S00021.39 | NUDIX family hydrolase |
| OPB37790 | T4742_S00017.57 | NUDIX family hydrolase |
| OPB41938 | T4742_S00004.40 | NUDIX family hydrolase |
| OPB46060 | T4742_S00008.127 | NUDIX family hydrolase |
| OPB46723 | T4742_S00009.165 | NUDIX family hydrolase |
| OPB43558 | T4742_S00016.78 | O6-methylguanine alkyltransferase |
| OPB39729 | T4742_S00015.73 | O-acetyl-L-homoserine sulfhydrylase, related to S. cerevisiae Met17p |
| OPB37951 | T4742_S00017.218 | O-acetyltransferase |
| OPB42680 | T4742_S00004.786 | O-acetyltransferase |
| OPB41196 | T4742_S00011.36 | O-acetyltransferase Trichothecene biosynthesis |
| OPB41195 | T4742_S00011.35 | octopine/nopaline dehydrogenase |
| OPB46028 | T4742_S00008.94 | oleate δ-12 desaturase |
| OPB43378 | T4742_S00019.111 | oleate-delta12-desaturase |
| OPB36240 | T4742_S00021.158 | oligopeptide transporter |
| OPB38276 | T4742_S00002.323 | oligopeptide transporter |
| OPB40786 | T4742_S00001.867 | oligopeptide transporter |
| OPB41957 | T4742_S00004.59 | oligopeptide transporter |
| OPB41958 | T4742_S00004.60 | oligopeptide transporter |
| OPB44078 | T4742_S00003.349 | oligopeptide transporter |
| OPB45599 | T4742_S00010.510 | oligopeptide transporter MTD1 |
| OPB38488 | T4742_S00002.537 | oligopeptide transporter, OPT family |
| OPB38496 | T4742_S00002.545 | oligopeptide transporter, OPT family |
| OPB39397 | T4742_S00006.457 | oligopeptide transporter, OPT family |
| OPB40340 | T4742_S00001.419 | oligopeptide transporter, OPT family |

| | | |
|---|---|---|
| OPB41162 | T4742_S00011.2 | oligopeptide transporter, OPT family |
| OPB41324 | T4742_S00011.165 | oligopeptide transporter, OPT family |
| OPB46425 | T4742_S00008.492 | oligopeptide transporter, OPT family |
| OPB42147 | T4742_S00004.249 | Oligosaccharyltransferase, alpha subunit |
| OPB41347 | T4742_S00011.188 | Oligosaccharyltransferase, beta subunit WBP1 |
| OPB42971 | T4742_S00012.208 | Oligosaccharyltransferase, epsilon subunit |
| OPB36028 | T4742_S00023.63 | Oligosaccharyltransferase, gamma subunit |
| OPB37024 | T4742_S00005.51 | o-methyltransferase |
| OPB40530 | T4742_S00001.609 | o-methyltransferase |
| OPB40672 | T4742_S00001.752 | o-methyltransferase |
| OPB40862 | T4742_S00001.943 | o-methyltransferase |
| OPB41373 | T4742_S00011.214 | o-methyltransferase |
| OPB41705 | T4742_S00018.28 | o-methyltransferase |
| OPB41869 | T4742_S00018.192 | o-methyltransferase |
| OPB42834 | T4742_S00012.71 | o-methyltransferase |
| OPB43352 | T4742_S00019.85 | o-methyltransferase |
| OPB44019 | T4742_S00003.290 | o-methyltransferase |
| OPB45886 | T4742_S00014.221 | o-methyltransferase |
| OPB46033 | T4742_S00008.99 | o-methyltransferase |
| OPB35926 | T4742_S00029.1 | origin recognition complex subunit Orc5, putative |
| OPB37399 | T4742_S00005.426 | origin recognition complex, subunit 2 |
| OPB45145 | T4742_S00010.52 | ornithine aminotransferase |
| OPB46416 | T4742_S00008.483 | ornithine aminotransferase |
| OPB42483 | T4742_S00004.585 | Ornithine carbamoyltransferase OTC/ARG3 |
| OPB37240 | T4742_S00005.267 | ornithine decarboxylase |
| OPB38437 | T4742_S00002.485 | Orotate phosphoribosyl transferase |
| OPB42385 | T4742_S00004.487 | Orotidine 5'-phosphate decarboxylase |
| OPB35922 | T4742_S00027.3 | orphan protein |
| OPB35923 | T4742_S00027.4 | orphan protein |
| OPB35924 | T4742_S00027.5 | orphan protein |
| OPB35969 | T4742_S00023.4 | orphan protein |
| OPB35970 | T4742_S00023.5 | orphan protein |
| OPB35985 | T4742_S00023.20 | orphan protein |
| OPB36029 | T4742_S00023.64 | orphan protein |
| OPB36033 | T4742_S00023.68 | orphan protein |
| OPB36035 | T4742_S00023.70 | orphan protein |

| | | |
|---|---|---|
| OPB36045 | T4742_S00024.6 | orphan protein |
| OPB36053 | T4742_S00024.14 | orphan protein |
| OPB36060 | T4742_S00024.21 | orphan protein |
| OPB36061 | T4742_S00024.22 | orphan protein |
| OPB36071 | T4742_S00024.32 | orphan protein |
| OPB36075 | T4742_S00024.36 | orphan protein |
| OPB36095 | T4742_S00021.11 | orphan protein |
| OPB36097 | T4742_S00021.13 | orphan protein |
| OPB36098 | T4742_S00021.14 | orphan protein |
| OPB36101 | T4742_S00021.17 | orphan protein |
| OPB36102 | T4742_S00021.18 | orphan protein |
| OPB36107 | T4742_S00021.23 | orphan protein |
| OPB36110 | T4742_S00021.26 | orphan protein |
| OPB36114 | T4742_S00021.30 | orphan protein |
| OPB36129 | T4742_S00021.46 | orphan protein |
| OPB36134 | T4742_S00021.51 | orphan protein |
| OPB36135 | T4742_S00021.52 | orphan protein |
| OPB36140 | T4742_S00021.57 | orphan protein |
| OPB36146 | T4742_S00021.63 | orphan protein |
| OPB36147 | T4742_S00021.64 | orphan protein |
| OPB36151 | T4742_S00021.68 | orphan protein |
| OPB36165 | T4742_S00021.82 | orphan protein |
| OPB36170 | T4742_S00021.87 | orphan protein |
| OPB36177 | T4742_S00021.95 | orphan protein |
| OPB36196 | T4742_S00021.114 | orphan protein |
| OPB36197 | T4742_S00021.115 | orphan protein |
| OPB36202 | T4742_S00021.120 | orphan protein |
| OPB36207 | T4742_S00021.125 | orphan protein |
| OPB36208 | T4742_S00021.126 | orphan protein |
| OPB36209 | T4742_S00021.127 | orphan protein |
| OPB36211 | T4742_S00021.129 | orphan protein |
| OPB36216 | T4742_S00021.134 | orphan protein |
| OPB36221 | T4742_S00021.139 | orphan protein |
| OPB36232 | T4742_S00021.150 | orphan protein |
| OPB36239 | T4742_S00021.157 | orphan protein |
| OPB36241 | T4742_S00021.159 | orphan protein |

| | | |
|---|---|---|
| OPB36243 | T4742_S00021.161 | orphan protein |
| OPB36244 | T4742_S00021.162 | orphan protein |
| OPB36245 | T4742_S00021.163 | orphan protein |
| OPB36248 | T4742_S00021.166 | orphan protein |
| OPB36253 | T4742_S00021.171 | orphan protein |
| OPB36262 | T4742_S00021.181 | orphan protein |
| OPB36266 | T4742_S00021.185 | orphan protein |
| OPB36283 | T4742_S00021.202 | orphan protein |
| OPB36286 | T4742_S00007.3 | orphan protein |
| OPB36300 | T4742_S00007.17 | orphan protein |
| OPB36404 | T4742_S00007.121 | orphan protein |
| OPB36420 | T4742_S00007.137 | orphan protein |
| OPB36424 | T4742_S00007.141 | orphan protein |
| OPB36454 | T4742_S00007.171 | orphan protein |
| OPB36455 | T4742_S00007.172 | orphan protein |
| OPB36459 | T4742_S00007.176 | orphan protein |
| OPB36466 | T4742_S00007.183 | orphan protein |
| OPB36565 | T4742_S00007.282 | orphan protein |
| OPB36577 | T4742_S00007.294 | orphan protein |
| OPB36590 | T4742_S00007.307 | orphan protein |
| OPB36608 | T4742_S00007.326 | orphan protein |
| OPB36609 | T4742_S00007.327 | orphan protein |
| OPB36630 | T4742_S00007.348 | orphan protein |
| OPB36636 | T4742_S00007.354 | orphan protein |
| OPB36762 | T4742_S00007.480 | orphan protein |
| OPB36785 | T4742_S00007.503 | orphan protein |
| OPB36802 | T4742_S00007.520 | orphan protein |
| OPB36817 | T4742_S00007.535 | orphan protein |
| OPB36834 | T4742_S00007.552 | orphan protein |
| OPB36841 | T4742_S00007.559 | orphan protein |
| OPB36844 | T4742_S00007.562 | orphan protein |
| OPB36869 | T4742_S00007.587 | orphan protein |
| OPB36872 | T4742_S00007.590 | orphan protein |
| OPB36917 | T4742_S00007.635 | orphan protein |
| OPB36989 | T4742_S00005.16 | orphan protein |
| OPB36991 | T4742_S00005.18 | orphan protein |

| | | |
|---|---|---|
| OPB37014 | T4742_S00005.41 | orphan protein |
| OPB37018 | T4742_S00005.45 | orphan protein |
| OPB37028 | T4742_S00005.55 | orphan protein |
| OPB37048 | T4742_S00005.75 | orphan protein |
| OPB37110 | T4742_S00005.137 | orphan protein |
| OPB37142 | T4742_S00005.169 | orphan protein |
| OPB37150 | T4742_S00005.177 | orphan protein |
| OPB37171 | T4742_S00005.198 | orphan protein |
| OPB37209 | T4742_S00005.236 | orphan protein |
| OPB37250 | T4742_S00005.277 | orphan protein |
| OPB37272 | T4742_S00005.299 | orphan protein |
| OPB37317 | T4742_S00005.344 | orphan protein |
| OPB37374 | T4742_S00005.401 | orphan protein |
| OPB37376 | T4742_S00005.403 | orphan protein |
| OPB37397 | T4742_S00005.424 | orphan protein |
| OPB37419 | T4742_S00005.446 | orphan protein |
| OPB37431 | T4742_S00005.458 | orphan protein |
| OPB37469 | T4742_S00005.496 | orphan protein |
| OPB37543 | T4742_S00005.570 | orphan protein |
| OPB37572 | T4742_S00005.599 | orphan protein |
| OPB37574 | T4742_S00005.601 | orphan protein |
| OPB37575 | T4742_S00005.602 | orphan protein |
| OPB37583 | T4742_S00005.610 | orphan protein |
| OPB37592 | T4742_S00005.619 | orphan protein |
| OPB37594 | T4742_S00005.621 | orphan protein |
| OPB37619 | T4742_S00005.646 | orphan protein |
| OPB37620 | T4742_S00005.647 | orphan protein |
| OPB37629 | T4742_S00005.656 | orphan protein |
| OPB37653 | T4742_S00005.680 | orphan protein |
| OPB37694 | T4742_S00005.721 | orphan protein |
| OPB37717 | T4742_S00005.745 | orphan protein |
| OPB37733 | T4742_S00005.761 | orphan protein |
| OPB37752 | T4742_S00017.19 | orphan protein |
| OPB37760 | T4742_S00017.27 | orphan protein |
| OPB37763 | T4742_S00017.30 | orphan protein |
| OPB37778 | T4742_S00017.45 | orphan protein |

| | | |
|---|---|---|
| OPB37802 | T4742_S00017.69 | orphan protein |
| OPB37803 | T4742_S00017.70 | orphan protein |
| OPB37804 | T4742_S00017.71 | orphan protein |
| OPB37822 | T4742_S00017.89 | orphan protein |
| OPB37850 | T4742_S00017.117 | orphan protein |
| OPB37906 | T4742_S00017.173 | orphan protein |
| OPB37912 | T4742_S00017.179 | orphan protein |
| OPB37925 | T4742_S00017.192 | orphan protein |
| OPB37929 | T4742_S00017.196 | orphan protein |
| OPB37930 | T4742_S00017.197 | orphan protein |
| OPB37933 | T4742_S00017.200 | orphan protein |
| OPB37942 | T4742_S00017.209 | orphan protein |
| OPB38003 | T4742_S00002.50 | orphan protein |
| OPB38024 | T4742_S00002.71 | orphan protein |
| OPB38079 | T4742_S00002.126 | orphan protein |
| OPB38083 | T4742_S00002.130 | orphan protein |
| OPB38092 | T4742_S00002.139 | orphan protein |
| OPB38113 | T4742_S00002.160 | orphan protein |
| OPB38120 | T4742_S00002.167 | orphan protein |
| OPB38123 | T4742_S00002.170 | orphan protein |
| OPB38131 | T4742_S00002.178 | orphan protein |
| OPB38136 | T4742_S00002.183 | orphan protein |
| OPB38140 | T4742_S00002.187 | orphan protein |
| OPB38160 | T4742_S00002.207 | orphan protein |
| OPB38163 | T4742_S00002.210 | orphan protein |
| OPB38182 | T4742_S00002.229 | orphan protein |
| OPB38190 | T4742_S00002.237 | orphan protein |
| OPB38228 | T4742_S00002.275 | orphan protein |
| OPB38240 | T4742_S00002.287 | orphan protein |
| OPB38241 | T4742_S00002.288 | orphan protein |
| OPB38248 | T4742_S00002.295 | orphan protein |
| OPB38252 | T4742_S00002.299 | orphan protein |
| OPB38269 | T4742_S00002.316 | orphan protein |
| OPB38308 | T4742_S00002.355 | orphan protein |
| OPB38363 | T4742_S00002.411 | orphan protein |
| OPB38377 | T4742_S00002.425 | orphan protein |

| | | |
|---|---|---|
| OPB38384 | T4742_S00002.432 | orphan protein |
| OPB38400 | T4742_S00002.448 | orphan protein |
| OPB38433 | T4742_S00002.481 | orphan protein |
| OPB38465 | T4742_S00002.513 | orphan protein |
| OPB38472 | T4742_S00002.520 | orphan protein |
| OPB38474 | T4742_S00002.522 | orphan protein |
| OPB38511 | T4742_S00002.560 | orphan protein |
| OPB38524 | T4742_S00002.573 | orphan protein |
| OPB38533 | T4742_S00002.582 | orphan protein |
| OPB38544 | T4742_S00002.593 | orphan protein |
| OPB38611 | T4742_S00002.660 | orphan protein |
| OPB38620 | T4742_S00002.669 | orphan protein |
| OPB38640 | T4742_S00002.689 | orphan protein |
| OPB38648 | T4742_S00002.697 | orphan protein |
| OPB38658 | T4742_S00002.707 | orphan protein |
| OPB38663 | T4742_S00002.712 | orphan protein |
| OPB38680 | T4742_S00002.729 | orphan protein |
| OPB38712 | T4742_S00002.761 | orphan protein |
| OPB38725 | T4742_S00002.774 | orphan protein |
| OPB38735 | T4742_S00002.784 | orphan protein |
| OPB38825 | T4742_S00002.874 | orphan protein |
| OPB38829 | T4742_S00002.878 | orphan protein |
| OPB38850 | T4742_S00002.899 | orphan protein |
| OPB38851 | T4742_S00002.900 | orphan protein |
| OPB38865 | T4742_S00002.914 | orphan protein |
| OPB38866 | T4742_S00002.915 | orphan protein |
| OPB38885 | T4742_S00002.934 | orphan protein |
| OPB38888 | T4742_S00002.937 | orphan protein |
| OPB38890 | T4742_S00002.939 | orphan protein |
| OPB38911 | T4742_S00002.960 | orphan protein |
| OPB38918 | T4742_S00002.967 | orphan protein |
| OPB38925 | T4742_S00002.974 | orphan protein |
| OPB38926 | T4742_S00002.975 | orphan protein |
| OPB38929 | T4742_S00002.978 | orphan protein |
| OPB38935 | T4742_S00002.984 | orphan protein |
| OPB38963 | T4742_S00006.22 | orphan protein |

| | | |
|---|---|---|
| OPB39004 | T4742_S00006.63 | orphan protein |
| OPB39039 | T4742_S00006.98 | orphan protein |
| OPB39072 | T4742_S00006.131 | orphan protein |
| OPB39113 | T4742_S00006.173 | orphan protein |
| OPB39145 | T4742_S00006.205 | orphan protein |
| OPB39147 | T4742_S00006.207 | orphan protein |
| OPB39164 | T4742_S00006.224 | orphan protein |
| OPB39180 | T4742_S00006.240 | orphan protein |
| OPB39186 | T4742_S00006.246 | orphan protein |
| OPB39189 | T4742_S00006.249 | orphan protein |
| OPB39215 | T4742_S00006.275 | orphan protein |
| OPB39224 | T4742_S00006.284 | orphan protein |
| OPB39233 | T4742_S00006.293 | orphan protein |
| OPB39296 | T4742_S00006.356 | orphan protein |
| OPB39301 | T4742_S00006.361 | orphan protein |
| OPB39314 | T4742_S00006.374 | orphan protein |
| OPB39340 | T4742_S00006.400 | orphan protein |
| OPB39363 | T4742_S00006.423 | orphan protein |
| OPB39486 | T4742_S00006.546 | orphan protein |
| OPB39497 | T4742_S00006.557 | orphan protein |
| OPB39529 | T4742_S00006.589 | orphan protein |
| OPB39539 | T4742_S00006.599 | orphan protein |
| OPB39604 | T4742_S00006.664 | orphan protein |
| OPB39651 | T4742_S00006.711 | orphan protein |
| OPB39659 | T4742_S00015.3 | orphan protein |
| OPB39661 | T4742_S00015.5 | orphan protein |
| OPB39674 | T4742_S00015.18 | orphan protein |
| OPB39675 | T4742_S00015.19 | orphan protein |
| OPB39676 | T4742_S00015.20 | orphan protein |
| OPB39678 | T4742_S00015.22 | orphan protein |
| OPB39744 | T4742_S00015.88 | orphan protein |
| OPB39771 | T4742_S00015.115 | orphan protein |
| OPB39784 | T4742_S00015.128 | orphan protein |
| OPB39787 | T4742_S00015.131 | orphan protein |
| OPB39789 | T4742_S00015.133 | orphan protein |
| OPB39793 | T4742_S00015.137 | orphan protein |

| | | |
|---|---|---|
| OPB39794 | T4742_S00015.138 | orphan protein |
| OPB39795 | T4742_S00015.139 | orphan protein |
| OPB39816 | T4742_S00015.160 | orphan protein |
| OPB39821 | T4742_S00015.165 | orphan protein |
| OPB39834 | T4742_S00015.178 | orphan protein |
| OPB39842 | T4742_S00015.186 | orphan protein |
| OPB39854 | T4742_S00015.198 | orphan protein |
| OPB39936 | T4742_S00001.15 | orphan protein |
| OPB39962 | T4742_S00001.41 | orphan protein |
| OPB40033 | T4742_S00001.112 | orphan protein |
| OPB40047 | T4742_S00001.126 | orphan protein |
| OPB40073 | T4742_S00001.152 | orphan protein |
| OPB40115 | T4742_S00001.194 | orphan protein |
| OPB40170 | T4742_S00001.249 | orphan protein |
| OPB40214 | T4742_S00001.293 | orphan protein |
| OPB40217 | T4742_S00001.296 | orphan protein |
| OPB40219 | T4742_S00001.298 | orphan protein |
| OPB40222 | T4742_S00001.301 | orphan protein |
| OPB40239 | T4742_S00001.318 | orphan protein |
| OPB40263 | T4742_S00001.342 | orphan protein |
| OPB40276 | T4742_S00001.355 | orphan protein |
| OPB40281 | T4742_S00001.360 | orphan protein |
| OPB40321 | T4742_S00001.400 | orphan protein |
| OPB40323 | T4742_S00001.402 | orphan protein |
| OPB40346 | T4742_S00001.425 | orphan protein |
| OPB40348 | T4742_S00001.427 | orphan protein |
| OPB40351 | T4742_S00001.430 | orphan protein |
| OPB40391 | T4742_S00001.470 | orphan protein |
| OPB40406 | T4742_S00001.485 | orphan protein |
| OPB40451 | T4742_S00001.530 | orphan protein |
| OPB40515 | T4742_S00001.594 | orphan protein |
| OPB40517 | T4742_S00001.596 | orphan protein |
| OPB40518 | T4742_S00001.597 | orphan protein |
| OPB40537 | T4742_S00001.616 | orphan protein |
| OPB40548 | T4742_S00001.627 | orphan protein |
| OPB40561 | T4742_S00001.640 | orphan protein |

| | | |
|---|---|---|
| OPB40580 | T4742_S00001.660 | orphan protein |
| OPB40595 | T4742_S00001.675 | orphan protein |
| OPB40597 | T4742_S00001.677 | orphan protein |
| OPB40616 | T4742_S00001.696 | orphan protein |
| OPB40622 | T4742_S00001.702 | orphan protein |
| OPB40626 | T4742_S00001.706 | orphan protein |
| OPB40628 | T4742_S00001.708 | orphan protein |
| OPB40633 | T4742_S00001.713 | orphan protein |
| OPB40634 | T4742_S00001.714 | orphan protein |
| OPB40647 | T4742_S00001.727 | orphan protein |
| OPB40681 | T4742_S00001.761 | orphan protein |
| OPB40693 | T4742_S00001.773 | orphan protein |
| OPB40721 | T4742_S00001.801 | orphan protein |
| OPB40732 | T4742_S00001.812 | orphan protein |
| OPB40733 | T4742_S00001.813 | orphan protein |
| OPB40736 | T4742_S00001.816 | orphan protein |
| OPB40741 | T4742_S00001.822 | orphan protein |
| OPB40782 | T4742_S00001.863 | orphan protein |
| OPB40800 | T4742_S00001.881 | orphan protein |
| OPB40813 | T4742_S00001.894 | orphan protein |
| OPB40830 | T4742_S00001.911 | orphan protein |
| OPB40834 | T4742_S00001.915 | orphan protein |
| OPB40879 | T4742_S00001.960 | orphan protein |
| OPB40881 | T4742_S00001.962 | orphan protein |
| OPB40904 | T4742_S00001.985 | orphan protein |
| OPB40905 | T4742_S00001.986 | orphan protein |
| OPB40910 | T4742_S00001.991 | orphan protein |
| OPB40923 | T4742_S00001.1004 | orphan protein |
| OPB40925 | T4742_S00001.1006 | orphan protein |
| OPB40929 | T4742_S00001.1010 | orphan protein |
| OPB40937 | T4742_S00001.1018 | orphan protein |
| OPB40938 | T4742_S00001.1019 | orphan protein |
| OPB40939 | T4742_S00001.1020 | orphan protein |
| OPB40940 | T4742_S00001.1021 | orphan protein |
| OPB40959 | T4742_S00001.1040 | orphan protein |
| OPB40970 | T4742_S00001.1051 | orphan protein |

| | | |
|---|---|---|
| OPB40979 | T4742_S00020.3 | orphan protein |
| OPB40995 | T4742_S00020.19 | orphan protein |
| OPB41001 | T4742_S00020.25 | orphan protein |
| OPB41004 | T4742_S00020.28 | orphan protein |
| OPB41023 | T4742_S00020.47 | orphan protein |
| OPB41075 | T4742_S00020.99 | orphan protein |
| OPB41076 | T4742_S00020.100 | orphan protein |
| OPB41086 | T4742_S00020.110 | orphan protein |
| OPB41103 | T4742_S00020.127 | orphan protein |
| OPB41110 | T4742_S00020.134 | orphan protein |
| OPB41113 | T4742_S00020.137 | orphan protein |
| OPB41120 | T4742_S00020.144 | orphan protein |
| OPB41136 | T4742_S00020.160 | orphan protein |
| OPB41147 | T4742_S00020.171 | orphan protein |
| OPB41156 | T4742_S00020.180 | orphan protein |
| OPB41166 | T4742_S00011.6 | orphan protein |
| OPB41181 | T4742_S00011.21 | orphan protein |
| OPB41204 | T4742_S00011.44 | orphan protein |
| OPB41230 | T4742_S00011.71 | orphan protein |
| OPB41279 | T4742_S00011.120 | orphan protein |
| OPB41294 | T4742_S00011.135 | orphan protein |
| OPB41295 | T4742_S00011.136 | orphan protein |
| OPB41306 | T4742_S00011.147 | orphan protein |
| OPB41322 | T4742_S00011.163 | orphan protein |
| OPB41332 | T4742_S00011.173 | orphan protein |
| OPB41333 | T4742_S00011.174 | orphan protein |
| OPB41336 | T4742_S00011.177 | orphan protein |
| OPB41352 | T4742_S00011.193 | orphan protein |
| OPB41357 | T4742_S00011.198 | orphan protein |
| OPB41369 | T4742_S00011.210 | orphan protein |
| OPB41374 | T4742_S00011.215 | orphan protein |
| OPB41383 | T4742_S00011.224 | orphan protein |
| OPB41405 | T4742_S00011.247 | orphan protein |
| OPB41445 | T4742_S00011.288 | orphan protein |
| OPB41450 | T4742_S00011.293 | orphan protein |
| OPB41451 | T4742_S00011.294 | orphan protein |

| | | |
|---|---|---|
| OPB41471 | T4742_S00011.314 | orphan protein |
| OPB41473 | T4742_S00011.316 | orphan protein |
| OPB41475 | T4742_S00011.318 | orphan protein |
| OPB41527 | T4742_S00011.370 | orphan protein |
| OPB41536 | T4742_S00011.379 | orphan protein |
| OPB41545 | T4742_S00011.388 | orphan protein |
| OPB41546 | T4742_S00011.389 | orphan protein |
| OPB41568 | T4742_S00011.411 | orphan protein |
| OPB41603 | T4742_S00011.446 | orphan protein |
| OPB41612 | T4742_S00011.455 | orphan protein |
| OPB41638 | T4742_S00011.481 | orphan protein |
| OPB41663 | T4742_S00011.506 | orphan protein |
| OPB41680 | T4742_S00018.2 | orphan protein |
| OPB41707 | T4742_S00018.30 | orphan protein |
| OPB41711 | T4742_S00018.34 | orphan protein |
| OPB41713 | T4742_S00018.36 | orphan protein |
| OPB41721 | T4742_S00018.44 | orphan protein |
| OPB41723 | T4742_S00018.46 | orphan protein |
| OPB41724 | T4742_S00018.47 | orphan protein |
| OPB41726 | T4742_S00018.49 | orphan protein |
| OPB41727 | T4742_S00018.50 | orphan protein |
| OPB41728 | T4742_S00018.51 | orphan protein |
| OPB41738 | T4742_S00018.61 | orphan protein |
| OPB41741 | T4742_S00018.64 | orphan protein |
| OPB41752 | T4742_S00018.75 | orphan protein |
| OPB41764 | T4742_S00018.87 | orphan protein |
| OPB41772 | T4742_S00018.95 | orphan protein |
| OPB41777 | T4742_S00018.100 | orphan protein |
| OPB41807 | T4742_S00018.130 | orphan protein |
| OPB41819 | T4742_S00018.142 | orphan protein |
| OPB41870 | T4742_S00018.193 | orphan protein |
| OPB41885 | T4742_S00018.208 | orphan protein |
| OPB41887 | T4742_S00018.210 | orphan protein |
| OPB41888 | T4742_S00018.211 | orphan protein |
| OPB41889 | T4742_S00018.212 | orphan protein |
| OPB41894 | T4742_S00018.217 | orphan protein |

| | | |
|---|---|---|
| OPB41939 | T4742_S00004.41 | orphan protein |
| OPB41956 | T4742_S00004.58 | orphan protein |
| OPB42016 | T4742_S00004.118 | orphan protein |
| OPB42036 | T4742_S00004.138 | orphan protein |
| OPB42042 | T4742_S00004.144 | orphan protein |
| OPB42084 | T4742_S00004.186 | orphan protein |
| OPB42089 | T4742_S00004.191 | orphan protein |
| OPB42114 | T4742_S00004.216 | orphan protein |
| OPB42116 | T4742_S00004.218 | orphan protein |
| OPB42153 | T4742_S00004.255 | orphan protein |
| OPB42168 | T4742_S00004.270 | orphan protein |
| OPB42275 | T4742_S00004.377 | orphan protein |
| OPB42285 | T4742_S00004.387 | orphan protein |
| OPB42323 | T4742_S00004.425 | orphan protein |
| OPB42367 | T4742_S00004.469 | orphan protein |
| OPB42369 | T4742_S00004.471 | orphan protein |
| OPB42390 | T4742_S00004.492 | orphan protein |
| OPB42417 | T4742_S00004.519 | orphan protein |
| OPB42418 | T4742_S00004.520 | orphan protein |
| OPB42463 | T4742_S00004.565 | orphan protein |
| OPB42469 | T4742_S00004.571 | orphan protein |
| OPB42490 | T4742_S00004.592 | orphan protein |
| OPB42497 | T4742_S00004.599 | orphan protein |
| OPB42500 | T4742_S00004.602 | orphan protein |
| OPB42516 | T4742_S00004.618 | orphan protein |
| OPB42528 | T4742_S00004.630 | orphan protein |
| OPB42540 | T4742_S00004.643 | orphan protein |
| OPB42561 | T4742_S00004.664 | orphan protein |
| OPB42565 | T4742_S00004.668 | orphan protein |
| OPB42577 | T4742_S00004.680 | orphan protein |
| OPB42579 | T4742_S00004.682 | orphan protein |
| OPB42583 | T4742_S00004.686 | orphan protein |
| OPB42585 | T4742_S00004.688 | orphan protein |
| OPB42590 | T4742_S00004.694 | orphan protein |
| OPB42606 | T4742_S00004.710 | orphan protein |
| OPB42617 | T4742_S00004.721 | orphan protein |

| | | |
|---|---|---|
| OPB42624 | T4742_S00004.729 | orphan protein |
| OPB42633 | T4742_S00004.738 | orphan protein |
| OPB42645 | T4742_S00004.750 | orphan protein |
| OPB42647 | T4742_S00004.752 | orphan protein |
| OPB42671 | T4742_S00004.776 | orphan protein |
| OPB42679 | T4742_S00004.784 | orphan protein |
| OPB42697 | T4742_S00004.803 | orphan protein |
| OPB42714 | T4742_S00004.820 | orphan protein |
| OPB42755 | T4742_S00004.861 | orphan protein |
| OPB42764 | T4742_S00012.1 | orphan protein |
| OPB42765 | T4742_S00012.2 | orphan protein |
| OPB42777 | T4742_S00012.14 | orphan protein |
| OPB42781 | T4742_S00012.18 | orphan protein |
| OPB42791 | T4742_S00012.28 | orphan protein |
| OPB42797 | T4742_S00012.34 | orphan protein |
| OPB42820 | T4742_S00012.57 | orphan protein |
| OPB42826 | T4742_S00012.63 | orphan protein |
| OPB42833 | T4742_S00012.70 | orphan protein |
| OPB42838 | T4742_S00012.75 | orphan protein |
| OPB42848 | T4742_S00012.85 | orphan protein |
| OPB42896 | T4742_S00012.133 | orphan protein |
| OPB42897 | T4742_S00012.134 | orphan protein |
| OPB42962 | T4742_S00012.199 | orphan protein |
| OPB43007 | T4742_S00012.244 | orphan protein |
| OPB43029 | T4742_S00012.266 | orphan protein |
| OPB43055 | T4742_S00012.292 | orphan protein |
| OPB43068 | T4742_S00012.305 | orphan protein |
| OPB43088 | T4742_S00012.325 | orphan protein |
| OPB43119 | T4742_S00012.356 | orphan protein |
| OPB43134 | T4742_S00012.371 | orphan protein |
| OPB43140 | T4742_S00012.377 | orphan protein |
| OPB43201 | T4742_S00022.4 | orphan protein |
| OPB43230 | T4742_S00022.33 | orphan protein |
| OPB43246 | T4742_S00022.49 | orphan protein |
| OPB43267 | T4742_S00022.71 | orphan protein |
| OPB43286 | T4742_S00019.19 | orphan protein |

| | | |
|---|---|---|
| OPB43291 | T4742_S00019.24 | orphan protein |
| OPB43306 | T4742_S00019.39 | orphan protein |
| OPB43308 | T4742_S00019.41 | orphan protein |
| OPB43338 | T4742_S00019.71 | orphan protein |
| OPB43360 | T4742_S00019.93 | orphan protein |
| OPB43372 | T4742_S00019.105 | orphan protein |
| OPB43373 | T4742_S00019.106 | orphan protein |
| OPB43390 | T4742_S00019.123 | orphan protein |
| OPB43406 | T4742_S00019.139 | orphan protein |
| OPB43432 | T4742_S00019.165 | orphan protein |
| OPB43441 | T4742_S00019.174 | orphan protein |
| OPB43472 | T4742_S00019.205 | orphan protein |
| OPB43479 | T4742_S00019.213 | orphan protein |
| OPB43533 | T4742_S00016.53 | orphan protein |
| OPB43538 | T4742_S00016.58 | orphan protein |
| OPB43539 | T4742_S00016.59 | orphan protein |
| OPB43542 | T4742_S00016.62 | orphan protein |
| OPB43555 | T4742_S00016.75 | orphan protein |
| OPB43607 | T4742_S00016.127 | orphan protein |
| OPB43627 | T4742_S00016.147 | orphan protein |
| OPB43628 | T4742_S00016.148 | orphan protein |
| OPB43645 | T4742_S00016.165 | orphan protein |
| OPB43677 | T4742_S00016.197 | orphan protein |
| OPB43682 | T4742_S00016.202 | orphan protein |
| OPB43684 | T4742_S00016.204 | orphan protein |
| OPB43689 | T4742_S00016.209 | orphan protein |
| OPB43697 | T4742_S00016.217 | orphan protein |
| OPB43699 | T4742_S00016.219 | orphan protein |
| OPB43724 | T4742_S00016.244 | orphan protein |
| OPB43734 | T4742_S00003.5 | orphan protein |
| OPB43741 | T4742_S00003.12 | orphan protein |
| OPB43754 | T4742_S00003.25 | orphan protein |
| OPB43765 | T4742_S00003.36 | orphan protein |
| OPB43776 | T4742_S00003.47 | orphan protein |
| OPB43800 | T4742_S00003.71 | orphan protein |
| OPB43812 | T4742_S00003.83 | orphan protein |

| | | |
|---|---|---|
| OPB43813 | T4742_S00003.84 | orphan protein |
| OPB43829 | T4742_S00003.100 | orphan protein |
| OPB43857 | T4742_S00003.128 | orphan protein |
| OPB43861 | T4742_S00003.132 | orphan protein |
| OPB43872 | T4742_S00003.143 | orphan protein |
| OPB43899 | T4742_S00003.170 | orphan protein |
| OPB43900 | T4742_S00003.171 | orphan protein |
| OPB43911 | T4742_S00003.182 | orphan protein |
| OPB43913 | T4742_S00003.184 | orphan protein |
| OPB43918 | T4742_S00003.189 | orphan protein |
| OPB43933 | T4742_S00003.204 | orphan protein |
| OPB43970 | T4742_S00003.241 | orphan protein |
| OPB44007 | T4742_S00003.278 | orphan protein |
| OPB44018 | T4742_S00003.289 | orphan protein |
| OPB44022 | T4742_S00003.293 | orphan protein |
| OPB44032 | T4742_S00003.303 | orphan protein |
| OPB44048 | T4742_S00003.319 | orphan protein |
| OPB44050 | T4742_S00003.321 | orphan protein |
| OPB44051 | T4742_S00003.322 | orphan protein |
| OPB44062 | T4742_S00003.333 | orphan protein |
| OPB44068 | T4742_S00003.339 | orphan protein |
| OPB44076 | T4742_S00003.347 | orphan protein |
| OPB44110 | T4742_S00003.381 | orphan protein |
| OPB44111 | T4742_S00003.382 | orphan protein |
| OPB44127 | T4742_S00003.398 | orphan protein |
| OPB44155 | T4742_S00003.426 | orphan protein |
| OPB44163 | T4742_S00003.434 | orphan protein |
| OPB44176 | T4742_S00003.447 | orphan protein |
| OPB44183 | T4742_S00003.454 | orphan protein |
| OPB44187 | T4742_S00003.458 | orphan protein |
| OPB44219 | T4742_S00003.490 | orphan protein |
| OPB44220 | T4742_S00003.491 | orphan protein |
| OPB44253 | T4742_S00003.524 | orphan protein |
| OPB44256 | T4742_S00003.527 | orphan protein |
| OPB44257 | T4742_S00003.528 | orphan protein |
| OPB44270 | T4742_S00003.541 | orphan protein |

| | | |
|---|---|---|
| OPB44283 | T4742_S00003.554 | orphan protein |
| OPB44318 | T4742_S00003.589 | orphan protein |
| OPB44349 | T4742_S00003.620 | orphan protein |
| OPB44353 | T4742_S00003.624 | orphan protein |
| OPB44358 | T4742_S00003.629 | orphan protein |
| OPB44364 | T4742_S00003.635 | orphan protein |
| OPB44365 | T4742_S00003.636 | orphan protein |
| OPB44366 | T4742_S00003.637 | orphan protein |
| OPB44384 | T4742_S00003.655 | orphan protein |
| OPB44391 | T4742_S00003.662 | orphan protein |
| OPB44414 | T4742_S00003.685 | orphan protein |
| OPB44440 | T4742_S00003.712 | orphan protein |
| OPB44441 | T4742_S00003.713 | orphan protein |
| OPB44469 | T4742_S00003.741 | orphan protein |
| OPB44502 | T4742_S00003.774 | orphan protein |
| OPB44537 | T4742_S00003.809 | orphan protein |
| OPB44553 | T4742_S00003.825 | orphan protein |
| OPB44555 | T4742_S00003.827 | orphan protein |
| OPB44588 | T4742_S00003.860 | orphan protein |
| OPB44638 | T4742_S00003.910 | orphan protein |
| OPB44655 | T4742_S00003.927 | orphan protein |
| OPB44669 | T4742_S00003.941 | orphan protein |
| OPB44670 | T4742_S00003.942 | orphan protein |
| OPB44674 | T4742_S00003.946 | orphan protein |
| OPB44736 | T4742_S00013.41 | orphan protein |
| OPB44738 | T4742_S00013.43 | orphan protein |
| OPB44739 | T4742_S00013.44 | orphan protein |
| OPB44747 | T4742_S00013.52 | orphan protein |
| OPB44750 | T4742_S00013.55 | orphan protein |
| OPB44772 | T4742_S00013.77 | orphan protein |
| OPB44821 | T4742_S00013.126 | orphan protein |
| OPB44843 | T4742_S00013.148 | orphan protein |
| OPB44846 | T4742_S00013.151 | orphan protein |
| OPB44854 | T4742_S00013.159 | orphan protein |
| OPB44869 | T4742_S00013.174 | orphan protein |
| OPB44872 | T4742_S00013.177 | orphan protein |

| | | |
|---|---|---|
| OPB44875 | T4742_S00013.180 | orphan protein |
| OPB44883 | T4742_S00013.188 | orphan protein |
| OPB44886 | T4742_S00013.191 | orphan protein |
| OPB44924 | T4742_S00013.229 | orphan protein |
| OPB44928 | T4742_S00013.233 | orphan protein |
| OPB44929 | T4742_S00013.234 | orphan protein |
| OPB44940 | T4742_S00013.245 | orphan protein |
| OPB44941 | T4742_S00013.246 | orphan protein |
| OPB44951 | T4742_S00013.256 | orphan protein |
| OPB44953 | T4742_S00013.258 | orphan protein |
| OPB44957 | T4742_S00013.262 | orphan protein |
| OPB44965 | T4742_S00013.270 | orphan protein |
| OPB44992 | T4742_S00013.297 | orphan protein |
| OPB45012 | T4742_S00013.317 | orphan protein |
| OPB45015 | T4742_S00013.320 | orphan protein |
| OPB45018 | T4742_S00013.323 | orphan protein |
| OPB45022 | T4742_S00013.327 | orphan protein |
| OPB45124 | T4742_S00010.31 | orphan protein |
| OPB45129 | T4742_S00010.36 | orphan protein |
| OPB45132 | T4742_S00010.39 | orphan protein |
| OPB45153 | T4742_S00010.60 | orphan protein |
| OPB45168 | T4742_S00010.75 | orphan protein |
| OPB45169 | T4742_S00010.76 | orphan protein |
| OPB45175 | T4742_S00010.82 | orphan protein |
| OPB45208 | T4742_S00010.116 | orphan protein |
| OPB45261 | T4742_S00010.170 | orphan protein |
| OPB45323 | T4742_S00010.234 | orphan protein |
| OPB45357 | T4742_S00010.268 | orphan protein |
| OPB45368 | T4742_S00010.279 | orphan protein |
| OPB45384 | T4742_S00010.295 | orphan protein |
| OPB45394 | T4742_S00010.305 | orphan protein |
| OPB45398 | T4742_S00010.309 | orphan protein |
| OPB45399 | T4742_S00010.310 | orphan protein |
| OPB45426 | T4742_S00010.337 | orphan protein |
| OPB45436 | T4742_S00010.347 | orphan protein |
| OPB45458 | T4742_S00010.369 | orphan protein |

| | | |
|---|---|---|
| OPB45460 | T4742_S00010.371 | orphan protein |
| OPB45461 | T4742_S00010.372 | orphan protein |
| OPB45462 | T4742_S00010.373 | orphan protein |
| OPB45470 | T4742_S00010.381 | orphan protein |
| OPB45491 | T4742_S00010.402 | orphan protein |
| OPB45517 | T4742_S00010.428 | orphan protein |
| OPB45533 | T4742_S00010.444 | orphan protein |
| OPB45547 | T4742_S00010.458 | orphan protein |
| OPB45548 | T4742_S00010.459 | orphan protein |
| OPB45570 | T4742_S00010.481 | orphan protein |
| OPB45572 | T4742_S00010.483 | orphan protein |
| OPB45575 | T4742_S00010.486 | orphan protein |
| OPB45587 | T4742_S00010.498 | orphan protein |
| OPB45619 | T4742_S00010.530 | orphan protein |
| OPB45620 | T4742_S00010.531 | orphan protein |
| OPB45630 | T4742_S00010.541 | orphan protein |
| OPB45661 | T4742_S00010.572 | orphan protein |
| OPB45662 | T4742_S00010.573 | orphan protein |
| OPB45678 | T4742_S00014.12 | orphan protein |
| OPB45692 | T4742_S00014.26 | orphan protein |
| OPB45703 | T4742_S00014.37 | orphan protein |
| OPB45711 | T4742_S00014.45 | orphan protein |
| OPB45713 | T4742_S00014.47 | orphan protein |
| OPB45721 | T4742_S00014.55 | orphan protein |
| OPB45724 | T4742_S00014.58 | orphan protein |
| OPB45731 | T4742_S00014.66 | orphan protein |
| OPB45781 | T4742_S00014.116 | orphan protein |
| OPB45797 | T4742_S00014.132 | orphan protein |
| OPB45799 | T4742_S00014.134 | orphan protein |
| OPB45800 | T4742_S00014.135 | orphan protein |
| OPB45801 | T4742_S00014.136 | orphan protein |
| OPB45802 | T4742_S00014.137 | orphan protein |
| OPB45809 | T4742_S00014.144 | orphan protein |
| OPB45811 | T4742_S00014.146 | orphan protein |
| OPB45812 | T4742_S00014.147 | orphan protein |
| OPB45813 | T4742_S00014.148 | orphan protein |

| | | |
|---|---|---|
| OPB45830 | T4742_S00014.165 | orphan protein |
| OPB45842 | T4742_S00014.177 | orphan protein |
| OPB45854 | T4742_S00014.189 | orphan protein |
| OPB45895 | T4742_S00014.231 | orphan protein |
| OPB45899 | T4742_S00014.235 | orphan protein |
| OPB45904 | T4742_S00014.240 | orphan protein |
| OPB45921 | T4742_S00014.258 | orphan protein |
| OPB45924 | T4742_S00014.261 | orphan protein |
| OPB45928 | T4742_S00014.265 | orphan protein |
| OPB45936 | T4742_S00008.2 | orphan protein |
| OPB45966 | T4742_S00008.32 | orphan protein |
| OPB45968 | T4742_S00008.34 | orphan protein |
| OPB45971 | T4742_S00008.37 | orphan protein |
| OPB45975 | T4742_S00008.41 | orphan protein |
| OPB45983 | T4742_S00008.49 | orphan protein |
| OPB46035 | T4742_S00008.101 | orphan protein |
| OPB46041 | T4742_S00008.107 | orphan protein |
| OPB46048 | T4742_S00008.114 | orphan protein |
| OPB46053 | T4742_S00008.119 | orphan protein |
| OPB46101 | T4742_S00008.168 | orphan protein |
| OPB46137 | T4742_S00008.204 | orphan protein |
| OPB46157 | T4742_S00008.224 | orphan protein |
| OPB46168 | T4742_S00008.235 | orphan protein |
| OPB46204 | T4742_S00008.271 | orphan protein |
| OPB46206 | T4742_S00008.273 | orphan protein |
| OPB46308 | T4742_S00008.375 | orphan protein |
| OPB46309 | T4742_S00008.376 | orphan protein |
| OPB46310 | T4742_S00008.377 | orphan protein |
| OPB46312 | T4742_S00008.379 | orphan protein |
| OPB46315 | T4742_S00008.382 | orphan protein |
| OPB46320 | T4742_S00008.387 | orphan protein |
| OPB46322 | T4742_S00008.389 | orphan protein |
| OPB46344 | T4742_S00008.411 | orphan protein |
| OPB46355 | T4742_S00008.422 | orphan protein |
| OPB46370 | T4742_S00008.437 | orphan protein |
| OPB46371 | T4742_S00008.438 | orphan protein |

| | | |
|---|---|---|
| OPB46378 | T4742_S00008.445 | orphan protein |
| OPB46396 | T4742_S00008.463 | orphan protein |
| OPB46427 | T4742_S00008.494 | orphan protein |
| OPB46452 | T4742_S00008.519 | orphan protein |
| OPB46457 | T4742_S00008.524 | orphan protein |
| OPB46469 | T4742_S00008.536 | orphan protein |
| OPB46478 | T4742_S00008.545 | orphan protein |
| OPB46480 | T4742_S00008.547 | orphan protein |
| OPB46502 | T4742_S00008.569 | orphan protein |
| OPB46509 | T4742_S00008.576 | orphan protein |
| OPB46521 | T4742_S00008.588 | orphan protein |
| OPB46536 | T4742_S00008.603 | orphan protein |
| OPB46581 | T4742_S00009.21 | orphan protein |
| OPB46586 | T4742_S00009.26 | orphan protein |
| OPB46591 | T4742_S00009.31 | orphan protein |
| OPB46598 | T4742_S00009.40 | orphan protein |
| OPB46599 | T4742_S00009.41 | orphan protein |
| OPB46635 | T4742_S00009.77 | orphan protein |
| OPB46638 | T4742_S00009.80 | orphan protein |
| OPB46639 | T4742_S00009.81 | orphan protein |
| OPB46649 | T4742_S00009.91 | orphan protein |
| OPB46685 | T4742_S00009.127 | orphan protein |
| OPB46702 | T4742_S00009.144 | orphan protein |
| OPB46721 | T4742_S00009.163 | orphan protein |
| OPB46731 | T4742_S00009.173 | orphan protein |
| OPB46750 | T4742_S00009.192 | orphan protein |
| OPB46752 | T4742_S00009.194 | orphan protein |
| OPB46758 | T4742_S00009.200 | orphan protein |
| OPB46767 | T4742_S00009.209 | orphan protein |
| OPB46779 | T4742_S00009.221 | orphan protein |
| OPB46780 | T4742_S00009.222 | orphan protein |
| OPB46844 | T4742_S00009.286 | orphan protein |
| OPB46850 | T4742_S00009.292 | orphan protein |
| OPB46852 | T4742_S00009.294 | orphan protein |
| OPB46887 | T4742_S00009.329 | orphan protein |
| OPB46888 | T4742_S00009.330 | orphan protein |

| | | |
|---|---|---|
| OPB46924 | T4742_S00009.366 | orphan protein |
| OPB46933 | T4742_S00009.375 | orphan protein |
| OPB46935 | T4742_S00009.377 | orphan protein |
| OPB46945 | T4742_S00009.387 | orphan protein |
| OPB46946 | T4742_S00009.388 | orphan protein |
| OPB46947 | T4742_S00009.389 | orphan protein |
| OPB46948 | T4742_S00009.390 | orphan protein |
| OPB46949 | T4742_S00009.391 | orphan protein |
| OPB46963 | T4742_S00009.405 | orphan protein |
| OPB46965 | T4742_S00009.407 | orphan protein |
| OPB46979 | T4742_S00009.421 | orphan protein |
| OPB47006 | T4742_S00009.448 | orphan protein |
| OPB47009 | T4742_S00009.451 | orphan protein |
| OPB47022 | T4742_S00009.464 | orphan protein |
| OPB47023 | T4742_S00009.465 | orphan protein |
| OPB47073 | T4742_S00009.515 | orphan protein |
| OPB47078 | T4742_S00009.520 | orphan protein |
| OPB47080 | T4742_S00009.522 | orphan protein |
| OPB47094 | T4742_S00009.536 | orphan protein |
| OPB47098 | T4742_S00009.540 | orphan protein |
| OPB47108 | T4742_S00009.550 | orphan protein |
| OPB47109 | T4742_S00009.551 | orphan protein |
| OPB47122 | T4742_S00009.564 | orphan protein |
| OPB47123 | T4742_S00009.565 | orphan protein |
| OPB47149 | T4742_S00009.591 | orphan protein |
| OPB47175 | T4742_S00009.617 | orphan protein |
| OPB44134 | T4742_S00003.405 | OTU-like cysteine protease, putative |
| OPB37519 | T4742_S00005.546 | outer membrane protein TOM13 |
| OPB37775 | T4742_S00017.42 | oxalate decarboxylase |
| OPB40837 | T4742_S00001.918 | oxalate decarboxylase |
| OPB42686 | T4742_S00004.792 | oxalate decarboxylase |
| OPB46026 | T4742_S00008.92 | oxaloacetase-like protein |
| OPB36933 | T4742_S00007.651 | oxidation resistance protein 1, putative |
| OPB42623 | T4742_S00004.728 | oxidoreductase CipA [Pochonia chlamydosporia 170] |
| OPB47085 | T4742_S00009.527 | oxidoreductase CipA-like, putative [Aspergillus fumigatus A1163] |
| OPB46989 | T4742_S00009.431 | oxidoreductase FAD-binding domain protein |

| | | |
|---|---|---|
| OPB41505 | T4742_S00011.348 | oxoglutarate/iron-dependent oxygenase |
| OPB36677 | T4742_S00007.395 | oxysterol binding protein |
| OPB38995 | T4742_S00006.54 | oxysterol binding protein |
| OPB40446 | T4742_S00001.525 | oxysterol binding protein |
| OPB45151 | T4742_S00010.58 | PabA, Anthranilate/para-aminobenzoate synthases component II |
| OPB46929 | T4742_S00009.371 | Paired amphipathic helix |
| OPB38513 | T4742_S00002.562 | PalI/RIM9 pH regulation protein |
| OPB45530 | T4742_S00010.441 | palmitoyl protein thioesterase |
| OPB37893 | T4742_S00017.160 | palmitoyltransferase |
| OPB45237 | T4742_S00010.146 | Palmitoyltransferase ERF2 |
| OPB38647 | T4742_S00002.696 | palmitoyltransferase PFA5, putative |
| OPB41985 | T4742_S00004.87 | Palmitoyltransferase SWF1 |
| OPB42531 | T4742_S00004.633 | Pantoate_beta-alanine ligase |
| OPB39583 | T4742_S00006.643 | PAP2 domain protein [Trichoderma guizhouense] |
| OPB36631 | T4742_S00007.349 | paptidase S8, subtilisin |
| OPB41391 | T4742_S00011.232 | paptidase S8, subtilisin |
| OPB46441 | T4742_S00008.508 | paptidase S8, subtilisin |
| OPB37301 | T4742_S00005.328 | Para-aminobenzoate (PABA) synthase PabaA |
| OPB36017 | T4742_S00023.52 | patched sphingolipid transporter |
| OPB38505 | T4742_S00002.554 | pathotenate kinase |
| OPB41032 | T4742_S00020.56 | paxU orthologue ? (indole-terpene biosynthesis?) |
| OPB38449 | T4742_S00002.497 | PDI related protein Eps1 |
| OPB42603 | T4742_S00004.707 | PDI related protein Mpd1/PrpA |
| OPB36422 | T4742_S00007.139 | PDR-type ABC transporters |
| OPB36591 | T4742_S00007.308 | PDR-type ABC transporters |
| OPB36629 | T4742_S00007.347 | PDR-type ABC transporters |
| OPB40554 | T4742_S00001.633 | PDR-type ABC transporters |
| OPB40694 | T4742_S00001.774 | PDR-type ABC transporters |
| OPB40926 | T4742_S00001.1007 | PDR-type ABC transporters |
| OPB45838 | T4742_S00014.173 | PDR-type ABC transporters |
| OPB46600 | T4742_S00009.42 | Pentafunctional Aromatic Polypeptide |
| OPB40332 | T4742_S00001.411 | PEP phosphonomutase |
| OPB43474 | T4742_S00019.207 | PEP_mutase |
| OPB46871 | T4742_S00009.313 | Pep1/vps10, transmembrane sorting receptor |
| OPB43358 | T4742_S00019.91 | PepN Aminopeptidase N |
| OPB38064 | T4742_S00002.111 | peptidase A1, aspartyl protease |

| | | |
|---|---|---|
| OPB40886 | T4742_S00001.967 | peptidase A1, aspartyl protease |
| OPB42632 | T4742_S00004.737 | peptidase A1, aspartyl protease |
| OPB43481 | T4742_S00019.215 | peptidase A1, aspartyl protease |
| OPB44316 | T4742_S00003.587 | peptidase A1, aspartyl protease |
| OPB44317 | T4742_S00003.588 | peptidase A1, aspartyl protease |
| OPB45209 | T4742_S00010.117 | peptidase A1, aspartyl protease |
| OPB45604 | T4742_S00010.515 | peptidase A1, aspartyl protease |
| OPB45634 | T4742_S00010.545 | peptidase A1, aspartyl protease |
| OPB46047 | T4742_S00008.113 | peptidase A1, aspartyl protease |
| OPB46151 | T4742_S00008.218 | peptidase A1, aspartyl protease |
| OPB46230 | T4742_S00008.297 | peptidase A1, aspartyl protease |
| OPB36909 | T4742_S00007.627 | Peptidase C12, ubiquitin carboxyl-terminal hydrolase 1 |
| OPB40137 | T4742_S00001.216 | Peptidase C19, ubiquitin carboxyl-terminal hydrolase 2 |
| OPB41817 | T4742_S00018.140 | Peptidase C19, ubiquitin carboxyl-terminal hydrolase 2 |
| OPB42862 | T4742_S00012.99 | Peptidase C19, ubiquitin carboxyl-terminal hydrolase 2 |
| OPB43997 | T4742_S00003.268 | Peptidase C19, ubiquitin carboxyl-terminal hydrolase 2, putative |
| OPB40791 | T4742_S00001.872 | peptidase C45 acyl-coenzyme A:6-aminopenicillanic acid acyl-transferase |
| OPB40177 | T4742_S00001.256 | Peptidase C48, SUMO/Sentrin/Ubl1 |
| OPB47079 | T4742_S00009.521 | Peptidase C48, SUMO/Sentrin/Ubl1 |
| OPB45201 | T4742_S00010.109 | Peptidase C50, separase bimB |
| OPB42854 | T4742_S00012.91 | Peptidase D |
| OPB46677 | T4742_S00009.119 | Peptidase D |
| OPB39932 | T4742_S00001.11 | peptidase family C78 [Colletotrichum fioriniae PJ7] |
| OPB44607 | T4742_S00003.879 | peptidase family M28 |
| OPB44880 | T4742_S00013.185 | Peptidase family M28 protein |
| OPB42000 | T4742_S00004.102 | peptidase family M3 |
| OPB39102 | T4742_S00006.162 | peptidase family S58 |
| OPB37025 | T4742_S00005.52 | Peptidase G1, eqolisin |
| OPB44713 | T4742_S00013.17 | Peptidase G1, eqolisin |
| OPB46292 | T4742_S00008.359 | Peptidase G1, eqolisin |
| OPB41474 | T4742_S00011.317 | peptidase M, neutral metallopeptidase |
| OPB37834 | T4742_S00017.101 | Peptidase M, neutral zinc metallopeptidases, zinc-binding site |
| OPB42522 | T4742_S00004.624 | Peptidase M, neutral zinc metallopeptidases, zinc-binding site |
| OPB42523 | T4742_S00004.625 | Peptidase M, neutral zinc metallopeptidases, zinc-binding site |
| OPB39679 | T4742_S00015.23 | Peptidase M1 |
| OPB44938 | T4742_S00013.243 | Peptidase M1 |

| | | |
|---|---|---|
| OPB45985 | T4742_S00008.51 | peptidase M14 |
| OPB41867 | T4742_S00018.190 | peptidase M15 D-ala-D-ala dipeptidase |
| OPB37385 | T4742_S00005.412 | peptidase M18 |
| OPB43413 | T4742_S00019.146 | peptidase M20, zinc metallopeptidase |
| OPB45773 | T4742_S00014.108 | peptidase M20, zinc metallopeptidase |
| OPB40001 | T4742_S00001.80 | Peptidase M22, glycoprotease |
| OPB42780 | T4742_S00012.17 | Peptidase M24, X-Pro dipeptidase |
| OPB36236 | T4742_S00021.154 | Peptidase M4, thermolysin, putative |
| OPB38636 | T4742_S00002.685 | peptidase M43 |
| OPB37474 | T4742_S00005.501 | Peptidase M48, Ste24p |
| OPB36132 | T4742_S00021.49 | peptidase S1 chymotrypsin |
| OPB42272 | T4742_S00004.374 | peptidase S1 chymotrypsin |
| OPB36329 | T4742_S00007.46 | peptidase S10 serine carboxypeptidase |
| OPB36605 | T4742_S00007.323 | peptidase S10 serine carboxypeptidase |
| OPB45550 | T4742_S00010.461 | peptidase S10 serine carboxypeptidase |
| OPB46665 | T4742_S00009.107 | peptidase S10 serine carboxypeptidase |
| OPB44949 | T4742_S00013.254 | Peptidase S10, serine carboxypeptidase |
| OPB38539 | T4742_S00002.588 | Peptidase S15 |
| OPB40325 | T4742_S00001.404 | peptidase S15 |
| OPB40720 | T4742_S00001.800 | Peptidase S15 |
| OPB40757 | T4742_S00001.838 | Peptidase S15 |
| OPB40712 | T4742_S00001.792 | Peptidase S15, PepX |
| OPB41012 | T4742_S00020.36 | Peptidase S15, PepX |
| OPB43458 | T4742_S00019.191 | Peptidase S15, PepX |
| OPB35937 | T4742_S00025.10 | peptidase S28, serine carboxypeptidase |
| OPB37753 | T4742_S00017.20 | peptidase S28, serine carboxypeptidase |
| OPB40635 | T4742_S00001.715 | peptidase S28, serine carboxypeptidase |
| OPB41878 | T4742_S00018.201 | peptidase S28, serine carboxypeptidase |
| OPB44621 | T4742_S00003.893 | peptidase S28, serine carboxypeptidase |
| OPB36178 | T4742_S00021.96 | peptidase S35, alkaline serine protease sedolisin |
| OPB37026 | T4742_S00005.53 | peptidase S35, alkaline serine protease sedolisin |
| OPB37173 | T4742_S00005.200 | peptidase S35, alkaline serine protease sedolisin |
| OPB37200 | T4742_S00005.227 | peptidase S35, alkaline serine protease sedolisin |
| OPB37201 | T4742_S00005.228 | peptidase S35, alkaline serine protease sedolisin |
| OPB44927 | T4742_S00013.232 | peptidase S35, alkaline serine protease sedolisin |
| OPB42730 | T4742_S00004.836 | peptidase S41 |

| | | |
|---|---|---|
| OPB43816 | T4742_S00003.87 | peptidase S41 |
| OPB45663 | T4742_S00010.574 | Peptidase S41 |
| OPB40375 | T4742_S00001.454 | peptidase S53 family protein [Metarhizium robertsii] |
| OPB45626 | T4742_S00010.537 | peptidase S53 sedolisin |
| OPB46007 | T4742_S00008.73 | peptidase S53 sedolisin |
| OPB42879 | T4742_S00012.116 | peptidase S53, sedolisin |
| OPB44813 | T4742_S00013.118 | peptidase S53, sedolisin |
| OPB45862 | T4742_S00014.197 | peptidase S53, sedolisin |
| OPB45864 | T4742_S00014.199 | peptidase S53, sedolisin |
| OPB36960 | T4742_S00007.678 | Peptidase S54, rhomboid |
| OPB43764 | T4742_S00003.35 | Peptidase S58, DmpA |
| OPB47127 | T4742_S00009.569 | Peptidase S8 and S53, subtilisin, kexin, sedolisin |
| OPB43954 | T4742_S00003.225 | Peptidase S8 and S53, subtilisin, kexin, sedolisin |
| OPB41344 | T4742_S00011.185 | peptidase S8 subtilisin |
| OPB35950 | T4742_S00025.23 | peptidase S8 subtilisin |
| OPB36006 | T4742_S00023.41 | peptidase S8 subtilisin |
| OPB36877 | T4742_S00007.595 | peptidase S8 subtilisin |
| OPB38805 | T4742_S00002.854 | peptidase S8 subtilisin |
| OPB40677 | T4742_S00001.757 | peptidase S8 subtilisin |
| OPB42125 | T4742_S00004.227 | peptidase S8 subtilisin |
| OPB42850 | T4742_S00012.87 | peptidase S8 subtilisin |
| OPB43411 | T4742_S00019.144 | peptidase S8 subtilisin |
| OPB43456 | T4742_S00019.189 | peptidase S8 subtilisin |
| OPB43761 | T4742_S00003.32 | peptidase S8 subtilisin |
| OPB44085 | T4742_S00003.356 | peptidase S8 subtilisin |
| OPB44227 | T4742_S00003.498 | peptidase S8 subtilisin |
| OPB45967 | T4742_S00008.33 | Peptidase S8/S53, subtilisin/kexin/sedolisin [Penicillium camemberti] |
| OPB46193 | T4742_S00008.260 | Peptidase S8/S53, subtilisin/kexin/sedolisin [Penicillium occitanis] |
| OPB41163 | T4742_S00011.3 | Peptidase S9, prolyl oligopeptidase active site region |
| OPB36094 | T4742_S00021.10 | Peptidase S9, prolyl oligopeptidase, catalytic domain protein [Cordyceps confragosa RCEF 1005] |
| OPB37315 | T4742_S00005.342 | Peptidase, trypsin-like serine and cysteine |
| OPB40490 | T4742_S00001.569 | Peptidase_C14 |
| OPB43843 | T4742_S00003.114 | Peptidase_M20 |
| OPB42567 | T4742_S00004.670 | Peptidase_M28 |
| OPB36065 | T4742_S00024.26 | Peptidase_S8 |
| OPB42218 | T4742_S00004.320 | Peptide chain release factor eRF/aRF subunit 1 (eRF1). |

| | | |
|---|---|---|
| OPB40141 | T4742_S00001.220 | peptide chain release factor RF-1 |
| OPB37818 | T4742_S00017.85 | Peptide methionine sulfoxide reductase |
| OPB44542 | T4742_S00003.814 | peptidyl arginine deiminase |
| OPB39116 | T4742_S00006.176 | peptidyl-prolyl cis/trans isomerase |
| OPB43826 | T4742_S00003.97 | peptidyl-prolyl cis-trans isomerase |
| OPB42342 | T4742_S00004.444 | Peptidyl-prolyl cis-trans isomerase PIN4 (EC 5.2.1.8) (PPIase PIN4) |
| OPB39796 | T4742_S00015.140 | Peptidyl-prolyl cis-trans isomerase, cyclophilin type |
| OPB39802 | T4742_S00015.146 | Peptidyl-prolyl cis-trans isomerase, cyclophilin type |
| OPB39942 | T4742_S00001.21 | Peptidyl-prolyl cis-trans isomerase, cyclophilin-type |
| OPB45963 | T4742_S00008.29 | Peptidyl-prolyl cis-trans isomerase, cyclophilin-type |
| OPB37897 | T4742_S00017.164 | Peptidylprolyl isomerase, FKBP-type |
| OPB44839 | T4742_S00013.144 | Peptidyl-tRNA hydrolase |
| OPB43144 | T4742_S00012.381 | peptidyl-tRNA hydrolase [Ophiocordyceps sinensis CO18] |
| OPB36469 | T4742_S00007.186 | peroxidase/hem containing |
| OPB43087 | T4742_S00012.324 | peroxin 8, putative |
| OPB42232 | T4742_S00004.334 | Peroxin-3 |
| OPB41945 | T4742_S00004.47 | peroxisomal biogenesis factor 11 |
| OPB45945 | T4742_S00008.11 | peroxisomal carrier protein |
| OPB38165 | T4742_S00002.212 | peroxisomal hydratase-dehydrogenase-epimerase |
| OPB36003 | T4742_S00023.38 | peroxisomal membrane anchor protein (Pex14), putative |
| OPB43216 | T4742_S00022.19 | Peroxisomal membrane anchor protein (Pex14p), putative |
| OPB46865 | T4742_S00009.307 | peroxisomal membrane protein (PmpP24) |
| OPB38833 | T4742_S00002.882 | peroxisomal membrane protein pex16 |
| OPB38414 | T4742_S00002.462 | peroxisomal targeting signal 2 receptor, putative |
| OPB42985 | T4742_S00012.222 | peroxisome biosynthesis protein (PAS1/Peroxin-1), |
| OPB44268 | T4742_S00003.539 | Pex13 protein |
| OPB37094 | T4742_S00005.121 | Pex19 (peroxin), a 40 kDa farnesylated protein associated with peroxisomes. |
| OPB42943 | T4742_S00012.180 | pex2 pex12 amino terminal region [Colletotrichum incanum] |
| OPB44411 | T4742_S00003.682 | Pex2/Pex12 |
| OPB38301 | T4742_S00002.348 | Pex2/Pex12 amino terminal region |
| OPB36327 | T4742_S00007.44 | Pfs NACHT and ankyrin domain protein |
| OPB38930 | T4742_S00002.979 | Pfs NACHT and ankyrin domain protein |
| OPB39837 | T4742_S00015.181 | Pfs NACHT and ankyrin domain protein |
| OPB46038 | T4742_S00008.104 | Pfs NACHT and ankyrin domain protein |
| OPB46069 | T4742_S00008.136 | Pfs NACHT and ankyrin domain protein |
| OPB46637 | T4742_S00009.79 | Pfs NACHT and ankyrin domain protein |

| | | |
|---|---|---|
| OPB45179 | T4742_S00010.86 | Pfs,NACHT and Ankyrin domain protein |
| OPB43515 | T4742_S00016.35 | Pfs,NACHT and WD domain protein |
| OPB39641 | T4742_S00006.701 | PHD-finger domain protein |
| OPB36522 | T4742_S00007.239 | Phenazine biosynthesis PhzC/PhzF protein |
| OPB37382 | T4742_S00005.409 | phenylacetaldoxime dehydratase family protein |
| OPB37542 | T4742_S00005.569 | phenylacetaldoxime dehydratase family protein |
| OPB41437 | T4742_S00011.280 | phenylacetyl-CoA ligase [Aspergillus fumigatus Af293] |
| OPB45543 | T4742_S00010.454 | phenylacrylic acid decarboxylase [Aspergillus kawachii IFO 4308] |
| OPB38848 | T4742_S00002.897 | Phenylalanyl-tRNA synthetase |
| OPB39763 | T4742_S00015.107 | Phenylalanyl-tRNA synthetase |
| OPB36374 | T4742_S00007.91 | PhoG [Trichoderma guizhouense] |
| OPB45330 | T4742_S00010.241 | phosducin like protein class II |
| OPB41083 | T4742_S00020.107 | phosducin like protein, class I |
| OPB37860 | T4742_S00017.127 | phosphate phosphoenolpyruvate translocator |
| OPB36582 | T4742_S00007.299 | phosphate transporter |
| OPB39556 | T4742_S00006.616 | phosphate transporter |
| OPB44792 | T4742_S00013.97 | Phosphate transporter |
| OPB45608 | T4742_S00010.519 | phosphate transporter |
| OPB45702 | T4742_S00014.36 | phosphate transporter (Pho88) |
| OPB45411 | T4742_S00010.322 | phosphate/H+ symporter |
| OPB40401 | T4742_S00001.480 | Phosphatidate cytidylyltransferase |
| OPB44305 | T4742_S00003.576 | Phosphatidate phosphatase |
| OPB37589 | T4742_S00005.616 | phosphatidate-phosphatase |
| OPB41583 | T4742_S00011.426 | phosphatidyl inositol-3-kinase-like proteins involved in DNA damage response |
| OPB41918 | T4742_S00004.20 | phosphatidyl synthase |
| OPB46527 | T4742_S00008.594 | phosphatidyl synthase |
| OPB38327 | T4742_S00002.375 | Phosphatidylethanolamine methyltransferase |
| OPB44059 | T4742_S00003.330 | phosphatidylethanolamine N-methyltransferase |
| OPB47021 | T4742_S00009.463 | phosphatidylethanolamine-binding protein |
| OPB38676 | T4742_S00002.725 | Phosphatidylinositol 3- and 4-kinase |
| OPB43198 | T4742_S00022.1 | Phosphatidylinositol 3-kinase Vps34 |
| OPB37068 | T4742_S00005.95 | Phosphatidylinositol 4,5-bisphosphate 5-phosphatase |
| OPB41563 | T4742_S00011.406 | Phosphatidylinositol 4-kinase |
| OPB39234 | T4742_S00006.294 | phosphatidylinositol phospholipase C |
| OPB41656 | T4742_S00011.499 | phosphatidylinositol polyphosphate phosphatase |
| OPB37225 | T4742_S00005.252 | Phosphatidylinositol transfer protein |

| | | |
|---|---|---|
| OPB38645 | T4742_S00002.694 | Phosphatidylinositol transfer protein |
| OPB45509 | T4742_S00010.420 | phosphatidylinositol transporter, putative |
| OPB39808 | T4742_S00015.152 | Phosphatidylinositol:UDP-GlcNAc a-GlcNAc-transferase (Pig-A) |
| OPB37827 | T4742_S00017.94 | phosphatidylinositol-3-phosphate 5-kinase; fab1 |
| OPB37828 | T4742_S00017.95 | phosphatidylinositol-3-phosphate 5-kinase; fab1 |
| OPB43712 | T4742_S00016.232 | Phosphatidylinositol-4-kinase stt4 |
| OPB36468 | T4742_S00007.185 | Phosphatidylinositol-4-phosphate 5-kinase, mss4 |
| OPB44631 | T4742_S00003.903 | phosphatidylinositol-glycan biosynthesis class S protein |
| OPB43491 | T4742_S00016.11 | phosphatidylinositolglycan class N, putative |
| OPB37233 | T4742_S00005.260 | phosphatidylinositol-specific phospholipase C (MPLC1) |
| OPB41630 | T4742_S00011.473 | phosphatidylinositol-specific phospholipase C, Y domain, putative |
| OPB37696 | T4742_S00005.723 | Phosphatidylserine decarboxylase |
| OPB43621 | T4742_S00016.141 | phosphatidylserine decarboxylase |
| OPB45033 | T4742_S00013.338 | phosphatidylserine decarboxylase |
| OPB45104 | T4742_S00010.11 | Phosphatidylserine decarboxylase |
| OPB47173 | T4742_S00009.615 | Phosphatidylserine decarboxylase |
| OPB39016 | T4742_S00006.75 | Phosphatidylserine receptor protein, contains F-box and transcription factor jumonji |
| OPB36920 | T4742_S00007.638 | Phosphatidylserine synthase |
| OPB37606 | T4742_S00005.633 | Phosphatidylserine/phosphatidylglycerophosphate/cardiolipin synthase |
| OPB43831 | T4742_S00003.102 | phosphatidyltransferase |
| OPB42164 | T4742_S00004.266 | Phospho-2-dehydro-3-deoxyheptonate aldolase (EC 2.5.1.54) |
| OPB45242 | T4742_S00010.151 | Phosphoadenosine phosphosulfate reductase |
| OPB36990 | T4742_S00005.17 | phosphoadenosine phosphosulfate reductase |
| OPB45225 | T4742_S00010.134 | phosphodiesterase/nucleotide pyrophosphatasetype 1 |
| OPB36548 | T4742_S00007.265 | phosphoenolpyruvate carboxykinase AcuF |
| OPB42829 | T4742_S00012.66 | phosphoenolpyruvate synthase [Aspergillus bombycis] |
| OPB45009 | T4742_S00013.314 | phosphoesterase, putative |
| OPB39201 | T4742_S00006.261 | phosphoglucomutase/phosphomannomutase |
| OPB45567 | T4742_S00010.478 | Phosphoglucose isomerase (PGI) |
| OPB42014 | T4742_S00004.116 | Phosphoglycerate dehydrogenase and related dehydrogenases |
| OPB45079 | T4742_S00013.384 | Phosphoglycerate dehydrogenase and related dehydrogenases; RibA GTP cyclohydrolase II |
| OPB40278 | T4742_S00001.357 | phosphoglycerate kinase |
| OPB36164 | T4742_S00021.81 | phosphoglycerate mutase family |
| OPB36681 | T4742_S00007.399 | phosphoglycerate mutase family |
| OPB38639 | T4742_S00002.688 | phosphoglycerate mutase family |
| OPB43742 | T4742_S00003.13 | phosphoglycerate mutase family |

| | | |
|---|---|---|
| OPB43844 | T4742_S00003.115 | phosphoglycerate mutase family |
| OPB44025 | T4742_S00003.296 | phosphoglycerate mutase family |
| OPB45839 | T4742_S00014.174 | phosphoglycerate mutase family |
| OPB38946 | T4742_S00006.5 | Phosphoglycerate/bisphosphoglycerate mutase |
| OPB44402 | T4742_S00003.673 | Phosphoinositide phosphatase sac1, involved inProtein trafficking and secretion |
| OPB39931 | T4742_S00001.10 | phospholipase A2 protein family |
| OPB42835 | T4742_S00012.72 | phospholipase A2 protein family |
| OPB44165 | T4742_S00003.436 | phospholipase A2 protein family |
| OPB40309 | T4742_S00001.388 | phospholipase C |
| OPB45416 | T4742_S00010.327 | phospholipase C |
| OPB47024 | T4742_S00009.466 | phospholipase C |
| OPB43089 | T4742_S00012.326 | phospholipase C, inositol-specific |
| OPB37075 | T4742_S00005.102 | Phospholipase D |
| OPB38045 | T4742_S00002.92 | phospholipase D |
| OPB38450 | T4742_S00002.498 | phospholipase D |
| OPB40915 | T4742_S00001.996 | phospholipase D |
| OPB44824 | T4742_S00013.129 | phospholipase D |
| OPB43038 | T4742_S00012.275 | phospholipase of papatin-family |
| OPB44637 | T4742_S00003.909 | phospholipase of papatin-family |
| OPB36308 | T4742_S00007.25 | Phospholipid/glycerol acyltransferase |
| OPB45334 | T4742_S00010.245 | phospholipid-binding protein, partial [Trichoderma reesei RUT C-30] |
| OPB42322 | T4742_S00004.424 | phospholipid-translocating P-type ATPase |
| OPB46898 | T4742_S00009.340 | Phospholipid-translocating P-type ATPase |
| OPB35961 | T4742_S00025.34 | phospholipid-translocating P-type ATPase |
| OPB42994 | T4742_S00012.231 | Phosphomannomutase (SEC53) |
| OPB42121 | T4742_S00004.223 | Phosphomethylpyrimidine kinase |
| OPB46784 | T4742_S00009.226 | Phosphomethylpyrimidine kinase type-1 |
| OPB43165 | T4742_S00012.402 | Phosphopantetheine-binding protein |
| OPB41627 | T4742_S00011.470 | phosphopantetheinyl transferase, putative |
| OPB38706 | T4742_S00002.755 | phosphopantothenate-cysteine ligase |
| OPB39995 | T4742_S00001.74 | phosphoprotein phosphatase [Metarhizium acridum CQMa 102] |
| OPB36175 | T4742_S00021.93 | phosphoribosylamine-glycine ligase |
| OPB43550 | T4742_S00016.70 | phosphoribosylamine-glycine ligase |
| OPB38933 | T4742_S00002.982 | phosphoribosylaminoimidazole carboxylase |
| OPB39315 | T4742_S00006.375 | Phosphoribosylformimino-aminoimidazole carboxamide ribonucleotide (ProFAR) isomerase |
| OPB41963 | T4742_S00004.65 | Phosphoribosylpyrophosphate synthetase |

| | | |
|---|---|---|
| OPB43603 | T4742_S00016.123 | Phosphoribosylpyrophosphate synthetase |
| OPB45909 | T4742_S00014.245 | phosphoribulokinase/uridine kinase family protein |
| OPB43958 | T4742_S00003.229 | phosphoribulokinase/uridine kinase family protein |
| OPB43280 | T4742_S00019.13 | phosphoserine phosphatase |
| OPB43782 | T4742_S00003.53 | phosphotransferase enzyme family protein |
| OPB43979 | T4742_S00003.250 | phosphotyrosine protein phosphatase |
| OPB41900 | T4742_S00004.2 | Phosphotyrosyl phosphatase activator, PTPA |
| OPB46755 | T4742_S00009.197 | Phosphotyrosyl phosphatase activator, PTPA |
| OPB45804 | T4742_S00014.139 | ph-response regulator protein pala rim-20 [Fusarium avenaceum] |
| OPB41447 | T4742_S00011.290 | pH-response regulator protein palC |
| OPB39475 | T4742_S00006.535 | Phytanoyl-CoA dioxygenase |
| OPB43735 | T4742_S00003.6 | Phytanoyl-CoA dioxygenase |
| OPB44161 | T4742_S00003.432 | Phytanoyl-CoA dioxygenase |
| OPB46104 | T4742_S00008.171 | Phytanoyl-CoA dioxygenase |
| OPB46513 | T4742_S00008.580 | Phytanoyl-CoA dioxygenase |
| OPB37992 | T4742_S00002.39 | phytase |
| OPB39227 | T4742_S00006.287 | phytase |
| OPB42099 | T4742_S00004.201 | phytase |
| OPB44962 | T4742_S00013.267 | phytase |
| OPB41570 | T4742_S00011.413 | pirin |
| OPB42187 | T4742_S00004.289 | pirin |
| OPB36092 | T4742_S00021.8 | PKS |
| OPB36124 | T4742_S00021.41 | PKS |
| OPB36200 | T4742_S00021.118 | PKS |
| OPB37351 | T4742_S00005.378 | PKS |
| OPB37527 | T4742_S00005.554 | PKS |
| OPB37944 | T4742_S00017.211 | PKS |
| OPB37950 | T4742_S00017.217 | PKS |
| OPB38808 | T4742_S00002.857 | PKS |
| OPB40686 | T4742_S00001.766 | PKS |
| OPB41371 | T4742_S00011.212 | PKS |
| OPB41436 | T4742_S00011.279 | PKS |
| OPB41643 | T4742_S00011.486 | PKS |
| OPB43931 | T4742_S00003.202 | PKS |
| OPB44035 | T4742_S00003.306 | PKS |
| OPB44473 | T4742_S00003.745 | PKS |

| | | |
|---|---|---|
| OPB44510 | T4742_S00003.782 | PKS |
| OPB45495 | T4742_S00010.406 | PKS |
| OPB45499 | T4742_S00010.410 | PKS |
| OPB45887 | T4742_S00014.222 | PKS |
| OPB45893 | T4742_S00014.229 | PKS |
| OPB46148 | T4742_S00008.215 | PKS |
| OPB46335 | T4742_S00008.402 | PKS |
| OPB46501 | T4742_S00008.568 | PKS |
| OPB46686 | T4742_S00009.128 | PKS |
| OPB36213 | T4742_S00021.131 | PKS-NRPS |
| OPB38892 | T4742_S00002.941 | PKS-NRPS |
| OPB41699 | T4742_S00018.21 | PKS-NRPS |
| OPB45595 | T4742_S00010.506 | PKS-NRPS |
| OPB42484 | T4742_S00004.586 | PL20 protein |
| OPB42715 | T4742_S00004.821 | PL7 alignate lyase |
| OPB41310 | T4742_S00011.151 | PL7 subfam. 4 alginate lyase-related |
| OPB38542 | T4742_S00002.591 | PL7_4 alginate lyase |
| OPB42776 | T4742_S00012.13 | PL8 alginate lyase, putative |
| OPB40417 | T4742_S00001.496 | PL8 polysaccharide lyase; distantly related to chondroitin lyases |
| OPB40853 | T4742_S00001.934 | plasma membrane H+ ATPase |
| OPB39736 | T4742_S00015.80 | plasma membrane H+ ATPase |
| OPB39945 | T4742_S00001.24 | Pleckstrin/ G-protein, interacting region |
| OPB43339 | T4742_S00019.72 | P-loop containing nucleoside triphosphate hydrolase protein [Auricularia subglabra TFB-10046 SS5] |
| OPB35998 | T4742_S00023.33 | PNP_UDP_1 |
| OPB38108 | T4742_S00002.155 | PNP_UDP_1,Peptidase_S8 |
| OPB44608 | T4742_S00003.880 | poly(3-hydroxybutyrate) depolymerase |
| OPB39433 | T4742_S00006.493 | Poly(A) binding protein, |
| OPB36821 | T4742_S00007.539 | poly(A) polymerase with similarity to the DNA polymerase sigma family |
| OPB45949 | T4742_S00008.15 | Poly(A) polymerase, RNA-binding region |
| OPB45293 | T4742_S00010.204 | Poly(ADP-ribose) glycohydrolase isoform |
| OPB36265 | T4742_S00021.184 | poly(aspartic acid) hydrolase |
| OPB37503 | T4742_S00005.530 | polyA polymerase (Poly[ADP-ribose] synthetase) |
| OPB43082 | T4742_S00012.319 | Polyadenylation factor subunit 2, putative |
| OPB42905 | T4742_S00012.142 | polyadenylation factor subunit CstF64 |
| OPB36659 | T4742_S00007.377 | Polynucleotide 5'-hydroxyl-kinase GRC3, putative |
| OPB43492 | T4742_S00016.12 | polynucleotide adenylyltransferase. |

| | | |
|---|---|---|
| OPB46459 | T4742_S00008.526 | polynucleotide kinase 3 phosphatase |
| OPB39914 | T4742_S00015.258 | polyphosphoinositide phosphatase, putative |
| OPB36067 | T4742_S00024.28 | polysaccharide deacetylase family protein [Trichoderma harzianum] |
| OPB44453 | T4742_S00003.725 | Polyubiquitin-binding protein Dsk2 |
| OPB37091 | T4742_S00005.118 | porin, outer mitochondrial membrane protein |
| OPB44052 | T4742_S00003.323 | porphobilinogen deaminase-like protein |
| OPB40196 | T4742_S00001.275 | porphobilinogen synthase-like protein |
| OPB45090 | T4742_S00013.395 | POS9-activating factor FAP7 |
| OPB44030 | T4742_S00003.301 | potassium transporter 8 |
| OPB40021 | T4742_S00001.100 | PQ loop repeat protein [Cordyceps militaris CM01] |
| OPB45278 | T4742_S00010.189 | hydrophobin HFB15 |
| OPB41153 | T4742_S00020.177 | Predicted acyltransferases |
| OPB35949 | T4742_S00025.22 | predicted protein [Trichoderma reesei QM6a] |
| OPB36649 | T4742_S00007.367 | prefoldin chaperone |
| OPB39487 | T4742_S00006.547 | prefoldin chaperone |
| OPB39598 | T4742_S00006.658 | prefoldin chaperone |
| OPB39938 | T4742_S00001.17 | prefoldin chaperone |
| OPB43778 | T4742_S00003.49 | prefoldin chaperone |
| OPB39648 | T4742_S00006.708 | prefoldin subunit 1, putative |
| OPB42968 | T4742_S00012.205 | prefoldin subunit 2 |
| OPB36471 | T4742_S00007.188 | prefoldin subunit 6, putative |
| OPB42094 | T4742_S00004.196 | pre-mRNA branch site protein p14 |
| OPB46683 | T4742_S00009.125 | pre-mRNA processing splicing factor 8 |
| OPB41944 | T4742_S00004.46 | pre-mRNA splicing factor |
| OPB38243 | T4742_S00002.290 | pre-mRNA splicing factor CEF1, putative |
| OPB38794 | T4742_S00002.843 | pre-mRNA splicing factor slt11 |
| OPB39572 | T4742_S00006.632 | pre-mRNA splicing factor, putative |
| OPB38722 | T4742_S00002.771 | pre-mRNA splicing factor, putative |
| OPB43893 | T4742_S00003.164 | pre-mRNA splicing factor, putative [Cryptococcus neoformans]. |
| OPB37008 | T4742_S00005.35 | pre-mRNA splicing helicase |
| OPB46813 | T4742_S00009.255 | PRP5 |
| OPB37910 | T4742_S00017.177 | pre-mRNA-processing factor 17 |
| OPB37067 | T4742_S00005.94 | pre-mRNA-processing factor 39 |
| OPB39006 | T4742_S00006.65 | Pre-mRNA-splicing ATP-dependent RNA helicase PRP28 |
| OPB46680 | T4742_S00009.122 | pre-mRNA-splicing factor 18 |
| OPB39571 | T4742_S00006.631 | pre-mRNA-splicing factor clf1 |

| | | |
|---|---|---|
| OPB37340 | T4742_S00005.367 | pre-mRNA-splicing factor CWC2 |
| OPB45154 | T4742_S00010.61 | pre-mRNA-splicing factor CWC21, putative |
| OPB41262 | T4742_S00011.103 | pre-mRNA-splicing factor CWC25, putative |
| OPB42318 | T4742_S00004.420 | Pre-mRNA-splicing factor ini1 |
| OPB41362 | T4742_S00011.203 | Pre-mRNA-splicing factor ISY1 |
| OPB45325 | T4742_S00010.236 | pre-mRNA-splicing factor PRP38 |
| OPB39373 | T4742_S00006.433 | Pre-mRNA-splicing factor RSE1 |
| OPB38556 | T4742_S00002.605 | pre-mRNA-splicing factor SLU7 |
| OPB38998 | T4742_S00006.57 | Prenylated Rab acceptor 1 Yip3 |
| OPB36683 | T4742_S00007.401 | prenylcysteine lyase, putative |
| OPB47010 | T4742_S00009.452 | prenyltransferase and squalene oxidase |
| OPB36303 | T4742_S00007.20 | prenyltransferase, related to E. nidulans CaaX farnesyltransferase beta subunit ram1 |
| OPB41521 | T4742_S00011.364 | Prephenate dehydratase |
| OPB40963 | T4742_S00001.1044 | Prephenate dehydrogenase |
| OPB36498 | T4742_S00007.215 | pre-rRNA processing nucleolar protein Sik1, putative |
| OPB45390 | T4742_S00010.301 | pre-rRNA processing protein Esf1 |
| OPB39706 | T4742_S00015.50 | pre-rRNA processing protein Rrp12 |
| OPB35958 | T4742_S00025.31 | pre-rRNA-processing protein IPI1, putative |
| OPB39610 | T4742_S00006.670 | pre-rRNA-processing protein PNO1 |
| OPB36338 | T4742_S00007.55 | pre-rRNA-processing protein TSR2 , putative |
| OPB40716 | T4742_S00001.796 | probable aromatic ring-opening dioxygenase family protein [Ramularia collo-cygni] |
| OPB46602 | T4742_S00009.44 | probable vacuolar protein sorting protein VpsB [Fusarium fujikuroi IMI 58289] |
| OPB41930 | T4742_S00004.32 | prohibitin-2 [Cordyceps confragosa RCEF 1005] |
| OPB36583 | T4742_S00007.300 | Prolidase and Aminopeptidase P [Trichoderma guizhouense] |
| OPB46785 | T4742_S00009.227 | proliferating cell nuclear antigen-like [Nilaparvata lugens] |
| OPB42676 | T4742_S00004.781 | Proline dehydrogenase |
| OPB42806 | T4742_S00012.43 | Proline dehydrogenase-family protein |
| OPB39021 | T4742_S00006.80 | proline iminopeptidase |
| OPB38503 | T4742_S00002.552 | Prolyl 4-hydroxylase, alpha subunit |
| OPB42784 | T4742_S00012.21 | Prolyl 4-hydroxylase, alpha subunit |
| OPB43118 | T4742_S00012.355 | Prolyl 4-hydroxylase, alpha subunit |
| OPB38214 | T4742_S00002.261 | prolyl aminopeptidase |
| OPB38361 | T4742_S00002.409 | Prolyl endopeptidase |
| OPB47001 | T4742_S00009.443 | prolyl-tRNA synthetase, class IIa. |
| OPB47052 | T4742_S00009.494 | Proteasome component Ecm29 |
| OPB38864 | T4742_S00002.913 | Proteasome component Ecm29 [Trichoderma guizhouense] |

| | | |
|---|---|---|
| OPB38340 | T4742_S00002.388 | Proteasome maturation factor UMP1 |
| OPB42252 | T4742_S00004.354 | protein  phosphatase 2A activator TIP41 |
| OPB36225 | T4742_S00021.143 | Protein bli-3 [Talaromyces marneffei PM1] |
| OPB37064 | T4742_S00005.91 | Protein disulfide isomerase 1 |
| OPB40086 | T4742_S00001.165 | Protein disulfide isomerase TigA |
| OPB37248 | T4742_S00005.275 | Protein farnesyltransferase, alpha subunit |
| OPB42310 | T4742_S00004.412 | protein for structural maintenance of chromosome protein 3 (sister chromatid cohesion complex Cohesin, subunit SMC3) |
| OPB38218 | T4742_S00002.265 | Protein import receptor MAS20 |
| OPB44092 | T4742_S00003.363 | Protein interacting with poly(A)-binding protein |
| OPB43178 | T4742_S00012.415 | protein involved in diphthamide synthesis. |
| OPB43208 | T4742_S00022.11 | Protein involved in ER to Golgi transport Sly41 |
| OPB45467 | T4742_S00010.378 | Protein involved in regulation of arginine-responsive genes |
| OPB42337 | T4742_S00004.439 | Protein invovled in ER retention Rer1 |
| OPB36291 | T4742_S00007.8 | Protein kinase |
| OPB37119 | T4742_S00005.146 | Protein kinase |
| OPB37558 | T4742_S00005.585 | Protein kinase |
| OPB38149 | T4742_S00002.196 | Protein kinase |
| OPB38297 | T4742_S00002.344 | Protein kinase |
| OPB40185 | T4742_S00001.264 | Protein kinase |
| OPB40437 | T4742_S00001.516 | Protein kinase |
| OPB41045 | T4742_S00020.69 | Protein kinase |
| OPB41060 | T4742_S00020.84 | protein kinase |
| OPB41233 | T4742_S00011.74 | Protein kinase |
| OPB41342 | T4742_S00011.183 | Protein kinase |
| OPB41808 | T4742_S00018.131 | protein kinase |
| OPB41810 | T4742_S00018.133 | Protein kinase |
| OPB42251 | T4742_S00004.353 | Protein kinase |
| OPB42710 | T4742_S00004.816 | Protein kinase |
| OPB43030 | T4742_S00012.267 | Protein kinase |
| OPB43079 | T4742_S00012.316 | Protein kinase |
| OPB43268 | T4742_S00019.1 | protein kinase |
| OPB43907 | T4742_S00003.178 | protein kinase |
| OPB44072 | T4742_S00003.343 | protein kinase |
| OPB44121 | T4742_S00003.392 | Protein kinase |
| OPB44565 | T4742_S00003.837 | protein kinase |
| OPB44628 | T4742_S00003.900 | protein kinase |

| | | |
|---|---|---|
| OPB44834 | T4742_S00013.139 | Protein kinase |
| OPB45096 | T4742_S00010.3 | Protein kinase |
| OPB45434 | T4742_S00010.345 | Protein kinase |
| OPB45952 | T4742_S00008.18 | Protein kinase |
| OPB45977 | T4742_S00008.43 | Protein kinase |
| OPB46066 | T4742_S00008.133 | Protein kinase |
| OPB46550 | T4742_S00008.617 | Protein kinase |
| OPB46774 | T4742_S00009.216 | Protein kinase |
| OPB46775 | T4742_S00009.217 | Protein kinase |
| OPB46970 | T4742_S00009.412 | Protein kinase |
| OPB47012 | T4742_S00009.454 | Protein kinase |
| OPB47029 | T4742_S00009.471 | Protein kinase |
| OPB36342 | T4742_S00007.59 | protein kinase STK49 |
| OPB41657 | T4742_S00011.500 | protein kinase A, catalytic subunit |
| OPB43278 | T4742_S00019.11 | Protein kinase C |
| OPB45004 | T4742_S00013.309 | protein kinase CAMK, STK26 |
| OPB37470 | T4742_S00005.497 | protein kinase family protein |
| OPB46263 | T4742_S00008.330 | protein kinase IME2, inducer of meiosis |
| OPB39555 | T4742_S00006.615 | protein kinase Rim15, response regulator receiver p |
| OPB38274 | T4742_S00002.321 | protein kinase RIO1 |
| OPB44682 | T4742_S00003.954 | Protein kinase, Ca2+-dependent |
| OPB42169 | T4742_S00004.271 | Protein kinase, related to N. crassa PSK-2 |
| OPB42172 | T4742_S00004.274 | Protein kinase, related to N. crassa SCK-1 |
| OPB45291 | T4742_S00010.202 | Protein kinase, related to NIM1 |
| OPB42247 | T4742_S00004.349 | protein kinase, related to S. cerevisiae Cla4p |
| OPB44448 | T4742_S00003.720 | Protein kinase-like (PK-like) [Glarea lozoyensis ATCC 20868] |
| OPB44569 | T4742_S00003.841 | Protein kinase-like domain protein [Cordyceps brongniartii RCEF 3172] |
| OPB37232 | T4742_S00005.259 | protein palmitoyl transferase PFA3 |
| OPB37878 | T4742_S00017.145 | protein phosphatase |
| OPB39774 | T4742_S00015.118 | protein phosphatase 2A |
| OPB39649 | T4742_S00006.709 | protein phosphatase 2A regulatory B subunit |
| OPB43292 | T4742_S00019.25 | Protein phosphatase 2A, catalytic domain |
| OPB37229 | T4742_S00005.256 | Protein phosphatase 2A, regulatory B subunit, B56 |
| OPB42379 | T4742_S00004.481 | Protein phosphatase 2C |
| OPB42400 | T4742_S00004.502 | protein phosphatase 2C |
| OPB41090 | T4742_S00020.114 | Protein phosphatase 2C type Ptc1 |

| | | |
|---|---|---|
| OPB44763 | T4742_S00013.68 | Protein phosphatase 2C, N-terminal |
| OPB41449 | T4742_S00011.292 | Protein phosphatase 2C-like |
| OPB46093 | T4742_S00008.160 | protein phosphatase myotubularin |
| OPB38250 | T4742_S00002.297 | Protein phosphatase PP2A associated protein (TAP42 like protein) |
| OPB46091 | T4742_S00008.158 | protein phosphatase PP2A regulatory subunit A |
| OPB44086 | T4742_S00003.357 | Protein phosphatase type 2C Ptc2 |
| OPB44237 | T4742_S00003.508 | Protein PNG1 [Escovopsis weberi] |
| OPB39094 | T4742_S00006.153 | Protein PNS1 [Trichoderma guizhouense] |
| OPB36258 | T4742_S00021.176 | protein RDR1 [Trichoderma harzianum] |
| OPB37861 | T4742_S00017.128 | protein required for sporulation |
| OPB36898 | T4742_S00007.616 | Protein synthesis factor, GTP-binding |
| OPB42018 | T4742_S00004.120 | protein synthesis factor, GTP-binding (elongation factor Tu). |
| OPB47143 | T4742_S00009.585 | Protein transport protein Sec1 |
| OPB45073 | T4742_S00013.378 | Protein transport protein sec39 [Valsa mali var. pyri] |
| OPB46991 | T4742_S00009.433 | Protein transport protein Yos1 |
| OPB39492 | T4742_S00006.552 | protein tyrosin kinase |
| OPB39951 | T4742_S00001.30 | Protein Tyrosine phosphatase |
| OPB41937 | T4742_S00004.39 | Protein Tyrosine phosphatase |
| OPB42050 | T4742_S00004.152 | protein tyrosine phosphatase |
| OPB42077 | T4742_S00004.179 | Protein Tyrosine phosphatase |
| OPB42267 | T4742_S00004.369 | Protein tyrosine phosphatase |
| OPB45507 | T4742_S00010.418 | protein tyrosine phosphatase |
| OPB45706 | T4742_S00014.40 | Protein Tyrosine phosphatase |
| OPB46429 | T4742_S00008.496 | protein tyrosine phosphatase |
| OPB36646 | T4742_S00007.364 | Protein tyrosine phosphatases |
| OPB39274 | T4742_S00006.334 | Protein tyrosine phosphatases |
| OPB39681 | T4742_S00015.25 | Protein tyrosine phosphatases |
| OPB46343 | T4742_S00008.410 | Protein tyrosine phosphatases |
| OPB45848 | T4742_S00014.183 | Protein urg3 [Valsa mali var. pyri] |
| OPB46565 | T4742_S00009.5 | protein VTS1 [Trichoderma gamsii] |
| OPB45555 | T4742_S00010.466 | Protein-tyrosine phosphatase, dual specificity |
| OPB40808 | T4742_S00001.889 | proteophosphoglycan PPG4 |
| OPB44646 | T4742_S00003.918 | Protoheme IX farnesyltransferase |
| OPB39437 | T4742_S00006.497 | protoporphyrinogen oxidase, putative |
| OPB40131 | T4742_S00001.210 | Pseudouridylate synthase TruB |
| OPB42915 | T4742_S00012.152 | Pso2 (Snm1), involved in DNA interstrand crosslink repair |

| | | |
|---|---|---|
| OPB36369 | T4742_S00007.86 | PTH11-like receptor |
| OPB36544 | T4742_S00007.261 | PTH11-like receptor |
| OPB36689 | T4742_S00007.407 | PTH11-like receptor |
| OPB36788 | T4742_S00007.506 | PTH11-like receptor |
| OPB36797 | T4742_S00007.515 | PTH11-like receptor |
| OPB37178 | T4742_S00005.205 | PTH11-like receptor |
| OPB37706 | T4742_S00005.734 | PTH11-like receptor |
| OPB37779 | T4742_S00017.46 | PTH11-like receptor |
| OPB38309 | T4742_S00002.356 | PTH11-like receptor |
| OPB38653 | T4742_S00002.702 | PTH11-like receptor |
| OPB38677 | T4742_S00002.726 | PTH11-like receptor |
| OPB38769 | T4742_S00002.818 | PTH11-like receptor |
| OPB40032 | T4742_S00001.111 | PTH11-like receptor |
| OPB40555 | T4742_S00001.634 | PTH11-like receptor |
| OPB40774 | T4742_S00001.855 | PTH11-like receptor |
| OPB40796 | T4742_S00001.877 | PTH11-like receptor |
| OPB41040 | T4742_S00020.64 | PTH11-like receptor |
| OPB43350 | T4742_S00019.83 | PTH11-like receptor |
| OPB43855 | T4742_S00003.126 | PTH11-like receptor |
| OPB44492 | T4742_S00003.764 | PTH11-like receptor |
| OPB44545 | T4742_S00003.817 | PTH11-like receptor |
| OPB44547 | T4742_S00003.819 | PTH11-like receptor |
| OPB45624 | T4742_S00010.535 | PTH11-like receptor |
| OPB45643 | T4742_S00010.554 | PTH11-like receptor |
| OPB45700 | T4742_S00014.34 | PTH11-like receptor |
| OPB45865 | T4742_S00014.200 | PTH11-like receptor |
| OPB46015 | T4742_S00008.81 | PTH11-like receptor |
| OPB46072 | T4742_S00008.139 | PTH11-like receptor |
| OPB46242 | T4742_S00008.309 | PTH11-like receptor |
| OPB46426 | T4742_S00008.493 | PTH11-like receptor |
| OPB46827 | T4742_S00009.269 | PTH11-like receptor |
| OPB46846 | T4742_S00009.288 | PTH11-like receptor |
| OPB37212 | T4742_S00005.239 | P-type ATPase |
| OPB37325 | T4742_S00005.352 | P-type ATPase with putative aminophospholipid translocase activity, neo1 |
| OPB40551 | T4742_S00001.630 | purine and uridine phosphorylase [Trichoderma reesei RUT C-30] |
| OPB41049 | T4742_S00020.73 | purine and uridine phosphorylase [Trichoderma reesei RUT C-30] |

| | | |
|---|---|---|
| OPB41114 | T4742_S00020.138 | purine and uridine phosphorylase [Trichoderma reesei RUT C-30] |
| OPB41148 | T4742_S00020.172 | purine and uridine phosphorylase [Trichoderma reesei RUT C-30] |
| OPB44688 | T4742_S00003.960 | purine and uridine phosphorylase [Trichoderma reesei RUT C-30] |
| OPB45148 | T4742_S00010.55 | purine and uridine phosphorylase [Trichoderma reesei RUT C-30] |
| OPB45523 | T4742_S00010.434 | purine and uridine phosphorylase [Trichoderma reesei RUT C-30] |
| OPB45794 | T4742_S00014.129 | purine and uridine phosphorylase [Trichoderma reesei RUT C-30] |
| OPB46636 | T4742_S00009.78 | purine and uridine phosphorylase [Trichoderma reesei RUT C-30] |
| OPB46687 | T4742_S00009.129 | purine and uridine phosphorylase [Trichoderma reesei RUT C-30] |
| OPB41226 | T4742_S00011.67 | Purple acid phosphatase |
| OPB45080 | T4742_S00013.385 | Putative 40S ribosomal protein S1 [Penicillium brasilianum] |
| OPB40789 | T4742_S00001.870 | putative aminotransferase [Neonectria ditissima] |
| OPB37153 | T4742_S00005.180 | putative intracellular serine protease protein [Trichoderma guizhouense] |
| OPB40964 | T4742_S00001.1045 | Putative Kinesin light chain 3 [Torrubiella hemipterigena] |
| OPB36396 | T4742_S00007.113 | putative nicotinamide N-methyltransferase [Trichoderma guizhouense] |
| OPB43655 | T4742_S00016.175 | putative RING finger protein, STAF5 |
| OPB43202 | T4742_S00022.5 | putative transcriptional regulator (Cti6) [Ustilaginoidea virens] |
| OPB40723 | T4742_S00001.803 | putative trihydroxytoluene oxygenase protein [Phaeoacremonium minimum UCRPA7] |
| OPB39985 | T4742_S00001.64 | Pyridine nucleotide-disulfide oxidoreductase, FAD/NAD(P)-binding domain protein [Aschersonia aleyrodis RCEF 2490] |
| OPB36322 | T4742_S00007.39 | Pyridine nucleotide-disulphide oxidoreductase, |
| OPB36445 | T4742_S00007.162 | Pyridine nucleotide-disulphide oxidoreductase, |
| OPB36733 | T4742_S00007.451 | Pyridine nucleotide-disulphide oxidoreductase, |
| OPB43223 | T4742_S00022.26 | Pyridine nucleotide-disulphide oxidoreductase, |
| OPB46507 | T4742_S00008.574 | Pyridine nucleotide-disulphide oxidoreductase, |
| OPB41035 | T4742_S00020.59 | Pyridoxal dependent decarboxylase |
| OPB39863 | T4742_S00015.207 | pyridoxal phosphate dependent enzymes class III |
| OPB44937 | T4742_S00013.242 | pyridoxal phosphate-dependent transferase, major domain-containing protein [Pochonia chlamydosporia 170] |
| OPB41578 | T4742_S00011.421 | pyridoxamine 5'-phosphate oxidase |
| OPB39772 | T4742_S00015.116 | pyridoxamine phosphate oxidase family protein |
| OPB47056 | T4742_S00009.498 | pyridoxine biosynthesis protein PDX1 |
| OPB38455 | T4742_S00002.503 | pyrimidine 5'-nucleotidase, putative |
| OPB46463 | T4742_S00008.530 | pyrophosphatase |
| OPB43407 | T4742_S00019.140 | pyrroline-5-carboxylate reductase |
| OPB42949 | T4742_S00012.186 | pyruvate carboxylase (cytosolic) |
| OPB36936 | T4742_S00007.654 | pyruvate decarboxylase |
| OPB38751 | T4742_S00002.800 | pyruvate decarboxylase |
| OPB43758 | T4742_S00003.29 | pyruvate decarboxylase |

| | | |
|---|---|---|
| OPB45520 | T4742_S00010.431 | pyruvate decarboxylase |
| OPB40007 | T4742_S00001.86 | pyruvate dehydrogenase kinase, putative |
| OPB41501 | T4742_S00011.344 | pyruvate dehydrogenase, E1 component alpha subunit |
| OPB45445 | T4742_S00010.356 | pyruvate formate lyase activating enzyme (radical SAM superfamily) |
| OPB44679 | T4742_S00003.951 | pyruvate kinase |
| OPB39081 | T4742_S00006.140 | QDE1, RdRP, essential for quelling |
| OPB37588 | T4742_S00005.615 | QDE-2 - post-transcriptional gene silencing protein QDE-2 |
| OPB42067 | T4742_S00004.169 | QDE3, RecQ helicase, essential for quelling |
| OPB46155 | T4742_S00008.222 | QI74 orthologue |
| OPB39421 | T4742_S00006.481 | QIP, Putative exonuclease protein, involved in quelling |
| OPB45361 | T4742_S00010.272 | queuine tRNA-ribosyltransferase. |
| OPB40014 | T4742_S00001.93 | Queuine/other tRNA-ribosyltransferase |
| OPB41479 | T4742_S00011.322 | quinate permease |
| OPB45380 | T4742_S00010.291 | quinone oxidoreductase |
| OPB40545 | T4742_S00001.624 | quinone oxidoreductase, putative [Colletotrichum orbiculare MAFF 240422] |
| OPB42748 | T4742_S00004.854 | Quinoprotein amine dehydrogenase beta chain-like |
| OPB43801 | T4742_S00003.72 | Quinoprotein amine dehydrogenase beta chain-like |
| OPB44861 | T4742_S00013.166 | Quinoprotein amine dehydrogenase beta chain-like |
| OPB38470 | T4742_S00002.518 | Rab family GTPase activating protein |
| OPB42703 | T4742_S00004.809 | Rab geranyl transferase escort protein |
| OPB43197 | T4742_S00012.434 | Rab GTPase activator |
| OPB45214 | T4742_S00010.122 | Rab GTPase activator |
| OPB37522 | T4742_S00005.549 | Rab GTPase interacting factor Yip1 |
| OPB44247 | T4742_S00003.518 | Rad1/Rec1/Rad17  repair protein |
| OPB36012 | T4742_S00023.47 | Rad10 endonuclease, nucleotide excision repair and recombinational repair. |
| OPB45701 | T4742_S00014.35 | Rad14, binsd to damaged DNA during nucleotide excisionRepair |
| OPB37241 | T4742_S00005.268 | Rad16 |
| OPB45218 | T4742_S00010.127 | Rad21/Rec8 like protein, C-terminal |
| OPB46863 | T4742_S00009.305 | Rad24, involved inReplication checkpoint control |
| OPB36758 | T4742_S00007.476 | Rad27, DNA structure-specific endonucleases involved in Okazaki fragment maturation |
| OPB47035 | T4742_S00009.477 | RAD28 |
| OPB41487 | T4742_S00011.330 | RAD2-like Flap structure-specific endonuclease [Colletotrichum tofieldiae] |
| OPB40866 | T4742_S00001.947 | Rad4/Cut5,  involved in DNA replication initiation and checkpoint control and DNA damage checkpoint response. |
| OPB43319 | T4742_S00019.52 | Rad4p |
| OPB36560 | T4742_S00007.277 | Rad4p,  involved in nucleotide excisionRepair. |
| OPB44425 | T4742_S00003.697 | RAD50 DNA-repair protein |

| | | |
|---|---|---|
| OPB37117 | T4742_S00005.144 | Rad52, DNA repair |
| OPB42008 | T4742_S00004.110 | Rad54-like |
| OPB38362 | T4742_S00002.410 | rad55 |
| OPB40870 | T4742_S00001.951 | Rad5p ubiquitin-protein ligase |
| OPB38288 | T4742_S00002.335 | Rad9 |
| OPB37293 | T4742_S00005.320 | Rad9 protein, DNA damage sensor. |
| OPB40961 | T4742_S00001.1042 | Rad9, that functions in DNA repair |
| OPB40152 | T4742_S00001.231 | RAI1 decapping nulease |
| OPB39887 | T4742_S00015.231 | ran binding protein in the microtubule-organising centre |
| OPB36963 | T4742_S00007.681 | ran-interacting Mog1 protein |
| OPB41085 | T4742_S00020.109 | Ran-specific GTPase-activating protein 1, putative |
| OPB40067 | T4742_S00001.146 | Rap-RAN GTPase activating protein tuberin |
| OPB46208 | T4742_S00008.275 | Ras GTPase |
| OPB46561 | T4742_S00009.1 | Ras GTPase-activating protein |
| OPB38282 | T4742_S00002.329 | RAS like small GTPase, Ras subfamily |
| OPB38593 | T4742_S00002.642 | RAS small GTPase, Rac subfamily |
| OPB37591 | T4742_S00005.618 | Ras small GTPase, Rho Type |
| OPB36694 | T4742_S00007.412 | RAS small GTPase,Ras subfamily |
| OPB43605 | T4742_S00016.125 | Ras small GTPase,Rho type |
| OPB43522 | T4742_S00016.42 | RAS1 |
| OPB44137 | T4742_S00003.408 | Ras2 |
| OPB36671 | T4742_S00007.389 | RAS-GTPase activating protein (RasGAP) |
| OPB45710 | T4742_S00014.44 | Ras-like GTPase, Rho type |
| OPB43922 | T4742_S00003.193 | recombination hotspot-binding protein (Translin) |
| OPB39292 | T4742_S00006.352 | RecQ subfamily of DNA helicases |
| OPB40395 | T4742_S00001.474 | redoxin domain protein |
| OPB43743 | T4742_S00003.14 | reductase [Coniochaeta ligniaria NRRL 30616] |
| OPB43495 | T4742_S00016.15 | Regulation of HR towards crossover |
| OPB47037 | T4742_S00009.479 | Regulation of HR towards crossover |
| OPB42037 | T4742_S00004.139 | Regulator of G protein signalling |
| OPB41078 | T4742_S00020.102 | regulator of MAPKKK STE50 |
| OPB38997 | T4742_S00006.56 | Regulator of Rac1, required for phagocytosis and cell migration |
| OPB36414 | T4742_S00007.131 | Regulatory protein involved in catabolite repression [Trichoderma guizhouense] |
| OPB43120 | T4742_S00012.357 | related to anaphase promoting complex subunit 10 [Fusarium proliferatum] |
| OPB36121 | T4742_S00021.38 | related to heterokaryon incompatibility protein het-6 [Fusarium proliferatum ET1] |
| OPB46567 | T4742_S00009.7 | related to transcription initiation factor TFIIIC [Fusarium fujikuroi] |

| | | |
|---|---|---|
| OPB40260 | T4742_S00001.339 | replication factor A, large subunit |
| OPB37006 | T4742_S00005.33 | Replication factor C subunit 4. This subunit may be involved in the elongation of the multiprimed DNA template. |
| OPB38787 | T4742_S00002.836 | replication fork protection component Swi3 |
| OPB38348 | T4742_S00002.396 | Replication protein A2 |
| OPB41906 | T4742_S00004.8 | Respiratory-chain NADH dehydrogenase, 51 kDa subunit |
| OPB38780 | T4742_S00002.829 | response regulator receiver |
| OPB44493 | T4742_S00003.765 | retinol dehydrogenase |
| OPB45853 | T4742_S00014.188 | retinol dehydrogenase |
| OPB45264 | T4742_S00010.173 | retrograde regulation protein 2 |
| OPB46856 | T4742_S00009.298 | retrograde regulation protein 2 |
| OPB46802 | T4742_S00009.244 | REV1 deoxycytidyl transferase involved in DNA translesion synthesis |
| OPB38557 | T4742_S00002.606 | Rev3p,  catalytic subunit of DNA polymerase zeta |
| OPB42462 | T4742_S00004.564 | Rfc3 of Saccharomyces cerevisiae. |
| OPB43161 | T4742_S00012.398 | Rfc3p of Saccharomyces cerevisiae. |
| OPB40118 | T4742_S00001.197 | Rfc4p (replication factor C) subunit 4 |
| OPB43209 | T4742_S00022.12 | RgsA, regulator of G-protein signaling |
| OPB37855 | T4742_S00017.122 | RgsB, regulator of G-protein signaling |
| OPB41951 | T4742_S00004.53 | RgsC, regulator of G-protein signaling |
| OPB41952 | T4742_S00004.54 | RgsC, regulator of G-protein signaling |
| OPB45085 | T4742_S00013.390 | RheB GTPase,Ras small GTPase,Ras-type,Related to Aspergillus fumigatusRheb GTPaseRhbA |
| OPB38697 | T4742_S00002.746 | Rho GTPase-activating protein involved in signal transduction mechanisms |
| OPB43293 | T4742_S00019.26 | RHO protein GDP dissociation inhibitor |
| OPB46549 | T4742_S00008.616 | RHO, guanyl nucleotide exchange factor |
| OPB42592 | T4742_S00004.696 | rho2 |
| OPB41311 | T4742_S00011.152 | rho3 |
| OPB42506 | T4742_S00004.608 | rho4 |
| OPB43635 | T4742_S00016.155 | RhoA GTPase effector arrestin |
| OPB46180 | T4742_S00008.247 | rhodanese-like domain-containing protein |
| OPB37621 | T4742_S00005.648 | RhoGAP |
| OPB41596 | T4742_S00011.439 | RhoGEF |
| OPB42913 | T4742_S00012.150 | RhoGEF |
| OPB43322 | T4742_S00019.55 | RhoGEF domain-containing protein |
| OPB37321 | T4742_S00005.348 | Rho-GTPase effector BNI1 |
| OPB41662 | T4742_S00011.505 | Rho-guanyl nucleotide exchange factor |
| OPB38266 | T4742_S00002.313 | Rho-type GTPase-activating protein |
| OPB39739 | T4742_S00015.83 | ribitol kinase |

| | | |
|---|---|---|
| OPB38416 | T4742_S00002.464 | riboflavin biosynthesis protein Rib7 |
| OPB36361 | T4742_S00007.78 | Riboflavin kinase / FAD synthetase |
| OPB43660 | T4742_S00016.180 | riboflavin synthase |
| OPB37966 | T4742_S00002.13 | Ribokinase |
| OPB38417 | T4742_S00002.465 | ribokinase |
| OPB40249 | T4742_S00001.328 | ribokinase |
| OPB40048 | T4742_S00001.127 | Ribokinase-like protein, partial [Trichoderma reesei RUT C-30] |
| OPB41157 | T4742_S00020.181 | ribonuclease |
| OPB43439 | T4742_S00019.172 | ribonuclease |
| OPB45126 | T4742_S00010.33 | Ribonuclease CAF1 |
| OPB39756 | T4742_S00015.100 | ribonuclease HI (RNaseH). |
| OPB39849 | T4742_S00015.193 | ribonuclease HII |
| OPB36981 | T4742_S00005.8 | Ribonuclease II |
| OPB38746 | T4742_S00002.795 | ribonuclease T1 |
| OPB38039 | T4742_S00002.86 | ribonuclease T2 |
| OPB38452 | T4742_S00002.500 | Ribonuclease, T2 family |
| OPB46277 | T4742_S00008.344 | Ribonuclease/ribotoxin |
| OPB38692 | T4742_S00002.741 | Ribonucleases P/MRP protein subunit POP1 containing protein |
| OPB46062 | T4742_S00008.129 | ribonucleotide reductase |
| OPB45328 | T4742_S00010.239 | ribonucleotide reductase inhibitor |
| OPB42184 | T4742_S00004.286 | Ribonucleotide reductase large subunit |
| OPB40387 | T4742_S00001.466 | Ribose/galactose isomerase |
| OPB39882 | T4742_S00015.226 | ribose-5-phosphate isomerase |
| OPB42988 | T4742_S00012.225 | Ribosomal biogenesis regulatory protein RRS1 |
| OPB46748 | T4742_S00009.190 | Ribosomal protein (60S) L11 |
| OPB43022 | T4742_S00012.259 | ribosomal protein (60S) P0 |
| OPB45531 | T4742_S00010.442 | ribosomal protein L1. |
| OPB43100 | T4742_S00012.337 | Ribosomal protein L10. |
| OPB44095 | T4742_S00003.366 | ribosomal protein L10e. |
| OPB38668 | T4742_S00002.717 | ribosomal protein L11 |
| OPB41604 | T4742_S00011.447 | ribosomal protein L14. |
| OPB39118 | T4742_S00006.178 | Ribosomal protein L14b |
| OPB37798 | T4742_S00017.65 | ribosomal protein L14b/L23e. |
| OPB40893 | T4742_S00001.974 | ribosomal protein L15 |
| OPB40894 | T4742_S00001.975 | ribosomal protein L15 |
| OPB42461 | T4742_S00004.563 | ribosomal protein L16 |

| | | |
|---|---|---|
| OPB40194 | T4742_S00001.273 | Ribosomal protein L17 |
| OPB45041 | T4742_S00013.346 | Ribosomal protein L17 |
| OPB36027 | T4742_S00023.62 | Ribosomal protein L18. |
| OPB37882 | T4742_S00017.149 | ribosomal protein L18ae |
| OPB39326 | T4742_S00006.386 | ribosomal protein L19 |
| OPB44109 | T4742_S00003.380 | ribosomal protein L22. |
| OPB41915 | T4742_S00004.17 | ribosomal protein L22/17 |
| OPB40974 | T4742_S00001.1055 | ribosomal protein L23 |
| OPB42030 | T4742_S00004.132 | ribosomal protein L23 |
| OPB39873 | T4742_S00015.217 | ribosomal protein L24e, putative |
| OPB37185 | T4742_S00005.212 | ribosomal protein L27 domain. Shows amino acid sequence similarity to S. cerevisiae mitochondrial ribosomal protein MRP7. |
| OPB43192 | T4742_S00012.429 | ribosomal protein L27e. Homologue of yeast RPL27a/b. |
| OPB44566 | T4742_S00003.838 | Ribosomal protein L28 (L29/L15, rp44, YL24 family). Homologue of S. cerevisiae RPL28. |
| OPB38440 | T4742_S00002.488 | ribosomal protein L29. Shows amino acid sequence similarity to S. cerevisiae mitochondrial ribosomal protein MRPL4. |
| OPB39719 | T4742_S00015.63 | ribosomal protein L3 |
| OPB43148 | T4742_S00012.385 | Ribosomal protein L30. |
| OPB37854 | T4742_S00017.121 | ribosomal protein L31e. |
| OPB41097 | T4742_S00020.121 | Ribosomal protein L32 based on homology to the corresponding protein in N. crassa. |
| OPB44292 | T4742_S00003.563 | ribosomal protein L36 |
| OPB39440 | T4742_S00006.500 | ribosomal protein L37 of the large (60S) ribosomal subunit. Belongs to the same family as yeast Rpl33Ap and has similarity to rat L35a. |
| OPB38489 | T4742_S00002.538 | ribosomal protein L37e from several filamentous fungi and yeasts. |
| OPB37614 | T4742_S00005.641 | ribosomal protein L4/L1e |
| OPB42307 | T4742_S00004.409 | Ribosomal protein L43A (L37 family) by homology to the corresponding protein of Candida albicans. |
| OPB36713 | T4742_S00007.431 | Ribosomal protein L49/IMG2 |
| OPB43623 | T4742_S00016.143 | Ribosomal protein L5 |
| OPB40138 | T4742_S00001.217 | Ribosomal protein L5 domain. |
| OPB41990 | T4742_S00004.92 | ribosomal protein L6 |
| OPB39361 | T4742_S00006.421 | ribosomal protein L7/12 domain. Shares amino acid sequence similarity to S. cerevisiae mitochondrial ribosomal protein MRPL12. |
| OPB43985 | T4742_S00003.256 | ribosomal protein L7AE domain. |
| OPB36009 | T4742_S00023.44 | Ribosomal protein L7Ae/L30e/S12e/Gadd45. Homologue of yeast SNU13. |
| OPB36542 | T4742_S00007.259 | Ribosomal protein L8 |
| OPB43939 | T4742_S00003.210 | ribosomal protein MRPL20. |
| OPB43518 | T4742_S00016.38 | ribosomal protein MRPL24. |
| OPB37615 | T4742_S00005.642 | ribosomal protein MRPL35. |
| OPB43665 | T4742_S00016.185 | ribosomal protein MRPL49. |
| OPB45249 | T4742_S00010.158 | ribosomal protein RSM22. |

| | | |
|---|---|---|
| OPB46568 | T4742_S00009.8 | Ribosomal protein S10 |
| OPB36707 | T4742_S00007.425 | ribosomal protein S10. |
| OPB40475 | T4742_S00001.554 | ribosomal protein S11 |
| OPB42341 | T4742_S00004.443 | Ribosomal protein S12 |
| OPB42925 | T4742_S00012.162 | Ribosomal protein S13 (S15 family) based on homology to the corresponding protein in Gibberella zeae. |
| OPB41263 | T4742_S00011.104 | ribosomal protein S14 |
| OPB43124 | T4742_S00012.361 | Ribosomal protein S15. |
| OPB45363 | T4742_S00010.274 | ribosomal protein S17e, and it shares extensive amino acid sequence identity with ribosomal protein S17e orthologues from other filamentous fungi and yeasts. |
| OPB38958 | T4742_S00006.17 | Ribosomal protein S18, S13 family |
| OPB44057 | T4742_S00003.328 | Ribosomal protein S18. |
| OPB37144 | T4742_S00005.171 | ribosomal protein S2 and tRNA/rRNA methyltransferase. |
| OPB42628 | T4742_S00004.733 | ribosomal protein S2. |
| OPB43556 | T4742_S00016.76 | ribosomal protein S2. |
| OPB39653 | T4742_S00006.713 | Ribosomal protein S23 |
| OPB43823 | T4742_S00003.94 | ribosomal protein S24e. Homologue of yeast RPS24a/b. |
| OPB45329 | T4742_S00010.240 | Ribosomal protein S27 based on homology to the corresponding protein of N. crassa. |
| OPB38696 | T4742_S00002.745 | Ribosomal protein S28 based on homology to the corresponding protein of Neurospora crassa. |
| OPB40087 | T4742_S00001.166 | Ribosomal protein S29 (S14 family) by homology to the corresponding protein of N. crassa. |
| OPB44166 | T4742_S00003.437 | Ribosomal protein S30 by homology with the corresponding protein of Ashbya gossypii. |
| OPB38041 | T4742_S00002.88 | ribosomal protein S4. |
| OPB44058 | T4742_S00003.329 | ribosomal protein S4. |
| OPB37982 | T4742_S00002.29 | ribosomal protein S40 |
| OPB39818 | T4742_S00015.162 | ribosomal protein S5, MRPS5. |
| OPB43169 | T4742_S00012.406 | Ribosomal protein S5, S7 family |
| OPB36384 | T4742_S00007.101 | Ribosomal protein S7 |
| OPB39045 | T4742_S00006.104 | Ribosomal protein S7 |
| OPB37915 | T4742_S00017.182 | ribosomal protein S8, MRPS8. |
| OPB39997 | T4742_S00001.76 | Ribosomal protein S9 |
| OPB38555 | T4742_S00002.604 | Ribosomal protein S9, S4 family |
| OPB43154 | T4742_S00012.391 | ribosomal protein YmL32 precursor. Contains possible InterPro domain for ribosomal L32p proteins. |
| OPB39825 | T4742_S00015.169 | Ribosomal protein(60S)  L152/L15B |
| OPB44286 | T4742_S00003.557 | ribosomal recycling factor [Purpureocillium lilacinum] |
| OPB44091 | T4742_S00003.362 | ribosomal RNA adenine methylase transferase. |
| OPB36026 | T4742_S00023.61 | ribosomal RNA methyltransferase RrmJ/FtsJ domain. |
| OPB37499 | T4742_S00005.526 | ribosomal RNA methyltransferase RrmJ/FtsJ domain. |
| OPB41961 | T4742_S00004.63 | RibosomalRNA methyltransferaseRrmJ/FtsJ domain. |

| | | |
|---|---|---|
| OPB44804 | T4742_S00013.109 | Ribosome biogenesis factor, NIP7 |
| OPB40101 | T4742_S00001.180 | ribosome biogenesis GTPase Lsg1 |
| OPB44408 | T4742_S00003.679 | ribosome biogenesis protein Pescadillo, putative |
| OPB42638 | T4742_S00004.743 | ribosome biogenesis protein Ssf2, putative |
| OPB37990 | T4742_S00002.37 | ribosome biogenesis protein Urb1 , putative |
| OPB46896 | T4742_S00009.338 | ribulose-phosphate 3-epimerase |
| OPB38686 | T4742_S00002.735 | RID1 |
| OPB38685 | T4742_S00002.734 | RID1 protein [Trichoderma guizhouense] |
| OPB43320 | T4742_S00019.53 | ring finger protein |
| OPB46134 | T4742_S00008.201 | RING finger protein |
| OPB36673 | T4742_S00007.391 | RING-5 |
| OPB38491 | T4742_S00002.540 | RING-type zinc finger protein |
| OPB41824 | T4742_S00018.147 | RluA family pseudouridine synthase |
| OPB44420 | T4742_S00003.692 | RMD5, regulator of gluconeogenesis |
| OPB45583 | T4742_S00010.494 | RmlC-like cupin, secreted |
| OPB39847 | T4742_S00015.191 | RNA 12 protein |
| OPB46769 | T4742_S00009.211 | RNA 3'-terminal phosphate cyclase |
| OPB45320 | T4742_S00010.231 | RNA 3'-terminal phosphate cyclase , putative |
| OPB42588 | T4742_S00004.692 | RNA binding domain protein PUA |
| OPB40148 | T4742_S00001.227 | RNA binding protein |
| OPB46875 | T4742_S00009.317 | RNA binding protein |
| OPB38273 | T4742_S00002.320 | RNA binding protein MSSP-2 |
| OPB37114 | T4742_S00005.141 | RNA binding protein Rnp24 |
| OPB37115 | T4742_S00005.142 | RNA binding protein RNP24 |
| OPB42973 | T4742_S00012.210 | RNA cap guanine-N2 methyltransferase, putative |
| OPB39893 | T4742_S00015.237 | RNA exonuclease 4 |
| OPB37266 | T4742_S00005.293 | RNA export mediator Gle1, putative |
| OPB37088 | T4742_S00005.115 | RNA helicase nonsense mRNA reducing factor (pNORF1) |
| OPB37017 | T4742_S00005.44 | RNA helicase, ATP-dependent, DEAD-box, conserved site |
| OPB36760 | T4742_S00007.478 | RNA helicases and translation initiation factor eIF4a subunit. Homologue of yeast TIF2. |
| OPB47071 | T4742_S00009.513 | RNA polymerase H/23 kD subunit |
| OPB45105 | T4742_S00010.12 | RNA polymerase I specific transcription stimulatory factor composed of Uaf30p |
| OPB43876 | T4742_S00003.147 | RNA polymerase I subunit Rpa43, putative |
| OPB40143 | T4742_S00001.222 | RNA polymerase II Elongator subunit |
| OPB44701 | T4742_S00013.5 | RNA polymerase II mediator complex component [Colletotrichum tofieldiae] |
| OPB44378 | T4742_S00003.649 | RNA polymerase II subunit A C-terminal domain phosphatase ssu-72 |

| | | |
|---|---|---|
| OPB42238 | T4742_S00004.340 | RNA polymerase II transcription elongation factor (Ctr9) |
| OPB41677 | T4742_S00011.520 | RNA polymerase II transcription factor B subunit |
| OPB36633 | T4742_S00007.351 | RNA polymerase II transcription factor B subunit 2 |
| OPB44106 | T4742_S00003.377 | RNA polymerase II transcription factor B subunit 5 |
| OPB38454 | T4742_S00002.502 | RNA polymerase II transcription mediator |
| OPB37560 | T4742_S00005.587 | RNA polymerase II transcription mediator complex subunit (Med6), putative |
| OPB37485 | T4742_S00005.512 | RNA polymerase II transcriptional coactivator, putative |
| OPB42032 | T4742_S00004.134 | RNA polymerase II-associated protein |
| OPB42338 | T4742_S00004.440 | RNA polymerase II-associated protein. |
| OPB40034 | T4742_S00001.113 | RNA polymerase III RPC4. |
| OPB41441 | T4742_S00011.284 | RNA polymerase III subunitRpc25 |
| OPB42258 | T4742_S00004.360 | RNA polymerase III transcription initiation factor complex (TFIIIC) subunit part of the TauA globular domain |
| OPB44320 | T4742_S00003.591 | RNA polymerase N/8 kDa subunit |
| OPB44357 | T4742_S00003.628 | RNA polymerase Rpb1 |
| OPB46575 | T4742_S00009.15 | RNA polymerase Rpb1 |
| OPB38310 | T4742_S00002.357 | RNA polymerase Rpb5 |
| OPB43041 | T4742_S00012.278 | RNA polymerase Rpb6 |
| OPB39911 | T4742_S00015.255 | RNA polymerase Rpb8 |
| OPB37767 | T4742_S00017.34 | RNA polymerase Rpc34 subunit |
| OPB43189 | T4742_S00012.426 | RNA polymerase TFIIH complex subunit Ssl1, putative |
| OPB39252 | T4742_S00006.312 | RNA recognition domain protein |
| OPB42412 | T4742_S00004.514 | RNA recognition domain protein |
| OPB43612 | T4742_S00016.132 | RNA recognition domain protein |
| OPB41904 | T4742_S00004.6 | RNA recognition domain-containing protein |
| OPB36941 | T4742_S00007.659 | RNA recognition domain-containing protein |
| OPB44167 | T4742_S00003.438 | RNA recognition domain-containing protein |
| OPB38546 | T4742_S00002.595 | RNA recognition motif, RNP-1 |
| OPB38629 | T4742_S00002.678 | RNA recognition motif, RNP-1 |
| OPB42376 | T4742_S00004.478 | RNA recognition motif, RNP-1 |
| OPB45072 | T4742_S00013.377 | RNA recognition motif, RNP-1 |
| OPB45305 | T4742_S00010.216 | RNA recognition motif, RNP-1 |
| OPB43618 | T4742_S00016.138 | RNA recognition protein, RNP-1 |
| OPB42264 | T4742_S00004.366 | RNA:NAD 2'-phosphotransferase TPT1. |
| OPB45465 | T4742_S00010.376 | RNA-binding ATP-dependent helicases. |
| OPB37996 | T4742_S00002.43 | RNA-binding La domain protein |
| OPB36578 | T4742_S00007.295 | RNA-binding protein Vip1 |

| | | |
|---|---|---|
| OPB42269 | T4742_S00004.371 | RNA-binding protein with KH domain |
| OPB46781 | T4742_S00009.223 | RNA-binding protein with KH domain |
| OPB45943 | T4742_S00008.9 | RNA-binding protein, Crucial for commitment to meiosis |
| OPB41494 | T4742_S00011.337 | RNA-binding, negative regulator of differentiation 1 |
| OPB40182 | T4742_S00001.261 | RNA-binding,Ran Zn-finger protein, probably involved inRegulation ofReceptor mediated transport between the nucleus and the cytoplasm |
| OPB38502 | T4742_S00002.551 | RNA-dependent RNA polymerase SAD-1 |
| OPB45674 | T4742_S00014.8 | RNA-dependent RNA-polymerase |
| OPB41913 | T4742_S00004.15 | RNA-processing protein, HAT helix |
| OPB37180 | T4742_S00005.207 | RNAse III domain protein |
| OPB38486 | T4742_S00002.534 | RNAse P, Rpr2/Rpp21 subunit |
| OPB42407 | T4742_S00004.509 | RNase3 domain-containing protein |
| OPB39813 | T4742_S00015.157 | Rpc82, DNA-directed RNA polymerase III subunit |
| OPB43728 | T4742_S00016.248 | RPF2, involved in the processing of pre-rRNA |
| OPB38010 | T4742_S00002.57 | Rpp14 family protein |
| OPB41098 | T4742_S00020.122 | Rps16 gene encoding protein component of the small (40S)Ribosomal subunit (S9 family). |
| OPB45270 | T4742_S00010.181 | Rps24 (family S16) |
| OPB43664 | T4742_S00016.184 | Rps8, resistance to killer toxin |
| OPB45616 | T4742_S00010.527 | RraA-like protein |
| OPB41018 | T4742_S00020.42 | RRB1, involved in early steps of Ribosome biogenesis. |
| OPB42966 | T4742_S00012.203 | RRN3, required for the transcription of rDNA |
| OPB38669 | T4742_S00002.718 | rRNA assembly protein Mis3 |
| OPB46708 | T4742_S00009.150 | rRNA biogenesis protein RRP5 |
| OPB39930 | T4742_S00001.9 | rRNA methyltransferase |
| OPB42039 | T4742_S00004.141 | rRNA processing protein Bystin |
| OPB45093 | T4742_S00013.398 | rRNA processing protein EBP2 |
| OPB38434 | T4742_S00002.482 | rRNA processing protein Rrp8 |
| OPB40119 | T4742_S00001.198 | rRNA-processing protein FCF1 |
| OPB40176 | T4742_S00001.255 | RRP9, encoding a protein involved in pre-rRNA processing, associated with U3 snRNP; component of SSU processosome. |
| OPB37312 | T4742_S00005.339 | R-SNARE Sec22; anterograde/retrograde transport ER-Golgi |
| OPB37514 | T4742_S00005.541 | RUM1 repressor [Trichoderma guizhouense] |
| OPB37466 | T4742_S00005.493 | S-(hydroxymethyl)glutathione dehydrogenase |
| OPB45479 | T4742_S00010.390 | S-(hydroxymethyl)glutathione dehydrogenase |
| OPB40129 | T4742_S00001.208 | S1, RNA binding |
| OPB35953 | T4742_S00025.26 | S1/P1 Nuclease |
| OPB46372 | T4742_S00008.439 | S1/P1 nuclease |
| OPB40312 | T4742_S00001.391 | S8 Proteinase inhibitor I10 |

| | | |
|---|---|---|
| OPB37986 | T4742_S00002.33 | S8 Proteinase inhibitor I9 |
| OPB42555 | T4742_S00004.658 | saccharopine dehydrogenase |
| OPB45410 | T4742_S00010.321 | Saccharopine dehydrogenase |
| OPB45767 | T4742_S00014.102 | Saccharopine dehydrogenase |
| OPB44489 | T4742_S00003.761 | saccharopine dehydrogenase [Metarhizium guizhouense ARSEF 977] |
| OPB45942 | T4742_S00008.8 | Sad1/UNC domain-containing protein [Colletotrichum nymphaeae SA-01] |
| OPB41419 | T4742_S00011.262 | S-adenosyl-L-homocysteine hydrolase |
| OPB37491 | T4742_S00005.518 | S-adenosyl-L-methionine-dependent methyltransferase [Trichoderma reesei RUT C-30] |
| OPB37673 | T4742_S00005.700 | S-adenosylmethionine decarboxylase |
| OPB39232 | T4742_S00006.292 | S-adenosylmethionine synthetase |
| OPB36651 | T4742_S00007.369 | SAGA complex bromodomain subunit Spt7, putative |
| OPB45405 | T4742_S00010.316 | SAGA complex subunit (Ada2) |
| OPB45955 | T4742_S00008.21 | SAGA-associated factor 73 [Fusarium oxysporum f. sp. cubense race 1] |
| OPB42110 | T4742_S00004.212 | SAICAR synthase |
| OPB44413 | T4742_S00003.684 | salicylaldehyde dehydrogenase [Diaporthe helianthi] |
| OPB36811 | T4742_S00007.529 | SAM dependent methyltransferase |
| OPB44064 | T4742_S00003.335 | SAM dependent methyltransferase |
| OPB47003 | T4742_S00009.445 | SAM dependent methyltransferase |
| OPB42512 | T4742_S00004.614 | SAM methyltransferase TRM9 (tRNA carboxylmethyl transferase). |
| OPB39775 | T4742_S00015.119 | SAM methyltransferase, TRM12 |
| OPB37278 | T4742_S00005.305 | SAM methyltransferase, tRNA-methylztransferase subunit GCD14 (yeast). |
| OPB45835 | T4742_S00014.170 | SAM-dependent methylransferase |
| OPB36090 | T4742_S00021.6 | SAM-dependent methyltransferase |
| OPB36615 | T4742_S00007.333 | SAM-dependent methyltransferase |
| OPB37476 | T4742_S00005.503 | SAM-dependent methyltransferase |
| OPB41644 | T4742_S00011.487 | SAM-dependent methyltransferase |
| OPB44020 | T4742_S00003.291 | SAM-dependent methyltransferase |
| OPB45391 | T4742_S00010.302 | SAM-dependent methyltransferase |
| OPB45650 | T4742_S00010.561 | SAM-dependent methyltransferase |
| OPB46125 | T4742_S00008.192 | SAM-dependent methyltransferase |
| OPB46532 | T4742_S00008.599 | SAM-dependent methyltransferase |
| OPB42932 | T4742_S00012.169 | SAM-dependent methyltransferase MTQ2 |
| OPB46732 | T4742_S00009.174 | SAM-dependent methyltransferase, partial [Trichoderma reesei RUT C-30] |
| OPB42288 | T4742_S00004.390 | SAM-dependent methyltransfersae with NOL1/NOP2/sun domain |
| OPB36446 | T4742_S00007.163 | SAM-dependent metyhltransferase |
| OPB36627 | T4742_S00007.345 | SAM-dependent metyhltransferase |

| | | |
|---|---|---|
| OPB38538 | T4742_S00002.587 | SAM-dependent metyhltransferase |
| OPB38675 | T4742_S00002.724 | SAM-dependent metyhltransferase |
| OPB38793 | T4742_S00002.842 | SAM-dependent metyhltransferase |
| OPB39266 | T4742_S00006.326 | SAM-dependent metyhltransferase |
| OPB40810 | T4742_S00001.891 | SAM-dependent metyhltransferase |
| OPB42672 | T4742_S00004.777 | SAM-dependent metyhltransferase |
| OPB44513 | T4742_S00003.785 | SAM-dependent metyhltransferase |
| OPB44616 | T4742_S00003.888 | SAM-dependent metyhltransferase |
| OPB46916 | T4742_S00009.358 | SAM-dependent metyhltransferase |
| OPB38058 | T4742_S00002.105 | SAP domain protein |
| OPB43078 | T4742_S00012.315 | Sar1 GTPase |
| OPB42637 | T4742_S00004.742 | sarcosine oxidase |
| OPB45578 | T4742_S00010.489 | sarcosine oxidase |
| OPB45139 | T4742_S00010.46 | SAS10, encoding a component of theSSU processosome required for pre-18S rRNa processing. |
| OPB42212 | T4742_S00004.314 | SCF complex subunit Skp1 |
| OPB38169 | T4742_S00002.216 | SCF ubiquitin ligase complex subunit, cullin CDC53 |
| OPB39041 | T4742_S00006.100 | SCON-2, E3-ubiquitin ligase LIMPET |
| OPB37447 | T4742_S00005.474 | SCP-2 sterol transfer family protein |
| OPB41372 | T4742_S00011.213 | scytalone dehydratase |
| OPB43924 | T4742_S00003.195 | Sec1 family member sly1 |
| OPB36752 | T4742_S00007.470 | SEC14, phosphatidylinositol/phosphatidylcholine transfer protein |
| OPB37871 | T4742_S00017.138 | Sec61 beta subunit |
| OPB45332 | T4742_S00010.243 | Sec61 gamma subunit |
| OPB37218 | T4742_S00005.245 | Sec61alpha subunit |
| OPB41565 | T4742_S00011.408 | Secretion related GTPase Rab5/Ypt51 |
| OPB44398 | T4742_S00003.669 | Secretion related GTPase Rab5/Ypt51 |
| OPB43571 | T4742_S00016.91 | Secretion related small GTPase Rab1/Ypt1 |
| OPB43214 | T4742_S00022.17 | Secretion related small GTPase Rab11/Ypt3 |
| OPB39725 | T4742_S00015.69 | Secretion related small GTPase Rab2 |
| OPB37809 | T4742_S00017.76 | Secretion related small GTPase Rab4 |
| OPB39883 | T4742_S00015.227 | Secretion related small GTPase Rab6/Ypt6/Ryh1 |
| OPB38195 | T4742_S00002.242 | Secretion related small GTPase RAB7 |
| OPB38664 | T4742_S00002.713 | Secretion related small GTPase Sec4 |
| OPB45074 | T4742_S00013.379 | Secretory pathway Sec39 |
| OPB39776 | T4742_S00015.120 | Secretory protein Ssp120, Ca2+ binding |
| OPB44993 | T4742_S00013.298 | sedoheptulose-1,7-bisphosphatase |

| | | |
|---|---|---|
| OPB40377 | T4742_S00001.456 | Selenocysteine lyase-like protein |
| OPB38684 | T4742_S00002.733 | SEN1 (tRNA splicing complex component). |
| OPB38945 | T4742_S00006.4 | Sensory transduction histidine kinase class V |
| OPB45307 | T4742_S00010.218 | septin-like protein |
| OPB44458 | T4742_S00003.730 | septum formation protein Maf |
| OPB45239 | T4742_S00010.148 | SerB Phosphoserine phosphatase |
| OPB37307 | T4742_S00005.334 | SerC Phosphoserine aminotransferase |
| OPB41108 | T4742_S00020.132 | Serine dehydratase |
| OPB36652 | T4742_S00007.370 | Serine hydroxymethyltransferase |
| OPB41910 | T4742_S00004.12 | Serine hydroxymethyltransferase |
| OPB37969 | T4742_S00002.16 | serine incorporator [Trichoderma guizhouense] |
| OPB36973 | T4742_S00007.691 | serine palmitoyl CoA transferase subunit-like protein |
| OPB41079 | T4742_S00020.103 | serine palmitoyl transferase, subunit LCBB |
| OPB39513 | T4742_S00006.573 | serine threonine protein kinase SNF1 |
| OPB41592 | T4742_S00011.435 | serine threonine protein phosphatase |
| OPB37935 | T4742_S00017.202 | serine/threonine protein kinase |
| OPB38254 | T4742_S00002.301 | serine/threonine protein kinase |
| OPB38374 | T4742_S00002.422 | serine/threonine protein kinase |
| OPB38667 | T4742_S00002.716 | serine/threonine protein kinase |
| OPB38816 | T4742_S00002.865 | serine/threonine protein kinase |
| OPB39857 | T4742_S00015.201 | serine/threonine protein kinase |
| OPB40485 | T4742_S00001.564 | serine/threonine protein kinase |
| OPB43019 | T4742_S00012.256 | serine/threonine protein kinase |
| OPB44004 | T4742_S00003.275 | serine/threonine protein kinase |
| OPB44437 | T4742_S00003.709 | serine/threonine protein kinase |
| OPB45136 | T4742_S00010.43 | serine/threonine protein kinase |
| OPB39447 | T4742_S00006.507 | serine/threonine protein kinase |
| OPB45723 | T4742_S00014.57 | serine/threonine protein kinase |
| OPB46619 | T4742_S00009.61 | serine/threonine protein kinase TPK2 |
| OPB45568 | T4742_S00010.479 | serine/threonine protein kinase containing Ca2+-dependent membrane-targeting module, related to S. cerevisiae ypk1p, which is involved in spingolipid-mediated and cell integrity |
| OPB36985 | T4742_S00005.12 | serine/threonine protein kinase cot1 |
| OPB41943 | T4742_S00004.45 | serine/threonine protein kinase kin1 involved in cellular morphogenesis |
| OPB43263 | T4742_S00022.67 | serine/threonine protein kinase nrc2 |
| OPB42660 | T4742_S00004.765 | serine/threonine protein kinase PRP4 |
| OPB37817 | T4742_S00017.84 | Serine/threonine protein kinase required for receptor-mediated endocytosis |
| OPB46079 | T4742_S00008.146 | serine/threonine protein kinase YAK1 |

| | | |
|---|---|---|
| OPB45143 | T4742_S00010.50 | serine/threonine protein kinase, |
| OPB38257 | T4742_S00002.304 | serine/threonine protein kinase, PAK/STE20 subfamily |
| OPB38018 | T4742_S00002.65 | Serine/threonine protein kinase, vps15, involved in vacuolar protein sorting |
| OPB38138 | T4742_S00002.185 | serine/threonine protein phosphatase |
| OPB44284 | T4742_S00003.555 | serine/threonine protein phosphatase |
| OPB39989 | T4742_S00001.68 | serine/threonine protein phosphatase type 5 |
| OPB41853 | T4742_S00018.176 | serine/threonine-protein kinase |
| OPB43272 | T4742_S00019.5 | serine/threonine-protein kinase |
| OPB43499 | T4742_S00016.19 | serine/threonine-protein kinase |
| OPB43666 | T4742_S00016.186 | serine/threonine-protein kinase |
| OPB44326 | T4742_S00003.597 | serine/threonine-protein kinase chk2 |
| OPB42229 | T4742_S00004.331 | serine/threonine-protein kinase hal4 |
| OPB39079 | T4742_S00006.138 | serine/threonine-protein kinase RIO1 |
| OPB37564 | T4742_S00005.591 | serine/threonine-protein kinase ripk4 |
| OPB38824 | T4742_S00002.873 | serine/threonine-protein kinase Sgk2 [Metarhizium guizhouense ARSEF 977] |
| OPB45172 | T4742_S00010.79 | Serine/threonine-protein kinase tel1 |
| OPB42260 | T4742_S00004.362 | serine/threonine-protein kinase, putative |
| OPB38216 | T4742_S00002.263 | serine/threonine-protein phosphatase PP2A catalytic subunit |
| OPB38847 | T4742_S00002.896 | serine-threonine kinase receptor-associated protein |
| OPB43262 | T4742_S00022.66 | seryl-tRNA synthetase, class IIa. |
| OPB45087 | T4742_S00013.392 | Seryl-tRNASynthetase, class IIa. |
| OPB39119 | T4742_S00006.179 | SET domain protein [Trichoderma guizhouense] |
| OPB42503 | T4742_S00004.605 | SET domain-containing protein [Coniochaeta ligniaria NRRL 30616] |
| OPB45286 | T4742_S00010.197 | Set1 complex component spp1 [Escovopsis weberi] |
| OPB44359 | T4742_S00003.630 | SET1 complex component swd1 |
| OPB46493 | T4742_S00008.560 | sexual development protein |
| OPB41679 | T4742_S00018.1 | Sexual differentiation process protein ISP4 |
| OPB44770 | T4742_S00013.75 | Sexual differentiation process protein ISP4 |
| OPB36938 | T4742_S00007.656 | SGT1 protein [Trichoderma guizhouense] |
| OPB41478 | T4742_S00011.321 | Shikimate dehydrogenase |
| OPB46739 | T4742_S00009.181 | short chain dehydrogenase reductase [Grosmannia clavigera kw1407] |
| OPB36099 | T4742_S00021.15 | short chain dehydrogenase/reductase |
| OPB36125 | T4742_S00021.42 | short chain dehydrogenase/reductase |
| OPB36230 | T4742_S00021.148 | short chain dehydrogenase/reductase |
| OPB36292 | T4742_S00007.9 | short chain dehydrogenase/reductase |
| OPB36344 | T4742_S00007.61 | short chain dehydrogenase/reductase |

| | | |
|---|---|---|
| OPB36365 | T4742_S00007.82 | short chain dehydrogenase/reductase |
| OPB36368 | T4742_S00007.85 | short chain dehydrogenase/reductase |
| OPB36438 | T4742_S00007.155 | short chain dehydrogenase/reductase |
| OPB36906 | T4742_S00007.624 | short chain dehydrogenase/reductase |
| OPB37417 | T4742_S00005.444 | short chain dehydrogenase/reductase |
| OPB37504 | T4742_S00005.531 | short chain dehydrogenase/reductase |
| OPB37549 | T4742_S00005.576 | short chain dehydrogenase/reductase |
| OPB37585 | T4742_S00005.612 | short chain dehydrogenase/reductase |
| OPB37630 | T4742_S00005.657 | short chain dehydrogenase/reductase |
| OPB37657 | T4742_S00005.684 | short chain dehydrogenase/reductase |
| OPB37688 | T4742_S00005.715 | short chain dehydrogenase/reductase |
| OPB37783 | T4742_S00017.50 | short chain dehydrogenase/reductase |
| OPB38615 | T4742_S00002.664 | short chain dehydrogenase/reductase |
| OPB38682 | T4742_S00002.731 | short chain dehydrogenase/reductase |
| OPB38759 | T4742_S00002.808 | short chain dehydrogenase/reductase |
| OPB38843 | T4742_S00002.892 | short chain dehydrogenase/reductase |
| OPB38909 | T4742_S00002.958 | short chain dehydrogenase/reductase |
| OPB39493 | T4742_S00006.553 | short chain dehydrogenase/reductase |
| OPB39766 | T4742_S00015.110 | short chain dehydrogenase/reductase |
| OPB39905 | T4742_S00015.249 | short chain dehydrogenase/reductase |
| OPB40271 | T4742_S00001.350 | short chain dehydrogenase/reductase |
| OPB40294 | T4742_S00001.373 | short chain dehydrogenase/reductase |
| OPB40336 | T4742_S00001.415 | short chain dehydrogenase/reductase |
| OPB40503 | T4742_S00001.582 | short chain dehydrogenase/reductase |
| OPB40557 | T4742_S00001.636 | short chain dehydrogenase/reductase |
| OPB40568 | T4742_S00001.647 | short chain dehydrogenase/reductase |
| OPB40744 | T4742_S00001.825 | short chain dehydrogenase/reductase |
| OPB40767 | T4742_S00001.848 | short chain dehydrogenase/reductase |
| OPB40846 | T4742_S00001.927 | short chain dehydrogenase/reductase |
| OPB40847 | T4742_S00001.928 | short chain dehydrogenase/reductase |
| OPB40885 | T4742_S00001.966 | short chain dehydrogenase/reductase |
| OPB40986 | T4742_S00020.10 | short chain dehydrogenase/reductase |
| OPB41002 | T4742_S00020.26 | short chain dehydrogenase/reductase |
| OPB41057 | T4742_S00020.81 | short chain dehydrogenase/reductase |
| OPB41116 | T4742_S00020.140 | short chain dehydrogenase/reductase |
| OPB41119 | T4742_S00020.143 | short chain dehydrogenase/reductase |

| | | |
|---|---|---|
| OPB41135 | T4742_S00020.159 | short chain dehydrogenase/reductase |
| OPB41160 | T4742_S00020.184 | short chain dehydrogenase/reductase |
| OPB41328 | T4742_S00011.169 | short chain dehydrogenase/reductase |
| OPB41416 | T4742_S00011.258 | short chain dehydrogenase/reductase |
| OPB41467 | T4742_S00011.310 | short chain dehydrogenase/reductase |
| OPB41715 | T4742_S00018.38 | short chain dehydrogenase/reductase |
| OPB41839 | T4742_S00018.162 | short chain dehydrogenase/reductase |
| OPB42023 | T4742_S00004.125 | short chain dehydrogenase/reductase |
| OPB42158 | T4742_S00004.260 | short chain dehydrogenase/reductase |
| OPB42486 | T4742_S00004.588 | short chain dehydrogenase/reductase |
| OPB42510 | T4742_S00004.612 | short chain dehydrogenase/reductase |
| OPB42547 | T4742_S00004.650 | short chain dehydrogenase/reductase |
| OPB42595 | T4742_S00004.699 | short chain dehydrogenase/reductase |
| OPB42721 | T4742_S00004.827 | short chain dehydrogenase/reductase |
| OPB42802 | T4742_S00012.39 | short chain dehydrogenase/reductase |
| OPB42870 | T4742_S00012.107 | short chain dehydrogenase/reductase |
| OPB42874 | T4742_S00012.111 | short chain dehydrogenase/reductase |
| OPB43091 | T4742_S00012.328 | short chain dehydrogenase/reductase |
| OPB43341 | T4742_S00019.74 | short chain dehydrogenase/reductase |
| OPB43345 | T4742_S00019.78 | short chain dehydrogenase/reductase |
| OPB43365 | T4742_S00019.98 | short chain dehydrogenase/reductase |
| OPB43927 | T4742_S00003.198 | short chain dehydrogenase/reductase |
| OPB44049 | T4742_S00003.320 | short chain dehydrogenase/reductase |
| OPB44055 | T4742_S00003.326 | short chain dehydrogenase/reductase |
| OPB44172 | T4742_S00003.443 | short chain dehydrogenase/reductase |
| OPB44497 | T4742_S00003.769 | short chain dehydrogenase/reductase |
| OPB44517 | T4742_S00003.789 | short chain dehydrogenase/reductase |
| OPB44541 | T4742_S00003.813 | short chain dehydrogenase/reductase |
| OPB44672 | T4742_S00003.944 | short chain dehydrogenase/reductase |
| OPB44712 | T4742_S00013.16 | short chain dehydrogenase/reductase |
| OPB44752 | T4742_S00013.57 | short chain dehydrogenase/reductase |
| OPB44767 | T4742_S00013.72 | short chain dehydrogenase/reductase |
| OPB44807 | T4742_S00013.112 | short chain dehydrogenase/reductase |
| OPB44935 | T4742_S00013.240 | short chain dehydrogenase/reductase |
| OPB44961 | T4742_S00013.266 | short chain dehydrogenase/reductase |
| OPB45241 | T4742_S00010.150 | short chain dehydrogenase/reductase |

| | | |
|---|---|---|
| OPB45260 | T4742_S00010.169 | short chain dehydrogenase/reductase |
| OPB45402 | T4742_S00010.313 | short chain dehydrogenase/reductase |
| OPB45437 | T4742_S00010.348 | short chain dehydrogenase/reductase |
| OPB45452 | T4742_S00010.363 | short chain dehydrogenase/reductase |
| OPB45559 | T4742_S00010.470 | short chain dehydrogenase/reductase |
| OPB45832 | T4742_S00014.167 | short chain dehydrogenase/reductase |
| OPB45889 | T4742_S00014.224 | short chain dehydrogenase/reductase |
| OPB45910 | T4742_S00014.246 | short chain dehydrogenase/reductase |
| OPB45911 | T4742_S00014.247 | short chain dehydrogenase/reductase |
| OPB45925 | T4742_S00014.262 | short chain dehydrogenase/reductase |
| OPB45973 | T4742_S00008.39 | short chain dehydrogenase/reductase |
| OPB45995 | T4742_S00008.61 | short chain dehydrogenase/reductase |
| OPB46042 | T4742_S00008.108 | short chain dehydrogenase/reductase |
| OPB46296 | T4742_S00008.363 | short chain dehydrogenase/reductase |
| OPB46324 | T4742_S00008.391 | short chain dehydrogenase/reductase |
| OPB46383 | T4742_S00008.450 | short chain dehydrogenase/reductase |
| OPB46385 | T4742_S00008.452 | short chain dehydrogenase/reductase |
| OPB46528 | T4742_S00008.595 | short chain dehydrogenase/reductase |
| OPB46537 | T4742_S00008.604 | short chain dehydrogenase/reductase |
| OPB46849 | T4742_S00009.291 | short chain dehydrogenase/reductase |
| OPB46943 | T4742_S00009.385 | short chain dehydrogenase/reductase |
| OPB47068 | T4742_S00009.510 | short chain dehydrogenase/reductase |
| OPB47167 | T4742_S00009.609 | short chain dehydrogenase/reductase |
| OPB37697 | T4742_S00005.724 | short chain dehydrogenase/reductase |
| OPB37810 | T4742_S00017.77 | short chain dehydrogenase/reductase |
| OPB38744 | T4742_S00002.793 | short chain dehydrogenase/reductase |
| OPB38764 | T4742_S00002.813 | short chain dehydrogenase/reductase |
| OPB38849 | T4742_S00002.898 | short chain dehydrogenase/reductase |
| OPB38855 | T4742_S00002.904 | short chain dehydrogenase/reductase |
| OPB39402 | T4742_S00006.462 | short chain dehydrogenase/reductase |
| OPB39561 | T4742_S00006.621 | short chain dehydrogenase/reductase |
| OPB40362 | T4742_S00001.441 | short chain dehydrogenase/reductase |
| OPB40871 | T4742_S00001.952 | short chain dehydrogenase/reductase |
| OPB41285 | T4742_S00011.126 | short chain dehydrogenase/reductase |
| OPB42817 | T4742_S00012.54 | short chain dehydrogenase/reductase |
| OPB43953 | T4742_S00003.224 | short chain dehydrogenase/reductase |

| | | |
|---|---|---|
| OPB44132 | T4742_S00003.403 | short chain dehydrogenase/reductase |
| OPB44507 | T4742_S00003.779 | short chain dehydrogenase/reductase |
| OPB46286 | T4742_S00008.353 | short chain dehydrogenase/reductase |
| OPB38570 | T4742_S00002.619 | short chain dehydrogenase/reductase [Trichoderma guizhouense] |
| OPB39105 | T4742_S00006.165 | short chain dehydrogenase/reductase family oxidoreductase |
| OPB40227 | T4742_S00001.306 | short chian dehydrogenase/reductase |
| OPB47150 | T4742_S00009.592 | short-chain dehydrogenase [Aspergillus ustus] |
| OPB46447 | T4742_S00008.514 | short-chain dehydrogenase reductase sdr [Colletotrichum gloeosporioides Nara gc5] |
| OPB35967 | T4742_S00023.2 | short-chain dehydrogenase/reductase |
| OPB36106 | T4742_S00021.22 | Short-chain dehydrogenase/reductase |
| OPB36153 | T4742_S00021.70 | Short-chain dehydrogenase/reductase |
| OPB36187 | T4742_S00021.105 | Short-chain dehydrogenase/reductase |
| OPB36328 | T4742_S00007.45 | Short-chain dehydrogenase/reductase |
| OPB36426 | T4742_S00007.143 | Short-chain dehydrogenase/reductase |
| OPB36546 | T4742_S00007.263 | Short-chain dehydrogenase/reductase |
| OPB36780 | T4742_S00007.498 | short-chain dehydrogenase/reductase |
| OPB37507 | T4742_S00005.534 | Short-chain dehydrogenase/reductase |
| OPB37641 | T4742_S00005.668 | Short-chain dehydrogenase/reductase |
| OPB38376 | T4742_S00002.424 | Short-chain dehydrogenase/reductase |
| OPB38860 | T4742_S00002.909 | Short-chain dehydrogenase/reductase |
| OPB38862 | T4742_S00002.911 | Short-chain dehydrogenase/reductase |
| OPB38868 | T4742_S00002.917 | short-chain dehydrogenase/reductase |
| OPB40335 | T4742_S00001.414 | Short-chain dehydrogenase/reductase |
| OPB40579 | T4742_S00001.659 | Short-chain dehydrogenase/reductase |
| OPB40780 | T4742_S00001.861 | Short-chain dehydrogenase/reductase |
| OPB41289 | T4742_S00011.130 | Short-chain dehydrogenase/reductase |
| OPB41290 | T4742_S00011.131 | Short-chain dehydrogenase/reductase |
| OPB41739 | T4742_S00018.62 | Short-chain dehydrogenase/reductase |
| OPB41753 | T4742_S00018.76 | Short-chain dehydrogenase/reductase |
| OPB41919 | T4742_S00004.21 | Short-chain dehydrogenase/reductase |
| OPB42128 | T4742_S00004.230 | Short-chain dehydrogenase/reductase |
| OPB42655 | T4742_S00004.760 | Short-chain dehydrogenase/reductase |
| OPB42760 | T4742_S00004.866 | Short-chain dehydrogenase/reductase |
| OPB42779 | T4742_S00012.16 | short-chain dehydrogenase/reductase |
| OPB42813 | T4742_S00012.50 | Short-chain dehydrogenase/reductase |
| OPB43159 | T4742_S00012.396 | Short-chain dehydrogenase/reductase |

| | | |
|---|---|---|
| OPB43429 | T4742_S00019.162 | Short-chain dehydrogenase/reductase |
| OPB43972 | T4742_S00003.243 | Short-chain dehydrogenase/reductase |
| OPB44338 | T4742_S00003.609 | Short-chain dehydrogenase/reductase |
| OPB44488 | T4742_S00003.760 | short-chain dehydrogenase/reductase |
| OPB44562 | T4742_S00003.834 | Short-chain dehydrogenase/reductase |
| OPB44578 | T4742_S00003.850 | Short-chain dehydrogenase/reductase |
| OPB44702 | T4742_S00013.6 | short-chain dehydrogenase/reductase |
| OPB44759 | T4742_S00013.64 | Short-chain dehydrogenase/reductase |
| OPB45025 | T4742_S00013.330 | Short-chain dehydrogenase/reductase |
| OPB45098 | T4742_S00010.5 | Short-chain dehydrogenase/reductase |
| OPB45186 | T4742_S00010.93 | Short-chain dehydrogenase/reductase |
| OPB45344 | T4742_S00010.255 | Short-chain dehydrogenase/reductase |
| OPB45366 | T4742_S00010.277 | short-chain dehydrogenase/reductase |
| OPB45449 | T4742_S00010.360 | Short-chain dehydrogenase/reductase |
| OPB45478 | T4742_S00010.389 | short-chain dehydrogenase/reductase |
| OPB45754 | T4742_S00014.89 | short-chain dehydrogenase/reductase |
| OPB45913 | T4742_S00014.249 | Short-chain dehydrogenase/reductase |
| OPB45938 | T4742_S00008.4 | Short-chain dehydrogenase/reductase |
| OPB46114 | T4742_S00008.181 | Short-chain dehydrogenase/reductase |
| OPB46141 | T4742_S00008.208 | Short-chain dehydrogenase/reductase |
| OPB46217 | T4742_S00008.284 | Short-chain dehydrogenase/reductase |
| OPB46313 | T4742_S00008.380 | Short-chain dehydrogenase/reductase |
| OPB46326 | T4742_S00008.393 | Short-chain dehydrogenase/reductase |
| OPB46330 | T4742_S00008.397 | short-chain dehydrogenase/reductase |
| OPB46395 | T4742_S00008.462 | Short-chain dehydrogenase/reductase |
| OPB46422 | T4742_S00008.489 | Short-chain dehydrogenase/reductase |
| OPB46555 | T4742_S00008.622 | short-chain dehydrogenase/reductase |
| OPB46700 | T4742_S00009.142 | Short-chain dehydrogenase/reductase |
| OPB47096 | T4742_S00009.538 | Short-chain dehydrogenase/reductase |
| OPB37367 | T4742_S00005.394 | short-chain dehydrogenase/reductase |
| OPB40903 | T4742_S00001.984 | short-chain dehydrogenase/reductase |
| OPB41214 | T4742_S00011.54 | short-chain dehydrogenase/reductase |
| OPB42837 | T4742_S00012.74 | short-chain dehydrogenase/reductase |
| OPB43430 | T4742_S00019.163 | short-chain dehydrogenase/reductase |
| OPB43859 | T4742_S00003.130 | short-chain dehydrogenase/reductase |
| OPB43888 | T4742_S00003.159 | short-chain dehydrogenase/reductase |

| | | |
|---|---|---|
| OPB44700 | T4742_S00013.4 | short-chain dehydrogenase/reductase |
| OPB45657 | T4742_S00010.568 | short-chain dehydrogenase/reductase |
| OPB45986 | T4742_S00008.52 | short-chain dehydrogenase/reductase |
| OPB46135 | T4742_S00008.202 | short-chain dehydrogenase/reductase |
| OPB41759 | T4742_S00018.82 | short-chain dehydrogenase/reductase |
| OPB42112 | T4742_S00004.214 | Short-chain dehydrogenase/reductase SOR8 |
| OPB37806 | T4742_S00017.73 | short-chain dehydrogenases/reductase |
| OPB42818 | T4742_S00012.55 | short-chain dehydrogenases/reductase |
| OPB45268 | T4742_S00010.179 | short-chain dehydrogenases/reductase |
| OPB47093 | T4742_S00009.535 | short-chain dehydrogenases/reductase |
| OPB46804 | T4742_S00009.246 | SHR3, an ER membrane protein involved in packing of amino acid permeases |
| OPB43031 | T4742_S00012.268 | sideroflexin-1 |
| OPB40727 | T4742_S00001.807 | siderophore biosynthesis lipase/esterase |
| OPB40724 | T4742_S00001.804 | siderophore transporter |
| OPB42731 | T4742_S00004.837 | siderophore transporter |
| OPB43222 | T4742_S00022.25 | siderophore transporter |
| OPB44584 | T4742_S00003.856 | siderophore transporter |
| OPB45590 | T4742_S00010.501 | siderophore transporter |
| OPB40178 | T4742_S00001.257 | Signal peptidase complex subunit Spc3 |
| OPB41439 | T4742_S00011.282 | Signal peptidase complex, subunit Spc2, SPC25 |
| OPB42626 | T4742_S00004.731 | Signal peptidase Sec11 |
| OPB46613 | T4742_S00009.55 | signal peptidase spc12 |
| OPB41453 | T4742_S00011.296 | signal recognition particle receptor, beta subunit |
| OPB39978 | T4742_S00001.57 | Signal recognition particle, SRP54 subunit, helical bundle |
| OPB36942 | T4742_S00007.660 | Signal recognition particle, subunit Srp14 |
| OPB42388 | T4742_S00004.490 | Signal recognition particle, subunit Srp19 |
| OPB37587 | T4742_S00005.614 | Signal recognition particle, subunit Srp54 |
| OPB38428 | T4742_S00002.476 | Signal recognition particle, subunit Srp68 |
| OPB45060 | T4742_S00013.365 | Signal recognition particle, subunit Srp72 |
| OPB46864 | T4742_S00009.306 | signalosome subunit 4 (CsnD) |
| OPB46737 | T4742_S00009.179 | signalosome subunit 5 (CsnE) putatively involved in regulation of sexual development |
| OPB45199 | T4742_S00010.107 | silencing information regulator, Sir2 family |
| OPB44419 | T4742_S00003.691 | Sin3 complex subunit Stb2 based on homology to the corresponding proteins of Aspergillus fumigatus and Neurospora crassa. |
| OPB46681 | T4742_S00009.123 | Sin3-associated polypeptide Sap18 |
| OPB46868 | T4742_S00009.310 | Sin4, RNA polymerase II Mediator complex subunit |
| OPB45740 | T4742_S00014.75 | Single-stranded nucleic acid binding R3H |

| | | |
|---|---|---|
| OPB45342 | T4742_S00010.253 | sir2 family histone deacetylase |
| OPB46971 | T4742_S00009.413 | sir2 family histone deacetylase |
| OPB42552 | T4742_S00004.655 | siroheme synthase |
| OPB45246 | T4742_S00010.155 | siroheme synthase |
| OPB46470 | T4742_S00008.537 | SIT4 phosphatase-associated protein |
| OPB38553 | T4742_S00002.602 | sla2, adaptor protein that links actin to clathrin |
| OPB37336 | T4742_S00005.363 | Small GTPase of the Rab/Ypt family, ypt7 and rab7 involved in vacuolar biogenesis |
| OPB44466 | T4742_S00003.738 | small nuclear ribonucleoprotein |
| OPB37422 | T4742_S00005.449 | Small nuclear ribonucleoprotein (Sm protein) |
| OPB42933 | T4742_S00012.170 | Small nuclear ribonucleoprotein (Sm protein) |
| OPB40984 | T4742_S00020.8 | small nuclear ribonucleoprotein E |
| OPB42608 | T4742_S00004.712 | small nuclear ribonucleoprotein F |
| OPB42916 | T4742_S00012.153 | small nuclear ribonucleoprotein LSM2 |
| OPB42175 | T4742_S00004.277 | small nuclear ribonucleoprotein Lsm8 |
| OPB37279 | T4742_S00005.306 | small nuclear ribonucleoprotein Sm D3 |
| OPB39030 | T4742_S00006.89 | SNARE complex subunit Vam7 |
| OPB40002 | T4742_S00001.81 | SNF1 kinase complex beta-subunit [Colletotrichum incanum] |
| OPB39272 | T4742_S00006.332 | snf2 chromatin remodeling protein |
| OPB42983 | T4742_S00012.220 | SNF2 family chromodomain-helicase DNA-binding protein |
| OPB36406 | T4742_S00007.123 | SNF2 family DNA-dependent ATPase |
| OPB38597 | T4742_S00002.646 | SNF2 family DNA-dependent ATPase |
| OPB42311 | T4742_S00004.413 | SNF2 family DNA-dependent ATPase domain-containing protein |
| OPB38213 | T4742_S00002.260 | SNF2 family domain-containing protein |
| OPB45459 | T4742_S00010.370 | SNF2 family domain-containing protein, partial [Metarhizium anisopliae ARSEF 549] |
| OPB38429 | T4742_S00002.477 | SNF2 family helicase |
| OPB39436 | T4742_S00006.496 | SNF2 family helicase/ATPase, putative |
| OPB45371 | T4742_S00010.282 | SNF2 family helicase/ATPase, related to ISWI2 |
| OPB44060 | T4742_S00003.331 | SNF2 family protein, Rad26 related |
| OPB45441 | T4742_S00010.352 | SNF2 family protein, Swr-like |
| OPB39443 | T4742_S00006.503 | SNF2-like helicase |
| OPB37109 | T4742_S00005.136 | SNF2-like helicase, ATPase domain |
| OPB39352 | T4742_S00006.412 | SNF2-related |
| OPB42045 | T4742_S00004.147 | snf5/smarcb1/ini1 |
| OPB39898 | T4742_S00015.242 | Snf7 family protein |
| OPB40743 | T4742_S00001.824 | SnoaL_4 |
| OPB47134 | T4742_S00009.576 | Sodium Bile acid symporter family protein |

| | | |
|---|---|---|
| OPB47141 | T4742_S00009.583 | Sodium/calcium exchanger membrane region |
| OPB41071 | T4742_S00020.95 | sodium/hydrogen antiporter |
| OPB39755 | T4742_S00015.99 | sodium/hydrogen exchanger |
| OPB45193 | T4742_S00010.101 | sodium/hydrogen exchanger |
| OPB38731 | T4742_S00002.780 | sorbitol dehydrogenase |
| OPB38461 | T4742_S00002.509 | Spc97 / Spc98 family proteins involved in spindle pole body |
| OPB38430 | T4742_S00002.478 | Spc97 / Spc98 family, spindle pole body |
| OPB40017 | T4742_S00001.96 | Spc97/Spc98 |
| OPB45303 | T4742_S00010.214 | Spermatogenesis-associated protein 20 [Escovopsis weberi] |
| OPB39472 | T4742_S00006.532 | Spermine/spermidine synthase |
| OPB41997 | T4742_S00004.99 | Spermine/spermidine synthase |
| OPB40449 | T4742_S00001.528 | Sphingoid long-chain base kinase |
| OPB46776 | T4742_S00009.218 | sphingomyelinase family protein, putative |
| OPB45309 | T4742_S00010.220 | sphingosine N-acyltransferase lac1 |
| OPB43160 | T4742_S00012.397 | sphingosine-1-phosphate phosphohydrolase |
| OPB45539 | T4742_S00010.450 | spindle assembly checkpoint component MAD1 |
| OPB46472 | T4742_S00008.539 | Spindle pole body component, |
| OPB36911 | T4742_S00007.629 | spindle-pole body protein (Pcp1) |
| OPB41361 | T4742_S00011.202 | Splicing coactivator SRm160/300, subunit SRm300 |
| OPB41994 | T4742_S00004.96 | Splicing coactivator SRm160/300, subunit SRm300 |
| OPB37515 | T4742_S00005.542 | splicing factor 3a |
| OPB36984 | T4742_S00005.11 | splicing factor 3A subunit 2 |
| OPB42922 | T4742_S00012.159 | splicing factor 3B subunit 1, putative |
| OPB38718 | T4742_S00002.767 | splicing factor 3B subunit 10 |
| OPB36570 | T4742_S00007.287 | splicing factor SPF30. |
| OPB38996 | T4742_S00006.55 | splicing factor U2AF 23 kDa subunit |
| OPB42924 | T4742_S00012.161 | splicing factor U2AF 65 kDa subunit |
| OPB38367 | T4742_S00002.415 | spo7 [Trichoderma guizhouense] |
| OPB36966 | T4742_S00007.684 | SprT family metallopeptidase |
| OPB44228 | T4742_S00003.499 | squalene synthase |
| OPB41635 | T4742_S00011.478 | SRP receptor, alphaSubunit |
| OPB39172 | T4742_S00006.232 | SRP receptor, betaSubunit |
| OPB36415 | T4742_S00007.132 | ß-arrestin protein, shares similarity with Aspergillus idulans CreD, possible inhibitor of G-protein coupled receptors |
| OPB46808 | T4742_S00009.250 | ß-arrestin, probable adaptor and transducer involved in signalling |
| OPB35941 | T4742_S00025.14 | SSCP |
| OPB36054 | T4742_S00024.15 | SSCP |

| | | |
|---|---|---|
| OPB36111 | T4742_S00021.27 | SSCP |
| OPB36120 | T4742_S00021.37 | SSCP |
| OPB36137 | T4742_S00021.54 | SSCP |
| OPB36179 | T4742_S00021.97 | SSCP |
| OPB36185 | T4742_S00021.103 | SSCP |
| OPB36613 | T4742_S00007.331 | SSCP |
| OPB37168 | T4742_S00005.195 | SSCP |
| OPB37186 | T4742_S00005.213 | SSCP |
| OPB37502 | T4742_S00005.529 | SSCP |
| OPB37603 | T4742_S00005.630 | SSCP |
| OPB37634 | T4742_S00005.661 | SSCP |
| OPB38872 | T4742_S00002.921 | SSCP |
| OPB40093 | T4742_S00001.172 | SSCP |
| OPB41044 | T4742_S00020.68 | SSCP |
| OPB41146 | T4742_S00020.170 | SSCP |
| OPB42122 | T4742_S00004.224 | SSCP |
| OPB43346 | T4742_S00019.79 | SSCP |
| OPB44487 | T4742_S00003.759 | SSCP |
| OPB44600 | T4742_S00003.872 | SSCP |
| OPB44820 | T4742_S00013.125 | SSCP |
| OPB45466 | T4742_S00010.377 | SSCP |
| OPB45876 | T4742_S00014.211 | SSCP |
| OPB37202 | T4742_S00005.229 | SSCP |
| OPB38523 | T4742_S00002.572 | SSCP |
| OPB38637 | T4742_S00002.686 | SSCP |
| OPB38899 | T4742_S00002.948 | SSCP |
| OPB38900 | T4742_S00002.949 | SSCP |
| OPB39052 | T4742_S00006.111 | SSCP |
| OPB39663 | T4742_S00015.7 | SSCP |
| OPB40230 | T4742_S00001.309 | SSCP |
| OPB40410 | T4742_S00001.489 | SSCP |
| OPB40452 | T4742_S00001.531 | SSCP |
| OPB40588 | T4742_S00001.668 | SSCP |
| OPB40661 | T4742_S00001.741 | SSCP |
| OPB40679 | T4742_S00001.759 | SSCP |
| OPB40918 | T4742_S00001.999 | SSCP |

| | | |
|---|---|---|
| OPB41390 | T4742_S00011.231 | SSCP |
| OPB41745 | T4742_S00018.68 | SSCP |
| OPB43042 | T4742_S00012.279 | SSCP |
| OPB43437 | T4742_S00019.170 | SSCP |
| OPB46132 | T4742_S00008.199 | SSCP |
| OPB35965 | T4742_S00025.38 | SSCP protein [Trichoderma guizhouense] |
| OPB37072 | T4742_S00005.99 | SSCP protein [Trichoderma guizhouense] |
| OPB37107 | T4742_S00005.134 | SSCP protein [Trichoderma guizhouense] |
| OPB37358 | T4742_S00005.385 | SSCP protein [Trichoderma guizhouense] |
| OPB38749 | T4742_S00002.798 | SSCP protein [Trichoderma guizhouense] |
| OPB39269 | T4742_S00006.329 | SSCP protein [Trichoderma guizhouense] |
| OPB39474 | T4742_S00006.534 | SSCP protein [Trichoderma guizhouense] |
| OPB39692 | T4742_S00015.36 | SSCP protein [Trichoderma guizhouense] |
| OPB42940 | T4742_S00012.177 | SSCP protein [Trichoderma guizhouense] |
| OPB43695 | T4742_S00016.215 | SSCP protein [Trichoderma guizhouense] |
| OPB39350 | T4742_S00006.410 | ssDNA binding protein |
| OPB36062 | T4742_S00024.23 | ß-lactamase class C |
| OPB40835 | T4742_S00001.916 | ß-lactamase class C |
| OPB40334 | T4742_S00001.413 | ß-lactamase-like protein |
| OPB42668 | T4742_S00004.773 | ß-lactamase-like protein |
| OPB40565 | T4742_S00001.644 | SSN6, General transcriptional corepressor |
| OPB43122 | T4742_S00012.359 | SSU processome component NO58 |
| OPB46250 | T4742_S00008.317 | SSU processome component UTP7 |
| OPB42160 | T4742_S00004.262 | SSU processome subunit |
| OPB46176 | T4742_S00008.243 | stage V sporulation protein K |
| OPB38258 | T4742_S00002.305 | stearic acid desaturase Sde1 |
| OPB37074 | T4742_S00005.101 | sterol delta(24(28))- reductase, |
| OPB36606 | T4742_S00007.324 | sterol desaturase family |
| OPB43083 | T4742_S00012.320 | sterol o-acyltransferase |
| OPB44983 | T4742_S00013.288 | Sterol O-acyltransferase/Diacylglycerol O-acyltransferase |
| OPB41539 | T4742_S00011.382 | Steryl ester hydrolase |
| OPB41590 | T4742_S00011.433 | steryl ester lipase TPL1 |
| OPB41967 | T4742_S00004.69 | stomatin-like protein |
| OPB43121 | T4742_S00012.358 | stomatin-like protein |
| OPB45115 | T4742_S00010.22 | stress activated MAP kinase interacting protein |
| OPB44755 | T4742_S00013.60 | stress response A/B barrel domain protein |

| | | |
|---|---|---|
| OPB39828 | T4742_S00015.172 | stress response protein Rds1; secreted |
| OPB36385 | T4742_S00007.102 | Stress response protein UspA |
| OPB36885 | T4742_S00007.603 | stress response regulator SrrA |
| OPB40750 | T4742_S00001.831 | stress responsive A/B barrel domain-containing protein [Purpureocillium lilacinum] |
| OPB40197 | T4742_S00001.276 | Stress responsive alpha-beta barrel |
| OPB36293 | T4742_S00007.10 | structural maintenance of chromosomes 5 smc5 |
| OPB36939 | T4742_S00007.657 | Structural maintenance of chromosomes protein 6 |
| OPB39015 | T4742_S00006.74 | Succinate dehydrogenase |
| OPB41448 | T4742_S00011.291 | succinate dehydrogenase |
| OPB37196 | T4742_S00005.223 | succinate dehydrogenase cytochrome b subunit |
| OPB39217 | T4742_S00006.277 | Succinate dehydrogenase, cytochrome b subunit |
| OPB36218 | T4742_S00021.136 | succinate semialdehyde dehydrogenase (NADP) |
| OPB45833 | T4742_S00014.168 | succinate semialdehyde dehydrogenase, NADP |
| OPB41774 | T4742_S00018.97 | succinate-CoA ligase, alpha subunit |
| OPB42727 | T4742_S00004.833 | succinate-semialdehyde dehydrogenase Uga2 |
| OPB36904 | T4742_S00007.622 | succinyl-CoA:3-ketoacid-coenzyme A transferase subunit A |
| OPB40244 | T4742_S00001.323 | sugar isomerase |
| OPB36104 | T4742_S00021.20 | Sulfatase |
| OPB37682 | T4742_S00005.709 | sulfatase |
| OPB37863 | T4742_S00017.130 | Sulfatase |
| OPB40031 | T4742_S00001.110 | sulfatase |
| OPB41243 | T4742_S00011.84 | sulfatase |
| OPB43809 | T4742_S00003.80 | Sulfatase |
| OPB38572 | T4742_S00002.621 | Sulfate adenylyltransferase |
| OPB35978 | T4742_S00023.13 | Sulfate permease |
| OPB40398 | T4742_S00001.477 | Sulfate permease |
| OPB43632 | T4742_S00016.152 | Sulfate permease |
| OPB46482 | T4742_S00008.549 | Sulfate permease |
| OPB37776 | T4742_S00017.43 | Sulfate permease I (CYS-13) |
| OPB40812 | T4742_S00001.893 | sulfide:quinone oxidoreductase/flavo-binding protein |
| OPB37687 | T4742_S00005.714 | Sulfite oxidase, molybdopterin-binding component |
| OPB40298 | T4742_S00001.377 | Sulfite oxidase, molybdopterin-binding component |
| OPB44668 | T4742_S00003.940 | Sulfite oxidase, molybdopterin-binding component |
| OPB39880 | T4742_S00015.224 | sulfite reductase, ß-subunit |
| OPB36514 | T4742_S00007.231 | sulfite reductase,Alpha subunit |
| OPB46832 | T4742_S00009.274 | Sulphate permease |

| | | |
|---|---|---|
| OPB41595 | T4742_S00011.438 | Superoxide dismutase, copper/zinc binding |
| OPB36667 | T4742_S00007.385 | Suppressor of meiotic Silencing QDE2 |
| OPB46648 | T4742_S00009.90 | Sur2 sphinganine C4-hydroxylase of Saccharomyces cerevisiae |
| OPB43487 | T4742_S00016.7 | Sur4p fatty acid elongase ofSaccharomyces cerevisiae |
| OPB38321 | T4742_S00002.369 | Survival factor 1 [Trichoderma guizhouense] |
| OPB38198 | T4742_S00002.245 | SWI/SNF complex protein |
| OPB43506 | T4742_S00016.26 | SWI/SNF complex protein |
| OPB45116 | T4742_S00010.23 | SWI/SNF complex protein |
| OPB45352 | T4742_S00010.263 | SWI/SNF complex protein |
| OPB43386 | T4742_S00019.119 | SWIB/MDM2 domain protein |
| OPB38728 | T4742_S00002.777 | SWI-SNF chromatin-remodeling complex protein |
| OPB39753 | T4742_S00015.97 | SWI-SNF chromatin-remodeling complex protein |
| OPB42243 | T4742_S00004.345 | SWI-SNF complex subunit (Snf5), putative |
| OPB45638 | T4742_S00010.549 | swollenin |
| OPB37442 | T4742_S00005.469 | SWR1-complex protein 4, putative |
| OPB39270 | T4742_S00006.330 | Swr1p complex component (Swc5), putative |
| OPB42326 | T4742_S00004.428 | SYF2 splicing factor |
| OPB44785 | T4742_S00013.90 | Synaptic vesicle transporter SVOP and related transporters (major facilitator superfamily) |
| OPB45227 | T4742_S00010.136 | Synaptobrevin/VAMP-like protein |
| OPB45557 | T4742_S00010.468 | Synaptobrevin/VAMP-like protein (VAMP71 family) |
| OPB46511 | T4742_S00008.578 | tagatose bisphosphate aldolase |
| OPB37195 | T4742_S00005.222 | TATA box binding protein associated factor (TAF) |
| OPB36391 | T4742_S00007.108 | TATA-binding protein |
| OPB37220 | T4742_S00005.247 | TATA-binding protein interacting (TIP20) , putative |
| OPB37597 | T4742_S00005.624 | Tautomerase |
| OPB42891 | T4742_S00012.128 | Tautomerase_3 |
| OPB37237 | T4742_S00005.264 | taxilin |
| OPB36858 | T4742_S00007.576 | T-complex protein 1 |
| OPB42059 | T4742_S00004.161 | T-complex protein 1 |
| OPB36864 | T4742_S00007.582 | T-complex protein 1, HSP60 family |
| OPB42019 | T4742_S00004.121 | T-complex protein 1, Hsp60 family |
| OPB46795 | T4742_S00009.237 | telomerase reverse transcriptase |
| OPB43057 | T4742_S00012.294 | Telomere and ribosome associated protein |
| OPB37429 | T4742_S00005.456 | Terpenoid synthase TSP9 |
| OPB37532 | T4742_S00005.559 | Terpenoid synthases |
| OPB37782 | T4742_S00017.49 | Terpenoid synthases |

| | | |
|---|---|---|
| OPB38922 | T4742_S00002.971 | Terpenoid synthases |
| OPB39108 | T4742_S00006.168 | Terpenoid synthases |
| OPB39262 | T4742_S00006.322 | Terpenoid synthases |
| OPB40643 | T4742_S00001.723 | Terpenoid synthases |
| OPB41795 | T4742_S00018.118 | Terpenoid synthases |
| OPB45420 | T4742_S00010.331 | Terpenoid synthases |
| OPB46243 | T4742_S00008.310 | Terpenoid synthases |
| OPB37116 | T4742_S00005.143 | Tethering factor for nuclear proteasome STS1 [Trichoderma guizhouense] |
| OPB39228 | T4742_S00006.288 | tetrahydrofolate dehydrogenase/cyclohydrolase |
| OPB39806 | T4742_S00015.150 | tetraspanin |
| OPB44828 | T4742_S00013.133 | tetratricopeptide [Trichoderma gamsii] |
| OPB40355 | T4742_S00001.434 | tetratricopeptide repeat domain containing protein [Aspergillus nomius NRRL 13137] |
| OPB44536 | T4742_S00003.808 | Tetratricopeptide-like helical domain [Umbilicaria pustulata] |
| OPB45229 | T4742_S00010.138 | Tetratricopeptide-like helical domain [Umbilicaria pustulata] |
| OPB45763 | T4742_S00014.98 | TFIID and SAGA complexe subunit |
| OPB37872 | T4742_S00017.139 | TFIIF-interacting CTD phosphatase (involved in RNA polymerase II regulation) |
| OPB39332 | T4742_S00006.392 | TFIIH |
| OPB41104 | T4742_S00020.128 | thiamin biosynthesis protein thi-4 , putative |
| OPB45867 | T4742_S00014.202 | Thiamine pyrophosphate-requiring enzyme / benzoyl formate decarboxylase |
| OPB39158 | T4742_S00006.218 | Thiamine pyrophosphate-requiring enzymes |
| OPB39910 | T4742_S00015.254 | thiamine pyrophosphokinase |
| OPB40344 | T4742_S00001.423 | Thiamine pyrophosphokinase |
| OPB37373 | T4742_S00005.400 | thiamine-repressible mitochondrial transport protein THI74, putative |
| OPB40386 | T4742_S00001.465 | Thiazole biosynthetic enzyme (Stress-inducible protein sti35) |
| OPB41432 | T4742_S00011.275 | Thioesterase |
| OPB36632 | T4742_S00007.350 | thioesterase family protein |
| OPB38300 | T4742_S00002.347 | thioesterase family protein |
| OPB39143 | T4742_S00006.203 | thioesterase family protein |
| OPB40779 | T4742_S00001.860 | thioesterase family protein |
| OPB41823 | T4742_S00018.146 | thioesterase family protein |
| OPB44899 | T4742_S00013.204 | thioesterase family protein |
| OPB45892 | T4742_S00014.228 | thioesterase family protein |
| OPB40399 | T4742_S00001.478 | Thiolase |
| OPB44133 | T4742_S00003.404 | Thiopurine S-methyltransferase |
| OPB37375 | T4742_S00005.402 | thioredoxin peroxidase |
| OPB38493 | T4742_S00002.542 | thioredoxin peroxidase |

| | | |
|---|---|---|
| OPB41006 | T4742_S00020.30 | thioredoxin peroxidase |
| OPB41024 | T4742_S00020.48 | thioredoxin peroxidase |
| OPB43117 | T4742_S00012.354 | thioredoxin peroxidase |
| OPB35968 | T4742_S00023.3 | thioredoxin reductase |
| OPB37640 | T4742_S00005.667 | Thioredoxin reductase |
| OPB41459 | T4742_S00011.302 | thioredoxin reductase |
| OPB43288 | T4742_S00019.21 | thioredoxin reductase |
| OPB44308 | T4742_S00003.579 | thioredoxin reductase |
| OPB44817 | T4742_S00013.122 | thioredoxin reductase |
| OPB46127 | T4742_S00008.194 | thioredoxin reductase |
| OPB46962 | T4742_S00009.404 | thioredoxin reductase |
| OPB37849 | T4742_S00017.116 | thiosulfate sulfurtransferase |
| OPB45413 | T4742_S00010.324 | tho2 protein [Cordyceps confragosa RCEF 1005] |
| OPB36684 | T4742_S00007.402 | threonine aldolase |
| OPB41401 | T4742_S00011.243 | threonine aldolase GLY1 |
| OPB37821 | T4742_S00017.88 | Threonine dehydratase |
| OPB42616 | T4742_S00004.720 | Threonine dehydratase |
| OPB43615 | T4742_S00016.135 | Threonine synthase |
| OPB44657 | T4742_S00003.929 | Threonine synthase |
| OPB40059 | T4742_S00001.138 | Threonine/serine dehydratases |
| OPB37061 | T4742_S00005.88 | threonyl-tRNA synthetase, class IIa. |
| OPB44456 | T4742_S00003.728 | threonyl-tRNA synthetase, class IIa. |
| OPB37600 | T4742_S00005.627 | Thymidylate kinase |
| OPB44242 | T4742_S00003.513 | thymidylate synthase |
| OPB39427 | T4742_S00006.487 | Top3 |
| OPB43274 | T4742_S00019.7 | topisomerase II associated protein (Pat1), putative |
| OPB36812 | T4742_S00007.530 | TOR kinase |
| OPB45601 | T4742_S00010.512 | trans-aconitate methyltransferase |
| OPB40827 | T4742_S00001.908 | transaldolase |
| OPB41593 | T4742_S00011.436 | transaldolase |
| OPB44647 | T4742_S00003.919 | transaldolase |
| OPB46423 | T4742_S00008.490 | transaldolase |
| OPB39991 | T4742_S00001.70 | transcription elongation factor SPT4 |
| OPB39521 | T4742_S00006.581 | Transcription elongation factor spt-6 |
| OPB41594 | T4742_S00011.437 | transcription factor (Snd1/p100) |
| OPB44327 | T4742_S00003.598 | transcription factor AbaA |

| | | |
|---|---|---|
| OPB45343 | T4742_S00010.254 | transcription factor CBF/NF-Y family |
| OPB41562 | T4742_S00011.405 | Transcription factor CBF/NF-Y/archaeal histone |
| OPB46563 | T4742_S00009.3 | Transcription factor CBF/NF-Y/archaeal histone |
| OPB38830 | T4742_S00002.879 | transcription factor IWS1 |
| OPB44380 | T4742_S00003.651 | Transcription factor jumonji |
| OPB45247 | T4742_S00010.156 | Transcription factor Jun |
| OPB37193 | T4742_S00005.220 | Transcription factor Opi1 |
| OPB42216 | T4742_S00004.318 | Transcription factor SipA3 |
| OPB42217 | T4742_S00004.319 | transcription factor SipA3 |
| OPB42393 | T4742_S00004.495 | transcription factor TFIID complex subunit Taf13 |
| OPB45065 | T4742_S00013.370 | transcription factor TFIIE complex alpha subunit |
| OPB43586 | T4742_S00016.106 | transcription factor TFIIH subunit Tfb4 |
| OPB38287 | T4742_S00002.334 | Transcription factor TFIIS |
| OPB44344 | T4742_S00003.615 | Transcription factor TFIIS |
| OPB46897 | T4742_S00009.339 | transcription initiation factor IIB |
| OPB39192 | T4742_S00006.252 | transcription initiation factor IID (TFIID-18) |
| OPB38397 | T4742_S00002.445 | Transcription initiation factor IID, 18 kDa subunit |
| OPB44205 | T4742_S00003.476 | transcription initiation factor TFIId 127kD subunit |
| OPB44979 | T4742_S00013.284 | transcription initiation factor TFIID subunit |
| OPB41087 | T4742_S00020.111 | transcription initiation factor TFIID subunit 14 |
| OPB46836 | T4742_S00009.278 | transcription initiation factor TFIID subunit 7 |
| OPB36354 | T4742_S00007.71 | transcription initiation factor TFIIE |
| OPB38788 | T4742_S00002.837 | Transcriptional activator HAC1, UPR regulator |
| OPB42946 | T4742_S00012.183 | transcriptional activator, zinc finger, NF-X1-type |
| OPB42470 | T4742_S00004.572 | transcriptional corepressor Cyc8 |
| OPB43294 | T4742_S00019.27 | transcriptional regulator APSES type |
| OPB43904 | T4742_S00003.175 | transcriptional regulator APSES type |
| OPB40267 | T4742_S00001.346 | transcriptional regulator ASM-1, involved in fruiting body development |
| OPB38320 | T4742_S00002.368 | transcriptional regulator GATA-type zinc finger protein ASD-4 (ascospore development protein 4) |
| OPB36891 | T4742_S00007.609 | transcriptional regulator Grainyhead/CP2 |
| OPB36998 | T4742_S00005.25 | transcriptional regulator HMG type |
| OPB39199 | T4742_S00006.259 | transcriptional regulator HMG type |
| OPB39701 | T4742_S00015.45 | transcriptional regulator HMG type |
| OPB44409 | T4742_S00003.680 | transcriptional regulator HMG type |
| OPB40389 | T4742_S00001.468 | transcriptional regulator MedA |
| OPB44447 | T4742_S00003.719 | transcriptional regulator NGG1, histone acetyltransferase subunit-3 like |

| | | |
|---|---|---|
| OPB40055 | T4742_S00001.134 | Transcriptional regulator PacG/VIB-1 |
| OPB45035 | T4742_S00013.340 | transcriptional regulator Rme1,  repressor of the meiosis regulator protein IME1 |
| OPB39318 | T4742_S00006.378 | transcriptional regulator STE12 |
| OPB44181 | T4742_S00003.452 | transcriptional regulator TAFII250, unknown |
| OPB42554 | T4742_S00004.657 | transcriptional regulator transcriptional regulator APSES type |
| OPB36284 | T4742_S00007.1 | transcriptional regulator UMC1 |
| OPB45978 | T4742_S00008.44 | transcriptional regulator, putative |
| OPB46273 | T4742_S00008.340 | transcriptional regulator, related to BDF1 |
| OPB37513 | T4742_S00005.540 | transcriptional regulator, unknown |
| OPB38013 | T4742_S00002.60 | transcriptional regulator, unknown |
| OPB39603 | T4742_S00006.663 | transcriptional regulator, unknown |
| OPB39901 | T4742_S00015.245 | transcriptional regulator, unknown |
| OPB42631 | T4742_S00004.736 | transcriptional regulator, unknown |
| OPB44276 | T4742_S00003.547 | transcriptional regulator, unknown |
| OPB44303 | T4742_S00003.574 | transcriptional regulator, unknown |
| OPB45508 | T4742_S00010.419 | transcriptional regulator, unknown |
| OPB41698 | T4742_S00018.20 | transcriptional regulatory protein [Colletotrichum gloeosporioides Nara gc5] |
| OPB46668 | T4742_S00009.110 | transferase [Trichoderma harzianum] |
| OPB36022 | T4742_S00023.57 | transferase hexapeptide domain protein [Trichoderma guizhouense] |
| OPB36108 | T4742_S00021.24 | transglutaminase |
| OPB39459 | T4742_S00006.519 | transketolase-like protein |
| OPB40293 | T4742_S00001.372 | transketolase-like protein |
| OPB43738 | T4742_S00003.9 | transketolase-like protein |
| OPB46281 | T4742_S00008.348 | translation elongation factor 1 beta by homology to the corresponding gene in other fungi. |
| OPB38715 | T4742_S00002.764 | Translation elongation factor 1a |
| OPB42524 | T4742_S00004.626 | translation elongation factor 3-like protein |
| OPB39996 | T4742_S00001.75 | Translation elongation factor EF1B, gamma chain, conserved |
| OPB40088 | T4742_S00001.167 | Translation elongation factor precursor from Aspergillus fumigatus. |
| OPB37977 | T4742_S00002.24 | translation factor eIF2 |
| OPB37832 | T4742_S00017.99 | translation initiation factor ? |
| OPB40208 | T4742_S00001.287 | translation initiation factor 2 beta subunit (Interpro motif IF5). |
| OPB38328 | T4742_S00002.376 | Translation initiation factor 3, subunit g (eIF-3g); homologue of yeastTIF35. |
| OPB39536 | T4742_S00006.596 | Translation initiation factor 3, subunit i (elf-3i) |
| OPB37625 | T4742_S00005.652 | translation initiation factor 3. |
| OPB40190 | T4742_S00001.269 | translation initiation factor 4e [Neurospora crassa]. |
| OPB46089 | T4742_S00008.156 | Translation initiation factor 6 (eIF6) by homologyToThe corresponding protein in other eukaryotes. |

| | | |
|---|---|---|
| OPB37555 | T4742_S00005.582 | translation initiation factor eIF-2B delta subunit. |
| OPB37557 | T4742_S00005.584 | translation initiation factor eIF2B subunit (Gcd2p). |
| OPB39382 | T4742_S00006.442 | translation initiation factor eIF3 subunit. |
| OPB46083 | T4742_S00008.150 | translation initiation factor eIF-4G |
| OPB46086 | T4742_S00008.153 | translation initiation factor eIF4-gamma/eIF5/eIF2-epsilon. |
| OPB37981 | T4742_S00002.28 | Translation initiation factor eIF-5A gene based on homologyToThe corresponding genes in several filamentous fungi. |
| OPB41928 | T4742_S00004.30 | translation initiation factor SUI1 and density-regulated protein. |
| OPB38561 | T4742_S00002.610 | translation initiation factor SUI1. |
| OPB38477 | T4742_S00002.525 | Translation initiation factor, eIF1A. |
| OPB42141 | T4742_S00004.243 | translation initiation protein Sua5p. |
| OPB41993 | T4742_S00004.95 | translation initiation regulator Gnc20 |
| OPB46209 | T4742_S00008.276 | Translation protein SH3-like |
| OPB36549 | T4742_S00007.266 | Translation release factor eRF3 in other fungi. |
| OPB46650 | T4742_S00009.92 | Translationally-controlled tumor protein homolog |
| OPB38453 | T4742_S00002.501 | Translocation protein Sec62 |
| OPB43037 | T4742_S00012.274 | Translocation protein Sec63 |
| OPB37012 | T4742_S00005.39 | Translocation protein Sec66 |
| OPB46806 | T4742_S00009.248 | translocator protein |
| OPB39983 | T4742_S00001.62 | Translocon-associated protein (TRAP), alpha subunit |
| OPB43384 | T4742_S00019.117 | Transmembran serin/threoninkinase Ire1 |
| OPB47044 | T4742_S00009.486 | Transmembrane adaptor Erv26 [Cordyceps brongniartii RCEF 3172] |
| OPB39645 | T4742_S00006.705 | Transport protein Uso1 [Trichoderma guizhouense] |
| OPB38170 | T4742_S00002.217 | transporter Avl9 [Trichoderma guizhouense] |
| OPB39505 | T4742_S00006.565 | transthyretin-like protein |
| OPB41688 | T4742_S00018.10 | TRAPP complex component Bet3 |
| OPB42231 | T4742_S00004.333 | TRAPP complex component Bet5 |
| OPB43075 | T4742_S00012.312 | TRAPP complex component Bet5 |
| OPB39899 | T4742_S00015.243 | TRAPP complex component Gsg1 |
| OPB37302 | T4742_S00005.329 | TRAPP complex component Trs120 |
| OPB37303 | T4742_S00005.330 | TRAPP complex component Trs120 |
| OPB47019 | T4742_S00009.461 | TRAPP complex component Trs130 |
| OPB41556 | T4742_S00011.399 | TRAPP complex component Trs20 |
| OPB41925 | T4742_S00004.27 | TRAPP complex component Trs23 |
| OPB46626 | T4742_S00009.68 | TRAPP complex component Trs33 |
| OPB37223 | T4742_S00005.250 | TRAPP complex componnet Trs31 |
| OPB42130 | T4742_S00004.232 | triacylglycerol lipase |

| | | |
|---|---|---|
| OPB43634 | T4742_S00016.154 | Triacylglycerol lipase |
| OPB36080 | T4742_S00024.41 | trihydroxytoluene oxygenase |
| OPB43193 | T4742_S00012.430 | triose phosphate/3-phosphoglycerate/phosphate translocator |
| OPB43097 | T4742_S00012.334 | triose-phosphate isomerase-like protein |
| OPB40100 | T4742_S00001.179 | tRNA (cytosine-5-)-methyltransferase NCL1 |
| OPB36883 | T4742_S00007.601 | tRNA (guanine-N(1)-)-methyltransferase, putative |
| OPB43583 | T4742_S00016.103 | tRNA (uracil-5-)-methyltransferase/TrmA. |
| OPB38314 | T4742_S00002.361 | tRNA cytosine-5-methylases |
| OPB39790 | T4742_S00015.134 | tRNA intron endonuclease, N-terminal. |
| OPB37219 | T4742_S00005.246 | tRNA isopentenyltransferase. |
| OPB40432 | T4742_S00001.511 | tRNA ligase TRL1 |
| OPB37314 | T4742_S00005.341 | tRNA modification GTPase TrmE, putative |
| OPB42343 | T4742_S00004.445 | tRNA pseudouridine synthase D |
| OPB35959 | T4742_S00025.32 | tRNA pseudouridine synthase. |
| OPB39061 | T4742_S00006.120 | tRNA pseudouridine synthase. |
| OPB43606 | T4742_S00016.126 | TRNA pseudouridine synthase. |
| OPB47053 | T4742_S00009.495 | tRNA selenocysteine-associated protein 1 |
| OPB39611 | T4742_S00006.671 | tRNA splicing endonuclease SEN15 of yeast. |
| OPB44360 | T4742_S00003.631 | tRNA(His) guanylyltransferase , putative |
| OPB37053 | T4742_S00005.80 | tRNA-dihydrouridine synthase. |
| OPB38993 | T4742_S00006.52 | tRNA-dihydrouridine synthase. |
| OPB39407 | T4742_S00006.467 | tRNA-dihydrouridine synthase. |
| OPB38498 | T4742_S00002.547 | tRNA-specific adenosine deaminase |
| OPB37529 | T4742_S00005.556 | tRNA-splicing endonuclease beta chain. |
| OPB46895 | T4742_S00009.337 | TrpD Anthranilate phosphoribosyltransferase |
| OPB42475 | T4742_S00004.577 | TrpE Anthranilate/para-aminobenzoate synthases component I |
| OPB45350 | T4742_S00010.261 | trypsin-like serine typically contains c-terminal pdz domain protein |
| OPB44879 | T4742_S00013.184 | tryptophan 2,3-dioxygenase |
| OPB43519 | T4742_S00016.39 | Tryptophan synthase |
| OPB41489 | T4742_S00011.332 | tryptophanyl-tRNA synthetase |
| OPB45939 | T4742_S00008.5 | tryptophanyl-tRNA Synthetase |
| OPB38956 | T4742_S00006.15 | Tryptophanyl-tRNA synthetase, class I of Neurospora crassa |
| OPB39631 | T4742_S00006.691 | t-SNARE Sec9; secretory vesicles-plasma membrane |
| OPB38729 | T4742_S00002.778 | t-SNARE syntaxin,cis-Golgi |
| OPB37267 | T4742_S00005.294 | t-SNARE Tlg1; fusion endosome-derived vesicles with late Golgi |
| OPB45054 | T4742_S00013.359 | t-SNARE Tlg2, endosome-derived vesicles-late Golgi |

| | | |
|---|---|---|
| OPB38335 | T4742_S00002.383 | t-SNARE Ufe1; ER membrane fusion and vesicular traffic |
| OPB43579 | T4742_S00016.99 | t-SNARE, Pep12, Golgi and vacuole |
| OPB37086 | T4742_S00005.113 | t-SNARE, SSO1 |
| OPB37841 | T4742_S00017.108 | t-SNARE, SSO2 |
| OPB39209 | T4742_S00006.269 | Tubulin alpha chain 1 |
| OPB39279 | T4742_S00006.339 | Tubulin alpha chain 1 |
| OPB39478 | T4742_S00006.538 | Tubulin beta chain 1 |
| OPB41992 | T4742_S00004.94 | Tubulin beta chain 2 |
| OPB38307 | T4742_S00002.354 | Tubulin cofactor D |
| OPB39465 | T4742_S00006.525 | Tubulin gamma chain |
| OPB36679 | T4742_S00007.397 | tubulin-specific chaperone c |
| OPB46973 | T4742_S00009.415 | tubulin-tyrosine ligase |
| OPB40084 | T4742_S00001.163 | TUL1 Golgi-localized RING-finger ubiquitin ligase (E3) |
| OPB45686 | T4742_S00014.20 | tungstate binding protein |
| OPB46621 | T4742_S00009.63 | Tup1 general transcription regulator |
| OPB39044 | T4742_S00006.103 | twinfilin-1 |
| OPB38249 | T4742_S00002.296 | Type II topoisomerase TOP2 |
| OPB36047 | T4742_S00024.8 | tyrocidine synthetase 1 [Magnaporthe oryzae P131] |
| OPB41408 | T4742_S00011.250 | Tyrosinase [Beauveria bassiana D1-5] |
| OPB46978 | T4742_S00009.420 | Tyrosinase [Beauveria bassiana D1-5] |
| OPB46608 | T4742_S00009.50 | tyrosyl-DNA phosphodiesterase, putative |
| OPB46609 | T4742_S00009.51 | tyrosyl-DNA phosphodiesterase, putative |
| OPB44451 | T4742_S00003.723 | tyrosyl-tRNA synthetase, class Ib. |
| OPB45824 | T4742_S00014.159 | tyrosyl-tRNA synthetase, class Ib. |
| OPB43099 | T4742_S00012.336 | U1 small nuclear ribonucleoprotein |
| OPB37613 | T4742_S00005.640 | U1 snRNP component |
| OPB40521 | T4742_S00001.600 | U1 snRNP splicing complex subunit (Luc7) |
| OPB38017 | T4742_S00002.64 | U2 small nuclear ribonucleoprotein A, putative |
| OPB43654 | T4742_S00016.174 | U3 small nucleolar ribonucleoprotein Mpp10 |
| OPB39759 | T4742_S00015.103 | U3 small nucleolar RNA associated protein (SOF1) |
| OPB42300 | T4742_S00004.402 | U3 snoRNA associated |
| OPB42173 | T4742_S00004.275 | U4/U6 small nuclear ribonucleoprotein Prp4 |
| OPB40189 | T4742_S00001.268 | U4/U6.U5 tri-snRNP-associated protein |
| OPB42499 | T4742_S00004.601 | U4/U6.U5 tri-snRNP-associated protein snu66 |
| OPB44201 | T4742_S00003.472 | U5 small nuclear ribonucleoprotein. Contains possible Interpro domain corresponding to elongation factor G. |
| OPB40857 | T4742_S00001.938 | U6 small nuclear ribonucleoprotein (Lsm3), putative |

| | | |
|---|---|---|
| OPB38033 | T4742_S00002.80 | U6 snRNA-associated Sm-like protein 4 |
| OPB39267 | T4742_S00006.327 | U6 snRNA-associated Sm-like protein LSm6 |
| OPB38632 | T4742_S00002.681 | U6 snRNA-associated Sm-like protein LSm7, putative |
| OPB39907 | T4742_S00015.251 | UBA/THIF-type NAD/FAD binding fold |
| OPB45131 | T4742_S00010.38 | UBA/THIF-type NAD/FAD binding fold |
| OPB36305 | T4742_S00007.22 | ubi4 ; polyubiquitin |
| OPB39652 | T4742_S00006.712 | UbiA prenyltransferase containing 9 transmembrane domains; related to S. cerevisiae Para-hydroxybenzoate-polyprenyltransferase, mitochondrial precursor |
| OPB45267 | T4742_S00010.178 | UbiA prenyltransferase, putative |
| OPB46283 | T4742_S00008.350 | UbiA prenyltransferase, putative |
| OPB46955 | T4742_S00009.397 | UbiD family decarbounknown proteinlase |
| OPB36024 | T4742_S00023.59 | UbiE/COQ5 ubiquinone methyltransferase |
| OPB47045 | T4742_S00009.487 | UbiE-like methylase |
| OPB46999 | T4742_S00009.441 | Ubiquinol cytochrome reductase |
| OPB42007 | T4742_S00004.109 | ubiquinol-cytochrome c reductase complex 17 kd protein, putative |
| OPB43984 | T4742_S00003.255 | ubiquinol-cytochrome-C oxidoreductase complex III subunit VIII |
| OPB42900 | T4742_S00012.137 | ubiquinone biosynthesis monooxygenase COQ6 |
| OPB36496 | T4742_S00007.213 | ubiquitin |
| OPB44315 | T4742_S00003.586 | ubiquitin |
| OPB45698 | T4742_S00014.32 | ubiquitin |
| OPB37274 | T4742_S00005.301 | Ubiquitin associated protein |
| OPB42297 | T4742_S00004.399 | Ubiquitin binding protein Rad23 |
| OPB45109 | T4742_S00010.16 | Ubiquitin carboxyl-terminal hydrolase |
| OPB37415 | T4742_S00005.442 | ubiquitin carboxyl-terminal hydrolase |
| OPB38642 | T4742_S00002.691 | ubiquitin carboxyl-terminal hydrolase |
| OPB41585 | T4742_S00011.428 | ubiquitin carboxyl-terminal hydrolase |
| OPB42206 | T4742_S00004.308 | ubiquitin carboxyl-terminal hydrolase |
| OPB43259 | T4742_S00022.63 | ubiquitin carboxyl-terminal hydrolase |
| OPB45031 | T4742_S00013.336 | ubiquitin carboxyl-terminal hydrolase |
| OPB39693 | T4742_S00015.37 | Ubiquitin chain assembly factor (E4) |
| OPB39694 | T4742_S00015.38 | Ubiquitin chain assembly factor (E4) |
| OPB38980 | T4742_S00006.39 | ubiquitin conjugating enzyme |
| OPB36589 | T4742_S00007.306 | Ubiquitin conjugating enzyme Ubc6 |
| OPB37275 | T4742_S00005.302 | Ubiquitin conjugating enzyme Ubc7 |
| OPB41966 | T4742_S00004.68 | Ubiquitin conjugating enzyme variant Mms2 |
| OPB42424 | T4742_S00004.526 | Ubiquitin fusion degradation protein Ufd1 |
| OPB37345 | T4742_S00005.372 | Ubiquitin interacting motif |

| | | |
|---|---|---|
| OPB42959 | T4742_S00012.196 | ubiquitin ligase |
| OPB46434 | T4742_S00008.501 | ubiquitin ligase F-Box/WD40 repeat protein FWD-1, targets the clock component FRQ for degradation |
| OPB40144 | T4742_S00001.223 | ubiquitin related modifier 1 |
| OPB41271 | T4742_S00011.112 | ubiquitin thiolesterase (OtuB1) |
| OPB47115 | T4742_S00009.557 | ubiquitin-activating enzyme UBA1 |
| OPB37005 | T4742_S00005.32 | ubiquitin-conjugating enzyme |
| OPB37093 | T4742_S00005.120 | ubiquitin-conjugating enzyme |
| OPB37344 | T4742_S00005.371 | ubiquitin-conjugating enzyme |
| OPB37853 | T4742_S00017.120 | ubiquitin-conjugating enzyme |
| OPB38418 | T4742_S00002.466 | ubiquitin-conjugating enzyme |
| OPB39000 | T4742_S00006.59 | ubiquitin-conjugating enzyme |
| OPB41607 | T4742_S00011.450 | ubiquitin-conjugating enzyme |
| OPB41975 | T4742_S00004.77 | ubiquitin-conjugating enzyme |
| OPB38393 | T4742_S00002.441 | Ubiquitin-conjugating enzyme (E2) |
| OPB38155 | T4742_S00002.202 | Ubiquitin-conjugating enzyme E2, putative |
| OPB44919 | T4742_S00013.224 | Ubiquitin-conjugating enzyme Ubc1 |
| OPB36464 | T4742_S00007.181 | Ubiquitin-conjugating enzyme, E2 |
| OPB39923 | T4742_S00001.2 | Ubiquitin-conjugating enzyme, E2 |
| OPB42648 | T4742_S00004.753 | Ubiquitin-conjugating enzyme, E2 |
| OPB39542 | T4742_S00006.602 | ubiquitin-like modifier SUMO, putative |
| OPB39383 | T4742_S00006.443 | Ubiquitin-like protein (HubA) |
| OPB42860 | T4742_S00012.97 | Ubiquitin-protein ligase (E3) |
| OPB45299 | T4742_S00010.210 | ubiquitin-protein ligase molybdopterin-converting factor |
| OPB40006 | T4742_S00001.85 | Ubiquitin-specific protease |
| OPB37041 | T4742_S00005.68 | Ubiquitin-specific protease involved in catabolite repression. Appears to be be part of a complex with creC that regulates creA |
| OPB37924 | T4742_S00017.191 | Ubitiquitin Ligase |
| OPB47050 | T4742_S00009.492 | Ubitiquitin Ligase |
| OPB45381 | T4742_S00010.292 | UDP glucose-4-epimerase |
| OPB43194 | T4742_S00012.431 | UDP-galactopyranose mutase |
| OPB42268 | T4742_S00004.370 | UDP-galactose transporter |
| OPB41613 | T4742_S00011.456 | UDP-GlcNAc:phosphatidylinositol acetylglucosaminyltransferase complex member |
| OPB37528 | T4742_S00005.555 | UDP-glucose ceramide glucosyltransferase |
| OPB37748 | T4742_S00017.15 | UDP-glucose glycoprotein a-glucosyltransferase |
| OPB37870 | T4742_S00017.137 | UDP-glucose:sterol b-glucosyltransferase |
| OPB40409 | T4742_S00001.488 | UDP-glucose-6-dehydrogenase |
| OPB46663 | T4742_S00009.105 | UDP-glucosyl transferase |

| | | |
|---|---|---|
| OPB41249 | T4742_S00011.90 | UDP-glucuronosyl and UDP-glucosyl transferase |
| OPB36448 | T4742_S00007.165 | UDP-glucuronosyl/UDP-glucosyltransferase |
| OPB44309 | T4742_S00003.580 | UDP-N-acetylglucosamine pyrophosphorylase |
| OPB40233 | T4742_S00001.312 | UDP-N-acetylglucosamine transferase subunit |
| OPB43510 | T4742_S00016.30 | UDP-N-acetylglucosamine transporter |
| OPB43835 | T4742_S00003.106 | Ume5 CMGC kinase |
| OPB38917 | T4742_S00002.966 | unique (orphan) protein |
| OPB40454 | T4742_S00001.533 | unique (orphan) protein |
| OPB40687 | T4742_S00001.767 | unique (orphan) protein |
| OPB40880 | T4742_S00001.961 | unique (orphan) protein |
| OPB40637 | T4742_S00001.717 | unique protein |
| OPB41977 | T4742_S00004.79 | uniquitin-like autophagy protein Apg12 |
| OPB36678 | T4742_S00007.396 | unknown  protein |
| OPB37541 | T4742_S00005.568 | unknown  protein |
| OPB38785 | T4742_S00002.834 | unknown  protein |
| OPB38897 | T4742_S00002.946 | unknown  protein |
| OPB39151 | T4742_S00006.211 | unknown  protein |
| OPB39322 | T4742_S00006.382 | unknown  protein |
| OPB40577 | T4742_S00001.657 | unknown  protein |
| OPB43775 | T4742_S00003.46 | unknown  protein |
| OPB43828 | T4742_S00003.99 | unknown  protein |
| OPB44178 | T4742_S00003.449 | unknown  protein |
| OPB45823 | T4742_S00014.158 | unknown  protein |
| OPB45863 | T4742_S00014.198 | unknown  protein |
| OPB46108 | T4742_S00008.175 | unknown  protein |
| OPB46788 | T4742_S00009.230 | unknown  protein |
| OPB35925 | T4742_S00027.6 | unknown protein |
| OPB35927 | T4742_S00029.2 | unknown protein |
| OPB35928 | T4742_S00025.1 | unknown protein |
| OPB35933 | T4742_S00025.6 | unknown protein |
| OPB35934 | T4742_S00025.7 | unknown protein |
| OPB35936 | T4742_S00025.9 | unknown protein |
| OPB35940 | T4742_S00025.13 | unknown protein |
| OPB35943 | T4742_S00025.16 | unknown protein |
| OPB35944 | T4742_S00025.17 | unknown protein |
| OPB35945 | T4742_S00025.18 | unknown protein |

| | | |
|---|---|---|
| OPB35946 | T4742_S00025.19 | unknown protein |
| OPB35947 | T4742_S00025.20 | unknown protein |
| OPB35948 | T4742_S00025.21 | unknown protein |
| OPB35954 | T4742_S00025.27 | unknown protein |
| OPB35955 | T4742_S00025.28 | unknown protein |
| OPB35956 | T4742_S00025.29 | unknown protein |
| OPB35957 | T4742_S00025.30 | unknown protein |
| OPB35962 | T4742_S00025.35 | unknown protein |
| OPB35964 | T4742_S00025.37 | unknown protein |
| OPB35966 | T4742_S00023.1 | unknown protein |
| OPB35971 | T4742_S00023.6 | unknown protein |
| OPB35972 | T4742_S00023.7 | unknown protein |
| OPB35973 | T4742_S00023.8 | unknown protein |
| OPB35974 | T4742_S00023.9 | unknown protein |
| OPB35976 | T4742_S00023.11 | unknown protein |
| OPB35977 | T4742_S00023.12 | unknown protein |
| OPB35981 | T4742_S00023.16 | unknown protein |
| OPB35982 | T4742_S00023.17 | unknown protein |
| OPB35983 | T4742_S00023.18 | unknown protein |
| OPB35986 | T4742_S00023.21 | unknown protein |
| OPB35987 | T4742_S00023.22 | unknown protein |
| OPB35989 | T4742_S00023.24 | unknown protein |
| OPB35990 | T4742_S00023.25 | unknown protein |
| OPB35992 | T4742_S00023.27 | unknown protein |
| OPB35999 | T4742_S00023.34 | unknown protein |
| OPB36007 | T4742_S00023.42 | unknown protein |
| OPB36016 | T4742_S00023.51 | unknown protein |
| OPB36018 | T4742_S00023.53 | unknown protein |
| OPB36019 | T4742_S00023.54 | unknown protein |
| OPB36020 | T4742_S00023.55 | unknown protein |
| OPB36037 | T4742_S00023.72 | unknown protein |
| OPB36038 | T4742_S00023.73 | unknown protein |
| OPB36040 | T4742_S00024.1 | unknown protein |
| OPB36041 | T4742_S00024.2 | unknown protein |
| OPB36046 | T4742_S00024.7 | unknown protein |
| OPB36064 | T4742_S00024.25 | unknown protein |

| | | |
|---|---|---|
| OPB36066 | T4742_S00024.27 | unknown protein |
| OPB36068 | T4742_S00024.29 | unknown protein |
| OPB36073 | T4742_S00024.34 | unknown protein |
| OPB36096 | T4742_S00021.12 | unknown protein |
| OPB36115 | T4742_S00021.32 | unknown protein |
| OPB36126 | T4742_S00021.43 | unknown protein |
| OPB36133 | T4742_S00021.50 | unknown protein |
| OPB36139 | T4742_S00021.56 | unknown protein |
| OPB36144 | T4742_S00021.61 | unknown protein |
| OPB36148 | T4742_S00021.65 | unknown protein |
| OPB36156 | T4742_S00021.73 | unknown protein |
| OPB36172 | T4742_S00021.89 | unknown protein |
| OPB36174 | T4742_S00021.92 | unknown protein |
| OPB36183 | T4742_S00021.101 | unknown protein |
| OPB36188 | T4742_S00021.106 | unknown protein |
| OPB36191 | T4742_S00021.109 | unknown protein |
| OPB36192 | T4742_S00021.110 | unknown protein |
| OPB36195 | T4742_S00021.113 | unknown protein |
| OPB36201 | T4742_S00021.119 | unknown protein |
| OPB36210 | T4742_S00021.128 | unknown protein |
| OPB36215 | T4742_S00021.133 | unknown protein |
| OPB36223 | T4742_S00021.141 | unknown protein |
| OPB36224 | T4742_S00021.142 | unknown protein |
| OPB36228 | T4742_S00021.146 | unknown protein |
| OPB36235 | T4742_S00021.153 | unknown protein |
| OPB36238 | T4742_S00021.156 | unknown protein |
| OPB36246 | T4742_S00021.164 | unknown protein |
| OPB36251 | T4742_S00021.169 | unknown protein |
| OPB36254 | T4742_S00021.172 | unknown protein |
| OPB36257 | T4742_S00021.175 | unknown protein |
| OPB36279 | T4742_S00021.198 | unknown protein |
| OPB36288 | T4742_S00007.5 | unknown protein |
| OPB36297 | T4742_S00007.14 | unknown protein |
| OPB36299 | T4742_S00007.16 | unknown protein |
| OPB36302 | T4742_S00007.19 | unknown protein |
| OPB36306 | T4742_S00007.23 | unknown protein |

| | | |
|---|---|---|
| OPB36310 | T4742_S00007.27 | unknown protein |
| OPB36312 | T4742_S00007.29 | unknown protein |
| OPB36319 | T4742_S00007.36 | unknown protein |
| OPB36326 | T4742_S00007.43 | unknown protein |
| OPB36333 | T4742_S00007.50 | unknown protein |
| OPB36335 | T4742_S00007.52 | unknown protein |
| OPB36336 | T4742_S00007.53 | unknown protein |
| OPB36339 | T4742_S00007.56 | unknown protein |
| OPB36343 | T4742_S00007.60 | unknown protein |
| OPB36346 | T4742_S00007.63 | unknown protein |
| OPB36348 | T4742_S00007.65 | unknown protein |
| OPB36351 | T4742_S00007.68 | unknown protein |
| OPB36363 | T4742_S00007.80 | unknown protein |
| OPB36364 | T4742_S00007.81 | unknown protein |
| OPB36376 | T4742_S00007.93 | unknown protein |
| OPB36378 | T4742_S00007.95 | unknown protein |
| OPB36380 | T4742_S00007.97 | unknown protein |
| OPB36383 | T4742_S00007.100 | unknown protein |
| OPB36386 | T4742_S00007.103 | unknown protein |
| OPB36387 | T4742_S00007.104 | unknown protein |
| OPB36393 | T4742_S00007.110 | unknown protein |
| OPB36395 | T4742_S00007.112 | unknown protein |
| OPB36398 | T4742_S00007.115 | unknown protein |
| OPB36400 | T4742_S00007.117 | unknown protein |
| OPB36403 | T4742_S00007.120 | unknown protein |
| OPB36405 | T4742_S00007.122 | unknown protein |
| OPB36407 | T4742_S00007.124 | unknown protein |
| OPB36416 | T4742_S00007.133 | unknown protein |
| OPB36417 | T4742_S00007.134 | unknown protein |
| OPB36425 | T4742_S00007.142 | unknown protein |
| OPB36428 | T4742_S00007.145 | unknown protein |
| OPB36447 | T4742_S00007.164 | unknown protein |
| OPB36450 | T4742_S00007.167 | unknown protein |
| OPB36453 | T4742_S00007.170 | unknown protein |
| OPB36456 | T4742_S00007.173 | unknown protein |
| OPB36458 | T4742_S00007.175 | unknown protein |

| | | |
|---|---|---|
| OPB36462 | T4742_S00007.179 | unknown protein |
| OPB36473 | T4742_S00007.190 | unknown protein |
| OPB36474 | T4742_S00007.191 | unknown protein |
| OPB36475 | T4742_S00007.192 | unknown protein |
| OPB36476 | T4742_S00007.193 | unknown protein |
| OPB36480 | T4742_S00007.197 | unknown protein |
| OPB36481 | T4742_S00007.198 | unknown protein |
| OPB36484 | T4742_S00007.201 | unknown protein |
| OPB36490 | T4742_S00007.207 | unknown protein |
| OPB36491 | T4742_S00007.208 | unknown protein |
| OPB36492 | T4742_S00007.209 | unknown protein |
| OPB36494 | T4742_S00007.211 | unknown protein |
| OPB36503 | T4742_S00007.220 | unknown protein |
| OPB36504 | T4742_S00007.221 | unknown protein |
| OPB36505 | T4742_S00007.222 | unknown protein |
| OPB36507 | T4742_S00007.224 | unknown protein |
| OPB36508 | T4742_S00007.225 | unknown protein |
| OPB36511 | T4742_S00007.228 | unknown protein |
| OPB36518 | T4742_S00007.235 | unknown protein |
| OPB36519 | T4742_S00007.236 | unknown protein |
| OPB36520 | T4742_S00007.237 | unknown protein |
| OPB36526 | T4742_S00007.243 | unknown protein |
| OPB36529 | T4742_S00007.246 | unknown protein |
| OPB36532 | T4742_S00007.249 | unknown protein |
| OPB36533 | T4742_S00007.250 | unknown protein |
| OPB36535 | T4742_S00007.252 | unknown protein |
| OPB36538 | T4742_S00007.255 | unknown protein |
| OPB36539 | T4742_S00007.256 | unknown protein |
| OPB36540 | T4742_S00007.257 | unknown protein |
| OPB36541 | T4742_S00007.258 | unknown protein |
| OPB36550 | T4742_S00007.267 | unknown protein |
| OPB36552 | T4742_S00007.269 | unknown protein |
| OPB36555 | T4742_S00007.272 | unknown protein |
| OPB36563 | T4742_S00007.280 | unknown protein |
| OPB36566 | T4742_S00007.283 | unknown protein |
| OPB36579 | T4742_S00007.296 | unknown protein |

| | | |
|---|---|---|
| OPB36580 | T4742_S00007.297 | unknown protein |
| OPB36581 | T4742_S00007.298 | unknown protein |
| OPB36584 | T4742_S00007.301 | unknown protein |
| OPB36585 | T4742_S00007.302 | unknown protein |
| OPB36587 | T4742_S00007.304 | unknown protein |
| OPB36588 | T4742_S00007.305 | unknown protein |
| OPB36592 | T4742_S00007.309 | unknown protein |
| OPB36593 | T4742_S00007.310 | unknown protein |
| OPB36594 | T4742_S00007.311 | unknown protein |
| OPB36596 | T4742_S00007.313 | unknown protein |
| OPB36600 | T4742_S00007.318 | unknown protein |
| OPB36601 | T4742_S00007.319 | unknown protein |
| OPB36602 | T4742_S00007.320 | unknown protein |
| OPB36604 | T4742_S00007.322 | unknown protein |
| OPB36611 | T4742_S00007.329 | unknown protein |
| OPB36614 | T4742_S00007.332 | unknown protein |
| OPB36616 | T4742_S00007.334 | unknown protein |
| OPB36622 | T4742_S00007.340 | unknown protein |
| OPB36624 | T4742_S00007.342 | unknown protein |
| OPB36626 | T4742_S00007.344 | unknown protein |
| OPB36628 | T4742_S00007.346 | unknown protein |
| OPB36634 | T4742_S00007.352 | unknown protein |
| OPB36635 | T4742_S00007.353 | unknown protein |
| OPB36638 | T4742_S00007.356 | unknown protein |
| OPB36647 | T4742_S00007.365 | unknown protein |
| OPB36648 | T4742_S00007.366 | unknown protein |
| OPB36654 | T4742_S00007.372 | unknown protein |
| OPB36655 | T4742_S00007.373 | unknown protein |
| OPB36658 | T4742_S00007.376 | unknown protein |
| OPB36663 | T4742_S00007.381 | unknown protein |
| OPB36664 | T4742_S00007.382 | unknown protein |
| OPB36665 | T4742_S00007.383 | unknown protein |
| OPB36670 | T4742_S00007.388 | unknown protein |
| OPB36672 | T4742_S00007.390 | unknown protein |
| OPB36675 | T4742_S00007.393 | unknown protein |
| OPB36676 | T4742_S00007.394 | unknown protein |

| | | |
|---|---|---|
| OPB36680 | T4742_S00007.398 | unknown protein |
| OPB36687 | T4742_S00007.405 | unknown protein |
| OPB36688 | T4742_S00007.406 | unknown protein |
| OPB36690 | T4742_S00007.408 | unknown protein |
| OPB36692 | T4742_S00007.410 | unknown protein |
| OPB36693 | T4742_S00007.411 | unknown protein |
| OPB36696 | T4742_S00007.414 | unknown protein |
| OPB36697 | T4742_S00007.415 | unknown protein |
| OPB36699 | T4742_S00007.417 | unknown protein |
| OPB36700 | T4742_S00007.418 | unknown protein |
| OPB36701 | T4742_S00007.419 | unknown protein |
| OPB36703 | T4742_S00007.421 | unknown protein |
| OPB36704 | T4742_S00007.422 | unknown protein |
| OPB36706 | T4742_S00007.424 | unknown protein |
| OPB36708 | T4742_S00007.426 | unknown protein |
| OPB36710 | T4742_S00007.428 | unknown protein |
| OPB36718 | T4742_S00007.436 | unknown protein |
| OPB36719 | T4742_S00007.437 | unknown protein |
| OPB36720 | T4742_S00007.438 | unknown protein |
| OPB36722 | T4742_S00007.440 | unknown protein |
| OPB36727 | T4742_S00007.445 | unknown protein |
| OPB36728 | T4742_S00007.446 | unknown protein |
| OPB36731 | T4742_S00007.449 | unknown protein |
| OPB36732 | T4742_S00007.450 | unknown protein |
| OPB36734 | T4742_S00007.452 | unknown protein |
| OPB36737 | T4742_S00007.455 | unknown protein |
| OPB36739 | T4742_S00007.457 | unknown protein |
| OPB36740 | T4742_S00007.458 | unknown protein |
| OPB36743 | T4742_S00007.461 | unknown protein |
| OPB36746 | T4742_S00007.464 | unknown protein |
| OPB36748 | T4742_S00007.466 | unknown protein |
| OPB36750 | T4742_S00007.468 | unknown protein |
| OPB36751 | T4742_S00007.469 | unknown protein |
| OPB36753 | T4742_S00007.471 | unknown protein |
| OPB36754 | T4742_S00007.472 | unknown protein |
| OPB36756 | T4742_S00007.474 | unknown protein |

| | | |
|---|---|---|
| OPB36766 | T4742_S00007.484 | unknown protein |
| OPB36767 | T4742_S00007.485 | unknown protein |
| OPB36768 | T4742_S00007.486 | unknown protein |
| OPB36771 | T4742_S00007.489 | unknown protein |
| OPB36773 | T4742_S00007.491 | unknown protein |
| OPB36774 | T4742_S00007.492 | unknown protein |
| OPB36791 | T4742_S00007.509 | unknown protein |
| OPB36806 | T4742_S00007.524 | unknown protein |
| OPB36808 | T4742_S00007.526 | unknown protein |
| OPB36809 | T4742_S00007.527 | unknown protein |
| OPB36813 | T4742_S00007.531 | unknown protein |
| OPB36819 | T4742_S00007.537 | unknown protein |
| OPB36833 | T4742_S00007.551 | unknown protein |
| OPB36838 | T4742_S00007.556 | unknown protein |
| OPB36839 | T4742_S00007.557 | unknown protein |
| OPB36845 | T4742_S00007.563 | unknown protein |
| OPB36851 | T4742_S00007.569 | unknown protein |
| OPB36852 | T4742_S00007.570 | unknown protein |
| OPB36857 | T4742_S00007.575 | unknown protein |
| OPB36870 | T4742_S00007.588 | unknown protein |
| OPB36871 | T4742_S00007.589 | unknown protein |
| OPB36874 | T4742_S00007.592 | unknown protein |
| OPB36876 | T4742_S00007.594 | unknown protein |
| OPB36880 | T4742_S00007.598 | unknown protein |
| OPB36882 | T4742_S00007.600 | unknown protein |
| OPB36887 | T4742_S00007.605 | unknown protein |
| OPB36888 | T4742_S00007.606 | unknown protein |
| OPB36889 | T4742_S00007.607 | Unknown protein |
| OPB36890 | T4742_S00007.608 | unknown protein |
| OPB36892 | T4742_S00007.610 | unknown protein |
| OPB36893 | T4742_S00007.611 | unknown protein |
| OPB36894 | T4742_S00007.612 | unknown protein |
| OPB36895 | T4742_S00007.613 | unknown protein |
| OPB36897 | T4742_S00007.615 | unknown protein |
| OPB36899 | T4742_S00007.617 | unknown protein |
| OPB36901 | T4742_S00007.619 | unknown protein |

| | | |
|---|---|---|
| OPB36902 | T4742_S00007.620 | unknown protein |
| OPB36903 | T4742_S00007.621 | unknown protein |
| OPB36905 | T4742_S00007.623 | unknown protein |
| OPB36908 | T4742_S00007.626 | unknown protein |
| OPB36910 | T4742_S00007.628 | unknown protein |
| OPB36913 | T4742_S00007.631 | unknown protein |
| OPB36915 | T4742_S00007.633 | unknown protein |
| OPB36916 | T4742_S00007.634 | unknown protein |
| OPB36918 | T4742_S00007.636 | unknown protein |
| OPB36923 | T4742_S00007.641 | unknown protein |
| OPB36926 | T4742_S00007.644 | unknown protein |
| OPB36927 | T4742_S00007.645 | unknown protein |
| OPB36930 | T4742_S00007.648 | unknown protein |
| OPB36937 | T4742_S00007.655 | unknown protein |
| OPB36940 | T4742_S00007.658 | unknown protein |
| OPB36947 | T4742_S00007.665 | unknown protein |
| OPB36948 | T4742_S00007.666 | unknown protein |
| OPB36949 | T4742_S00007.667 | unknown protein |
| OPB36950 | T4742_S00007.668 | unknown protein |
| OPB36965 | T4742_S00007.683 | unknown protein |
| OPB36967 | T4742_S00007.685 | unknown protein |
| OPB36974 | T4742_S00005.1 | unknown protein |
| OPB36976 | T4742_S00005.3 | unknown protein |
| OPB36977 | T4742_S00005.4 | unknown protein |
| OPB36978 | T4742_S00005.5 | unknown protein |
| OPB36979 | T4742_S00005.6 | unknown protein |
| OPB36980 | T4742_S00005.7 | unknown protein |
| OPB36987 | T4742_S00005.14 | unknown protein |
| OPB36993 | T4742_S00005.20 | unknown protein |
| OPB36994 | T4742_S00005.21 | unknown protein |
| OPB36999 | T4742_S00005.26 | unknown protein |
| OPB37004 | T4742_S00005.31 | unknown protein |
| OPB37007 | T4742_S00005.34 | unknown protein |
| OPB37010 | T4742_S00005.37 | unknown protein |
| OPB37011 | T4742_S00005.38 | unknown protein |
| OPB37015 | T4742_S00005.42 | unknown protein |

| | | |
|---|---|---|
| OPB37029 | T4742_S00005.56 | unknown protein |
| OPB37033 | T4742_S00005.60 | unknown protein |
| OPB37035 | T4742_S00005.62 | unknown protein |
| OPB37037 | T4742_S00005.64 | unknown protein |
| OPB37038 | T4742_S00005.65 | unknown protein |
| OPB37051 | T4742_S00005.78 | unknown protein |
| OPB37052 | T4742_S00005.79 | unknown protein |
| OPB37062 | T4742_S00005.89 | unknown protein |
| OPB37063 | T4742_S00005.90 | unknown protein |
| OPB37069 | T4742_S00005.96 | unknown protein |
| OPB37077 | T4742_S00005.104 | unknown protein |
| OPB37080 | T4742_S00005.107 | unknown protein |
| OPB37083 | T4742_S00005.110 | unknown protein |
| OPB37095 | T4742_S00005.122 | unknown protein |
| OPB37101 | T4742_S00005.128 | unknown protein |
| OPB37102 | T4742_S00005.129 | unknown protein |
| OPB37103 | T4742_S00005.130 | unknown protein |
| OPB37105 | T4742_S00005.132 | unknown protein |
| OPB37106 | T4742_S00005.133 | unknown protein |
| OPB37108 | T4742_S00005.135 | unknown protein |
| OPB37113 | T4742_S00005.140 | unknown protein |
| OPB37121 | T4742_S00005.148 | unknown protein |
| OPB37122 | T4742_S00005.149 | unknown protein |
| OPB37123 | T4742_S00005.150 | unknown protein |
| OPB37124 | T4742_S00005.151 | unknown protein |
| OPB37128 | T4742_S00005.155 | unknown protein |
| OPB37129 | T4742_S00005.156 | unknown protein |
| OPB37131 | T4742_S00005.158 | unknown protein |
| OPB37132 | T4742_S00005.159 | unknown protein |
| OPB37134 | T4742_S00005.161 | unknown protein |
| OPB37141 | T4742_S00005.168 | unknown protein |
| OPB37143 | T4742_S00005.170 | unknown protein |
| OPB37146 | T4742_S00005.173 | unknown protein |
| OPB37149 | T4742_S00005.176 | unknown protein |
| OPB37152 | T4742_S00005.179 | unknown protein |
| OPB37154 | T4742_S00005.181 | unknown protein |

| | | |
|---|---|---|
| OPB37155 | T4742_S00005.182 | unknown protein |
| OPB37163 | T4742_S00005.190 | unknown protein |
| OPB37164 | T4742_S00005.191 | unknown protein |
| OPB37165 | T4742_S00005.192 | unknown protein |
| OPB37169 | T4742_S00005.196 | unknown protein |
| OPB37170 | T4742_S00005.197 | unknown protein |
| OPB37174 | T4742_S00005.201 | unknown protein |
| OPB37181 | T4742_S00005.208 | unknown protein |
| OPB37183 | T4742_S00005.210 | unknown protein |
| OPB37184 | T4742_S00005.211 | unknown protein |
| OPB37187 | T4742_S00005.214 | unknown protein |
| OPB37191 | T4742_S00005.218 | unknown protein |
| OPB37197 | T4742_S00005.224 | unknown protein |
| OPB37203 | T4742_S00005.230 | unknown protein |
| OPB37204 | T4742_S00005.231 | unknown protein |
| OPB37214 | T4742_S00005.241 | unknown protein |
| OPB37215 | T4742_S00005.242 | unknown protein |
| OPB37217 | T4742_S00005.244 | unknown protein |
| OPB37222 | T4742_S00005.249 | unknown protein |
| OPB37226 | T4742_S00005.253 | unknown protein |
| OPB37227 | T4742_S00005.254 | unknown protein |
| OPB37228 | T4742_S00005.255 | unknown protein |
| OPB37234 | T4742_S00005.261 | unknown protein |
| OPB37235 | T4742_S00005.262 | unknown protein |
| OPB37239 | T4742_S00005.266 | unknown protein |
| OPB37252 | T4742_S00005.279 | unknown protein |
| OPB37254 | T4742_S00005.281 | unknown protein |
| OPB37255 | T4742_S00005.282 | unknown protein |
| OPB37259 | T4742_S00005.286 | unknown protein |
| OPB37260 | T4742_S00005.287 | unknown protein |
| OPB37263 | T4742_S00005.290 | Unknown protein |
| OPB37264 | T4742_S00005.291 | unknown protein |
| OPB37268 | T4742_S00005.295 | unknown protein |
| OPB37269 | T4742_S00005.296 | unknown protein |
| OPB37270 | T4742_S00005.297 | unknown protein |
| OPB37271 | T4742_S00005.298 | unknown protein |

| | | |
|---|---|---|
| OPB37273 | T4742_S00005.300 | unknown protein |
| OPB37280 | T4742_S00005.307 | unknown protein |
| OPB37281 | T4742_S00005.308 | unknown protein |
| OPB37283 | T4742_S00005.310 | unknown protein |
| OPB37287 | T4742_S00005.314 | unknown protein |
| OPB37292 | T4742_S00005.319 | unknown protein |
| OPB37295 | T4742_S00005.322 | unknown protein |
| OPB37298 | T4742_S00005.325 | unknown protein |
| OPB37305 | T4742_S00005.332 | unknown protein |
| OPB37306 | T4742_S00005.333 | unknown protein |
| OPB37310 | T4742_S00005.337 | unknown protein |
| OPB37311 | T4742_S00005.338 | unknown protein |
| OPB37313 | T4742_S00005.340 | unknown protein |
| OPB37322 | T4742_S00005.349 | unknown protein |
| OPB37327 | T4742_S00005.354 | unknown protein |
| OPB37332 | T4742_S00005.359 | unknown protein |
| OPB37333 | T4742_S00005.360 | unknown protein |
| OPB37335 | T4742_S00005.362 | unknown protein |
| OPB37338 | T4742_S00005.365 | unknown protein |
| OPB37342 | T4742_S00005.369 | unknown protein |
| OPB37354 | T4742_S00005.381 | unknown protein |
| OPB37355 | T4742_S00005.382 | unknown protein |
| OPB37357 | T4742_S00005.384 | unknown protein |
| OPB37359 | T4742_S00005.386 | unknown protein |
| OPB37361 | T4742_S00005.388 | unknown protein |
| OPB37362 | T4742_S00005.389 | unknown protein |
| OPB37366 | T4742_S00005.393 | unknown protein |
| OPB37371 | T4742_S00005.398 | unknown protein |
| OPB37379 | T4742_S00005.406 | unknown protein |
| OPB37387 | T4742_S00005.414 | unknown protein |
| OPB37388 | T4742_S00005.415 | unknown protein |
| OPB37389 | T4742_S00005.416 | unknown protein |
| OPB37392 | T4742_S00005.419 | unknown protein |
| OPB37393 | T4742_S00005.420 | unknown protein |
| OPB37396 | T4742_S00005.423 | unknown protein |
| OPB37400 | T4742_S00005.427 | unknown protein |

| | | |
|---|---|---|
| OPB37401 | T4742_S00005.428 | unknown protein |
| OPB37405 | T4742_S00005.432 | unknown protein |
| OPB37410 | T4742_S00005.437 | unknown protein |
| OPB37412 | T4742_S00005.439 | unknown protein |
| OPB37414 | T4742_S00005.441 | Unknown protein |
| OPB37416 | T4742_S00005.443 | unknown protein |
| OPB37426 | T4742_S00005.453 | unknown protein |
| OPB37428 | T4742_S00005.455 | unknown protein |
| OPB37436 | T4742_S00005.463 | unknown protein |
| OPB37438 | T4742_S00005.465 | unknown protein |
| OPB37439 | T4742_S00005.466 | unknown protein |
| OPB37446 | T4742_S00005.473 | unknown protein |
| OPB37448 | T4742_S00005.475 | unknown protein |
| OPB37449 | T4742_S00005.476 | unknown protein |
| OPB37450 | T4742_S00005.477 | unknown protein |
| OPB37453 | T4742_S00005.480 | unknown protein |
| OPB37454 | T4742_S00005.481 | unknown protein |
| OPB37457 | T4742_S00005.484 | unknown protein |
| OPB37459 | T4742_S00005.486 | unknown protein |
| OPB37461 | T4742_S00005.488 | unknown protein |
| OPB37465 | T4742_S00005.492 | unknown protein |
| OPB37467 | T4742_S00005.494 | unknown protein |
| OPB37471 | T4742_S00005.498 | unknown protein |
| OPB37475 | T4742_S00005.502 | unknown protein |
| OPB37478 | T4742_S00005.505 | unknown protein |
| OPB37480 | T4742_S00005.507 | unknown protein |
| OPB37481 | T4742_S00005.508 | unknown protein |
| OPB37482 | T4742_S00005.509 | unknown protein |
| OPB37483 | T4742_S00005.510 | unknown protein |
| OPB37484 | T4742_S00005.511 | unknown protein |
| OPB37490 | T4742_S00005.517 | unknown protein |
| OPB37492 | T4742_S00005.519 | unknown protein |
| OPB37493 | T4742_S00005.520 | unknown protein |
| OPB37497 | T4742_S00005.524 | unknown protein |
| OPB37500 | T4742_S00005.527 | unknown protein |
| OPB37501 | T4742_S00005.528 | unknown protein |

| | | |
|---|---|---|
| OPB37506 | T4742_S00005.533 | unknown protein |
| OPB37510 | T4742_S00005.537 | unknown protein |
| OPB37517 | T4742_S00005.544 | unknown protein |
| OPB37520 | T4742_S00005.547 | unknown protein |
| OPB37523 | T4742_S00005.550 | unknown protein |
| OPB37526 | T4742_S00005.553 | unknown protein |
| OPB37531 | T4742_S00005.558 | unknown protein |
| OPB37535 | T4742_S00005.562 | unknown protein |
| OPB37550 | T4742_S00005.577 | unknown protein |
| OPB37551 | T4742_S00005.578 | unknown protein |
| OPB37559 | T4742_S00005.586 | unknown protein |
| OPB37562 | T4742_S00005.589 | unknown protein |
| OPB37563 | T4742_S00005.590 | unknown protein |
| OPB37566 | T4742_S00005.593 | unknown protein |
| OPB37571 | T4742_S00005.598 | unknown protein |
| OPB37577 | T4742_S00005.604 | unknown protein |
| OPB37580 | T4742_S00005.607 | unknown protein |
| OPB37593 | T4742_S00005.620 | unknown protein |
| OPB37595 | T4742_S00005.622 | unknown protein |
| OPB37596 | T4742_S00005.623 | unknown protein |
| OPB37599 | T4742_S00005.626 | unknown protein |
| OPB37608 | T4742_S00005.635 | unknown protein |
| OPB37610 | T4742_S00005.637 | unknown protein |
| OPB37611 | T4742_S00005.638 | unknown protein |
| OPB37624 | T4742_S00005.651 | unknown protein |
| OPB37626 | T4742_S00005.653 | unknown protein |
| OPB37632 | T4742_S00005.659 | unknown protein |
| OPB37633 | T4742_S00005.660 | unknown protein |
| OPB37649 | T4742_S00005.676 | unknown protein |
| OPB37656 | T4742_S00005.683 | unknown protein |
| OPB37666 | T4742_S00005.693 | unknown protein |
| OPB37672 | T4742_S00005.699 | unknown protein |
| OPB37674 | T4742_S00005.701 | unknown protein |
| OPB37675 | T4742_S00005.702 | unknown protein |
| OPB37677 | T4742_S00005.704 | unknown protein |
| OPB37692 | T4742_S00005.719 | Unknown protein |

| | | |
|---|---|---|
| OPB37703 | T4742_S00005.731 | unknown protein |
| OPB37705 | T4742_S00005.733 | unknown protein |
| OPB37710 | T4742_S00005.738 | unknown protein |
| OPB37723 | T4742_S00005.751 | unknown protein |
| OPB37725 | T4742_S00005.753 | unknown protein |
| OPB37735 | T4742_S00017.2 | unknown protein |
| OPB37739 | T4742_S00017.6 | unknown protein |
| OPB37743 | T4742_S00017.10 | unknown protein |
| OPB37756 | T4742_S00017.23 | unknown protein |
| OPB37758 | T4742_S00017.25 | unknown protein |
| OPB37770 | T4742_S00017.37 | unknown protein |
| OPB37772 | T4742_S00017.39 | unknown protein |
| OPB37774 | T4742_S00017.41 | unknown protein |
| OPB37777 | T4742_S00017.44 | unknown protein |
| OPB37785 | T4742_S00017.52 | unknown protein |
| OPB37786 | T4742_S00017.53 | unknown protein |
| OPB37787 | T4742_S00017.54 | unknown protein |
| OPB37793 | T4742_S00017.60 | unknown protein |
| OPB37795 | T4742_S00017.62 | unknown protein |
| OPB37797 | T4742_S00017.64 | unknown protein |
| OPB37799 | T4742_S00017.66 | unknown protein |
| OPB37800 | T4742_S00017.67 | unknown protein |
| OPB37812 | T4742_S00017.79 | unknown protein |
| OPB37820 | T4742_S00017.87 | unknown protein |
| OPB37824 | T4742_S00017.91 | unknown protein |
| OPB37825 | T4742_S00017.92 | unknown protein |
| OPB37829 | T4742_S00017.96 | unknown protein |
| OPB37835 | T4742_S00017.102 | unknown protein |
| OPB37836 | T4742_S00017.103 | unknown protein |
| OPB37837 | T4742_S00017.104 | unknown protein |
| OPB37840 | T4742_S00017.107 | unknown protein |
| OPB37842 | T4742_S00017.109 | unknown protein |
| OPB37843 | T4742_S00017.110 | unknown protein |
| OPB37845 | T4742_S00017.112 | unknown protein |
| OPB37846 | T4742_S00017.113 | unknown protein |
| OPB37847 | T4742_S00017.114 | unknown protein |

| | | |
|---|---|---|
| OPB37848 | T4742_S00017.115 | Unknown protein |
| OPB37856 | T4742_S00017.123 | unknown protein |
| OPB37857 | T4742_S00017.124 | unknown protein |
| OPB37858 | T4742_S00017.125 | unknown protein |
| OPB37864 | T4742_S00017.131 | unknown protein |
| OPB37865 | T4742_S00017.132 | unknown protein |
| OPB37868 | T4742_S00017.135 | unknown protein |
| OPB37869 | T4742_S00017.136 | unknown protein |
| OPB37875 | T4742_S00017.142 | unknown protein |
| OPB37876 | T4742_S00017.143 | unknown protein |
| OPB37886 | T4742_S00017.153 | unknown protein |
| OPB37890 | T4742_S00017.157 | unknown protein |
| OPB37891 | T4742_S00017.158 | unknown protein |
| OPB37895 | T4742_S00017.162 | unknown protein |
| OPB37896 | T4742_S00017.163 | unknown protein |
| OPB37899 | T4742_S00017.166 | unknown protein |
| OPB37900 | T4742_S00017.167 | unknown protein |
| OPB37903 | T4742_S00017.170 | unknown protein |
| OPB37904 | T4742_S00017.171 | unknown protein |
| OPB37909 | T4742_S00017.176 | unknown protein |
| OPB37916 | T4742_S00017.183 | unknown protein |
| OPB37920 | T4742_S00017.187 | unknown protein |
| OPB37922 | T4742_S00017.189 | unknown protein |
| OPB37926 | T4742_S00017.193 | unknown protein |
| OPB37943 | T4742_S00017.210 | unknown protein |
| OPB37949 | T4742_S00017.216 | unknown protein |
| OPB37954 | T4742_S00002.1 | unknown protein |
| OPB37955 | T4742_S00002.2 | unknown protein |
| OPB37968 | T4742_S00002.15 | unknown protein |
| OPB37984 | T4742_S00002.31 | unknown protein |
| OPB37991 | T4742_S00002.38 | unknown protein |
| OPB37993 | T4742_S00002.40 | unknown protein |
| OPB38000 | T4742_S00002.47 | unknown protein |
| OPB38001 | T4742_S00002.48 | unknown protein |
| OPB38004 | T4742_S00002.51 | unknown protein |
| OPB38005 | T4742_S00002.52 | unknown protein |

| | | |
|---|---|---|
| OPB38009 | T4742_S00002.56 | unknown protein |
| OPB38014 | T4742_S00002.61 | unknown protein |
| OPB38015 | T4742_S00002.62 | unknown protein |
| OPB38019 | T4742_S00002.66 | unknown protein |
| OPB38021 | T4742_S00002.68 | unknown protein |
| OPB38022 | T4742_S00002.69 | unknown protein |
| OPB38032 | T4742_S00002.79 | unknown protein |
| OPB38037 | T4742_S00002.84 | unknown protein |
| OPB38056 | T4742_S00002.103 | unknown protein |
| OPB38057 | T4742_S00002.104 | unknown protein |
| OPB38062 | T4742_S00002.109 | unknown protein |
| OPB38065 | T4742_S00002.112 | unknown protein |
| OPB38067 | T4742_S00002.114 | unknown protein |
| OPB38068 | T4742_S00002.115 | unknown protein |
| OPB38069 | T4742_S00002.116 | unknown protein |
| OPB38070 | T4742_S00002.117 | unknown protein |
| OPB38071 | T4742_S00002.118 | unknown protein |
| OPB38076 | T4742_S00002.123 | unknown protein |
| OPB38078 | T4742_S00002.125 | unknown protein |
| OPB38081 | T4742_S00002.128 | unknown protein |
| OPB38084 | T4742_S00002.131 | unknown protein |
| OPB38085 | T4742_S00002.132 | unknown protein |
| OPB38090 | T4742_S00002.137 | unknown protein |
| OPB38091 | T4742_S00002.138 | unknown protein |
| OPB38093 | T4742_S00002.140 | unknown protein |
| OPB38096 | T4742_S00002.143 | unknown protein |
| OPB38099 | T4742_S00002.146 | unknown protein |
| OPB38101 | T4742_S00002.148 | unknown protein |
| OPB38103 | T4742_S00002.150 | unknown protein |
| OPB38104 | T4742_S00002.151 | unknown protein |
| OPB38110 | T4742_S00002.157 | unknown protein |
| OPB38112 | T4742_S00002.159 | unknown protein |
| OPB38117 | T4742_S00002.164 | unknown protein |
| OPB38118 | T4742_S00002.165 | unknown protein |
| OPB38122 | T4742_S00002.169 | unknown protein |
| OPB38124 | T4742_S00002.171 | unknown protein |

| | | |
|---|---|---|
| OPB38128 | T4742_S00002.175 | unknown protein |
| OPB38130 | T4742_S00002.177 | unknown protein |
| OPB38134 | T4742_S00002.181 | unknown protein |
| OPB38137 | T4742_S00002.184 | unknown protein |
| OPB38141 | T4742_S00002.188 | unknown protein |
| OPB38143 | T4742_S00002.190 | unknown protein |
| OPB38145 | T4742_S00002.192 | unknown protein |
| OPB38146 | T4742_S00002.193 | unknown protein |
| OPB38147 | T4742_S00002.194 | unknown protein |
| OPB38150 | T4742_S00002.197 | unknown protein |
| OPB38152 | T4742_S00002.199 | unknown protein |
| OPB38159 | T4742_S00002.206 | unknown protein |
| OPB38164 | T4742_S00002.211 | unknown protein |
| OPB38166 | T4742_S00002.213 | unknown protein |
| OPB38167 | T4742_S00002.214 | unknown protein |
| OPB38168 | T4742_S00002.215 | unknown protein |
| OPB38174 | T4742_S00002.221 | unknown protein |
| OPB38175 | T4742_S00002.222 | unknown protein |
| OPB38176 | T4742_S00002.223 | unknown protein |
| OPB38178 | T4742_S00002.225 | unknown protein |
| OPB38181 | T4742_S00002.228 | unknown protein |
| OPB38183 | T4742_S00002.230 | unknown protein |
| OPB38184 | T4742_S00002.231 | unknown protein |
| OPB38187 | T4742_S00002.234 | unknown protein |
| OPB38188 | T4742_S00002.235 | unknown protein |
| OPB38189 | T4742_S00002.236 | unknown protein |
| OPB38191 | T4742_S00002.238 | unknown protein |
| OPB38193 | T4742_S00002.240 | unknown protein |
| OPB38194 | T4742_S00002.241 | unknown protein |
| OPB38196 | T4742_S00002.243 | unknown protein |
| OPB38199 | T4742_S00002.246 | unknown protein |
| OPB38200 | T4742_S00002.247 | unknown protein |
| OPB38201 | T4742_S00002.248 | unknown protein |
| OPB38203 | T4742_S00002.250 | unknown protein |
| OPB38208 | T4742_S00002.255 | unknown protein |
| OPB38221 | T4742_S00002.268 | unknown protein |

| | | |
|---|---|---|
| OPB38222 | T4742_S00002.269 | unknown protein |
| OPB38225 | T4742_S00002.272 | unknown protein |
| OPB38226 | T4742_S00002.273 | unknown protein |
| OPB38229 | T4742_S00002.276 | unknown protein |
| OPB38231 | T4742_S00002.278 | unknown protein |
| OPB38235 | T4742_S00002.282 | unknown protein |
| OPB38239 | T4742_S00002.286 | unknown protein |
| OPB38247 | T4742_S00002.294 | unknown protein |
| OPB38251 | T4742_S00002.298 | unknown protein |
| OPB38253 | T4742_S00002.300 | unknown protein |
| OPB38259 | T4742_S00002.306 | unknown protein |
| OPB38262 | T4742_S00002.309 | unknown protein |
| OPB38263 | T4742_S00002.310 | unknown protein |
| OPB38264 | T4742_S00002.311 | unknown protein |
| OPB38265 | T4742_S00002.312 | unknown protein |
| OPB38267 | T4742_S00002.314 | unknown protein |
| OPB38268 | T4742_S00002.315 | unknown protein |
| OPB38278 | T4742_S00002.325 | unknown protein |
| OPB38279 | T4742_S00002.326 | unknown protein |
| OPB38280 | T4742_S00002.327 | unknown protein |
| OPB38281 | T4742_S00002.328 | unknown protein |
| OPB38284 | T4742_S00002.331 | unknown protein |
| OPB38290 | T4742_S00002.337 | unknown protein |
| OPB38292 | T4742_S00002.339 | unknown protein |
| OPB38296 | T4742_S00002.343 | unknown protein |
| OPB38299 | T4742_S00002.346 | unknown protein |
| OPB38304 | T4742_S00002.351 | unknown protein |
| OPB38305 | T4742_S00002.352 | unknown protein |
| OPB38312 | T4742_S00002.359 | unknown protein |
| OPB38316 | T4742_S00002.363 | unknown protein |
| OPB38317 | T4742_S00002.365 | unknown protein |
| OPB38318 | T4742_S00002.366 | unknown protein |
| OPB38323 | T4742_S00002.371 | unknown protein |
| OPB38324 | T4742_S00002.372 | unknown protein |
| OPB38326 | T4742_S00002.374 | unknown protein |
| OPB38332 | T4742_S00002.380 | unknown protein |

| | | |
|---|---|---|
| OPB38333 | T4742_S00002.381 | unknown protein |
| OPB38337 | T4742_S00002.385 | Unknown protein |
| OPB38339 | T4742_S00002.387 | unknown protein |
| OPB38341 | T4742_S00002.389 | unknown protein |
| OPB38342 | T4742_S00002.390 | unknown protein |
| OPB38343 | T4742_S00002.391 | unknown protein |
| OPB38344 | T4742_S00002.392 | unknown protein |
| OPB38349 | T4742_S00002.397 | unknown protein |
| OPB38354 | T4742_S00002.402 | unknown protein |
| OPB38356 | T4742_S00002.404 | unknown protein |
| OPB38370 | T4742_S00002.418 | unknown protein |
| OPB38379 | T4742_S00002.427 | unknown protein |
| OPB38380 | T4742_S00002.428 | unknown protein |
| OPB38382 | T4742_S00002.430 | unknown protein |
| OPB38388 | T4742_S00002.436 | unknown protein |
| OPB38391 | T4742_S00002.439 | unknown protein |
| OPB38395 | T4742_S00002.443 | unknown protein |
| OPB38398 | T4742_S00002.446 | unknown protein |
| OPB38401 | T4742_S00002.449 | unknown protein |
| OPB38402 | T4742_S00002.450 | unknown protein |
| OPB38403 | T4742_S00002.451 | unknown protein |
| OPB38404 | T4742_S00002.452 | unknown protein |
| OPB38406 | T4742_S00002.454 | unknown protein |
| OPB38410 | T4742_S00002.458 | unknown protein |
| OPB38419 | T4742_S00002.467 | unknown protein |
| OPB38422 | T4742_S00002.470 | unknown protein |
| OPB38424 | T4742_S00002.472 | unknown protein |
| OPB38426 | T4742_S00002.474 | unknown protein |
| OPB38442 | T4742_S00002.490 | unknown protein |
| OPB38445 | T4742_S00002.493 | unknown protein |
| OPB38446 | T4742_S00002.494 | unknown protein |
| OPB38447 | T4742_S00002.495 | unknown protein |
| OPB38456 | T4742_S00002.504 | unknown protein |
| OPB38457 | T4742_S00002.505 | unknown protein |
| OPB38458 | T4742_S00002.506 | unknown protein |
| OPB38460 | T4742_S00002.508 | unknown protein |

| | | |
|---|---|---|
| OPB38463 | T4742_S00002.511 | unknown protein |
| OPB38464 | T4742_S00002.512 | unknown protein |
| OPB38466 | T4742_S00002.514 | unknown protein |
| OPB38469 | T4742_S00002.517 | unknown protein |
| OPB38471 | T4742_S00002.519 | unknown protein |
| OPB38476 | T4742_S00002.524 | unknown protein |
| OPB38479 | T4742_S00002.527 | unknown protein |
| OPB38481 | T4742_S00002.529 | unknown protein |
| OPB38483 | T4742_S00002.531 | unknown protein |
| OPB38484 | T4742_S00002.532 | unknown protein |
| OPB38490 | T4742_S00002.539 | unknown protein |
| OPB38494 | T4742_S00002.543 | unknown protein |
| OPB38495 | T4742_S00002.544 | unknown protein |
| OPB38504 | T4742_S00002.553 | unknown protein |
| OPB38506 | T4742_S00002.555 | unknown protein |
| OPB38508 | T4742_S00002.557 | unknown protein |
| OPB38512 | T4742_S00002.561 | unknown protein |
| OPB38514 | T4742_S00002.563 | unknown protein |
| OPB38516 | T4742_S00002.565 | unknown protein |
| OPB38517 | T4742_S00002.566 | unknown protein |
| OPB38519 | T4742_S00002.568 | unknown protein |
| OPB38526 | T4742_S00002.575 | unknown protein |
| OPB38527 | T4742_S00002.576 | unknown protein |
| OPB38535 | T4742_S00002.584 | unknown protein |
| OPB38537 | T4742_S00002.586 | unknown protein |
| OPB38547 | T4742_S00002.596 | unknown protein |
| OPB38554 | T4742_S00002.603 | unknown protein |
| OPB38558 | T4742_S00002.607 | unknown protein |
| OPB38560 | T4742_S00002.609 | unknown protein |
| OPB38562 | T4742_S00002.611 | unknown protein |
| OPB38571 | T4742_S00002.620 | unknown protein |
| OPB38574 | T4742_S00002.623 | unknown protein |
| OPB38576 | T4742_S00002.625 | unknown protein |
| OPB38577 | T4742_S00002.626 | unknown protein |
| OPB38581 | T4742_S00002.630 | unknown protein |
| OPB38582 | T4742_S00002.631 | unknown protein |

| | | |
|---|---|---|
| OPB38585 | T4742_S00002.634 | unknown protein |
| OPB38586 | T4742_S00002.635 | unknown protein |
| OPB38588 | T4742_S00002.637 | unknown protein |
| OPB38589 | T4742_S00002.638 | unknown protein |
| OPB38590 | T4742_S00002.639 | unknown protein |
| OPB38591 | T4742_S00002.640 | unknown protein |
| OPB38594 | T4742_S00002.643 | unknown protein |
| OPB38598 | T4742_S00002.647 | unknown protein |
| OPB38599 | T4742_S00002.648 | unknown protein |
| OPB38603 | T4742_S00002.652 | unknown protein |
| OPB38604 | T4742_S00002.653 | unknown protein |
| OPB38605 | T4742_S00002.654 | unknown protein |
| OPB38617 | T4742_S00002.666 | unknown protein |
| OPB38622 | T4742_S00002.671 | unknown protein |
| OPB38623 | T4742_S00002.672 | unknown protein |
| OPB38626 | T4742_S00002.675 | unknown protein |
| OPB38633 | T4742_S00002.682 | unknown protein |
| OPB38634 | T4742_S00002.683 | unknown protein |
| OPB38635 | T4742_S00002.684 | unknown protein |
| OPB38641 | T4742_S00002.690 | unknown protein |
| OPB38644 | T4742_S00002.693 | unknown protein |
| OPB38646 | T4742_S00002.695 | unknown protein |
| OPB38650 | T4742_S00002.699 | unknown protein |
| OPB38656 | T4742_S00002.705 | unknown protein |
| OPB38660 | T4742_S00002.709 | unknown protein |
| OPB38661 | T4742_S00002.710 | unknown protein |
| OPB38671 | T4742_S00002.720 | unknown protein |
| OPB38672 | T4742_S00002.721 | unknown protein |
| OPB38673 | T4742_S00002.722 | unknown protein |
| OPB38674 | T4742_S00002.723 | unknown protein |
| OPB38678 | T4742_S00002.727 | unknown protein |
| OPB38679 | T4742_S00002.728 | unknown protein |
| OPB38683 | T4742_S00002.732 | unknown protein |
| OPB38687 | T4742_S00002.736 | unknown protein |
| OPB38691 | T4742_S00002.740 | unknown protein |
| OPB38693 | T4742_S00002.742 | unknown protein |

| | | |
|---|---|---|
| OPB38694 | T4742_S00002.743 | unknown protein |
| OPB38695 | T4742_S00002.744 | unknown protein |
| OPB38698 | T4742_S00002.747 | unknown protein |
| OPB38699 | T4742_S00002.748 | unknown protein |
| OPB38700 | T4742_S00002.749 | unknown protein |
| OPB38704 | T4742_S00002.753 | unknown protein |
| OPB38707 | T4742_S00002.756 | unknown protein |
| OPB38708 | T4742_S00002.757 | unknown protein |
| OPB38709 | T4742_S00002.758 | unknown protein |
| OPB38710 | T4742_S00002.759 | unknown protein |
| OPB38711 | T4742_S00002.760 | unknown protein |
| OPB38713 | T4742_S00002.762 | unknown protein |
| OPB38714 | T4742_S00002.763 | unknown protein |
| OPB38716 | T4742_S00002.765 | unknown protein |
| OPB38717 | T4742_S00002.766 | unknown protein |
| OPB38719 | T4742_S00002.768 | unknown protein |
| OPB38734 | T4742_S00002.783 | unknown protein |
| OPB38737 | T4742_S00002.786 | unknown protein |
| OPB38739 | T4742_S00002.788 | unknown protein |
| OPB38740 | T4742_S00002.789 | unknown protein |
| OPB38750 | T4742_S00002.799 | unknown protein |
| OPB38754 | T4742_S00002.803 | unknown protein |
| OPB38756 | T4742_S00002.805 | unknown protein |
| OPB38758 | T4742_S00002.807 | unknown protein |
| OPB38765 | T4742_S00002.814 | unknown protein |
| OPB38771 | T4742_S00002.820 | unknown protein |
| OPB38778 | T4742_S00002.827 | unknown protein |
| OPB38779 | T4742_S00002.828 | unknown protein |
| OPB38781 | T4742_S00002.830 | unknown protein |
| OPB38786 | T4742_S00002.835 | unknown protein |
| OPB38789 | T4742_S00002.838 | unknown protein |
| OPB38795 | T4742_S00002.844 | unknown protein |
| OPB38797 | T4742_S00002.846 | unknown protein |
| OPB38800 | T4742_S00002.849 | unknown protein |
| OPB38801 | T4742_S00002.850 | unknown protein |
| OPB38802 | T4742_S00002.851 | unknown protein |

| | | |
|---|---|---|
| OPB38803 | T4742_S00002.852 | unknown protein |
| OPB38809 | T4742_S00002.858 | unknown protein |
| OPB38811 | T4742_S00002.860 | unknown protein |
| OPB38813 | T4742_S00002.862 | unknown protein |
| OPB38815 | T4742_S00002.864 | unknown protein |
| OPB38823 | T4742_S00002.872 | unknown protein |
| OPB38827 | T4742_S00002.876 | unknown protein |
| OPB38836 | T4742_S00002.885 | unknown protein |
| OPB38839 | T4742_S00002.888 | unknown protein |
| OPB38844 | T4742_S00002.893 | unknown protein |
| OPB38852 | T4742_S00002.901 | unknown protein |
| OPB38854 | T4742_S00002.903 | unknown protein |
| OPB38871 | T4742_S00002.920 | unknown protein |
| OPB38879 | T4742_S00002.928 | Unknown protein |
| OPB38882 | T4742_S00002.931 | unknown protein |
| OPB38886 | T4742_S00002.935 | unknown protein |
| OPB38889 | T4742_S00002.938 | unknown protein |
| OPB38893 | T4742_S00002.942 | unknown protein |
| OPB38896 | T4742_S00002.945 | unknown protein |
| OPB38898 | T4742_S00002.947 | unknown protein |
| OPB38905 | T4742_S00002.954 | unknown protein |
| OPB38920 | T4742_S00002.969 | unknown protein |
| OPB38923 | T4742_S00002.972 | unknown protein |
| OPB38924 | T4742_S00002.973 | unknown protein |
| OPB38931 | T4742_S00002.980 | unknown protein |
| OPB38936 | T4742_S00002.985 | unknown protein |
| OPB38947 | T4742_S00006.6 | unknown protein |
| OPB38949 | T4742_S00006.8 | unknown protein |
| OPB38950 | T4742_S00006.9 | unknown protein |
| OPB38951 | T4742_S00006.10 | unknown protein |
| OPB38953 | T4742_S00006.12 | unknown protein |
| OPB38957 | T4742_S00006.16 | unknown protein |
| OPB38959 | T4742_S00006.18 | unknown protein |
| OPB38960 | T4742_S00006.19 | unknown protein |
| OPB38961 | T4742_S00006.20 | unknown protein |
| OPB38967 | T4742_S00006.26 | unknown protein |

| | | |
|---|---|---|
| OPB38970 | T4742_S00006.29 | unknown protein |
| OPB38977 | T4742_S00006.36 | unknown protein |
| OPB38988 | T4742_S00006.47 | Unknown protein |
| OPB38990 | T4742_S00006.49 | unknown protein |
| OPB38991 | T4742_S00006.50 | unknown protein |
| OPB38992 | T4742_S00006.51 | unknown protein |
| OPB38994 | T4742_S00006.53 | unknown protein |
| OPB39002 | T4742_S00006.61 | unknown protein |
| OPB39007 | T4742_S00006.66 | unknown protein |
| OPB39011 | T4742_S00006.70 | unknown protein |
| OPB39013 | T4742_S00006.72 | unknown protein |
| OPB39017 | T4742_S00006.76 | unknown protein |
| OPB39018 | T4742_S00006.77 | unknown protein |
| OPB39019 | T4742_S00006.78 | unknown protein |
| OPB39020 | T4742_S00006.79 | unknown protein |
| OPB39023 | T4742_S00006.82 | unknown protein |
| OPB39025 | T4742_S00006.84 | unknown protein |
| OPB39031 | T4742_S00006.90 | unknown protein |
| OPB39032 | T4742_S00006.91 | unknown protein |
| OPB39035 | T4742_S00006.94 | unknown protein |
| OPB39046 | T4742_S00006.105 | unknown protein |
| OPB39047 | T4742_S00006.106 | unknown protein |
| OPB39048 | T4742_S00006.107 | unknown protein |
| OPB39049 | T4742_S00006.108 | unknown protein |
| OPB39051 | T4742_S00006.110 | unknown protein |
| OPB39054 | T4742_S00006.113 | unknown protein |
| OPB39057 | T4742_S00006.116 | unknown protein |
| OPB39062 | T4742_S00006.121 | unknown protein |
| OPB39063 | T4742_S00006.122 | unknown protein |
| OPB39065 | T4742_S00006.124 | unknown protein |
| OPB39073 | T4742_S00006.132 | unknown protein |
| OPB39074 | T4742_S00006.133 | unknown protein |
| OPB39075 | T4742_S00006.134 | unknown protein |
| OPB39076 | T4742_S00006.135 | unknown protein |
| OPB39083 | T4742_S00006.142 | unknown protein |
| OPB39095 | T4742_S00006.154 | unknown protein |

| | | |
|---|---|---|
| OPB39096 | T4742_S00006.155 | unknown protein |
| OPB39097 | T4742_S00006.156 | unknown protein |
| OPB39106 | T4742_S00006.166 | unknown protein |
| OPB39109 | T4742_S00006.169 | unknown protein |
| OPB39111 | T4742_S00006.171 | unknown protein |
| OPB39112 | T4742_S00006.172 | unknown protein |
| OPB39115 | T4742_S00006.175 | unknown protein |
| OPB39120 | T4742_S00006.180 | unknown protein |
| OPB39130 | T4742_S00006.190 | unknown protein |
| OPB39134 | T4742_S00006.194 | unknown protein |
| OPB39135 | T4742_S00006.195 | unknown protein |
| OPB39136 | T4742_S00006.196 | unknown protein |
| OPB39140 | T4742_S00006.200 | unknown protein |
| OPB39141 | T4742_S00006.201 | unknown protein |
| OPB39146 | T4742_S00006.206 | unknown protein |
| OPB39152 | T4742_S00006.212 | unknown protein |
| OPB39157 | T4742_S00006.217 | unknown protein |
| OPB39160 | T4742_S00006.220 | unknown protein |
| OPB39163 | T4742_S00006.223 | unknown protein |
| OPB39169 | T4742_S00006.229 | unknown protein |
| OPB39173 | T4742_S00006.233 | unknown protein |
| OPB39176 | T4742_S00006.236 | unknown protein |
| OPB39181 | T4742_S00006.241 | unknown protein |
| OPB39182 | T4742_S00006.242 | unknown protein |
| OPB39191 | T4742_S00006.251 | unknown protein |
| OPB39193 | T4742_S00006.253 | unknown protein |
| OPB39196 | T4742_S00006.256 | unknown protein |
| OPB39198 | T4742_S00006.258 | unknown protein |
| OPB39200 | T4742_S00006.260 | unknown protein |
| OPB39203 | T4742_S00006.263 | unknown protein |
| OPB39204 | T4742_S00006.264 | unknown protein |
| OPB39205 | T4742_S00006.265 | unknown protein |
| OPB39206 | T4742_S00006.266 | unknown protein |
| OPB39208 | T4742_S00006.268 | unknown protein |
| OPB39212 | T4742_S00006.272 | unknown protein |
| OPB39214 | T4742_S00006.274 | unknown protein |

| | | |
|---|---|---|
| OPB39218 | T4742_S00006.278 | unknown protein |
| OPB39219 | T4742_S00006.279 | unknown protein |
| OPB39225 | T4742_S00006.285 | unknown protein |
| OPB39226 | T4742_S00006.286 | unknown protein |
| OPB39229 | T4742_S00006.289 | unknown protein |
| OPB39230 | T4742_S00006.290 | unknown protein |
| OPB39235 | T4742_S00006.295 | unknown protein |
| OPB39238 | T4742_S00006.298 | unknown protein |
| OPB39241 | T4742_S00006.301 | unknown protein |
| OPB39243 | T4742_S00006.303 | unknown protein |
| OPB39245 | T4742_S00006.305 | unknown protein |
| OPB39246 | T4742_S00006.306 | unknown protein |
| OPB39247 | T4742_S00006.307 | unknown protein |
| OPB39248 | T4742_S00006.308 | unknown protein |
| OPB39249 | T4742_S00006.309 | unknown protein |
| OPB39255 | T4742_S00006.315 | unknown protein |
| OPB39256 | T4742_S00006.316 | unknown protein |
| OPB39260 | T4742_S00006.320 | unknown protein |
| OPB39261 | T4742_S00006.321 | unknown protein |
| OPB39265 | T4742_S00006.325 | unknown protein |
| OPB39268 | T4742_S00006.328 | unknown protein |
| OPB39271 | T4742_S00006.331 | unknown protein |
| OPB39273 | T4742_S00006.333 | unknown protein |
| OPB39275 | T4742_S00006.335 | unknown protein |
| OPB39276 | T4742_S00006.336 | unknown protein |
| OPB39277 | T4742_S00006.337 | unknown protein |
| OPB39278 | T4742_S00006.338 | unknown protein |
| OPB39284 | T4742_S00006.344 | unknown protein |
| OPB39288 | T4742_S00006.348 | unknown protein |
| OPB39290 | T4742_S00006.350 | unknown protein |
| OPB39291 | T4742_S00006.351 | unknown protein |
| OPB39293 | T4742_S00006.353 | unknown protein |
| OPB39298 | T4742_S00006.358 | unknown protein |
| OPB39304 | T4742_S00006.364 | unknown protein |
| OPB39308 | T4742_S00006.368 | unknown protein |
| OPB39316 | T4742_S00006.376 | unknown protein |

| | | |
|---|---|---|
| OPB39319 | T4742_S00006.379 | unknown protein |
| OPB39325 | T4742_S00006.385 | unknown protein |
| OPB39336 | T4742_S00006.396 | unknown protein |
| OPB39339 | T4742_S00006.399 | unknown protein |
| OPB39341 | T4742_S00006.401 | unknown protein |
| OPB39344 | T4742_S00006.404 | unknown protein |
| OPB39345 | T4742_S00006.405 | unknown protein |
| OPB39346 | T4742_S00006.406 | unknown protein |
| OPB39347 | T4742_S00006.407 | unknown protein |
| OPB39348 | T4742_S00006.408 | unknown protein |
| OPB39354 | T4742_S00006.414 | unknown protein |
| OPB39358 | T4742_S00006.418 | unknown protein |
| OPB39367 | T4742_S00006.427 | unknown protein |
| OPB39372 | T4742_S00006.432 | unknown protein |
| OPB39374 | T4742_S00006.434 | unknown protein |
| OPB39379 | T4742_S00006.439 | unknown protein |
| OPB39388 | T4742_S00006.448 | unknown protein |
| OPB39389 | T4742_S00006.449 | unknown protein |
| OPB39390 | T4742_S00006.450 | unknown protein |
| OPB39392 | T4742_S00006.452 | unknown protein |
| OPB39394 | T4742_S00006.454 | unknown protein |
| OPB39403 | T4742_S00006.463 | unknown protein |
| OPB39404 | T4742_S00006.464 | unknown protein |
| OPB39405 | T4742_S00006.465 | unknown protein |
| OPB39406 | T4742_S00006.466 | unknown protein |
| OPB39408 | T4742_S00006.468 | unknown protein |
| OPB39409 | T4742_S00006.469 | unknown protein |
| OPB39413 | T4742_S00006.473 | unknown protein |
| OPB39414 | T4742_S00006.474 | unknown protein |
| OPB39415 | T4742_S00006.475 | unknown protein |
| OPB39416 | T4742_S00006.476 | unknown protein |
| OPB39417 | T4742_S00006.477 | unknown protein |
| OPB39418 | T4742_S00006.478 | unknown protein |
| OPB39419 | T4742_S00006.479 | unknown protein |
| OPB39423 | T4742_S00006.483 | unknown protein |
| OPB39424 | T4742_S00006.484 | unknown protein |

| | | |
|---|---|---|
| OPB39426 | T4742_S00006.486 | unknown protein |
| OPB39428 | T4742_S00006.488 | unknown protein |
| OPB39434 | T4742_S00006.494 | unknown protein |
| OPB39435 | T4742_S00006.495 | unknown protein |
| OPB39441 | T4742_S00006.501 | unknown protein |
| OPB39444 | T4742_S00006.504 | unknown protein |
| OPB39448 | T4742_S00006.508 | unknown protein |
| OPB39449 | T4742_S00006.509 | unknown protein |
| OPB39450 | T4742_S00006.510 | unknown protein |
| OPB39451 | T4742_S00006.511 | unknown protein |
| OPB39452 | T4742_S00006.512 | unknown protein |
| OPB39453 | T4742_S00006.513 | unknown protein |
| OPB39456 | T4742_S00006.516 | unknown protein |
| OPB39458 | T4742_S00006.518 | unknown protein |
| OPB39460 | T4742_S00006.520 | unknown protein |
| OPB39462 | T4742_S00006.522 | unknown protein |
| OPB39463 | T4742_S00006.523 | unknown protein |
| OPB39476 | T4742_S00006.536 | unknown protein |
| OPB39479 | T4742_S00006.539 | unknown protein |
| OPB39481 | T4742_S00006.541 | unknown protein |
| OPB39482 | T4742_S00006.542 | unknown protein |
| OPB39483 | T4742_S00006.543 | unknown protein |
| OPB39485 | T4742_S00006.545 | unknown protein |
| OPB39488 | T4742_S00006.548 | unknown protein |
| OPB39489 | T4742_S00006.549 | unknown protein |
| OPB39491 | T4742_S00006.551 | unknown protein |
| OPB39496 | T4742_S00006.556 | unknown protein |
| OPB39500 | T4742_S00006.560 | unknown protein |
| OPB39503 | T4742_S00006.563 | unknown protein |
| OPB39504 | T4742_S00006.564 | unknown protein |
| OPB39509 | T4742_S00006.569 | unknown protein |
| OPB39510 | T4742_S00006.570 | unknown protein |
| OPB39516 | T4742_S00006.576 | unknown protein |
| OPB39523 | T4742_S00006.583 | unknown protein |
| OPB39526 | T4742_S00006.586 | unknown protein |
| OPB39527 | T4742_S00006.587 | unknown protein |

| | | |
|---|---|---|
| OPB39528 | T4742_S00006.588 | unknown protein |
| OPB39530 | T4742_S00006.590 | unknown protein |
| OPB39532 | T4742_S00006.592 | unknown protein |
| OPB39533 | T4742_S00006.593 | unknown protein |
| OPB39537 | T4742_S00006.597 | unknown protein |
| OPB39540 | T4742_S00006.600 | unknown protein |
| OPB39543 | T4742_S00006.603 | unknown protein |
| OPB39546 | T4742_S00006.606 | unknown protein |
| OPB39550 | T4742_S00006.610 | unknown protein |
| OPB39551 | T4742_S00006.611 | unknown protein |
| OPB39559 | T4742_S00006.619 | unknown protein |
| OPB39565 | T4742_S00006.625 | unknown protein |
| OPB39569 | T4742_S00006.629 | unknown protein |
| OPB39570 | T4742_S00006.630 | unknown protein |
| OPB39575 | T4742_S00006.635 | unknown protein |
| OPB39576 | T4742_S00006.636 | unknown protein |
| OPB39579 | T4742_S00006.639 | unknown protein |
| OPB39585 | T4742_S00006.645 | unknown protein |
| OPB39588 | T4742_S00006.648 | unknown protein |
| OPB39589 | T4742_S00006.649 | unknown protein |
| OPB39590 | T4742_S00006.650 | unknown protein |
| OPB39591 | T4742_S00006.651 | unknown protein |
| OPB39595 | T4742_S00006.655 | Unknown protein |
| OPB39596 | T4742_S00006.656 | unknown protein |
| OPB39599 | T4742_S00006.659 | unknown protein |
| OPB39601 | T4742_S00006.661 | unknown protein |
| OPB39619 | T4742_S00006.679 | unknown protein |
| OPB39628 | T4742_S00006.688 | unknown protein |
| OPB39632 | T4742_S00006.692 | unknown protein |
| OPB39633 | T4742_S00006.693 | unknown protein |
| OPB39635 | T4742_S00006.695 | unknown protein |
| OPB39636 | T4742_S00006.696 | unknown protein |
| OPB39642 | T4742_S00006.702 | unknown protein |
| OPB39643 | T4742_S00006.703 | unknown protein |
| OPB39650 | T4742_S00006.710 | unknown protein |
| OPB39654 | T4742_S00006.714 | unknown protein |

| | | |
|---|---|---|
| OPB39656 | T4742_S00006.716 | unknown protein |
| OPB39657 | T4742_S00015.1 | unknown protein |
| OPB39658 | T4742_S00015.2 | unknown protein |
| OPB39664 | T4742_S00015.8 | unknown protein |
| OPB39665 | T4742_S00015.9 | unknown protein |
| OPB39666 | T4742_S00015.10 | unknown protein |
| OPB39668 | T4742_S00015.12 | unknown protein |
| OPB39680 | T4742_S00015.24 | unknown protein |
| OPB39687 | T4742_S00015.31 | unknown protein |
| OPB39691 | T4742_S00015.35 | unknown protein |
| OPB39696 | T4742_S00015.40 | unknown protein |
| OPB39700 | T4742_S00015.44 | unknown protein |
| OPB39702 | T4742_S00015.46 | unknown protein |
| OPB39705 | T4742_S00015.49 | unknown protein |
| OPB39708 | T4742_S00015.52 | unknown protein |
| OPB39709 | T4742_S00015.53 | unknown protein |
| OPB39710 | T4742_S00015.54 | unknown protein |
| OPB39712 | T4742_S00015.56 | unknown protein |
| OPB39713 | T4742_S00015.57 | unknown protein |
| OPB39714 | T4742_S00015.58 | unknown protein |
| OPB39715 | T4742_S00015.59 | unknown protein |
| OPB39717 | T4742_S00015.61 | unknown protein |
| OPB39718 | T4742_S00015.62 | unknown protein |
| OPB39720 | T4742_S00015.64 | unknown protein |
| OPB39726 | T4742_S00015.70 | unknown protein |
| OPB39727 | T4742_S00015.71 | unknown protein |
| OPB39730 | T4742_S00015.74 | unknown protein |
| OPB39731 | T4742_S00015.75 | unknown protein |
| OPB39732 | T4742_S00015.76 | unknown protein |
| OPB39735 | T4742_S00015.79 | unknown protein |
| OPB39737 | T4742_S00015.81 | unknown protein |
| OPB39738 | T4742_S00015.82 | unknown protein |
| OPB39741 | T4742_S00015.85 | unknown protein |
| OPB39742 | T4742_S00015.86 | unknown protein |
| OPB39745 | T4742_S00015.89 | unknown protein |
| OPB39747 | T4742_S00015.91 | unknown protein |

| | | |
|---|---|---|
| OPB39748 | T4742_S00015.92 | unknown protein |
| OPB39749 | T4742_S00015.93 | unknown protein |
| OPB39750 | T4742_S00015.94 | unknown protein |
| OPB39751 | T4742_S00015.95 | unknown protein |
| OPB39754 | T4742_S00015.98 | unknown protein |
| OPB39762 | T4742_S00015.106 | unknown protein |
| OPB39765 | T4742_S00015.109 | unknown protein |
| OPB39768 | T4742_S00015.112 | unknown protein |
| OPB39769 | T4742_S00015.113 | unknown protein |
| OPB39773 | T4742_S00015.117 | unknown protein |
| OPB39777 | T4742_S00015.121 | unknown protein |
| OPB39779 | T4742_S00015.123 | unknown protein |
| OPB39781 | T4742_S00015.125 | unknown protein |
| OPB39785 | T4742_S00015.129 | unknown protein |
| OPB39786 | T4742_S00015.130 | unknown protein |
| OPB39788 | T4742_S00015.132 | unknown protein |
| OPB39791 | T4742_S00015.135 | unknown protein |
| OPB39792 | T4742_S00015.136 | unknown protein |
| OPB39797 | T4742_S00015.141 | unknown protein |
| OPB39799 | T4742_S00015.143 | unknown protein |
| OPB39800 | T4742_S00015.144 | unknown protein |
| OPB39803 | T4742_S00015.147 | unknown protein |
| OPB39809 | T4742_S00015.153 | unknown protein |
| OPB39811 | T4742_S00015.155 | unknown protein |
| OPB39815 | T4742_S00015.159 | unknown protein |
| OPB39817 | T4742_S00015.161 | unknown protein |
| OPB39819 | T4742_S00015.163 | unknown protein |
| OPB39822 | T4742_S00015.166 | unknown protein |
| OPB39823 | T4742_S00015.167 | unknown protein |
| OPB39824 | T4742_S00015.168 | unknown protein |
| OPB39826 | T4742_S00015.170 | unknown protein |
| OPB39827 | T4742_S00015.171 | unknown protein |
| OPB39829 | T4742_S00015.173 | unknown protein |
| OPB39833 | T4742_S00015.177 | unknown protein |
| OPB39835 | T4742_S00015.179 | unknown protein |
| OPB39846 | T4742_S00015.190 | unknown protein |

| | | |
|---|---|---|
| OPB39848 | T4742_S00015.192 | unknown protein |
| OPB39850 | T4742_S00015.194 | unknown protein |
| OPB39852 | T4742_S00015.196 | unknown protein |
| OPB39859 | T4742_S00015.203 | unknown protein |
| OPB39861 | T4742_S00015.205 | unknown protein |
| OPB39862 | T4742_S00015.206 | unknown protein |
| OPB39865 | T4742_S00015.209 | unknown protein |
| OPB39868 | T4742_S00015.212 | unknown protein |
| OPB39869 | T4742_S00015.213 | unknown protein |
| OPB39872 | T4742_S00015.216 | unknown protein |
| OPB39874 | T4742_S00015.218 | unknown protein |
| OPB39881 | T4742_S00015.225 | unknown protein |
| OPB39884 | T4742_S00015.228 | unknown protein |
| OPB39890 | T4742_S00015.234 | unknown protein |
| OPB39892 | T4742_S00015.236 | unknown protein |
| OPB39900 | T4742_S00015.244 | unknown protein |
| OPB39902 | T4742_S00015.246 | unknown protein |
| OPB39903 | T4742_S00015.247 | unknown protein |
| OPB39909 | T4742_S00015.253 | unknown protein |
| OPB39912 | T4742_S00015.256 | unknown protein |
| OPB39913 | T4742_S00015.257 | unknown protein |
| OPB39915 | T4742_S00015.259 | unknown protein |
| OPB39918 | T4742_S00015.262 | unknown protein |
| OPB39921 | T4742_S00015.265 | unknown protein |
| OPB39925 | T4742_S00001.4 | unknown protein |
| OPB39926 | T4742_S00001.5 | unknown protein |
| OPB39934 | T4742_S00001.13 | unknown protein |
| OPB39937 | T4742_S00001.16 | unknown protein |
| OPB39939 | T4742_S00001.18 | unknown protein |
| OPB39941 | T4742_S00001.20 | unknown protein |
| OPB39946 | T4742_S00001.25 | unknown protein |
| OPB39947 | T4742_S00001.26 | unknown protein |
| OPB39948 | T4742_S00001.27 | unknown protein |
| OPB39949 | T4742_S00001.28 | unknown protein |
| OPB39950 | T4742_S00001.29 | unknown protein |
| OPB39952 | T4742_S00001.31 | unknown protein |

| | | |
|---|---|---|
| OPB39953 | T4742_S00001.32 | unknown protein |
| OPB39954 | T4742_S00001.33 | unknown protein |
| OPB39955 | T4742_S00001.34 | unknown protein |
| OPB39956 | T4742_S00001.35 | unknown protein |
| OPB39967 | T4742_S00001.46 | unknown protein |
| OPB39968 | T4742_S00001.47 | unknown protein |
| OPB39969 | T4742_S00001.48 | unknown protein |
| OPB39976 | T4742_S00001.55 | unknown protein |
| OPB39977 | T4742_S00001.56 | unknown protein |
| OPB39980 | T4742_S00001.59 | unknown protein |
| OPB39981 | T4742_S00001.60 | unknown protein |
| OPB39982 | T4742_S00001.61 | unknown protein |
| OPB39984 | T4742_S00001.63 | unknown protein |
| OPB39990 | T4742_S00001.69 | unknown protein |
| OPB39992 | T4742_S00001.71 | unknown protein |
| OPB39993 | T4742_S00001.72 | Unknown protein |
| OPB39994 | T4742_S00001.73 | unknown protein |
| OPB39998 | T4742_S00001.77 | unknown protein |
| OPB40000 | T4742_S00001.79 | unknown protein |
| OPB40003 | T4742_S00001.82 | unknown protein |
| OPB40010 | T4742_S00001.89 | unknown protein |
| OPB40011 | T4742_S00001.90 | unknown protein |
| OPB40015 | T4742_S00001.94 | unknown protein |
| OPB40016 | T4742_S00001.95 | unknown protein |
| OPB40019 | T4742_S00001.98 | unknown protein |
| OPB40025 | T4742_S00001.104 | unknown protein |
| OPB40026 | T4742_S00001.105 | unknown protein |
| OPB40030 | T4742_S00001.109 | unknown protein |
| OPB40037 | T4742_S00001.116 | unknown protein |
| OPB40041 | T4742_S00001.120 | unknown protein |
| OPB40043 | T4742_S00001.122 | unknown protein |
| OPB40050 | T4742_S00001.129 | unknown protein |
| OPB40052 | T4742_S00001.131 | unknown protein |
| OPB40053 | T4742_S00001.132 | unknown protein |
| OPB40054 | T4742_S00001.133 | unknown protein |
| OPB40056 | T4742_S00001.135 | unknown protein |

| | | |
|---|---|---|
| OPB40057 | T4742_S00001.136 | unknown protein |
| OPB40058 | T4742_S00001.137 | unknown protein |
| OPB40061 | T4742_S00001.140 | unknown protein |
| OPB40065 | T4742_S00001.144 | unknown protein |
| OPB40066 | T4742_S00001.145 | unknown protein |
| OPB40069 | T4742_S00001.148 | unknown protein |
| OPB40072 | T4742_S00001.151 | unknown protein |
| OPB40076 | T4742_S00001.155 | unknown protein |
| OPB40077 | T4742_S00001.156 | unknown protein |
| OPB40081 | T4742_S00001.160 | unknown protein |
| OPB40082 | T4742_S00001.161 | unknown protein |
| OPB40090 | T4742_S00001.169 | unknown protein |
| OPB40091 | T4742_S00001.170 | unknown protein |
| OPB40094 | T4742_S00001.173 | unknown protein |
| OPB40096 | T4742_S00001.175 | unknown protein |
| OPB40103 | T4742_S00001.182 | unknown protein |
| OPB40104 | T4742_S00001.183 | unknown protein |
| OPB40108 | T4742_S00001.187 | unknown protein |
| OPB40112 | T4742_S00001.191 | unknown protein |
| OPB40117 | T4742_S00001.196 | unknown protein |
| OPB40124 | T4742_S00001.203 | Unknown protein |
| OPB40125 | T4742_S00001.204 | unknown protein |
| OPB40127 | T4742_S00001.206 | unknown protein |
| OPB40130 | T4742_S00001.209 | unknown protein |
| OPB40132 | T4742_S00001.211 | unknown protein |
| OPB40133 | T4742_S00001.212 | unknown protein |
| OPB40142 | T4742_S00001.221 | unknown protein |
| OPB40146 | T4742_S00001.225 | unknown protein |
| OPB40150 | T4742_S00001.229 | unknown protein |
| OPB40153 | T4742_S00001.232 | unknown protein |
| OPB40154 | T4742_S00001.233 | unknown protein |
| OPB40156 | T4742_S00001.235 | unknown protein |
| OPB40157 | T4742_S00001.236 | unknown protein |
| OPB40159 | T4742_S00001.238 | unknown protein |
| OPB40161 | T4742_S00001.240 | unknown protein |
| OPB40163 | T4742_S00001.242 | unknown protein |

| | | |
|---|---|---|
| OPB40168 | T4742_S00001.247 | unknown protein |
| OPB40172 | T4742_S00001.251 | unknown protein |
| OPB40173 | T4742_S00001.252 | unknown protein |
| OPB40181 | T4742_S00001.260 | unknown protein |
| OPB40183 | T4742_S00001.262 | unknown protein |
| OPB40184 | T4742_S00001.263 | unknown protein |
| OPB40186 | T4742_S00001.265 | unknown protein |
| OPB40187 | T4742_S00001.266 | unknown protein |
| OPB40191 | T4742_S00001.270 | unknown protein |
| OPB40193 | T4742_S00001.272 | unknown protein |
| OPB40195 | T4742_S00001.274 | unknown protein |
| OPB40198 | T4742_S00001.277 | unknown protein |
| OPB40200 | T4742_S00001.279 | unknown protein |
| OPB40203 | T4742_S00001.282 | unknown protein |
| OPB40204 | T4742_S00001.283 | unknown protein |
| OPB40205 | T4742_S00001.284 | unknown protein |
| OPB40207 | T4742_S00001.286 | unknown protein |
| OPB40209 | T4742_S00001.288 | unknown protein |
| OPB40210 | T4742_S00001.289 | unknown protein |
| OPB40211 | T4742_S00001.290 | unknown protein |
| OPB40225 | T4742_S00001.304 | unknown protein |
| OPB40229 | T4742_S00001.308 | unknown protein |
| OPB40232 | T4742_S00001.311 | unknown protein |
| OPB40234 | T4742_S00001.313 | unknown protein |
| OPB40236 | T4742_S00001.315 | unknown protein |
| OPB40237 | T4742_S00001.316 | unknown protein |
| OPB40240 | T4742_S00001.319 | unknown protein |
| OPB40241 | T4742_S00001.320 | unknown protein |
| OPB40245 | T4742_S00001.324 | unknown protein |
| OPB40246 | T4742_S00001.325 | unknown protein |
| OPB40248 | T4742_S00001.327 | unknown protein |
| OPB40251 | T4742_S00001.330 | unknown protein |
| OPB40253 | T4742_S00001.332 | unknown protein |
| OPB40255 | T4742_S00001.334 | unknown protein |
| OPB40256 | T4742_S00001.335 | unknown protein |
| OPB40259 | T4742_S00001.338 | unknown protein |

| | | |
|---|---|---|
| OPB40261 | T4742_S00001.340 | unknown protein |
| OPB40265 | T4742_S00001.344 | unknown protein |
| OPB40266 | T4742_S00001.345 | unknown protein |
| OPB40268 | T4742_S00001.347 | unknown protein |
| OPB40269 | T4742_S00001.348 | unknown protein |
| OPB40274 | T4742_S00001.353 | unknown protein |
| OPB40275 | T4742_S00001.354 | unknown protein |
| OPB40277 | T4742_S00001.356 | unknown protein |
| OPB40279 | T4742_S00001.358 | unknown protein |
| OPB40280 | T4742_S00001.359 | unknown protein |
| OPB40283 | T4742_S00001.362 | unknown protein |
| OPB40284 | T4742_S00001.363 | unknown protein |
| OPB40295 | T4742_S00001.374 | unknown protein |
| OPB40300 | T4742_S00001.379 | unknown protein |
| OPB40304 | T4742_S00001.383 | unknown protein |
| OPB40306 | T4742_S00001.385 | unknown protein |
| OPB40307 | T4742_S00001.386 | unknown protein |
| OPB40317 | T4742_S00001.396 | unknown protein |
| OPB40318 | T4742_S00001.397 | unknown protein |
| OPB40327 | T4742_S00001.406 | unknown protein |
| OPB40347 | T4742_S00001.426 | unknown protein |
| OPB40356 | T4742_S00001.435 | unknown protein |
| OPB40357 | T4742_S00001.436 | unknown protein |
| OPB40359 | T4742_S00001.438 | unknown protein |
| OPB40360 | T4742_S00001.439 | Unknown protein |
| OPB40361 | T4742_S00001.440 | unknown protein |
| OPB40364 | T4742_S00001.443 | unknown protein |
| OPB40368 | T4742_S00001.447 | unknown protein |
| OPB40369 | T4742_S00001.448 | unknown protein |
| OPB40373 | T4742_S00001.452 | unknown protein |
| OPB40374 | T4742_S00001.453 | unknown protein |
| OPB40376 | T4742_S00001.455 | unknown protein |
| OPB40379 | T4742_S00001.458 | unknown protein |
| OPB40380 | T4742_S00001.459 | unknown protein |
| OPB40405 | T4742_S00001.484 | unknown protein |
| OPB40407 | T4742_S00001.486 | unknown protein |

| | | |
|---|---|---|
| OPB40408 | T4742_S00001.487 | unknown protein |
| OPB40415 | T4742_S00001.494 | unknown protein |
| OPB40421 | T4742_S00001.500 | unknown protein |
| OPB40422 | T4742_S00001.501 | unknown protein |
| OPB40423 | T4742_S00001.502 | unknown protein |
| OPB40425 | T4742_S00001.504 | unknown protein |
| OPB40430 | T4742_S00001.509 | unknown protein |
| OPB40431 | T4742_S00001.510 | unknown protein |
| OPB40434 | T4742_S00001.513 | unknown protein |
| OPB40439 | T4742_S00001.518 | unknown protein |
| OPB40441 | T4742_S00001.520 | unknown protein |
| OPB40442 | T4742_S00001.521 | unknown protein |
| OPB40443 | T4742_S00001.522 | unknown protein |
| OPB40444 | T4742_S00001.523 | unknown protein |
| OPB40448 | T4742_S00001.527 | unknown protein |
| OPB40457 | T4742_S00001.536 | unknown protein |
| OPB40468 | T4742_S00001.547 | unknown protein |
| OPB40471 | T4742_S00001.550 | unknown protein |
| OPB40474 | T4742_S00001.553 | unknown protein |
| OPB40477 | T4742_S00001.556 | unknown protein |
| OPB40480 | T4742_S00001.559 | unknown protein |
| OPB40486 | T4742_S00001.565 | unknown protein |
| OPB40487 | T4742_S00001.566 | unknown protein |
| OPB40488 | T4742_S00001.567 | unknown protein |
| OPB40491 | T4742_S00001.570 | unknown protein |
| OPB40495 | T4742_S00001.574 | unknown protein |
| OPB40496 | T4742_S00001.575 | unknown protein |
| OPB40497 | T4742_S00001.576 | unknown protein |
| OPB40499 | T4742_S00001.578 | unknown protein |
| OPB40500 | T4742_S00001.579 | unknown protein |
| OPB40502 | T4742_S00001.581 | unknown protein |
| OPB40507 | T4742_S00001.586 | unknown protein |
| OPB40509 | T4742_S00001.588 | unknown protein |
| OPB40522 | T4742_S00001.601 | unknown protein |
| OPB40523 | T4742_S00001.602 | unknown protein |
| OPB40524 | T4742_S00001.603 | unknown protein |

| | | |
|---|---|---|
| OPB40525 | T4742_S00001.604 | unknown protein |
| OPB40527 | T4742_S00001.606 | unknown protein |
| OPB40529 | T4742_S00001.608 | unknown protein |
| OPB40531 | T4742_S00001.610 | unknown protein |
| OPB40533 | T4742_S00001.612 | unknown protein |
| OPB40535 | T4742_S00001.614 | unknown protein |
| OPB40539 | T4742_S00001.618 | unknown protein |
| OPB40542 | T4742_S00001.621 | unknown protein |
| OPB40544 | T4742_S00001.623 | unknown protein |
| OPB40559 | T4742_S00001.638 | unknown protein |
| OPB40560 | T4742_S00001.639 | unknown protein |
| OPB40566 | T4742_S00001.645 | unknown protein |
| OPB40581 | T4742_S00001.661 | unknown protein |
| OPB40584 | T4742_S00001.664 | unknown protein |
| OPB40589 | T4742_S00001.669 | unknown protein |
| OPB40593 | T4742_S00001.673 | unknown protein |
| OPB40594 | T4742_S00001.674 | unknown protein |
| OPB40596 | T4742_S00001.676 | unknown protein |
| OPB40598 | T4742_S00001.678 | unknown protein |
| OPB40599 | T4742_S00001.679 | unknown protein |
| OPB40600 | T4742_S00001.680 | unknown protein |
| OPB40601 | T4742_S00001.681 | unknown protein |
| OPB40604 | T4742_S00001.684 | unknown protein |
| OPB40608 | T4742_S00001.688 | unknown protein |
| OPB40609 | T4742_S00001.689 | unknown protein |
| OPB40612 | T4742_S00001.692 | unknown protein |
| OPB40617 | T4742_S00001.697 | unknown protein |
| OPB40618 | T4742_S00001.698 | unknown protein |
| OPB40619 | T4742_S00001.699 | unknown protein |
| OPB40620 | T4742_S00001.700 | unknown protein |
| OPB40621 | T4742_S00001.701 | unknown protein |
| OPB40623 | T4742_S00001.703 | unknown protein |
| OPB40624 | T4742_S00001.704 | unknown protein |
| OPB40625 | T4742_S00001.705 | unknown protein |
| OPB40627 | T4742_S00001.707 | unknown protein |
| OPB40648 | T4742_S00001.728 | unknown protein |

| | | |
|---|---|---|
| OPB40650 | T4742_S00001.730 | unknown protein |
| OPB40659 | T4742_S00001.739 | unknown protein |
| OPB40685 | T4742_S00001.765 | unknown protein |
| OPB40689 | T4742_S00001.769 | unknown protein |
| OPB40703 | T4742_S00001.783 | unknown protein |
| OPB40704 | T4742_S00001.784 | unknown protein |
| OPB40706 | T4742_S00001.786 | unknown protein |
| OPB40711 | T4742_S00001.791 | unknown protein |
| OPB40731 | T4742_S00001.811 | unknown protein |
| OPB40734 | T4742_S00001.814 | unknown protein |
| OPB40735 | T4742_S00001.815 | unknown protein |
| OPB40754 | T4742_S00001.835 | unknown protein |
| OPB40764 | T4742_S00001.845 | unknown protein |
| OPB40766 | T4742_S00001.847 | unknown protein |
| OPB40787 | T4742_S00001.868 | unknown protein |
| OPB40792 | T4742_S00001.873 | unknown protein |
| OPB40793 | T4742_S00001.874 | unknown protein |
| OPB40795 | T4742_S00001.876 | unknown protein |
| OPB40804 | T4742_S00001.885 | unknown protein |
| OPB40805 | T4742_S00001.886 | unknown protein |
| OPB40816 | T4742_S00001.897 | unknown protein |
| OPB40819 | T4742_S00001.900 | unknown protein |
| OPB40822 | T4742_S00001.903 | unknown protein |
| OPB40838 | T4742_S00001.919 | unknown protein |
| OPB40839 | T4742_S00001.920 | unknown protein |
| OPB40844 | T4742_S00001.925 | unknown protein |
| OPB40851 | T4742_S00001.932 | unknown protein |
| OPB40854 | T4742_S00001.935 | unknown protein |
| OPB40858 | T4742_S00001.939 | unknown protein |
| OPB40859 | T4742_S00001.940 | unknown protein |
| OPB40860 | T4742_S00001.941 | unknown protein |
| OPB40865 | T4742_S00001.946 | unknown protein |
| OPB40867 | T4742_S00001.948 | unknown protein |
| OPB40874 | T4742_S00001.955 | unknown protein |
| OPB40884 | T4742_S00001.965 | unknown protein |
| OPB40887 | T4742_S00001.968 | unknown protein |

| | | |
|---|---|---|
| OPB40896 | T4742_S00001.977 | unknown protein |
| OPB40897 | T4742_S00001.978 | unknown protein |
| OPB40899 | T4742_S00001.980 | unknown protein |
| OPB40900 | T4742_S00001.981 | unknown protein |
| OPB40907 | T4742_S00001.988 | unknown protein |
| OPB40912 | T4742_S00001.993 | unknown protein |
| OPB40924 | T4742_S00001.1005 | unknown protein |
| OPB40928 | T4742_S00001.1009 | unknown protein |
| OPB40934 | T4742_S00001.1015 | unknown protein |
| OPB40935 | T4742_S00001.1016 | unknown protein |
| OPB40942 | T4742_S00001.1023 | unknown protein |
| OPB40945 | T4742_S00001.1026 | unknown protein |
| OPB40952 | T4742_S00001.1033 | unknown protein |
| OPB40954 | T4742_S00001.1035 | unknown protein |
| OPB40955 | T4742_S00001.1036 | unknown protein |
| OPB40958 | T4742_S00001.1039 | unknown protein |
| OPB40960 | T4742_S00001.1041 | unknown protein |
| OPB40965 | T4742_S00001.1046 | unknown protein |
| OPB40971 | T4742_S00001.1052 | unknown protein |
| OPB40977 | T4742_S00020.1 | unknown protein |
| OPB40978 | T4742_S00020.2 | unknown protein |
| OPB40983 | T4742_S00020.7 | unknown protein |
| OPB40990 | T4742_S00020.14 | unknown protein |
| OPB40992 | T4742_S00020.16 | unknown protein |
| OPB40994 | T4742_S00020.18 | unknown protein |
| OPB40996 | T4742_S00020.20 | unknown protein |
| OPB40998 | T4742_S00020.22 | unknown protein |
| OPB40999 | T4742_S00020.23 | unknown protein |
| OPB41005 | T4742_S00020.29 | unknown protein |
| OPB41014 | T4742_S00020.38 | unknown protein |
| OPB41015 | T4742_S00020.39 | unknown protein |
| OPB41028 | T4742_S00020.52 | unknown protein |
| OPB41031 | T4742_S00020.55 | unknown protein |
| OPB41033 | T4742_S00020.57 | unknown protein |
| OPB41043 | T4742_S00020.67 | unknown protein |
| OPB41053 | T4742_S00020.77 | unknown protein |

| | | |
|---|---|---|
| OPB41054 | T4742_S00020.78 | unknown protein |
| OPB41056 | T4742_S00020.80 | unknown protein |
| OPB41059 | T4742_S00020.83 | unknown protein |
| OPB41062 | T4742_S00020.86 | unknown protein |
| OPB41064 | T4742_S00020.88 | unknown protein |
| OPB41067 | T4742_S00020.91 | unknown protein |
| OPB41069 | T4742_S00020.93 | unknown protein |
| OPB41070 | T4742_S00020.94 | unknown protein |
| OPB41072 | T4742_S00020.96 | unknown protein |
| OPB41073 | T4742_S00020.97 | unknown protein |
| OPB41077 | T4742_S00020.101 | unknown protein |
| OPB41080 | T4742_S00020.104 | unknown protein |
| OPB41084 | T4742_S00020.108 | unknown protein |
| OPB41089 | T4742_S00020.113 | unknown protein |
| OPB41094 | T4742_S00020.118 | unknown protein |
| OPB41099 | T4742_S00020.123 | unknown protein |
| OPB41107 | T4742_S00020.131 | unknown protein |
| OPB41115 | T4742_S00020.139 | unknown protein |
| OPB41128 | T4742_S00020.152 | unknown protein |
| OPB41129 | T4742_S00020.153 | unknown protein |
| OPB41131 | T4742_S00020.155 | unknown protein |
| OPB41137 | T4742_S00020.161 | unknown protein |
| OPB41138 | T4742_S00020.162 | unknown protein |
| OPB41139 | T4742_S00020.163 | unknown protein |
| OPB41140 | T4742_S00020.164 | unknown protein |
| OPB41141 | T4742_S00020.165 | unknown protein |
| OPB41142 | T4742_S00020.166 | unknown protein |
| OPB41143 | T4742_S00020.167 | unknown protein |
| OPB41149 | T4742_S00020.173 | unknown protein |
| OPB41150 | T4742_S00020.174 | unknown protein |
| OPB41151 | T4742_S00020.175 | unknown protein |
| OPB41154 | T4742_S00020.178 | unknown protein |
| OPB41165 | T4742_S00011.5 | unknown protein |
| OPB41174 | T4742_S00011.14 | unknown protein |
| OPB41178 | T4742_S00011.18 | unknown protein |
| OPB41199 | T4742_S00011.39 | unknown protein |

| | | |
|---|---|---|
| OPB41215 | T4742_S00011.55 | unknown protein |
| OPB41217 | T4742_S00011.57 | unknown protein |
| OPB41218 | T4742_S00011.58 | unknown protein |
| OPB41224 | T4742_S00011.65 | unknown protein |
| OPB41234 | T4742_S00011.75 | unknown protein |
| OPB41239 | T4742_S00011.80 | unknown protein |
| OPB41240 | T4742_S00011.81 | unknown protein |
| OPB41242 | T4742_S00011.83 | unknown protein |
| OPB41244 | T4742_S00011.85 | unknown protein |
| OPB41245 | T4742_S00011.86 | unknown protein |
| OPB41246 | T4742_S00011.87 | unknown protein |
| OPB41266 | T4742_S00011.107 | unknown protein |
| OPB41273 | T4742_S00011.114 | unknown protein |
| OPB41274 | T4742_S00011.115 | unknown protein |
| OPB41283 | T4742_S00011.124 | unknown protein |
| OPB41291 | T4742_S00011.132 | unknown protein |
| OPB41297 | T4742_S00011.138 | unknown protein |
| OPB41299 | T4742_S00011.140 | unknown protein |
| OPB41308 | T4742_S00011.149 | unknown protein |
| OPB41312 | T4742_S00011.153 | unknown protein |
| OPB41316 | T4742_S00011.157 | unknown protein |
| OPB41318 | T4742_S00011.159 | unknown protein |
| OPB41329 | T4742_S00011.170 | unknown protein |
| OPB41334 | T4742_S00011.175 | unknown protein |
| OPB41337 | T4742_S00011.178 | unknown protein |
| OPB41338 | T4742_S00011.179 | unknown protein |
| OPB41339 | T4742_S00011.180 | unknown protein |
| OPB41340 | T4742_S00011.181 | unknown protein |
| OPB41343 | T4742_S00011.184 | unknown protein |
| OPB41345 | T4742_S00011.186 | unknown protein |
| OPB41363 | T4742_S00011.204 | unknown protein |
| OPB41365 | T4742_S00011.206 | unknown protein |
| OPB41377 | T4742_S00011.218 | unknown protein |
| OPB41381 | T4742_S00011.222 | unknown protein |
| OPB41382 | T4742_S00011.223 | unknown protein |
| OPB41384 | T4742_S00011.225 | unknown protein |

| | | |
|---|---|---|
| OPB41388 | T4742_S00011.229 | unknown protein |
| OPB41392 | T4742_S00011.234 | unknown protein |
| OPB41398 | T4742_S00011.240 | unknown protein |
| OPB41399 | T4742_S00011.241 | unknown protein |
| OPB41406 | T4742_S00011.248 | unknown protein |
| OPB41407 | T4742_S00011.249 | unknown protein |
| OPB41409 | T4742_S00011.251 | unknown protein |
| OPB41415 | T4742_S00011.257 | unknown protein |
| OPB41417 | T4742_S00011.260 | unknown protein |
| OPB41422 | T4742_S00011.265 | Unknown protein |
| OPB41424 | T4742_S00011.267 | unknown protein |
| OPB41425 | T4742_S00011.268 | unknown protein |
| OPB41428 | T4742_S00011.271 | unknown protein |
| OPB41429 | T4742_S00011.272 | unknown protein |
| OPB41434 | T4742_S00011.277 | unknown protein |
| OPB41435 | T4742_S00011.278 | unknown protein |
| OPB41438 | T4742_S00011.281 | unknown protein |
| OPB41440 | T4742_S00011.283 | unknown protein |
| OPB41442 | T4742_S00011.285 | unknown protein |
| OPB41443 | T4742_S00011.286 | unknown protein |
| OPB41454 | T4742_S00011.297 | unknown protein |
| OPB41455 | T4742_S00011.298 | unknown protein |
| OPB41457 | T4742_S00011.300 | unknown protein |
| OPB41460 | T4742_S00011.303 | unknown protein |
| OPB41462 | T4742_S00011.305 | unknown protein |
| OPB41484 | T4742_S00011.327 | unknown protein |
| OPB41488 | T4742_S00011.331 | unknown protein |
| OPB41490 | T4742_S00011.333 | unknown protein |
| OPB41492 | T4742_S00011.335 | unknown protein |
| OPB41493 | T4742_S00011.336 | unknown protein |
| OPB41497 | T4742_S00011.340 | unknown protein |
| OPB41498 | T4742_S00011.341 | unknown protein |
| OPB41506 | T4742_S00011.349 | unknown protein |
| OPB41508 | T4742_S00011.351 | unknown protein |
| OPB41509 | T4742_S00011.352 | unknown protein |
| OPB41510 | T4742_S00011.353 | unknown protein |

| | | |
|---|---|---|
| OPB41511 | T4742_S00011.354 | unknown protein |
| OPB41514 | T4742_S00011.357 | unknown protein |
| OPB41515 | T4742_S00011.358 | unknown protein |
| OPB41518 | T4742_S00011.361 | unknown protein |
| OPB41519 | T4742_S00011.362 | unknown protein |
| OPB41522 | T4742_S00011.365 | unknown protein |
| OPB41523 | T4742_S00011.366 | unknown protein |
| OPB41531 | T4742_S00011.374 | unknown protein |
| OPB41534 | T4742_S00011.377 | unknown protein |
| OPB41548 | T4742_S00011.391 | unknown protein |
| OPB41549 | T4742_S00011.392 | unknown protein |
| OPB41551 | T4742_S00011.394 | unknown protein |
| OPB41552 | T4742_S00011.395 | unknown protein |
| OPB41559 | T4742_S00011.402 | unknown protein |
| OPB41560 | T4742_S00011.403 | unknown protein |
| OPB41564 | T4742_S00011.407 | unknown protein |
| OPB41566 | T4742_S00011.409 | unknown protein |
| OPB41567 | T4742_S00011.410 | unknown protein |
| OPB41569 | T4742_S00011.412 | unknown protein |
| OPB41574 | T4742_S00011.417 | unknown protein |
| OPB41575 | T4742_S00011.418 | unknown protein |
| OPB41577 | T4742_S00011.420 | unknown protein |
| OPB41580 | T4742_S00011.423 | unknown protein |
| OPB41581 | T4742_S00011.424 | unknown protein |
| OPB41582 | T4742_S00011.425 | unknown protein |
| OPB41584 | T4742_S00011.427 | unknown protein |
| OPB41586 | T4742_S00011.429 | unknown protein |
| OPB41591 | T4742_S00011.434 | unknown protein |
| OPB41597 | T4742_S00011.440 | unknown protein |
| OPB41599 | T4742_S00011.442 | unknown protein |
| OPB41600 | T4742_S00011.443 | unknown protein |
| OPB41601 | T4742_S00011.444 | unknown protein |
| OPB41602 | T4742_S00011.445 | unknown protein |
| OPB41605 | T4742_S00011.448 | unknown protein |
| OPB41610 | T4742_S00011.453 | unknown protein |
| OPB41618 | T4742_S00011.461 | unknown protein |

| | | |
|---|---|---|
| OPB41619 | T4742_S00011.462 | unknown protein |
| OPB41620 | T4742_S00011.463 | unknown protein |
| OPB41623 | T4742_S00011.466 | unknown protein |
| OPB41629 | T4742_S00011.472 | unknown protein |
| OPB41631 | T4742_S00011.474 | unknown protein |
| OPB41633 | T4742_S00011.476 | unknown protein |
| OPB41634 | T4742_S00011.477 | unknown protein |
| OPB41639 | T4742_S00011.482 | unknown protein |
| OPB41640 | T4742_S00011.483 | unknown protein |
| OPB41645 | T4742_S00011.488 | unknown protein |
| OPB41646 | T4742_S00011.489 | unknown protein |
| OPB41648 | T4742_S00011.491 | unknown protein |
| OPB41650 | T4742_S00011.493 | unknown protein |
| OPB41651 | T4742_S00011.494 | unknown protein |
| OPB41652 | T4742_S00011.495 | unknown protein |
| OPB41653 | T4742_S00011.496 | unknown protein |
| OPB41654 | T4742_S00011.497 | unknown protein |
| OPB41659 | T4742_S00011.502 | unknown protein |
| OPB41660 | T4742_S00011.503 | unknown protein |
| OPB41665 | T4742_S00011.508 | unknown protein |
| OPB41666 | T4742_S00011.509 | unknown protein |
| OPB41667 | T4742_S00011.510 | unknown protein |
| OPB41676 | T4742_S00011.519 | unknown protein |
| OPB41684 | T4742_S00018.6 | unknown protein |
| OPB41685 | T4742_S00018.7 | unknown protein |
| OPB41687 | T4742_S00018.9 | unknown protein |
| OPB41689 | T4742_S00018.11 | unknown protein |
| OPB41690 | T4742_S00018.12 | unknown protein |
| OPB41694 | T4742_S00018.16 | unknown protein |
| OPB41720 | T4742_S00018.43 | unknown protein |
| OPB41722 | T4742_S00018.45 | unknown protein |
| OPB41725 | T4742_S00018.48 | unknown protein |
| OPB41729 | T4742_S00018.52 | unknown protein |
| OPB41730 | T4742_S00018.53 | unknown protein |
| OPB41733 | T4742_S00018.56 | unknown protein |
| OPB41735 | T4742_S00018.58 | unknown protein |

| | | |
|---|---|---|
| OPB41742 | T4742_S00018.65 | unknown protein |
| OPB41744 | T4742_S00018.67 | unknown protein |
| OPB41750 | T4742_S00018.73 | unknown protein |
| OPB41751 | T4742_S00018.74 | unknown protein |
| OPB41756 | T4742_S00018.79 | unknown protein |
| OPB41757 | T4742_S00018.80 | unknown protein |
| OPB41760 | T4742_S00018.83 | unknown protein |
| OPB41761 | T4742_S00018.84 | unknown protein |
| OPB41762 | T4742_S00018.85 | unknown protein |
| OPB41767 | T4742_S00018.90 | unknown protein |
| OPB41771 | T4742_S00018.94 | unknown protein |
| OPB41773 | T4742_S00018.96 | unknown protein |
| OPB41776 | T4742_S00018.99 | unknown protein |
| OPB41781 | T4742_S00018.104 | unknown protein |
| OPB41800 | T4742_S00018.123 | unknown protein |
| OPB41801 | T4742_S00018.124 | unknown protein |
| OPB41802 | T4742_S00018.125 | unknown protein |
| OPB41814 | T4742_S00018.137 | unknown protein |
| OPB41815 | T4742_S00018.138 | unknown protein |
| OPB41825 | T4742_S00018.148 | unknown protein |
| OPB41827 | T4742_S00018.150 | unknown protein |
| OPB41828 | T4742_S00018.151 | unknown protein |
| OPB41831 | T4742_S00018.154 | unknown protein |
| OPB41832 | T4742_S00018.155 | unknown protein |
| OPB41849 | T4742_S00018.172 | unknown protein |
| OPB41854 | T4742_S00018.177 | unknown protein |
| OPB41857 | T4742_S00018.180 | unknown protein |
| OPB41859 | T4742_S00018.182 | unknown protein |
| OPB41863 | T4742_S00018.186 | unknown protein |
| OPB41871 | T4742_S00018.194 | unknown protein |
| OPB41873 | T4742_S00018.196 | unknown protein |
| OPB41876 | T4742_S00018.199 | unknown protein |
| OPB41891 | T4742_S00018.214 | unknown protein |
| OPB41893 | T4742_S00018.216 | unknown protein |
| OPB41899 | T4742_S00004.1 | Unknown protein |
| OPB41901 | T4742_S00004.3 | unknown protein |

| | | |
|---|---|---|
| OPB41916 | T4742_S00004.18 | unknown protein |
| OPB41917 | T4742_S00004.19 | unknown protein |
| OPB41922 | T4742_S00004.24 | unknown protein |
| OPB41923 | T4742_S00004.25 | unknown protein |
| OPB41924 | T4742_S00004.26 | unknown protein |
| OPB41927 | T4742_S00004.29 | unknown protein |
| OPB41929 | T4742_S00004.31 | unknown protein |
| OPB41932 | T4742_S00004.34 | unknown protein |
| OPB41935 | T4742_S00004.37 | unknown protein |
| OPB41942 | T4742_S00004.44 | unknown protein |
| OPB41947 | T4742_S00004.49 | unknown protein |
| OPB41954 | T4742_S00004.56 | unknown protein |
| OPB41965 | T4742_S00004.67 | unknown protein |
| OPB41968 | T4742_S00004.70 | unknown protein |
| OPB41974 | T4742_S00004.76 | unknown protein |
| OPB41978 | T4742_S00004.80 | unknown protein |
| OPB41982 | T4742_S00004.84 | unknown protein |
| OPB41987 | T4742_S00004.89 | unknown protein |
| OPB41988 | T4742_S00004.90 | unknown protein |
| OPB41995 | T4742_S00004.97 | unknown protein |
| OPB41996 | T4742_S00004.98 | unknown protein |
| OPB42004 | T4742_S00004.106 | unknown protein |
| OPB42005 | T4742_S00004.107 | unknown protein |
| OPB42006 | T4742_S00004.108 | unknown protein |
| OPB42010 | T4742_S00004.112 | unknown protein |
| OPB42013 | T4742_S00004.115 | unknown protein |
| OPB42020 | T4742_S00004.122 | unknown protein |
| OPB42021 | T4742_S00004.123 | unknown protein |
| OPB42022 | T4742_S00004.124 | unknown protein |
| OPB42028 | T4742_S00004.130 | unknown protein |
| OPB42034 | T4742_S00004.136 | unknown protein |
| OPB42035 | T4742_S00004.137 | unknown protein |
| OPB42038 | T4742_S00004.140 | unknown protein |
| OPB42040 | T4742_S00004.142 | unknown protein |
| OPB42041 | T4742_S00004.143 | unknown protein |
| OPB42049 | T4742_S00004.151 | unknown protein |

| | | |
|---|---|---|
| OPB42053 | T4742_S00004.155 | unknown protein |
| OPB42054 | T4742_S00004.156 | unknown protein |
| OPB42056 | T4742_S00004.158 | unknown protein |
| OPB42060 | T4742_S00004.162 | unknown protein |
| OPB42063 | T4742_S00004.165 | unknown protein |
| OPB42064 | T4742_S00004.166 | unknown protein |
| OPB42065 | T4742_S00004.167 | unknown protein |
| OPB42066 | T4742_S00004.168 | unknown protein |
| OPB42068 | T4742_S00004.170 | unknown protein |
| OPB42071 | T4742_S00004.173 | unknown protein |
| OPB42072 | T4742_S00004.174 | unknown protein |
| OPB42074 | T4742_S00004.176 | unknown protein |
| OPB42076 | T4742_S00004.178 | unknown protein |
| OPB42078 | T4742_S00004.180 | unknown protein |
| OPB42081 | T4742_S00004.183 | unknown protein |
| OPB42083 | T4742_S00004.185 | unknown protein |
| OPB42086 | T4742_S00004.188 | unknown protein |
| OPB42088 | T4742_S00004.190 | unknown protein |
| OPB42090 | T4742_S00004.192 | unknown protein |
| OPB42095 | T4742_S00004.197 | unknown protein |
| OPB42096 | T4742_S00004.198 | unknown protein |
| OPB42097 | T4742_S00004.199 | unknown protein |
| OPB42098 | T4742_S00004.200 | unknown protein |
| OPB42101 | T4742_S00004.203 | unknown protein |
| OPB42104 | T4742_S00004.206 | unknown protein |
| OPB42105 | T4742_S00004.207 | unknown protein |
| OPB42107 | T4742_S00004.209 | unknown protein |
| OPB42108 | T4742_S00004.210 | unknown protein |
| OPB42109 | T4742_S00004.211 | unknown protein |
| OPB42113 | T4742_S00004.215 | Unknown protein |
| OPB42115 | T4742_S00004.217 | unknown protein |
| OPB42117 | T4742_S00004.219 | unknown protein |
| OPB42119 | T4742_S00004.221 | unknown protein |
| OPB42120 | T4742_S00004.222 | unknown protein |
| OPB42123 | T4742_S00004.225 | unknown protein |
| OPB42126 | T4742_S00004.228 | unknown protein |

| | | |
|---|---|---|
| OPB42129 | T4742_S00004.231 | unknown protein |
| OPB42131 | T4742_S00004.233 | unknown protein |
| OPB42134 | T4742_S00004.236 | unknown protein |
| OPB42135 | T4742_S00004.237 | unknown protein |
| OPB42136 | T4742_S00004.238 | unknown protein |
| OPB42145 | T4742_S00004.247 | unknown protein |
| OPB42148 | T4742_S00004.250 | unknown protein |
| OPB42156 | T4742_S00004.258 | unknown protein |
| OPB42167 | T4742_S00004.269 | unknown protein |
| OPB42170 | T4742_S00004.272 | unknown protein |
| OPB42177 | T4742_S00004.279 | unknown protein |
| OPB42178 | T4742_S00004.280 | unknown protein |
| OPB42179 | T4742_S00004.281 | unknown protein |
| OPB42180 | T4742_S00004.282 | unknown protein |
| OPB42181 | T4742_S00004.283 | unknown protein |
| OPB42182 | T4742_S00004.284 | unknown protein |
| OPB42190 | T4742_S00004.292 | unknown protein |
| OPB42196 | T4742_S00004.298 | unknown protein |
| OPB42197 | T4742_S00004.299 | unknown protein |
| OPB42198 | T4742_S00004.300 | unknown protein |
| OPB42200 | T4742_S00004.302 | unknown protein |
| OPB42202 | T4742_S00004.304 | unknown protein |
| OPB42203 | T4742_S00004.305 | unknown protein |
| OPB42204 | T4742_S00004.306 | unknown protein |
| OPB42208 | T4742_S00004.310 | unknown protein |
| OPB42209 | T4742_S00004.311 | unknown protein |
| OPB42215 | T4742_S00004.317 | unknown protein |
| OPB42219 | T4742_S00004.321 | Unknown protein |
| OPB42220 | T4742_S00004.322 | unknown protein |
| OPB42221 | T4742_S00004.323 | unknown protein |
| OPB42223 | T4742_S00004.325 | unknown protein |
| OPB42228 | T4742_S00004.330 | unknown protein |
| OPB42233 | T4742_S00004.335 | Unknown protein |
| OPB42236 | T4742_S00004.338 | unknown protein |
| OPB42241 | T4742_S00004.343 | unknown protein |
| OPB42245 | T4742_S00004.347 | unknown protein |

| | | |
|---|---|---|
| OPB42246 | T4742_S00004.348 | unknown protein |
| OPB42255 | T4742_S00004.357 | unknown protein |
| OPB42257 | T4742_S00004.359 | unknown protein |
| OPB42259 | T4742_S00004.361 | unknown protein |
| OPB42261 | T4742_S00004.363 | unknown protein |
| OPB42262 | T4742_S00004.364 | unknown protein |
| OPB42263 | T4742_S00004.365 | unknown protein |
| OPB42270 | T4742_S00004.372 | unknown protein |
| OPB42274 | T4742_S00004.376 | unknown protein |
| OPB42276 | T4742_S00004.378 | unknown protein |
| OPB42278 | T4742_S00004.380 | unknown protein |
| OPB42280 | T4742_S00004.382 | unknown protein |
| OPB42281 | T4742_S00004.383 | unknown protein |
| OPB42289 | T4742_S00004.391 | unknown protein |
| OPB42292 | T4742_S00004.394 | unknown protein |
| OPB42294 | T4742_S00004.396 | Unknown protein |
| OPB42298 | T4742_S00004.400 | unknown protein |
| OPB42299 | T4742_S00004.401 | unknown protein |
| OPB42301 | T4742_S00004.403 | unknown protein |
| OPB42304 | T4742_S00004.406 | unknown protein |
| OPB42305 | T4742_S00004.407 | unknown protein |
| OPB42306 | T4742_S00004.408 | unknown protein |
| OPB42308 | T4742_S00004.410 | unknown protein |
| OPB42313 | T4742_S00004.415 | unknown protein |
| OPB42314 | T4742_S00004.416 | unknown protein |
| OPB42315 | T4742_S00004.417 | unknown protein |
| OPB42317 | T4742_S00004.419 | unknown protein |
| OPB42319 | T4742_S00004.421 | unknown protein |
| OPB42320 | T4742_S00004.422 | unknown protein |
| OPB42321 | T4742_S00004.423 | unknown protein |
| OPB42324 | T4742_S00004.426 | unknown protein |
| OPB42325 | T4742_S00004.427 | unknown protein |
| OPB42327 | T4742_S00004.429 | unknown protein |
| OPB42329 | T4742_S00004.431 | unknown protein |
| OPB42333 | T4742_S00004.435 | unknown protein |
| OPB42346 | T4742_S00004.448 | unknown protein |

| | | |
|---|---|---|
| OPB42357 | T4742_S00004.459 | unknown protein |
| OPB42358 | T4742_S00004.460 | unknown protein |
| OPB42359 | T4742_S00004.461 | unknown protein |
| OPB42360 | T4742_S00004.462 | unknown protein |
| OPB42361 | T4742_S00004.463 | unknown protein |
| OPB42370 | T4742_S00004.472 | unknown protein |
| OPB42372 | T4742_S00004.474 | unknown protein |
| OPB42374 | T4742_S00004.476 | unknown protein |
| OPB42380 | T4742_S00004.482 | unknown protein |
| OPB42387 | T4742_S00004.489 | unknown protein |
| OPB42392 | T4742_S00004.494 | unknown protein |
| OPB42394 | T4742_S00004.496 | unknown protein |
| OPB42395 | T4742_S00004.497 | unknown protein |
| OPB42397 | T4742_S00004.499 | unknown protein |
| OPB42398 | T4742_S00004.500 | unknown protein |
| OPB42401 | T4742_S00004.503 | unknown protein |
| OPB42404 | T4742_S00004.506 | unknown protein |
| OPB42405 | T4742_S00004.507 | unknown protein |
| OPB42411 | T4742_S00004.513 | unknown protein |
| OPB42413 | T4742_S00004.515 | unknown protein |
| OPB42415 | T4742_S00004.517 | unknown protein |
| OPB42416 | T4742_S00004.518 | Unknown protein |
| OPB42420 | T4742_S00004.522 | unknown protein |
| OPB42421 | T4742_S00004.523 | unknown protein |
| OPB42425 | T4742_S00004.527 | unknown protein |
| OPB42426 | T4742_S00004.528 | unknown protein |
| OPB42427 | T4742_S00004.529 | unknown protein |
| OPB42428 | T4742_S00004.530 | unknown protein |
| OPB42429 | T4742_S00004.531 | unknown protein |
| OPB42430 | T4742_S00004.532 | unknown protein |
| OPB42431 | T4742_S00004.533 | unknown protein |
| OPB42432 | T4742_S00004.534 | unknown protein |
| OPB42433 | T4742_S00004.535 | unknown protein |
| OPB42434 | T4742_S00004.536 | unknown protein |
| OPB42437 | T4742_S00004.539 | unknown protein |
| OPB42440 | T4742_S00004.542 | unknown protein |

| | | |
|---|---|---|
| OPB42442 | T4742_S00004.544 | unknown protein |
| OPB42443 | T4742_S00004.545 | unknown protein |
| OPB42444 | T4742_S00004.546 | unknown protein |
| OPB42445 | T4742_S00004.547 | unknown protein |
| OPB42447 | T4742_S00004.549 | unknown protein |
| OPB42448 | T4742_S00004.550 | unknown protein |
| OPB42450 | T4742_S00004.552 | unknown protein |
| OPB42451 | T4742_S00004.553 | unknown protein |
| OPB42452 | T4742_S00004.554 | unknown protein |
| OPB42455 | T4742_S00004.557 | unknown protein |
| OPB42456 | T4742_S00004.558 | unknown protein |
| OPB42457 | T4742_S00004.559 | unknown protein |
| OPB42466 | T4742_S00004.568 | unknown protein |
| OPB42468 | T4742_S00004.570 | unknown protein |
| OPB42471 | T4742_S00004.573 | unknown protein |
| OPB42472 | T4742_S00004.574 | unknown protein |
| OPB42474 | T4742_S00004.576 | unknown protein |
| OPB42477 | T4742_S00004.579 | unknown protein |
| OPB42481 | T4742_S00004.583 | unknown protein |
| OPB42485 | T4742_S00004.587 | unknown protein |
| OPB42487 | T4742_S00004.589 | unknown protein |
| OPB42489 | T4742_S00004.591 | unknown protein |
| OPB42493 | T4742_S00004.595 | unknown protein |
| OPB42494 | T4742_S00004.596 | unknown protein |
| OPB42495 | T4742_S00004.597 | unknown protein |
| OPB42496 | T4742_S00004.598 | unknown protein |
| OPB42502 | T4742_S00004.604 | unknown protein |
| OPB42517 | T4742_S00004.619 | unknown protein |
| OPB42518 | T4742_S00004.620 | unknown protein |
| OPB42520 | T4742_S00004.622 | unknown protein |
| OPB42526 | T4742_S00004.628 | unknown protein |
| OPB42527 | T4742_S00004.629 | unknown protein |
| OPB42529 | T4742_S00004.631 | unknown protein |
| OPB42535 | T4742_S00004.637 | unknown protein |
| OPB42536 | T4742_S00004.638 | unknown protein |
| OPB42537 | T4742_S00004.640 | unknown protein |

| | | |
|---|---|---|
| OPB42539 | T4742_S00004.642 | unknown protein |
| OPB42543 | T4742_S00004.646 | unknown protein |
| OPB42544 | T4742_S00004.647 | unknown protein |
| OPB42545 | T4742_S00004.648 | unknown protein |
| OPB42546 | T4742_S00004.649 | unknown protein |
| OPB42548 | T4742_S00004.651 | unknown protein |
| OPB42549 | T4742_S00004.652 | unknown protein |
| OPB42556 | T4742_S00004.659 | unknown protein |
| OPB42559 | T4742_S00004.662 | unknown protein |
| OPB42563 | T4742_S00004.666 | unknown protein |
| OPB42578 | T4742_S00004.681 | unknown protein |
| OPB42587 | T4742_S00004.691 | unknown protein |
| OPB42589 | T4742_S00004.693 | unknown protein |
| OPB42591 | T4742_S00004.695 | unknown protein |
| OPB42593 | T4742_S00004.697 | unknown protein |
| OPB42594 | T4742_S00004.698 | unknown protein |
| OPB42596 | T4742_S00004.700 | unknown protein |
| OPB42597 | T4742_S00004.701 | unknown protein |
| OPB42598 | T4742_S00004.702 | unknown protein |
| OPB42599 | T4742_S00004.703 | unknown protein |
| OPB42601 | T4742_S00004.705 | unknown protein |
| OPB42602 | T4742_S00004.706 | unknown protein |
| OPB42607 | T4742_S00004.711 | unknown protein |
| OPB42612 | T4742_S00004.716 | unknown protein |
| OPB42614 | T4742_S00004.718 | unknown protein |
| OPB42625 | T4742_S00004.730 | unknown protein |
| OPB42627 | T4742_S00004.732 | unknown protein |
| OPB42629 | T4742_S00004.734 | unknown protein |
| OPB42630 | T4742_S00004.735 | unknown protein |
| OPB42634 | T4742_S00004.739 | unknown protein |
| OPB42636 | T4742_S00004.741 | unknown protein |
| OPB42640 | T4742_S00004.745 | unknown protein |
| OPB42641 | T4742_S00004.746 | unknown protein |
| OPB42643 | T4742_S00004.748 | unknown protein |
| OPB42644 | T4742_S00004.749 | unknown protein |
| OPB42649 | T4742_S00004.754 | unknown protein |

| | | |
|---|---|---|
| OPB42650 | T4742_S00004.755 | unknown protein |
| OPB42651 | T4742_S00004.756 | unknown protein |
| OPB42652 | T4742_S00004.757 | unknown protein |
| OPB42653 | T4742_S00004.758 | unknown protein |
| OPB42656 | T4742_S00004.761 | unknown protein |
| OPB42664 | T4742_S00004.769 | unknown protein |
| OPB42667 | T4742_S00004.772 | unknown protein |
| OPB42670 | T4742_S00004.775 | unknown protein |
| OPB42675 | T4742_S00004.780 | unknown protein |
| OPB42677 | T4742_S00004.782 | unknown protein |
| OPB42678 | T4742_S00004.783 | unknown protein |
| OPB42688 | T4742_S00004.794 | unknown protein |
| OPB42692 | T4742_S00004.798 | unknown protein |
| OPB42693 | T4742_S00004.799 | unknown protein |
| OPB42698 | T4742_S00004.804 | unknown protein |
| OPB42704 | T4742_S00004.810 | unknown protein |
| OPB42705 | T4742_S00004.811 | unknown protein |
| OPB42711 | T4742_S00004.817 | Unknown protein |
| OPB42712 | T4742_S00004.818 | unknown protein |
| OPB42719 | T4742_S00004.825 | unknown protein |
| OPB42729 | T4742_S00004.835 | unknown protein |
| OPB42735 | T4742_S00004.841 | unknown protein |
| OPB42742 | T4742_S00004.848 | unknown protein |
| OPB42756 | T4742_S00004.862 | unknown protein |
| OPB42758 | T4742_S00004.864 | unknown protein |
| OPB42772 | T4742_S00012.9 | unknown protein |
| OPB42774 | T4742_S00012.11 | unknown protein |
| OPB42783 | T4742_S00012.20 | unknown protein |
| OPB42790 | T4742_S00012.27 | unknown protein |
| OPB42794 | T4742_S00012.31 | unknown protein |
| OPB42795 | T4742_S00012.32 | unknown protein |
| OPB42798 | T4742_S00012.35 | unknown protein |
| OPB42812 | T4742_S00012.49 | unknown protein |
| OPB42815 | T4742_S00012.52 | unknown protein |
| OPB42823 | T4742_S00012.60 | unknown protein |
| OPB42824 | T4742_S00012.61 | unknown protein |

| | | |
|---|---|---|
| OPB42836 | T4742_S00012.73 | unknown protein |
| OPB42839 | T4742_S00012.76 | unknown protein |
| OPB42841 | T4742_S00012.78 | unknown protein |
| OPB42843 | T4742_S00012.80 | unknown protein |
| OPB42846 | T4742_S00012.83 | unknown protein |
| OPB42847 | T4742_S00012.84 | unknown protein |
| OPB42849 | T4742_S00012.86 | unknown protein |
| OPB42852 | T4742_S00012.89 | unknown protein |
| OPB42853 | T4742_S00012.90 | unknown protein |
| OPB42855 | T4742_S00012.92 | unknown protein |
| OPB42856 | T4742_S00012.93 | unknown protein |
| OPB42859 | T4742_S00012.96 | unknown protein |
| OPB42861 | T4742_S00012.98 | unknown protein |
| OPB42864 | T4742_S00012.101 | unknown protein |
| OPB42873 | T4742_S00012.110 | unknown protein |
| OPB42876 | T4742_S00012.113 | unknown protein |
| OPB42887 | T4742_S00012.124 | unknown protein |
| OPB42892 | T4742_S00012.129 | unknown protein |
| OPB42901 | T4742_S00012.138 | unknown protein |
| OPB42907 | T4742_S00012.144 | unknown protein |
| OPB42909 | T4742_S00012.146 | unknown protein |
| OPB42912 | T4742_S00012.149 | unknown protein |
| OPB42914 | T4742_S00012.151 | unknown protein |
| OPB42926 | T4742_S00012.163 | unknown protein |
| OPB42927 | T4742_S00012.164 | unknown protein |
| OPB42928 | T4742_S00012.165 | unknown protein |
| OPB42937 | T4742_S00012.174 | unknown protein |
| OPB42939 | T4742_S00012.176 | unknown protein |
| OPB42941 | T4742_S00012.178 | unknown protein |
| OPB42948 | T4742_S00012.185 | unknown protein |
| OPB42950 | T4742_S00012.187 | unknown protein |
| OPB42951 | T4742_S00012.188 | unknown protein |
| OPB42953 | T4742_S00012.190 | unknown protein |
| OPB42954 | T4742_S00012.191 | unknown protein |
| OPB42956 | T4742_S00012.193 | unknown protein |
| OPB42958 | T4742_S00012.195 | unknown protein |

| | | |
|---|---|---|
| OPB42961 | T4742_S00012.198 | unknown protein |
| OPB42965 | T4742_S00012.202 | unknown protein |
| OPB42969 | T4742_S00012.206 | unknown protein |
| OPB42970 | T4742_S00012.207 | unknown protein |
| OPB42975 | T4742_S00012.212 | unknown protein |
| OPB42979 | T4742_S00012.216 | unknown protein |
| OPB42980 | T4742_S00012.217 | unknown protein |
| OPB42981 | T4742_S00012.218 | unknown protein |
| OPB42984 | T4742_S00012.221 | unknown protein |
| OPB42991 | T4742_S00012.228 | unknown protein |
| OPB42998 | T4742_S00012.235 | unknown protein |
| OPB43000 | T4742_S00012.237 | unknown protein |
| OPB43008 | T4742_S00012.245 | unknown protein |
| OPB43009 | T4742_S00012.246 | unknown protein |
| OPB43010 | T4742_S00012.247 | unknown protein |
| OPB43011 | T4742_S00012.248 | unknown protein |
| OPB43012 | T4742_S00012.249 | unknown protein |
| OPB43013 | T4742_S00012.250 | unknown protein |
| OPB43014 | T4742_S00012.251 | unknown protein |
| OPB43023 | T4742_S00012.260 | unknown protein |
| OPB43025 | T4742_S00012.262 | unknown protein |
| OPB43026 | T4742_S00012.263 | unknown protein |
| OPB43032 | T4742_S00012.269 | unknown protein |
| OPB43036 | T4742_S00012.273 | unknown protein |
| OPB43044 | T4742_S00012.281 | unknown protein |
| OPB43045 | T4742_S00012.282 | unknown protein |
| OPB43048 | T4742_S00012.285 | unknown protein |
| OPB43049 | T4742_S00012.286 | unknown protein |
| OPB43051 | T4742_S00012.288 | unknown protein |
| OPB43052 | T4742_S00012.289 | unknown protein |
| OPB43053 | T4742_S00012.290 | unknown protein |
| OPB43054 | T4742_S00012.291 | unknown protein |
| OPB43058 | T4742_S00012.295 | unknown protein |
| OPB43061 | T4742_S00012.298 | unknown protein |
| OPB43062 | T4742_S00012.299 | unknown protein |
| OPB43063 | T4742_S00012.300 | unknown protein |

| | | |
|---|---|---|
| OPB43065 | T4742_S00012.302 | unknown protein |
| OPB43069 | T4742_S00012.306 | unknown protein |
| OPB43070 | T4742_S00012.307 | unknown protein |
| OPB43073 | T4742_S00012.310 | unknown protein |
| OPB43074 | T4742_S00012.311 | unknown protein |
| OPB43077 | T4742_S00012.314 | unknown protein |
| OPB43080 | T4742_S00012.317 | unknown protein |
| OPB43085 | T4742_S00012.322 | unknown protein |
| OPB43086 | T4742_S00012.323 | unknown protein |
| OPB43090 | T4742_S00012.327 | unknown protein |
| OPB43092 | T4742_S00012.329 | unknown protein |
| OPB43093 | T4742_S00012.330 | unknown protein |
| OPB43095 | T4742_S00012.332 | unknown protein |
| OPB43102 | T4742_S00012.339 | unknown protein |
| OPB43103 | T4742_S00012.340 | unknown protein |
| OPB43104 | T4742_S00012.341 | unknown protein |
| OPB43106 | T4742_S00012.343 | unknown protein |
| OPB43109 | T4742_S00012.346 | unknown protein |
| OPB43110 | T4742_S00012.347 | unknown protein |
| OPB43111 | T4742_S00012.348 | unknown protein |
| OPB43112 | T4742_S00012.349 | unknown protein |
| OPB43115 | T4742_S00012.352 | unknown protein |
| OPB43125 | T4742_S00012.362 | unknown protein |
| OPB43128 | T4742_S00012.365 | unknown protein |
| OPB43129 | T4742_S00012.366 | unknown protein |
| OPB43130 | T4742_S00012.367 | unknown protein |
| OPB43131 | T4742_S00012.368 | unknown protein |
| OPB43141 | T4742_S00012.378 | unknown protein |
| OPB43142 | T4742_S00012.379 | unknown protein |
| OPB43145 | T4742_S00012.382 | unknown protein |
| OPB43157 | T4742_S00012.394 | unknown protein |
| OPB43163 | T4742_S00012.400 | unknown protein |
| OPB43166 | T4742_S00012.403 | unknown protein |
| OPB43170 | T4742_S00012.407 | unknown protein |
| OPB43174 | T4742_S00012.411 | unknown protein |
| OPB43179 | T4742_S00012.416 | unknown protein |

| | | |
|---|---|---|
| OPB43181 | T4742_S00012.418 | unknown protein |
| OPB43184 | T4742_S00012.421 | unknown protein |
| OPB43191 | T4742_S00012.428 | unknown protein |
| OPB43196 | T4742_S00012.433 | unknown protein |
| OPB43203 | T4742_S00022.6 | unknown protein |
| OPB43204 | T4742_S00022.7 | unknown protein |
| OPB43205 | T4742_S00022.8 | unknown protein |
| OPB43206 | T4742_S00022.9 | unknown protein |
| OPB43207 | T4742_S00022.10 | unknown protein |
| OPB43210 | T4742_S00022.13 | unknown protein |
| OPB43217 | T4742_S00022.20 | unknown protein |
| OPB43218 | T4742_S00022.21 | unknown protein |
| OPB43221 | T4742_S00022.24 | unknown protein |
| OPB43227 | T4742_S00022.30 | unknown protein |
| OPB43228 | T4742_S00022.31 | unknown protein |
| OPB43229 | T4742_S00022.32 | unknown protein |
| OPB43232 | T4742_S00022.35 | unknown protein |
| OPB43233 | T4742_S00022.36 | unknown protein |
| OPB43235 | T4742_S00022.38 | unknown protein |
| OPB43237 | T4742_S00022.40 | unknown protein |
| OPB43238 | T4742_S00022.41 | unknown protein |
| OPB43242 | T4742_S00022.45 | unknown protein |
| OPB43245 | T4742_S00022.48 | unknown protein |
| OPB43248 | T4742_S00022.51 | unknown protein |
| OPB43249 | T4742_S00022.52 | unknown protein |
| OPB43250 | T4742_S00022.54 | unknown protein |
| OPB43251 | T4742_S00022.55 | unknown protein |
| OPB43252 | T4742_S00022.56 | unknown protein |
| OPB43253 | T4742_S00022.57 | unknown protein |
| OPB43254 | T4742_S00022.58 | unknown protein |
| OPB43255 | T4742_S00022.59 | unknown protein |
| OPB43264 | T4742_S00022.68 | unknown protein |
| OPB43270 | T4742_S00019.3 | unknown protein |
| OPB43271 | T4742_S00019.4 | unknown protein |
| OPB43273 | T4742_S00019.6 | unknown protein |
| OPB43279 | T4742_S00019.12 | unknown protein |

| | | |
|---|---|---|
| OPB43285 | T4742_S00019.18 | unknown protein |
| OPB43287 | T4742_S00019.20 | unknown protein |
| OPB43298 | T4742_S00019.31 | unknown protein |
| OPB43300 | T4742_S00019.33 | unknown protein |
| OPB43311 | T4742_S00019.44 | unknown protein |
| OPB43313 | T4742_S00019.46 | unknown protein |
| OPB43315 | T4742_S00019.48 | unknown protein |
| OPB43316 | T4742_S00019.49 | unknown protein |
| OPB43317 | T4742_S00019.50 | unknown protein |
| OPB43321 | T4742_S00019.54 | unknown protein |
| OPB43324 | T4742_S00019.57 | unknown protein |
| OPB43326 | T4742_S00019.59 | unknown protein |
| OPB43327 | T4742_S00019.60 | unknown protein |
| OPB43328 | T4742_S00019.61 | unknown protein |
| OPB43329 | T4742_S00019.62 | unknown protein |
| OPB43331 | T4742_S00019.64 | unknown protein |
| OPB43343 | T4742_S00019.76 | unknown protein |
| OPB43348 | T4742_S00019.81 | unknown protein |
| OPB43349 | T4742_S00019.82 | unknown protein |
| OPB43366 | T4742_S00019.99 | unknown protein |
| OPB43369 | T4742_S00019.102 | unknown protein |
| OPB43370 | T4742_S00019.103 | unknown protein |
| OPB43371 | T4742_S00019.104 | unknown protein |
| OPB43376 | T4742_S00019.109 | unknown protein |
| OPB43377 | T4742_S00019.110 | unknown protein |
| OPB43379 | T4742_S00019.112 | unknown protein |
| OPB43380 | T4742_S00019.113 | unknown protein |
| OPB43381 | T4742_S00019.114 | unknown protein |
| OPB43383 | T4742_S00019.116 | unknown protein |
| OPB43385 | T4742_S00019.118 | unknown protein |
| OPB43387 | T4742_S00019.120 | unknown protein |
| OPB43389 | T4742_S00019.122 | unknown protein |
| OPB43397 | T4742_S00019.130 | unknown protein |
| OPB43416 | T4742_S00019.149 | unknown protein |
| OPB43422 | T4742_S00019.155 | unknown protein |
| OPB43425 | T4742_S00019.158 | unknown protein |

| | | |
|---|---|---|
| OPB43426 | T4742_S00019.159 | unknown protein |
| OPB43433 | T4742_S00019.166 | unknown protein |
| OPB43442 | T4742_S00019.175 | unknown protein |
| OPB43449 | T4742_S00019.182 | unknown protein |
| OPB43450 | T4742_S00019.183 | unknown protein |
| OPB43454 | T4742_S00019.187 | unknown protein |
| OPB43457 | T4742_S00019.190 | unknown protein |
| OPB43460 | T4742_S00019.193 | unknown protein |
| OPB43473 | T4742_S00019.206 | unknown protein |
| OPB43475 | T4742_S00019.208 | unknown protein |
| OPB43484 | T4742_S00016.4 | unknown protein |
| OPB43488 | T4742_S00016.8 | unknown protein |
| OPB43493 | T4742_S00016.13 | unknown protein |
| OPB43496 | T4742_S00016.16 | unknown protein |
| OPB43500 | T4742_S00016.20 | unknown protein |
| OPB43507 | T4742_S00016.27 | unknown protein |
| OPB43517 | T4742_S00016.37 | unknown protein |
| OPB43520 | T4742_S00016.40 | unknown protein |
| OPB43529 | T4742_S00016.49 | unknown protein |
| OPB43541 | T4742_S00016.61 | unknown protein |
| OPB43543 | T4742_S00016.63 | unknown protein |
| OPB43545 | T4742_S00016.65 | unknown protein |
| OPB43546 | T4742_S00016.66 | unknown protein |
| OPB43547 | T4742_S00016.67 | unknown protein |
| OPB43548 | T4742_S00016.68 | unknown protein |
| OPB43549 | T4742_S00016.69 | unknown protein |
| OPB43551 | T4742_S00016.71 | unknown protein |
| OPB43562 | T4742_S00016.82 | unknown protein |
| OPB43565 | T4742_S00016.85 | unknown protein |
| OPB43568 | T4742_S00016.88 | unknown protein |
| OPB43569 | T4742_S00016.89 | unknown protein |
| OPB43570 | T4742_S00016.90 | unknown protein |
| OPB43572 | T4742_S00016.92 | unknown protein |
| OPB43576 | T4742_S00016.96 | unknown protein |
| OPB43577 | T4742_S00016.97 | unknown protein |
| OPB43578 | T4742_S00016.98 | unknown protein |

| | | |
|---|---|---|
| OPB43580 | T4742_S00016.100 | unknown protein |
| OPB43585 | T4742_S00016.105 | unknown protein |
| OPB43590 | T4742_S00016.110 | unknown protein |
| OPB43592 | T4742_S00016.112 | unknown protein |
| OPB43594 | T4742_S00016.114 | unknown protein |
| OPB43596 | T4742_S00016.116 | unknown protein |
| OPB43599 | T4742_S00016.119 | unknown protein |
| OPB43601 | T4742_S00016.121 | unknown protein |
| OPB43602 | T4742_S00016.122 | unknown protein |
| OPB43608 | T4742_S00016.128 | unknown protein |
| OPB43609 | T4742_S00016.129 | unknown protein |
| OPB43611 | T4742_S00016.131 | unknown protein |
| OPB43616 | T4742_S00016.136 | unknown protein |
| OPB43622 | T4742_S00016.142 | unknown protein |
| OPB43624 | T4742_S00016.144 | Unknown protein |
| OPB43626 | T4742_S00016.146 | unknown protein |
| OPB43629 | T4742_S00016.149 | unknown protein |
| OPB43631 | T4742_S00016.151 | unknown protein |
| OPB43633 | T4742_S00016.153 | unknown protein |
| OPB43636 | T4742_S00016.156 | unknown protein |
| OPB43644 | T4742_S00016.164 | unknown protein |
| OPB43658 | T4742_S00016.178 | unknown protein |
| OPB43659 | T4742_S00016.179 | unknown protein |
| OPB43670 | T4742_S00016.190 | unknown protein |
| OPB43671 | T4742_S00016.191 | unknown protein |
| OPB43675 | T4742_S00016.195 | unknown protein |
| OPB43676 | T4742_S00016.196 | unknown protein |
| OPB43678 | T4742_S00016.198 | unknown protein |
| OPB43680 | T4742_S00016.200 | unknown protein |
| OPB43683 | T4742_S00016.203 | unknown protein |
| OPB43685 | T4742_S00016.205 | unknown protein |
| OPB43686 | T4742_S00016.206 | unknown protein |
| OPB43694 | T4742_S00016.214 | unknown protein |
| OPB43698 | T4742_S00016.218 | unknown protein |
| OPB43700 | T4742_S00016.220 | unknown protein |
| OPB43701 | T4742_S00016.221 | unknown protein |

| | | |
|---|---|---|
| OPB43703 | T4742_S00016.223 | unknown protein |
| OPB43704 | T4742_S00016.224 | unknown protein |
| OPB43705 | T4742_S00016.225 | unknown protein |
| OPB43707 | T4742_S00016.227 | unknown protein |
| OPB43714 | T4742_S00016.234 | unknown protein |
| OPB43717 | T4742_S00016.237 | unknown protein |
| OPB43719 | T4742_S00016.239 | unknown protein |
| OPB43720 | T4742_S00016.240 | unknown protein |
| OPB43721 | T4742_S00016.241 | unknown protein |
| OPB43727 | T4742_S00016.247 | unknown protein |
| OPB43744 | T4742_S00003.15 | unknown protein |
| OPB43745 | T4742_S00003.16 | unknown protein |
| OPB43752 | T4742_S00003.23 | unknown protein |
| OPB43759 | T4742_S00003.30 | unknown protein |
| OPB43772 | T4742_S00003.43 | unknown protein |
| OPB43774 | T4742_S00003.45 | unknown protein |
| OPB43777 | T4742_S00003.48 | unknown protein |
| OPB43781 | T4742_S00003.52 | unknown protein |
| OPB43787 | T4742_S00003.58 | unknown protein |
| OPB43795 | T4742_S00003.66 | unknown protein |
| OPB43796 | T4742_S00003.67 | unknown protein |
| OPB43802 | T4742_S00003.73 | unknown protein |
| OPB43805 | T4742_S00003.76 | unknown protein |
| OPB43815 | T4742_S00003.86 | unknown protein |
| OPB43818 | T4742_S00003.89 | unknown protein |
| OPB43824 | T4742_S00003.95 | unknown protein |
| OPB43825 | T4742_S00003.96 | unknown protein |
| OPB43827 | T4742_S00003.98 | unknown protein |
| OPB43830 | T4742_S00003.101 | unknown protein |
| OPB43833 | T4742_S00003.104 | unknown protein |
| OPB43838 | T4742_S00003.109 | unknown protein |
| OPB43839 | T4742_S00003.110 | unknown protein |
| OPB43847 | T4742_S00003.118 | unknown protein |
| OPB43856 | T4742_S00003.127 | unknown protein |
| OPB43858 | T4742_S00003.129 | unknown protein |
| OPB43860 | T4742_S00003.131 | unknown protein |

| | | |
|---|---|---|
| OPB43862 | T4742_S00003.133 | unknown protein |
| OPB43863 | T4742_S00003.134 | unknown protein |
| OPB43864 | T4742_S00003.135 | unknown protein |
| OPB43866 | T4742_S00003.137 | unknown protein |
| OPB43871 | T4742_S00003.142 | unknown protein |
| OPB43879 | T4742_S00003.150 | unknown protein |
| OPB43886 | T4742_S00003.157 | unknown protein |
| OPB43887 | T4742_S00003.158 | unknown protein |
| OPB43890 | T4742_S00003.161 | unknown protein |
| OPB43891 | T4742_S00003.162 | unknown protein |
| OPB43894 | T4742_S00003.165 | unknown protein |
| OPB43901 | T4742_S00003.172 | unknown protein |
| OPB43902 | T4742_S00003.173 | unknown protein |
| OPB43905 | T4742_S00003.176 | unknown protein |
| OPB43906 | T4742_S00003.177 | unknown protein |
| OPB43908 | T4742_S00003.179 | unknown protein |
| OPB43909 | T4742_S00003.180 | unknown protein |
| OPB43910 | T4742_S00003.181 | unknown protein |
| OPB43912 | T4742_S00003.183 | unknown protein |
| OPB43914 | T4742_S00003.185 | unknown protein |
| OPB43915 | T4742_S00003.186 | unknown protein |
| OPB43916 | T4742_S00003.187 | unknown protein |
| OPB43920 | T4742_S00003.191 | unknown protein |
| OPB43923 | T4742_S00003.194 | unknown protein |
| OPB43928 | T4742_S00003.199 | unknown protein |
| OPB43929 | T4742_S00003.200 | unknown protein |
| OPB43938 | T4742_S00003.209 | unknown protein |
| OPB43945 | T4742_S00003.216 | unknown protein |
| OPB43947 | T4742_S00003.218 | unknown protein |
| OPB43955 | T4742_S00003.226 | unknown protein |
| OPB43959 | T4742_S00003.230 | unknown protein |
| OPB43960 | T4742_S00003.231 | unknown protein |
| OPB43961 | T4742_S00003.232 | unknown protein |
| OPB43963 | T4742_S00003.234 | unknown protein |
| OPB43965 | T4742_S00003.236 | unknown protein |
| OPB43974 | T4742_S00003.245 | unknown protein |

| | | |
|---|---|---|
| OPB43976 | T4742_S00003.247 | unknown protein |
| OPB43977 | T4742_S00003.248 | unknown protein |
| OPB43980 | T4742_S00003.251 | unknown protein |
| OPB43986 | T4742_S00003.257 | Unknown protein |
| OPB43989 | T4742_S00003.260 | unknown protein |
| OPB43992 | T4742_S00003.263 | unknown protein |
| OPB43994 | T4742_S00003.265 | unknown protein |
| OPB44005 | T4742_S00003.276 | unknown protein |
| OPB44008 | T4742_S00003.279 | unknown protein |
| OPB44013 | T4742_S00003.284 | unknown protein |
| OPB44014 | T4742_S00003.285 | unknown protein |
| OPB44021 | T4742_S00003.292 | unknown protein |
| OPB44023 | T4742_S00003.294 | unknown protein |
| OPB44026 | T4742_S00003.297 | unknown protein |
| OPB44031 | T4742_S00003.302 | unknown protein |
| OPB44037 | T4742_S00003.308 | Unknown protein |
| OPB44038 | T4742_S00003.309 | unknown protein |
| OPB44041 | T4742_S00003.312 | unknown protein |
| OPB44042 | T4742_S00003.313 | unknown protein |
| OPB44044 | T4742_S00003.315 | unknown protein |
| OPB44045 | T4742_S00003.316 | unknown protein |
| OPB44047 | T4742_S00003.318 | unknown protein |
| OPB44054 | T4742_S00003.325 | unknown protein |
| OPB44056 | T4742_S00003.327 | unknown protein |
| OPB44061 | T4742_S00003.332 | unknown protein |
| OPB44063 | T4742_S00003.334 | unknown protein |
| OPB44080 | T4742_S00003.351 | unknown protein |
| OPB44081 | T4742_S00003.352 | unknown protein |
| OPB44082 | T4742_S00003.353 | unknown protein |
| OPB44084 | T4742_S00003.355 | unknown protein |
| OPB44087 | T4742_S00003.358 | unknown protein |
| OPB44088 | T4742_S00003.359 | unknown protein |
| OPB44089 | T4742_S00003.360 | unknown protein |
| OPB44090 | T4742_S00003.361 | unknown protein |
| OPB44093 | T4742_S00003.364 | unknown protein |
| OPB44096 | T4742_S00003.367 | unknown protein |

| | | |
|---|---|---|
| OPB44097 | T4742_S00003.368 | unknown protein |
| OPB44098 | T4742_S00003.369 | unknown protein |
| OPB44099 | T4742_S00003.370 | unknown protein |
| OPB44101 | T4742_S00003.372 | unknown protein |
| OPB44102 | T4742_S00003.373 | unknown protein |
| OPB44103 | T4742_S00003.374 | unknown protein |
| OPB44107 | T4742_S00003.378 | unknown protein |
| OPB44113 | T4742_S00003.384 | unknown protein |
| OPB44117 | T4742_S00003.388 | unknown protein |
| OPB44119 | T4742_S00003.390 | unknown protein |
| OPB44125 | T4742_S00003.396 | unknown protein |
| OPB44128 | T4742_S00003.399 | unknown protein |
| OPB44129 | T4742_S00003.400 | unknown protein |
| OPB44130 | T4742_S00003.401 | unknown protein |
| OPB44131 | T4742_S00003.402 | unknown protein |
| OPB44138 | T4742_S00003.409 | unknown protein |
| OPB44144 | T4742_S00003.415 | unknown protein |
| OPB44148 | T4742_S00003.419 | unknown protein |
| OPB44151 | T4742_S00003.422 | unknown protein |
| OPB44154 | T4742_S00003.425 | unknown protein |
| OPB44158 | T4742_S00003.429 | unknown protein |
| OPB44164 | T4742_S00003.435 | unknown protein |
| OPB44168 | T4742_S00003.439 | unknown protein |
| OPB44175 | T4742_S00003.446 | unknown protein |
| OPB44177 | T4742_S00003.448 | unknown protein |
| OPB44179 | T4742_S00003.450 | unknown protein |
| OPB44180 | T4742_S00003.451 | unknown protein |
| OPB44186 | T4742_S00003.457 | unknown protein |
| OPB44188 | T4742_S00003.459 | unknown protein |
| OPB44190 | T4742_S00003.461 | unknown protein |
| OPB44194 | T4742_S00003.465 | unknown protein |
| OPB44195 | T4742_S00003.466 | unknown protein |
| OPB44197 | T4742_S00003.468 | unknown protein |
| OPB44198 | T4742_S00003.469 | unknown protein |
| OPB44200 | T4742_S00003.471 | unknown protein |
| OPB44208 | T4742_S00003.479 | unknown protein |

| | | |
|---|---|---|
| OPB44209 | T4742_S00003.480 | unknown protein |
| OPB44215 | T4742_S00003.486 | unknown protein |
| OPB44216 | T4742_S00003.487 | unknown protein |
| OPB44218 | T4742_S00003.489 | unknown protein |
| OPB44224 | T4742_S00003.495 | unknown protein |
| OPB44225 | T4742_S00003.496 | unknown protein |
| OPB44226 | T4742_S00003.497 | unknown protein |
| OPB44229 | T4742_S00003.500 | unknown protein |
| OPB44231 | T4742_S00003.502 | unknown protein |
| OPB44232 | T4742_S00003.503 | unknown protein |
| OPB44239 | T4742_S00003.510 | unknown protein |
| OPB44241 | T4742_S00003.512 | unknown protein |
| OPB44243 | T4742_S00003.514 | unknown protein |
| OPB44244 | T4742_S00003.515 | unknown protein |
| OPB44246 | T4742_S00003.517 | unknown protein |
| OPB44248 | T4742_S00003.519 | unknown protein |
| OPB44249 | T4742_S00003.520 | unknown protein |
| OPB44250 | T4742_S00003.521 | unknown protein |
| OPB44255 | T4742_S00003.526 | unknown protein |
| OPB44260 | T4742_S00003.531 | unknown protein |
| OPB44262 | T4742_S00003.533 | unknown protein |
| OPB44272 | T4742_S00003.543 | unknown protein |
| OPB44278 | T4742_S00003.549 | unknown protein |
| OPB44280 | T4742_S00003.551 | unknown protein |
| OPB44281 | T4742_S00003.552 | unknown protein |
| OPB44282 | T4742_S00003.553 | unknown protein |
| OPB44285 | T4742_S00003.556 | unknown protein |
| OPB44288 | T4742_S00003.559 | unknown protein |
| OPB44290 | T4742_S00003.561 | unknown protein |
| OPB44291 | T4742_S00003.562 | unknown protein |
| OPB44293 | T4742_S00003.564 | unknown protein |
| OPB44294 | T4742_S00003.565 | unknown protein |
| OPB44306 | T4742_S00003.577 | unknown protein |
| OPB44311 | T4742_S00003.582 | unknown protein |
| OPB44312 | T4742_S00003.583 | unknown protein |
| OPB44313 | T4742_S00003.584 | unknown protein |

| | | |
|---|---|---|
| OPB44319 | T4742_S00003.590 | unknown protein |
| OPB44321 | T4742_S00003.592 | unknown protein |
| OPB44322 | T4742_S00003.593 | unknown protein |
| OPB44323 | T4742_S00003.594 | unknown protein |
| OPB44324 | T4742_S00003.595 | unknown protein |
| OPB44328 | T4742_S00003.599 | unknown protein |
| OPB44330 | T4742_S00003.601 | unknown protein |
| OPB44337 | T4742_S00003.608 | unknown protein |
| OPB44341 | T4742_S00003.612 | unknown protein |
| OPB44342 | T4742_S00003.613 | unknown protein |
| OPB44346 | T4742_S00003.617 | unknown protein |
| OPB44348 | T4742_S00003.619 | unknown protein |
| OPB44350 | T4742_S00003.621 | unknown protein |
| OPB44354 | T4742_S00003.625 | unknown protein |
| OPB44355 | T4742_S00003.626 | unknown protein |
| OPB44361 | T4742_S00003.632 | unknown protein |
| OPB44362 | T4742_S00003.633 | unknown protein |
| OPB44363 | T4742_S00003.634 | unknown protein |
| OPB44369 | T4742_S00003.640 | unknown protein |
| OPB44370 | T4742_S00003.641 | unknown protein |
| OPB44371 | T4742_S00003.642 | unknown protein |
| OPB44375 | T4742_S00003.646 | unknown protein |
| OPB44379 | T4742_S00003.650 | unknown protein |
| OPB44381 | T4742_S00003.652 | unknown protein |
| OPB44382 | T4742_S00003.653 | unknown protein |
| OPB44383 | T4742_S00003.654 | unknown protein |
| OPB44388 | T4742_S00003.659 | unknown protein |
| OPB44389 | T4742_S00003.660 | unknown protein |
| OPB44390 | T4742_S00003.661 | unknown protein |
| OPB44392 | T4742_S00003.663 | unknown protein |
| OPB44393 | T4742_S00003.664 | unknown protein |
| OPB44397 | T4742_S00003.668 | unknown protein |
| OPB44401 | T4742_S00003.672 | unknown protein |
| OPB44403 | T4742_S00003.674 | unknown protein |
| OPB44405 | T4742_S00003.676 | unknown protein |
| OPB44406 | T4742_S00003.677 | unknown protein |

| | | |
|---|---|---|
| OPB44410 | T4742_S00003.681 | unknown protein |
| OPB44412 | T4742_S00003.683 | unknown protein |
| OPB44421 | T4742_S00003.693 | unknown protein |
| OPB44422 | T4742_S00003.694 | unknown protein |
| OPB44428 | T4742_S00003.700 | unknown protein |
| OPB44430 | T4742_S00003.702 | unknown protein |
| OPB44431 | T4742_S00003.703 | unknown protein |
| OPB44433 | T4742_S00003.705 | unknown protein |
| OPB44439 | T4742_S00003.711 | unknown protein |
| OPB44442 | T4742_S00003.714 | unknown protein |
| OPB44443 | T4742_S00003.715 | unknown protein |
| OPB44462 | T4742_S00003.734 | unknown protein |
| OPB44465 | T4742_S00003.737 | unknown protein |
| OPB44467 | T4742_S00003.739 | unknown protein |
| OPB44472 | T4742_S00003.744 | unknown protein |
| OPB44476 | T4742_S00003.748 | unknown protein |
| OPB44491 | T4742_S00003.763 | unknown protein |
| OPB44496 | T4742_S00003.768 | unknown protein |
| OPB44498 | T4742_S00003.770 | unknown protein |
| OPB44499 | T4742_S00003.771 | unknown protein |
| OPB44512 | T4742_S00003.784 | unknown protein |
| OPB44521 | T4742_S00003.793 | unknown protein |
| OPB44527 | T4742_S00003.799 | unknown protein |
| OPB44530 | T4742_S00003.802 | unknown protein |
| OPB44532 | T4742_S00003.804 | unknown protein |
| OPB44543 | T4742_S00003.815 | unknown protein |
| OPB44550 | T4742_S00003.822 | unknown protein |
| OPB44556 | T4742_S00003.828 | unknown protein |
| OPB44557 | T4742_S00003.829 | unknown protein |
| OPB44560 | T4742_S00003.832 | unknown protein |
| OPB44563 | T4742_S00003.835 | unknown protein |
| OPB44571 | T4742_S00003.843 | unknown protein |
| OPB44572 | T4742_S00003.844 | unknown protein |
| OPB44576 | T4742_S00003.848 | unknown protein |
| OPB44577 | T4742_S00003.849 | unknown protein |
| OPB44581 | T4742_S00003.853 | unknown protein |

| | | |
|---|---|---|
| OPB44582 | T4742_S00003.854 | unknown protein |
| OPB44585 | T4742_S00003.857 | unknown protein |
| OPB44589 | T4742_S00003.861 | unknown protein |
| OPB44602 | T4742_S00003.874 | unknown protein |
| OPB44606 | T4742_S00003.878 | unknown protein |
| OPB44611 | T4742_S00003.883 | unknown protein |
| OPB44614 | T4742_S00003.886 | unknown protein |
| OPB44620 | T4742_S00003.892 | unknown protein |
| OPB44625 | T4742_S00003.897 | unknown protein |
| OPB44626 | T4742_S00003.898 | unknown protein |
| OPB44627 | T4742_S00003.899 | unknown protein |
| OPB44630 | T4742_S00003.902 | unknown protein |
| OPB44632 | T4742_S00003.904 | unknown protein |
| OPB44635 | T4742_S00003.907 | unknown protein |
| OPB44643 | T4742_S00003.915 | unknown protein |
| OPB44644 | T4742_S00003.916 | unknown protein |
| OPB44648 | T4742_S00003.920 | unknown protein |
| OPB44652 | T4742_S00003.924 | unknown protein |
| OPB44653 | T4742_S00003.925 | unknown protein |
| OPB44654 | T4742_S00003.926 | unknown protein |
| OPB44656 | T4742_S00003.928 | unknown protein |
| OPB44659 | T4742_S00003.931 | unknown protein |
| OPB44660 | T4742_S00003.932 | unknown protein |
| OPB44663 | T4742_S00003.935 | unknown protein |
| OPB44664 | T4742_S00003.936 | unknown protein |
| OPB44666 | T4742_S00003.938 | unknown protein |
| OPB44671 | T4742_S00003.943 | unknown protein |
| OPB44676 | T4742_S00003.948 | unknown protein |
| OPB44677 | T4742_S00003.949 | unknown protein |
| OPB44678 | T4742_S00003.950 | unknown protein |
| OPB44683 | T4742_S00003.955 | unknown protein |
| OPB44686 | T4742_S00003.958 | unknown protein |
| OPB44689 | T4742_S00003.961 | unknown protein |
| OPB44692 | T4742_S00003.964 | unknown protein |
| OPB44698 | T4742_S00013.2 | unknown protein |
| OPB44699 | T4742_S00013.3 | unknown protein |

| | | |
|---|---|---|
| OPB44707 | T4742_S00013.11 | unknown protein |
| OPB44710 | T4742_S00013.14 | unknown protein |
| OPB44714 | T4742_S00013.18 | unknown protein |
| OPB44721 | T4742_S00013.26 | unknown protein |
| OPB44741 | T4742_S00013.46 | unknown protein |
| OPB44742 | T4742_S00013.47 | unknown protein |
| OPB44745 | T4742_S00013.50 | unknown protein |
| OPB44746 | T4742_S00013.51 | unknown protein |
| OPB44748 | T4742_S00013.53 | unknown protein |
| OPB44749 | T4742_S00013.54 | unknown protein |
| OPB44751 | T4742_S00013.56 | unknown protein |
| OPB44761 | T4742_S00013.66 | unknown protein |
| OPB44762 | T4742_S00013.67 | unknown protein |
| OPB44764 | T4742_S00013.69 | unknown protein |
| OPB44769 | T4742_S00013.74 | unknown protein |
| OPB44774 | T4742_S00013.79 | unknown protein |
| OPB44775 | T4742_S00013.80 | unknown protein |
| OPB44777 | T4742_S00013.82 | unknown protein |
| OPB44787 | T4742_S00013.92 | unknown protein |
| OPB44799 | T4742_S00013.104 | Unknown protein |
| OPB44812 | T4742_S00013.117 | unknown protein |
| OPB44814 | T4742_S00013.119 | unknown protein |
| OPB44815 | T4742_S00013.120 | unknown protein |
| OPB44826 | T4742_S00013.131 | unknown protein |
| OPB44827 | T4742_S00013.132 | unknown protein |
| OPB44845 | T4742_S00013.150 | unknown protein |
| OPB44849 | T4742_S00013.154 | unknown protein |
| OPB44851 | T4742_S00013.156 | unknown protein |
| OPB44853 | T4742_S00013.158 | unknown protein |
| OPB44858 | T4742_S00013.163 | unknown protein |
| OPB44868 | T4742_S00013.173 | unknown protein |
| OPB44874 | T4742_S00013.179 | unknown protein |
| OPB44888 | T4742_S00013.193 | unknown protein |
| OPB44889 | T4742_S00013.194 | unknown protein |
| OPB44893 | T4742_S00013.198 | unknown protein |
| OPB44896 | T4742_S00013.201 | unknown protein |

| | | |
|---|---|---|
| OPB44897 | T4742_S00013.202 | unknown protein |
| OPB44898 | T4742_S00013.203 | unknown protein |
| OPB44904 | T4742_S00013.209 | unknown protein |
| OPB44906 | T4742_S00013.211 | unknown protein |
| OPB44907 | T4742_S00013.212 | unknown protein |
| OPB44912 | T4742_S00013.217 | unknown protein |
| OPB44913 | T4742_S00013.218 | unknown protein |
| OPB44915 | T4742_S00013.220 | unknown protein |
| OPB44916 | T4742_S00013.221 | unknown protein |
| OPB44917 | T4742_S00013.222 | unknown protein |
| OPB44922 | T4742_S00013.227 | unknown protein |
| OPB44925 | T4742_S00013.230 | unknown protein |
| OPB44930 | T4742_S00013.235 | unknown protein |
| OPB44933 | T4742_S00013.238 | unknown protein |
| OPB44934 | T4742_S00013.239 | unknown protein |
| OPB44936 | T4742_S00013.241 | unknown protein |
| OPB44942 | T4742_S00013.247 | unknown protein |
| OPB44945 | T4742_S00013.250 | unknown protein |
| OPB44947 | T4742_S00013.252 | unknown protein |
| OPB44948 | T4742_S00013.253 | unknown protein |
| OPB44950 | T4742_S00013.255 | unknown protein |
| OPB44955 | T4742_S00013.260 | unknown protein |
| OPB44960 | T4742_S00013.265 | unknown protein |
| OPB44966 | T4742_S00013.271 | unknown protein |
| OPB44967 | T4742_S00013.272 | unknown protein |
| OPB44970 | T4742_S00013.275 | unknown protein |
| OPB44975 | T4742_S00013.280 | unknown protein |
| OPB44976 | T4742_S00013.281 | unknown protein |
| OPB44977 | T4742_S00013.282 | unknown protein |
| OPB44978 | T4742_S00013.283 | unknown protein |
| OPB44981 | T4742_S00013.286 | unknown protein |
| OPB44984 | T4742_S00013.289 | unknown protein |
| OPB44985 | T4742_S00013.290 | unknown protein |
| OPB44986 | T4742_S00013.291 | unknown protein |
| OPB44994 | T4742_S00013.299 | unknown protein |
| OPB44995 | T4742_S00013.300 | unknown protein |

| | | |
|---|---|---|
| OPB44997 | T4742_S00013.302 | unknown protein |
| OPB44999 | T4742_S00013.304 | unknown protein |
| OPB45000 | T4742_S00013.305 | unknown protein |
| OPB45002 | T4742_S00013.307 | unknown protein |
| OPB45005 | T4742_S00013.310 | unknown protein |
| OPB45006 | T4742_S00013.311 | unknown protein |
| OPB45007 | T4742_S00013.312 | unknown protein |
| OPB45011 | T4742_S00013.316 | unknown protein |
| OPB45013 | T4742_S00013.318 | unknown protein |
| OPB45014 | T4742_S00013.319 | unknown protein |
| OPB45017 | T4742_S00013.322 | unknown protein |
| OPB45019 | T4742_S00013.324 | unknown protein |
| OPB45023 | T4742_S00013.328 | unknown protein |
| OPB45024 | T4742_S00013.329 | unknown protein |
| OPB45026 | T4742_S00013.331 | unknown protein |
| OPB45027 | T4742_S00013.332 | unknown protein |
| OPB45029 | T4742_S00013.334 | unknown protein |
| OPB45030 | T4742_S00013.335 | unknown protein |
| OPB45032 | T4742_S00013.337 | unknown protein |
| OPB45034 | T4742_S00013.339 | unknown protein |
| OPB45039 | T4742_S00013.344 | unknown protein |
| OPB45040 | T4742_S00013.345 | unknown protein |
| OPB45044 | T4742_S00013.349 | unknown protein |
| OPB45045 | T4742_S00013.350 | unknown protein |
| OPB45050 | T4742_S00013.355 | unknown protein |
| OPB45052 | T4742_S00013.357 | unknown protein |
| OPB45053 | T4742_S00013.358 | unknown protein |
| OPB45057 | T4742_S00013.362 | unknown protein |
| OPB45058 | T4742_S00013.363 | unknown protein |
| OPB45068 | T4742_S00013.373 | unknown protein |
| OPB45070 | T4742_S00013.375 | unknown protein |
| OPB45075 | T4742_S00013.380 | unknown protein |
| OPB45076 | T4742_S00013.381 | unknown protein |
| OPB45083 | T4742_S00013.388 | unknown protein |
| OPB45086 | T4742_S00013.391 | unknown protein |
| OPB45092 | T4742_S00013.397 | unknown protein |

| | | |
|---|---|---|
| OPB45094 | T4742_S00013.399 | unknown protein |
| OPB45097 | T4742_S00010.4 | unknown protein |
| OPB45099 | T4742_S00010.6 | unknown protein |
| OPB45107 | T4742_S00010.14 | unknown protein |
| OPB45108 | T4742_S00010.15 | unknown protein |
| OPB45113 | T4742_S00010.20 | unknown protein |
| OPB45114 | T4742_S00010.21 | unknown protein |
| OPB45117 | T4742_S00010.24 | unknown protein |
| OPB45121 | T4742_S00010.28 | unknown protein |
| OPB45122 | T4742_S00010.29 | unknown protein |
| OPB45125 | T4742_S00010.32 | unknown protein |
| OPB45128 | T4742_S00010.35 | unknown protein |
| OPB45130 | T4742_S00010.37 | unknown protein |
| OPB45133 | T4742_S00010.40 | unknown protein |
| OPB45134 | T4742_S00010.41 | unknown protein |
| OPB45138 | T4742_S00010.45 | unknown protein |
| OPB45142 | T4742_S00010.49 | unknown protein |
| OPB45150 | T4742_S00010.57 | unknown protein |
| OPB45156 | T4742_S00010.63 | unknown protein |
| OPB45159 | T4742_S00010.66 | unknown protein |
| OPB45160 | T4742_S00010.67 | unknown protein |
| OPB45162 | T4742_S00010.69 | unknown protein |
| OPB45165 | T4742_S00010.72 | unknown protein |
| OPB45166 | T4742_S00010.73 | unknown protein |
| OPB45173 | T4742_S00010.80 | unknown protein |
| OPB45180 | T4742_S00010.87 | unknown protein |
| OPB45188 | T4742_S00010.95 | unknown protein |
| OPB45190 | T4742_S00010.98 | unknown protein |
| OPB45191 | T4742_S00010.99 | unknown protein |
| OPB45200 | T4742_S00010.108 | unknown protein |
| OPB45204 | T4742_S00010.112 | unknown protein |
| OPB45221 | T4742_S00010.130 | unknown protein |
| OPB45223 | T4742_S00010.132 | unknown protein |
| OPB45232 | T4742_S00010.141 | unknown protein |
| OPB45233 | T4742_S00010.142 | unknown protein |
| OPB45238 | T4742_S00010.147 | unknown protein |

| | | |
|---|---|---|
| OPB45243 | T4742_S00010.152 | unknown protein |
| OPB45244 | T4742_S00010.153 | Unknown protein |
| OPB45252 | T4742_S00010.161 | unknown protein |
| OPB45254 | T4742_S00010.163 | unknown protein |
| OPB45259 | T4742_S00010.168 | unknown protein |
| OPB45265 | T4742_S00010.176 | unknown protein |
| OPB45269 | T4742_S00010.180 | unknown protein |
| OPB45272 | T4742_S00010.183 | unknown protein |
| OPB45274 | T4742_S00010.185 | unknown protein |
| OPB45277 | T4742_S00010.188 | unknown protein |
| OPB45279 | T4742_S00010.190 | unknown protein |
| OPB45280 | T4742_S00010.191 | unknown protein |
| OPB45281 | T4742_S00010.192 | unknown protein |
| OPB45285 | T4742_S00010.196 | unknown protein |
| OPB45288 | T4742_S00010.199 | unknown protein |
| OPB45290 | T4742_S00010.201 | unknown protein |
| OPB45292 | T4742_S00010.203 | unknown protein |
| OPB45297 | T4742_S00010.208 | unknown protein |
| OPB45298 | T4742_S00010.209 | unknown protein |
| OPB45300 | T4742_S00010.211 | unknown protein |
| OPB45301 | T4742_S00010.212 | unknown protein |
| OPB45302 | T4742_S00010.213 | unknown protein |
| OPB45308 | T4742_S00010.219 | unknown protein |
| OPB45317 | T4742_S00010.228 | unknown protein |
| OPB45324 | T4742_S00010.235 | unknown protein |
| OPB45327 | T4742_S00010.238 | unknown protein |
| OPB45335 | T4742_S00010.246 | unknown protein |
| OPB45336 | T4742_S00010.247 | unknown protein |
| OPB45338 | T4742_S00010.249 | unknown protein |
| OPB45339 | T4742_S00010.250 | unknown protein |
| OPB45340 | T4742_S00010.251 | unknown protein |
| OPB45347 | T4742_S00010.258 | unknown protein |
| OPB45354 | T4742_S00010.265 | unknown protein |
| OPB45355 | T4742_S00010.266 | unknown protein |
| OPB45362 | T4742_S00010.273 | unknown protein |
| OPB45364 | T4742_S00010.275 | unknown protein |

| | | |
|---|---|---|
| OPB45369 | T4742_S00010.280 | unknown protein |
| OPB45370 | T4742_S00010.281 | unknown protein |
| OPB45375 | T4742_S00010.286 | unknown protein |
| OPB45382 | T4742_S00010.293 | unknown protein |
| OPB45385 | T4742_S00010.296 | unknown protein |
| OPB45386 | T4742_S00010.297 | unknown protein |
| OPB45387 | T4742_S00010.298 | unknown protein |
| OPB45401 | T4742_S00010.312 | unknown protein |
| OPB45406 | T4742_S00010.317 | unknown protein |
| OPB45408 | T4742_S00010.319 | unknown protein |
| OPB45415 | T4742_S00010.326 | unknown protein |
| OPB45417 | T4742_S00010.328 | unknown protein |
| OPB45418 | T4742_S00010.329 | unknown protein |
| OPB45422 | T4742_S00010.333 | unknown protein |
| OPB45425 | T4742_S00010.336 | unknown protein |
| OPB45435 | T4742_S00010.346 | unknown protein |
| OPB45443 | T4742_S00010.354 | unknown protein |
| OPB45444 | T4742_S00010.355 | unknown protein |
| OPB45446 | T4742_S00010.357 | unknown protein |
| OPB45447 | T4742_S00010.358 | unknown protein |
| OPB45450 | T4742_S00010.361 | unknown protein |
| OPB45451 | T4742_S00010.362 | unknown protein |
| OPB45454 | T4742_S00010.365 | unknown protein |
| OPB45455 | T4742_S00010.366 | unknown protein |
| OPB45456 | T4742_S00010.367 | unknown protein |
| OPB45464 | T4742_S00010.375 | unknown protein |
| OPB45485 | T4742_S00010.396 | unknown protein |
| OPB45487 | T4742_S00010.398 | unknown protein |
| OPB45489 | T4742_S00010.400 | unknown protein |
| OPB45494 | T4742_S00010.405 | Unknown protein |
| OPB45497 | T4742_S00010.408 | unknown protein |
| OPB45503 | T4742_S00010.414 | unknown protein |
| OPB45504 | T4742_S00010.415 | unknown protein |
| OPB45506 | T4742_S00010.417 | unknown protein |
| OPB45511 | T4742_S00010.422 | unknown protein |
| OPB45513 | T4742_S00010.424 | unknown protein |

| | | |
|---|---|---|
| OPB45514 | T4742_S00010.425 | unknown protein |
| OPB45532 | T4742_S00010.443 | unknown protein |
| OPB45534 | T4742_S00010.445 | unknown protein |
| OPB45540 | T4742_S00010.451 | unknown protein |
| OPB45544 | T4742_S00010.455 | unknown protein |
| OPB45545 | T4742_S00010.456 | unknown protein |
| OPB45546 | T4742_S00010.457 | unknown protein |
| OPB45551 | T4742_S00010.462 | unknown protein |
| OPB45553 | T4742_S00010.464 | unknown protein |
| OPB45554 | T4742_S00010.465 | unknown protein |
| OPB45558 | T4742_S00010.469 | unknown protein |
| OPB45560 | T4742_S00010.471 | unknown protein |
| OPB45562 | T4742_S00010.473 | unknown protein |
| OPB45563 | T4742_S00010.474 | unknown protein |
| OPB45564 | T4742_S00010.475 | unknown protein |
| OPB45565 | T4742_S00010.476 | unknown protein |
| OPB45573 | T4742_S00010.484 | unknown protein |
| OPB45574 | T4742_S00010.485 | unknown protein |
| OPB45580 | T4742_S00010.491 | unknown protein |
| OPB45581 | T4742_S00010.492 | unknown protein |
| OPB45588 | T4742_S00010.499 | unknown protein |
| OPB45594 | T4742_S00010.505 | unknown protein |
| OPB45606 | T4742_S00010.517 | unknown protein |
| OPB45609 | T4742_S00010.520 | unknown protein |
| OPB45611 | T4742_S00010.522 | unknown protein |
| OPB45615 | T4742_S00010.526 | unknown protein |
| OPB45631 | T4742_S00010.542 | unknown protein |
| OPB45636 | T4742_S00010.547 | unknown protein |
| OPB45640 | T4742_S00010.551 | unknown protein |
| OPB45645 | T4742_S00010.556 | unknown protein |
| OPB45646 | T4742_S00010.557 | unknown protein |
| OPB45655 | T4742_S00010.566 | unknown protein |
| OPB45669 | T4742_S00014.3 | unknown protein |
| OPB45670 | T4742_S00014.4 | unknown protein |
| OPB45671 | T4742_S00014.5 | unknown protein |
| OPB45672 | T4742_S00014.6 | unknown protein |

| | | |
|---|---|---|
| OPB45675 | T4742_S00014.9 | unknown protein |
| OPB45676 | T4742_S00014.10 | unknown protein |
| OPB45679 | T4742_S00014.13 | unknown protein |
| OPB45680 | T4742_S00014.14 | unknown protein |
| OPB45681 | T4742_S00014.15 | unknown protein |
| OPB45687 | T4742_S00014.21 | unknown protein |
| OPB45690 | T4742_S00014.24 | unknown protein |
| OPB45691 | T4742_S00014.25 | unknown protein |
| OPB45693 | T4742_S00014.27 | unknown protein |
| OPB45694 | T4742_S00014.28 | unknown protein |
| OPB45695 | T4742_S00014.29 | unknown protein |
| OPB45697 | T4742_S00014.31 | unknown protein |
| OPB45707 | T4742_S00014.41 | unknown protein |
| OPB45709 | T4742_S00014.43 | unknown protein |
| OPB45714 | T4742_S00014.48 | unknown protein |
| OPB45716 | T4742_S00014.50 | unknown protein |
| OPB45717 | T4742_S00014.51 | unknown protein |
| OPB45725 | T4742_S00014.59 | unknown protein |
| OPB45726 | T4742_S00014.60 | unknown protein |
| OPB45727 | T4742_S00014.61 | unknown protein |
| OPB45730 | T4742_S00014.65 | unknown protein |
| OPB45732 | T4742_S00014.67 | unknown protein |
| OPB45734 | T4742_S00014.69 | unknown protein |
| OPB45735 | T4742_S00014.70 | unknown protein |
| OPB45736 | T4742_S00014.71 | unknown protein |
| OPB45747 | T4742_S00014.82 | unknown protein |
| OPB45753 | T4742_S00014.88 | unknown protein |
| OPB45757 | T4742_S00014.92 | unknown protein |
| OPB45759 | T4742_S00014.94 | unknown protein |
| OPB45761 | T4742_S00014.96 | unknown protein |
| OPB45765 | T4742_S00014.100 | unknown protein |
| OPB45766 | T4742_S00014.101 | unknown protein |
| OPB45769 | T4742_S00014.104 | unknown protein |
| OPB45783 | T4742_S00014.118 | unknown protein |
| OPB45784 | T4742_S00014.119 | unknown protein |
| OPB45785 | T4742_S00014.120 | unknown protein |

| | | |
|---|---|---|
| OPB45786 | T4742_S00014.121 | unknown protein |
| OPB45788 | T4742_S00014.123 | unknown protein |
| OPB45789 | T4742_S00014.124 | unknown protein |
| OPB45803 | T4742_S00014.138 | unknown protein |
| OPB45806 | T4742_S00014.141 | unknown protein |
| OPB45836 | T4742_S00014.171 | unknown protein |
| OPB45846 | T4742_S00014.181 | unknown protein |
| OPB45847 | T4742_S00014.182 | unknown protein |
| OPB45857 | T4742_S00014.192 | unknown protein |
| OPB45859 | T4742_S00014.194 | unknown protein |
| OPB45860 | T4742_S00014.195 | unknown protein |
| OPB45861 | T4742_S00014.196 | unknown protein |
| OPB45872 | T4742_S00014.207 | unknown protein |
| OPB45880 | T4742_S00014.215 | unknown protein |
| OPB45884 | T4742_S00014.219 | unknown protein |
| OPB45885 | T4742_S00014.220 | unknown protein |
| OPB45890 | T4742_S00014.225 | unknown protein |
| OPB45896 | T4742_S00014.232 | unknown protein |
| OPB45897 | T4742_S00014.233 | unknown protein |
| OPB45898 | T4742_S00014.234 | unknown protein |
| OPB45903 | T4742_S00014.239 | unknown protein |
| OPB45908 | T4742_S00014.244 | unknown protein |
| OPB45915 | T4742_S00014.251 | unknown protein |
| OPB45918 | T4742_S00014.254 | unknown protein |
| OPB45923 | T4742_S00014.260 | unknown protein |
| OPB45933 | T4742_S00014.270 | unknown protein |
| OPB45951 | T4742_S00008.17 | unknown protein |
| OPB45954 | T4742_S00008.20 | unknown protein |
| OPB45957 | T4742_S00008.23 | unknown protein |
| OPB45965 | T4742_S00008.31 | unknown protein |
| OPB45970 | T4742_S00008.36 | unknown protein |
| OPB45972 | T4742_S00008.38 | unknown protein |
| OPB45980 | T4742_S00008.46 | unknown protein |
| OPB45981 | T4742_S00008.47 | unknown protein |
| OPB45982 | T4742_S00008.48 | unknown protein |
| OPB45984 | T4742_S00008.50 | unknown protein |

| | | |
|---|---|---|
| OPB45988 | T4742_S00008.54 | unknown protein |
| OPB45992 | T4742_S00008.58 | unknown protein |
| OPB46002 | T4742_S00008.68 | unknown protein |
| OPB46008 | T4742_S00008.74 | unknown protein |
| OPB46011 | T4742_S00008.77 | unknown protein |
| OPB46014 | T4742_S00008.80 | unknown protein |
| OPB46016 | T4742_S00008.82 | unknown protein |
| OPB46034 | T4742_S00008.100 | unknown protein |
| OPB46040 | T4742_S00008.106 | unknown protein |
| OPB46043 | T4742_S00008.109 | unknown protein |
| OPB46046 | T4742_S00008.112 | unknown protein |
| OPB46052 | T4742_S00008.118 | unknown protein |
| OPB46054 | T4742_S00008.120 | unknown protein |
| OPB46056 | T4742_S00008.122 | unknown protein |
| OPB46059 | T4742_S00008.126 | unknown protein |
| OPB46061 | T4742_S00008.128 | unknown protein |
| OPB46064 | T4742_S00008.131 | unknown protein |
| OPB46065 | T4742_S00008.132 | unknown protein |
| OPB46075 | T4742_S00008.142 | unknown protein |
| OPB46076 | T4742_S00008.143 | unknown protein |
| OPB46077 | T4742_S00008.144 | unknown protein |
| OPB46080 | T4742_S00008.147 | unknown protein |
| OPB46081 | T4742_S00008.148 | unknown protein |
| OPB46090 | T4742_S00008.157 | unknown protein |
| OPB46095 | T4742_S00008.162 | unknown protein |
| OPB46105 | T4742_S00008.172 | unknown protein |
| OPB46106 | T4742_S00008.173 | unknown protein |
| OPB46107 | T4742_S00008.174 | unknown protein |
| OPB46111 | T4742_S00008.178 | unknown protein |
| OPB46116 | T4742_S00008.183 | unknown protein |
| OPB46128 | T4742_S00008.195 | unknown protein |
| OPB46138 | T4742_S00008.205 | unknown protein |
| OPB46139 | T4742_S00008.206 | unknown protein |
| OPB46144 | T4742_S00008.211 | unknown protein |
| OPB46147 | T4742_S00008.214 | unknown protein |
| OPB46150 | T4742_S00008.217 | unknown protein |

| | | |
|---|---|---|
| OPB46153 | T4742_S00008.220 | unknown protein |
| OPB46158 | T4742_S00008.225 | unknown protein |
| OPB46161 | T4742_S00008.228 | unknown protein |
| OPB46163 | T4742_S00008.230 | unknown protein |
| OPB46166 | T4742_S00008.233 | unknown protein |
| OPB46167 | T4742_S00008.234 | unknown protein |
| OPB46169 | T4742_S00008.236 | unknown protein |
| OPB46170 | T4742_S00008.237 | unknown protein |
| OPB46171 | T4742_S00008.238 | unknown protein |
| OPB46173 | T4742_S00008.240 | unknown protein |
| OPB46174 | T4742_S00008.241 | unknown protein |
| OPB46175 | T4742_S00008.242 | unknown protein |
| OPB46177 | T4742_S00008.244 | unknown protein |
| OPB46187 | T4742_S00008.254 | unknown protein |
| OPB46189 | T4742_S00008.256 | unknown protein |
| OPB46190 | T4742_S00008.257 | unknown protein |
| OPB46192 | T4742_S00008.259 | unknown protein |
| OPB46199 | T4742_S00008.266 | unknown protein |
| OPB46200 | T4742_S00008.267 | unknown protein |
| OPB46213 | T4742_S00008.280 | unknown protein |
| OPB46216 | T4742_S00008.283 | unknown protein |
| OPB46219 | T4742_S00008.286 | unknown protein |
| OPB46220 | T4742_S00008.287 | unknown protein |
| OPB46231 | T4742_S00008.298 | unknown protein |
| OPB46233 | T4742_S00008.300 | unknown protein |
| OPB46239 | T4742_S00008.306 | unknown protein |
| OPB46240 | T4742_S00008.307 | unknown protein |
| OPB46244 | T4742_S00008.311 | unknown protein |
| OPB46245 | T4742_S00008.312 | unknown protein |
| OPB46249 | T4742_S00008.316 | unknown protein |
| OPB46254 | T4742_S00008.321 | unknown protein |
| OPB46256 | T4742_S00008.323 | unknown protein |
| OPB46259 | T4742_S00008.326 | unknown protein |
| OPB46260 | T4742_S00008.327 | unknown protein |
| OPB46262 | T4742_S00008.329 | unknown protein |
| OPB46267 | T4742_S00008.334 | unknown protein |

| | | |
|---|---|---|
| OPB46270 | T4742_S00008.337 | unknown protein |
| OPB46271 | T4742_S00008.338 | unknown protein |
| OPB46274 | T4742_S00008.341 | unknown protein |
| OPB46275 | T4742_S00008.342 | unknown protein |
| OPB46278 | T4742_S00008.345 | unknown protein |
| OPB46279 | T4742_S00008.346 | unknown protein |
| OPB46280 | T4742_S00008.347 | unknown protein |
| OPB46288 | T4742_S00008.355 | unknown protein |
| OPB46289 | T4742_S00008.356 | unknown protein |
| OPB46295 | T4742_S00008.362 | unknown protein |
| OPB46297 | T4742_S00008.364 | unknown protein |
| OPB46298 | T4742_S00008.365 | unknown protein |
| OPB46306 | T4742_S00008.373 | unknown protein |
| OPB46316 | T4742_S00008.383 | unknown protein |
| OPB46317 | T4742_S00008.384 | unknown protein |
| OPB46318 | T4742_S00008.385 | unknown protein |
| OPB46319 | T4742_S00008.386 | unknown protein |
| OPB46323 | T4742_S00008.390 | unknown protein |
| OPB46327 | T4742_S00008.394 | unknown protein |
| OPB46334 | T4742_S00008.401 | unknown protein |
| OPB46336 | T4742_S00008.403 | unknown protein |
| OPB46339 | T4742_S00008.406 | unknown protein |
| OPB46341 | T4742_S00008.408 | unknown protein |
| OPB46346 | T4742_S00008.413 | unknown protein |
| OPB46347 | T4742_S00008.414 | unknown protein |
| OPB46348 | T4742_S00008.415 | unknown protein |
| OPB46352 | T4742_S00008.419 | unknown protein |
| OPB46354 | T4742_S00008.421 | unknown protein |
| OPB46356 | T4742_S00008.423 | unknown protein |
| OPB46358 | T4742_S00008.425 | unknown protein |
| OPB46375 | T4742_S00008.442 | unknown protein |
| OPB46377 | T4742_S00008.444 | unknown protein |
| OPB46380 | T4742_S00008.447 | unknown protein |
| OPB46382 | T4742_S00008.449 | unknown protein |
| OPB46388 | T4742_S00008.455 | unknown protein |
| OPB46392 | T4742_S00008.459 | unknown protein |

| | | |
|---|---|---|
| OPB46393 | T4742_S00008.460 | unknown protein |
| OPB46397 | T4742_S00008.464 | unknown protein |
| OPB46398 | T4742_S00008.465 | unknown protein |
| OPB46399 | T4742_S00008.466 | unknown protein |
| OPB46400 | T4742_S00008.467 | unknown protein |
| OPB46401 | T4742_S00008.468 | unknown protein |
| OPB46402 | T4742_S00008.469 | unknown protein |
| OPB46404 | T4742_S00008.471 | unknown protein |
| OPB46406 | T4742_S00008.473 | unknown protein |
| OPB46413 | T4742_S00008.480 | unknown protein |
| OPB46424 | T4742_S00008.491 | unknown protein |
| OPB46430 | T4742_S00008.497 | unknown protein |
| OPB46432 | T4742_S00008.499 | unknown protein |
| OPB46436 | T4742_S00008.503 | unknown protein |
| OPB46438 | T4742_S00008.505 | unknown protein |
| OPB46445 | T4742_S00008.512 | unknown protein |
| OPB46446 | T4742_S00008.513 | unknown protein |
| OPB46449 | T4742_S00008.516 | unknown protein |
| OPB46450 | T4742_S00008.517 | unknown protein |
| OPB46451 | T4742_S00008.518 | unknown protein |
| OPB46454 | T4742_S00008.521 | unknown protein |
| OPB46455 | T4742_S00008.522 | unknown protein |
| OPB46456 | T4742_S00008.523 | unknown protein |
| OPB46458 | T4742_S00008.525 | unknown protein |
| OPB46461 | T4742_S00008.528 | unknown protein |
| OPB46462 | T4742_S00008.529 | unknown protein |
| OPB46467 | T4742_S00008.534 | unknown protein |
| OPB46468 | T4742_S00008.535 | unknown protein |
| OPB46473 | T4742_S00008.540 | unknown protein |
| OPB46476 | T4742_S00008.543 | unknown protein |
| OPB46481 | T4742_S00008.548 | unknown protein |
| OPB46483 | T4742_S00008.550 | unknown protein |
| OPB46496 | T4742_S00008.563 | unknown protein |
| OPB46504 | T4742_S00008.571 | unknown protein |
| OPB46508 | T4742_S00008.575 | unknown protein |
| OPB46516 | T4742_S00008.583 | unknown protein |

| | | |
|---|---|---|
| OPB46517 | T4742_S00008.584 | unknown protein |
| OPB46519 | T4742_S00008.586 | unknown protein |
| OPB46529 | T4742_S00008.596 | unknown protein |
| OPB46530 | T4742_S00008.597 | unknown protein |
| OPB46564 | T4742_S00009.4 | unknown protein |
| OPB46571 | T4742_S00009.11 | unknown protein |
| OPB46573 | T4742_S00009.13 | unknown protein |
| OPB46574 | T4742_S00009.14 | unknown protein |
| OPB46579 | T4742_S00009.19 | unknown protein |
| OPB46593 | T4742_S00009.33 | unknown protein |
| OPB46603 | T4742_S00009.45 | unknown protein |
| OPB46605 | T4742_S00009.47 | unknown protein |
| OPB46607 | T4742_S00009.49 | unknown protein |
| OPB46610 | T4742_S00009.52 | unknown protein |
| OPB46611 | T4742_S00009.53 | unknown protein |
| OPB46615 | T4742_S00009.57 | unknown protein |
| OPB46616 | T4742_S00009.58 | unknown protein |
| OPB46617 | T4742_S00009.59 | unknown protein |
| OPB46623 | T4742_S00009.65 | unknown protein |
| OPB46624 | T4742_S00009.66 | unknown protein |
| OPB46628 | T4742_S00009.70 | unknown protein |
| OPB46631 | T4742_S00009.73 | unknown protein |
| OPB46632 | T4742_S00009.74 | unknown protein |
| OPB46633 | T4742_S00009.75 | Unknown protein |
| OPB46641 | T4742_S00009.83 | unknown protein |
| OPB46642 | T4742_S00009.84 | unknown protein |
| OPB46644 | T4742_S00009.86 | unknown protein |
| OPB46646 | T4742_S00009.88 | unknown protein |
| OPB46647 | T4742_S00009.89 | unknown protein |
| OPB46651 | T4742_S00009.93 | unknown protein |
| OPB46652 | T4742_S00009.94 | unknown protein |
| OPB46653 | T4742_S00009.95 | unknown protein |
| OPB46657 | T4742_S00009.99 | unknown protein |
| OPB46659 | T4742_S00009.101 | unknown protein |
| OPB46664 | T4742_S00009.106 | unknown protein |
| OPB46671 | T4742_S00009.113 | unknown protein |

| | | |
|---|---|---|
| OPB46672 | T4742_S00009.114 | unknown protein |
| OPB46673 | T4742_S00009.115 | unknown protein |
| OPB46679 | T4742_S00009.121 | unknown protein |
| OPB46682 | T4742_S00009.124 | unknown protein |
| OPB46684 | T4742_S00009.126 | unknown protein |
| OPB46688 | T4742_S00009.130 | unknown protein |
| OPB46692 | T4742_S00009.134 | unknown protein |
| OPB46695 | T4742_S00009.137 | unknown protein |
| OPB46696 | T4742_S00009.138 | unknown protein |
| OPB46697 | T4742_S00009.139 | unknown protein |
| OPB46698 | T4742_S00009.140 | unknown protein |
| OPB46701 | T4742_S00009.143 | unknown protein |
| OPB46703 | T4742_S00009.145 | unknown protein |
| OPB46704 | T4742_S00009.146 | unknown protein |
| OPB46706 | T4742_S00009.148 | unknown protein |
| OPB46709 | T4742_S00009.151 | unknown protein |
| OPB46712 | T4742_S00009.154 | unknown protein |
| OPB46717 | T4742_S00009.159 | unknown protein |
| OPB46720 | T4742_S00009.162 | unknown protein |
| OPB46724 | T4742_S00009.166 | unknown protein |
| OPB46725 | T4742_S00009.167 | unknown protein |
| OPB46726 | T4742_S00009.168 | unknown protein |
| OPB46727 | T4742_S00009.169 | unknown protein |
| OPB46733 | T4742_S00009.175 | unknown protein |
| OPB46735 | T4742_S00009.177 | unknown protein |
| OPB46742 | T4742_S00009.184 | unknown protein |
| OPB46743 | T4742_S00009.185 | unknown protein |
| OPB46744 | T4742_S00009.186 | unknown protein |
| OPB46746 | T4742_S00009.188 | unknown protein |
| OPB46747 | T4742_S00009.189 | unknown protein |
| OPB46751 | T4742_S00009.193 | unknown protein |
| OPB46753 | T4742_S00009.195 | Unknown protein |
| OPB46756 | T4742_S00009.198 | unknown protein |
| OPB46762 | T4742_S00009.204 | unknown protein |
| OPB46763 | T4742_S00009.205 | unknown protein |
| OPB46764 | T4742_S00009.206 | unknown protein |

| | | |
|---|---|---|
| OPB46766 | T4742_S00009.208 | unknown protein |
| OPB46768 | T4742_S00009.210 | unknown protein |
| OPB46770 | T4742_S00009.212 | unknown protein |
| OPB46771 | T4742_S00009.213 | unknown protein |
| OPB46772 | T4742_S00009.214 | unknown protein |
| OPB46777 | T4742_S00009.219 | unknown protein |
| OPB46778 | T4742_S00009.220 | unknown protein |
| OPB46782 | T4742_S00009.224 | unknown protein |
| OPB46786 | T4742_S00009.228 | unknown protein |
| OPB46787 | T4742_S00009.229 | unknown protein |
| OPB46790 | T4742_S00009.232 | unknown protein |
| OPB46792 | T4742_S00009.234 | unknown protein |
| OPB46793 | T4742_S00009.235 | unknown protein |
| OPB46794 | T4742_S00009.236 | unknown protein |
| OPB46796 | T4742_S00009.238 | unknown protein |
| OPB46798 | T4742_S00009.240 | unknown protein |
| OPB46801 | T4742_S00009.243 | unknown protein |
| OPB46811 | T4742_S00009.253 | unknown protein |
| OPB46812 | T4742_S00009.254 | unknown protein |
| OPB46814 | T4742_S00009.256 | unknown protein |
| OPB46816 | T4742_S00009.258 | Unknown protein |
| OPB46820 | T4742_S00009.262 | unknown protein |
| OPB46826 | T4742_S00009.268 | unknown protein |
| OPB46829 | T4742_S00009.271 | unknown protein |
| OPB46830 | T4742_S00009.272 | unknown protein |
| OPB46833 | T4742_S00009.275 | unknown protein |
| OPB46835 | T4742_S00009.277 | unknown protein |
| OPB46837 | T4742_S00009.279 | unknown protein |
| OPB46838 | T4742_S00009.280 | unknown protein |
| OPB46840 | T4742_S00009.282 | unknown protein |
| OPB46843 | T4742_S00009.285 | unknown protein |
| OPB46845 | T4742_S00009.287 | unknown protein |
| OPB46848 | T4742_S00009.290 | unknown protein |
| OPB46854 | T4742_S00009.296 | unknown protein |
| OPB46855 | T4742_S00009.297 | unknown protein |
| OPB46857 | T4742_S00009.299 | unknown protein |

| | | |
|---|---|---|
| OPB46858 | T4742_S00009.300 | unknown protein |
| OPB46859 | T4742_S00009.301 | unknown protein |
| OPB46860 | T4742_S00009.302 | unknown protein |
| OPB46861 | T4742_S00009.303 | unknown protein |
| OPB46862 | T4742_S00009.304 | unknown protein |
| OPB46867 | T4742_S00009.309 | unknown protein |
| OPB46870 | T4742_S00009.312 | unknown protein |
| OPB46872 | T4742_S00009.314 | unknown protein |
| OPB46880 | T4742_S00009.322 | unknown protein |
| OPB46881 | T4742_S00009.323 | unknown protein |
| OPB46884 | T4742_S00009.326 | unknown protein |
| OPB46885 | T4742_S00009.327 | unknown protein |
| OPB46891 | T4742_S00009.333 | unknown protein |
| OPB46892 | T4742_S00009.334 | unknown protein |
| OPB46894 | T4742_S00009.336 | unknown protein |
| OPB46900 | T4742_S00009.342 | unknown protein |
| OPB46901 | T4742_S00009.343 | unknown protein |
| OPB46907 | T4742_S00009.349 | unknown protein |
| OPB46909 | T4742_S00009.351 | unknown protein |
| OPB46911 | T4742_S00009.353 | unknown protein |
| OPB46917 | T4742_S00009.359 | unknown protein |
| OPB46918 | T4742_S00009.360 | unknown protein |
| OPB46919 | T4742_S00009.361 | unknown protein |
| OPB46920 | T4742_S00009.362 | unknown protein |
| OPB46921 | T4742_S00009.363 | unknown protein |
| OPB46922 | T4742_S00009.364 | unknown protein |
| OPB46927 | T4742_S00009.369 | unknown protein |
| OPB46928 | T4742_S00009.370 | unknown protein |
| OPB46930 | T4742_S00009.372 | unknown protein |
| OPB46936 | T4742_S00009.378 | unknown protein |
| OPB46941 | T4742_S00009.383 | unknown protein |
| OPB46944 | T4742_S00009.386 | unknown protein |
| OPB46950 | T4742_S00009.392 | unknown protein |
| OPB46956 | T4742_S00009.398 | unknown protein |
| OPB46957 | T4742_S00009.399 | unknown protein |
| OPB46958 | T4742_S00009.400 | unknown protein |

| | | |
|---|---|---|
| OPB46960 | T4742_S00009.402 | unknown protein |
| OPB46964 | T4742_S00009.406 | Unknown protein |
| OPB46966 | T4742_S00009.408 | unknown protein |
| OPB46968 | T4742_S00009.410 | unknown protein |
| OPB46974 | T4742_S00009.416 | unknown protein |
| OPB46975 | T4742_S00009.417 | unknown protein |
| OPB46976 | T4742_S00009.418 | unknown protein |
| OPB46977 | T4742_S00009.419 | unknown protein |
| OPB46982 | T4742_S00009.424 | unknown protein |
| OPB46993 | T4742_S00009.435 | unknown protein |
| OPB46995 | T4742_S00009.437 | unknown protein |
| OPB46997 | T4742_S00009.439 | unknown protein |
| OPB47002 | T4742_S00009.444 | unknown protein |
| OPB47008 | T4742_S00009.450 | unknown protein |
| OPB47011 | T4742_S00009.453 | unknown protein |
| OPB47013 | T4742_S00009.455 | unknown protein |
| OPB47015 | T4742_S00009.457 | unknown protein |
| OPB47016 | T4742_S00009.458 | unknown protein |
| OPB47017 | T4742_S00009.459 | unknown protein |
| OPB47020 | T4742_S00009.462 | unknown protein |
| OPB47025 | T4742_S00009.467 | unknown protein |
| OPB47036 | T4742_S00009.478 | unknown protein |
| OPB47039 | T4742_S00009.481 | unknown protein |
| OPB47043 | T4742_S00009.485 | unknown protein |
| OPB47055 | T4742_S00009.497 | unknown protein |
| OPB47059 | T4742_S00009.501 | unknown protein |
| OPB47063 | T4742_S00009.505 | unknown protein |
| OPB47067 | T4742_S00009.509 | unknown protein |
| OPB47069 | T4742_S00009.511 | unknown protein |
| OPB47070 | T4742_S00009.512 | unknown protein |
| OPB47072 | T4742_S00009.514 | unknown protein |
| OPB47074 | T4742_S00009.516 | unknown protein |
| OPB47077 | T4742_S00009.519 | unknown protein |
| OPB47082 | T4742_S00009.524 | unknown protein |
| OPB47087 | T4742_S00009.529 | unknown protein |
| OPB47088 | T4742_S00009.530 | unknown protein |

| | | |
|---|---|---|
| OPB47095 | T4742_S00009.537 | unknown protein |
| OPB47104 | T4742_S00009.546 | unknown protein |
| OPB47106 | T4742_S00009.548 | unknown protein |
| OPB47110 | T4742_S00009.552 | unknown protein |
| OPB47113 | T4742_S00009.555 | unknown protein |
| OPB47117 | T4742_S00009.559 | unknown protein |
| OPB47118 | T4742_S00009.560 | unknown protein |
| OPB47119 | T4742_S00009.561 | unknown protein |
| OPB47120 | T4742_S00009.562 | unknown protein |
| OPB47121 | T4742_S00009.563 | unknown protein |
| OPB47124 | T4742_S00009.566 | unknown protein |
| OPB47126 | T4742_S00009.568 | unknown protein |
| OPB47129 | T4742_S00009.571 | unknown protein |
| OPB47130 | T4742_S00009.572 | unknown protein |
| OPB47131 | T4742_S00009.573 | unknown protein |
| OPB47132 | T4742_S00009.574 | unknown protein |
| OPB47133 | T4742_S00009.575 | unknown protein |
| OPB47140 | T4742_S00009.582 | unknown protein |
| OPB47142 | T4742_S00009.584 | unknown protein |
| OPB47144 | T4742_S00009.586 | unknown protein |
| OPB47146 | T4742_S00009.588 | unknown protein |
| OPB47147 | T4742_S00009.589 | unknown protein |
| OPB47152 | T4742_S00009.594 | unknown protein |
| OPB47159 | T4742_S00009.601 | unknown protein |
| OPB47162 | T4742_S00009.604 | unknown protein |
| OPB47163 | T4742_S00009.605 | unknown protein |
| OPB47164 | T4742_S00009.606 | unknown protein |
| OPB47169 | T4742_S00009.611 | unknown protein |
| OPB47170 | T4742_S00009.612 | unknown protein |
| OPB47172 | T4742_S00009.614 | unknown protein |
| OPB47174 | T4742_S00009.616 | unknown protein |
| OPB36571 | T4742_S00007.288 | unknown protein |
| OPB37133 | T4742_S00005.160 | unknown protein |
| OPB38478 | T4742_S00002.526 | unknown protein |
| OPB40472 | T4742_S00001.551 | unknown protein |
| OPB43554 | T4742_S00016.74 | unknown protein |

| | | |
|---|---|---|
| OPB46036 | T4742_S00008.102 | unknown protein |
| OPB40201 | T4742_S00001.280 | unknown protein (tyrosin phosphatase domain) |
| OPB37880 | T4742_S00017.147 | unknown protein containing a putative BTB/POZ domain |
| OPB39139 | T4742_S00006.199 | unknown protein containing a putative BTB/POZ domain |
| OPB36509 | T4742_S00007.226 | unknown protein Duf1479 |
| OPB40089 | T4742_S00001.168 | unknown protein DUF1531 |
| OPB43344 | T4742_S00019.77 | unknown protein GPR1/FUN34/yaaH-like |
| OPB40464 | T4742_S00001.543 | unknown protein in Sordariomycetes |
| OPB45473 | T4742_S00010.384 | unknown protein NTF2-like domain |
| OPB42303 | T4742_S00004.405 | unknown protein of HSP60 family |
| OPB37111 | T4742_S00005.138 | unknown protein of ORM1 family |
| OPB40976 | T4742_S00001.1057 | unknown protein required for sexual development |
| OPB44429 | T4742_S00003.701 | unknown protein UPF0183 |
| OPB36650 | T4742_S00007.368 | unknown protein VanZ like family |
| OPB39807 | T4742_S00015.151 | unknown protein with  DEAD/DEAH box helicase domain |
| OPB43276 | T4742_S00019.9 | unknown protein with a possible Interpro domain corresponding to ribosomal protein S12/S23. Possible homologue of yeast mitochondrial ribosomal protein YNR036C. |
| OPB38529 | T4742_S00002.578 | Unknown protein with a putative zinc finger binding motif |
| OPB36853 | T4742_S00007.571 | unknown protein with BolA domain |
| OPB37830 | T4742_S00017.97 | unknown protein with BolA domain |
| OPB43213 | T4742_S00022.16 | unknown protein with brix domain |
| OPB43005 | T4742_S00012.242 | unknown protein with Ca2+-dependent membrane-targeting module |
| OPB38375 | T4742_S00002.423 | unknown protein with caleosin domain |
| OPB43613 | T4742_S00016.133 | unknown protein with CCHC finger |
| OPB36311 | T4742_S00007.28 | unknown protein with chromo domain |
| OPB37884 | T4742_S00017.151 | unknown protein with chromo-domain |
| OPB37032 | T4742_S00005.59 | unknown protein with CORD and CS domain |
| OPB45210 | T4742_S00010.118 | unknown protein with D111/G-patch [Cordyceps brongniartii RCEF 3172] |
| OPB45377 | T4742_S00010.288 | unknown protein with D111/G-patch [Cordyceps brongniartii RCEF 3172] |
| OPB42478 | T4742_S00004.580 | unknown protein with dDENN domain |
| OPB42189 | T4742_S00004.291 | unknown protein with DENN domain |
| OPB43046 | T4742_S00012.283 | unknown protein with DnaJ domain, HSP40 |
| OPB44230 | T4742_S00003.501 | unknown protein with DnaJ domain, HSP40 |
| OPB42047 | T4742_S00004.149 | Unknown protein with DNL zinc finger domain. |
| OPB39895 | T4742_S00015.239 | unknown protein with Duf1237 domain |
| OPB36850 | T4742_S00007.568 | unknown protein with DUF1767 domain |
| OPB38050 | T4742_S00002.97 | unknown protein with DUF1868 domain |

| | | |
|---|---|---|
| OPB43875 | T4742_S00003.146 | unknown protein with Duf218 domain |
| OPB44642 | T4742_S00003.914 | unknown protein with fasciclin domain |
| OPB43940 | T4742_S00003.211 | Unknown protein with FYVE/PHD zinc finger domain. |
| OPB36958 | T4742_S00007.676 | unknown protein with HIT domain |
| OPB38245 | T4742_S00002.292 | unknown protein with HIT domain |
| OPB44271 | T4742_S00003.542 | unknown protein with Kelch domain |
| OPB41529 | T4742_S00011.372 | unknown protein with kelch repeats |
| OPB42974 | T4742_S00012.211 | unknown protein with Kelch repeats |
| OPB44274 | T4742_S00003.545 | unknown protein with Kelch repeats |
| OPB43987 | T4742_S00003.258 | unknown protein with Nif domain |
| OPB37789 | T4742_S00017.56 | unknown protein with NUDIX domain (hydrolase) |
| OPB41267 | T4742_S00011.108 | unknown protein with NUDIX domain (hydrolase) |
| OPB37402 | T4742_S00005.429 | unknown protein with patatin domain |
| OPB46342 | T4742_S00008.409 | unknown protein with pfs domain |
| OPB36922 | T4742_S00007.640 | unknown protein with PH domain (putative phosphatidylinositol 4,5-bisphosphate protein) |
| OPB38423 | T4742_S00002.471 | unknown protein with pleckstrin like domain |
| OPB37009 | T4742_S00005.36 | unknown protein with PWWP domain |
| OPB43015 | T4742_S00012.252 | unknown protein with PX domain |
| OPB36818 | T4742_S00007.536 | unknown protein with RCC1 domain |
| OPB39778 | T4742_S00015.122 | Unknown protein with reductase domain |
| OPB41096 | T4742_S00020.120 | unknown protein with RING finger SH3 domain |
| OPB36388 | T4742_S00007.105 | Unknown protein with RNA binding domains |
| OPB36440 | T4742_S00007.157 | unknown protein with RTA1 domain, 7 TM responds to xenobiotic stimuli |
| OPB40483 | T4742_S00001.562 | unknown protein with RTA1 domain, 7 TM responds to xenobiotic stimuli |
| OPB40777 | T4742_S00001.858 | unknown protein with RTA1 domain, 7 TM responds to xenobiotic stimuli |
| OPB41009 | T4742_S00020.33 | unknown protein with RTA1 domain, 7 TM responds to xenobiotic stimuli |
| OPB41168 | T4742_S00011.8 | unknown protein with RTA1 domain, 7 TM responds to xenobiotic stimuli |
| OPB42718 | T4742_S00004.824 | unknown protein with RTA1 domain, 7 TM responds to xenobiotic stimuli |
| OPB43066 | T4742_S00012.303 | unknown protein with RTA1 domain, 7 TM responds to xenobiotic stimuli |
| OPB43296 | T4742_S00019.29 | unknown protein with RTA1 domain, 7 TM responds to xenobiotic stimuli |
| OPB39461 | T4742_S00006.521 | unknown protein with SacI domain |
| OPB39673 | T4742_S00015.17 | unknown protein with SDA1 domain |
| OPB43600 | T4742_S00016.120 | unknown protein with SET domain |
| OPB38237 | T4742_S00002.284 | unknown protein with SH3 domain |
| OPB44377 | T4742_S00003.648 | unknown protein with SH3 domain |
| OPB36116 | T4742_S00021.33 | Unknown protein with ThiJ/PfpI domain |

| | | |
|---|---|---|
| OPB39100 | T4742_S00006.160 | unknown protein with ThiJ/PfpI domain |
| OPB41201 | T4742_S00011.41 | unknown protein with thij/pfpi domain |
| OPB43266 | T4742_S00022.70 | Unknown protein with ThiJ/PfpI domain |
| OPB43368 | T4742_S00019.101 | Unknown protein with ThiJ/PfpI domain |
| OPB43746 | T4742_S00003.17 | Unknown protein with ThiJ/PfpI domain |
| OPB44586 | T4742_S00003.858 | unknown protein with ThiJ/PfpI domain |
| OPB40607 | T4742_S00001.687 | unknown protein with TIM barrel |
| OPB45149 | T4742_S00010.56 | unknown protein with Tim10/DDP family zinc finger |
| OPB39178 | T4742_S00006.238 | unknown protein with TLC domain |
| OPB39179 | T4742_S00006.239 | unknown protein with TLC domain |
| OPB44258 | T4742_S00003.529 | unknown protein with TonB box |
| OPB40489 | T4742_S00001.568 | unknown protein with TPR domain |
| OPB36849 | T4742_S00007.567 | unknown protein with TPR repeat |
| OPB45315 | T4742_S00010.226 | unknown protein with TPR repeats |
| OPB45947 | T4742_S00008.13 | Unknown protein with TPR structural motif. |
| OPB41675 | T4742_S00011.518 | unknown protein with WD domain |
| OPB36242 | T4742_S00021.160 | unknown protein with WD repeats |
| OPB36260 | T4742_S00021.178 | unknown protein with WD repeats |
| OPB37060 | T4742_S00005.87 | unknown protein with WD repeats |
| OPB38126 | T4742_S00002.173 | unknown protein with WD repeats |
| OPB38858 | T4742_S00002.907 | unknown protein with WD repeats |
| OPB38914 | T4742_S00002.963 | unknown protein with WD repeats |
| OPB39317 | T4742_S00006.377 | unknown protein with WD repeats |
| OPB39927 | T4742_S00001.6 | unknown protein with WD repeats |
| OPB40009 | T4742_S00001.88 | unknown protein with WD repeats |
| OPB40070 | T4742_S00001.149 | unknown protein with WD repeats |
| OPB40516 | T4742_S00001.595 | unknown protein with WD repeats |
| OPB40908 | T4742_S00001.989 | unknown protein with WD repeats |
| OPB41608 | T4742_S00011.451 | unknown protein with WD repeats |
| OPB42657 | T4742_S00004.762 | unknown protein with WD repeats |
| OPB43502 | T4742_S00016.22 | unknown protein with WD repeats |
| OPB43573 | T4742_S00016.93 | unknown protein with WD repeats |
| OPB45306 | T4742_S00010.217 | unknown protein with WD repeats |
| OPB41787 | T4742_S00018.110 | unknown protein with WD40 repeat |
| OPB38125 | T4742_S00002.172 | unknown protein with WD40 repeats |
| OPB39810 | T4742_S00015.154 | Unknown protein with WD40 repeats |

| | | |
|---|---|---|
| OPB38315 | T4742_S00002.362 | Unknown protein with WD-40 repeats |
| OPB42467 | T4742_S00004.569 | Unknown protein with WD40 repeats and a LisH domain which mediation protein dimerisation and tetramerisation |
| OPB39381 | T4742_S00006.441 | unknown protein with WSC domain |
| OPB40060 | T4742_S00001.139 | unknown protein with WSC domains |
| OPB37983 | T4742_S00002.30 | unknown protein with YIP1 domain |
| OPB38971 | T4742_S00006.30 | Unknown protein with Zn-finger |
| OPB38578 | T4742_S00002.627 | unknown protein, RRM domain |
| OPB45231 | T4742_S00010.140 | unknown protein, RRM domain |
| OPB43921 | T4742_S00003.192 | unknown protein, transmembrane spanning |
| OPB36757 | T4742_S00007.475 | unknown protein, WD40 repeat superfamily |
| OPB42271 | T4742_S00004.373 | unknownprotein with TBC domain |
| OPB45718 | T4742_S00014.52 | unknownprotein with TBC domain |
| OPB39331 | T4742_S00006.391 | unknwon zinc finger protein |
| OPB40943 | T4742_S00001.1024 | uracil phosphoribosyl transferase |
| OPB45082 | T4742_S00013.387 | uracil phosphoribosyltransferase |
| OPB44351 | T4742_S00003.622 | uracil-DNA glycosylase |
| OPB41793 | T4742_S00018.116 | urea amidolyase |
| OPB46421 | T4742_S00008.488 | urea amidolyase |
| OPB41183 | T4742_S00011.23 | urea carboxylase |
| OPB37158 | T4742_S00005.185 | Urea transporter |
| OPB41573 | T4742_S00011.416 | urea transporter |
| OPB43405 | T4742_S00019.138 | Urea transporter |
| OPB44446 | T4742_S00003.718 | urease |
| OPB40975 | T4742_S00001.1056 | ureate oxidase |
| OPB40035 | T4742_S00001.114 | UreD urease accessory protein |
| OPB37341 | T4742_S00005.368 | UreF urease accessory protein |
| OPB37136 | T4742_S00005.163 | ureidoglycolate hydrolase |
| OPB37137 | T4742_S00005.164 | ureidoglycolate hydrolase |
| OPB39886 | T4742_S00015.230 | uridine kinase |
| OPB43001 | T4742_S00012.238 | uridylate kinase |
| OPB41743 | T4742_S00018.66 | uridylate kinase [Trichoderma harzianum] |
| OPB39634 | T4742_S00006.694 | uroporphyrinogen decarboxylase |
| OPB36934 | T4742_S00007.652 | uroporphyrinogen synthase |
| OPB37602 | T4742_S00005.629 | uroporphyrinogen synthase |
| OPB39356 | T4742_S00006.416 | UTP10, encoding a component of the SSU processome containing the U3 snoRNA |
| OPB44153 | T4742_S00003.424 | UTP11, encoding a component of the SSU processome |

| | | |
|---|---|---|
| OPB40188 | T4742_S00001.267 | UTP13, encoding a component of the SSU processome |
| OPB36314 | T4742_S00007.31 | UTP14, encoding a component of the SSU processome |
| OPB37451 | T4742_S00005.478 | UTP15, encoding a component of the SSU processome |
| OPB42159 | T4742_S00004.261 | Utp18p, a component of the SSU processome |
| OPB42048 | T4742_S00004.150 | UTP20. snoRNA-binding protein and member of the SSU processome. |
| OPB36907 | T4742_S00007.625 | UTP22. U3 snoRNP protein involved in maturation of pre-18S rRNA and SSU processome protein. |
| OPB44236 | T4742_S00003.507 | UTP4, a component of the SSU processome |
| OPB46272 | T4742_S00008.339 | UTP5, encoding a component of the SSU processome |
| OPB36711 | T4742_S00007.429 | UTP6, a Component of the SSU processome |
| OPB42106 | T4742_S00004.208 | UTP-glucose-1-phosphate uridylyltransferase |
| OPB42069 | T4742_S00004.171 | UvrB, C-terminal UvrC-binding |
| OPB40400 | T4742_S00001.479 | UVSD; probable regulator of DNA damage response |
| OPB36717 | T4742_S00007.435 | Vacuolar assembly protein Vps41 |
| OPB45668 | T4742_S00014.2 | Vacuolar assembly/sorting protein VPS9 |
| OPB37073 | T4742_S00005.100 | Vacuolar ATP synthase 16 kDa proteolipid subunit |
| OPB38012 | T4742_S00002.59 | Vacuolar ATP synthase 20 kDa proteolipid subunit |
| OPB39871 | T4742_S00015.215 | vacuolar ATP synthase 98 kDa subunit |
| OPB37258 | T4742_S00005.285 | vacuolar ATP synthase catalytic subunit A |
| OPB39281 | T4742_S00006.341 | Vacuolar ATP synthase subunit B |
| OPB39919 | T4742_S00015.263 | vacuolar ATP synthase subunit D |
| OPB43123 | T4742_S00012.360 | vacuolar ATP synthase subunit d |
| OPB38086 | T4742_S00002.133 | vacuolar ATP synthase subunit F |
| OPB35932 | T4742_S00025.5 | vacuolar ATPase |
| OPB39442 | T4742_S00006.502 | vacuolar ATPase assembly integral membrane protein VMA21, putative |
| OPB41211 | T4742_S00011.51 | Vacuolar carboxypeptidase Cps1 |
| OPB43067 | T4742_S00012.304 | Vacuolar carboxypeptidase Cps1 |
| OPB43688 | T4742_S00016.208 | vacuolar endopolyphosphatase |
| OPB42963 | T4742_S00012.200 | vacuolar fusion protein MON1 |
| OPB36735 | T4742_S00007.453 | vacuolar import and degradation protein 27 |
| OPB40436 | T4742_S00001.515 | vacuolar import and degradation protein, vid24 |
| OPB37300 | T4742_S00005.327 | vacuolar protein sorting 55 |
| OPB44535 | T4742_S00003.807 | vacuolar protein sorting associated protein 62 |
| OPB43314 | T4742_S00019.47 | vacuolar protein sorting protein (Vps36), putative |
| OPB44902 | T4742_S00013.207 | vacuolar protein sorting protein Vps66, putative |
| OPB36198 | T4742_S00021.116 | vacuolar protein sorting-associated protein 62 [Metarhizium robertsii ARSEF 23] |
| OPB40126 | T4742_S00001.205 | Vacuolar protein sorting-associated protein 68 |

| | | |
|---|---|---|
| OPB39686 | T4742_S00015.30 | Vacuolar protein sorting-associated protein Vps11 |
| OPB46474 | T4742_S00008.541 | Vacuolar protein sorting-associated protein Vps13 |
| OPB46713 | T4742_S00009.155 | Vacuolar protein sorting-associated protein Vps16 |
| OPB41091 | T4742_S00020.115 | Vacuolar protein sorting-associated protein Vps2 |
| OPB43610 | T4742_S00016.130 | Vacuolar protein sorting-associated protein Vps24 |
| OPB36298 | T4742_S00007.15 | Vacuolar protein sorting-associated protein Vps27 |
| OPB37826 | T4742_S00017.93 | Vacuolar protein sorting-associated protein Vps28 |
| OPB38981 | T4742_S00006.40 | Vacuolar protein sorting-associated protein Vps33 |
| OPB36472 | T4742_S00007.189 | Vacuolar protein sorting-associated protein Vps4 [Trichoderma guizhouense] |
| OPB37433 | T4742_S00005.460 | Vacuolar protein sorting-associated protein Vps45 |
| OPB38790 | T4742_S00002.839 | Vacuolar protein sorting-associated protein Vps46 |
| OPB44296 | T4742_S00003.567 | Vacuolar protein sorting-associated protein Vps8 |
| OPB44297 | T4742_S00003.568 | Vacuolar protein sorting-associated protein Vps8 |
| OPB46435 | T4742_S00008.502 | Vacuolar protein sorting-associated protein Vsp60 |
| OPB46910 | T4742_S00009.352 | Vacuolar protein sorting-associated protein Vsp9 |
| OPB36530 | T4742_S00007.247 | vacuolar protein-sorting machinery class E protein HSE1 |
| OPB38044 | T4742_S00002.91 | vacuolar protein-sorting protein BRO1 |
| OPB42553 | T4742_S00004.656 | Vacuolar segregation protein Pep7 |
| OPB41483 | T4742_S00011.326 | Vacuolar sorting protein Pep3/Vps18 |
| OPB46729 | T4742_S00009.171 | Vacuolar sorting protein VPS1, dynamin |
| OPB37980 | T4742_S00002.27 | Vacuolar sorting protein Vps52 |
| OPB36900 | T4742_S00007.618 | Vacuolar sorting protein Vps74 |
| OPB38129 | T4742_S00002.176 | vacuolar sorting protein, vps17 |
| OPB45037 | T4742_S00013.342 | vacuolar sorting protein, vps3, and TGF beta receptor associated protein 1 |
| OPB35930 | T4742_S00025.3 | Vacuolar sorting protien/ubiquitin receptor Vps23 |
| OPB40238 | T4742_S00001.317 | Vacuolar transporter chaperone |
| OPB42144 | T4742_S00004.246 | Vacuolar transporter chaperone 4 (vtc4), putative |
| OPB40967 | T4742_S00001.1048 | vacuolar-sorting protein SNF8, putative |
| OPB36968 | T4742_S00007.686 | vacuole-associated enzyme activator complex component (Vac14) |
| OPB39005 | T4742_S00006.64 | valyl-tRNA synthetase, mitochondrial precursor. |
| OPB44972 | T4742_S00013.277 | valyl-tRNA synthetase, mitochondrial precursor. |
| OPB39592 | T4742_S00006.652 | V-ATPase subunit C |
| OPB39830 | T4742_S00015.174 | vegetative incompatibility protein HET-D2Y [Podospora anserina] |
| OPB43531 | T4742_S00016.51 | vegetative incompatibility protein HET-E-1 [Aspergillus lentulus] |
| OPB40350 | T4742_S00001.429 | Vegetative incompatibility protein HET-E-1 [Talaromyces marneffei PM1] |
| OPB39752 | T4742_S00015.96 | vel1, velvet protein |

| | | |
|---|---|---|
| OPB43501 | T4742_S00016.21 | velB |
| OPB36234 | T4742_S00021.152 | very-long-chain acyl-CoA dehydrogenase |
| OPB37749 | T4742_S00017.16 | Vesicle coat complex COPI, alpha subunit |
| OPB40438 | T4742_S00001.517 | Vesicle coat complex COPI, beta subunit |
| OPB45569 | T4742_S00010.480 | Vesicle coat complex COPI, beta` subunit |
| OPB42142 | T4742_S00004.244 | Vesicle coat complex COPI, epsilon subunit |
| OPB38625 | T4742_S00002.674 | Vesicle coat complex COPI, gamma subunit |
| OPB41936 | T4742_S00004.38 | Vesicle coat complex COPII, Sec13 |
| OPB36931 | T4742_S00007.649 | Vesicle coat complex COPII, Sec24 |
| OPB42410 | T4742_S00004.512 | Vesicle coat complex COPII, Sec31 |
| OPB36769 | T4742_S00007.487 | Vesicle coat complex COPII, Sfb3 |
| OPB39038 | T4742_S00006.97 | violaceus kinesin |
| OPB43536 | T4742_S00016.56 | violaceus kinesin |
| OPB41879 | T4742_S00018.202 | vitamin B6 transporter |
| OPB41499 | T4742_S00011.342 | voltage gated chloride channel |
| OPB43598 | T4742_S00016.118 | VPS24, Vacuolar protein sorting-associated protein |
| OPB40511 | T4742_S00001.590 | vps9-ankyrin repeat protein |
| OPB39746 | T4742_S00015.90 | v-SNARE Bet1, ER-Golgi complex |
| OPB40343 | T4742_S00001.422 | v-SNARE Bos1, ER-Golgi |
| OPB46821 | T4742_S00009.263 | v-SNARE Gos1, Golgi transport |
| OPB42151 | T4742_S00004.253 | v-SNARE Sec20 |
| OPB46951 | T4742_S00009.393 | v-Snare Sft1; intra-Golgi |
| OPB43595 | T4742_S00016.115 | v-SNARE Vti1; cis-Golgi membrane traffic; vacuolar sorting pathways |
| OPB38197 | T4742_S00002.244 | v-SNARE Ykt6; trafficking to and within Golgi, endocytic trafficking to vacuole, vacuolar fusion |
| OPB39334 | T4742_S00006.394 | Vsp9 domain protein |
| OPB47058 | T4742_S00009.500 | VTS1-SAM cotaining protein |
| OPB43307 | T4742_S00019.40 | wall-associated protein [Aspergillus udagawae] |
| OPB41572 | T4742_S00011.415 | WbbJ Acetyltransferase (isoleucine patch superfamily) |
| OPB39801 | T4742_S00015.145 | Winged helix repressor DNA-binding |
| OPB39922 | T4742_S00001.1 | Winged helix repressor DNA-binding |
| OPB39973 | T4742_S00001.52 | Winged helix repressor DNA-binding |
| OPB41933 | T4742_S00004.35 | Winged helix repressor DNA-binding |
| OPB43575 | T4742_S00016.95 | Winged helix repressor DNA-binding |
| OPB39698 | T4742_S00015.42 | xanthine dehydrogenase |
| OPB36840 | T4742_S00007.558 | xanthine phosphoribosyltransferase 1 |
| OPB38768 | T4742_S00002.817 | xanthine/uracil permease |

| | | |
|---|---|---|
| OPB44665 | T4742_S00003.937 | Xanthine/uracil permease family |
| OPB39920 | T4742_S00015.264 | XPA binding protein 2 (XAB2) involved in pre-mRNA splicing, transcription, and transcription-coupled DNA repair |
| OPB44233 | T4742_S00003.504 | Xrs2, Interacts with Tel1, enhances Mre11 nuclease activity |
| OPB44156 | T4742_S00003.427 | xylitol dehydrogenase XDH1 |
| OPB37877 | T4742_S00017.144 | xylose reductase |
| OPB45197 | T4742_S00010.105 | xylulokinase |
| OPB39974 | T4742_S00001.53 | Yip1 interacting protein Yop1 |
| OPB43710 | T4742_S00016.230 | YTH family RNA binding protein, |
| OPB41709 | T4742_S00018.32 | zinc binding alcohol dehydrogenase |
| OPB43464 | T4742_S00019.197 | zinc binding alcohol dehydrogenase |
| OPB36786 | T4742_S00007.504 | Zinc carboxypeptidase |
| OPB40820 | T4742_S00001.901 | zinc containing alcohol dehydrogenase superfamily |
| OPB43445 | T4742_S00019.178 | zinc containing alcohol dehydrogenase superfamily |
| OPB43470 | T4742_S00019.203 | zinc containing alcohol dehydrogenase superfamily |
| OPB36321 | T4742_S00007.38 | zinc dependent alcohol dehydrogenase |
| OPB37707 | T4742_S00005.735 | zinc dependent alcohol dehydrogenase |
| OPB38233 | T4742_S00002.280 | zinc dependent alcohol dehydrogenase |
| OPB38932 | T4742_S00002.981 | zinc dependent alcohol dehydrogenase |
| OPB43199 | T4742_S00022.2 | zinc dependent alcohol dehydrogenase |
| OPB43639 | T4742_S00016.159 | zinc dependent alcohol dehydrogenase |
| OPB45482 | T4742_S00010.393 | zinc dependent alcohol dehydrogenase |
| OPB36859 | T4742_S00007.577 | Zinc finger domain protein, MYND-type |
| OPB35991 | T4742_S00023.26 | Zinc finger, CCCH-type, partial [Metarhizium anisopliae ARSEF 549] |
| OPB45174 | T4742_S00010.81 | Zinc finger, RING-type |
| OPB45224 | T4742_S00010.133 | Zinc finger, RING-type |
| OPB46612 | T4742_S00009.54 | Zinc finger, RING-type |
| OPB40258 | T4742_S00001.337 | Zinc finger, U1-C type |
| OPB39507 | T4742_S00006.567 | zinc knuckle domain-containing protein MPE1 |
| OPB37759 | T4742_S00017.26 | zinc transporter |
| OPB43039 | T4742_S00012.276 | zinc transporter |
| OPB46740 | T4742_S00009.182 | zinc transporter |
| OPB38049 | T4742_S00002.96 | Zinc transporter |
| OPB45119 | T4742_S00010.26 | zinc transporter |
| OPB36117 | T4742_S00021.34 | Zinc-dependent alcohol dehydrogenase |
| OPB36123 | T4742_S00021.40 | Zinc-dependent alcohol dehydrogenase |
| OPB36186 | T4742_S00021.104 | zinc-dependent alcohol dehydrogenase |

| | | |
|---|---|---|
| OPB36441 | T4742_S00007.158 | zinc-dependent alcohol dehydrogenase |
| OPB37211 | T4742_S00005.238 | zinc-dependent alcohol dehydrogenase |
| OPB37383 | T4742_S00005.410 | zinc-dependent alcohol dehydrogenase |
| OPB37668 | T4742_S00005.695 | Zinc-dependent alcohol dehydrogenase |
| OPB37712 | T4742_S00005.740 | Zinc-dependent alcohol dehydrogenase |
| OPB37807 | T4742_S00017.74 | Zinc-dependent alcohol dehydrogenase |
| OPB37946 | T4742_S00017.213 | Zinc-dependent alcohol dehydrogenase |
| OPB38670 | T4742_S00002.719 | Zinc-dependent alcohol dehydrogenase |
| OPB38745 | T4742_S00002.794 | Zinc-dependent alcohol dehydrogenase |
| OPB38747 | T4742_S00002.796 | zinc-dependent alcohol dehydrogenase |
| OPB38784 | T4742_S00002.833 | Zinc-dependent alcohol dehydrogenase |
| OPB38915 | T4742_S00002.964 | Zinc-dependent alcohol dehydrogenase |
| OPB39033 | T4742_S00006.92 | zinc-dependent alcohol dehydrogenase |
| OPB39034 | T4742_S00006.93 | zinc-dependent alcohol dehydrogenase |
| OPB39053 | T4742_S00006.112 | Zinc-dependent alcohol dehydrogenase |
| OPB39064 | T4742_S00006.123 | Zinc-dependent alcohol dehydrogenase |
| OPB39087 | T4742_S00006.146 | Zinc-dependent alcohol dehydrogenase |
| OPB39430 | T4742_S00006.490 | zinc-dependent alcohol dehydrogenase |
| OPB40262 | T4742_S00001.341 | Zinc-dependent alcohol dehydrogenase |
| OPB40322 | T4742_S00001.401 | zinc-dependent alcohol dehydrogenase |
| OPB40366 | T4742_S00001.445 | zinc-dependent alcohol dehydrogenase |
| OPB40563 | T4742_S00001.642 | zinc-dependent alcohol dehydrogenase |
| OPB40674 | T4742_S00001.754 | zinc-dependent alcohol dehydrogenase |
| OPB40769 | T4742_S00001.850 | Zinc-dependent alcohol dehydrogenase |
| OPB40818 | T4742_S00001.899 | Zinc-dependent alcohol dehydrogenase |
| OPB40824 | T4742_S00001.905 | zinc-dependent alcohol dehydrogenase |
| OPB40845 | T4742_S00001.926 | Zinc-dependent alcohol dehydrogenase |
| OPB40877 | T4742_S00001.958 | zinc-dependent alcohol dehydrogenase |
| OPB40987 | T4742_S00020.11 | Zinc-dependent alcohol dehydrogenase |
| OPB41210 | T4742_S00011.50 | zinc-dependent alcohol dehydrogenase |
| OPB41280 | T4742_S00011.121 | Zinc-dependent alcohol dehydrogenase |
| OPB41307 | T4742_S00011.148 | Zinc-dependent alcohol dehydrogenase |
| OPB41702 | T4742_S00018.24 | Zinc-dependent alcohol dehydrogenase |
| OPB41858 | T4742_S00018.181 | Zinc-dependent alcohol dehydrogenase |
| OPB41886 | T4742_S00018.209 | zinc-dependent alcohol dehydrogenase |
| OPB42701 | T4742_S00004.807 | zinc-dependent alcohol dehydrogenase |

| | | |
|---|---|---|
| OPB42741 | T4742_S00004.847 | zinc-dependent alcohol dehydrogenase |
| OPB43340 | T4742_S00019.73 | Zinc-dependent alcohol dehydrogenase |
| OPB43451 | T4742_S00019.184 | Zinc-dependent alcohol dehydrogenase |
| OPB43748 | T4742_S00003.19 | zinc-dependent alcohol dehydrogenase |
| OPB43806 | T4742_S00003.77 | Zinc-dependent alcohol dehydrogenase |
| OPB43964 | T4742_S00003.235 | Zinc-dependent alcohol dehydrogenase |
| OPB44238 | T4742_S00003.509 | Zinc-dependent alcohol dehydrogenase |
| OPB44336 | T4742_S00003.607 | Zinc-dependent alcohol dehydrogenase |
| OPB44454 | T4742_S00003.726 | Zinc-dependent alcohol dehydrogenase |
| OPB44508 | T4742_S00003.780 | zinc-dependent alcohol dehydrogenase |
| OPB44587 | T4742_S00003.859 | Zinc-dependent alcohol dehydrogenase |
| OPB44610 | T4742_S00003.882 | zinc-dependent alcohol dehydrogenase |
| OPB44624 | T4742_S00003.896 | Zinc-dependent alcohol dehydrogenase |
| OPB44791 | T4742_S00013.96 | Zinc-dependent alcohol dehydrogenase |
| OPB45739 | T4742_S00014.74 | zinc-dependent alcohol dehydrogenase |
| OPB45772 | T4742_S00014.107 | zinc-dependent alcohol dehydrogenase |
| OPB45912 | T4742_S00014.248 | Zinc-dependent alcohol dehydrogenase |
| OPB46031 | T4742_S00008.97 | Zinc-dependent alcohol dehydrogenase |
| OPB46221 | T4742_S00008.288 | Zinc-dependent alcohol dehydrogenase |
| OPB46337 | T4742_S00008.404 | Zinc-dependent alcohol dehydrogenase |
| OPB46366 | T4742_S00008.433 | Zinc-dependent alcohol dehydrogenase |
| OPB46411 | T4742_S00008.478 | Zinc-dependent alcohol dehydrogenase |
| OPB46484 | T4742_S00008.551 | zinc-dependent alcohol dehydrogenase |
| OPB46534 | T4742_S00008.601 | Zinc-dependent alcohol dehydrogenase |
| OPB47042 | T4742_S00009.484 | Zinc-dependent alcohol dehydrogenase |
| OPB47138 | T4742_S00009.580 | Zinc-dependent alcohol dehydrogenase |
| OPB47161 | T4742_S00009.603 | zinc-dependent alcohol dehydrogenase |
| OPB47168 | T4742_S00009.610 | Zinc-dependent alcohol dehydrogenase |
| OPB42465 | T4742_S00004.567 | Zinc-dependent metalloprotease, ADAM_fungal subgroup |
| OPB39086 | T4742_S00006.145 | zinc-regulated transporter 1 |
| OPB43477 | T4742_S00019.211 | zinc-regulated transporter 1 |
| OPB44658 | T4742_S00003.930 | Zn(2)-C6 fungal-type DNA-binding domain protein [Cordyceps confragosa RCEF 1005] |
| OPB41845 | T4742_S00018.168 | Zn(2Cys6 transcriptional regulator |
| OPB37731 | T4742_S00005.759 | Zn2Cys6 [Trichoderma guizhouense] |
| OPB36109 | T4742_S00021.25 | Zn2-Cys6 binuclear cluster domain protein |
| OPB41354 | T4742_S00011.195 | Zn2Cys6 transcription factor [Trichoderma guizhouense] |

| | | |
|---|---|---|
| OPB39770 | T4742_S00015.114 | Zn2Cys6 transcription regulator, C. albicans Fcr1 |
| OPB35942 | T4742_S00025.15 | Zn2Cys6 transcriptional regulator |
| OPB36182 | T4742_S00021.100 | Zn2Cys6 transcriptional regulator |
| OPB36220 | T4742_S00021.138 | Zn2Cys6 transcriptional regulator |
| OPB36278 | T4742_S00021.197 | Zn2Cys6 transcriptional regulator |
| OPB36304 | T4742_S00007.21 | Zn2Cys6 transcriptional regulator |
| OPB36360 | T4742_S00007.77 | Zn2Cys6 transcriptional regulator |
| OPB36435 | T4742_S00007.152 | Zn2Cys6 transcriptional regulator |
| OPB36457 | T4742_S00007.174 | Zn2Cys6 transcriptional regulator |
| OPB36506 | T4742_S00007.223 | Zn2Cys6 transcriptional regulator |
| OPB36547 | T4742_S00007.264 | Zn2Cys6 transcriptional regulator |
| OPB36556 | T4742_S00007.273 | Zn2Cys6 transcriptional regulator |
| OPB36574 | T4742_S00007.291 | Zn2Cys6 transcriptional regulator |
| OPB36575 | T4742_S00007.292 | Zn2Cys6 transcriptional regulator |
| OPB36781 | T4742_S00007.499 | Zn2Cys6 transcriptional regulator |
| OPB36827 | T4742_S00007.545 | Zn2Cys6 transcriptional regulator |
| OPB36929 | T4742_S00007.647 | Zn2Cys6 transcriptional regulator |
| OPB37079 | T4742_S00005.106 | Zn2Cys6 transcriptional regulator |
| OPB37148 | T4742_S00005.175 | Zn2Cys6 transcriptional regulator |
| OPB37188 | T4742_S00005.215 | Zn2Cys6 transcriptional regulator |
| OPB37198 | T4742_S00005.225 | Zn2Cys6 transcriptional regulator |
| OPB37199 | T4742_S00005.226 | Zn2Cys6 transcriptional regulator |
| OPB37205 | T4742_S00005.232 | Zn2Cys6 transcriptional regulator |
| OPB37210 | T4742_S00005.237 | Zn2Cys6 transcriptional regulator |
| OPB37288 | T4742_S00005.315 | Zn2Cys6 transcriptional regulator |
| OPB37363 | T4742_S00005.390 | Zn2Cys6 transcriptional regulator |
| OPB37365 | T4742_S00005.392 | Zn2Cys6 transcriptional regulator |
| OPB37369 | T4742_S00005.396 | Zn2Cys6 transcriptional regulator |
| OPB37381 | T4742_S00005.408 | Zn2Cys6 transcriptional regulator |
| OPB37409 | T4742_S00005.436 | Zn2Cys6 transcriptional regulator |
| OPB37440 | T4742_S00005.467 | Zn2Cys6 transcriptional regulator |
| OPB37458 | T4742_S00005.485 | Zn2Cys6 transcriptional regulator |
| OPB37479 | T4742_S00005.506 | Zn2Cys6 transcriptional regulator |
| OPB37509 | T4742_S00005.536 | Zn2Cys6 transcriptional regulator |
| OPB37586 | T4742_S00005.613 | Zn2Cys6 transcriptional regulator |
| OPB37627 | T4742_S00005.654 | Zn2Cys6 transcriptional regulator |

| | | |
|---|---|---|
| OPB37637 | T4742_S00005.664 | Zn2Cys6 transcriptional regulator |
| OPB37639 | T4742_S00005.666 | Zn2Cys6 transcriptional regulator |
| OPB37642 | T4742_S00005.669 | Zn2Cys6 transcriptional regulator |
| OPB37651 | T4742_S00005.678 | Zn2Cys6 transcriptional regulator |
| OPB37663 | T4742_S00005.690 | Zn2Cys6 transcriptional regulator |
| OPB37684 | T4742_S00005.711 | Zn2Cys6 transcriptional regulator |
| OPB37685 | T4742_S00005.712 | Zn2Cys6 transcriptional regulator |
| OPB37708 | T4742_S00005.736 | Zn2Cys6 transcriptional regulator |
| OPB37714 | T4742_S00005.742 | Zn2Cys6 transcriptional regulator |
| OPB37721 | T4742_S00005.749 | Zn2Cys6 transcriptional regulator |
| OPB37727 | T4742_S00005.755 | Zn2Cys6 transcriptional regulator |
| OPB37805 | T4742_S00017.72 | Zn2Cys6 transcriptional regulator |
| OPB37813 | T4742_S00017.80 | Zn2Cys6 transcriptional regulator |
| OPB37816 | T4742_S00017.83 | Zn2Cys6 transcriptional regulator |
| OPB37936 | T4742_S00017.203 | Zn2Cys6 transcriptional regulator |
| OPB37945 | T4742_S00017.212 | Zn2Cys6 transcriptional regulator |
| OPB37948 | T4742_S00017.215 | Zn2Cys6 transcriptional regulator |
| OPB37962 | T4742_S00002.9 | Zn2Cys6 transcriptional regulator |
| OPB38020 | T4742_S00002.67 | Zn2Cys6 transcriptional regulator |
| OPB38030 | T4742_S00002.77 | Zn2Cys6 transcriptional regulator |
| OPB38133 | T4742_S00002.180 | Zn2Cys6 transcriptional regulator |
| OPB38223 | T4742_S00002.270 | Zn2Cys6 transcriptional regulator |
| OPB38346 | T4742_S00002.394 | Zn2Cys6 transcriptional regulator |
| OPB38432 | T4742_S00002.480 | Zn2Cys6 transcriptional regulator |
| OPB38609 | T4742_S00002.658 | Zn2Cys6 transcriptional regulator |
| OPB38638 | T4742_S00002.687 | Zn2Cys6 transcriptional regulator |
| OPB38724 | T4742_S00002.773 | Zn2Cys6 transcriptional regulator |
| OPB38742 | T4742_S00002.791 | Zn2Cys6 transcriptional regulator |
| OPB38752 | T4742_S00002.801 | Zn2Cys6 transcriptional regulator |
| OPB38775 | T4742_S00002.824 | Zn2Cys6 transcriptional regulator |
| OPB38798 | T4742_S00002.847 | Zn2Cys6 transcriptional regulator |
| OPB38820 | T4742_S00002.869 | Zn2Cys6 transcriptional regulator |
| OPB38837 | T4742_S00002.886 | Zn2Cys6 transcriptional regulator |
| OPB38861 | T4742_S00002.910 | Zn2Cys6 transcriptional regulator |
| OPB38869 | T4742_S00002.918 | Zn2Cys6 transcriptional regulator |
| OPB38881 | T4742_S00002.930 | Zn2Cys6 transcriptional regulator |

| | | |
|---|---|---|
| OPB38910 | T4742_S00002.959 | Zn2Cys6 transcriptional regulator |
| OPB39070 | T4742_S00006.129 | Zn2Cys6 transcriptional regulator |
| OPB39080 | T4742_S00006.139 | Zn2Cys6 transcriptional regulator |
| OPB39155 | T4742_S00006.215 | Zn2Cys6 transcriptional regulator |
| OPB39174 | T4742_S00006.234 | Zn2Cys6 transcriptional regulator |
| OPB39294 | T4742_S00006.354 | Zn2Cys6 transcriptional regulator |
| OPB39329 | T4742_S00006.389 | Zn2Cys6 transcriptional regulator |
| OPB39396 | T4742_S00006.456 | Zn2Cys6 transcriptional regulator |
| OPB39547 | T4742_S00006.607 | Zn2Cys6 transcriptional regulator |
| OPB39578 | T4742_S00006.638 | Zn2Cys6 transcriptional regulator |
| OPB39695 | T4742_S00015.39 | Zn2Cys6 transcriptional regulator |
| OPB39697 | T4742_S00015.41 | Zn2Cys6 transcriptional regulator |
| OPB40028 | T4742_S00001.107 | Zn2Cys6 transcriptional regulator |
| OPB40083 | T4742_S00001.162 | Zn2Cys6 transcriptional regulator |
| OPB40120 | T4742_S00001.199 | Zn2Cys6 transcriptional regulator |
| OPB40169 | T4742_S00001.248 | Zn2Cys6 transcriptional regulator |
| OPB40292 | T4742_S00001.371 | Zn2Cys6 transcriptional regulator |
| OPB40316 | T4742_S00001.395 | Zn2Cys6 transcriptional regulator |
| OPB40337 | T4742_S00001.416 | Zn2Cys6 transcriptional regulator |
| OPB40367 | T4742_S00001.446 | Zn2Cys6 transcriptional regulator |
| OPB40382 | T4742_S00001.461 | Zn2Cys6 transcriptional regulator |
| OPB40383 | T4742_S00001.462 | Zn2Cys6 transcriptional regulator |
| OPB40413 | T4742_S00001.492 | Zn2Cys6 transcriptional regulator |
| OPB40424 | T4742_S00001.503 | Zn2Cys6 transcriptional regulator |
| OPB40547 | T4742_S00001.626 | Zn2Cys6 transcriptional regulator |
| OPB40611 | T4742_S00001.691 | Zn2Cys6 transcriptional regulator |
| OPB40652 | T4742_S00001.732 | Zn2Cys6 transcriptional regulator |
| OPB40660 | T4742_S00001.740 | Zn2Cys6 transcriptional regulator |
| OPB40667 | T4742_S00001.747 | Zn2Cys6 transcriptional regulator |
| OPB40670 | T4742_S00001.750 | Zn2Cys6 transcriptional regulator |
| OPB40676 | T4742_S00001.756 | Zn2Cys6 transcriptional regulator |
| OPB40696 | T4742_S00001.776 | Zn2Cys6 transcriptional regulator |
| OPB40748 | T4742_S00001.829 | Zn2Cys6 transcriptional regulator |
| OPB40760 | T4742_S00001.841 | Zn2Cys6 transcriptional regulator |
| OPB40765 | T4742_S00001.846 | Zn2Cys6 transcriptional regulator |
| OPB40771 | T4742_S00001.852 | Zn2Cys6 transcriptional regulator |

| | | |
|---|---|---|
| OPB40773 | T4742_S00001.854 | Zn2Cys6 transcriptional regulator |
| OPB40778 | T4742_S00001.859 | Zn2Cys6 transcriptional regulator |
| OPB40783 | T4742_S00001.864 | Zn2Cys6 transcriptional regulator |
| OPB40807 | T4742_S00001.888 | Zn2Cys6 transcriptional regulator |
| OPB40823 | T4742_S00001.904 | Zn2Cys6 transcriptional regulator |
| OPB40825 | T4742_S00001.906 | Zn2Cys6 transcriptional regulator |
| OPB40832 | T4742_S00001.913 | Zn2Cys6 transcriptional regulator |
| OPB40855 | T4742_S00001.936 | Zn2Cys6 transcriptional regulator |
| OPB40901 | T4742_S00001.982 | Zn2Cys6 transcriptional regulator |
| OPB40909 | T4742_S00001.990 | Zn2Cys6 transcriptional regulator |
| OPB40927 | T4742_S00001.1008 | Zn2Cys6 transcriptional regulator |
| OPB40931 | T4742_S00001.1012 | Zn2Cys6 transcriptional regulator |
| OPB40956 | T4742_S00001.1037 | Zn2Cys6 transcriptional regulator |
| OPB40982 | T4742_S00020.6 | Zn2Cys6 transcriptional regulator |
| OPB41008 | T4742_S00020.32 | Zn2Cys6 transcriptional regulator |
| OPB41036 | T4742_S00020.60 | Zn2Cys6 transcriptional regulator |
| OPB41041 | T4742_S00020.65 | Zn2Cys6 transcriptional regulator |
| OPB41088 | T4742_S00020.112 | Zn2Cys6 transcriptional regulator |
| OPB41111 | T4742_S00020.135 | Zn2Cys6 transcriptional regulator |
| OPB41117 | T4742_S00020.141 | Zn2Cys6 transcriptional regulator |
| OPB41118 | T4742_S00020.142 | Zn2Cys6 transcriptional regulator |
| OPB41152 | T4742_S00020.176 | Zn2Cys6 transcriptional regulator |
| OPB41182 | T4742_S00011.22 | Zn2Cys6 transcriptional regulator |
| OPB41232 | T4742_S00011.73 | Zn2Cys6 transcriptional regulator |
| OPB41252 | T4742_S00011.93 | Zn2Cys6 transcriptional regulator |
| OPB41288 | T4742_S00011.129 | Zn2Cys6 transcriptional regulator |
| OPB41376 | T4742_S00011.217 | Zn2Cys6 transcriptional regulator |
| OPB41414 | T4742_S00011.256 | Zn2Cys6 transcriptional regulator |
| OPB41456 | T4742_S00011.299 | Zn2Cys6 transcriptional regulator |
| OPB41470 | T4742_S00011.313 | Zn2Cys6 transcriptional regulator |
| OPB41480 | T4742_S00011.323 | Zn2Cys6 transcriptional regulator |
| OPB41525 | T4742_S00011.368 | Zn2Cys6 transcriptional regulator |
| OPB41528 | T4742_S00011.371 | Zn2Cys6 transcriptional regulator |
| OPB41669 | T4742_S00011.512 | Zn2Cys6 transcriptional regulator |
| OPB41672 | T4742_S00011.515 | Zn2Cys6 transcriptional regulator |
| OPB41706 | T4742_S00018.29 | Zn2Cys6 transcriptional regulator |

| | | |
|---|---|---|
| OPB41740 | T4742_S00018.63 | Zn2Cys6 transcriptional regulator |
| OPB41783 | T4742_S00018.106 | Zn2Cys6 transcriptional regulator |
| OPB41836 | T4742_S00018.159 | Zn2Cys6 transcriptional regulator |
| OPB41838 | T4742_S00018.161 | Zn2Cys6 transcriptional regulator |
| OPB41850 | T4742_S00018.173 | Zn2Cys6 transcriptional regulator |
| OPB41852 | T4742_S00018.175 | Zn2Cys6 transcriptional regulator |
| OPB41856 | T4742_S00018.179 | Zn2Cys6 transcriptional regulator |
| OPB41898 | T4742_S00018.221 | Zn2Cys6 transcriptional regulator |
| OPB41921 | T4742_S00004.23 | Zn2Cys6 transcriptional regulator |
| OPB42043 | T4742_S00004.145 | Zn2Cys6 transcriptional regulator |
| OPB42044 | T4742_S00004.146 | Zn2Cys6 transcriptional regulator |
| OPB42185 | T4742_S00004.287 | Zn2Cys6 transcriptional regulator |
| OPB42349 | T4742_S00004.451 | Zn2Cys6 transcriptional regulator |
| OPB42355 | T4742_S00004.457 | Zn2Cys6 transcriptional regulator |
| OPB42386 | T4742_S00004.488 | Zn2Cys6 transcriptional regulator |
| OPB42409 | T4742_S00004.511 | Zn2Cys6 transcriptional regulator |
| OPB42449 | T4742_S00004.551 | Zn2Cys6 transcriptional regulator |
| OPB42515 | T4742_S00004.617 | Zn2Cys6 transcriptional regulator |
| OPB42542 | T4742_S00004.645 | Zn2Cys6 transcriptional regulator |
| OPB42550 | T4742_S00004.653 | Zn2Cys6 transcriptional regulator |
| OPB42684 | T4742_S00004.790 | Zn2Cys6 transcriptional regulator |
| OPB42716 | T4742_S00004.822 | Zn2Cys6 transcriptional regulator |
| OPB42722 | T4742_S00004.828 | Zn2Cys6 transcriptional regulator |
| OPB42745 | T4742_S00004.851 | Zn2Cys6 transcriptional regulator |
| OPB42747 | T4742_S00004.853 | Zn2Cys6 transcriptional regulator |
| OPB42749 | T4742_S00004.855 | Zn2Cys6 transcriptional regulator |
| OPB42757 | T4742_S00004.863 | Zn2Cys6 transcriptional regulator |
| OPB42811 | T4742_S00012.48 | Zn2Cys6 transcriptional regulator |
| OPB42814 | T4742_S00012.51 | Zn2Cys6 transcriptional regulator |
| OPB42819 | T4742_S00012.56 | Zn2Cys6 transcriptional regulator |
| OPB42831 | T4742_S00012.68 | Zn2Cys6 transcriptional regulator |
| OPB42888 | T4742_S00012.125 | Zn2Cys6 transcriptional regulator |
| OPB42960 | T4742_S00012.197 | Zn2Cys6 transcriptional regulator |
| OPB43094 | T4742_S00012.331 | Zn2Cys6 transcriptional regulator |
| OPB43107 | T4742_S00012.344 | Zn2Cys6 transcriptional regulator |
| OPB43135 | T4742_S00012.372 | Zn2Cys6 transcriptional regulator |

| | | |
|---|---|---|
| OPB43290 | T4742_S00019.23 | Zn2Cys6 transcriptional regulator |
| OPB43302 | T4742_S00019.35 | Zn2Cys6 transcriptional regulator |
| OPB43353 | T4742_S00019.86 | Zn2Cys6 transcriptional regulator |
| OPB43359 | T4742_S00019.92 | Zn2Cys6 transcriptional regulator |
| OPB43364 | T4742_S00019.97 | Zn2Cys6 transcriptional regulator |
| OPB43394 | T4742_S00019.127 | Zn2Cys6 transcriptional regulator |
| OPB43402 | T4742_S00019.135 | Zn2Cys6 transcriptional regulator |
| OPB43403 | T4742_S00019.136 | Zn2Cys6 transcriptional regulator |
| OPB43410 | T4742_S00019.143 | Zn2Cys6 transcriptional regulator |
| OPB43478 | T4742_S00019.212 | Zn2Cys6 transcriptional regulator |
| OPB43524 | T4742_S00016.44 | Zn2Cys6 transcriptional regulator |
| OPB43528 | T4742_S00016.48 | Zn2Cys6 transcriptional regulator |
| OPB43593 | T4742_S00016.113 | Zn2Cys6 transcriptional regulator |
| OPB43625 | T4742_S00016.145 | Zn2Cys6 transcriptional regulator |
| OPB43650 | T4742_S00016.170 | Zn2Cys6 transcriptional regulator |
| OPB43673 | T4742_S00016.193 | Zn2Cys6 transcriptional regulator |
| OPB43693 | T4742_S00016.213 | Zn2Cys6 transcriptional regulator |
| OPB43725 | T4742_S00016.245 | Zn2Cys6 transcriptional regulator |
| OPB43729 | T4742_S00016.249 | Zn2Cys6 transcriptional regulator |
| OPB43737 | T4742_S00003.8 | Zn2Cys6 transcriptional regulator |
| OPB43766 | T4742_S00003.37 | Zn2Cys6 transcriptional regulator |
| OPB43786 | T4742_S00003.57 | Zn2Cys6 transcriptional regulator |
| OPB43808 | T4742_S00003.79 | Zn2Cys6 transcriptional regulator |
| OPB43832 | T4742_S00003.103 | Zn2Cys6 transcriptional regulator |
| OPB43842 | T4742_S00003.113 | Zn2Cys6 transcriptional regulator |
| OPB43880 | T4742_S00003.151 | Zn2Cys6 transcriptional regulator |
| OPB43883 | T4742_S00003.154 | Zn2Cys6 transcriptional regulator |
| OPB43889 | T4742_S00003.160 | Zn2Cys6 transcriptional regulator |
| OPB43966 | T4742_S00003.237 | Zn2Cys6 transcriptional regulator |
| OPB43971 | T4742_S00003.242 | Zn2Cys6 transcriptional regulator |
| OPB44028 | T4742_S00003.299 | Zn2Cys6 transcriptional regulator |
| OPB44075 | T4742_S00003.346 | Zn2Cys6 transcriptional regulator |
| OPB44162 | T4742_S00003.433 | Zn2Cys6 transcriptional regulator |
| OPB44199 | T4742_S00003.470 | Zn2Cys6 transcriptional regulator |
| OPB44415 | T4742_S00003.686 | Zn2Cys6 transcriptional regulator |
| OPB44463 | T4742_S00003.735 | Zn2Cys6 transcriptional regulator |

| | | |
|---|---|---|
| OPB44464 | T4742_S00003.736 | Zn2Cys6 transcriptional regulator |
| OPB44481 | T4742_S00003.753 | Zn2Cys6 transcriptional regulator |
| OPB44518 | T4742_S00003.790 | Zn2Cys6 transcriptional regulator |
| OPB44534 | T4742_S00003.806 | Zn2Cys6 transcriptional regulator |
| OPB44573 | T4742_S00003.845 | Zn2Cys6 transcriptional regulator |
| OPB44595 | T4742_S00003.867 | Zn2Cys6 transcriptional regulator |
| OPB44612 | T4742_S00003.884 | Zn2Cys6 transcriptional regulator |
| OPB44725 | T4742_S00013.30 | Zn2Cys6 transcriptional regulator |
| OPB44783 | T4742_S00013.88 | Zn2Cys6 transcriptional regulator |
| OPB44789 | T4742_S00013.94 | Zn2Cys6 transcriptional regulator |
| OPB44793 | T4742_S00013.98 | Zn2Cys6 transcriptional regulator |
| OPB44856 | T4742_S00013.161 | Zn2Cys6 transcriptional regulator |
| OPB44864 | T4742_S00013.169 | Zn2Cys6 transcriptional regulator |
| OPB44954 | T4742_S00013.259 | Zn2Cys6 transcriptional regulator |
| OPB44968 | T4742_S00013.273 | Zn2Cys6 transcriptional regulator |
| OPB45020 | T4742_S00013.325 | Zn2Cys6 transcriptional regulator |
| OPB45182 | T4742_S00010.89 | Zn2Cys6 transcriptional regulator |
| OPB45202 | T4742_S00010.110 | Zn2Cys6 transcriptional regulator |
| OPB45240 | T4742_S00010.149 | Zn2Cys6 transcriptional regulator |
| OPB45256 | T4742_S00010.165 | Zn2Cys6 transcriptional regulator |
| OPB45395 | T4742_S00010.306 | Zn2Cys6 transcriptional regulator |
| OPB45432 | T4742_S00010.343 | Zn2Cys6 transcriptional regulator |
| OPB45475 | T4742_S00010.386 | Zn2Cys6 transcriptional regulator |
| OPB45542 | T4742_S00010.453 | Zn2Cys6 transcriptional regulator |
| OPB45651 | T4742_S00010.562 | Zn2Cys6 transcriptional regulator |
| OPB45656 | T4742_S00010.567 | Zn2Cys6 transcriptional regulator |
| OPB45704 | T4742_S00014.38 | Zn2Cys6 transcriptional regulator |
| OPB45729 | T4742_S00014.64 | Zn2Cys6 transcriptional regulator |
| OPB45756 | T4742_S00014.91 | Zn2Cys6 transcriptional regulator |
| OPB45814 | T4742_S00014.149 | Zn2Cys6 transcriptional regulator |
| OPB45828 | T4742_S00014.163 | Zn2Cys6 transcriptional regulator |
| OPB45852 | T4742_S00014.187 | Zn2Cys6 transcriptional regulator |
| OPB45878 | T4742_S00014.213 | Zn2Cys6 transcriptional regulator |
| OPB45914 | T4742_S00014.250 | Zn2Cys6 transcriptional regulator |
| OPB45962 | T4742_S00008.28 | Zn2Cys6 transcriptional regulator |
| OPB45996 | T4742_S00008.62 | Zn2Cys6 transcriptional regulator |

| | | |
|---|---|---|
| OPB46004 | T4742_S00008.70 | Zn2Cys6 transcriptional regulator |
| OPB46010 | T4742_S00008.76 | Zn2Cys6 transcriptional regulator |
| OPB46022 | T4742_S00008.88 | Zn2Cys6 transcriptional regulator |
| OPB46025 | T4742_S00008.91 | Zn2Cys6 transcriptional regulator |
| OPB46058 | T4742_S00008.125 | Zn2Cys6 transcriptional regulator |
| OPB46110 | T4742_S00008.177 | Zn2Cys6 transcriptional regulator |
| OPB46112 | T4742_S00008.179 | Zn2Cys6 transcriptional regulator |
| OPB46122 | T4742_S00008.189 | Zn2Cys6 transcriptional regulator |
| OPB46136 | T4742_S00008.203 | Zn2Cys6 transcriptional regulator |
| OPB46181 | T4742_S00008.248 | Zn2Cys6 transcriptional regulator |
| OPB46195 | T4742_S00008.262 | Zn2Cys6 transcriptional regulator |
| OPB46222 | T4742_S00008.289 | Zn2Cys6 transcriptional regulator |
| OPB46248 | T4742_S00008.315 | Zn2Cys6 transcriptional regulator |
| OPB46265 | T4742_S00008.332 | Zn2Cys6 transcriptional regulator |
| OPB46307 | T4742_S00008.374 | Zn2Cys6 transcriptional regulator |
| OPB46359 | T4742_S00008.426 | Zn2Cys6 transcriptional regulator |
| OPB46364 | T4742_S00008.431 | Zn2Cys6 transcriptional regulator |
| OPB46368 | T4742_S00008.435 | Zn2Cys6 transcriptional regulator |
| OPB46417 | T4742_S00008.484 | Zn2Cys6 transcriptional regulator |
| OPB46489 | T4742_S00008.556 | Zn2Cys6 transcriptional regulator |
| OPB46492 | T4742_S00008.559 | Zn2Cys6 transcriptional regulator |
| OPB46494 | T4742_S00008.561 | Zn2Cys6 transcriptional regulator |
| OPB46533 | T4742_S00008.600 | Zn2Cys6 transcriptional regulator |
| OPB46538 | T4742_S00008.605 | Zn2Cys6 transcriptional regulator |
| OPB46678 | T4742_S00009.120 | Zn2Cys6 transcriptional regulator |
| OPB46797 | T4742_S00009.239 | Zn2Cys6 transcriptional regulator |
| OPB46906 | T4742_S00009.348 | Zn2Cys6 transcriptional regulator |
| OPB46914 | T4742_S00009.356 | Zn2Cys6 transcriptional regulator |
| OPB46984 | T4742_S00009.426 | Zn2Cys6 transcriptional regulator |
| OPB47007 | T4742_S00009.449 | Zn2Cys6 transcriptional regulator |
| OPB47027 | T4742_S00009.469 | Zn2Cys6 transcriptional regulator |
| OPB47066 | T4742_S00009.508 | Zn2Cys6 transcriptional regulator |
| OPB47092 | T4742_S00009.534 | Zn2Cys6 transcriptional regulator |
| OPB47136 | T4742_S00009.578 | Zn2Cys6 transcriptional regulator |
| OPB47156 | T4742_S00009.598 | Zn2Cys6 transcriptional regulator |
| OPB42957 | T4742_S00012.194 | Zn2Cys6 transcriptional regulator PRO1 (adv1), involved in hyphal fusion and fruiting body development |

| | | |
|---|---|---|
| OPB38038 | T4742_S00002.85 | Zn2Cys6 transcriptional regulator XYR1 |
| OPB36259 | T4742_S00021.177 | Zn2Cys6 transcriptional regulator [Trichoderma guizhouense] |
| OPB37441 | T4742_S00005.468 | Zn2Cys6 transcriptional regulator [Trichoderma guizhouense] |
| OPB36832 | T4742_S00007.550 | Zn2Cys6 transcriptional regulator ACE3 |
| OPB37796 | T4742_S00017.63 | Zn2Cys6 transcriptional regulator ADA3 |
| OPB39856 | T4742_S00015.200 | Zn2Cys6 transcriptional regulator AmyR, amylase regulator |
| OPB38568 | T4742_S00002.617 | Zn2Cys6 transcriptional regulator CLR-2 |
| OPB40098 | T4742_S00001.177 | Zn2Cys6 transcriptional regulator CTF1ß (cutinase) |
| OPB38832 | T4742_S00002.881 | Zn2Cys6 transcriptional regulator NIR1 |
| OPB41998 | T4742_S00004.100 | Zn2Cys6 transcriptional regulator TamA |
| OPB37762 | T4742_S00017.29 | Zn2Cys6 transcriptional regulator UME6 |
| OPB37540 | T4742_S00005.567 | Zn2Cys6 transcriptional regulator, plus SCDR domain |
| OPB40985 | T4742_S00020.9 | Zn2Cys6 transcriptional regulator, plus SCDR domain |
| OPB38421 | T4742_S00002.469 | Zn2Cys6 transcriptional regulator, purine catabolism |
| OPB38040 | T4742_S00002.87 | Zn2Cys6 transcriptional regulator, related to NIT4 |
| OPB46443 | T4742_S00008.510 | Zn2Cys6 transcriptional regulator, RosA like |
| OPB41865 | T4742_S00018.188 | Zn2Cys6 trascriptional regulator |
| OPB38631 | T4742_S00002.680 | Zn2Cys6 ztanscriptional regulator |
| OPB40755 | T4742_S00001.836 | Zn2Cys6 ztanscriptional regulator |
| OPB43318 | T4742_S00019.51 | Zn-finger protein AN1 |
| OPB38796 | T4742_S00002.845 | zuotin, HSP40 family |
| OPB45056 | T4742_S00013.361 | γ-adaptin, apl4, large subunit of the adaptor protein (AP) complex of clathrin-coated vesicles |
| OPB42613 | T4742_S00004.717 | γ-glutamyl phosphate reductase GPR |
| OPB39128 | T4742_S00006.188 | γ-glutamyltranspeptidase |
| OPB43850 | T4742_S00003.121 | γ-glutamyltranspeptidase |

**The manual annotation of defferentially expressed genes of *Fusarium oxysporum* f. sp. *cubense* 4 (Foc4)**

| NCBI assessio | PI | Annotation |
|---|---|---|
| 88 | FOC4g11556 | L-amino-acid oxidase |
| 90 | FOC4g11557 | AA9 copper-dependent polysaccharide monooxygenase |
| 32 | FOC4g11601 | unknown protein |
| 48 | FOC4g11617 | Zn2Cys6 transcriptional regulator |
| 03 | FOC4g11674 | copia-type retrotransposon |
| 38 | FOC4g11709 | MFS sugar permease |
| 51 | FOC4g11722 | MDR, Multidrug resistance-associated protein |
| 53 | FOC4g11724 | unknown protein |
| 58 | FOC4g11729 | Siderophore iron transporter mirB |

| | | |
|---|---|---|
| 61 | FOC4g11732 | orphan protein |
| 63 | FOC4g11734 | RNAse H1 |
| 75 | FOC4g11746 | unknown protein with hAT dimerization domain |
| 17 | FOC4g11789 | RTA1 transmembrane protein; binds extracellular toxins |
| 31 | FOC4g11803 | flavin-containing monooxygenase |
| 33 | FOC4g11805 | AA3_2 GMC oxidoreductase |
| 64 | FOC4g11840 | Zn2Cys6 transcriptional activator |
| 83 | FOC4g11858 | Cell wall protein SED1 |
| 96 | FOC4g11872 | aryl alcohol dehydrogenase |
| 14 | FOC4g11890 | aminoglycoside phosphotransferase |
| 34 | FOC4g11910 | WD domain, G-beta repeat |
| 48 | FOC4g12030 | unknown protein |
| 58 | FOC4g12039 | unknown protein |
| 66 | FOC4g12046 | ATP-dependent DNA helicase tlh2, partial |
| 69 | FOC4g12049 | RNAse H1 |
| 71 | FOC4g12051 | Pyridoxine biosynthesis protein PDX1 |
| 76 | FOC4g12056 | unknown protein |
| 77 | FOC4g12057 | alpha/beta hydrolase fold |
| 00 | FOC4g12080 | ribonuclease H1 |
| 11 | FOC4g12092 | cardiolipin-specific deacylase, mitochondrial |
| 16 | FOC4g12097 | unknown protein |
| 18 | FOC4g12099 | peroxisomal catalase |
| 38 | FOC4g12120 | MFS quinate permease |
| 55 | FOC4g12140 | Zn2Cys6 transcriptional activator |
| 64 | FOC4g12149 | LrgB membrane protein, controls murein hydrolase in bacteria |
| 67 | FOC4g12152 | MFS, hexose transporter HXT15 |
| 93 | FOC4g12181 | SET-type methyltransferase |
| 94 | FOC4g12182 | Sterol 24-C-methyltransferase |
| 98 | FOC4g12186 | CAAX protease self-immunity |
| 21 | FOC4g12209 | [illegible] ubiquitin-hydrolases and other protein |
| 67 | FOC4g12257 | 6-phosphogluconate dehydrogenase, decarboxylating |
| 16 | FOC4g14056 | unknown protein |
| 44 | FOC4g14084 | Glutathione S-transferase |
| 45 | FOC4g14085 | GH16 endo-ß-1,3/1.4-glucanase |
| 55 | FOC4g14095 | Histidine kinase, part of a two component signal transduction system |
| 70 | FOC4g14111 | Monocarboxylate transporter |

| | | |
|---|---|---|
| 72 | FOC4g14113 | integral membrane protein |
| 87 | FOC4g14128 | Sterol-4-alpha-carboxylate 3-dehydrogenase, decarboxylating |
| 06 | FOC4g14148 | GNAT-acetyltransferase |
| 39 | FOC4g14185 | unknown protein |
| 43 | FOC4g14189 | HET domain protein |
| 68 | FOC4g14217 | GH31 α-1,6-glucosidase |
| 29 | FOC4g14280 | Homocitrate synthase, mitochondrial |
| 41 | FOC4g14292 | 60S ribosomal protein L23-B |
| 48 | FOC4g14299 | Deoxyribodipyrimidine photo-lyase |
| 52 | FOC4g14303 | unknown protein |
| 58 | FOC4g14309 | peptide transporter |
| 73 | FOC4g14325 | Zn2Cys6 transcriptional activator |
| 78 | FOC4g14331 | DDE superfamily endonuclease |
| 85 | FOC4g14338 | FAD-containing monooxygenase EthA |
| 18 | FOC4g14372 | DNA damage response protein WSS1 |
| 49 | FOC4g14404 | Thiol-specific monooxygenase |
| 58 | FOC4g14413 | 3-hydroxypropionyl-coenzyme A dehydratase |
| 60 | FOC4g14520 | NAD(P)-dependent dehydrogenase |
| 11 | FOC4g14573 | Ubiquinone/menaquinone biosynthesis methyltransferase ubiE |
| 41 | FOC4g14604 | FAD-binding oxidoreductase |
| 85 | FOC4g14854 | unknown protein |
| 59 | FOC4g14931 | Cellobiose dehydrogenase with Cytb561 domain |
| 22 | FOC4g14996 | GH5 Glycoside Hydrolase Family 5 / Subf 24 |
| 35 | FOC4g15009 | Siderophore iron transporter mirB |
| 53 | FOC4g15028 | Cytochrome c oxidase copper chaperone |
| 74 | FOC4g15049 | GH7 endo-ß-1,4-glucanase |
| 81 | FOC4g15056 | Molybdenum cofactor sulfurase |
| 03 | FOC4g15079 | AA3_2 GMC oxidoreductase |
| 61 | FOC4g15139 | unknown protein |
| 62 | FOC4g15140 | PL4 rhamnogalacturonan endolyase |
| 39 | FOC4g15221 | Cytochrome P450 4V2 |
| 65 | FOC4g15249 | fermentation/respiration switch protein FrsA |
| 46 | FOC4g15333 | RING finger protein B |
| 66 | FOC4g15353 | Histone-lysine N-methyltransferase |
| 73 | FOC4g15360 | unknown protein |
| 74 | FOC4g15361 | short chain dehydrogenase/reductase |

| | | |
|---|---|---|
| 36 | FOC4g15425 | phosphate carrier, mitochondrial |
| 67 | FOC4g05981 | Glycerol-3-phosphate dehydrogenase, mitochondrial |
| 86 | FOC4g06000 | Cytochrome P450 4F12 |
| 07 | FOC4g06022 | AMP-binding protein, possibly acyl-CoA synthetase |
| 08 | FOC4g06023 | lysine N-acyltransferase |
| 09 | FOC4g06024 | Pyridine nucleotide-disulphide oxidoreductase, class I |
| 10 | FOC4g06025 | MDR multidrug transporter; Leptomycin B resistance protein |
| 22 | FOC4g06038 | A1 aspartyl pepidase |
| 24 | FOC4g06040 | unknown protein |
| 58 | FOC4g06075 | related to bacterio-opsin activator, phototropin 2 |
| 61 | FOC4g06078 | unknown protein |
| 62 | FOC4g06079 | Meiotically up-regulated protein 14 protein |
| 63 | FOC4g06080 | Carboxylesterase family |
| 27 | FOC4g06146 | PL4 pectate lyase |
| 29 | FOC4g06148 | DDE superfamily endonuclease, possibly membrane bound |
| 33 | FOC4g06153 | MFS permease |
| 50 | FOC4g06170 | Glutathione S-transferase |
| 56 | FOC4g06176 | reverse trascriptase/integrase |
| 61 | FOC4g06182 | orphan protein |
| 63 | FOC4g06184 | Arginase |
| 64 | FOC4g06185 | arrestin_N terminal |
| 80 | FOC4g06202 | Flavohemoprotein |
| 96 | FOC4g06219 | unknown protein |
| 99 | FOC4g06222 | unknown protein |
| 24 | FOC4g06249 | unknown protein |
| 43 | FOC4g06268 | MFS D-galactonate permease |
| 47 | FOC4g06272 | Carboxypeptidase cpdS |
| 69 | FOC4g06295 | CE8 pectin methylesterase |
| 72 | FOC4g06298 | unknown protein |
| 85 | FOC4g06314 | unknown protein |
| 95 | FOC4g06326 | unknown protein |
| 01 | FOC4g06333 | MFS L-fucose permease |
| 04 | FOC4g06337 | Carbonic anhydrase |
| 09 | FOC4g06342 | HET domain protein |
| 18 | FOC4g06351 | Amino-acid permease inda1 |
| 19 | FOC4g06352 | MFS quinate permease |

| | | |
|---|---|---|
| 22 | FOC4g06355 | MFS α-glucosides permease MPH2 |
| 31 | FOC4g06364 | NmrA-like transcription repressor |
| 64 | FOC4g06402 | NmrA-like transcription repressor |
| 04 | FOC4g06443 | unknown protein |
| 10 | FOC4g06449 | Cytochrome P450 monooxygenase |
| 22 | FOC4g06462 | MFS-type transporter PB1E7.08c |
| 42 | FOC4g06484 | haloacid dehalogenase 4A |
| 45 | FOC4g06487 | unknown protein |
| 52 | FOC4g06494 | unknown protein |
| 66 | FOC4g01062 | Zn2Cys6 transcriptional activator |
| 35 | FOC4g01133 | Flavohemoprotein |
| 70 | FOC4g13163 | SAM-dependent methyltransferase |
| 17 | FOC4g13213 | Ankyrin |
| 19 | FOC4g13215 | MFS related to multidrug resistant protein |
| 44 | FOC4g13245 | L-asparaginase |
| 68 | FOC4g13272 | RNAse H1 |
| 78 | FOC4g13282 | retinol dehydrogenase |
| 79 | FOC4g13283 | SAM-dependent methyltransferase |
| 91 | FOC4g13295 | Cytochrome P450-Salicylate hydroxylase |
| 93 | FOC4g13297 | short chain dehydrogenaye/reductase |
| 05 | FOC4g13310 | argininosuccinate lyase |
| 06 | FOC4g13311 | short-chain dehydrogenase/reductase, untypical |
| 09 | FOC4g13314 | unknown protein with hAT dimerization domain |
| 46 | FOC4g13361 | Glutathione S-transferase |
| 47 | FOC4g13362 | unknown protein |
| 48 | FOC4g13363 | Caspase domain |
| 52 | FOC4g13366 | FAD/FMN-dependent oxidoreductase |
| 61 | FOC4g13375 | unknown protein |
| 62 | FOC4g13376 | FAD-dependent oxydoreductase |
| 70 | FOC4g13385 | short-chain dehydrogenase/reductase |
| 75 | FOC4g13391 | Threonine dehydratase |
| 82 | FOC4g13399 | L-amino-acid oxidase |
| 13 | FOC4g13432 | AA3_2 GMC oxidoreductase |
| 15 | FOC4g13434 | 60S acidic ribosomal protein P1 |
| 17 | FOC4g13436 | unknown protein |
| 26 | FOC4g13445 | Vitamin H transporter |

| | | |
|---|---|---|
| 51 | FOC4g13469 | NmrA-like transcription repressor |
| 54 | FOC4g13472 | GH28 endopolygalacturonase |
| 58 | FOC4g13476 | Catalase-peroxidase 2 |
| 66 | FOC4g13484 | unknown protein |
| 73 | FOC4g13493 | NmrA-like transcription repressor |
| 90 | FOC4g13510 | amino acid permease LysP |
| 29 | FOC4g13549 | cerato-platanin |
| 33 | FOC4g13553 | aldo/keto reductase |
| 15 | FOC4g13640 | bZIP transcription factor |
| 29 | FOC4g13655 | 60S ribosomal protein L44 |
| 67 | FOC4g13695 | CCHC-zinc finger protein; DNA binding |
| 77 | FOC4g13705 | unknown protein |
| 92 | FOC4g13720 | Quinone oxidoreductase PIG3 |
| 98 | FOC4g13727 | Ketol-acid reductoisomerase, mitochondrial |
| 18 | FOC4g13747 | 2-haloalkanoic acid dehalogenase |
| 19 | FOC4g13748 | MFS multidrug transporter |
| 31 | FOC4g13763 | Zinc-dependent medium chain dehydrogenase/reductase |
| 32 | FOC4g13764 | 60S ribosomal protein L22 |
| 35 | FOC4g13767 | unknown protein |
| 52 | FOC4g13786 | unknown protein |
| 71 | FOC4g13805 | unknown protein |
| 98 | FOC4g13834 | mannitol dehydrogenase |
| 42 | FOC4g13883 | Fatty acid-binding protein |
| 43 | FOC4g13884 | 2-nitropropan dioxygenase |
| 60 | FOC4g13904 | Calcineurin-like metallophosphoesterase |
| 65 | FOC4g13910 | FAD/FMN-dependent oxidoreductase |
| 53 | FOC4g14000 | Isocitrate lyase |
| 91 | FOC4g14040 | Sterol O-acyltransferase 2 |
| 14 | FOC4g10285 | HET domain protein |
| 15 | FOC4g10286 | 28.5 kDa protein in 7S RNA 5\'region |
| 16 | FOC4g10390 | 60S ribosomal protein L43 |
| 68 | FOC4g10445 | non-LTR RNAse |
| 74 | FOC4g10451 | 40S ribosomal protein S12 |
| 62 | FOC4g10546 | short chain dehydrogenase/reductase, hydroxysteroid dehydrogenase |
| 76 | FOC4g10561 | unknown protein |
| 35 | FOC4g10623 | unknown protein |

| | | |
|---|---|---|
| 56 | FOC4g10643 | unknown protein |
| 90 | FOC4g10679 | unknown protein |
| 00 | FOC4g10690 | Cycloeucalenol cycloisomerase |
| 30 | FOC4g10721 | fasciclin-domain adhesion protein |
| 32 | FOC4g10723 | short chain dehydrogenase/reductase; phenylcoumaran benzylic ether reductase |
| 33 | FOC4g10724 | catalase |
| 34 | FOC4g10725 | RTA1 transmembrane protein; binds extracellular toxins |
| 38 | FOC4g10729 | unknown protein with hAT dimerization domain |
| 65 | FOC4g10758 | Succinate/fumarate mitochondrial transporter |
| 71 | FOC4g10764 | unknown protein with ICP4 domain |
| 74 | FOC4g10767 | Benzoylformate decarboxylase |
| 95 | FOC4g10789 | unknown protein |
| 08 | FOC4g10803 | DDE superfamily endonuclease |
| 22 | FOC4g10817 | unknown protein |
| 37 | FOC4g10833 | unknown protein |
| 46 | FOC4g10843 | short chain dehydrogenase/reductase; phenylcoumaran benzylic ether reductase |
| 48 | FOC4g10845 | Thioredoxin reductase |
| 49 | FOC4g10846 | Pimeloyl-ACP methyl ester carboxylesterase |
| 66 | FOC4g10864 | restriction endonuclease, putative, DUF3883 protein |
| 73 | FOC4g10871 | Expansin-like protein |
| 76 | FOC4g10874 | Zinc-dependent alcohol dehydrogenase |
| 83 | FOC4g10882 | unknown protein |
| 84 | FOC4g10883 | unknown protein |
| 90 | FOC4g10889 | Epoxide hydrolase, soluble |
| 91 | FOC4g10890 | Acyl-CoA dehydrogenase |
| 01 | FOC4g10900 | PL1 pectate lyase |
| 02 | FOC4g10901 | unknown protein |
| 06 | FOC4g07077 | GH5 endo-ß-1,4-glucanase plus CBM1 |
| 07 | FOC4g07078 | unknown protein |
| 28 | FOC4g07100 | unknown protein |
| 32 | FOC4g07105 | unknown protein |
| 50 | FOC4g07124 | Cystathionine beta-lyases/cystathionine gamma-synthases |
| 99 | FOC4g07174 | Threonine dehydratase |
| 26 | FOC4g07304 | CE5 acetyl-xylan esterase |
| 33 | FOC4g07312 | MFS toxin efflux pump |
| 43 | FOC4g07323 | unknown protein |

| | | |
|---|---|---|
| [illegible] 45 | FOC4g07325 | retinol dehydrogenase |
| [illegible] 46 | FOC4g07326 | Ankyrin |
| [illegible] 54 | FOC4g07334 | MFS L-fucose-proton symporter |
| [illegible] 55 | FOC4g07335 | Phosphate-repressible phosphate permease |
| [illegible] 69 | FOC4g07350 | PL1 pectate lyase |
| [illegible] 84 | FOC4g07365 | GH5 Glycoside Hydrolase Family 5 / Subf 5 |
| [illegible] 37 | FOC4g07420 | ABC transporter for intermediates of thiamine biosynthesis |
| [illegible] 38 | FOC4g07421 | 5-methylthioadenosine/S-adenosylhomocysteine deaminase |
| [illegible] 39 | FOC4g07422 | ß-hydroxysteroid dehydrogenase |
| [illegible] 43 | FOC4g07426 | GH1 ß-glycosidase |
| [illegible] 48 | FOC4g07431 | metallopeptidase, putative |
| [illegible] 49 | FOC4g07432 | Pimeloyl-ACP-acyl ester carboxyesterase |
| [illegible] 51 | FOC4g07434 | Zn2Cys6 transcriptional activator |
| [illegible] 57 | FOC4g07441 | NADP-dependent alcohol dehydrogenase 6 |
| [illegible] 58 | FOC4g07442 | unknown protein |
| [illegible] 59 | FOC4g07443 | unknown protein |
| [illegible] 72 | FOC4g07456 | UDP-glucose 4-epimerase |
| [illegible] 88 | FOC4g07473 | MFS toxin efflux pump |
| [illegible] 99 | FOC4g07486 | DNAJ protein, involved in HSP70 regulation |
| [illegible] 00 | FOC4g07488 | unknown protein |
| [illegible] 21 | FOC4g07512 | HNH-endonuclease |
| [illegible] 46 | FOC4g07540 | unknown protein |
| [illegible] 50 | FOC4g07544 | MFS permease |
| [illegible] 60 | FOC4g07556 | NmrA like protein |
| [illegible] 61 | FOC4g07557 | GT17 ß-1,4-mannosyl-glycoprotein N-acetylglucosaminyltransferase |
| [illegible] 68 | FOC4g07565 | unknown protein |
| [illegible] 69 | FOC4g07566 | unknown protein |
| [illegible] 77 | FOC4g07574 | unknown protein |
| [illegible] 00 | FOC4g07597 | unknown protein |
| [illegible] 14 | FOC4g07615 | cupin-related protein |
| [illegible] 61 | FOC4g11036 | RNAse H1 |
| [illegible] 66 | FOC4g11041 | Nucleosome assembly protein |
| [illegible] 72 | FOC4g11047 | ABC transporter |
| [illegible] 18 | FOC4g11095 | ABC transporter, multidrug resistance protein |
| [illegible] 31 | FOC4g11108 | peptide transporter ptr2 |
| [illegible] 61 | FOC4g11139 | 40S ribosomal protein S29 |

| | | |
|---|---|---|
| 70 | FOC4g11148 | NADP-specific glutamate dehydrogenase |
| 21 | FOC4g11199 | ChrH transcriptional activator, cupin protein |
| 55 | FOC4g11338 | unknown protein |
| 68 | FOC4g11351 | orphan protein |
| 68 | FOC4g11454 | Reticulocyte-binding protein 2 like protein a |
| 03 | FOC4g11490 | unknown protein |
| 46 | FOC4g07647 | short chain dehydrogenase/reductase |
| 56 | FOC4g07659 | Rieske non-heme iron aromatic ring dioxygenase |
| 57 | FOC4g07660 | unknown protein |
| 85 | FOC4g07688 | SAM-dependent methyltransferase |
| 93 | FOC4g07696 | Aldehyde reductase 2 |
| 01 | FOC4g07704 | unknown protein with  DUF1771 domain protein |
| 02 | FOC4g07705 | 40S ribosomal protein S20 |
| 03 | FOC4g07706 | DJ-1/PfpI family |
| 07 | FOC4g07710 | GH31 α-1,6-glucosidase |
| 17 | FOC4g07721 | Carboxylesterase family |
| 30 | FOC4g07735 | O-acyl transferase, aromatic substrates |
| 48 | FOC4g07755 | Carboxylesterase family |
| 54 | FOC4g07761 | D-isomer soecific hydroxyacid dehydrogenase |
| 59 | FOC4g07767 | GH32 inulinase plus CBM38 inulin binding domain |
| 61 | FOC4g07769 | unknown protein |
| 73 | FOC4g07781 | Zinc-dependent alcohol dehydrogenase |
| 74 | FOC4g07782 | amidohydrolase YcaC, secreted |
| 81 | FOC4g07789 | unknown protein |
| 16 | FOC4g07826 | AA9 copper-dependent polysaccharide monooxygenase |
| 17 | FOC4g07827 | Integral membrane protein |
| 21 | FOC4g07832 | amino acid permease GAP1 |
| 28 | FOC4g07839 | integral membrane protein |
| 83 | FOC4g07894 | NADH oxidase |
| 91 | FOC4g07902 | extracellular plant pathogenesis protein |
| 92 | FOC4g07903 | Betaine aldehyde dehydrogenase |
| 05 | FOC4g07916 | unknown protein |
| 12 | FOC4g07924 | HLH transcriptional regulator |
| 18 | FOC4g07930 | unknown protein |
| 22 | FOC4g07934 | cytidine deaminase-like protein, secreted |
| 70 | FOC4g07984 | aldehyde dehydrogenase, NADP-specific |

| | | |
|---|---|---|
| 94 | FOC4g08009 | Eburicol 14-alpha-demethylase |
| 98 | FOC4g08013 | tubulin-tyrosine ligase |
| 13 | FOC4g08028 | flavin-containing monooxygenase |
| 34 | FOC4g08049 | Superoxide dismutase 1 copper chaperone |
| 70 | FOC4g08086 | Glutathione S-transferase |
| 03 | FOC4g08122 | MFS drug efflux transporter |
| 12 | FOC4g08131 | unknown protein |
| 27 | FOC4g08147 | MFS glucose transporter RCO1-like |
| 31 | FOC4g08155 | unknown protein |
| 34 | FOC4g08158 | unknown protein |
| 39 | FOC4g08163 | Zinc-dependent alcohol dehydrogenase |
| 40 | FOC4g08164 | Monocarboxylate transporter |
| 44 | FOC4g08168 | N-acetyltransferase ats1 |
| 73 | FOC4g08199 | Calcium-transporting ATPase 3 |
| 81 | FOC4g08208 | Bifunctional purine biosynthetic protein ADE1 |
| 82 | FOC4g08209 | Bifunctional purine biosynthetic protein ADE1 |
| 83 | FOC4g08210 | short chain dehydrogenase/reductase |
| 06 | FOC4g08234 | unknown protein |
| 19 | FOC4g08250 | unknown protein |
| 26 | FOC4g06515 | unknown protein |
| 39 | FOC4g06528 | unknown protein |
| 45 | FOC4g06534 | Tripeptidyl aminopeptidase |
| 46 | FOC4g06535 | AA3_2 GMC oxidoreductase |
| 68 | FOC4g06558 | Cytochrome P450 monooxygenase |
| 83 | FOC4g06573 | unknown protein |
| 90 | FOC4g06580 | reverse transcriptase with Gag-preintegration domain |
| 28 | FOC4g06624 | Alcohol dehydrogenase 1 |
| 31 | FOC4g06627 | Ankyrin |
| 47 | FOC4g06643 | amino acid permease LysP |
| 69 | FOC4g06667 | glutathione S-transferase |
| 82 | FOC4g06681 | unknown protein |
| 83 | FOC4g06682 | unknown protein |
| 85 | FOC4g06684 | GNAT family N-acetyltransferase |
| 11 | FOC4g06712 | nucleobase/cation transporter |
| 16 | FOC4g06717 | Zn2Cys6 transcriptional activator |
| 26 | FOC4g06728 | GPCR Class B. secretin receptor |

| | | |
|---|---|---|
| 28 | FOC4g06730 | Ankyrin |
| 37 | FOC4g06741 | Cytochrome P450 monooxygenase |
| 25 | FOC4g06830 | HET domain protein |
| 68 | FOC4g06873 | Aspartyl-tRNA synthetase, cytoplasmic |
| 73 | FOC4g06878 | Sulfate permease |
| 75 | FOC4g06880 | MDR, Multidrug resistance-associated protein |
| 81 | FOC4g06889 | Myosin heavy chain kinase B |
| 86 | FOC4g06895 | aldehyde dehydrogenase |
| 89 | FOC4g06898 | unknown protein |
| 97 | FOC4g06906 | triose phosphate transporter |
| 98 | FOC4g06907 | Long-chain-fatty-acid--CoA ligase 1, adenylate forming domain |
| 01 | FOC4g06910 | [illegible] reductase |
| 04 | FOC4g06913 | AA9 copper-dependent polysaccharide monooxygenase |
| 05 | FOC4g06914 | AB hydrolase |
| 24 | FOC4g06933 | AB hydrolase |
| 27 | FOC4g06936 | unknown protein |
| 45 | FOC4g06954 | amino acid permease |
| 54 | FOC4g06963 | unknown protein |
| 86 | FOC4g06997 | FAD/FMN-dependent oxidoreductase |
| 89 | FOC4g07000 | Vacuolar iron transporter |
| 01 | FOC4g07013 | 5-methylthioadenosine/S-adenosylhomocysteine deaminase |
| 03 | FOC4g07015 | unknown protein |
| 06 | FOC4g07018 | chloride channel, bestrophin family |
| 43 | FOC4g07058 | alpha/beta hydrolase |
| 73 | FOC4g03002 | 40S ribosomal protein S28 |
| 97 | FOC4g03027 | Cytochrome P450 52A13 |
| 63 | FOC4g03096 | Alternative oxidase, mitochondrial |
| 75 | FOC4g03108 | unknown protein |
| 87 | FOC4g03120 | unknown protein |
| 11 | FOC4g00497 | unknown protein |
| 36 | FOC4g00074 | unknown protein |
| 55 | FOC4g00035 | HET domain protein |
| 71 | FOC4g03406 | integral membrane protein |
| 01 | FOC4g03437 | short chain dehydrogenase/reductase |
| 10 | FOC4g03446 | unknown protein |
| 13 | FOC4g03449 | adenine nucleotide alpha hydrolase |

| | | |
|---|---|---|
| 21 | FOC4g03458 | MFS polyol transporter |
| 22 | FOC4g03459 | E3 ubiquitin-protein ligase HACE1 |
| 28 | FOC4g03466 | GNAT family N-acetyltransferase |
| 29 | FOC4g03467 | Aldehyde reductase |
| 35 | FOC4g03473 | unknown protein |
| 42 | FOC4g03480 | aminoglycoside phosphotransferase |
| 49 | FOC4g03487 | Na+/H+antiporter |
| 50 | FOC4g03488 | Na+/H+antiporter |
| 51 | FOC4g03490 | Sarcosine dehydrogenase, mitochondrial |
| 55 | FOC4g03494 | Sterol-4-alpha-carboxylate 3-dehydrogenase, decarboxylating |
| 56 | FOC4g03495 | unknown protein |
| 57 | FOC4g03496 | A1 aspartyl pepidase |
| 63 | FOC4g03502 | unknown protein |
| 70 | FOC4g03509 | RNAse H1 |
| 72 | FOC4g03511 | Arylamine N-acetyltransferase, pineal gland isozyme NAT-10 |
| 79 | FOC4g03518 | D-amino acid oxidase |
| 87 | FOC4g03527 | Zinc-dependent medium chain dehydrogenase/reductase |
| 22 | FOC4g03563 | agmatinase |
| 33 | FOC4g03576 | galactonate dehydratase |
| 34 | FOC4g03577 | unknown protein |
| 44 | FOC4g03588 | MFS alpha-glucoside permease |
| 47 | FOC4g03591 | pantothenate kinase CAB1 |
| 73 | FOC4g03618 | E3 ubiquitin ligase |
| 77 | FOC4g03623 | amino acid permease GAP1 |
| 80 | FOC4g03626 | non-LTR RNAse |
| 93 | FOC4g03639 | Old yellow enyzme, FMN/NADPH-dependent reductase |
| 01 | FOC4g03647 | unknown protein |
| 02 | FOC4g03648 | Zn2Cys6 transcriptional activator |
| 03 | FOC4g03649 | short-chain dehydrogenase/reductase |
| 04 | FOC4g03650 | unknown protein with PLAC8 domain |
| 06 | FOC4g03652 | Malic acid transport protein |
| 09 | FOC4g03655 | Sulfite oxidase |
| 10 | FOC4g03656 | peroxisomal catalase |
| 11 | FOC4g03657 | transposase of the activator superfamily |
| 16 | FOC4g03662 | L-ascorbate oxidase |
| 26 | FOC4g03672 | GH16 endo-ß-1,3/1.4-glucanase |

| | | |
|---|---|---|
| 28 | FOC4g03674 | unknown protein |
| 32 | FOC4g03678 | Cytochrome P450 sterigmatocystin biosynthesis monooxygenase |
| 43 | FOC4g03689 | NRPS, lovastatin nonaketide synthase |
| 45 | FOC4g03691 | Zinc-dependent alcohol dehydrogenase |
| 47 | FOC4g03693 | MFS permease |
| 48 | FOC4g03694 | Cytochrome P450 monooxygenase |
| 50 | FOC4g03696 | unknown protein |
| 72 | FOC4g03720 | FAD/FMN-dependent oxidoreductase |
| 77 | FOC4g05353 | unknown protein |
| 83 | FOC4g05359 | Zn2Cys6 transcriptional activator |
| 86 | FOC4g05362 | MFS permease |
| 27 | FOC4g05411 | cupin domain protein |
| 31 | FOC4g05417 | unknown protein with bacterial sortase domain |
| 63 | FOC4g05452 | unknown protein |
| 65 | FOC4g05454 | Pyridine nucleotide-disulphide oxidoreductase, |
| 74 | FOC4g05463 | GH12 xyloglucan-specific endo-beta-1,4-glucanase A |
| 75 | FOC4g05464 | epoxide hydrolase |
| 77 | FOC4g05466 | 2-enoyl-thioester reductase |
| 83 | FOC4g05472 | heme-binding peroxidase |
| 02 | FOC4g05491 | unknown protein |
| 25 | FOC4g05515 | short chain dehydrogenase/reductase |
| 41 | FOC4g05531 | HET domain protein |
| 67 | FOC4g05558 | unknown protein |
| 77 | FOC4g05569 | Cytochrome P450 monooxygenase |
| 86 | FOC4g05579 | Metallocarboxypeptidase A |
| 96 | FOC4g05590 | unknown protein |
| 03 | FOC4g05597 | PL1 pectate lyase plus CBM1 |
| 10 | FOC4g05604 | unknown protein |
| 11 | FOC4g05605 | UbiA prenyltransferase |
| 12 | FOC4g05606 | Cytochrome P450 monooxygenase |
| 13 | FOC4g05607 | Epoxide hydrolase |
| 14 | FOC4g05608 | Tryptophan dimethylallyltransferase |
| 16 | FOC4g05611 | X-Pro dipeptidyl peptidase (S15 family)/X-Pro dipeptidyl peptidase C terminal non-catalytic domain |
| 19 | FOC4g05614 | MFS permease |
| 20 | FOC4g05615 | aldehyde dehydrogenase |
| 26 | FOC4g05622 | unknown protein |

| | | |
|---|---|---|
| 29 | FOC4g05625 | Alcohol dehydrogenase |
| 30 | FOC4g05626 | Disintegrin and metalloproteinase domain-containing protein |
| 35 | FOC4g05633 | AA2, FAD-dependent choline dehydrogenase |
| 38 | FOC4g05637 | Zn2Cys6 Oleate activated transcription factor 3 |
| 39 | FOC4g05638 | Ceramide very long chain fatty acid hydroxylase SCS7 |
| 45 | FOC4g05644 | CFEM domain protein |
| 53 | FOC4g05653 | orphan protein |
| 57 | FOC4g05657 | Fumarylacetoacetase |
| 63 | FOC4g05664 | unknown protein |
| 64 | FOC4g05665 | MDR multidrug transporter |
| 81 | FOC4g05683 | AB hydrolase superfamily protein yisY |
| 82 | FOC4g05684 | Zn2Cys6 transcriptional regulator |
| 91 | FOC4g05693 | unknown protein |
| 96 | FOC4g05698 | FAD-dependent monooxygenase |
| 02 | FOC4g05704 | unknown protein |
| 20 | FOC4g05723 | Zn2Cys6 transcriptional activator |
| 29 | FOC4g05734 | MFS, Maltose permease MAL31 |
| 42 | FOC4g05747 | unknown protein |
| 45 | FOC4g05750 | oxalate decarboxylase oxdC |
| 46 | FOC4g05751 | Zn2Cys6 transcriptional regulator |
| 47 | FOC4g05753 | sulfate permease |
| 48 | FOC4g05754 | cerato-platanin |
| 58 | FOC4g05765 | GH74 xyloglucanase |
| 74 | FOC4g05781 | SAM-dependent methyltransferase |
| 91 | FOC4g05799 | Aspartate aminotransferase, cytoplasmic |
| 92 | FOC4g05800 | [illegible] tetrahydroxynaphthalene/trihydroxynaphthalene reductase |
| 94 | FOC4g05802 | MFS sugar phosphate permease |
| 99 | FOC4g05807 | Pimeloyl-ACP methyl ester carboxylesterase |
| 02 | FOC4g05810 | short-chain dehydrogenase/reductase |
| 11 | FOC4g05819 | DNA repair helicase ercc3 |
| 12 | FOC4g05820 | Cholinesterase |
| 13 | FOC4g05821 | unknown protein |
| 16 | FOC4g05824 | M35 metallopeptidase, deuterolysin |
| 22 | FOC4g05830 | unknown protein |
| 26 | FOC4g05834 | short chain dehydrogenase/reductase |
| 29 | FOC4g05837 | unknown protein |

| | | |
|---|---|---|
| 37 | FOC4g05845 | GH5 endo-ß-1,4-glucanase plus CBM1 |
| 43 | FOC4g05852 | Glutathione S-transferase |
| 81 | FOC4g05890 | cerato-platanin |
| 89 | FOC4g05898 | oxysterol-bindung protein |
| 06 | FOC4g05919 | GH12 endo-ß-1,4-glucanase |
| 09 | FOC4g05922 | MFS glucose transporter rco-3 |
| 60 | FOC4g00669 | agmatine deiminase |
| 75 | FOC4g00684 | AB hydrolase |
| 09 | FOC4g12305 | Amine oxidase, membrane-bound |
| 16 | FOC4g12312 | Zinc-dependent medium chain dehydrogenase/reductase |
| 26 | FOC4g12322 | FAD/FMN-dependent oxidoreductase |
| 30 | FOC4g12326 | Aldehyde dehydrogenase |
| 51 | FOC4g12347 | retinol dehydrogenase |
| 61 | FOC4g12357 | 2OG (FeII)- homogentisate 1,2-dioxygenase |
| 66 | FOC4g12362 | Demethylmenaquinone methyltransferase |
| 68 | FOC4g12364 | Zn2Cys6 transcriptional activator |
| 73 | FOC4g12369 | Nitrile-specifier protein |
| 76 | FOC4g12372 | unknown protein |
| 87 | FOC4g12383 | unknown protein with similarity to retinal |
| 88 | FOC4g12384 | GH28 endopolygalacturonase |
| 90 | FOC4g12386 | unknown protein |
| 92 | FOC4g12388 | S8 peptidase |
| 93 | FOC4g12389 | Ankyrin |
| 04 | FOC4g12403 | Zn2Cys6 transcriptional activator |
| 08 | FOC4g12408 | GH12 xyloglucan-specific endo-beta-1,4-glucanase |
| 71 | FOC4g12477 | non-LTR RNAse |
| 78 | FOC4g12483 | 60S ribosomal protein L38 |
| 79 | FOC4g12484 | GH28 endopolygalacturonase |
| 89 | FOC4g12494 | unknown protein with hAT dimerization domain |
| 27 | FOC4g12535 | Cytochrome b2, mitochondrial |
| 29 | FOC4g12537 | Cocaine esterase |
| 51 | FOC4g12559 | glutathione-dependent formaldehyde activating enzyme |
| 53 | FOC4g12563 | Cytochrome P450 Cholesterol 7-alpha-monooxygenase |
| 54 | FOC4g12564 | oxysterol binding protein |
| 56 | FOC4g12566 | unknown protein |
| 65 | FOC4g12576 | amino acid permease GABA |

| | | |
|---|---|---|
| 66 | FOC4g12577 | RTA1 transmembrane protein; binds extracellular toxins |
| 69 | FOC4g12580 | unknown protein |
| 78 | FOC4g12589 | unknown protein |
| 50 | FOC4g12663 | ATP synthase subunit 9, mitochondrial |
| 79 | FOC4g12691 | Cytochrome P450 monooxygense, Isotrichodermin C-15 hydroxylase |
| 88 | FOC4g12701 | Cytosceletal protein, putative |
| 04 | FOC4g12718 | polyketide cyclase |
| 11 | FOC4g12725 | Argininosuccinate synthase, partial |
| 49 | FOC4g12765 | polyketide cyclase |
| 75 | FOC4g12792 | peroxiredoxin HYR1 |
| 15 | FOC4g12831 | Zn2Cys6 transcriptional activator |
| 22 | FOC4g12838 | Ceramide very long chain fatty acid hydroxylase SCS7 |
| 33 | FOC4g12849 | CFEM domain protein |
| 60 | FOC4g12877 | unknown protein |
| 69 | FOC4g12886 | MFS quinate permease |
| 89 | FOC4g12906 | MDR multidrug transporter; Leptomycin B resistance protein |
| 50 | FOC4g12966 | GH13 Glucoamylase |
| 67 | FOC4g12984 | Serine hydroxymethyltransferase, cytosolic |
| 08 | FOC4g13027 | Zn2Cys6 transcriptional activator, sterol uptake control |
| 22 | FOC4g13041 | monocarboxylate transporter |
| 81 | FOC4g13103 | FAD-linked sulfhydryl oxidase ALR |
| 33 | FOC4g00158 | unknown protein |
| 41 | FOC4g04825 | unknown protein |
| 43 | FOC4g04827 | [illegible] reductase |
| 75 | FOC4g04861 | unknown protein |
| 76 | FOC4g04862 | unknown protein with transferase domain |
| 80 | FOC4g04867 | Pisatin demethylase |
| 81 | FOC4g04868 | SRPBCC lipid binding protein |
| 92 | FOC4g04880 | glutamine sythase |
| 22 | FOC4g04910 | short chain dehydrogenase/reductase |
| 24 | FOC4g04912 | HET domain protein |
| 35 | FOC4g04924 | Carboxylesterase family |
| 48 | FOC4g04939 | short chain dehydrogenase/reductase |
| 64 | FOC4g04956 | amino acid permease |
| 73 | FOC4g04965 | monocarboxylate permease |
| 92 | FOC4g04987 | Flavin-dependent amine oxidoreductase |

| | | |
|---|---|---|
| 02 | FOC4g04997 | Phytoene desaturase |
| 03 | FOC4g04998 | Bifunctional lycopene cyclase/phytoene synthase |
| 12 | FOC4g05007 | Aspartate aminotransferase, cytoplasmic |
| 28 | FOC4g05024 | UbiA family prenyltransferase |
| 29 | FOC4g05025 | chloride channel, bestrophin family |
| 44 | FOC4g05041 | lipoprotein, secreted |
| 55 | FOC4g05052 | unknown protein |
| 89 | FOC4g05086 | GH5 Glycoside Hydrolase Family 76 |
| 95 | FOC4g05093 | Cytochrome P450 monooxygenase |
| 96 | FOC4g05094 | unknown protein with WD repeats |
| 97 | FOC4g05095 | Cytochrome P450 4F12 |
| 99 | FOC4g05097 | Presilphiperfolan-8-beta-ol synthase |
| 02 | FOC4g05100 | Spore wall maturation protein DIT1 |
| 03 | FOC4g05101 | Cytochrome P450 trichodiene oxygenase |
| 13 | FOC4g05111 | ornithin cyclodeaminase |
| 15 | FOC4g05113 | unknown protein |
| 17 | FOC4g05115 | GPR1/FUN34/yaaH plasma membrane protein |
| 28 | FOC4g05126 | Phototropin-2 |
| 29 | FOC4g05127 | orphan protein |
| 44 | FOC4g05143 | unknown protein |
| 46 | FOC4g05145 | Old yellow enyzme, FMN/NADPH-dependent reductase |
| 55 | FOC4g05154 | unknown protein, secreted; maybe cell wall protein |
| 56 | FOC4g05155 | Cytochrome p450 protein |
| 58 | FOC4g05157 | Lipid phosphate phosphatase 1 |
| 59 | FOC4g05158 | Pimeloyl-ACP-acyl ester carboxyesterase |
| 65 | FOC4g05166 | short chain dehydrogenase/reductase |
| 66 | FOC4g05167 | Glutamate carboxypeptidase |
| 71 | FOC4g05172 | urea active transporter |
| 74 | FOC4g05175 | pathogenesis-related protein 1C |
| 81 | FOC4g05182 | LysM protein |
| 82 | FOC4g05183 | oxalate decarboxylase oxdC |
| 83 | FOC4g05184 | unknown protein with DUF1275 domain protein |
| 85 | FOC4g05186 | sulfate permease SutB |
| 89 | FOC4g05190 | short chain dehydrogenase/reductase |
| 91 | FOC4g05192 | Zinc finger protein zas1 |
| 99 | FOC4g05200 | unknown protein, weak similarity to sortilin |

| | | |
|---|---|---|
| 03 | FOC4g05205 | sulfatase |
| 12 | FOC4g05214 | MFS permease |
| 16 | FOC4g05218 | MFS Lactose permease |
| 18 | FOC4g05220 | CE8 pectin methylesterase |
| 21 | FOC4g05223 | short chain dehydrogenase/reductase |
| 23 | FOC4g05225 | [illegible] cyclohexadiene-1,4-diol dehydrogenase |
| 27 | FOC4g05229 | Amino-acid permease inda1 |
| 28 | FOC4g05230 | Zinc-dependent alcohol dehydrogenase |
| 29 | FOC4g05231 | Dimethylglycine dehydrogenase, mitochondrial |
| 38 | FOC4g05240 | Cytochrome P450 55A1 |
| 39 | FOC4g05242 | phenolic acid decarboxylase padC |
| 40 | FOC4g05243 | unknown protein |
| 48 | FOC4g05252 | unknown protein |
| 52 | FOC4g05256 | Acid phosphatase |
| 61 | FOC4g05266 | unknown protein |
| 64 | FOC4g05269 | Zeaxanthin epoxidase, chloroplastic |
| 74 | FOC4g05279 | unknown protein |
| 75 | FOC4g05280 | GH12 endo-ß-1,4-glucanase |
| 76 | FOC4g05281 | haloalkanoic acid dehalogenase |
| 77 | FOC4g05282 | PKS, granaticin polyketide synthase |
| 80 | FOC4g05285 | unknown protein |
| 85 | FOC4g05290 | AB domain hydrolase |
| 89 | FOC4g05294 | AA2, FAD-dependent choline dehydrogenase |
| 92 | FOC4g05297 | Cytochrome P450 trichodiene oxygenase |
| 41 | FOC4g00290 | PIM1, ATP-dependent serine endopeptidase reacting to heat shock |
| 56 | FOC4g00241 | amino-acid permease P7G5.06 |
| 63 | FOC4g00575 | unknown protein with hAT dimerization domain |
| 64 | FOC4g00576 | unknown protein with hAT dimerization domain |
| 96 | FOC4g01246 | unknown protein |
| 00 | FOC4g01250 | orphan protein |
| 07 | FOC4g01258 | unknown protein with hAT dimerization domain |
| 13 | FOC4g01266 | GH28 endopolygalacturonase |
| 18 | FOC4g01272 | NCS1 permease for cytosine/purines, uracil, thiamine, allantoin |
| 22 | FOC4g01276 | FAD-dependent 3-hydroxybenzoate 6-monooxygenase |
| 23 | FOC4g01277 | SAM-dependent methyltransferase |
| 24 | FOC4g01278 | Zn2Cys6 transcriptional regulator |

| | | |
|---|---|---|
| 25 | FOC4g01279 | phosphate carrier, mitochondrial |
| 26 | FOC4g01280 | Glycerol-3-phosphate dehydrogenase, mitochondrial |
| 65 | FOC4g01319 | MFS permease |
| 69 | FOC4g01323 | Zn2Cys6 transcriptional activator |
| 73 | FOC4g01327 | 2OG-Fe(II) dioxygenase |
| 24 | FOC4g03146 | Long-chain-fatty-acid--CoA ligase 1 |
| 29 | FOC4g03151 | unknown protein |
| 36 | FOC4g03158 | Polyamine oxidase |
| 39 | FOC4g03161 | cyanoviridin N |
| 44 | FOC4g03166 | phospholipase A2 subgroup |
| 45 | FOC4g03167 | unknown protein |
| 47 | FOC4g03170 | 2OG-Fe(II) dioxygenase |
| 61 | FOC4g03185 | C2H2 transcriptional activator, uncertain |
| 72 | FOC4g03196 | amino acid permease |
| 86 | FOC4g03211 | Mannose-specific lectin |
| 92 | FOC4g03217 | Cyanamide hydratase |
| 98 | FOC4g03224 | [illegible] protein |
| 99 | FOC4g03225 | FAD/FMN-dependent oxidoreductase |
| 13 | FOC4g03241 | unknown protein |
| 16 | FOC4g03246 | Trans-2-enoyl-CoA reductase, mitochondrial |
| 17 | FOC4g03247 | 17ß-hydroxysteroid dehydrogenase |
| 24 | FOC4g03254 | GH5 endo-ß-1,4-glucanase plus CBM1 |
| 29 | FOC4g03259 | polyphenol oxidase, tyrosinase |
| 39 | FOC4g03269 | HET domain protein |
| 49 | FOC4g03280 | GNAT family N-acetyltransferase |
| 53 | FOC4g03284 | Enoyl-[acyl-carrier-protein] reductase |
| 54 | FOC4g03285 | Endoglycoceramidase |
| 72 | FOC4g03303 | GH13 Alpha-amylase A type-1/2 |
| 74 | FOC4g03305 | chromate transporter |
| 76 | FOC4g03307 | 2OG-Fe(II) sulfonate dioxygenase |
| 77 | FOC4g03308 | Nucleosome assembly protein |
| 83 | FOC4g03314 | glutathione S-transferase |
| 90 | FOC4g03321 | unknown protein |
| 94 | FOC4g03326 | unknown protein |
| 95 | FOC4g03327 | OPT oligopeptide transporter |
| 96 | FOC4g03328 | FAD-dependent 3-hydroxybenzoate 6-monooxygenase |

| | | |
|---|---|---|
| 19 | FOC4g03351 | unknown protein |
| 24 | FOC4g03358 | MFS drug efflux transporter |
| 26 | FOC4g03360 | 1,3-propanediol dehydrogenase |
| 32 | FOC4g03366 | 5-methylthioadenosine/S-adenosylhomocysteine deaminase |
| 36 | FOC4g03370 | unknown protein |
| 37 | FOC4g03371 | 2OG-Fe(II) dioxygenase |
| 38 | FOC4g03372 | ABC transporter |
| 03 | FOC4g00982 | unknown protein |
| 09 | FOC4g00989 | unknown protein |
| 35 | FOC4g00861 | 2-succinyl-6-hydroxy-2,4-cyclohexadiene-1-carboxylate synthase |
| 39 | FOC4g00865 | Zn2Cys6 transcriptional activator |
| 40 | FOC4g00866 | unknown protein |
| 81 | FOC4g00907 | orphan protein |
| 84 | FOC4g00114 | unknown protein |
| 85 | FOC4g00115 | [illegible] reductase |
| 30 | FOC4g08267 | NADP-dependent alcohol dehydrogenase 6 |
| 39 | FOC4g08276 | unknown protein |
| 90 | FOC4g08330 | DDE superfamily endonuclease |
| 15 | FOC4g08356 | unknown protein |
| 16 | FOC4g08357 | retinol dehydrogenase |
| 17 | FOC4g08358 | unknown protein |
| 37 | FOC4g08378 | unknown protein |
| 40 | FOC4g08381 | GH3 ß-glycosidase |
| 58 | FOC4g08398 | cyanoviridin N |
| 02 | FOC4g08442 | Ankyrin |
| 15 | FOC4g08455 | Nitrogen assimilation transcription factor nirA |
| 13 | FOC4g08557 | Carboxylesterase |
| 20 | FOC4g08564 | transposase of the activator superfamily |
| 34 | FOC4g08579 | peroxisomal NADH pyrophosphatase NUDT12 |
| 25 | FOC4g08671 | 40S ribosomal protein S20 |
| 58 | FOC4g08704 | A1 aspartyl pepidase |
| 59 | FOC4g08705 | V-type proton ATPase 16 kDa proteolipid subunit |
| 72 | FOC4g08718 | unknown protein |
| 80 | FOC4g08726 | Mercuric reductase |
| 88 | FOC4g08734 | unknown protein |
| 68 | FOC4g08816 | 60S ribosomal protein L29, partial |

| | | |
|---|---|---|
| 77 | FOC4g08822 | Zn2Cys6 transcriptional activator |
| 94 | FOC4g08840 | RTA1 transmembrane protein; binds extracellular toxins |
| 99 | FOC4g08846 | GH43 ß-xylosidase/alpha-arabinosidase |
| 16 | FOC4g08865 | [illegible] protein/coenzyme A (ACP/CoA) to acyl-(ACP/CoA) |
| 23 | FOC4g08872 | unknown protein |
| 38 | FOC4g08888 | orphan protein |
| 39 | FOC4g08889 | NAD(P)H dependent FMN reductase |
| 48 | FOC4g08898 | reverse transcriptase |
| 54 | FOC4g08904 | Betaine aldehyde dehydrogenase |
| 55 | FOC4g08905 | Reticulon-4-interacting protein 1 like protein, mitochondrial |
| 70 | FOC4g08929 | short-chain dehydrogenase/reductase |
| 71 | FOC4g08930 | PL1 pectate lyase |
| 79 | FOC4g08938 | Enoyl-CoA hydratase 2, peroxisomal |
| 82 | FOC4g08941 | HET domain protein |
| 96 | FOC4g08957 | unknown protein with DUF4237 |
| 97 | FOC4g08958 | aldehyde reductase |
| 98 | FOC4g08959 | Enoyl-CoA hydratase domain-containing protein 2, mitochondrial |
| 99 | FOC4g08960 | Dihydroflavonol-4-reductase |
| 30 | FOC4g08993 | short-chain dehydrogenase/reductase |
| 33 | FOC4g08996 | ABC transporter CDR4 |
| 34 | FOC4g08997 | unknown protein |
| 46 | FOC4g09009 | unknown protein |
| 47 | FOC4g09010 | NADH oxidase |
| 68 | FOC4g09031 | GH16 endo-ß-1,3/1.4-glucanase |
| 76 | FOC4g09040 | 2OG (FeII)- Indoleamine 2,3-dioxygenase family protein |
| 92 | FOC4g09057 | unknown protein with DUF427 domain |
| 93 | FOC4g09058 | cupin domain protein |
| 00 | FOC4g09065 | MFS-multidrug-resistance transporter |
| 42 | FOC4g09109 | Glutamine Amidotransferase PDX2 involved in pyridoxal biosynthesis |
| 72 | FOC4g09241 | 40S ribosomal protein S19 |
| 73 | FOC4g09242 | Zinc-regulated transporter 2 |
| 92 | FOC4g09262 | NADH dehydrogenase |
| 16 | FOC4g09287 | Lipase |
| 21 | FOC4g09292 | Mitochondrial intermembrane space import and assembly protein 40 |
| 46 | FOC4g09319 | unknown protein |
| 49 | FOC4g09322 | Cytochrome P450 monooxygenase |

| | | |
|---|---|---|
| 53 | FOC4g09326 | Ribonuclease Trv |
| 60 | FOC4g09333 | 1-aminocyclopropane-1-carboxylate deaminase |
| 61 | FOC4g09334 | unknown protein |
| 62 | FOC4g09335 | di/tripeptide transporter PTR2 |
| 63 | FOC4g09336 | Initiation-specific alpha-1,6-mannosyltransferase |
| 89 | FOC4g09363 | WD domain, G-beta repeat |
| 15 | FOC4g09390 | unknown protein |
| 16 | FOC4g09391 | unknown protein |
| 32 | FOC4g09407 | unknown protein |
| 43 | FOC4g09419 | Cell wall protein PRY3 |
| 48 | FOC4g09424 | bZIP transcription factor |
| 51 | FOC4g09427 | MFS multidrug transporter |
| 05 | FOC4g09484 | Zinc-dependent alcohol dehydrogenase |
| 10 | FOC4g09489 | unknown protein with hAT dimerization domain |
| 12 | FOC4g09491 | SH3 domain-containing YSC84-like protein 1 |
| 16 | FOC4g09495 | Nitrogen assimilation transcription factor nirA |
| 18 | FOC4g09497 | unknown protein |
| 20 | FOC4g09499 | [illegible] subunit A |
| 28 | FOC4g09507 | aminoglycoside phosphotransferase, putative |
| 29 | FOC4g09508 | Zn2Cys6 transcriptional activator |
| 35 | FOC4g09514 | AB hydrolase |
| 36 | FOC4g09515 | amino acyl tRNA ligase |
| 39 | FOC4g09518 | galactose oxidase with discoidin andKelch domain |
| 41 | FOC4g09520 | MFS drug efflux transporter |
| 50 | FOC4g09529 | unknown protein |
| 54 | FOC4g09533 | GH18 chitinase |
| 55 | FOC4g09534 | unknown protein |
| 74 | FOC4g09560 | Cytochrome P450 monooxygenase |
| 93 | FOC4g09583 | retinol dehydrogenase |
| 94 | FOC4g09584 | unknown protein |
| 97 | FOC4g09587 | Zinc-dependent alcohol dehydrogenase |
| 98 | FOC4g09588 | NmrA-like transcription repressor |
| 01 | FOC4g09591 | integral membrane protein |
| 02 | FOC4g09592 | tyrosine protein kinase |
| 04 | FOC4g09595 | Vacuolar calcium ion transporter |
| 12 | FOC4g09603 | GH6 cellobiohydrolase 2 |

| | | |
|---|---|---|
| [illegible] 16 | FOC4g09607 | AA9 copper-dependent polysaccharide monooxygenase |
| [illegible] 21 | FOC4g09612 | unknown protein |
| [illegible] 29 | FOC4g09620 | GH28 endo-polygalacturonase |
| [illegible] 30 | FOC4g09621 | Malic acid transport protein |
| [illegible] 38 | FOC4g09631 | PL8 pectate lyase |
| [illegible] 45 | FOC4g09641 | unknown protein |
| [illegible] 49 | FOC4g09646 | MFS inositol transporter |
| [illegible] 57 | FOC4g09654 | Cytochrome P450 61 |
| [illegible] 61 | FOC4g09658 | unknown protein |
| [illegible] 62 | FOC4g09659 | enoyl-(acyl carrier protein) reductase; |
| [illegible] 63 | FOC4g09660 | unknown protein |
| [illegible] 66 | FOC4g09663 | Zinc-dependent alcohol dehydrogenase |
| [illegible] 78 | FOC4g09675 | Zn2Cys6 transcriptional activator |
| [illegible] 79 | FOC4g09676 | short chain dehydrogenase/reductase |
| [illegible] 80 | FOC4g09677 | transmembrane protein, function unknown |
| [illegible] 83 | FOC4g09682 | flavodoxin |
| [illegible] 86 | FOC4g09685 | cytochrome P450-Salicylate hydroxylase |
| [illegible] 88 | FOC4g09686 | Pyruvate decarboxylase |
| [illegible] 96 | FOC4g09694 | peroxidase family 3 |
| [illegible] 16 | FOC4g09714 | NADH oxidase |
| [illegible] 21 | FOC4g09719 | MFS quinate permease |
| [illegible] 29 | FOC4g09728 | unknown protein |
| [illegible] 38 | FOC4g09737 | NmrA-like transcription repressor |
| [illegible] 42 | FOC4g09741 | unknown protein |
| [illegible] 50 | FOC4g09749 | unknown protein |
| [illegible] 56 | FOC4g09755 | VipA, FAR1-domain zinc-binding DNA-binding |
| [illegible] 63 | FOC4g09762 | siderophore esterase IroE |
| [illegible] 65 | FOC4g09764 | Acyl-CoA N-acyltransferase; Siderophore biosynthesis |
| [illegible] 73 | FOC4g09772 | orphan protein |
| [illegible] 80 | FOC4g09778 | Dyp-type peroxidase |
| [illegible] 83 | FOC4g09781 | unknown protein |
| [illegible] 90 | FOC4g09788 | Copper-containing nitrite reductase |
| [illegible] 91 | FOC4g09789 | Endoplasmatic reticulum oxidising protein Ero1 |
| [illegible] 92 | FOC4g09790 | unknown protein |
| [illegible] 99 | FOC4g09797 | peptide transporter PTR2 |
| [illegible] 01 | FOC4g09799 | unknown protein |

| | | |
|---|---|---|
| 17 | FOC4g09815 | unknown protein |
| 30 | FOC4g09828 | NEDD4-like E3 ubiquitin-protein ligase WWP2 |
| 51 | FOC4g09849 | unknown protein |
| 64 | FOC4g09863 | unknown protein |
| 75 | FOC4g09874 | NmrA-like transcription repressor |
| 78 | FOC4g09877 | Zn2Cys6 transcriptional activator |
| 83 | FOC4g09882 | Histone-lysine N-methyltransferase |
| 84 | FOC4g09883 | unknown protein with hAT dimerization domain |
| 89 | FOC4g09888 | Zn2Cys6 transcriptional regulator |
| 90 | FOC4g09889 | Alcohol dehydrogenase |
| 91 | FOC4g09890 | gamma-glutamyl cyclotransferase |
| 92 | FOC4g09891 | amidoligase family 2 |
| 99 | FOC4g09898 | unknown protein |
| 14 | FOC4g09914 | GH32 invertase |
| 28 | FOC4g09928 | unknown protein |
| 41 | FOC4g09941 | RNAse H1 |
| 56 | FOC4g09957 | unknown protein |
| 64 | FOC4g09965 | phosphoglycerate mutase family protein |
| 71 | FOC4g09972 | Sulfide:quinone oxidoreductase, mitochondrial |
| 73 | FOC4g09975 | Pisatin demethylase |
| 89 | FOC4g09992 | NADP-dependent oxidoreductase yfmJ |
| 02 | FOC4g10006 | unknown protein |
| 09 | FOC4g10014 | L-lactate dehydrogenase A |
| 32 | FOC4g10037 | Serine/threonine-protein kinase SRPK |
| 33 | FOC4g10038 | Vacuolar calcium ion transporter |
| 38 | FOC4g10043 | unknown protein |
| 51 | FOC4g10056 | GH5 Glycoside Hydrolase Family 5 / Subf 15 |
| 55 | FOC4g10060 | MFS Major facilitator superfamily domain-containing protein 1 |
| 61 | FOC4g10066 | orphan protein |
| 70 | FOC4g10076 | nucleotide sugar transferase, involved in polysaccharide biosynthesis |
| 72 | FOC4g10078 | unknown protein |
| 75 | FOC4g10081 | MFS inositol transporter 1 |
| 76 | FOC4g10082 | Zinc-dependent alcohol dehydrogenase |
| 77 | FOC4g10083 | RNAse H1 |
| 78 | FOC4g10084 | Cytochrome P450 monooxygense, Isotrichodermin C-15 hydroxylase |
| 79 | FOC4g10085 | unknown protein |

| | | |
|---|---|---|
| 85 | FOC4g10093 | short chain dehydrogenase/reductase |
| 86 | FOC4g10094 | unknown protein |
| 89 | FOC4g10097 | Cytosolic phospholipase A2 |
| 91 | FOC4g10100 | unknown protein |
| 00 | FOC4g10111 | HET domain protein |
| 04 | FOC4g10115 | unknown protein |
| 16 | FOC4g10129 | formate transporter |
| 65 | FOC4g10179 | MFS Lactose permease |
| 88 | FOC4g10202 | Pyruvate decarboxylase |
| 90 | FOC4g10204 | HLH domain DNA binding protein |
| 91 | FOC4g10206 | NACHT domain protein, possibly involved in apoptosis |
| 08 | FOC4g10223 | Zinc-dependent alcohol dehydrogenase |
| 35 | FOC4g10251 | Glutamate decarboxylase |
| 42 | FOC4g10259 | Zn2Cys6 transcriptional activator |
| 45 | FOC4g10262 | Trichothecene 3-O-acetyltransferase |
| 47 | FOC4g10266 | unknown protein |
| 59 | FOC4g00433 | Glutamine Amidotransferase PDX2 involved in pyridoxal biosynthesis |
| 24 | FOC4g01413 | mRNA 3\'-end-processing protein YTH1 |
| 39 | FOC4g01428 | 17ß-hydroxysteroid dehydrogenase |
| 43 | FOC4g01429 | FAD/FMN-dependent oxidoreductase |
| 59 | FOC4g01449 | Endoplasmic oxidoreductin-1 |
| 63 | FOC4g01454 | GH3 ß-glycosidase |
| 74 | FOC4g01466 | orphan protein |
| 95 | FOC4g03738 | N-acetyltransferase YoaA |
| 08 | FOC4g03751 | AA9 copper-dependent polysaccharide monooxygenase |
| 16 | FOC4g03759 | Zinc-dependent medium chain dehydrogenase/reductase |
| 19 | FOC4g03762 | Pisatin demethylase |
| 31 | FOC4g03775 | N-acetyltransferase ats1 |
| 32 | FOC4g03776 | Zn2Cys6 transcriptional activator |
| 36 | FOC4g03780 | MFS quinate permease |
| 39 | FOC4g03783 | unknown protein |
| 42 | FOC4g03786 | NADP/FMN dependent dehydrogenase/reductase |
| 45 | FOC4g03789 | short-chain dehydrogenase/reductase |
| 53 | FOC4g03797 | C2H2 transcriptional activator |
| 55 | FOC4g03799 | orphan protein |
| 59 | FOC4g03804 | unknown protein |

| | | |
|---|---|---|
| 77 | FOC4g03822 | unknown protein |
| 78 | FOC4g03823 | alcohol dehydrogenase |
| 80 | FOC4g03825 | S8 subtilisin-like endopeptidase |
| 81 | FOC4g03826 | Aax Laccase |
| 86 | FOC4g03832 | Enoyl-[acyl-carrier-protein] reductase |
| 87 | FOC4g03833 | Calcium-independent phospholipase A2-gamma |
| 89 | FOC4g03835 | Calcium-independent phospholipase A2-gamma |
| 97 | FOC4g03843 | Aldehyde reductase 2 |
| 02 | FOC4g03847 | PKS/NRPS HYBRID |
| 03 | FOC4g03849 | Zinc-dependent alcohol dehydrogenase |
| 04 | FOC4g03850 | Cytochrome P450 monooxygenase |
| 07 | FOC4g03853 | Cytochrome P450 monooxygenase |
| 09 | FOC4g03855 | unknown protein |
| 11 | FOC4g03857 | Cytochrome P450 monooxygenase |
| 12 | FOC4g03858 | NADH oxidase |
| 22 | FOC4g03869 | GTP-binding protein |
| 40 | FOC4g03887 | FAD-dependent 2,4-dichlorophenol 6-monooxygenase |
| 57 | FOC4g03905 | Phosphoenolpyruvate synthase |
| 58 | FOC4g03906 | L-lactate dehydrogenase A |
| 72 | FOC4g03922 | unknown protein |
| 76 | FOC4g03926 | Aspartyl-tRNA ligase |
| 81 | FOC4g03931 | unknown protein involved in amino acid or purine biosynthesis |
| 82 | FOC4g03932 | unknown protein |
| 88 | FOC4g03938 | HET domain protein |
| 06 | FOC4g03959 | unknown protein |
| 10 | FOC4g03963 | 30 kDa heat shock protein |
| 12 | FOC4g03965 | Trehalose phosphorylase |
| 23 | FOC4g03976 | Quinone oxidoreductase PIG3 |
| 42 | FOC4g03998 | AB hydrolase |
| 45 | FOC4g04001 | purine and uridine phosphorylase |
| 48 | FOC4g04004 | unknown protein |
| 49 | FOC4g04005 | [illegible] terminal non-catalytic domain |
| 58 | FOC4g04014 | GNAT family N-acetyltransferase |
| 59 | FOC4g04015 | Acid phosphatase |
| 60 | FOC4g04016 | Beta-1,3-glucan-binding protein |
| 67 | FOC4g04023 | HET domain protein |

| | | |
|---|---|---|
| 85 | FOC4g04042 | unknown protein with Duf3425 domain |
| 91 | FOC4g04049 | unknown protein with hAT dimerization domain |
| 05 | FOC4g04066 | MFS aminotriazole efflux protein |
| 36 | FOC4g01685 | Salicylaldehyde dehydrogenase |
| 87 | FOC4g01738 | unknown protein |
| 96 | FOC4g01747 | Trichothecene 3-O-acetyltransferase |
| 99 | FOC4g01750 | Carbonic anhydrase |
| 01 | FOC4g01752 | high affinity ADP-ribose binding protein |
| 57 | FOC4g01807 | MFS alpha-glucosides permease MPH2 |
| 69 | FOC4g00441 | polyphenol oxidase, tyrosinase |
| 90 | FOC4g00332 | orphan protein |
| 24 | FOC4g01573 | GH95 alpha-L-fucosidase 2 |
| 31 | FOC4g01581 | unknown protein |
| 35 | FOC4g01585 | ATP-dependent RNA helicase DBP5 |
| 36 | FOC4g01586 | ATP-dependent RNA helicase |
| 42 | FOC4g01593 | Flavohemoprotein |
| 64 | FOC4g01615 | fasciclin-domain adhesion protein |
| 66 | FOC4g01617 | Aromatic amino acid aminotransferase |
| 84 | FOC4g01637 | RTA1 transmembrane protein; binds extracellular toxins |
| 85 | FOC4g01638 | unknown protein |
| 94 | FOC4g01647 | unknown protein with hAT dimerization domain |
| 98 | FOC4g01651 | Zn2Cys6 transcriptional activator |
| 15 | FOC4g01669 | DNA transposase |
| 17 | FOC4g01671 | unknown protein |
| 19 | FOC4g01673 | unknown protein |
| 41 | FOC4g01176 | Cytochrome c oxidase copper chaperone |
| 42 | FOC4g01177 | GH7 Cellobiohydrolase CBH1/CEL7a, CBM1 |
| 59 | FOC4g01195 | Molybdenum cofactor sulfurase |
| 72 | FOC4g01208 | flavin-containing monooxygenase |
| 98 | FOC4g01236 | Leucine aminopeptidase 1 |
| 25 | FOC4g04098 | short chain dehydrogenase/reductase; phenylcoumaran benzylic ether reductase |
| 78 | FOC4g04154 | Inorganic phosphate transporter |
| 79 | FOC4g04155 | Cytochrome P450 sterigmatocystin biosynthesis monooxygenase |
| 81 | FOC4g04157 | heme-dependent peroxidase(linoleate diol synthase ?) |
| 88 | FOC4g04164 | transposase IS4 |
| 89 | FOC4g04165 | Zn2Cys6 transcriptional activator |

| | | |
|---|---|---|
| 90 | FOC4g04166 | FMN-dependent dehydrogenase; Alpha-hydroxy acid dehydrogenase |
| 92 | FOC4g04168 | O-acetylhomoserine/O-acetylserine sulfhydrylase |
| 94 | FOC4g04170 | Zinc-dependent alcohol dehydrogenase |
| 95 | FOC4g04171 | Zinc-dependent alcohol dehydrogenase |
| 26 | FOC4g04203 | Zinc-dependent medium chain dehydrogenase/reductase |
| 27 | FOC4g04204 | endonuclease/Exonuclease/phosphatase |
| 29 | FOC4g04206 | unknown protein |
| 30 | FOC4g04207 | unknown protein |
| 32 | FOC4g04209 | MFS permease |
| 36 | FOC4g04214 | Zn2Cys6 transcriptional regulator |
| 39 | FOC4g04217 | glyoxalase I |
| 43 | FOC4g04222 | MFS permease |
| 58 | FOC4g04237 | orphan protein |
| 81 | FOC4g04263 | unknown protein |
| 82 | FOC4g04264 | 2-amino-3-carboxymuconate-6-semialdehyde decarboxylase |
| 83 | FOC4g04265 | Delta(12) fatty acid desaturase |
| 86 | FOC4g04268 | mRNA 3\'-end-processing protein YTH1 |
| 01 | FOC4g04284 | unknown protein |
| 05 | FOC4g04290 | acyl-CoA dehydrogenase, putative |
| 08 | FOC4g04293 | L-ascorbate oxidase |
| 09 | FOC4g04294 | GH31 α-1,6-glucosidase |
| 11 | FOC4g04296 | HET domain protein |
| 29 | FOC4g04314 | Ras-related protein Rab11D |
| 32 | FOC4g04317 | agmatinase |
| 34 | FOC4g04319 | unknown protein |
| 35 | FOC4g04320 | retinol dehydrogenase |
| 38 | FOC4g04323 | single stranded RNA-binding protein |
| 41 | FOC4g04326 | orphan protein |
| 42 | FOC4g04327 | MFS permease |
| 49 | FOC4g04335 | MFS Lactose permease |
| 51 | FOC4g04338 | unknown protein |
| 59 | FOC4g04346 | short chain dehydrogenase/reductase |
| 63 | FOC4g04351 | Thioredoxin reductase |
| 93 | FOC4g04384 | short chain dehydrogenase/reductase |
| 99 | FOC4g04391 | Alcohol oxidase |
| 16 | FOC4g04408 | ABC transporter B family member 6 |

| | | |
|---|---|---|
| 50 | FOC4g00194 | unknown protein |
| 51 | FOC4g00195 | unknown protein |
| 64 | FOC4g00399 | unknown protein |
| 76 | FOC4g00064 | MFS tetracyclin efflux permease |
| 77 | FOC4g00350 | unknown protein |
| 78 | FOC4g00351 | unknown protein |
| 42 | FOC4g04506 | peroxisomal catalase |
| 53 | FOC4g04518 | endoribonuclease L-PSP |
| 58 | FOC4g04524 | glutathione-dependent formaldehyde-activating enzyme |
| 78 | FOC4g04544 | Acyl-CoA dehydrogenase |
| 94 | FOC4g04559 | Holin-like protein CidB |
| 18 | FOC4g04584 | 5-methyltetrahydropteroyltriglutamate--homocysteine methyltransferase |
| 39 | FOC4g04605 | Histone-lysine N-methyltransferase |
| 44 | FOC4g04610 | MFS permease |
| 51 | FOC4g04617 | Non-specific lipid-transfer protein |
| 83 | FOC4g04649 | Cytochrome c |
| 10 | FOC4g04676 | 40S ribosomal protein S0 |
| 20 | FOC4g04686 | Urea amidolyase |
| 27 | FOC4g04693 | NADP-dependent oxidoreductase yfmJ |
| 33 | FOC4g04700 | RTA1 transmembrane protein; binds extracellular toxins |
| 35 | FOC4g04702 | retinol dehydrogenase |
| 01 | FOC4g04773 | unknown protein with Duf1479 domain |
| 21 | FOC4g04795 | Demethylmenaquinone methyltransferase |
| 29 | FOC4g01494 | non-LTR RNAse |
| 67 | FOC4g02053 | L-asparaginase |
| 68 | FOC4g02054 | unknown protein with DUF3429 |
| 71 | FOC4g02057 | unknown protein |
| 74 | FOC4g02060 | orphan protein |
| 78 | FOC4g02065 | oxysterol binding protein |
| 79 | FOC4g02066 | unknown protein with hAT dimerization domain |
| 85 | FOC4g02072 | amino acid permease |
| 86 | FOC4g02073 | 2OG (FeII)- Indoleamine 2,3-dioxygenase family protein |
| 95 | FOC4g02084 | unknown protein |
| 05 | FOC4g02094 | orphan protein |
| 07 | FOC4g02096 | Enoyl-[acyl-carrier-protein] reductase |
| 17 | FOC4g02106 | 30S ribosomal protein S17P-like protein |

| | | |
|---|---|---|
| 20 | FOC4g02109 | MFS alpha-glucoside permease |
| 21 | FOC4g02110 | GH31 α-1,6-glucosidase |
| 24 | FOC4g02113 | Zinc-dependent medium chain dehydrogenase/reductase |
| 26 | FOC4g02115 | HTH-type DNA-binding domain-containing acetyltransferase ybfA |
| 38 | FOC4g02128 | unknown protein with WD40 domains |
| 42 | FOC4g02132 | orphan protein |
| 43 | FOC4g02133 | 2,3-dihydroxybenzoate decarboxylase |
| 48 | FOC4g02138 | MFS quinate permease |
| 55 | FOC4g02145 | orphan protein |
| 61 | FOC4g02151 | unknown protein |
| 62 | FOC4g02152 | C2H2 transcriptional activator, uncertain |
| 95 | FOC4g02187 | HET domain protein |
| 06 | FOC4g02201 | Histidine kinase, part of a two component signal transduction system |
| 21 | FOC4g00782 | MFS permease, putative riboflavin transporter |
| 22 | FOC4g00783 | monocarboxylate transporter |
| 72 | FOC4g02800 | enoyl-CoA hydratase, mitochondrial |
| 83 | FOC4g02811 | GH105 d-4,5-unsaturated β-glucuronyl hydrolase |
| 84 | FOC4g02812 | Tip elongation aberrant protein 3 |
| 86 | FOC4g02814 | Thioredoxin reductase |
| 89 | FOC4g02818 | flavoprotein involved in K+ transport |
| 97 | FOC4g02826 | short chain dehydrogenase/reductase |
| 99 | FOC4g02829 | Dityrosine transporter 1 |
| 02 | FOC4g02832 | Stabilin-2 |
| 05 | FOC4g02836 | PKS, lovastatin nonaketide synthase |
| 11 | FOC4g02843 | Cytochrome P450-Salicylate hydroxylase |
| 13 | FOC4g02846 | orphan protein |
| 14 | FOC4g02847 | Epoxide hydrolase, soluble |
| 16 | FOC4g02850 | unknown protein |
| 22 | FOC4g02857 | short chain dehydrogenase/reductase |
| 26 | FOC4g02861 | Pyridine nucleotide-disulphide oxidoreductase, NAD-binding region |
| 36 | FOC4g02871 | GPCR Class B. secretin receptor |
| 41 | FOC4g02876 | NmrA-like transcription repressor |
| 44 | FOC4g02880 | O-acetyl-L-homoserine sulfhydrylase, related to S. cerevisiae Met17p |
| 47 | FOC4g02883 | 40S ribosomal protein S2 |
| 49 | FOC4g02885 | unknown protein with RNAse domain |
| 68 | FOC4g02905 | actin |

| | | |
|---|---|---|
| 77 | FOC4g02914 | unknown protein |
| 82 | FOC4g02919 | short-chain dehydrogenase/reductase |
| 93 | FOC4g02930 | Ankyrin |
| 98 | FOC4g02936 | serine racemase |
| 01 | FOC4g02939 | L-amino-acid oxidase |
| 17 | FOC4g02957 | AA2 GMC-oxidoreductase |
| 19 | FOC4g02959 | unknown protein |
| 32 | FOC4g02974 | Variant-surface-glycoprotein phospholipase C |
| 45 | FOC4g00200 | flavin-containing monooxygenase |
| 63 | FOC4g00235 | Catalase-peroxidase 2, partial |
| 78 | FOC4g01932 | HLH domain DNA binding protein |
| 82 | FOC4g01936 | O-methyltransferase, family 2 |
| 84 | FOC4g01938 | Carboxylyase-related; FMN-binding split barrel-related |
| 00 | FOC4g01953 | Anthranilate N-benzoyltransferase protein 1 |
| 05 | FOC4g01958 | non-LTR RNAse |
| 09 | FOC4g01962 | FAD-dependent oxidoreductase |
| 10 | FOC4g01963 | Amino acid permease |
| 14 | FOC4g01968 | GH28 endopolygalacturonase |
| 20 | FOC4g01975 | 30 kDa heat shock protein |
| 23 | FOC4g01978 | gluconolactonase |
| 36 | FOC4g01993 | AA2, FAD-dependent choline dehydrogenase |
| 37 | FOC4g01994 | unknown protein |
| 43 | FOC4g02001 | Alcohol dehydrogenase, iron-type |
| 46 | FOC4g02004 | Glucose/ribitol dehydrogenase; NmrA-like |
| 63 | FOC4g02021 | Zinc-dependent alcohol dehydrogenase |
| 95 | FOC4g02571 | helicase |
| 06 | FOC4g02581 | orphan protein |
| 07 | FOC4g02582 | orphan protein |
| 11 | FOC4g02586 | Zn2Cys6 transcriptional activator |
| 14 | FOC4g02589 | unknown protein |
| 15 | FOC4g02590 | unknown protein |
| 26 | FOC4g02602 | [illegible] enoyl reductase |
| 32 | FOC4g02608 | Cytochrome P450 monooxygenase |
| 52 | FOC4g02629 | short chain dehydrogenase/reductase |
| 58 | FOC4g02635 | acyl-CoA-cholesterin acetyltransferase |
| 64 | FOC4g02641 | Cytochrome P450 4F5 |

| | | |
|---|---|---|
| 73 | FOC4g02653 | Histidine kinase, part of a two component signal transduction system |
| 75 | FOC4g02655 | 60S ribosomal protein L43 |
| 86 | FOC4g02666 | Lipase |
| 87 | FOC4g02667 | unknown protein |
| 94 | FOC4g02674 | N-acyl homoserine lactonase AttM |
| 06 | FOC4g02686 | unknown protein with Duf342 domain |
| 10 | FOC4g02691 | Ergosterol biosynthesis protein 7-dehydrocholesterol reductase |
| 14 | FOC4g02695 | DNA polymerase III subunits alpha and tau |
| 16 | FOC4g02697 | GH31 α-1,6-glucosidase |
| 30 | FOC4g02711 | voltage-gated potassium channel subunit beta |
| 31 | FOC4g02712 | Monocarboxylate transporter |
| 38 | FOC4g02719 | Pisatin demethylase |
| 39 | FOC4g02720 | unknown protein with aldol condensation activity |
| 44 | FOC4g02725 | oligopeptide transporter, sexual differentiation process protein isp4 |
| 66 | FOC4g02748 | Succinate/fumarate mitochondrial transporter |
| 67 | FOC4g02750 | unknown protein |
| 68 | FOC4g02751 | Benzoylformate decarboxylase |
| 79 | FOC4g02762 | PTH11 receptor |
| 83 | FOC4g02766 | transmembrane protein, function unknown |
| 90 | FOC4g02773 | [illegible] response |
| 18 | FOC4g02419 | Ubiquinone/menaquinone biosynthesis methyltransferase ubiE |
| 20 | FOC4g02421 | epoxide hydrolase |
| 55 | FOC4g02466 | unknown protein with DUF3533 domain |
| 73 | FOC4g02486 | unknown protein with DUF676,PRELI |
| 95 | FOC4g02507 | Canalicular multispecific organic anion transporter 2 |
| 00 | FOC4g02513 | Cupin_5 |
| 01 | FOC4g02514 | unknown protein with DUF952 |
| 07 | FOC4g02520 | Cytochrome P450 monooxygenase |
| 08 | FOC4g02521 | TRAMP complex protein with RNA polymerase activity |
| 09 | FOC4g02523 | unknown protein with F-Box domain |
| 10 | FOC4g02524 | Cytochrome P450 4V3 |
| 11 | FOC4g02525 | Cytochrome P450- luciferin 4-monooxygenase |
| 21 | FOC4g02535 | Branched-chain-amino-acid aminotransferase, cytosolic |
| 23 | FOC4g02537 | orphan protein |
| 34 | FOC4g02549 | AA2, FAD-dependent choline dehydrogenase |
| 36 | FOC4g02551 | peroxidase family 2 |

| | | |
|---|---|---|
| [illegible] 53 | FOC4g00915 | NADH oxidase |
| [illegible] 55 | FOC4g00917 | FAD-containing monooxygenase EthA |
| [illegible] 56 | FOC4g00918 | HET domain protein |
| [illegible] 59 | FOC4g00921 | unknown protein |
| [illegible] 64 | FOC4g00926 | Aldehyde dehydrogenase |
| [illegible] 75 | FOC4g00937 | unknown protein |
| [illegible] 90 | FOC4g00952 | orphan protein |
| [illegible] 18 | FOC4g01025 | transposase IS4 |
| [illegible] 42 | FOC4g01051 | Sterol 24-C-methyltransferase |
| [illegible] 56 | FOC4g00134 | mRNA 3\'-end-processing protein YTH1 |
| [illegible] 98 | FOC4g00722 | glutathione S-transferase |
| [illegible] 06 | FOC4g00730 | amino acid permease,high-affinity methionine permease |
| [illegible] 21 | FOC4g00748 | Calcium/calmodulin-dependent protein kinase |
| [illegible] 38 | FOC4g00765 | [illegible] reductase |
| [illegible] 39 | FOC4g00766 | GH71 α-1,3-glucanase plus 2 CBM24 α-glucan binding domains |
| [illegible] 68 | FOC4g00631 | unknown protein |
